# Supplementary material for: Mapping and modeling the semantic space of math concepts
Source: Cognition. Author manuscript; Available in PMC 2025 Nov 26. (PMC7618406; doi:10.1016/j.cognition.2024.105971)
Supplement: Supplementary Material [file EMS210436-supplement-Supplementary_Material.zip › 1-s2.0-S0010027724002579-mmc1.pdf]

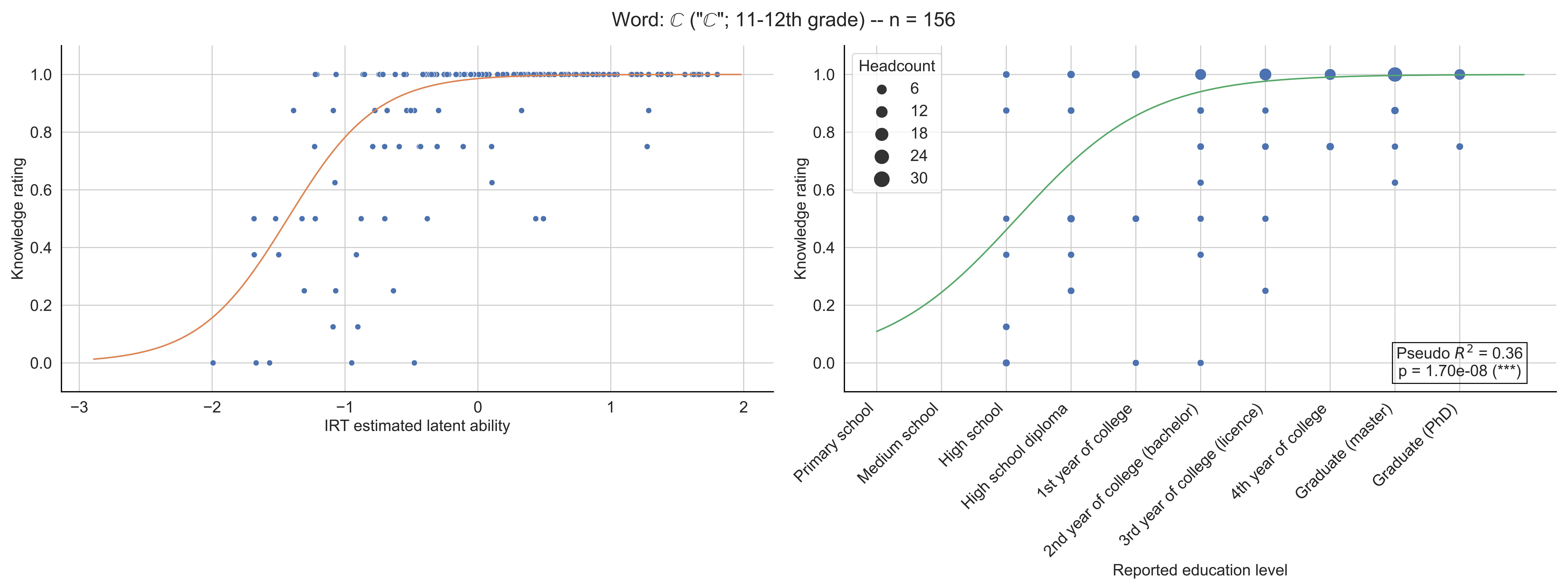

Word: ℂ ("ℂ"; 11-12th grade) -- n = 156
Knowledge rating
IRT estimated latent ability
Headcount
6
12
18
24
30
Pseudo R^2 = 0.36
p = 1.70e-08 (***)
Primary school
Medium school
High school
High school diploma
1st year of college
2nd year of college (bachelor)
3rd year of college (licence)
4th year of college
Graduate (master)
Graduate (PhD)
Reported education level

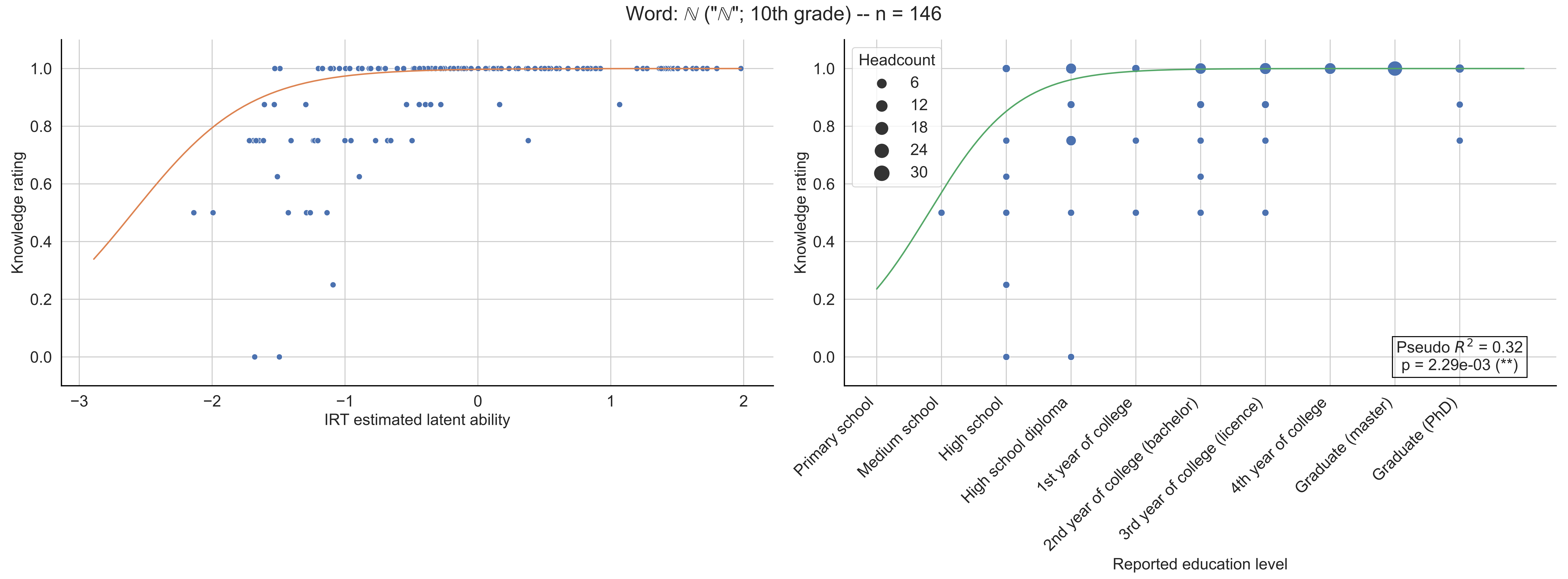
Word: ℕ ("ℕ"; 10th grade) -- n = 146
Knowledge rating
IRT estimated latent ability
Headcount
6
12
18
24
30
Pseudo R^2 = 0.32
p = 2.29e-03 (**)
Primary school
Medium school
High school
High school diploma
1st year of college
2nd year of college (bachelor)
3rd year of college (licence)
4th year of college
Graduate (master)
Graduate (PhD)
Reported education level

Word: $\mathbb{Q}$ ("$\mathbb{Q}$"; 10th grade) -- n = 152

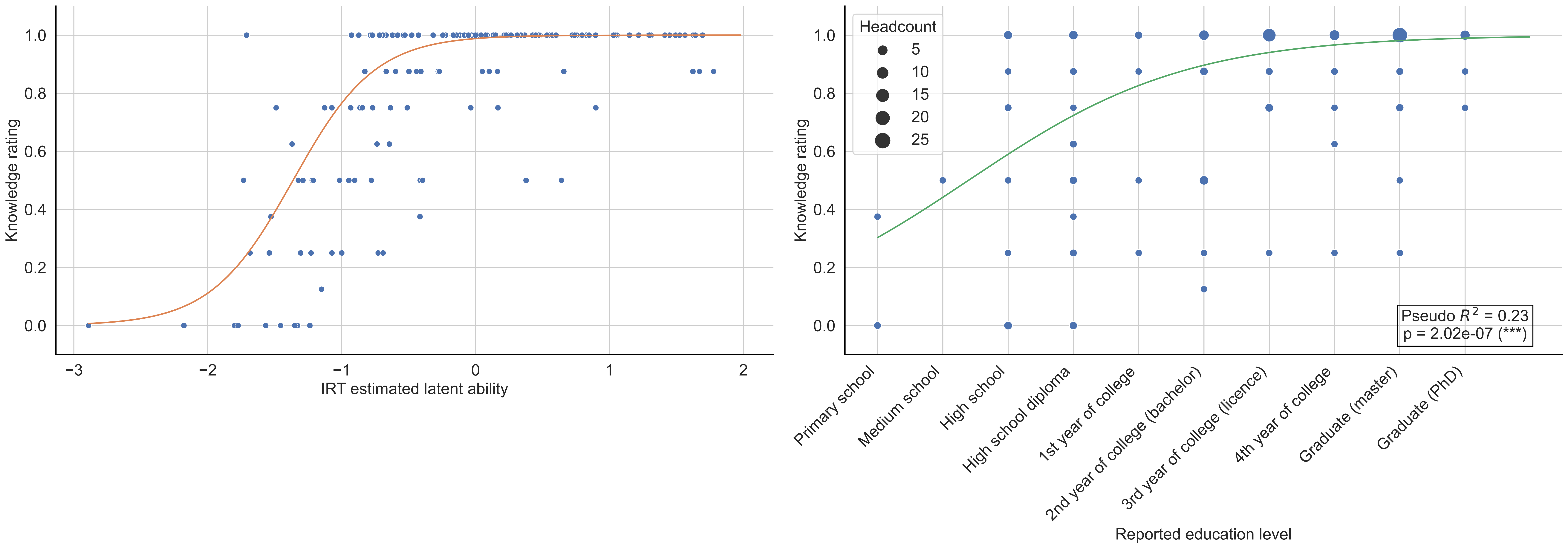

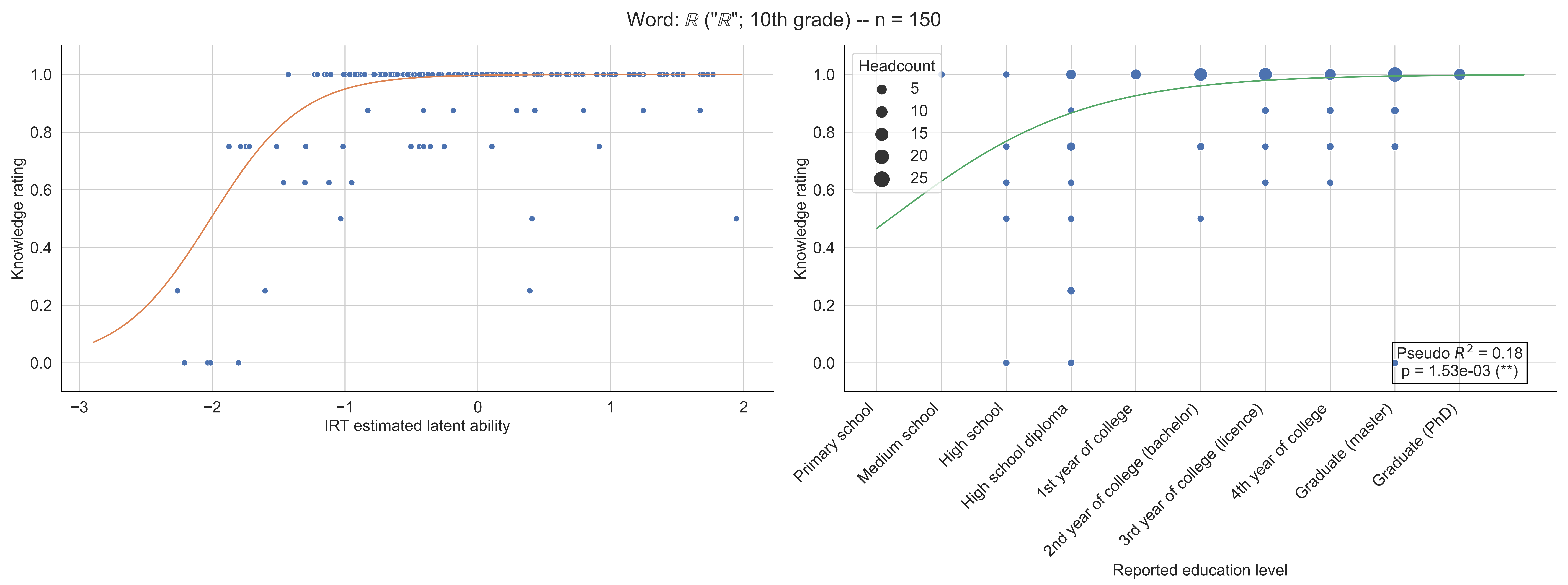
Word: ℝ ("ℝ"; 10th grade) -- n = 150
Knowledge rating
IRT estimated latent ability
Headcount
5
10
15
20
25
Pseudo R^2 = 0.18
p = 1.53e-03 (**)
Primary school
Medium school
High school
High school diploma
1st year of college
2nd year of college (bachelor)
3rd year of college (licence)
4th year of college
Graduate (master)
Graduate (PhD)
Reported education level

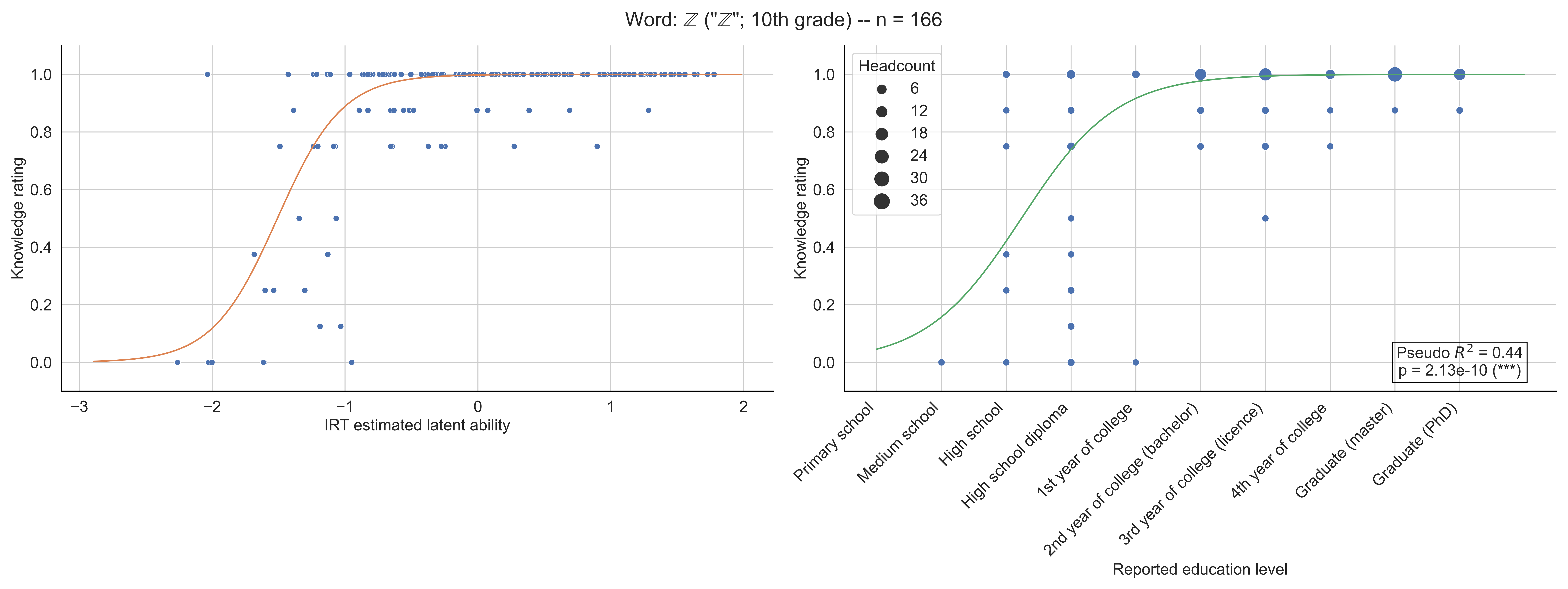
Word: ℤ ("ℤ"; 10th grade) -- n = 166
Knowledge rating
IRT estimated latent ability
Headcount
6
12
18
24
30
36
Pseudo R^2 = 0.44
p = 2.13e-10 (***)
Primary school
Medium school
High school
High school diploma
1st year of college
2nd year of college (bachelor)
3rd year of college (licence)
4th year of college
Graduate (master)
Graduate (PhD)
Reported education level

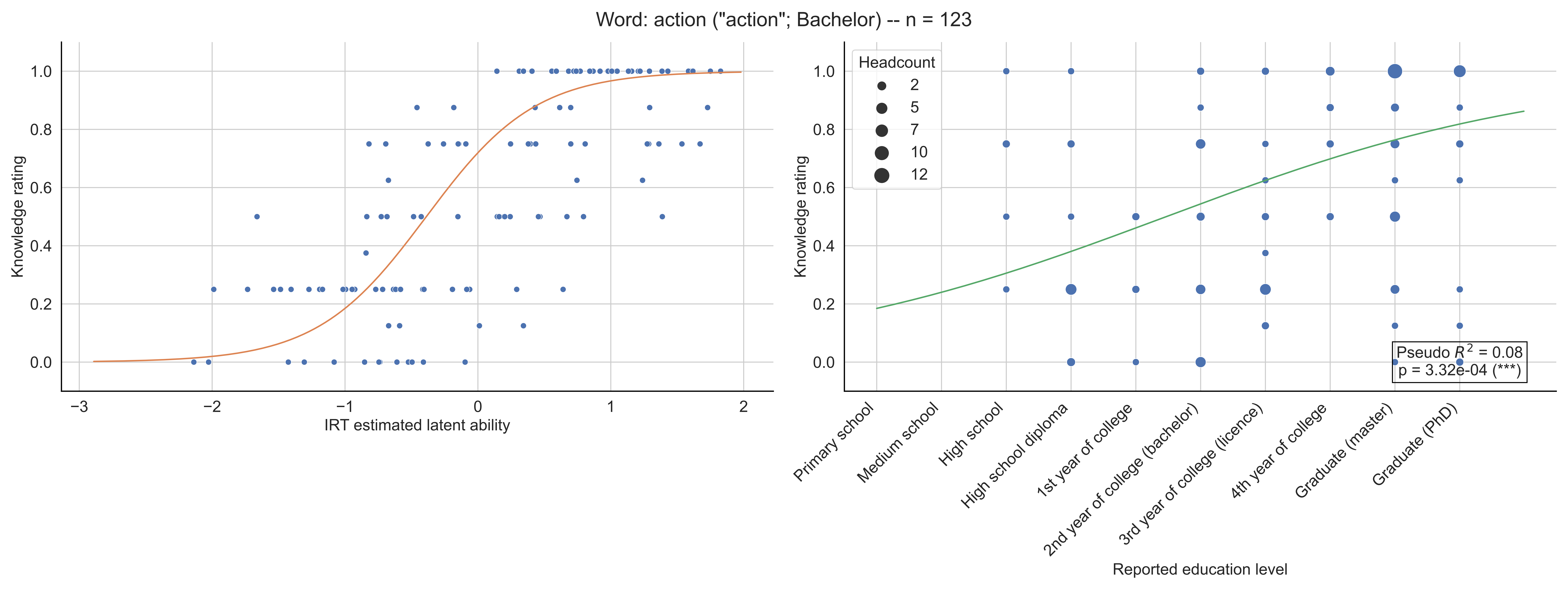

Word: action ("action"; Bachelor) -- n = 123
Knowledge rating
IRT estimated latent ability
Headcount
2
5
7
10
12
Pseudo R^2 = 0.08
p = 3.32e-04 (***)
Primary school
Medium school
High school
High school diploma
1st year of college
2nd year of college (bachelor)
3rd year of college (licence)
4th year of college
Graduate (master)
Graduate (PhD)
Reported education level

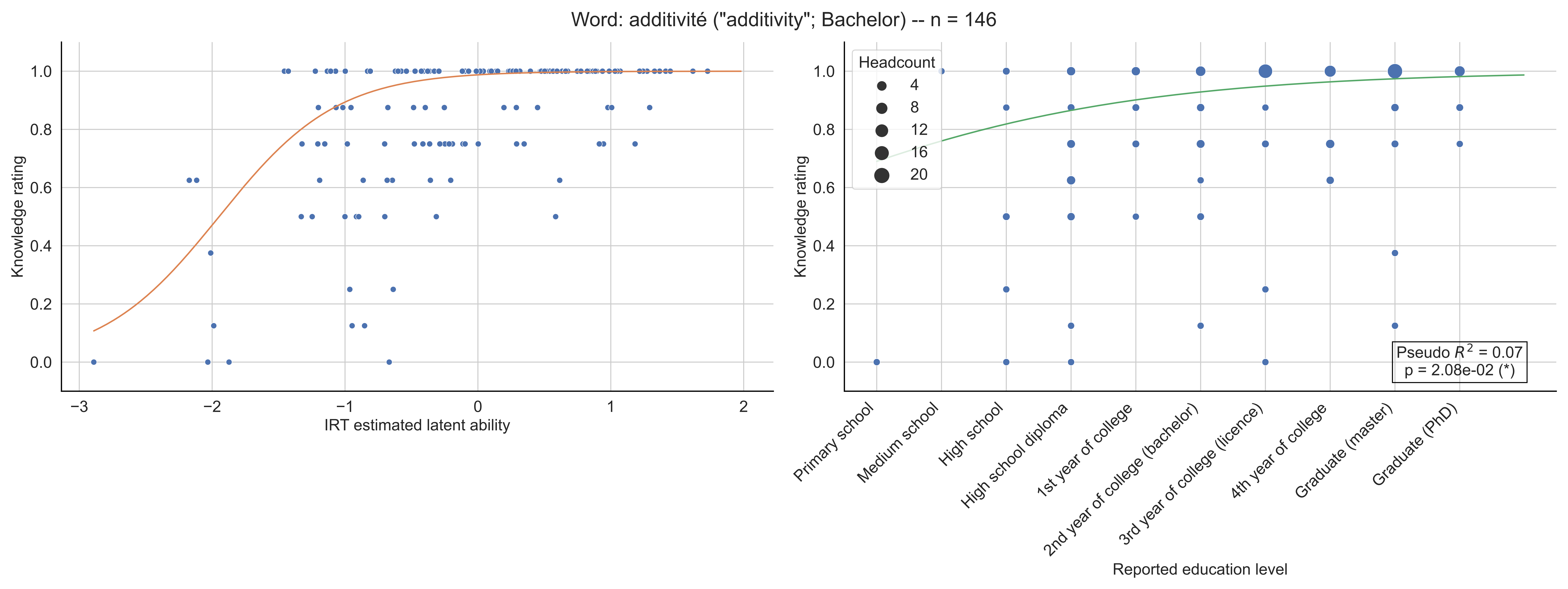

Word: additivité ("additivity"; Bachelor) -- n = 146
Knowledge rating
IRT estimated latent ability
Headcount
4
8
12
16
20
Pseudo R^2 = 0.07
p = 2.08e-02 (*)
Primary school
Medium school
High school
High school diploma
1st year of college
2nd year of college (bachelor)
3rd year of college (licence)
4th year of college
Graduate (master)
Graduate (PhD)
Reported education level

Word: adjoint ("adjoint"; Bachelor) -- n = 158

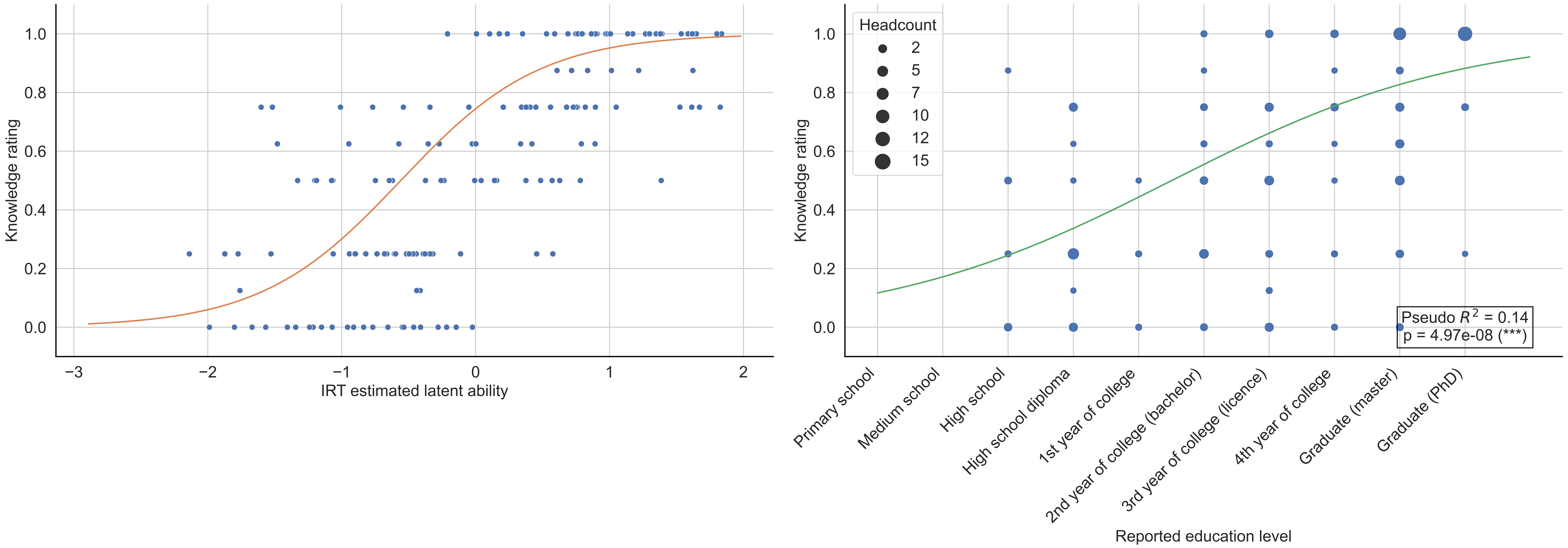

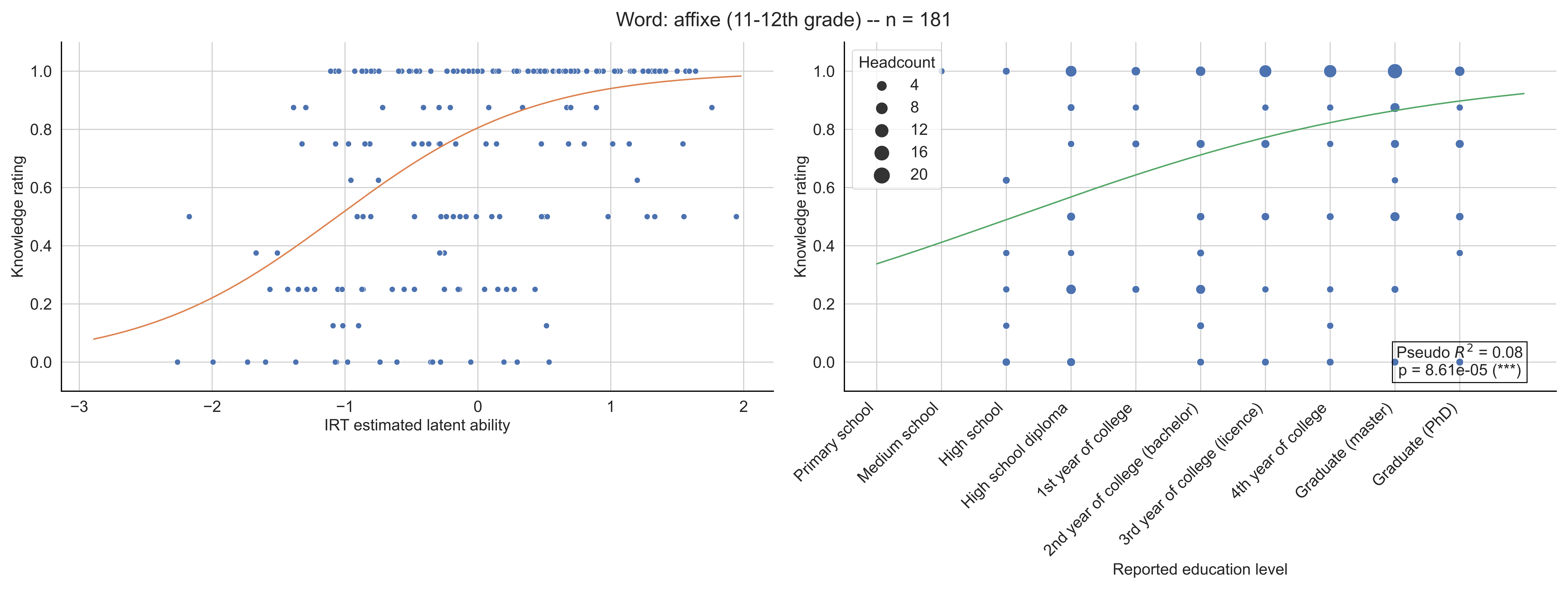

Word: affixe (11-12th grade) -- n = 181
Knowledge rating
IRT estimated latent ability
Headcount
4
8
12
16
20
Pseudo R^2 = 0.08
p = 8.61e-05 (***)
Primary school
Medium school
High school
High school diploma
1st year of college
2nd year of college (bachelor)
3rd year of college (licence)
4th year of college
Graduate (master)
Graduate (PhD)
Reported education level

Word: algorithmique ("algorithmic"; 6-7th grade) -- n = 139

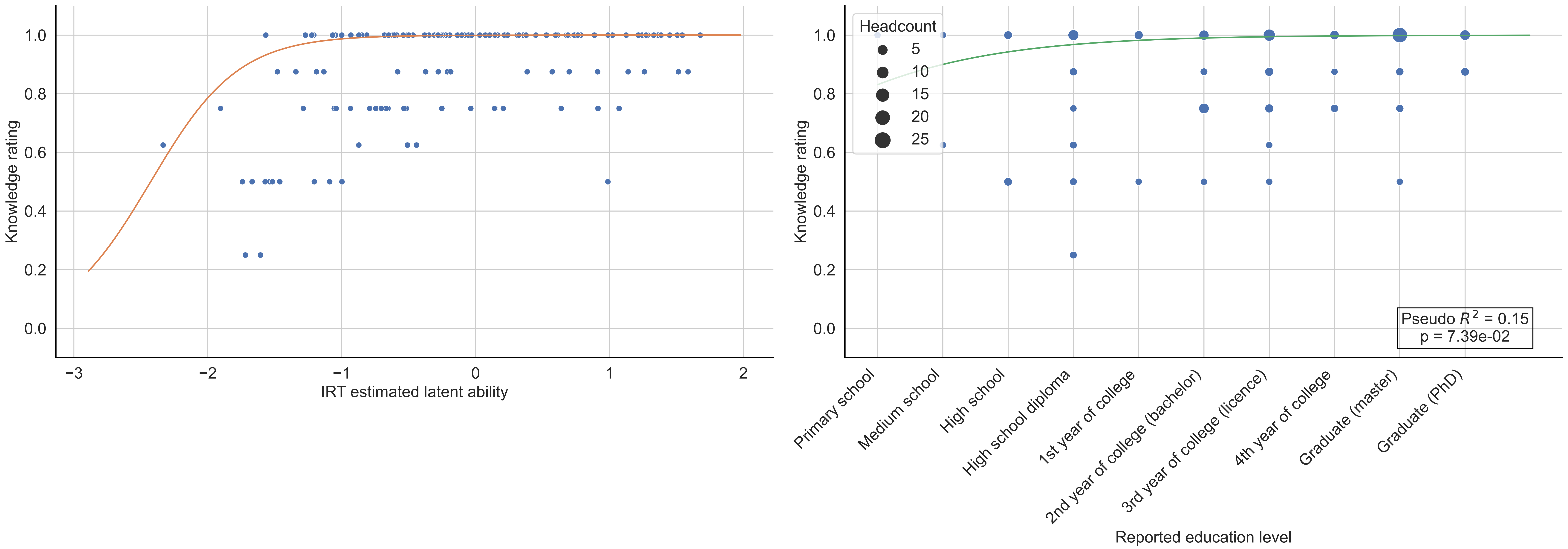

Word: anneau ("ring"; Bachelor) -- n = 168

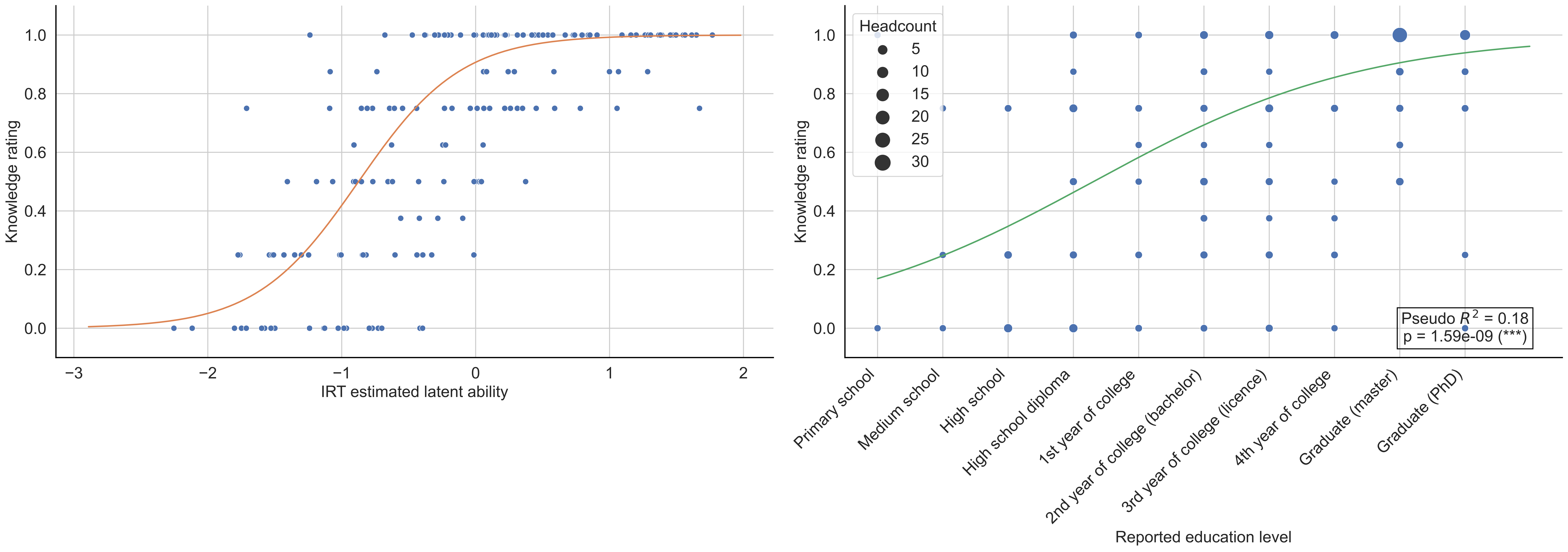

Word: application ("map"; Bachelor) -- n = 162

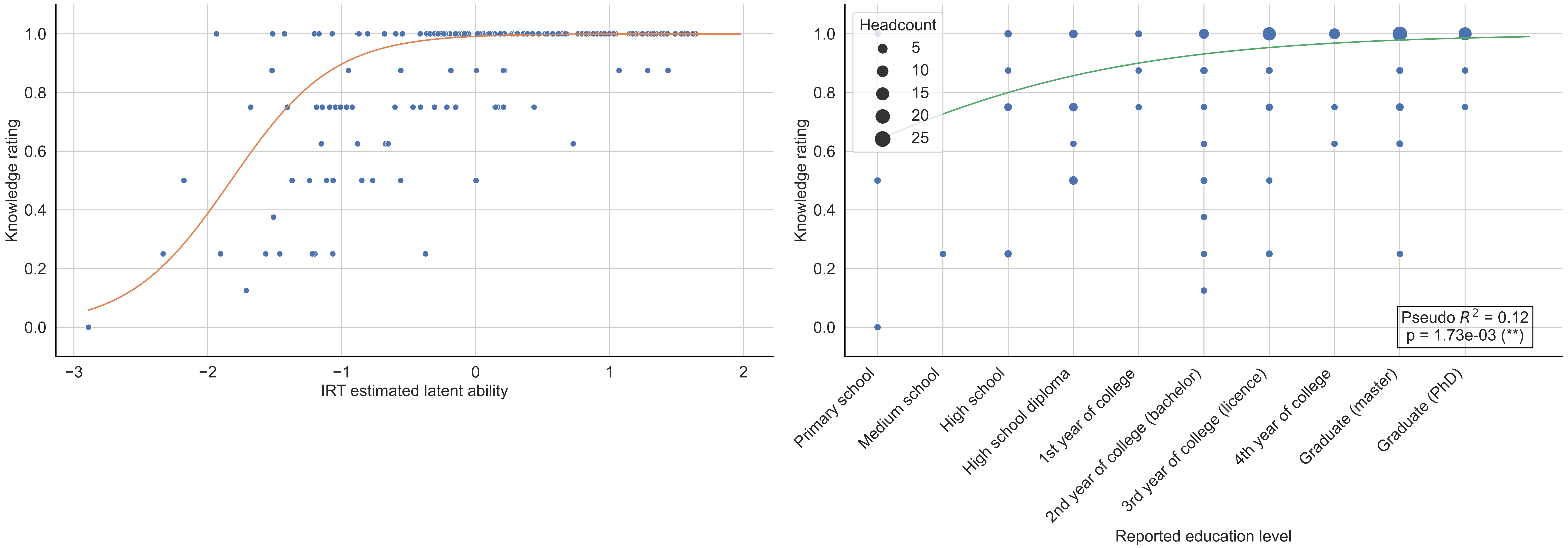

Word: arbre ("tree"; 10th grade) -- n = 134

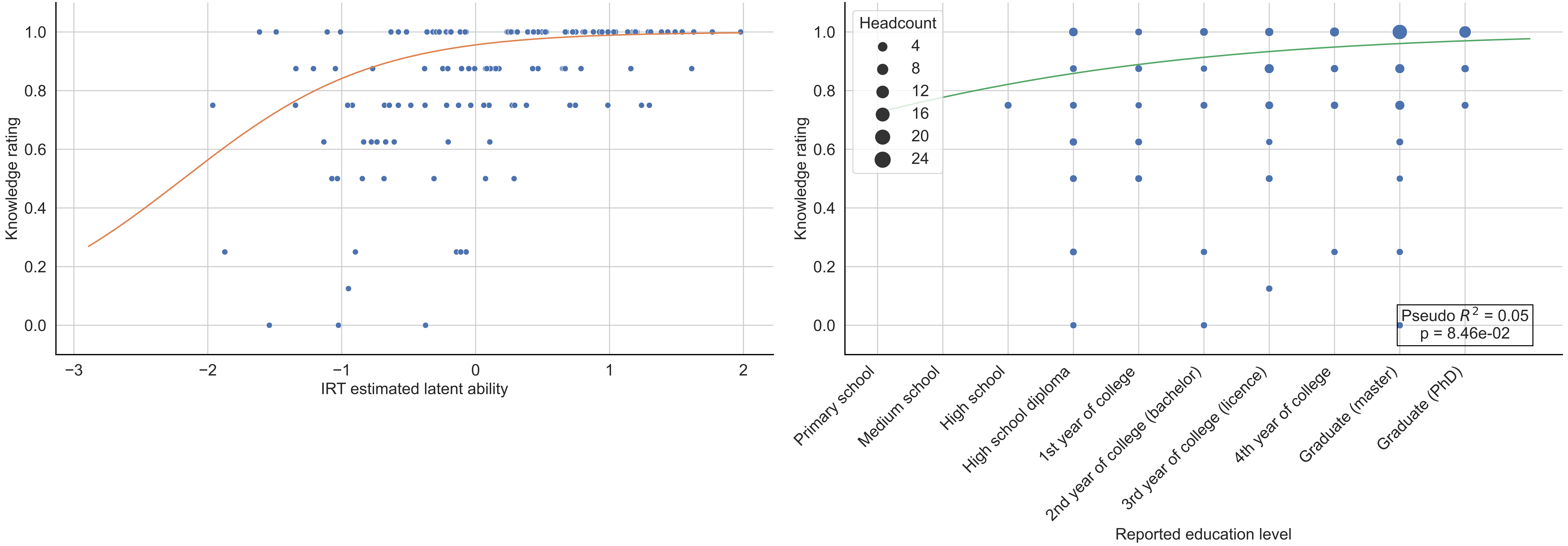

Word: arc ("arc"; 6-7th grade) -- n = 150

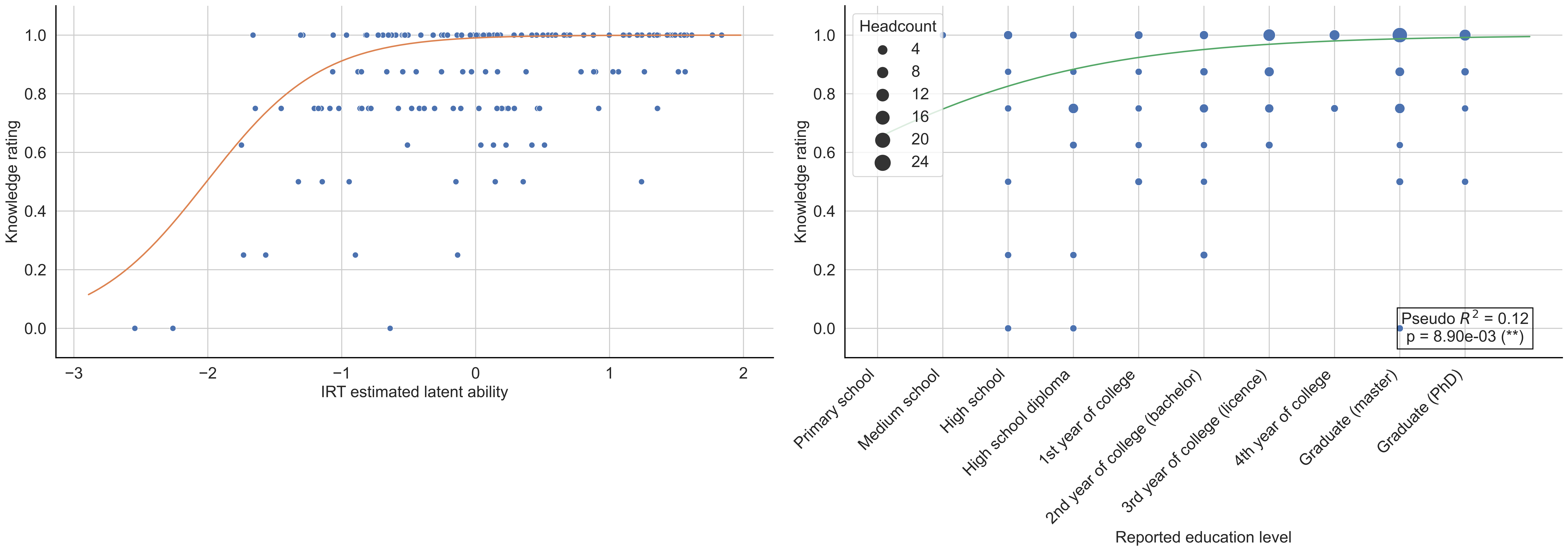

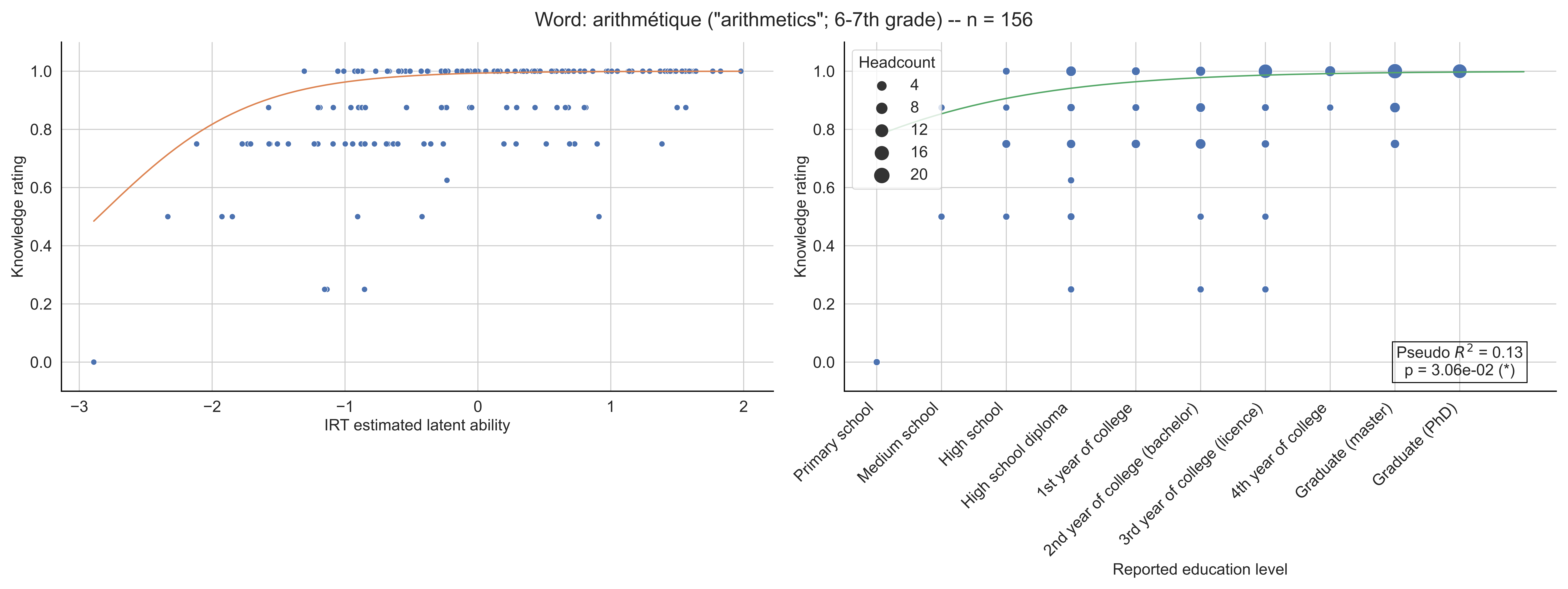

Word: arithmétique ("arithmetics"; 6-7th grade) -- n = 156
Knowledge rating
IRT estimated latent ability
Headcount
4
8
12
16
20
Primary school
Medium school
High school
High school diploma
1st year of college
2nd year of college (bachelor)
3rd year of college (licence)
4th year of college
Graduate (master)
Graduate (PhD)
Reported education level
Pseudo R^2 = 0.13
p = 3.06e-02 (*)

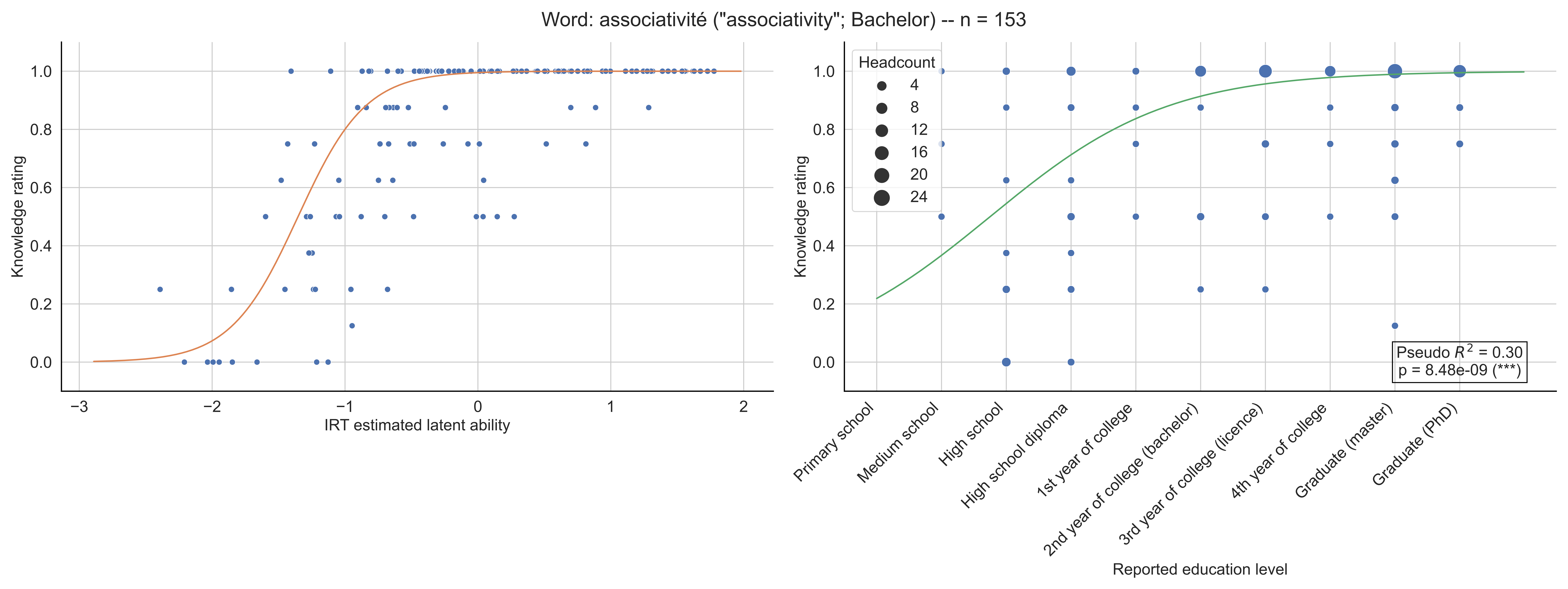

Word: associativité ("associativity"; Bachelor) -- n = 153
Knowledge rating
IRT estimated latent ability
Headcount
4
8
12
16
20
24
Pseudo R^2 = 0.30
p = 8.48e-09 (***)
Primary school
Medium school
High school
High school diploma
1st year of college
2nd year of college (bachelor)
3rd year of college (licence)
4th year of college
Graduate (master)
Graduate (PhD)
Reported education level

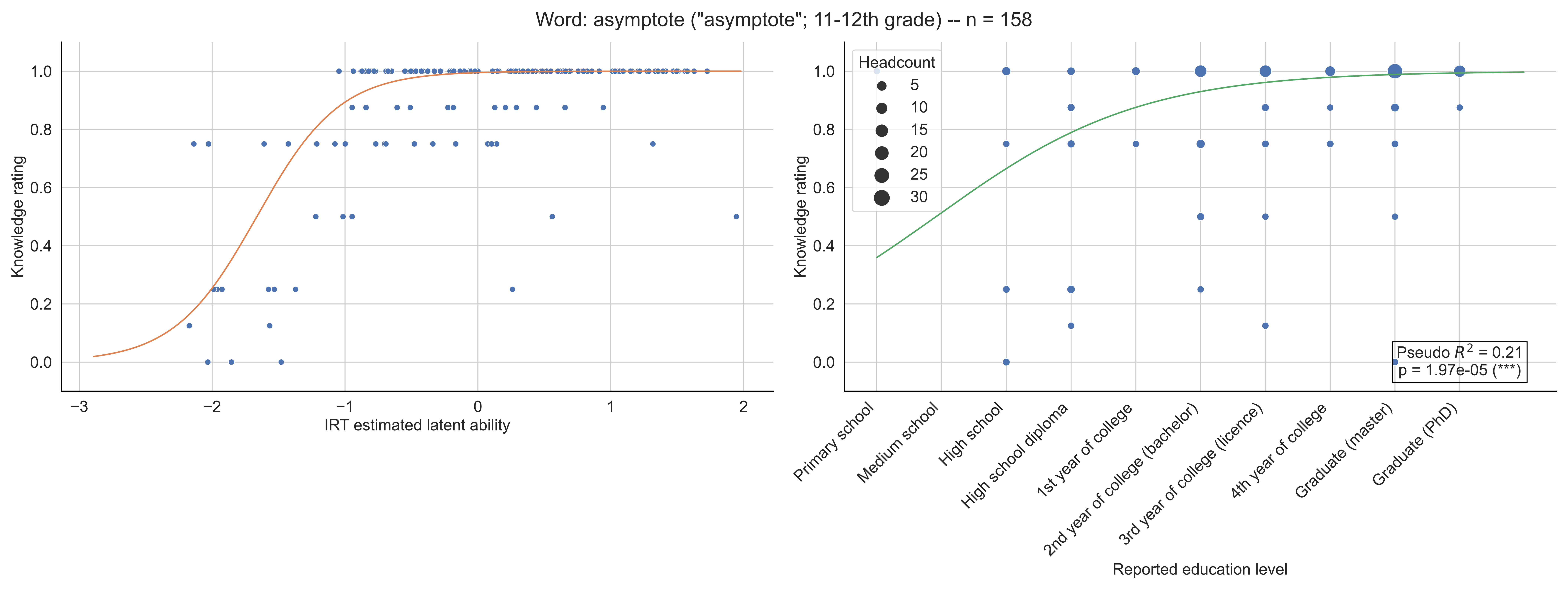
Word: asymptote ("asymptote"; 11-12th grade) -- n = 158
Knowledge rating
IRT estimated latent ability
Headcount
5
10
15
20
25
30
Primary school
Medium school
High school
High school diploma
1st year of college
2nd year of college (bachelor)
3rd year of college (licence)
4th year of college
Graduate (master)
Graduate (PhD)
Reported education level
Pseudo R^2 = 0.21
p = 1.97e-05 (***)

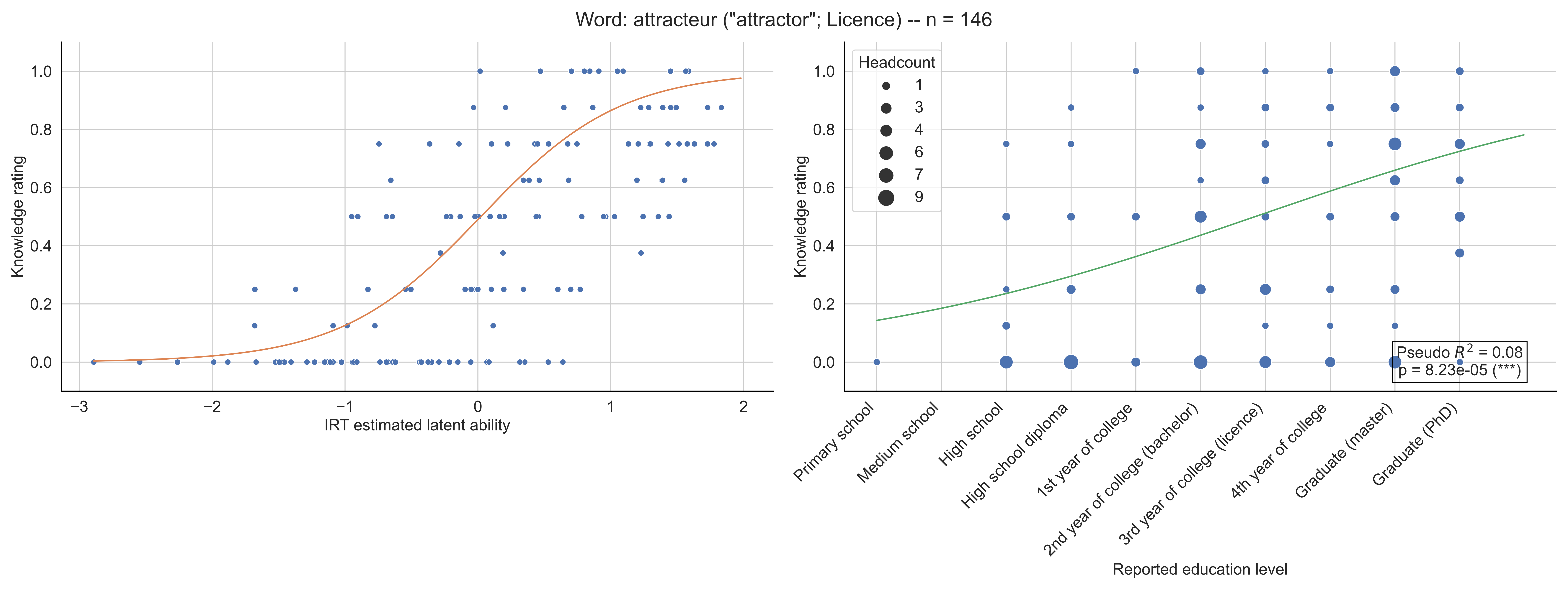

Word: attracteur ("attractor"; Licence) -- n = 146
Knowledge rating
IRT estimated latent ability
Headcount
1
3
4
6
7
9
Pseudo R^2 = 0.08
p = 8.23e-05 (***)
Primary school
Medium school
High school
High school diploma
1st year of college
2nd year of college (bachelor)
3rd year of college (licence)
4th year of college
Graduate (master)
Graduate (PhD)
Reported education level

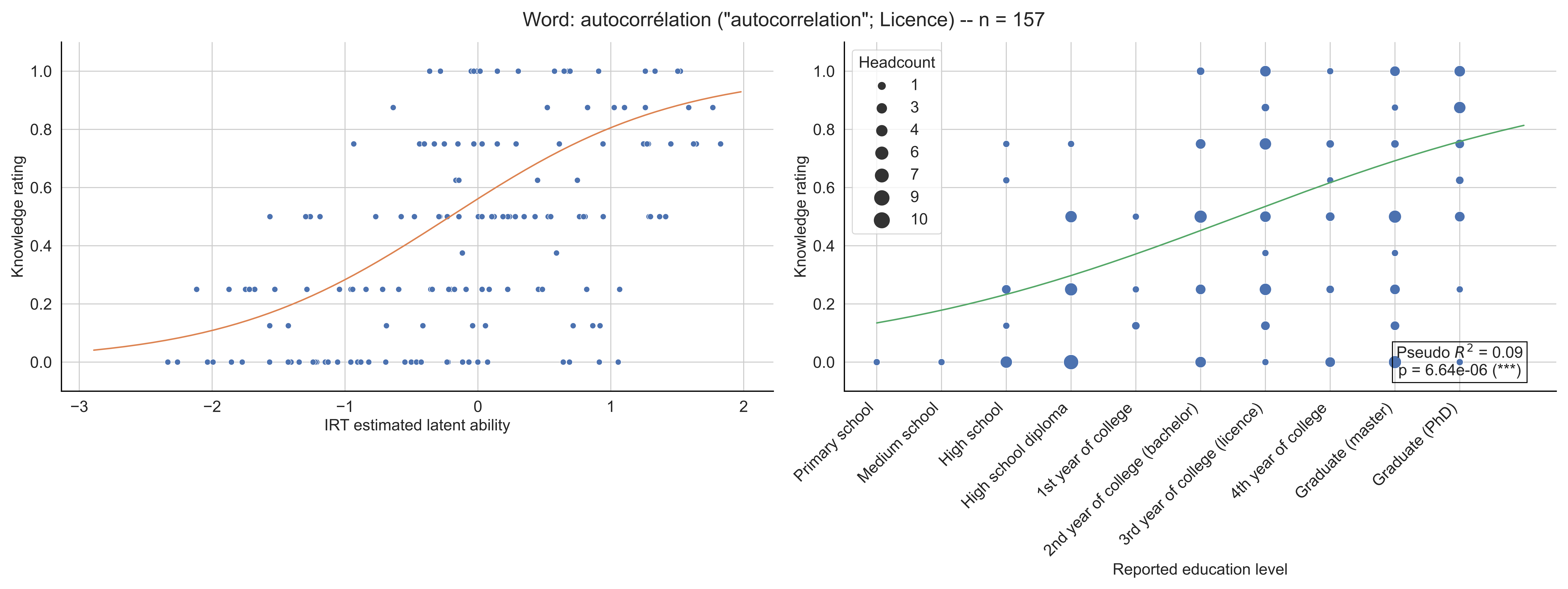

Word: autocorrélation ("autocorrelation"; Licence) -- n = 157
Knowledge rating
IRT estimated latent ability
Headcount
1
3
4
6
7
9
10
Pseudo R^2 = 0.09
p = 6.64e-06 (***)
Primary school
Medium school
High school
High school diploma
1st year of college
2nd year of college (bachelor)
3rd year of college (licence)
4th year of college
Graduate (master)
Graduate (PhD)
Reported education level

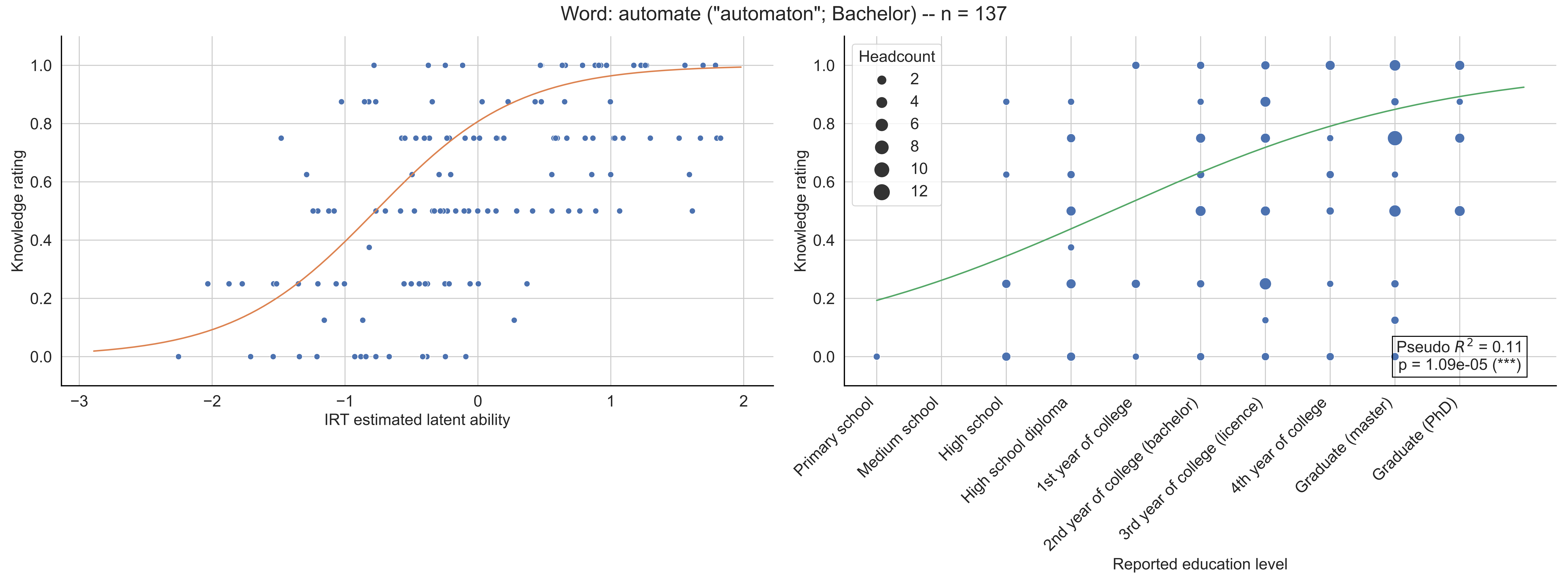

Word: automate ("automaton"; Bachelor) -- n = 137
Knowledge rating
IRT estimated latent ability
Headcount
2
4
6
8
10
12
Primary school
Medium school
High school
High school diploma
1st year of college
2nd year of college (bachelor)
3rd year of college (licence)
4th year of college
Graduate (master)
Graduate (PhD)
Reported education level
Pseudo R^2 = 0.11
p = 1.09e-05 (***)

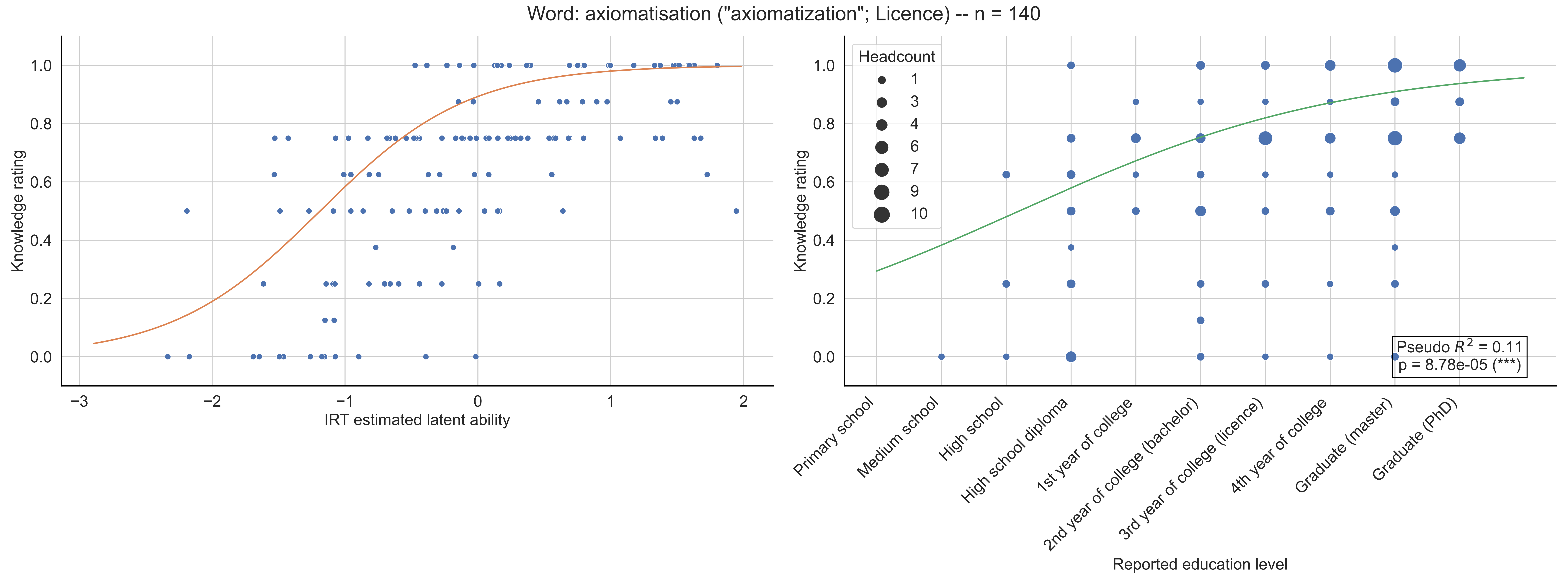

Word: axiomatisation ("axiomatization"; Licence) -- n = 140
Knowledge rating
IRT estimated latent ability
Headcount
1
3
4
6
7
9
10
Pseudo R^2 = 0.11
p = 8.78e-05 (***)
Primary school
Medium school
High school
High school diploma
1st year of college
2nd year of college (bachelor)
3rd year of college (licence)
4th year of college
Graduate (master)
Graduate (PhD)
Reported education level

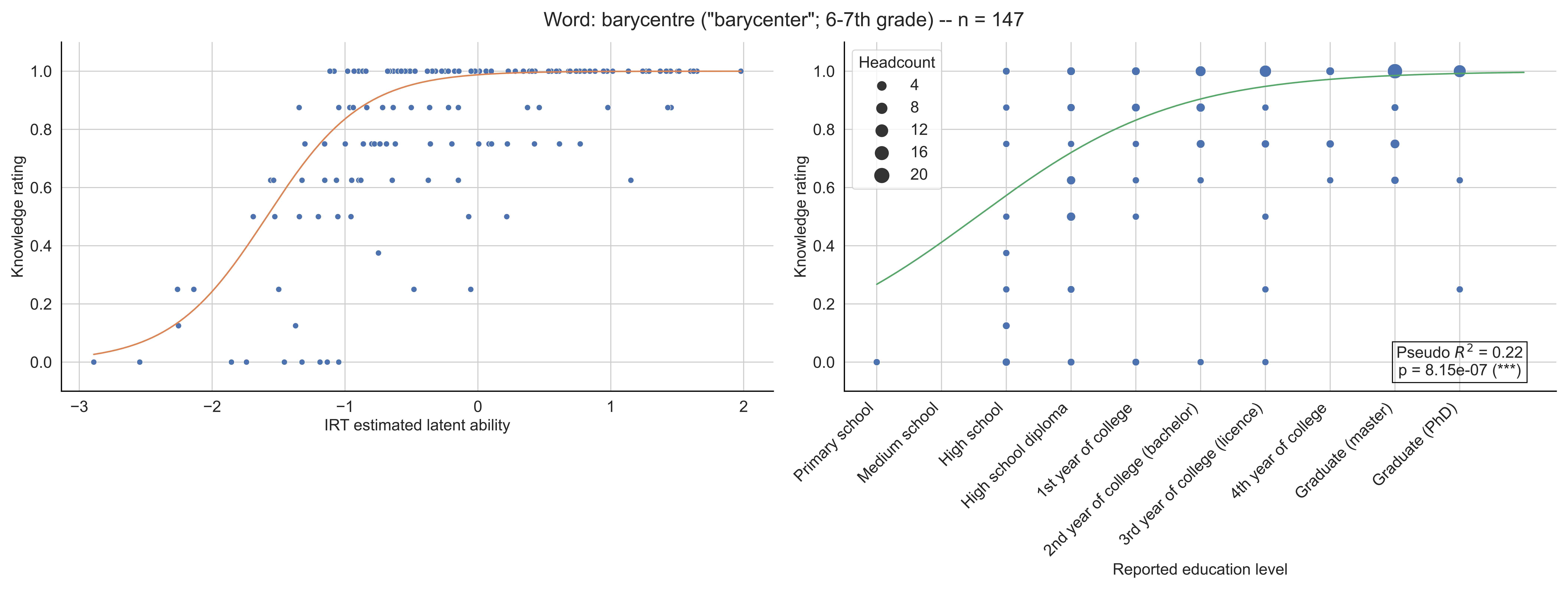

Word: barycentre ("barycenter"; 6-7th grade) -- n = 147
Knowledge rating
IRT estimated latent ability
Headcount
4
8
12
16
20
Pseudo R^2 = 0.22
p = 8.15e-07 (***)
Primary school
Medium school
High school
High school diploma
1st year of college
2nd year of college (bachelor)
3rd year of college (licence)
4th year of college
Graduate (master)
Graduate (PhD)
Reported education level

Word: bilinéaire ("bilinear"; Bachelor) -- n = 137

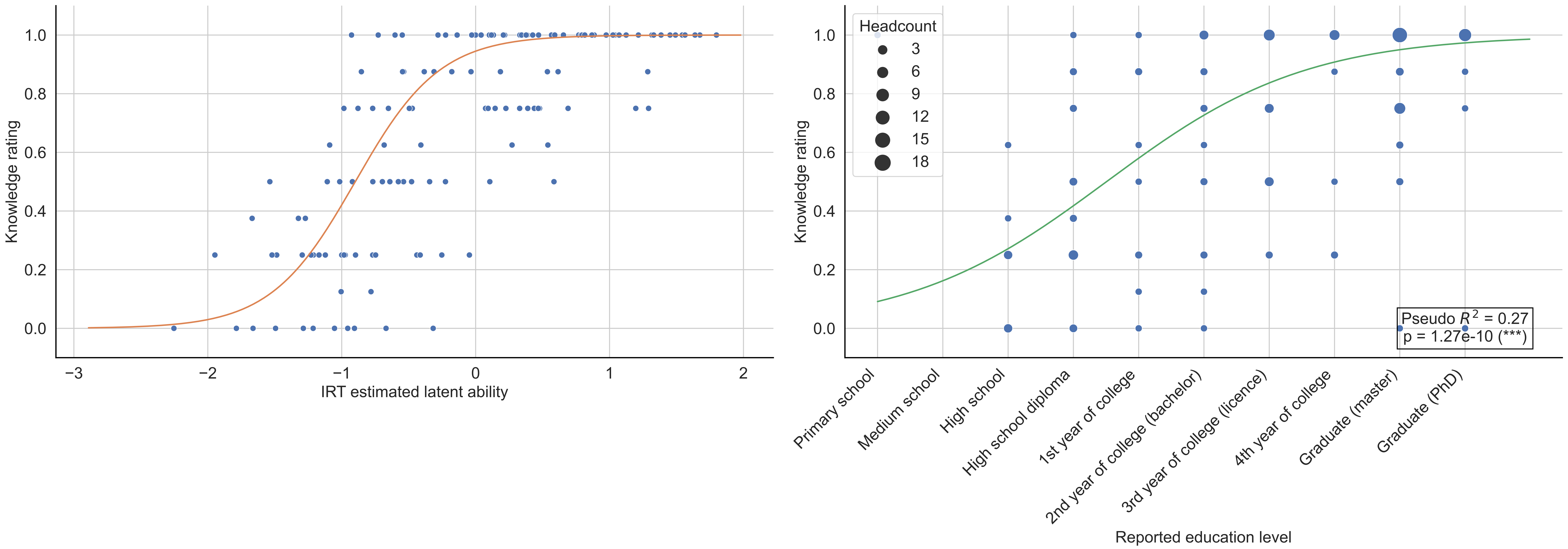

Word: binaire ("binary"; 10th grade) -- n = 148

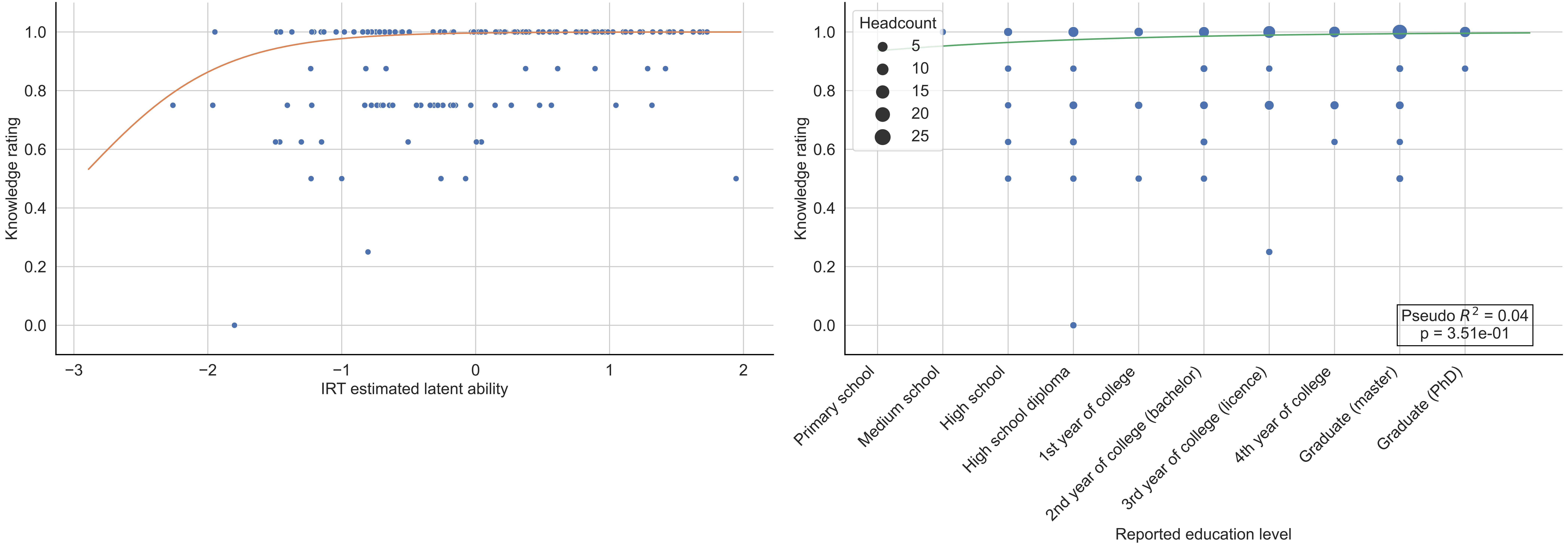

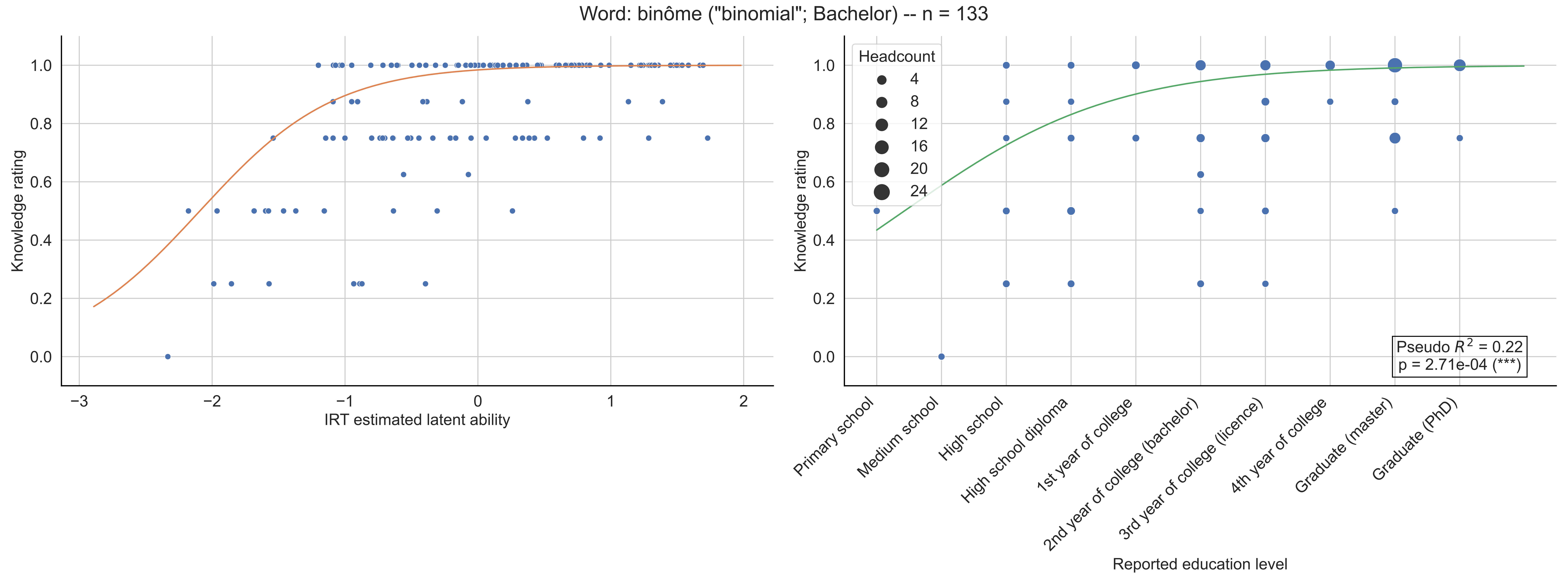

Word: binôme ("binomial"; Bachelor) -- n = 133
Knowledge rating
IRT estimated latent ability
Headcount
4
8
12
16
20
24
Pseudo R^2 = 0.22
p = 2.71e-04 (***)
Primary school
Medium school
High school
High school diploma
1st year of college
2nd year of college (bachelor)
3rd year of college (licence)
4th year of college
Graduate (master)
Graduate (PhD)
Reported education level

Word: bissectrice ("bisector"; Primary school) -- n = 136

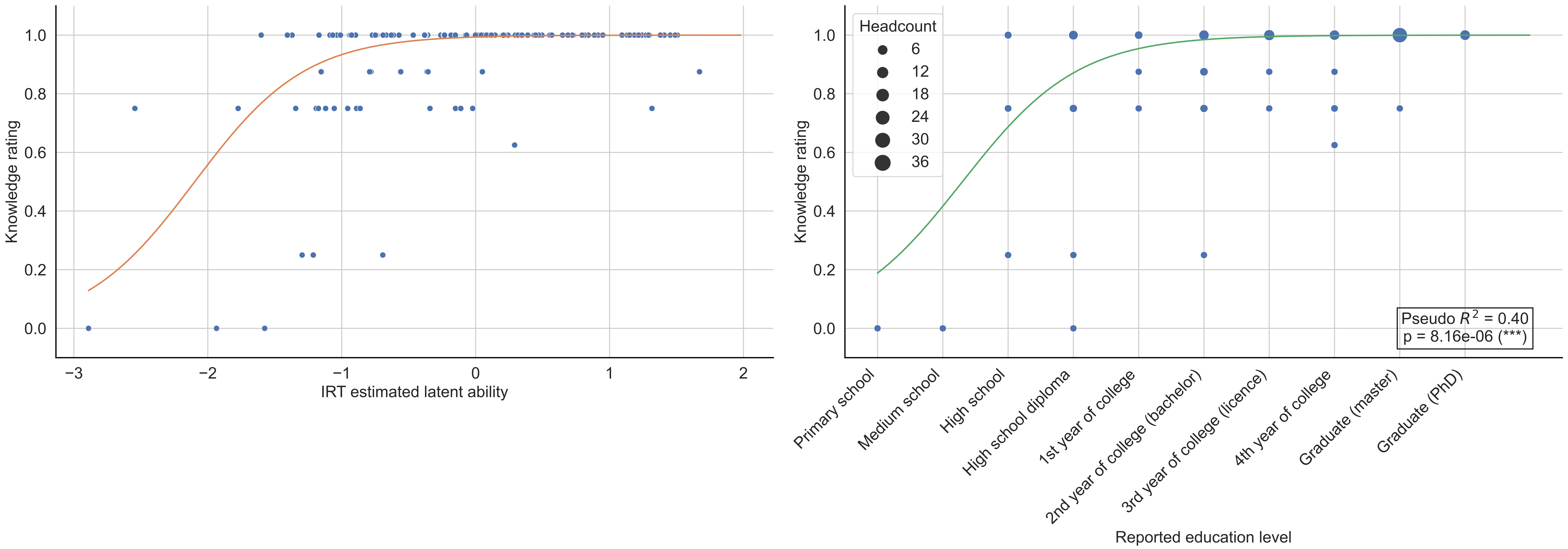

Word: booléen ("boolean"; 10th grade) -- n = 151

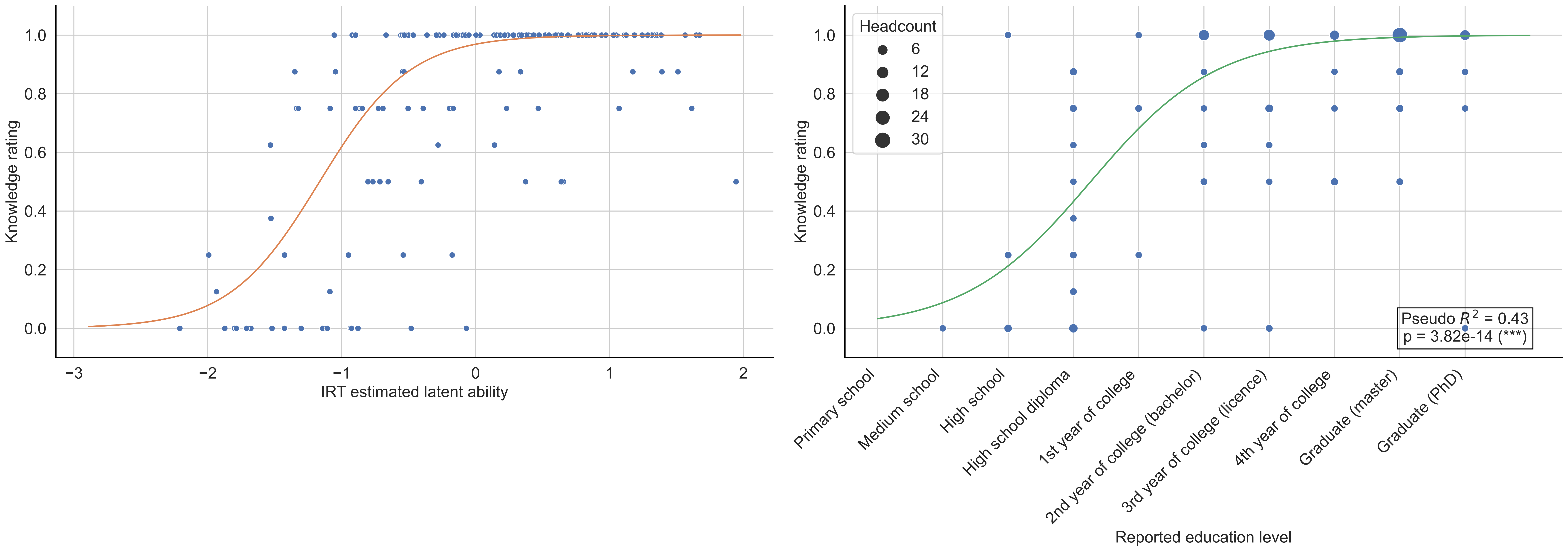

Word: borne ("bound"; 11-12th grade) -- n = 159

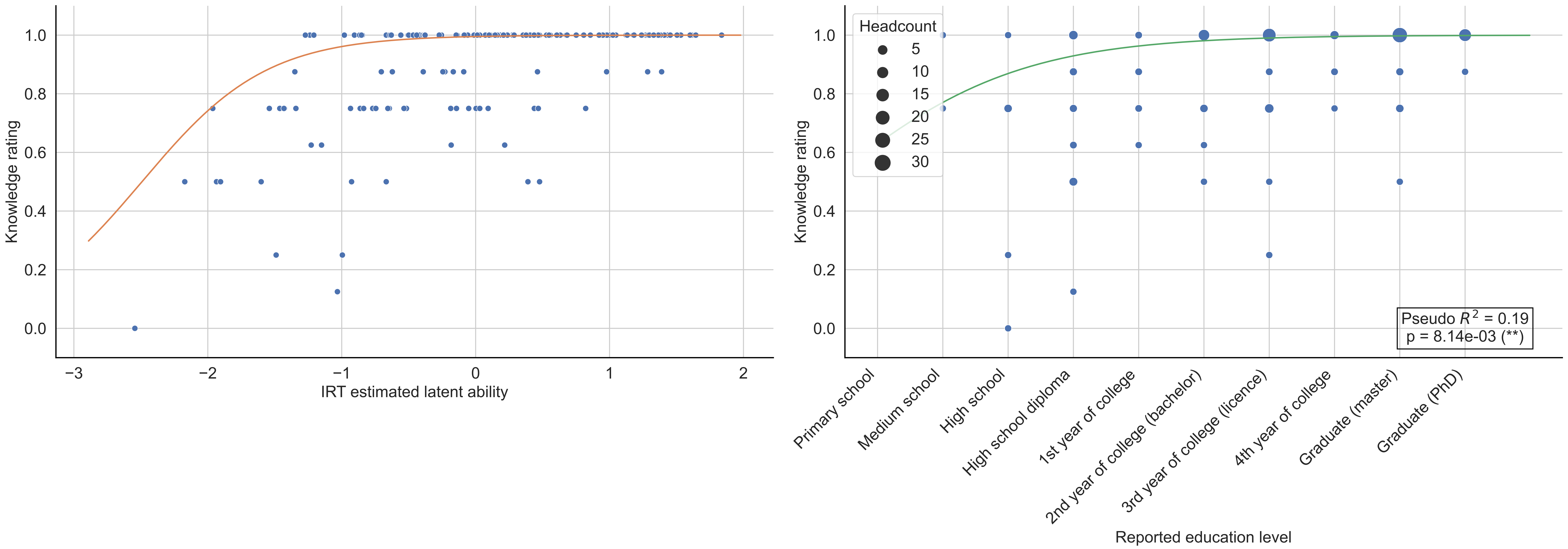

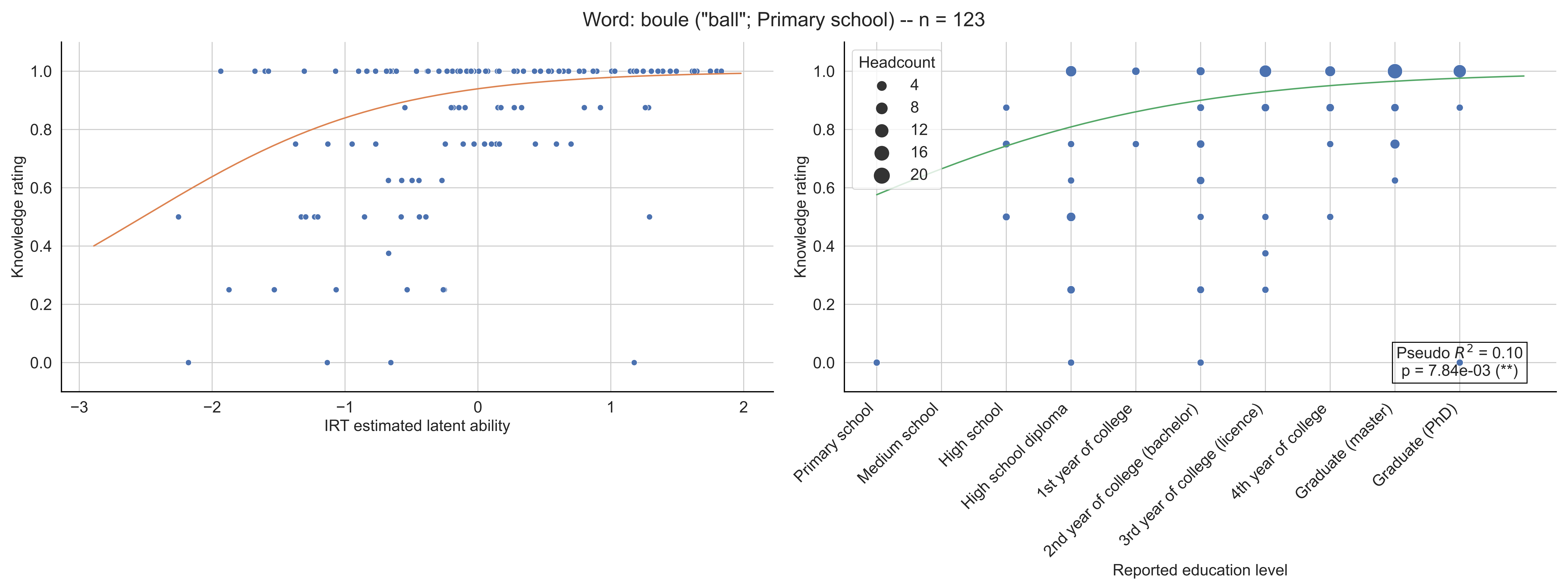

Word: boule ("ball"; Primary school) -- n = 123
Knowledge rating
IRT estimated latent ability
Headcount
4
8
12
16
20
Pseudo R^2 = 0.10
p = 7.84e-03 (**)
Primary school
Medium school
High school
High school diploma
1st year of college
2nd year of college (bachelor)
3rd year of college (licence)
4th year of college
Graduate (master)
Graduate (PhD)
Reported education level

Word: calculabilité ("computability"; Licence) -- n = 137

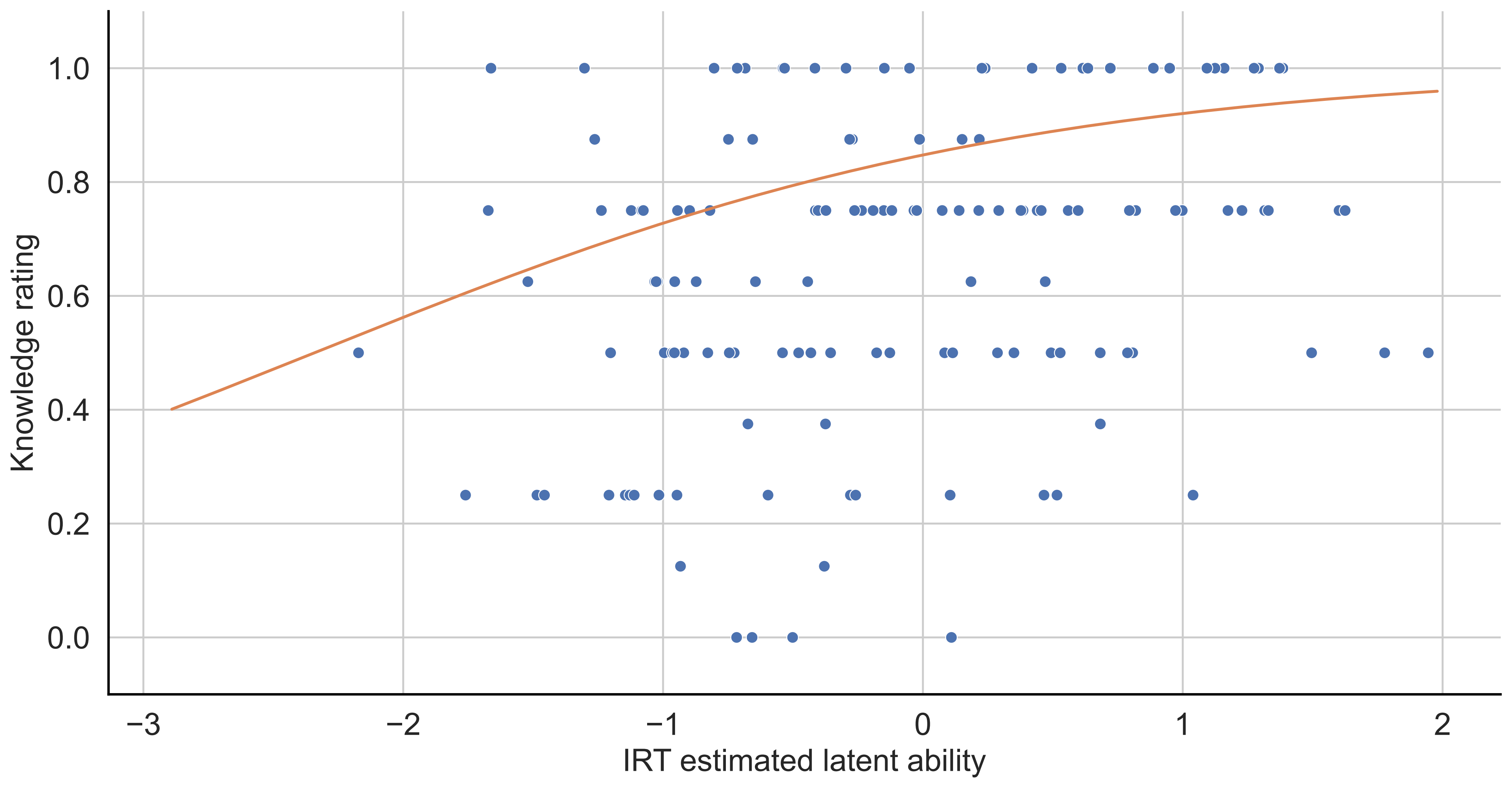

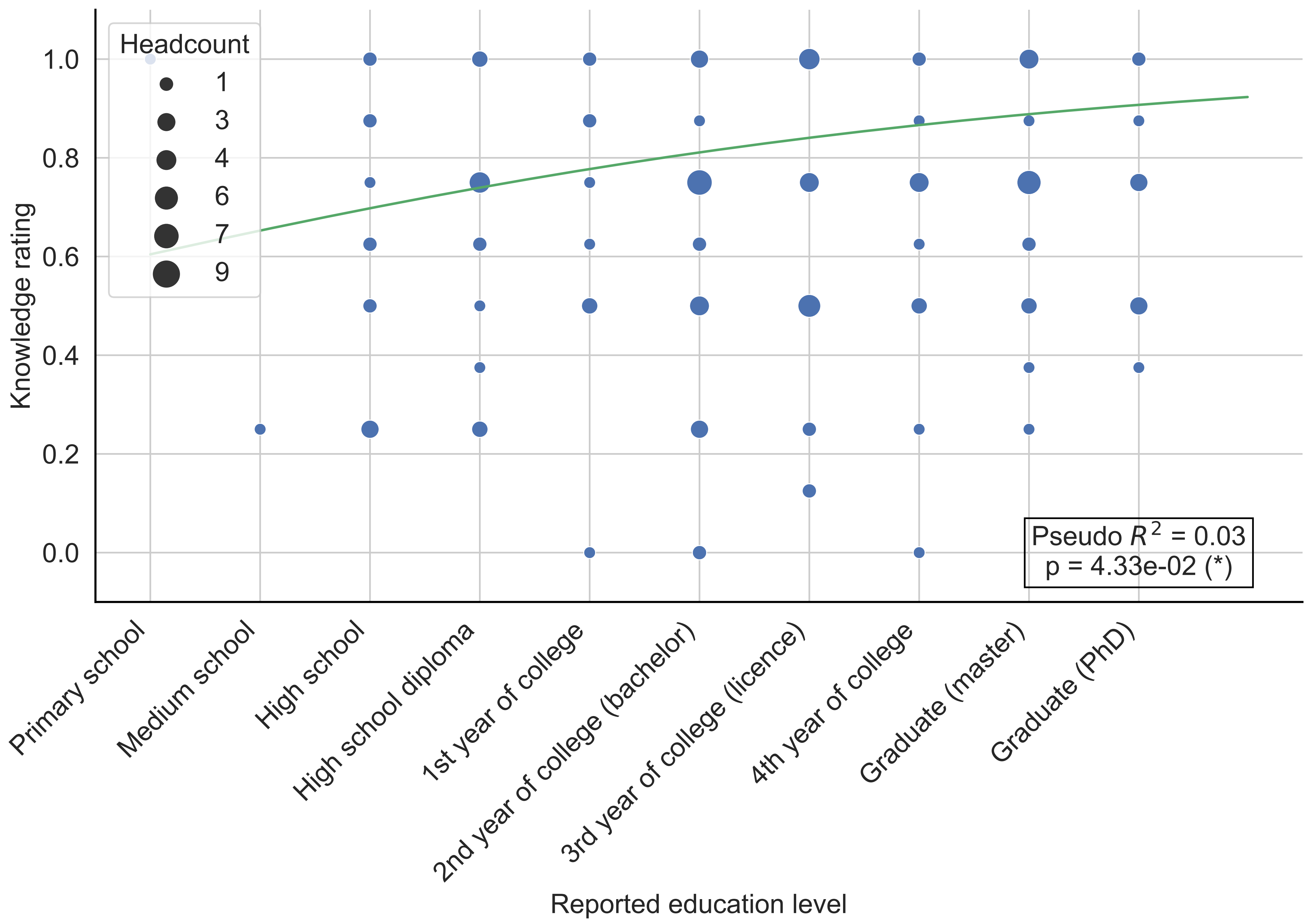

Word: caractéristique ("characteristic"; Bachelor) -- n = 126

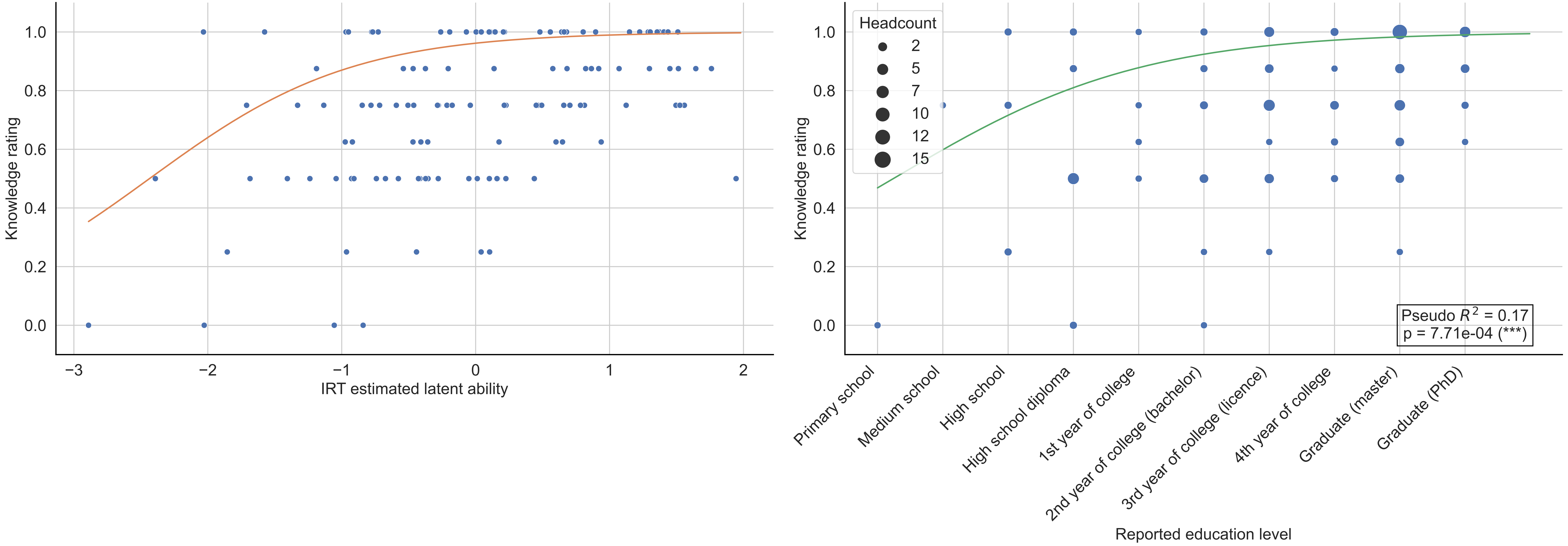

Word: cardinal ("cardinal"; Bachelor) -- n = 157

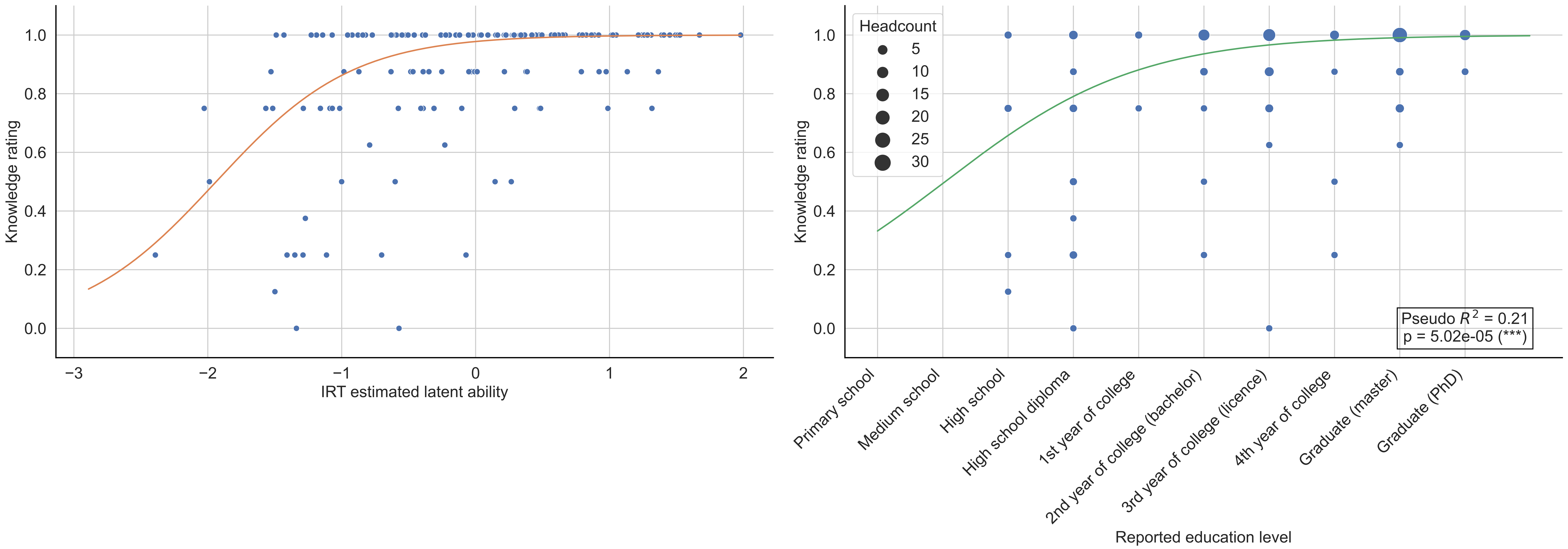

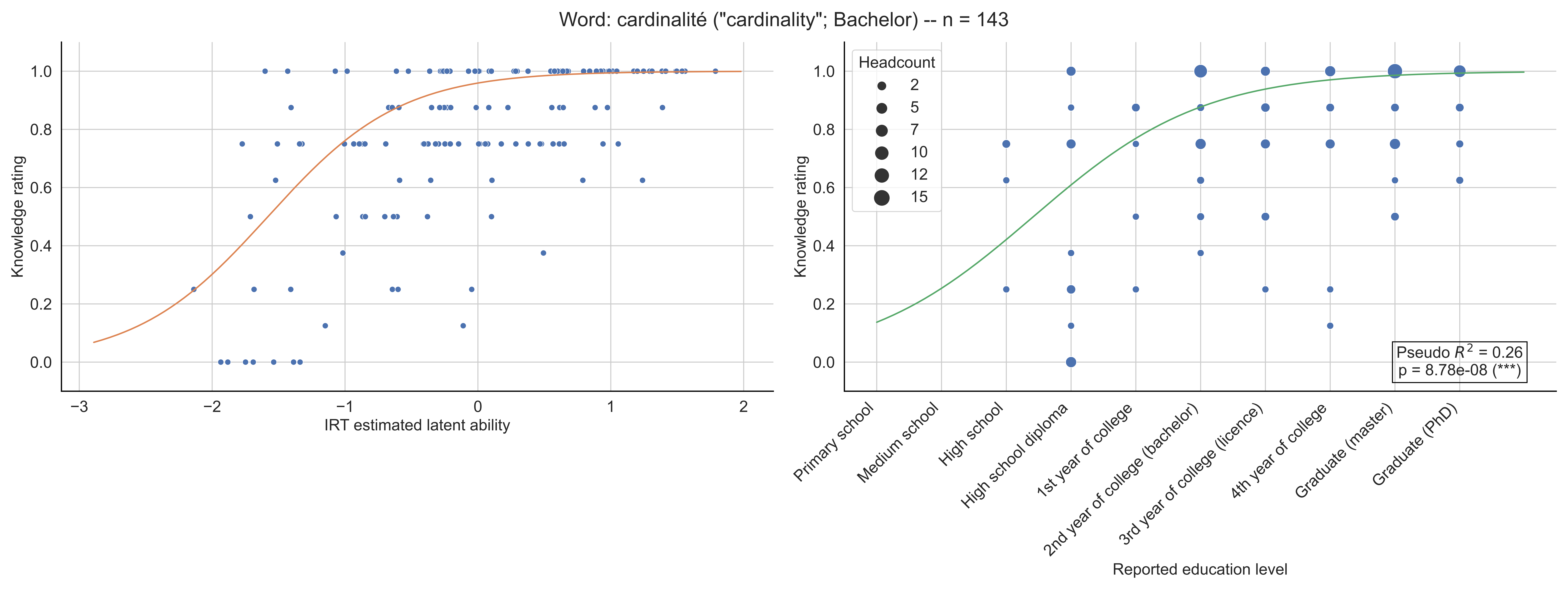

Word: cardinalité ("cardinality"; Bachelor) -- n = 143
Knowledge rating
IRT estimated latent ability
Headcount
2
5
7
10
12
15
Pseudo R^2 = 0.26
p = 8.78e-08 (***)
Primary school
Medium school
High school
High school diploma
1st year of college
2nd year of college (bachelor)
3rd year of college (licence)
4th year of college
Graduate (master)
Graduate (PhD)
Reported education level

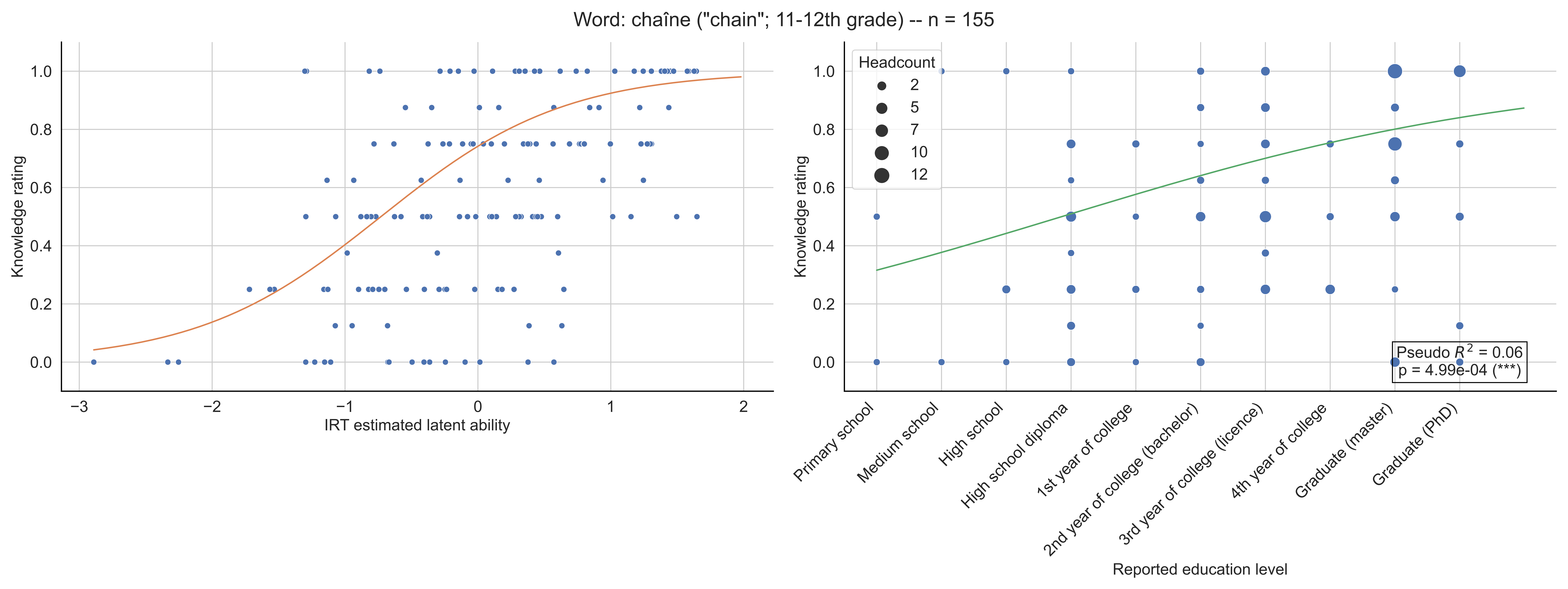

Word: chaîne ("chain"; 11-12th grade) -- n = 155
Knowledge rating
IRT estimated latent ability
Headcount
2
5
7
10
12
Pseudo R^2 = 0.06
p = 4.99e-04 (***)
Primary school
Medium school
High school
High school diploma
1st year of college
2nd year of college (bachelor)
3rd year of college (licence)
4th year of college
Graduate (master)
Graduate (PhD)
Reported education level

Word: codimension ("codimension"; Bachelor) -- n = 145

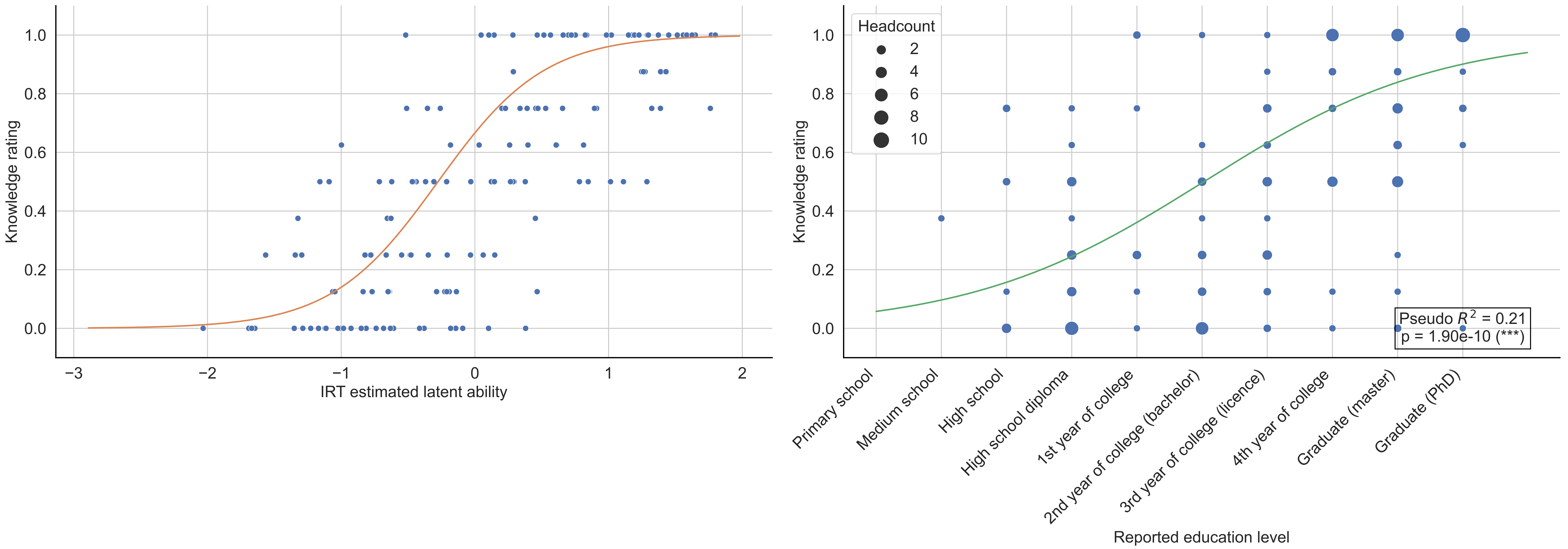

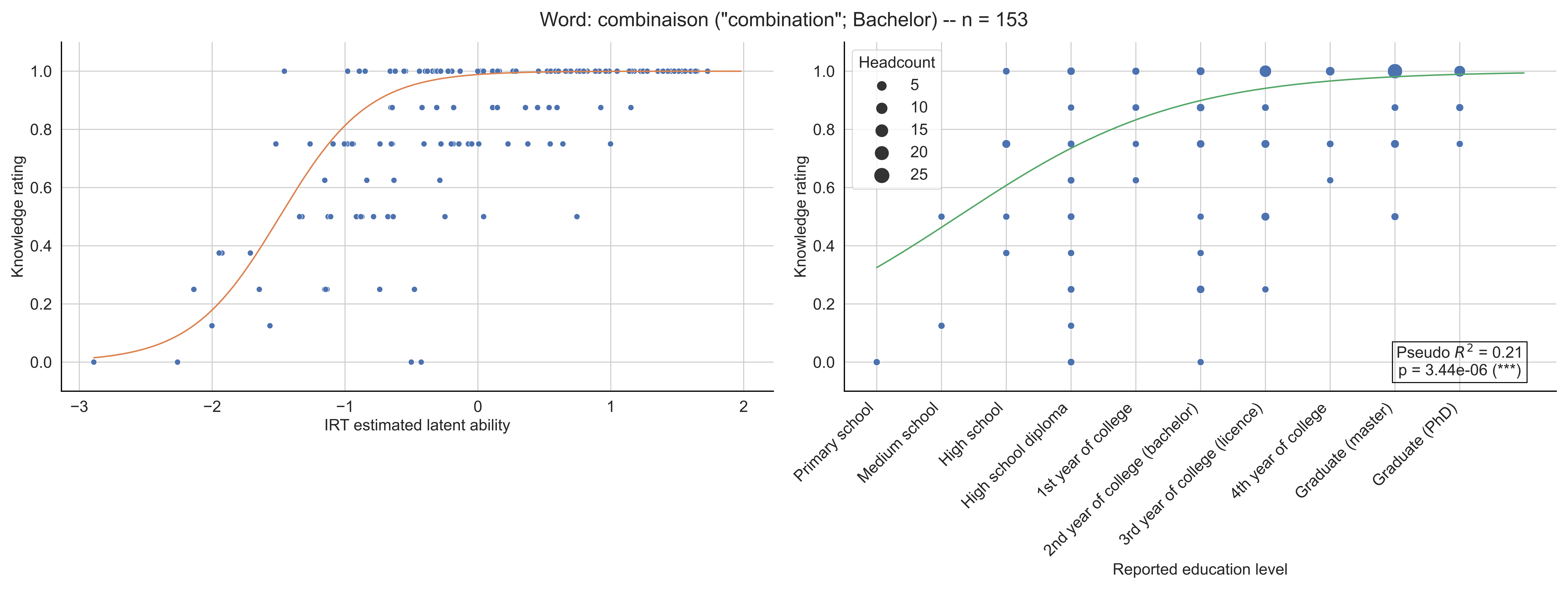
Word: combinaison ("combination"; Bachelor) -- n = 153
Knowledge rating
IRT estimated latent ability
−3
−2
−1
0
1
2
0.0
0.2
0.4
0.6
0.8
1.0
Headcount
5
10
15
20
25
Pseudo R^2 = 0.21
p = 3.44e-06 (***)
Primary school
Medium school
High school
High school diploma
1st year of college
2nd year of college (bachelor)
3rd year of college (licence)
4th year of college
Graduate (master)
Graduate (PhD)
Reported education level

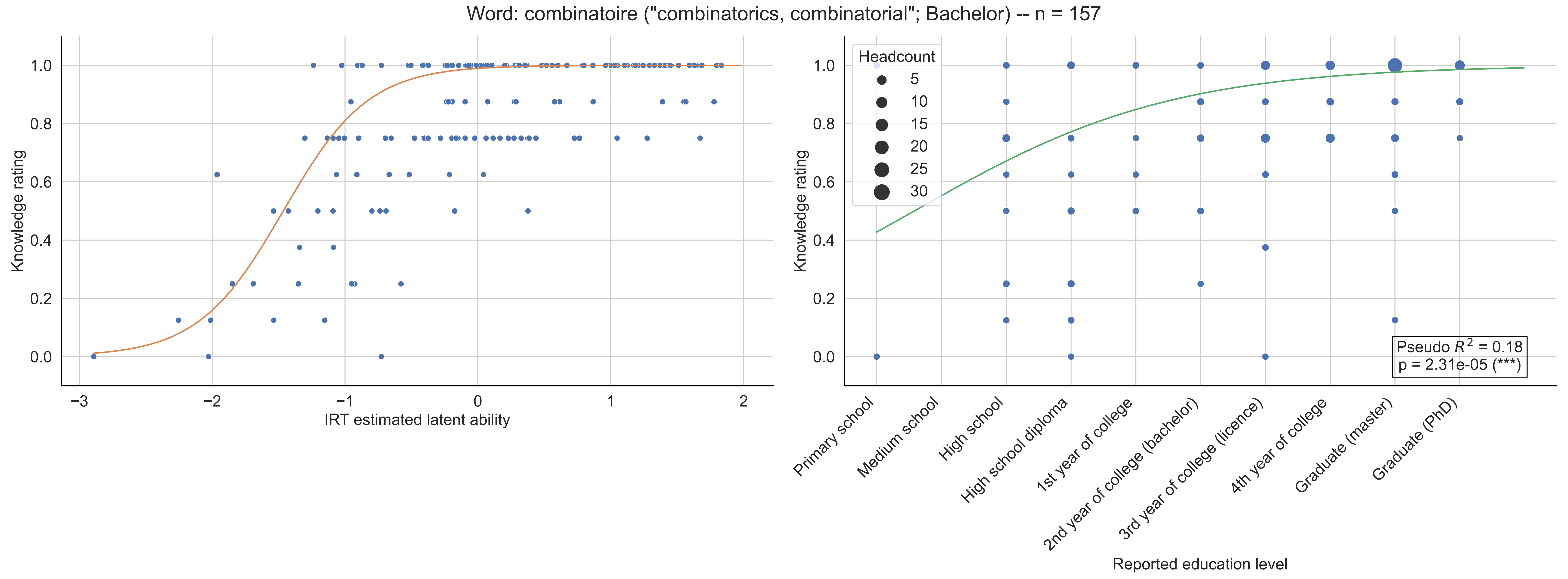
Word: combinatoire ("combinatorics, combinatorial"; Bachelor) -- n = 157
Knowledge rating
IRT estimated latent ability
Headcount
5
10
15
20
25
30
Pseudo R^2 = 0.18
p = 2.31e-05 (***)
Primary school
Medium school
High school
High school diploma
1st year of college
2nd year of college (bachelor)
3rd year of college (licence)
4th year of college
Graduate (master)
Graduate (PhD)
Reported education level

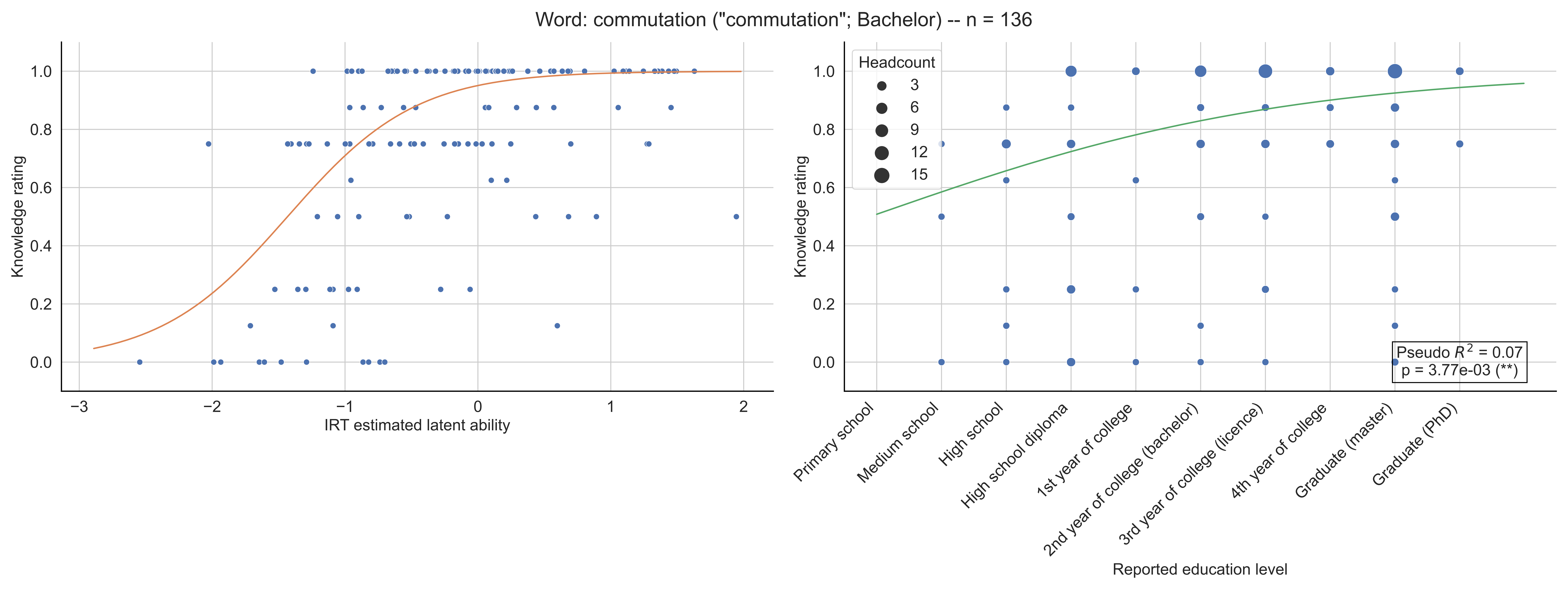

Word: commutation ("commutation"; Bachelor) -- n = 136
Knowledge rating
IRT estimated latent ability
Headcount
3
6
9
12
15
Pseudo R^2 = 0.07
p = 3.77e-03 (**)
Primary school
Medium school
High school
High school diploma
1st year of college
2nd year of college (bachelor)
3rd year of college (licence)
4th year of college
Graduate (master)
Graduate (PhD)
Reported education level

Word: commutativité ("commutativity"; Bachelor) -- n = 146

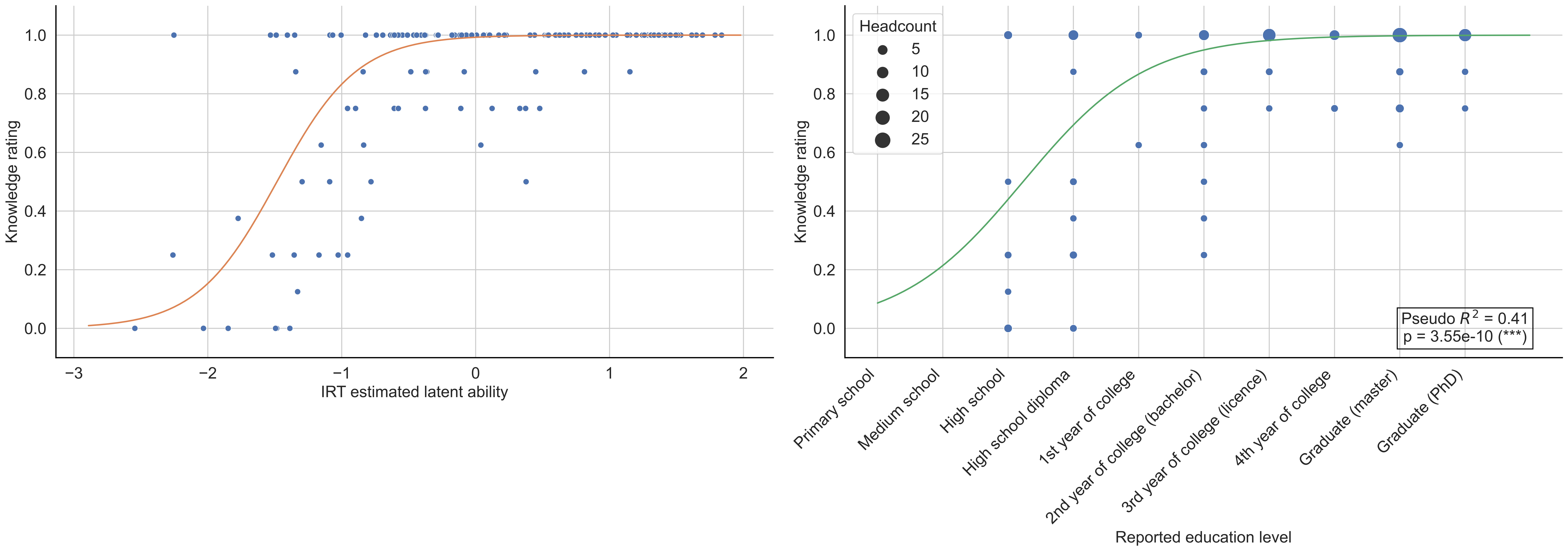

Word: compacité ("compactness"; Bachelor) -- n = 139

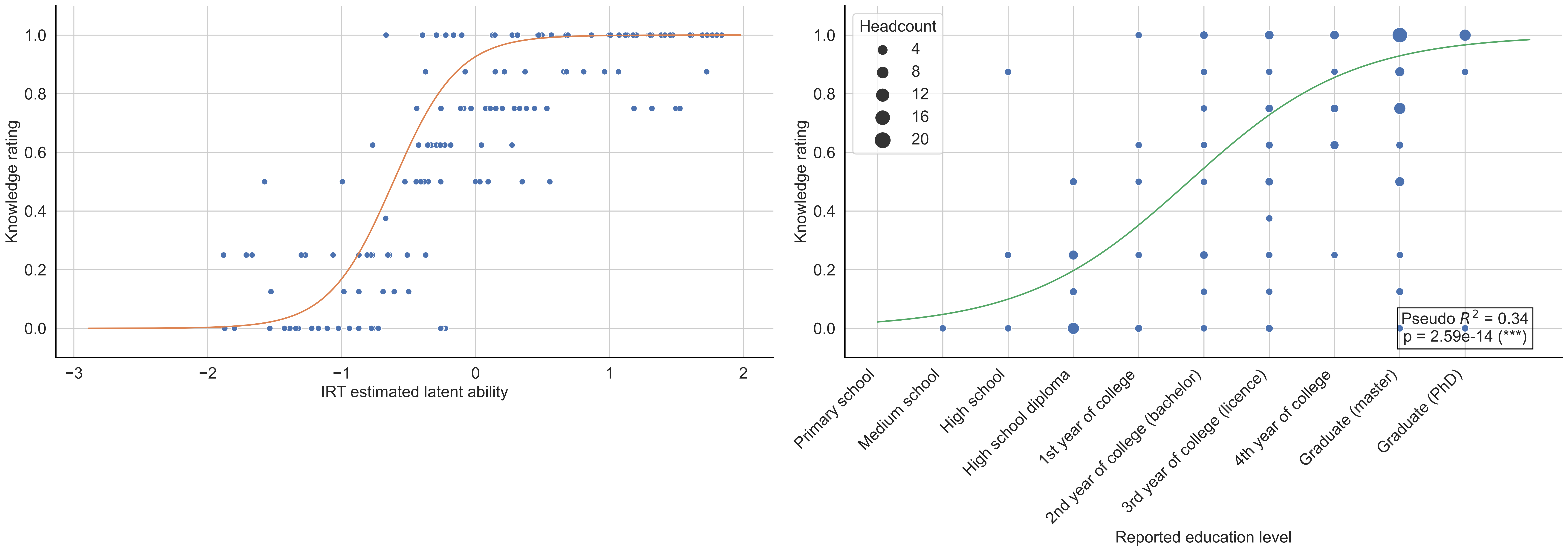

Word: complexe ("complex"; 11-12th grade) -- n = 121

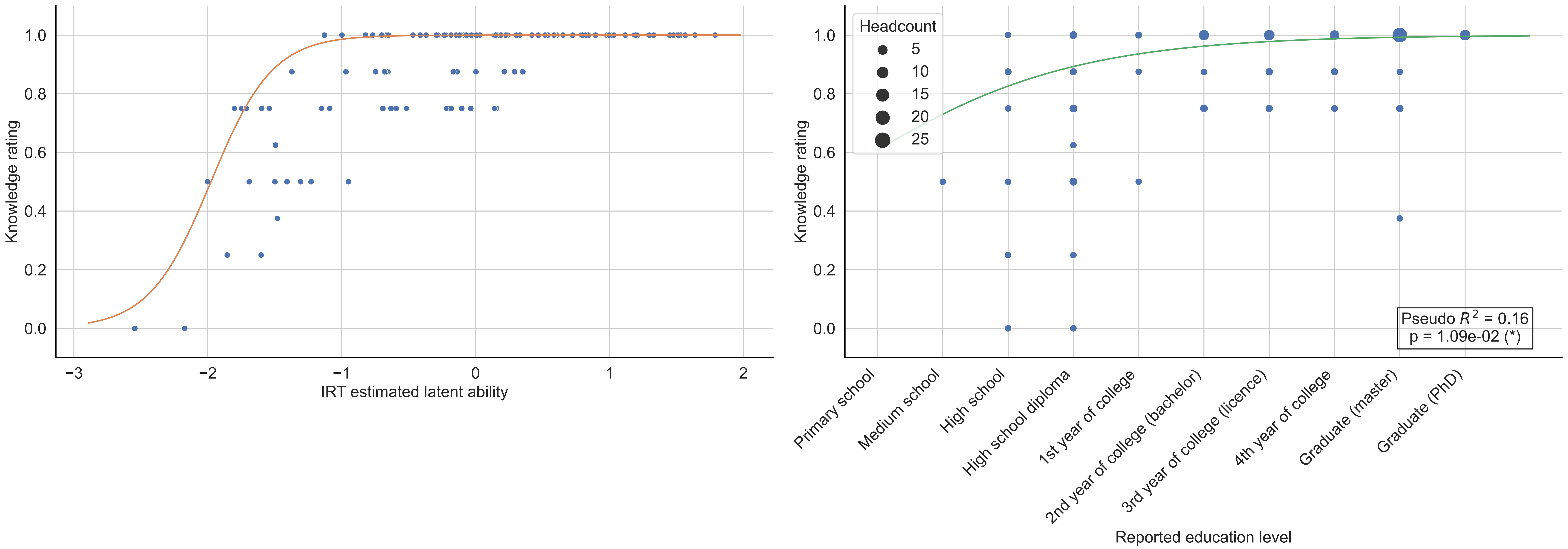

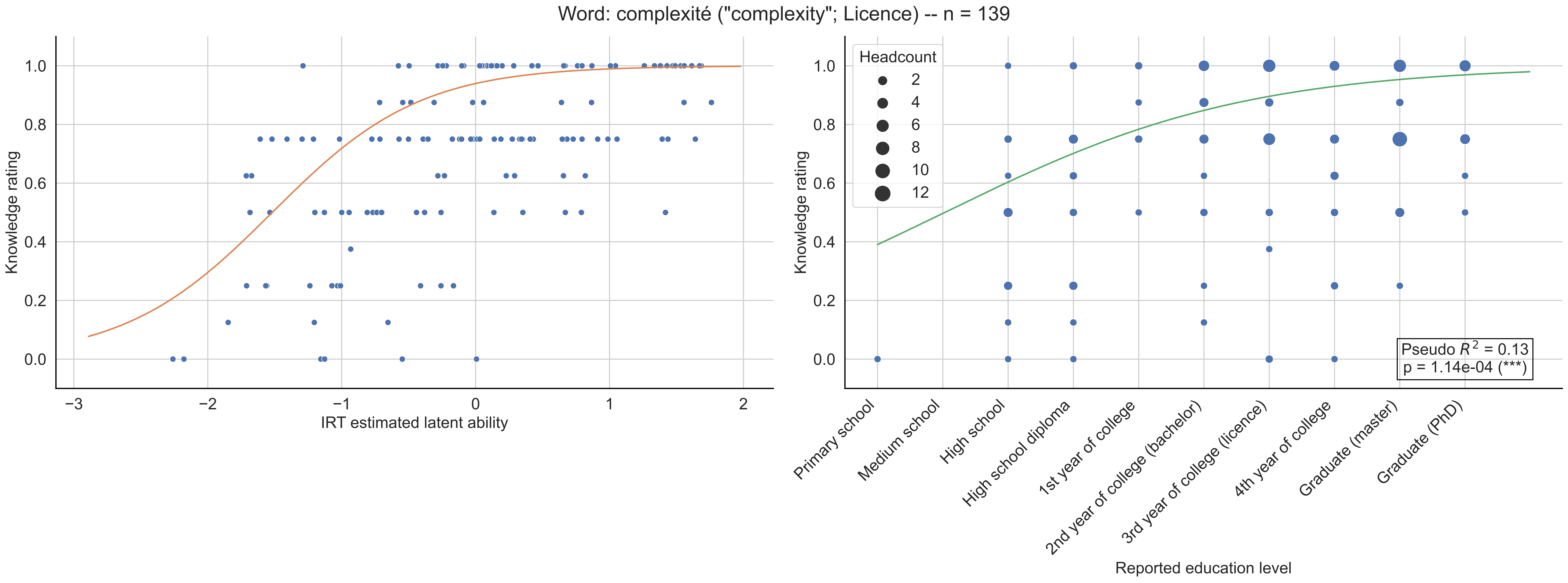

Word: complexité ("complexity"; Licence) -- n = 139
Knowledge rating
IRT estimated latent ability
Headcount
2
4
6
8
10
12
Pseudo R^2 = 0.13
p = 1.14e-04 (***)
Primary school
Medium school
High school
High school diploma
1st year of college
2nd year of college (bachelor)
3rd year of college (licence)
4th year of college
Graduate (master)
Graduate (PhD)
Reported education level

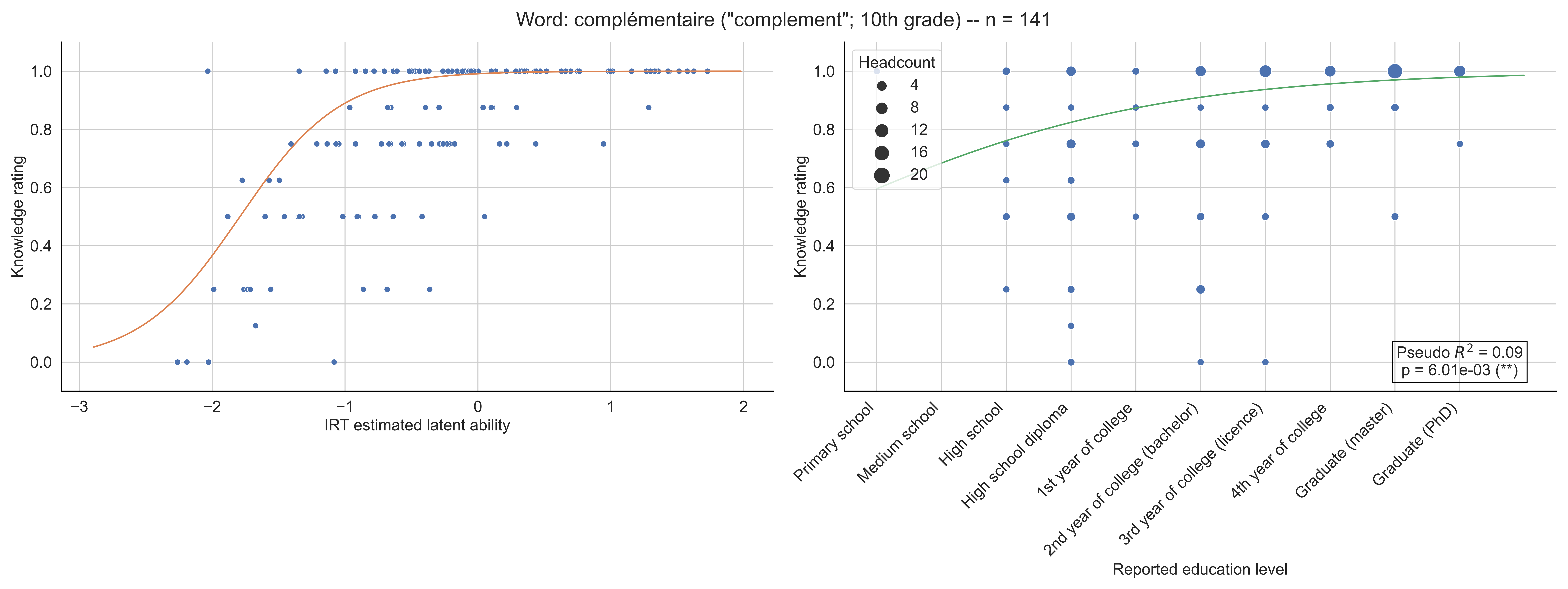

Word: complémentaire ("complement"; 10th grade) -- n = 141
Knowledge rating
IRT estimated latent ability
Headcount
4
8
12
16
20
Pseudo R^2 = 0.09
p = 6.01e-03 (**)
Primary school
Medium school
High school
High school diploma
1st year of college
2nd year of college (bachelor)
3rd year of college (licence)
4th year of college
Graduate (master)
Graduate (PhD)
Reported education level

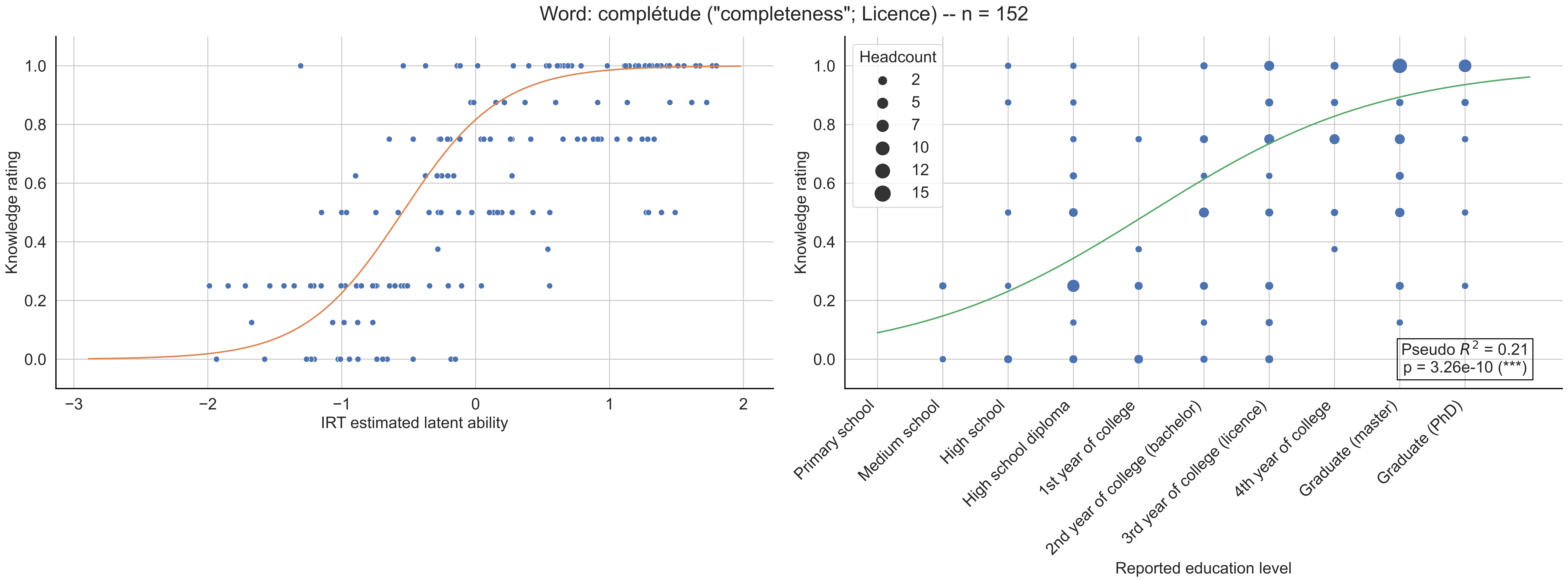
Word: complétude ("completeness"; Licence) -- n = 152
Knowledge rating
IRT estimated latent ability
Headcount
2
5
7
10
12
15
Pseudo R^2 = 0.21
p = 3.26e-10 (***)
Primary school
Medium school
High school
High school diploma
1st year of college
2nd year of college (bachelor)
3rd year of college (licence)
4th year of college
Graduate (master)
Graduate (PhD)
Reported education level

Word: composition ("composition"; 11-12th grade) -- n = 150

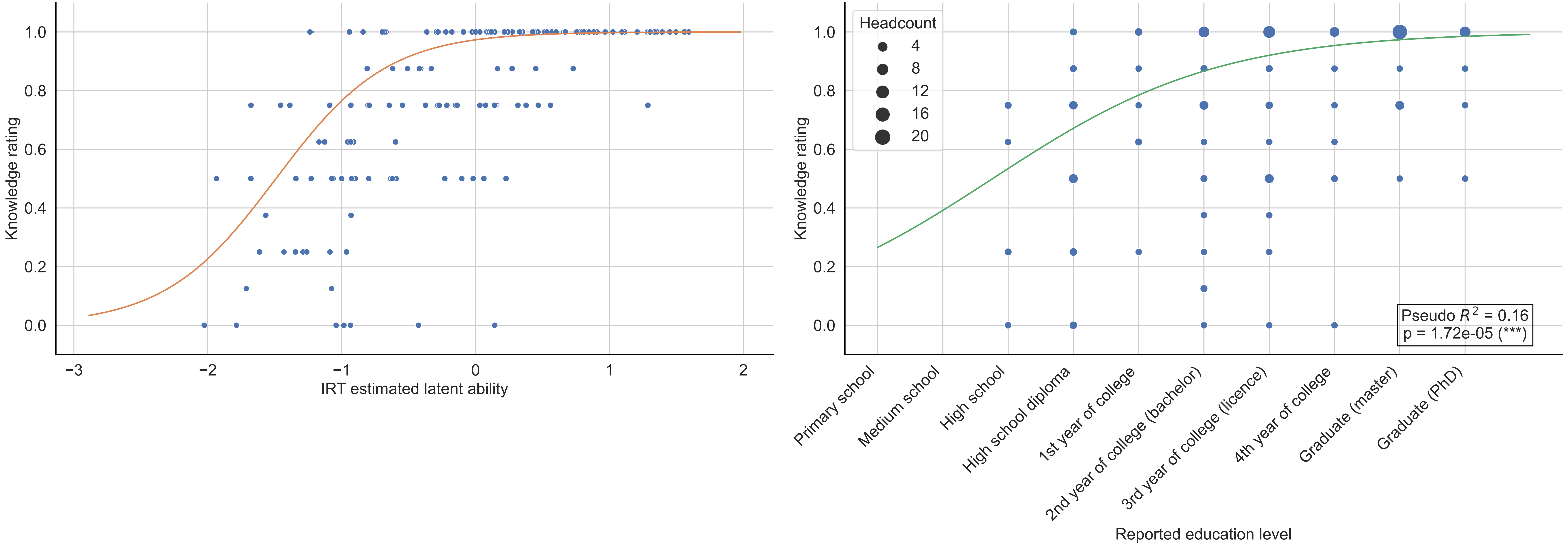

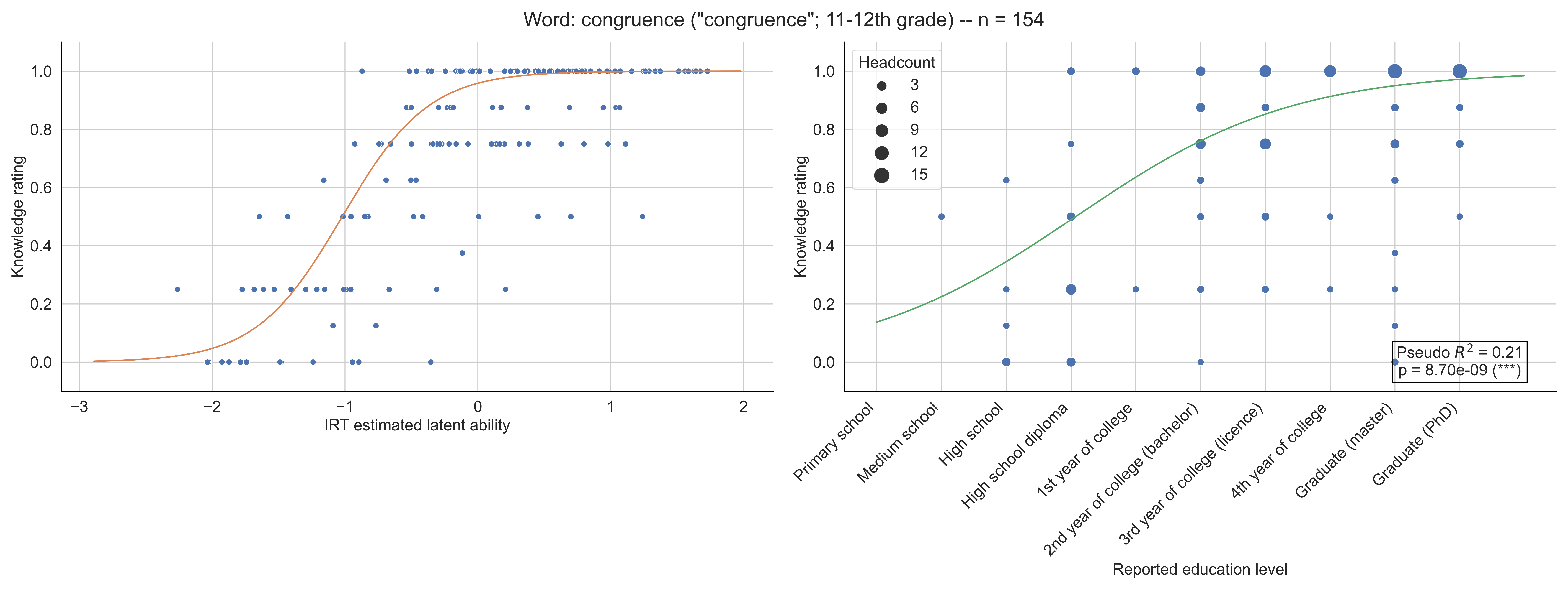

Word: congruence ("congruence"; 11-12th grade) -- n = 154
Knowledge rating
IRT estimated latent ability
Headcount
3
6
9
12
15
Pseudo R^2 = 0.21
p = 8.70e-09 (***)
Reported education level
Primary school
Medium school
High school
High school diploma
1st year of college
2nd year of college (bachelor)
3rd year of college (licence)
4th year of college
Graduate (master)
Graduate (PhD)

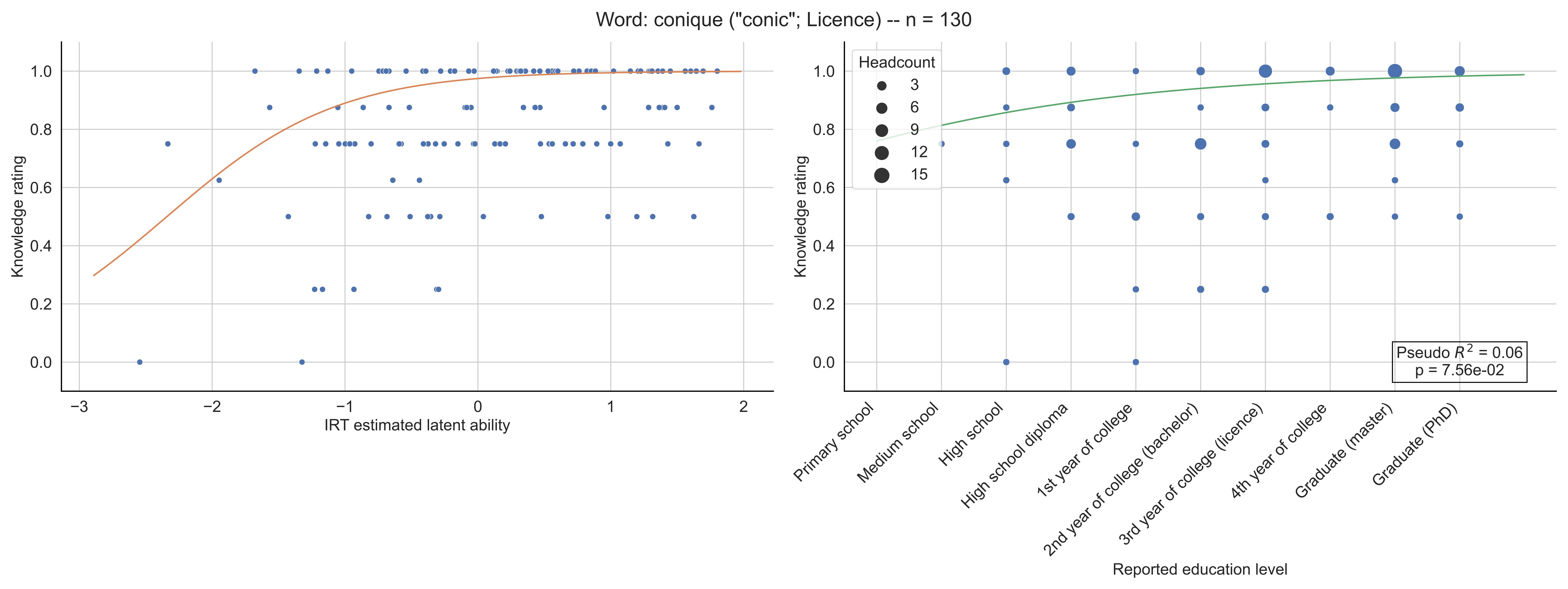

Word: conique ("conic"; Licence) -- n = 130
Knowledge rating
IRT estimated latent ability
Headcount
3
6
9
12
15
Pseudo R^2 = 0.06
p = 7.56e-02
Primary school
Medium school
High school
High school diploma
1st year of college
2nd year of college (bachelor)
3rd year of college (licence)
4th year of college
Graduate (master)
Graduate (PhD)
Reported education level

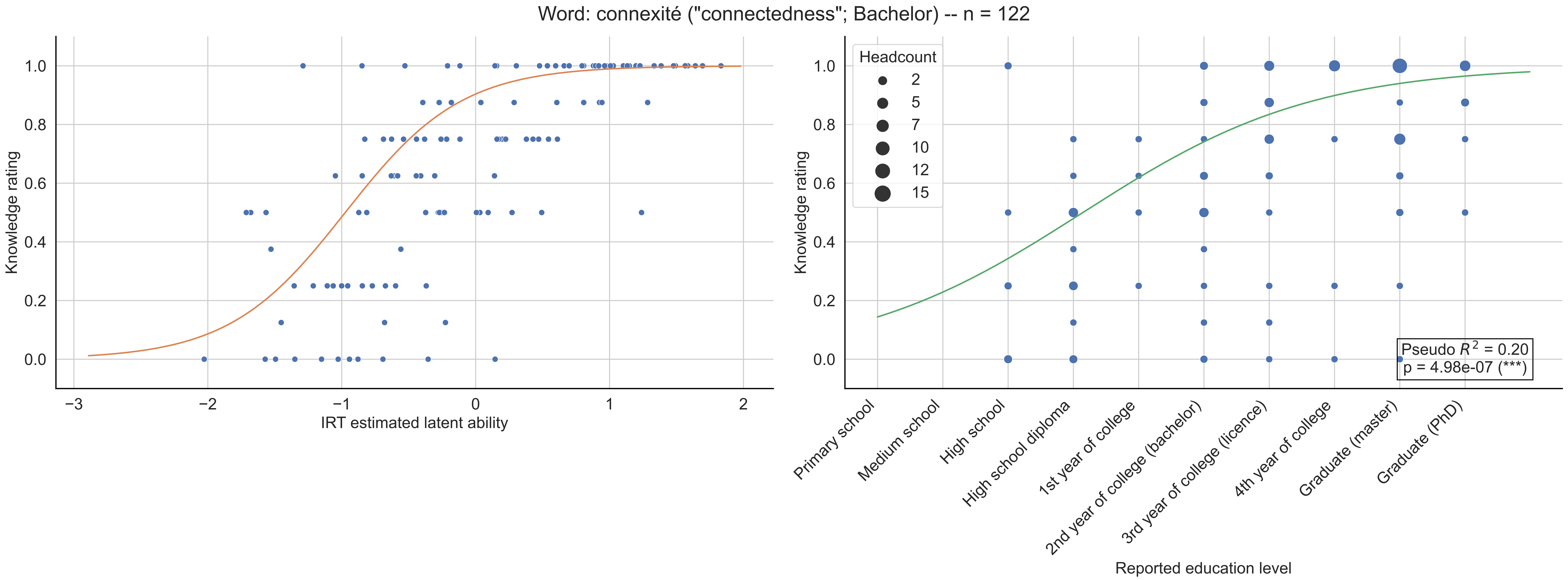

Word: connexité ("connectedness"; Bachelor) -- n = 122
Knowledge rating
IRT estimated latent ability
Headcount
2
5
7
10
12
15
Pseudo R^2 = 0.20
p = 4.98e-07 (***)
Primary school
Medium school
High school
High school diploma
1st year of college
2nd year of college (bachelor)
3rd year of college (licence)
4th year of college
Graduate (master)
Graduate (PhD)
Reported education level

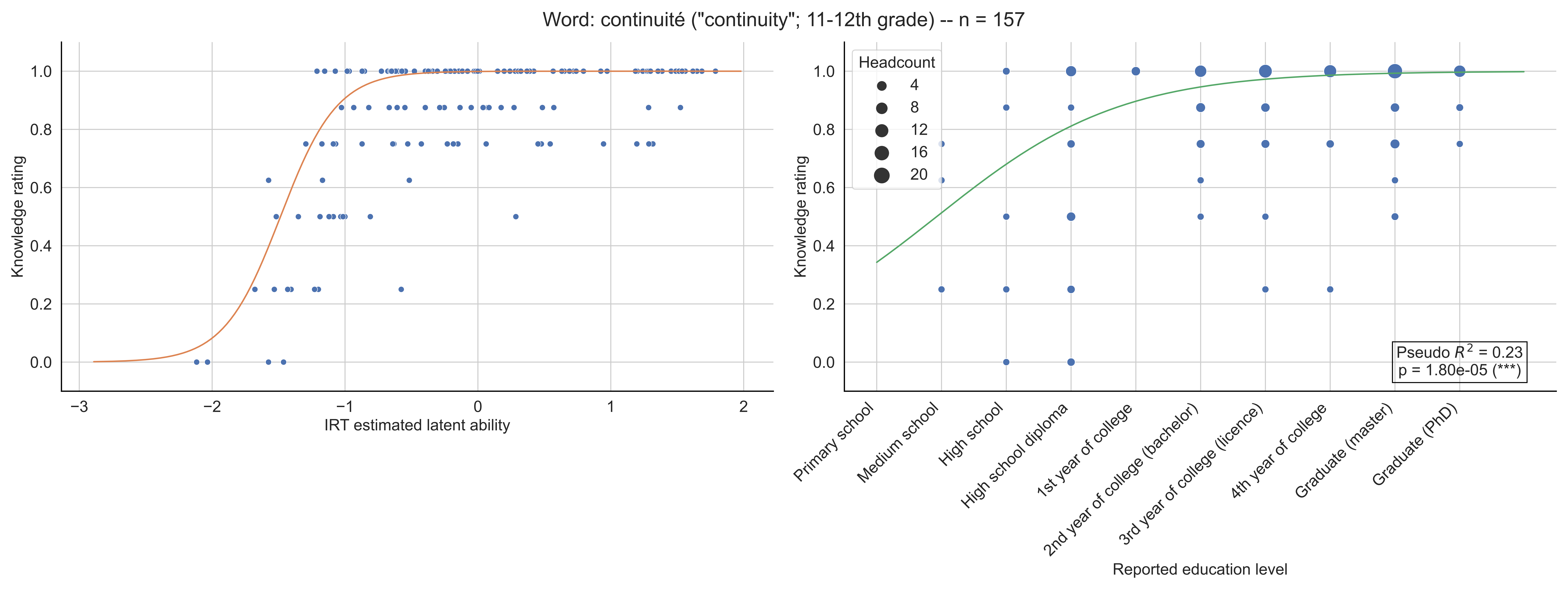

Word: continuité ("continuity"; 11-12th grade) -- n = 157
Knowledge rating
IRT estimated latent ability
Headcount
4
8
12
16
20
Pseudo R^2 = 0.23
p = 1.80e-05 (***)
Primary school
Medium school
High school
High school diploma
1st year of college
2nd year of college (bachelor)
3rd year of college (licence)
4th year of college
Graduate (master)
Graduate (PhD)
Reported education level

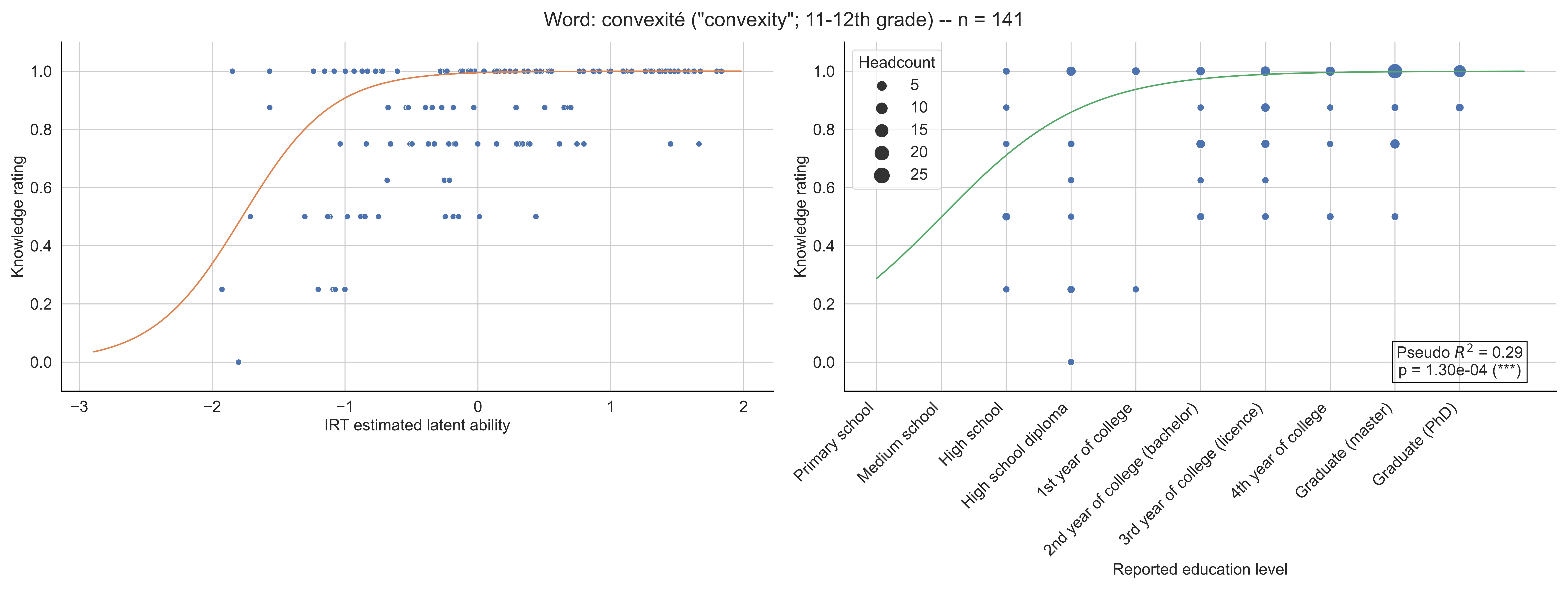
Word: convexité ("convexity"; 11-12th grade) -- n = 141
Knowledge rating
IRT estimated latent ability
Headcount
5
10
15
20
25
Pseudo R^2 = 0.29
p = 1.30e-04 (***)
Primary school
Medium school
High school
High school diploma
1st year of college
2nd year of college (bachelor)
3rd year of college (licence)
4th year of college
Graduate (master)
Graduate (PhD)
Reported education level

Word: convolution ("convolution"; Licence) -- n = 152

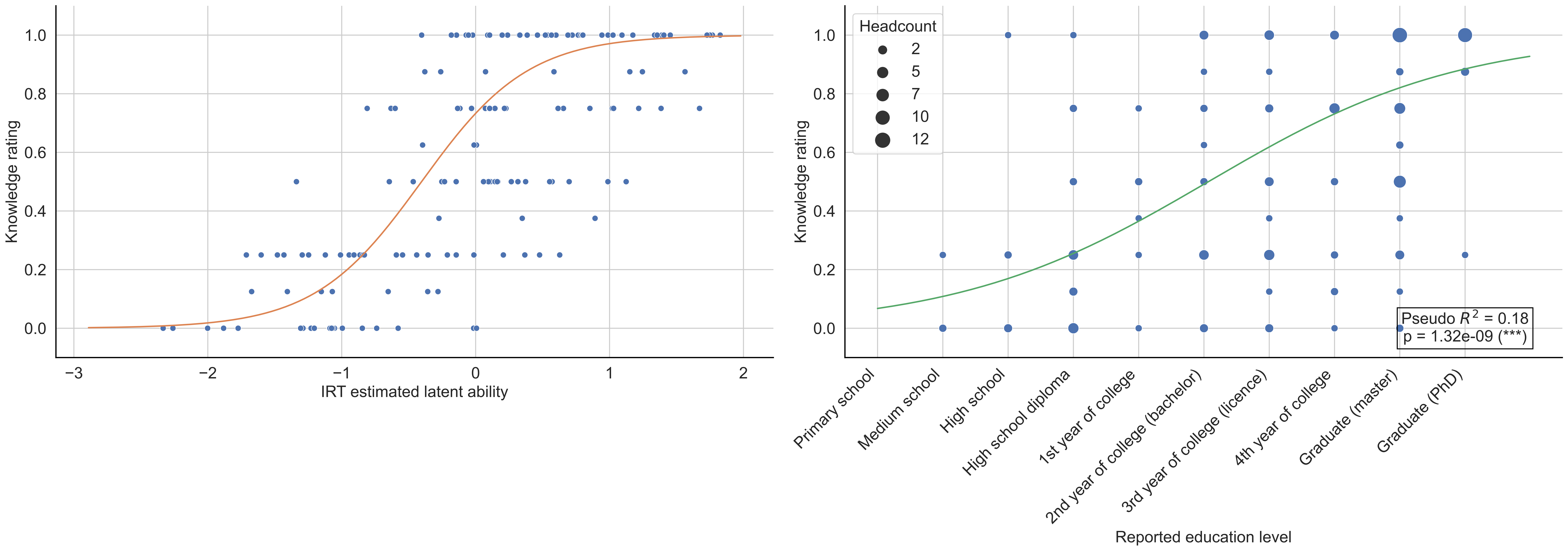

Word: corde ("chord"; 6-7th grade) -- n = 145

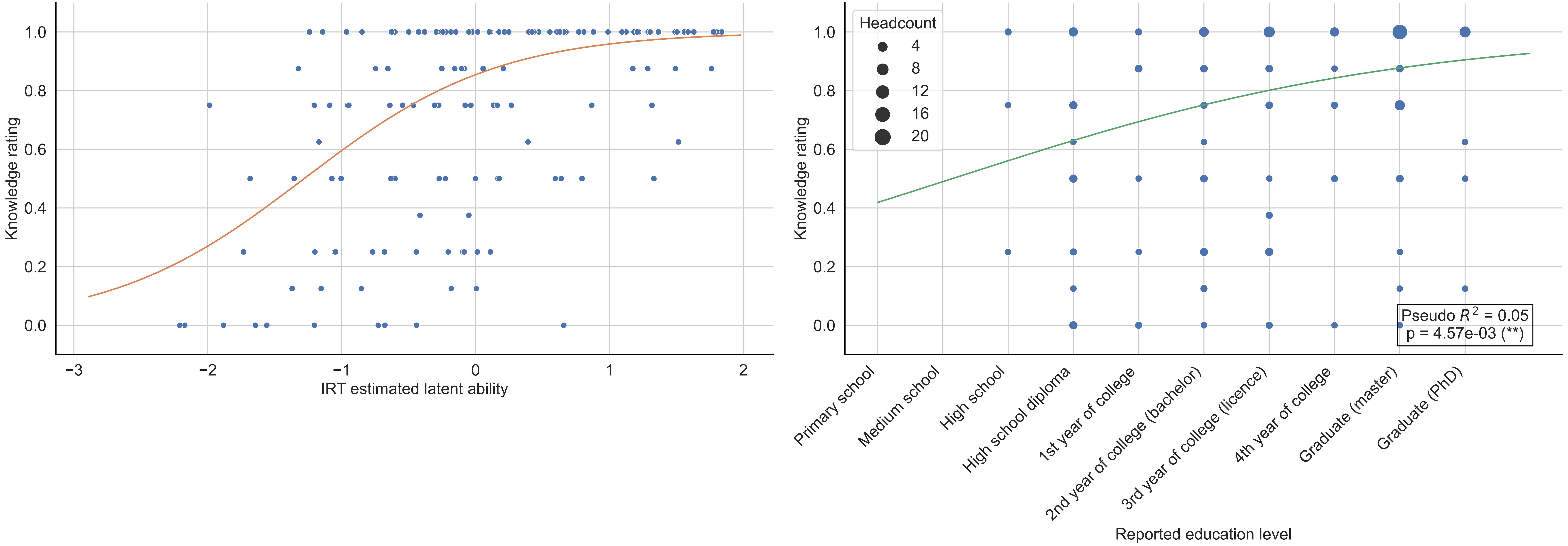

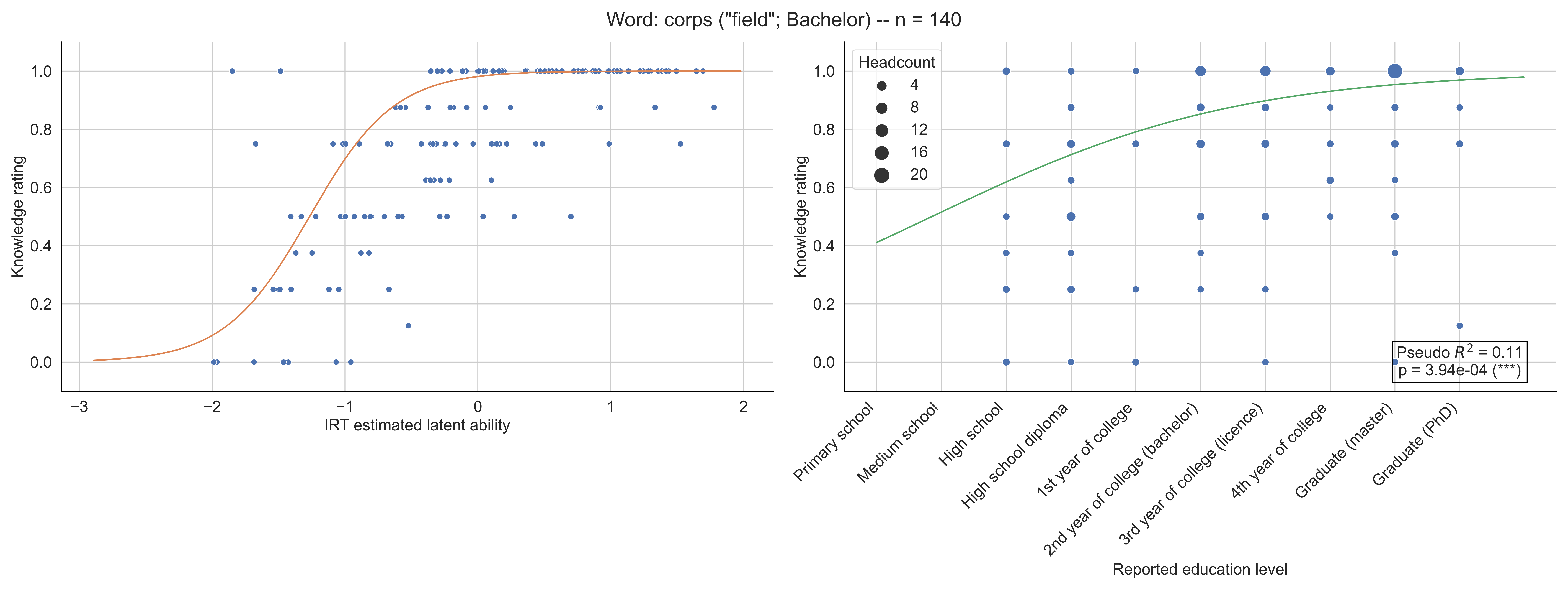
Word: corps ("field"; Bachelor) -- n = 140
Knowledge rating
IRT estimated latent ability
Headcount
4
8
12
16
20
Pseudo R^2 = 0.11
p = 3.94e-04 (***)
Primary school
Medium school
High school
High school diploma
1st year of college
2nd year of college (bachelor)
3rd year of college (licence)
4th year of college
Graduate (master)
Graduate (PhD)
Reported education level

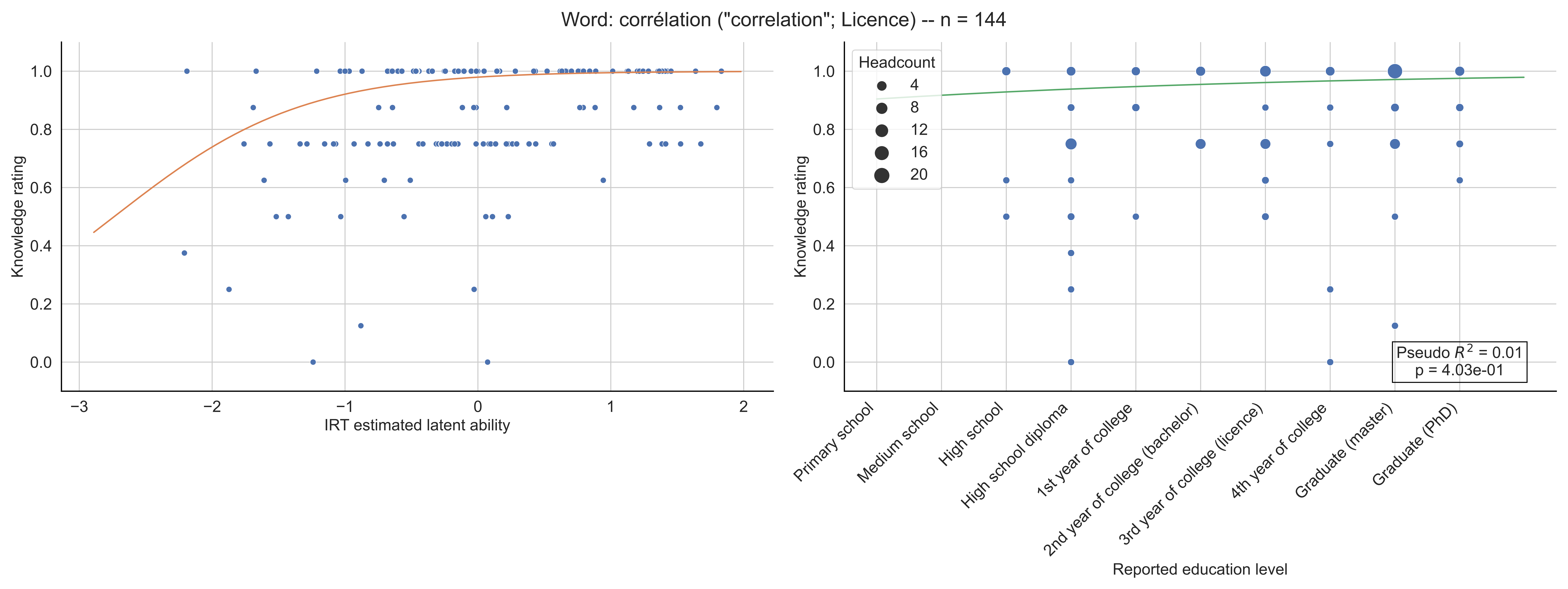

Word: corrélation ("correlation"; Licence) -- n = 144
Knowledge rating
IRT estimated latent ability
Headcount
4
8
12
16
20
Pseudo R^2 = 0.01
p = 4.03e-01
Primary school
Medium school
High school
High school diploma
1st year of college
2nd year of college (bachelor)
3rd year of college (licence)
4th year of college
Graduate (master)
Graduate (PhD)
Reported education level

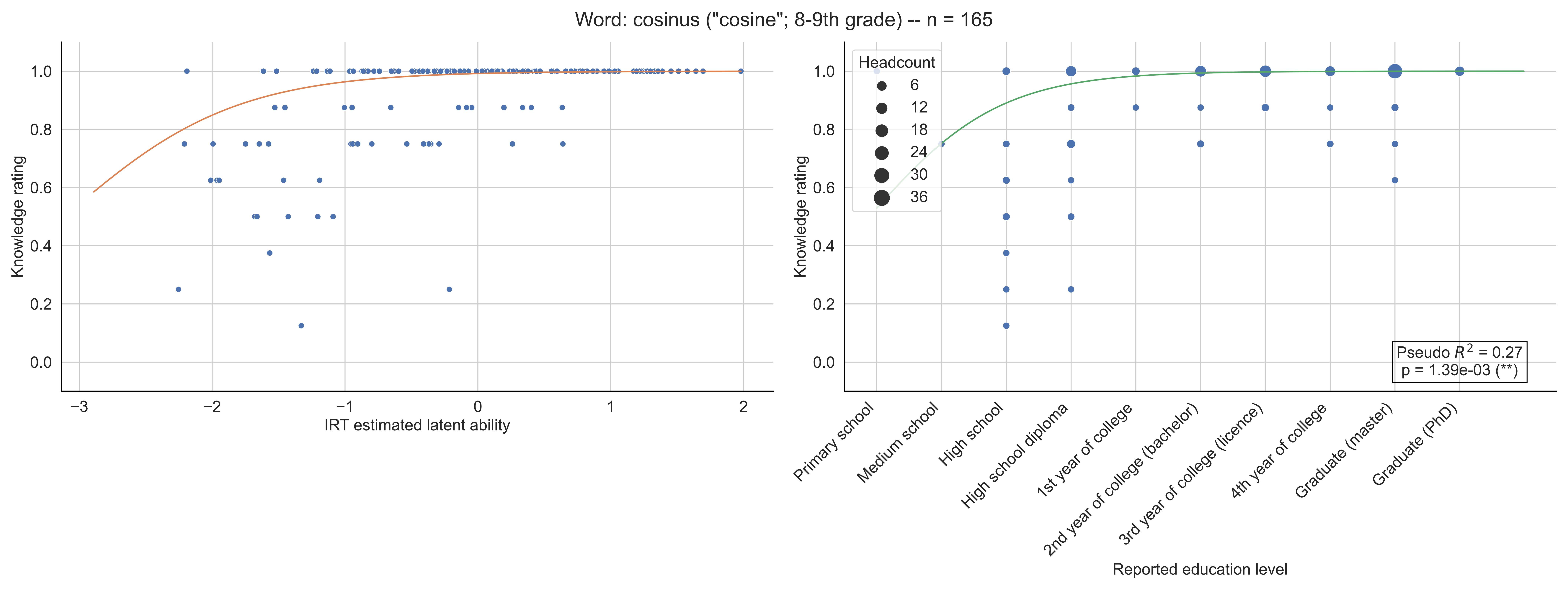

Word: cosinus ("cosine"; 8-9th grade) -- n = 165
Knowledge rating
IRT estimated latent ability
Headcount
6
12
18
24
30
36
Pseudo R^2 = 0.27
p = 1.39e-03 (**)
Primary school
Medium school
High school
High school diploma
1st year of college
2nd year of college (bachelor)
3rd year of college (licence)
4th year of college
Graduate (master)
Graduate (PhD)
Reported education level

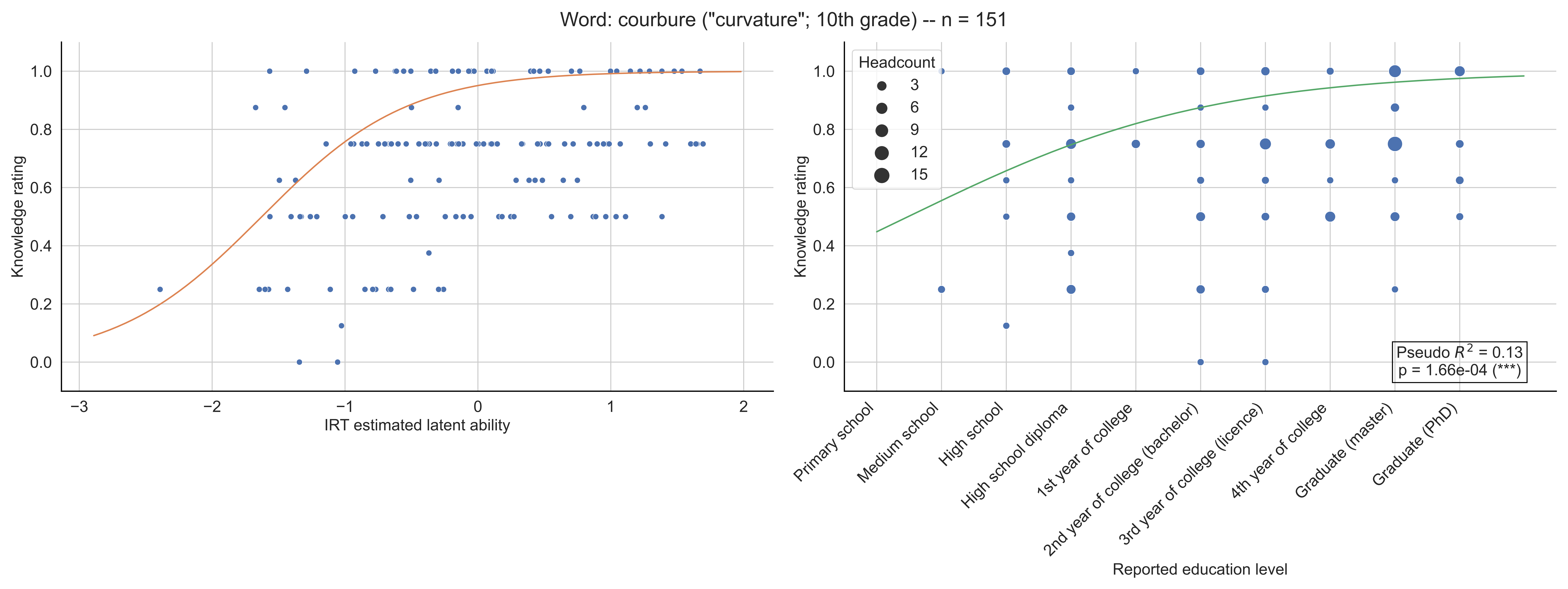
Word: courbure ("curvature"; 10th grade) -- n = 151
Knowledge rating
IRT estimated latent ability
Headcount
3
6
9
12
15
Pseudo R^2 = 0.13
p = 1.66e-04 (***)
Primary school
Medium school
High school
High school diploma
1st year of college
2nd year of college (bachelor)
3rd year of college (licence)
4th year of college
Graduate (master)
Graduate (PhD)
Reported education level

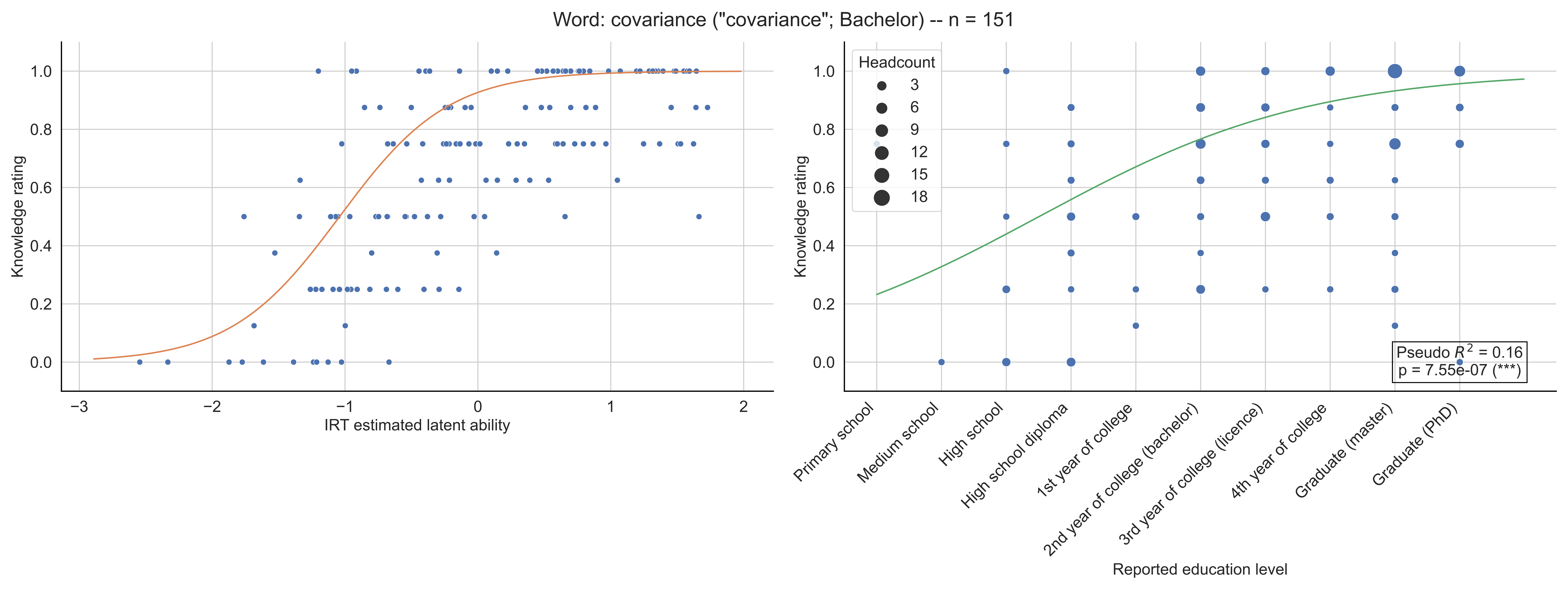

Word: covariance ("covariance"; Bachelor) -- n = 151
Knowledge rating
IRT estimated latent ability
Headcount
3
6
9
12
15
18
Pseudo R^2 = 0.16
p = 7.55e-07 (***)
Primary school
Medium school
High school
High school diploma
1st year of college
2nd year of college (bachelor)
3rd year of college (licence)
4th year of college
Graduate (master)
Graduate (PhD)
Reported education level

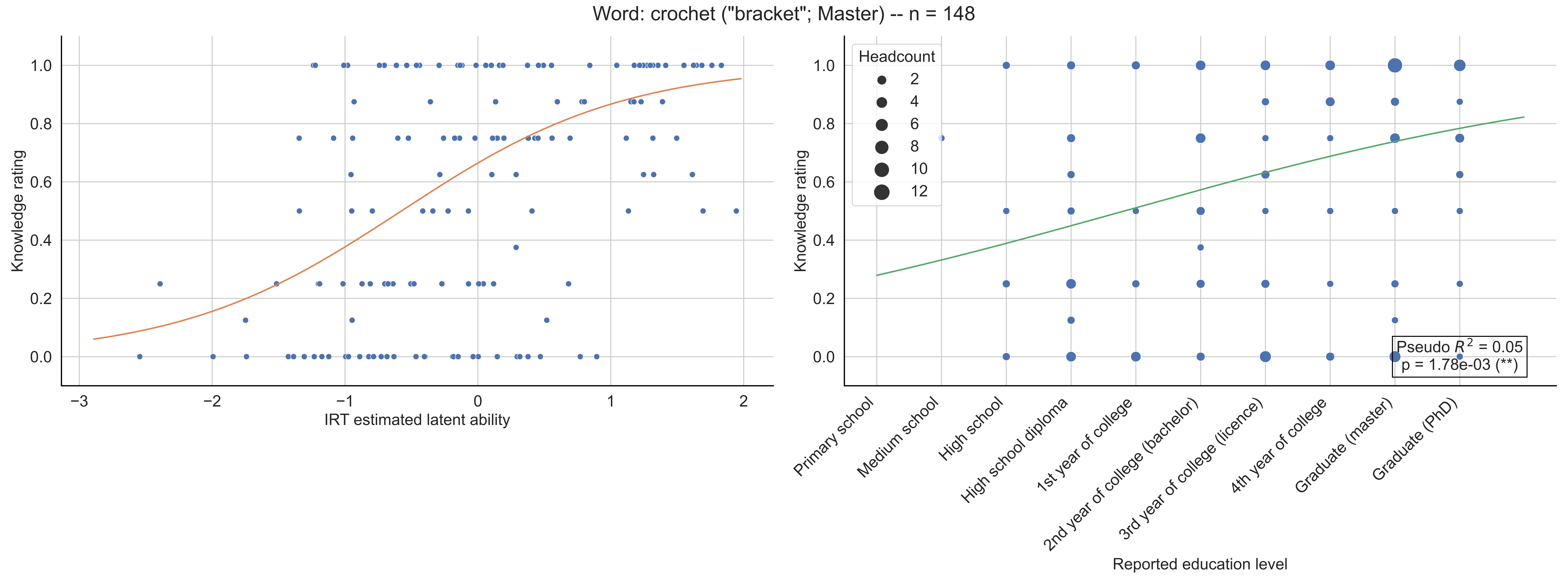

Word: crochet ("bracket"; Master) -- n = 148
Knowledge rating
IRT estimated latent ability
Headcount
2
4
6
8
10
12
Pseudo R^2 = 0.05
p = 1.78e-03 (**)
Primary school
Medium school
High school
High school diploma
1st year of college
2nd year of college (bachelor)
3rd year of college (licence)
4th year of college
Graduate (master)
Graduate (PhD)
Reported education level

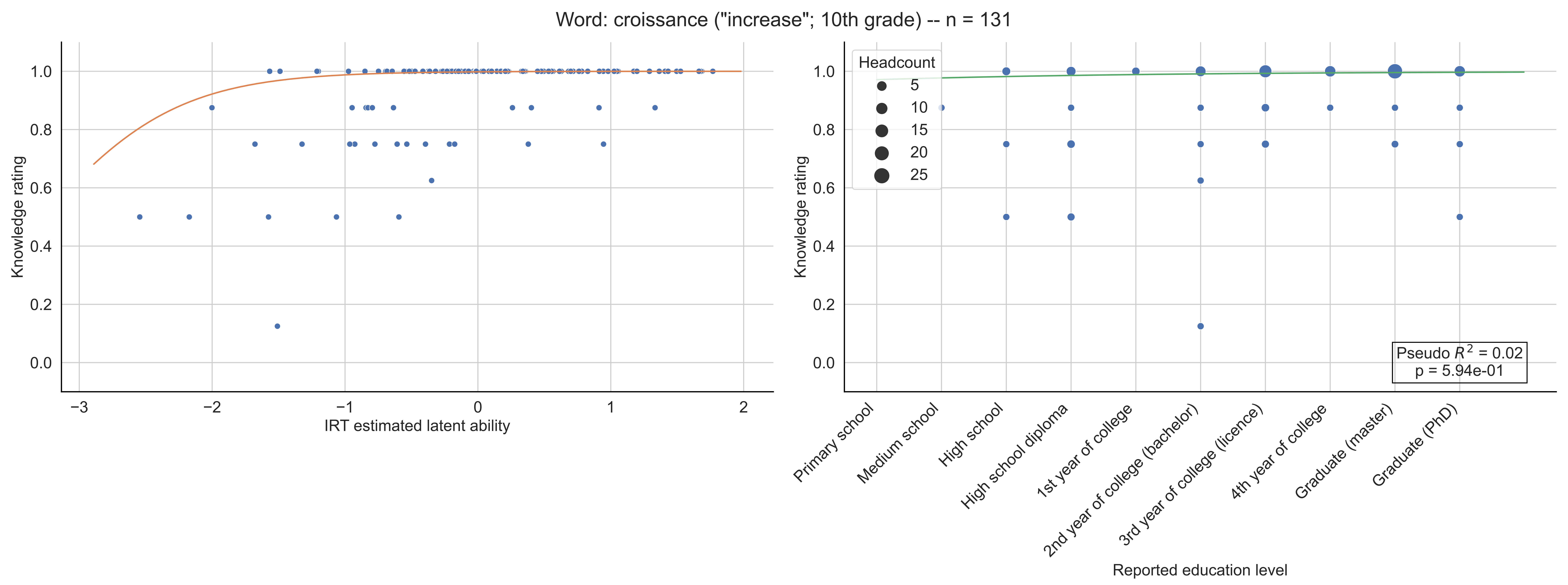

Word: croissance ("increase"; 10th grade) -- n = 131
Knowledge rating
IRT estimated latent ability
−3
−2
−1
0
1
2
0.0
0.2
0.4
0.6
0.8
1.0
Headcount
5
10
15
20
25
Pseudo R^2 = 0.02
p = 5.94e-01
Primary school
Medium school
High school
High school diploma
1st year of college
2nd year of college (bachelor)
3rd year of college (licence)
4th year of college
Graduate (master)
Graduate (PhD)
Reported education level

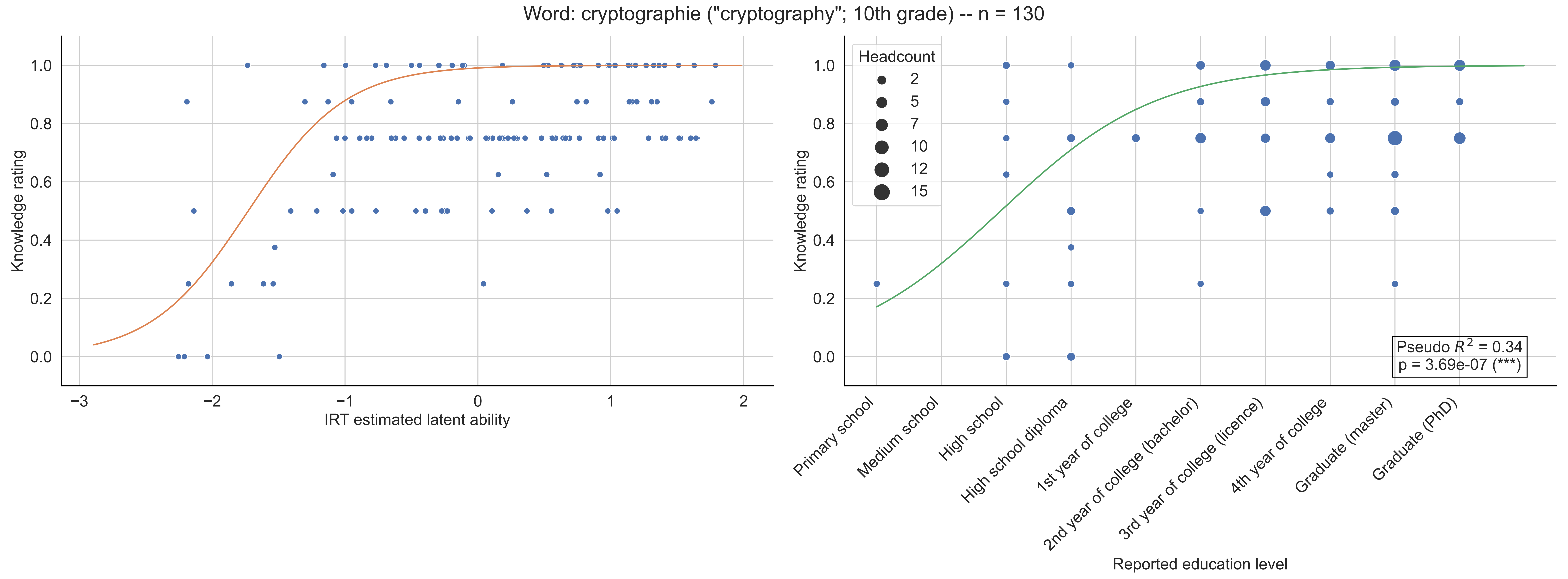

Word: cryptographie ("cryptography"; 10th grade) -- n = 130
Knowledge rating
IRT estimated latent ability
Headcount
2
5
7
10
12
15
Pseudo R^2 = 0.34
p = 3.69e-07 (***)
Primary school
Medium school
High school
High school diploma
1st year of college
2nd year of college (bachelor)
3rd year of college (licence)
4th year of college
Graduate (master)
Graduate (PhD)
Reported education level

Word: cubique ("cubic"; Primary school) -- n = 167

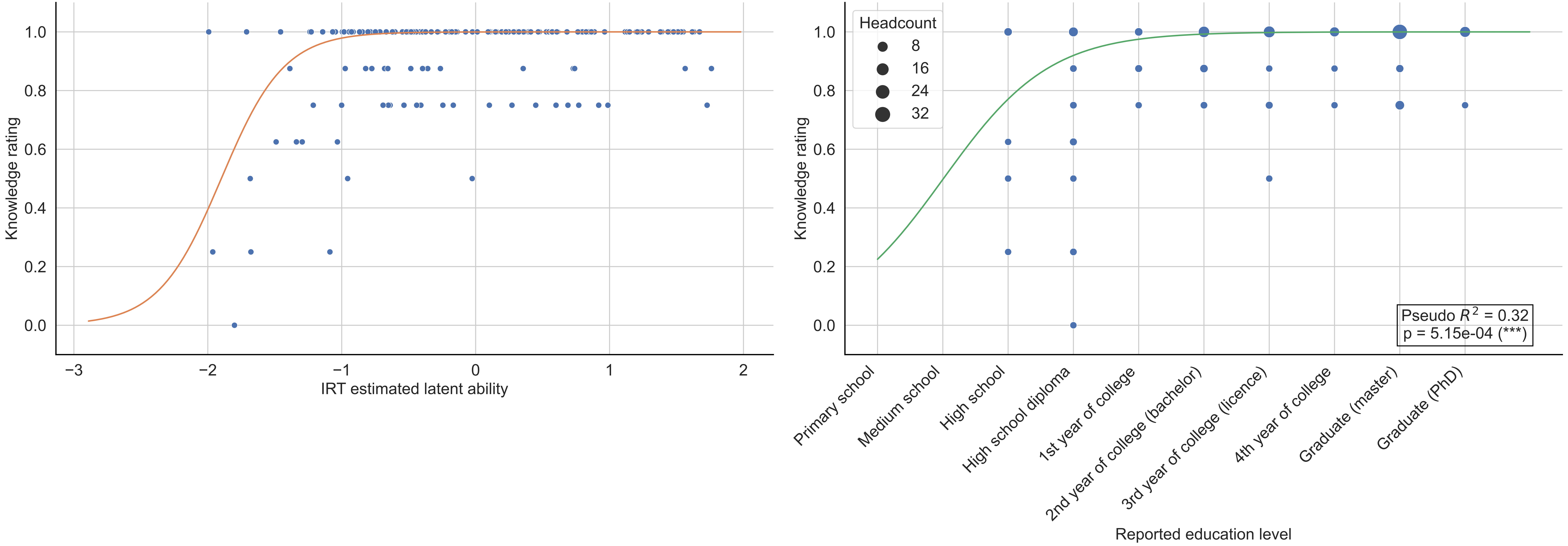

Word: cycle ("cycle"; 11-12th grade) -- n = 147

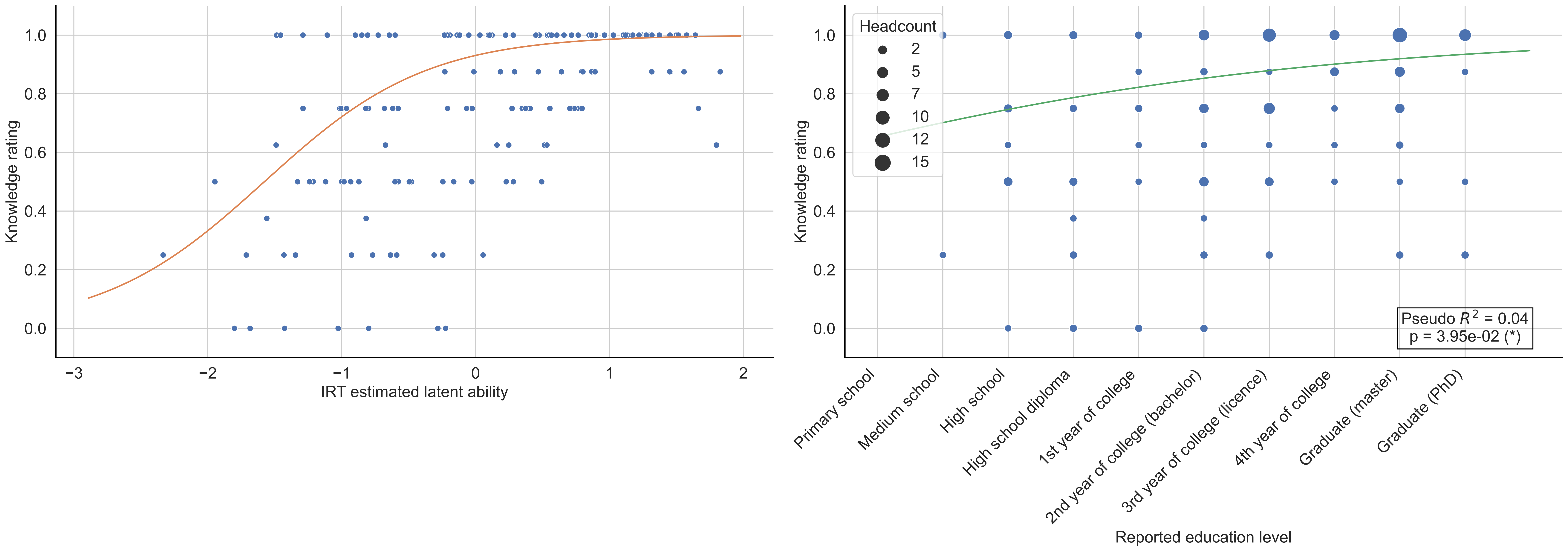

Word: cycloïde ("cycloid"; 11-12th grade) -- n = 144

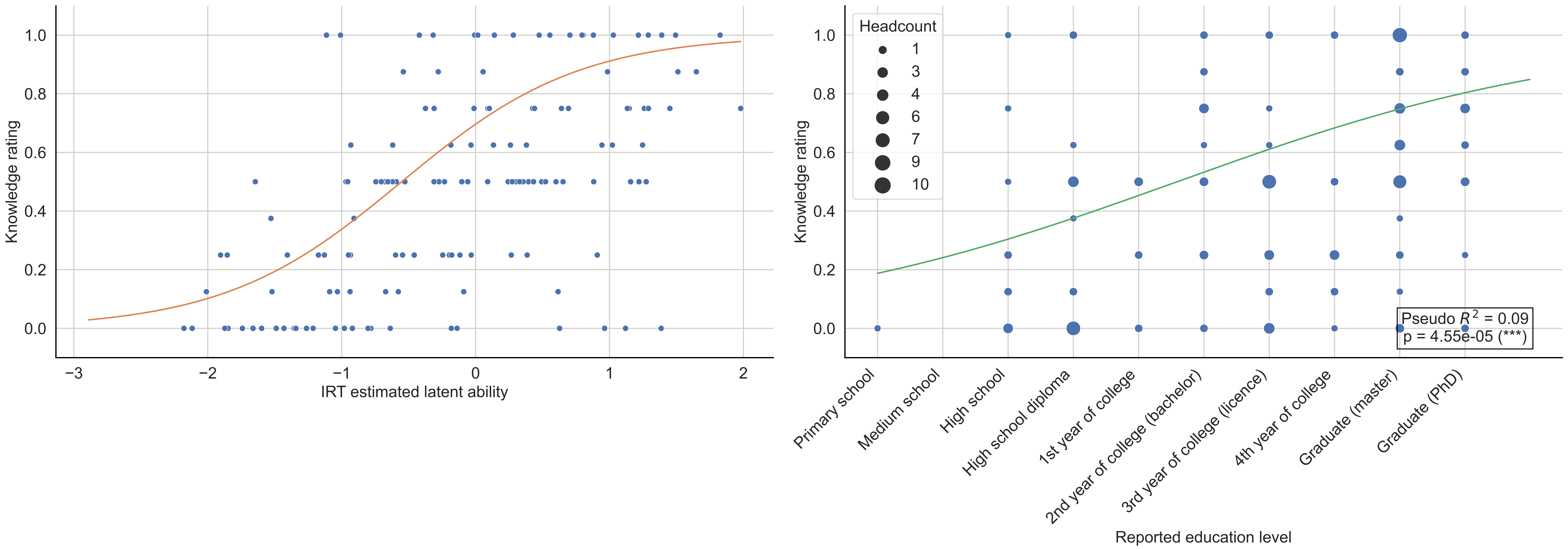

Word: cône ("cone"; Primary school) -- n = 161

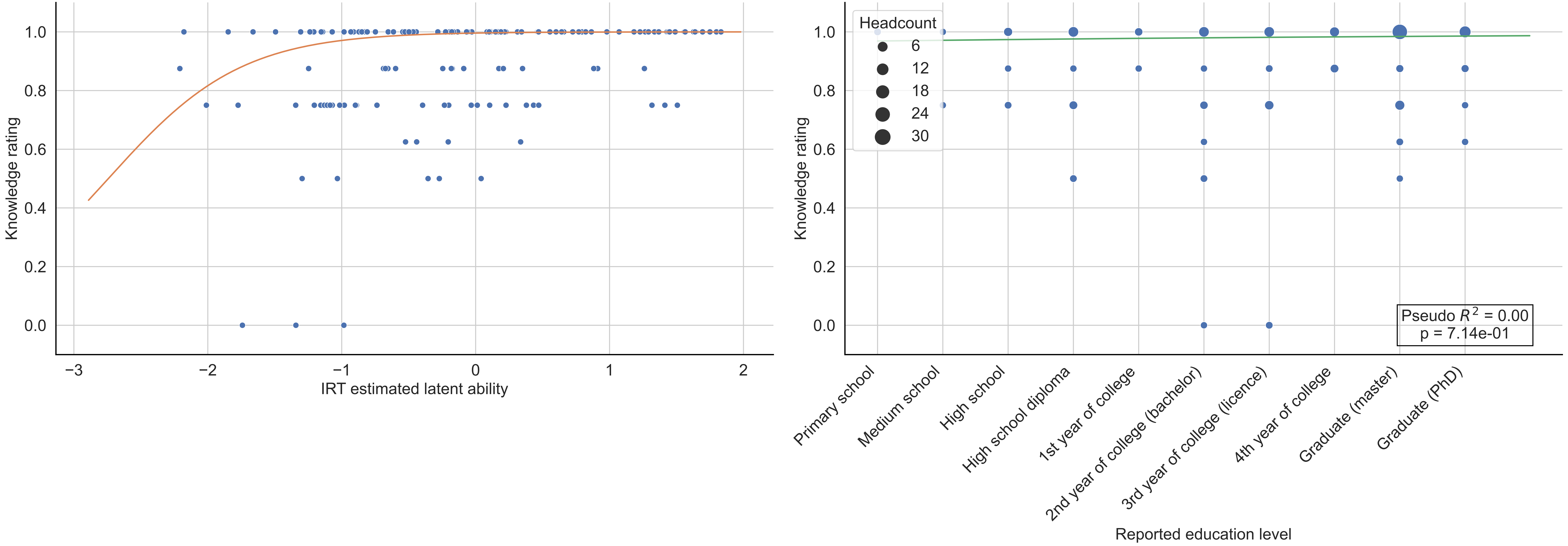

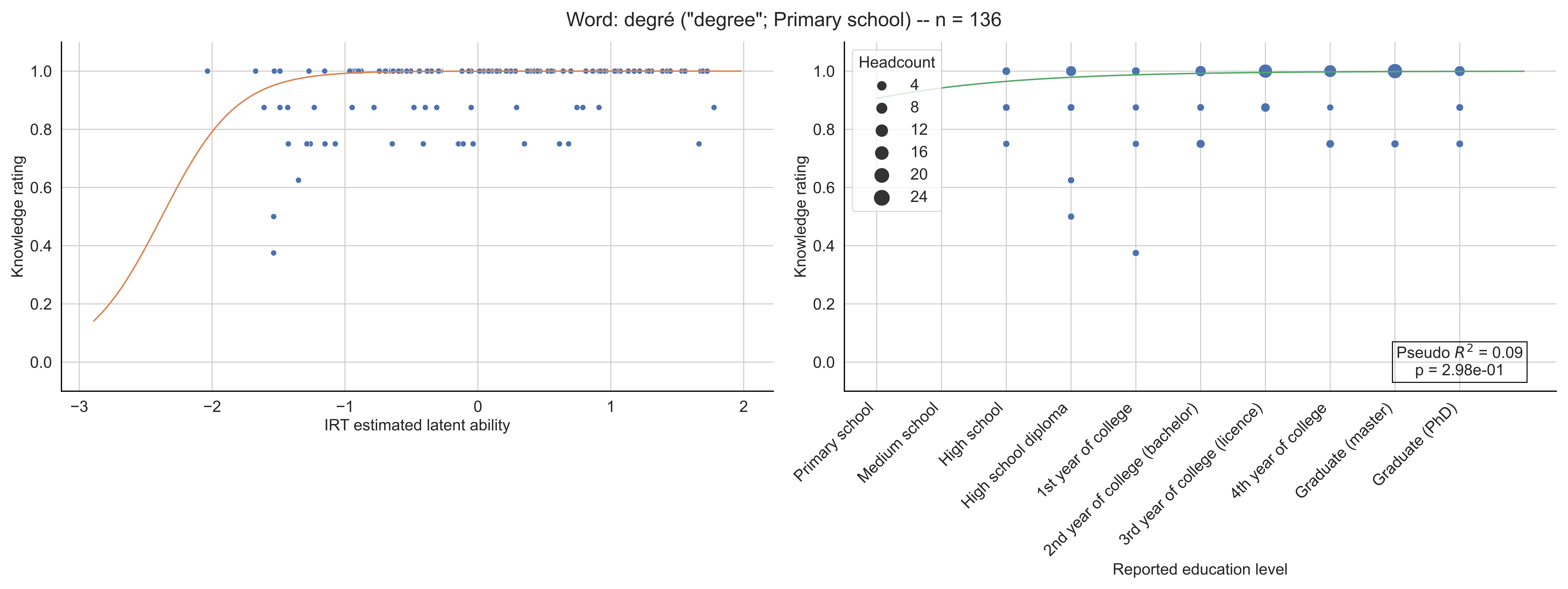

Word: degré ("degree"; Primary school) -- n = 136
Knowledge rating
IRT estimated latent ability
Headcount
4
8
12
16
20
24
Pseudo R^2 = 0.09
p = 2.98e-01
Primary school
Medium school
High school
High school diploma
1st year of college
2nd year of college (bachelor)
3rd year of college (licence)
4th year of college
Graduate (master)
Graduate (PhD)
Reported education level

Word: densité ("density"; 11-12th grade) -- n = 132

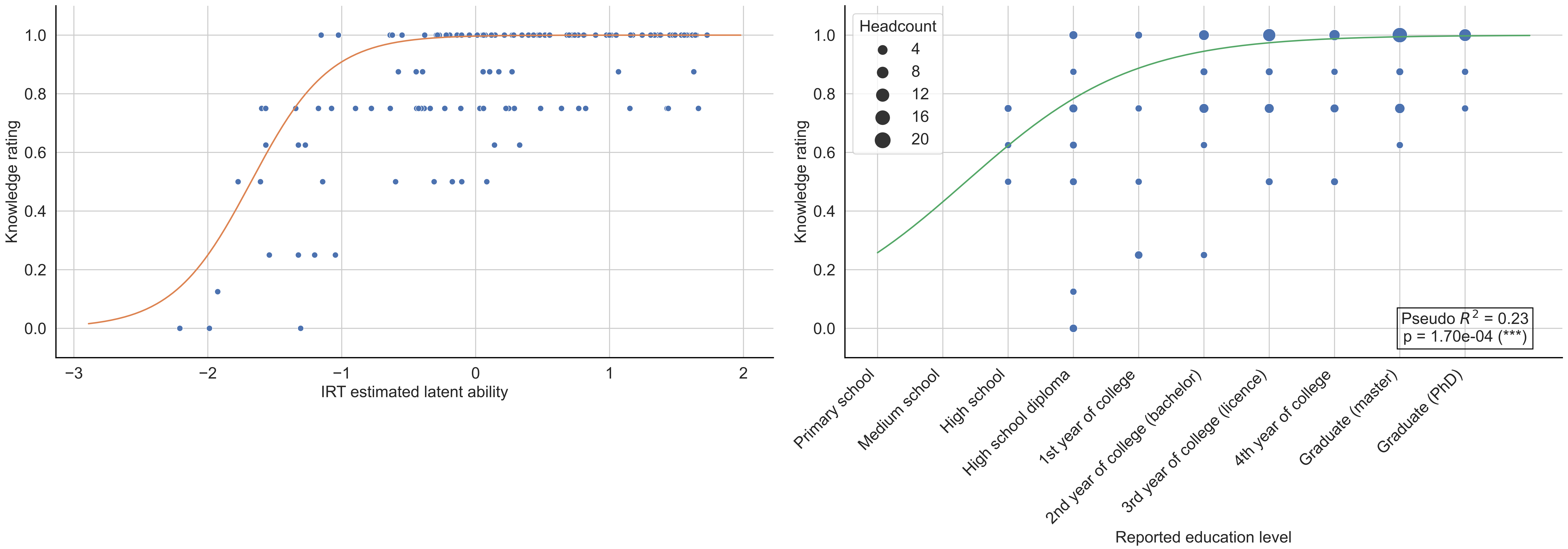

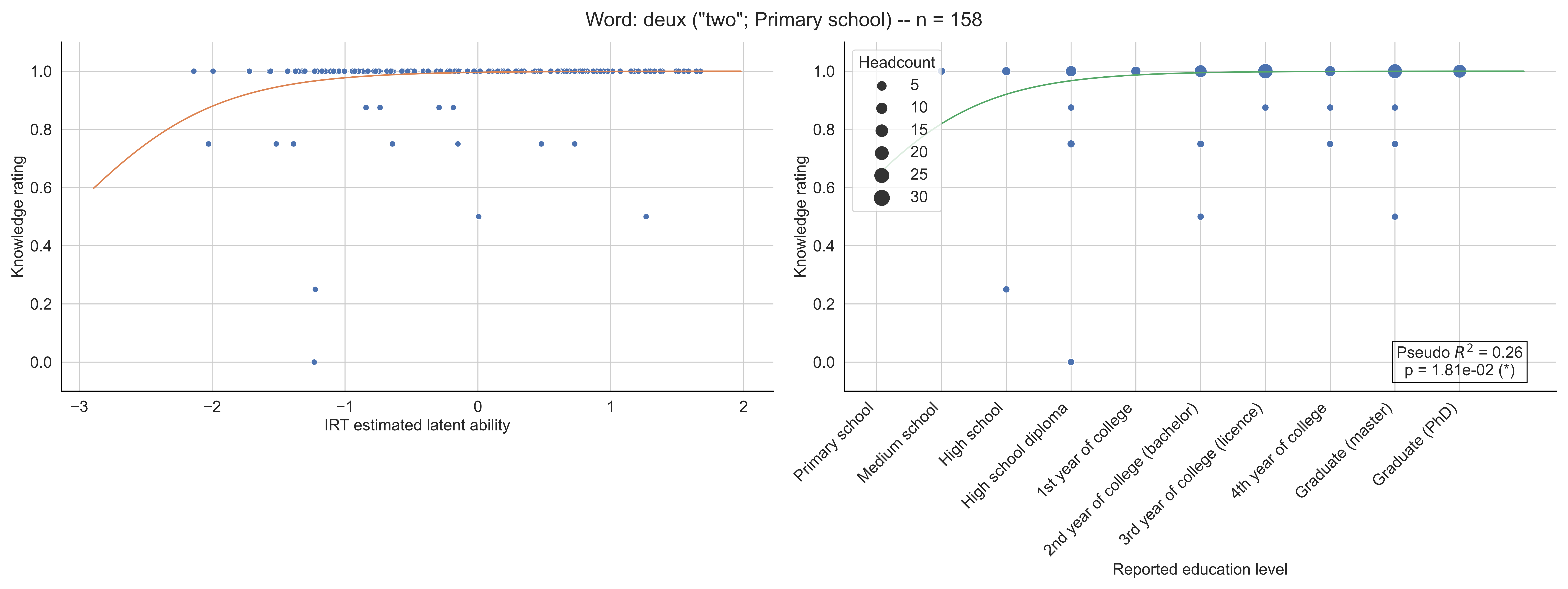

Word: deux ("two"; Primary school) -- n = 158
Knowledge rating
IRT estimated latent ability
Headcount
5
10
15
20
25
30
Pseudo R^2 = 0.26
p = 1.81e-02 (*)
Primary school
Medium school
High school
High school diploma
1st year of college
2nd year of college (bachelor)
3rd year of college (licence)
4th year of college
Graduate (master)
Graduate (PhD)
Reported education level

Word: diagramme ("diagram"; Primary school) -- n = 152

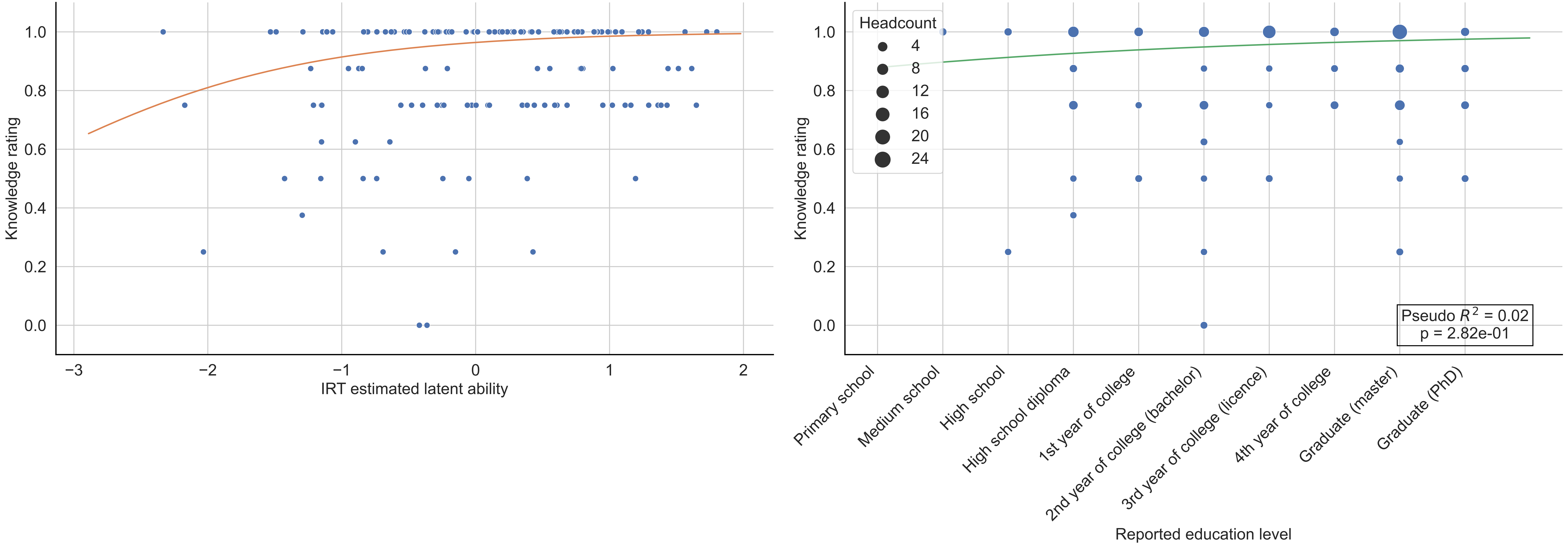

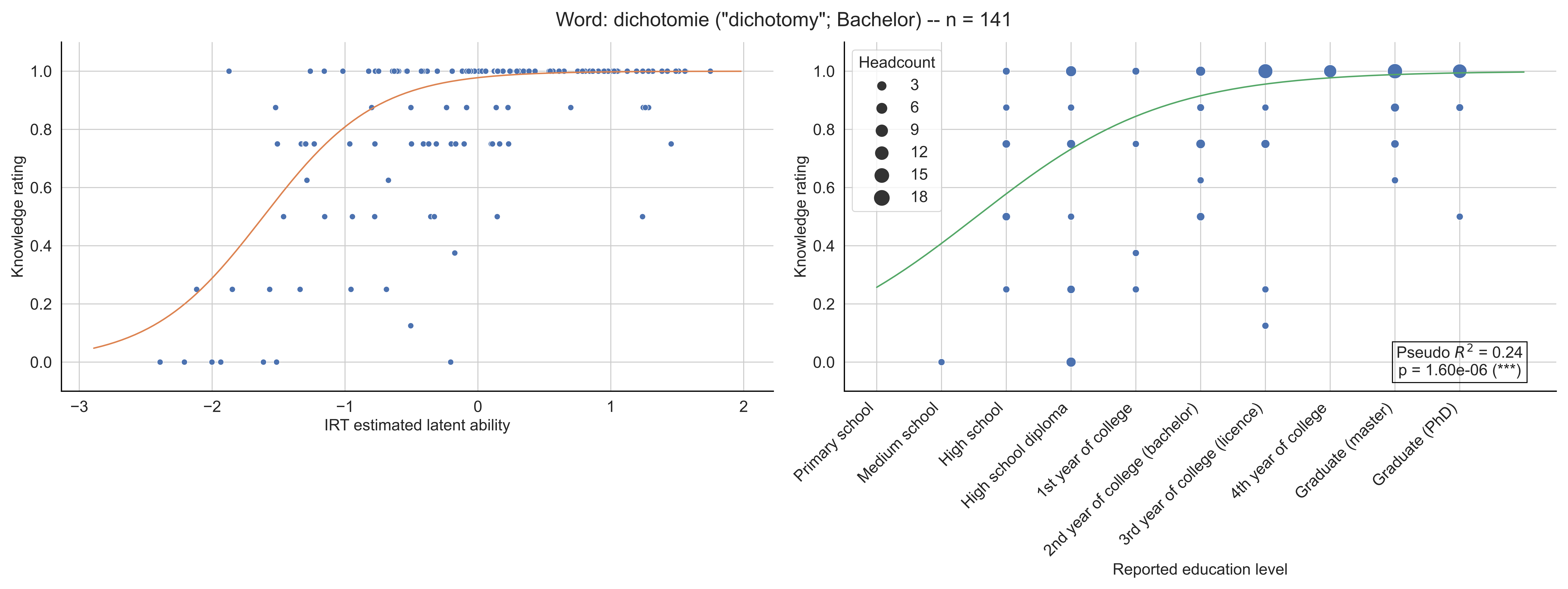

Word: dichotomie ("dichotomy"; Bachelor) -- n = 141
Knowledge rating
IRT estimated latent ability
Headcount
3
6
9
12
15
18
Pseudo R^2 = 0.24
p = 1.60e-06 (***)
Primary school
Medium school
High school
High school diploma
1st year of college
2nd year of college (bachelor)
3rd year of college (licence)
4th year of college
Graduate (master)
Graduate (PhD)
Reported education level

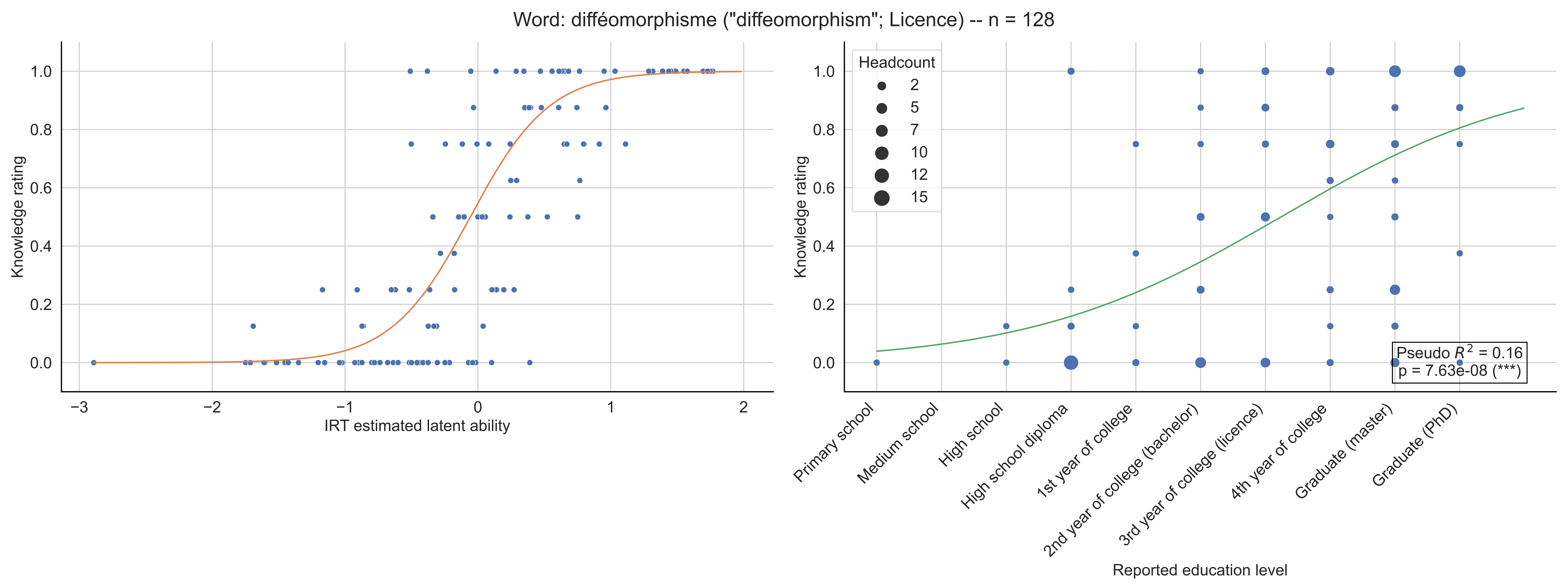
Word: difféomorphisme ("diffeomorphism"; Licence) -- n = 128
Knowledge rating
IRT estimated latent ability
Headcount
2
5
7
10
12
15
Pseudo R^2 = 0.16
p = 7.63e-08 (***)
Primary school
Medium school
High school
High school diploma
1st year of college
2nd year of college (bachelor)
3rd year of college (licence)
4th year of college
Graduate (master)
Graduate (PhD)
Reported education level

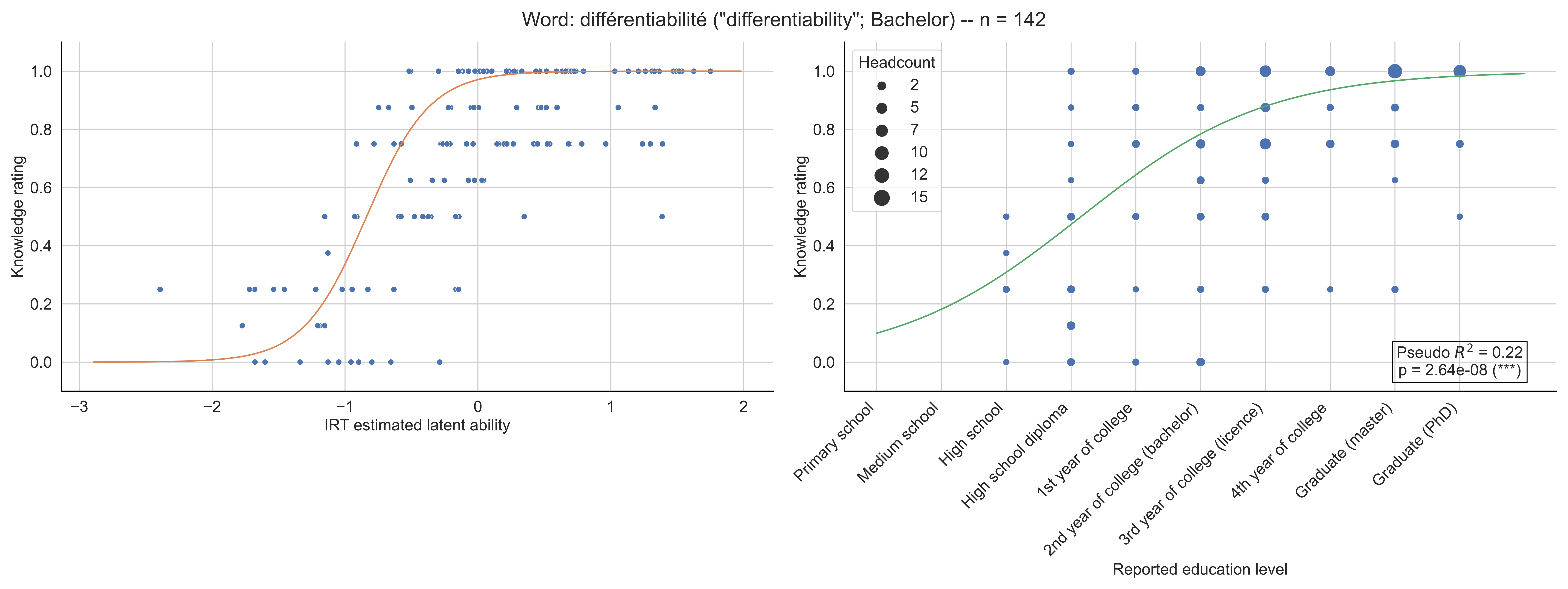

Word: différentiabilité ("differentiability"; Bachelor) -- n = 142
Knowledge rating
IRT estimated latent ability
Headcount
2
5
7
10
12
15
Pseudo R^2 = 0.22
p = 2.64e-08 (***)
Primary school
Medium school
High school
High school diploma
1st year of college
2nd year of college (bachelor)
3rd year of college (licence)
4th year of college
Graduate (master)
Graduate (PhD)
Reported education level

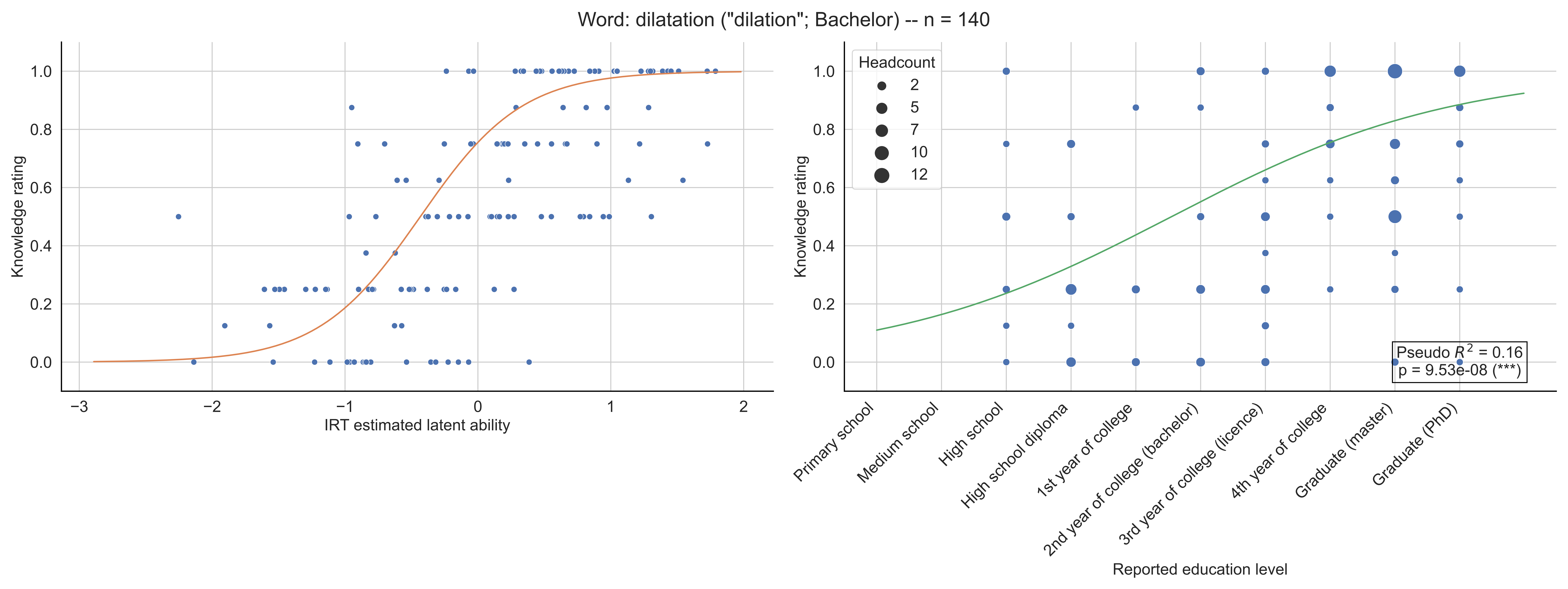
Word: dilatation ("dilation"; Bachelor) -- n = 140
Knowledge rating
IRT estimated latent ability
Headcount
2
5
7
10
12
Pseudo R^2 = 0.16
p = 9.53e-08 (***)
Primary school
Medium school
High school
High school diploma
1st year of college
2nd year of college (bachelor)
3rd year of college (licence)
4th year of college
Graduate (master)
Graduate (PhD)
Reported education level

Word: dimension ("dimension"; Primary school) -- n = 151

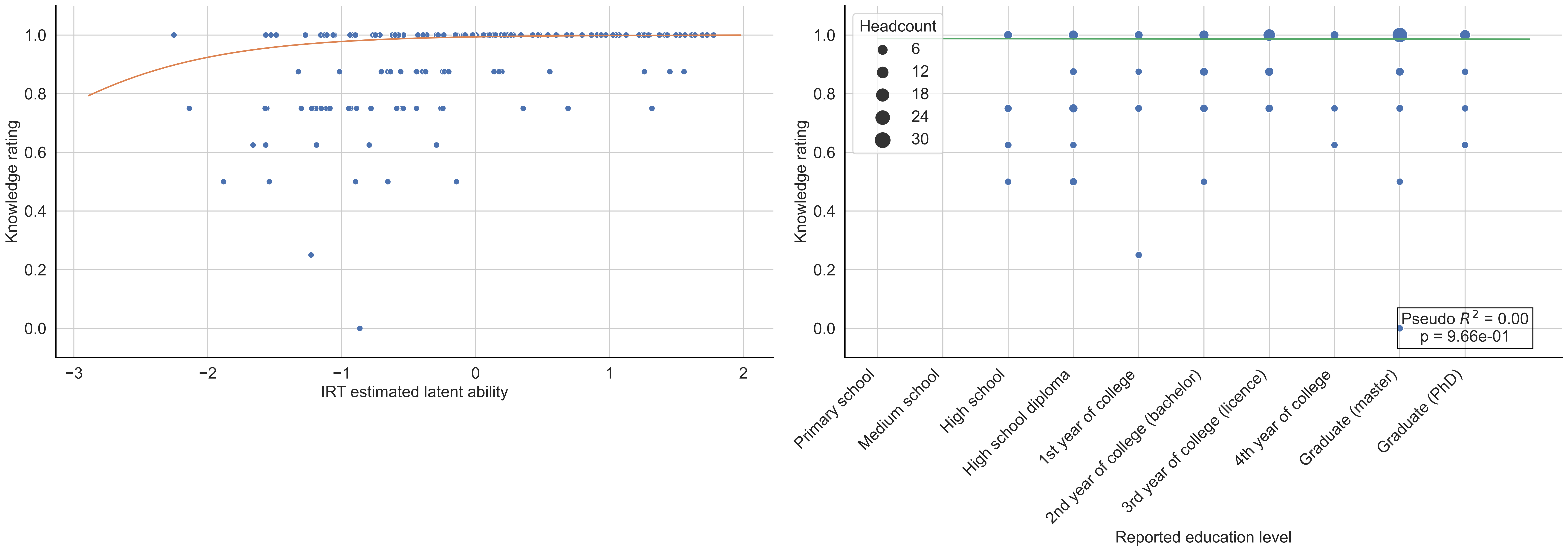

Word: discontinuité ("discontinuity"; 11-12th grade) -- n = 157

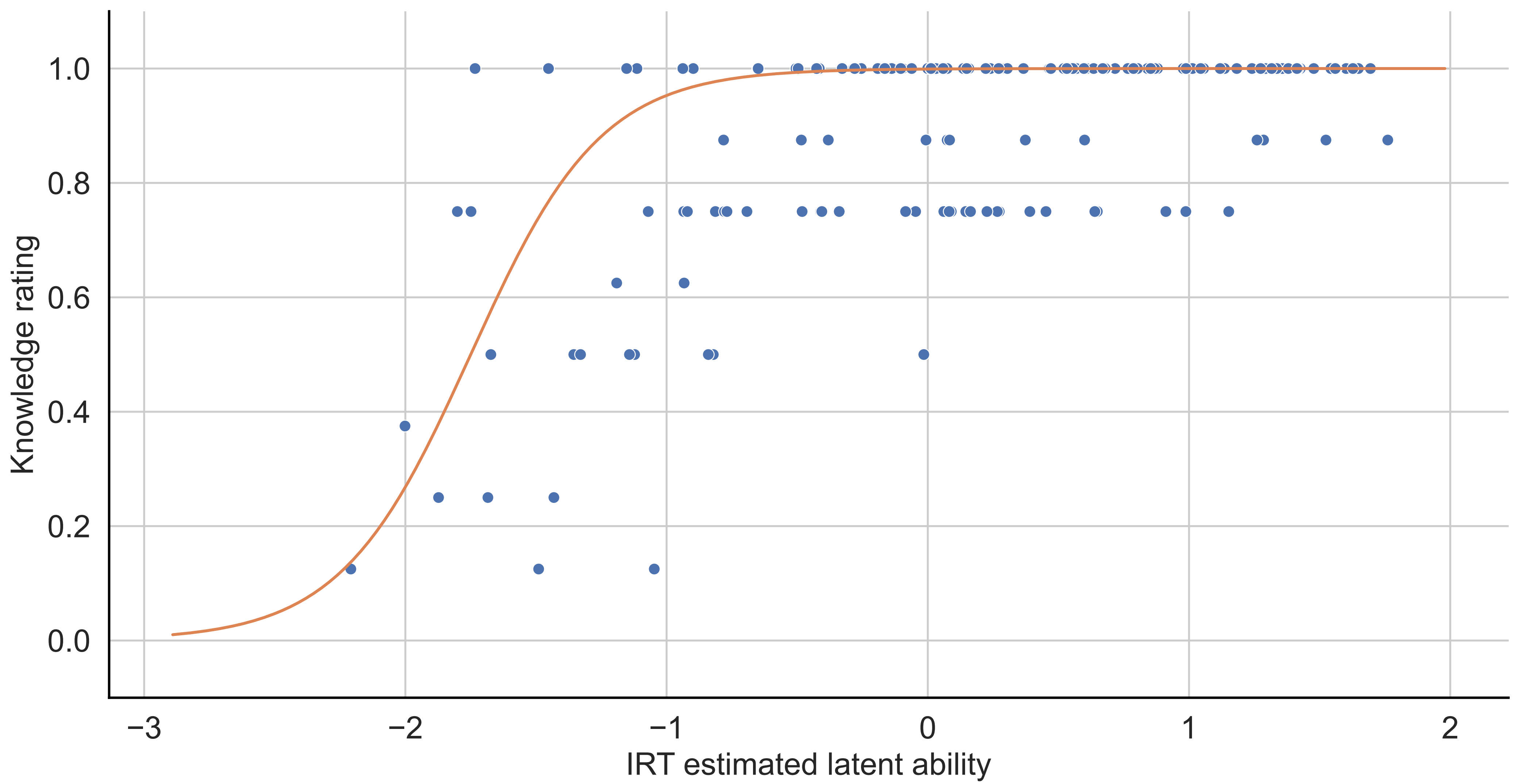

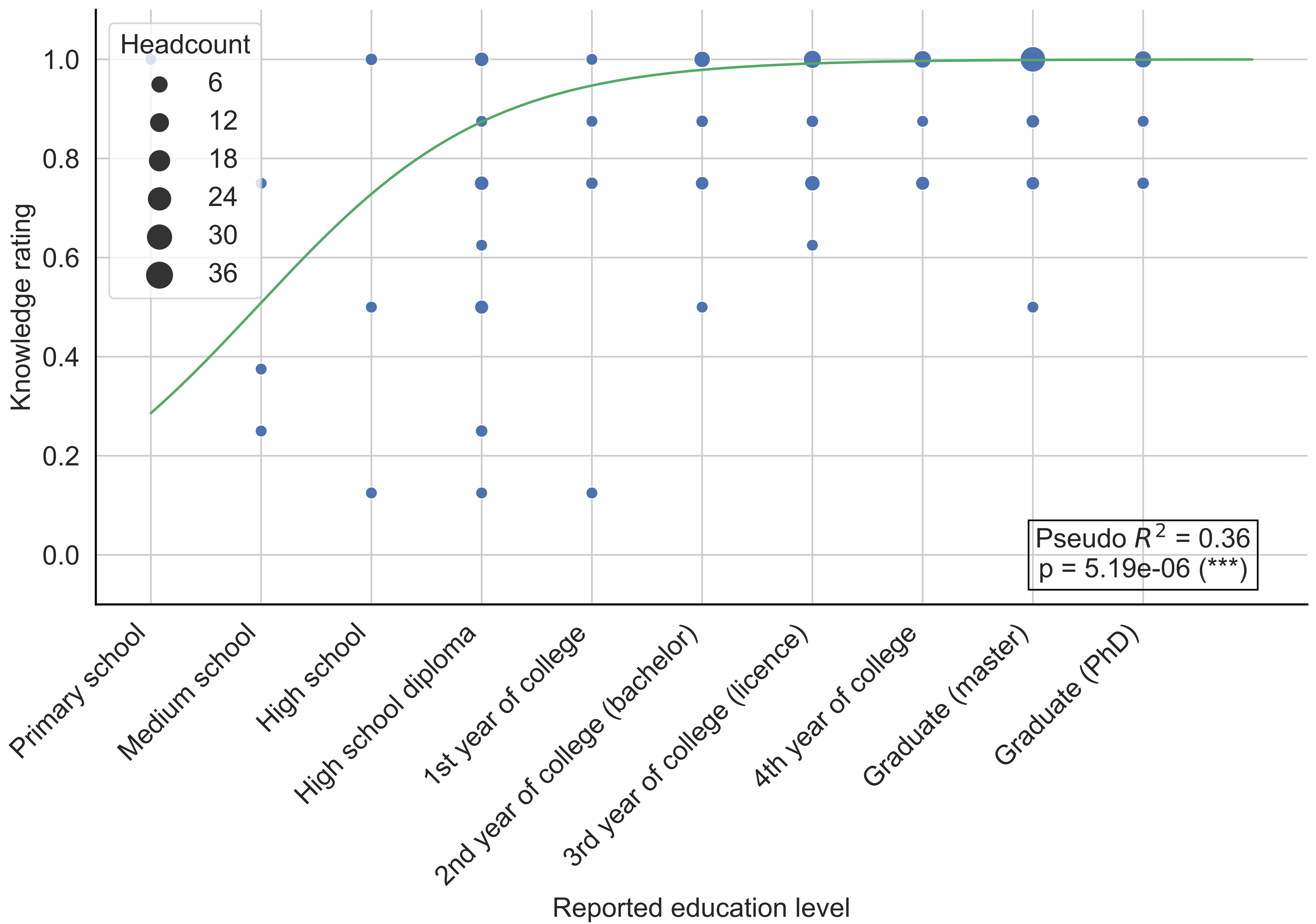

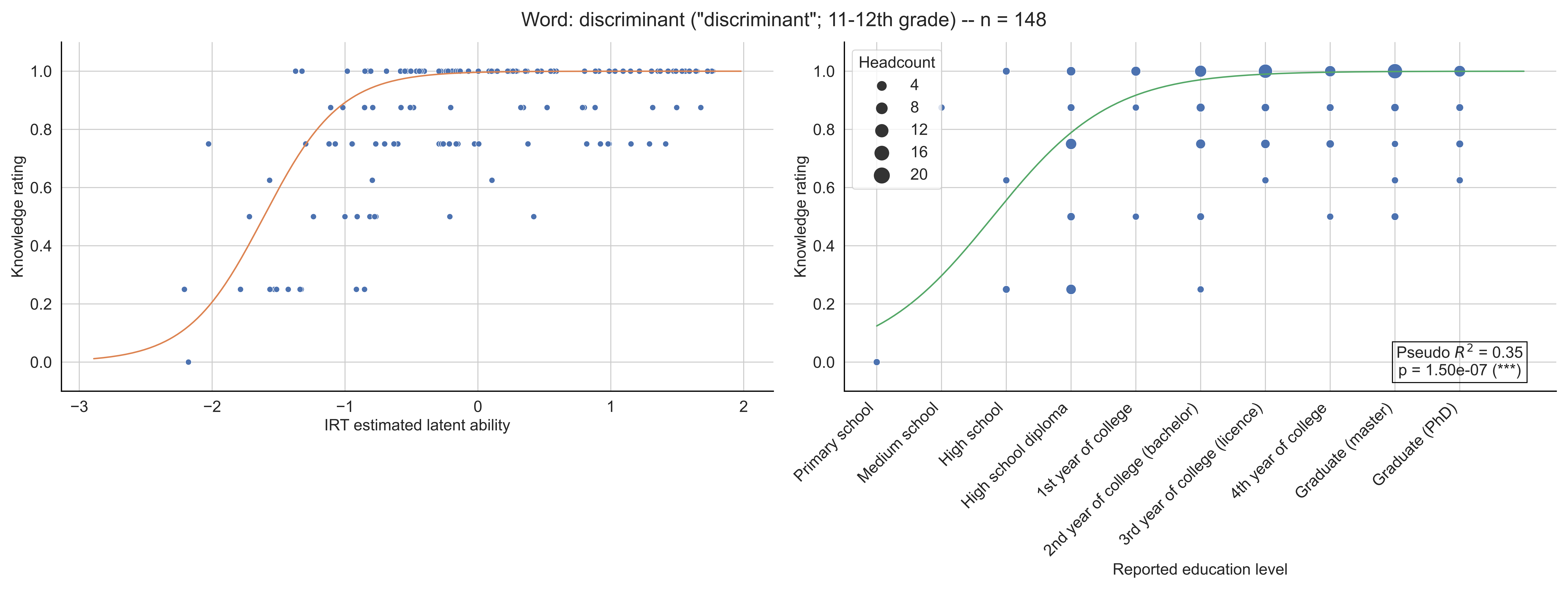

Word: discriminant ("discriminant"; 11-12th grade) -- n = 148
Knowledge rating
IRT estimated latent ability
Headcount
4
8
12
16
20
Pseudo R^2 = 0.35
p = 1.50e-07 (***)
Primary school
Medium school
High school
High school diploma
1st year of college
2nd year of college (bachelor)
3rd year of college (licence)
4th year of college
Graduate (master)
Graduate (PhD)
Reported education level

Word: disque ("disk"; Primary school) -- n = 136

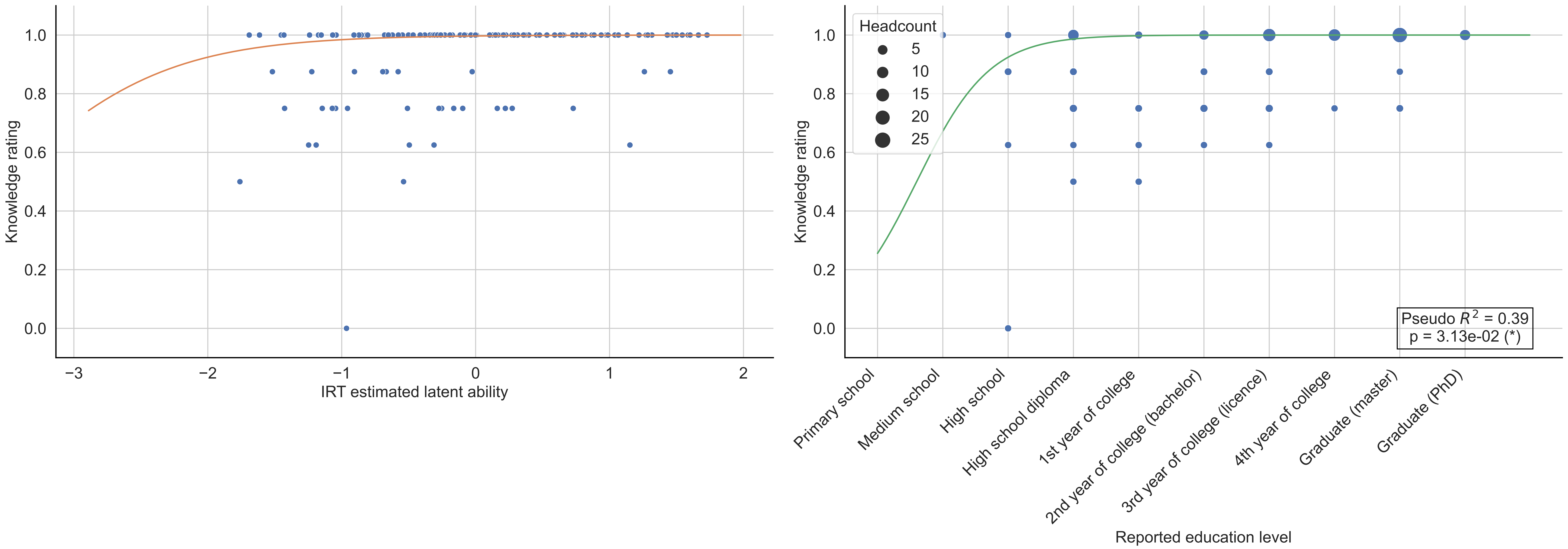

Word: distribution ("distribution"; 11-12th grade) -- n = 132

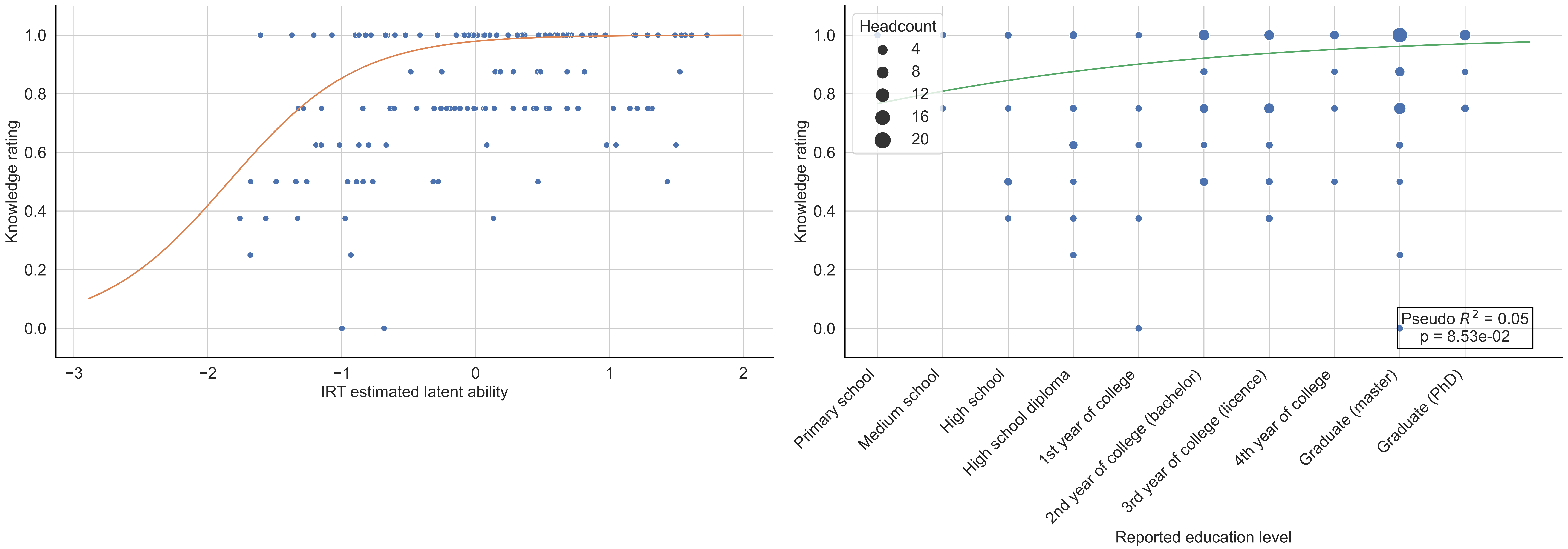

Word: distributivité ("distributivity"; 8-9th grade) -- n = 162

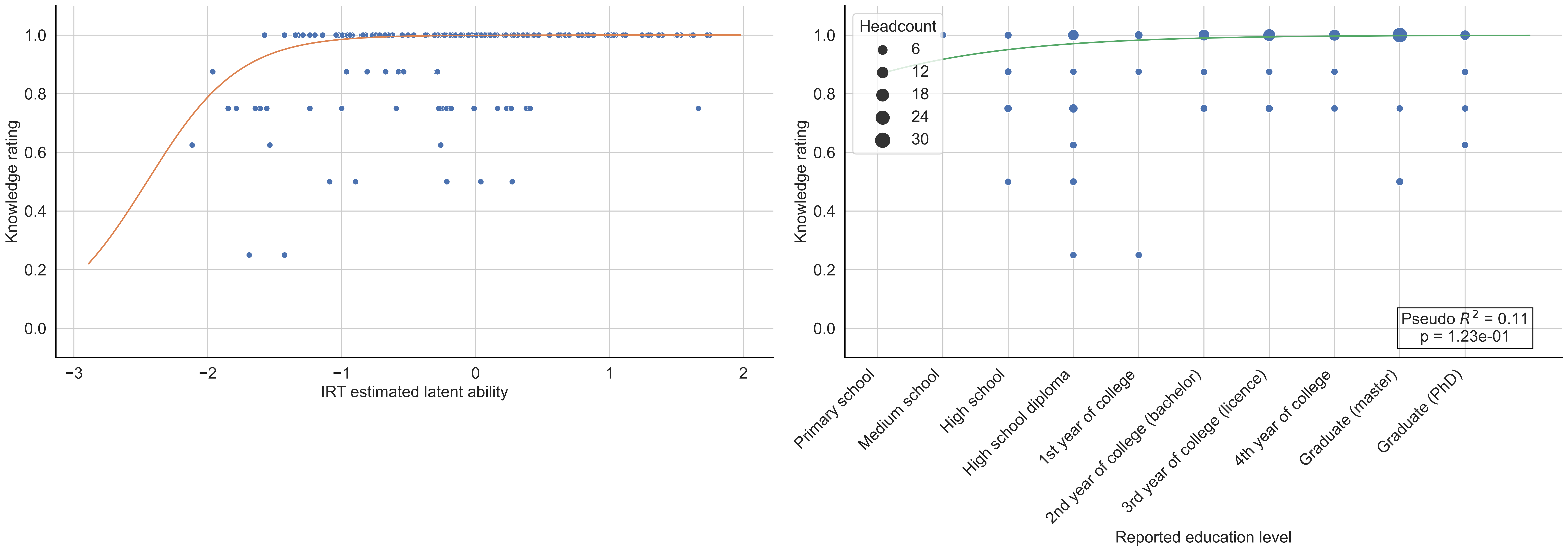

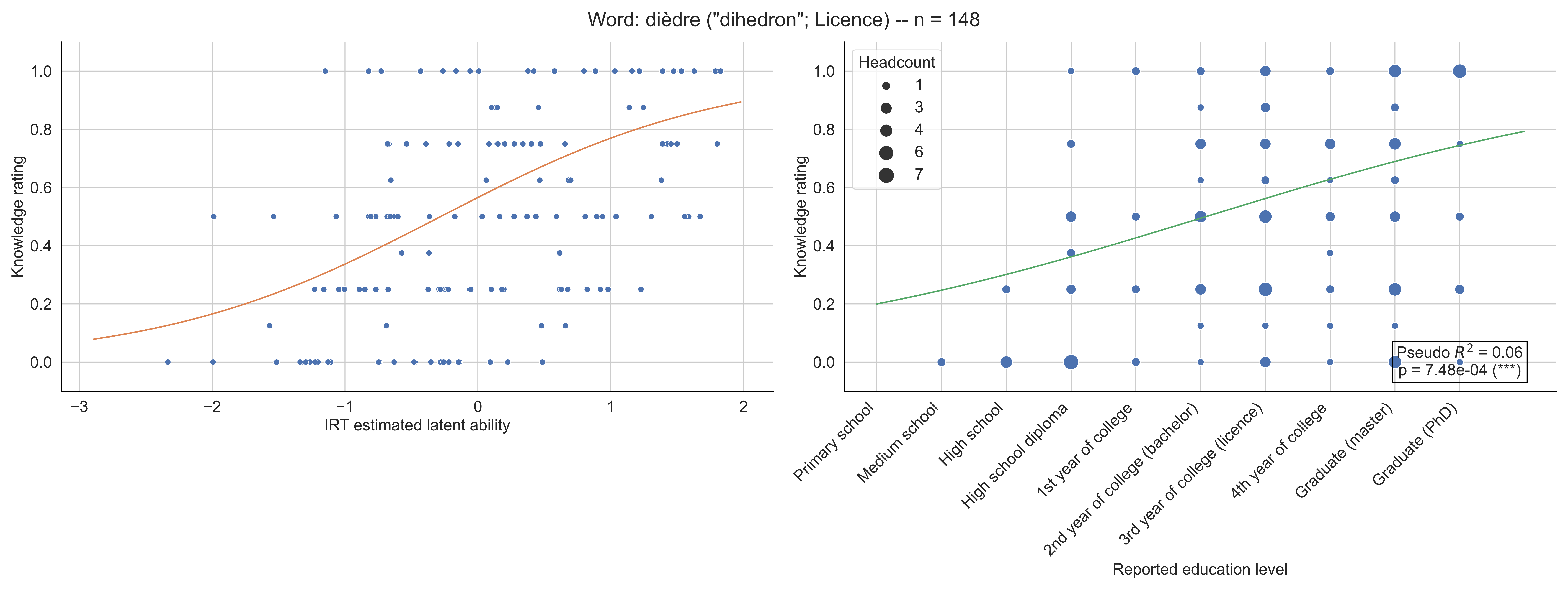
Word: dièdre ("dihedron"; Licence) -- n = 148
Knowledge rating
IRT estimated latent ability
Headcount
1
3
4
6
7
Pseudo R^2 = 0.06
p = 7.48e-04 (***)
Primary school
Medium school
High school
High school diploma
1st year of college
2nd year of college (bachelor)
3rd year of college (licence)
4th year of college
Graduate (master)
Graduate (PhD)
Reported education level

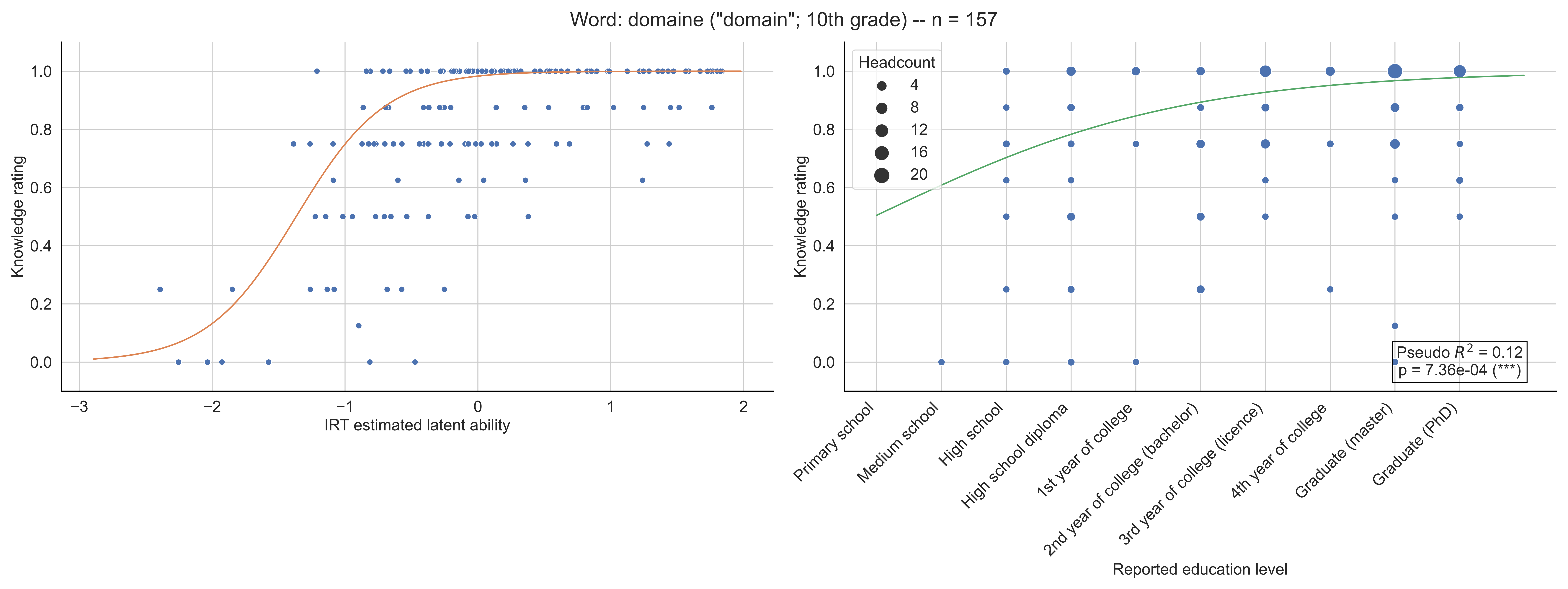
Word: domaine ("domain"; 10th grade) -- n = 157
Knowledge rating
IRT estimated latent ability
Headcount
4
8
12
16
20
Pseudo R^2 = 0.12
p = 7.36e-04 (***)
Primary school
Medium school
High school
High school diploma
1st year of college
2nd year of college (bachelor)
3rd year of college (licence)
4th year of college
Graduate (master)
Graduate (PhD)
Reported education level

Word: douze ("twelve"; Primary school) -- n = 129

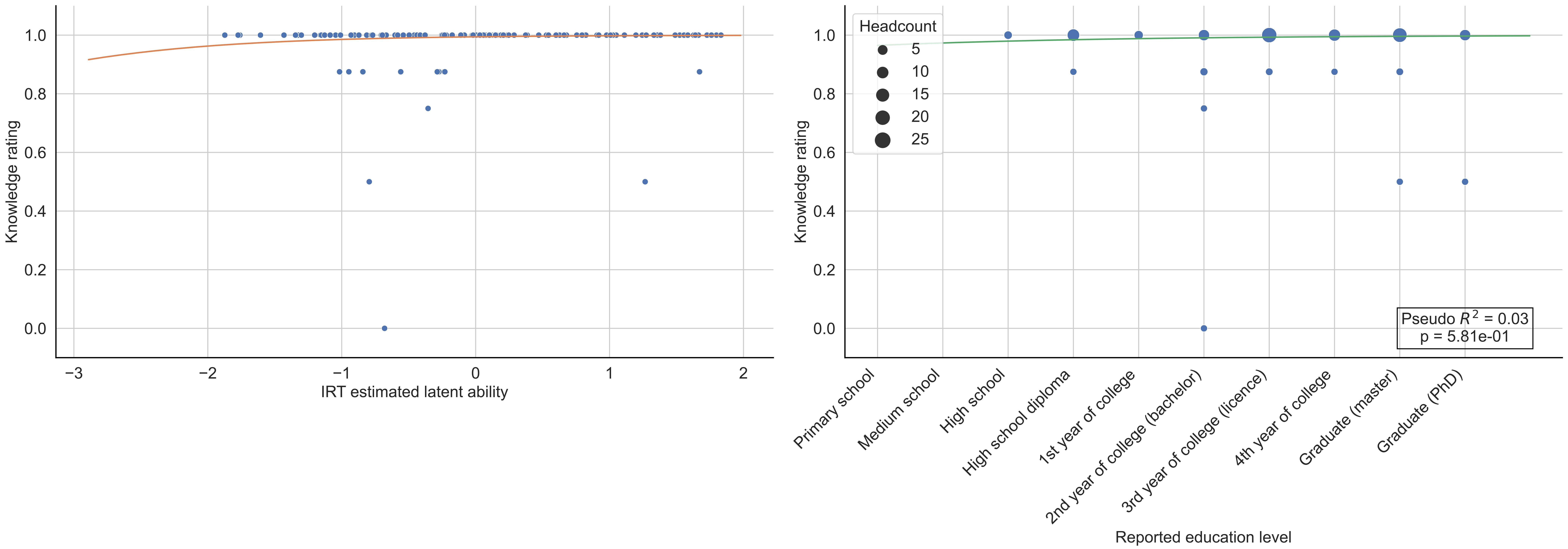

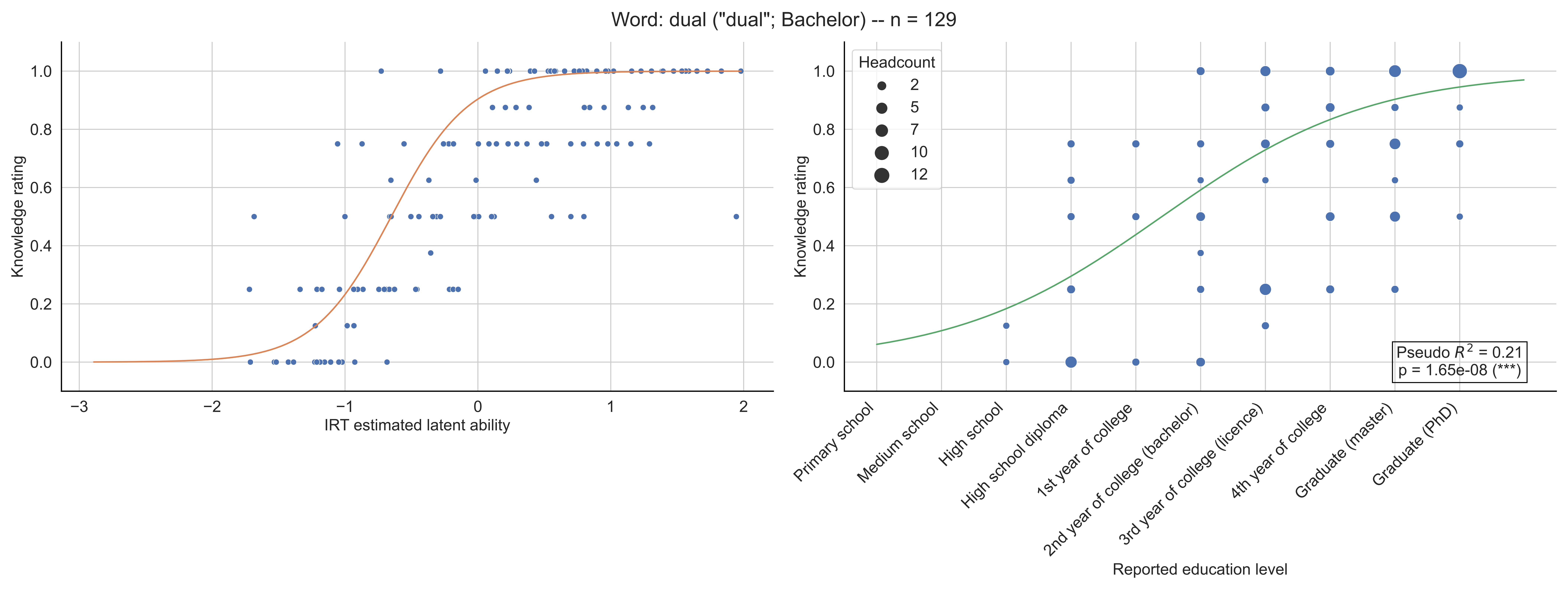
Word: dual ("dual"; Bachelor) -- n = 129
Knowledge rating
IRT estimated latent ability
Headcount
2
5
7
10
12
Pseudo R^2 = 0.21
p = 1.65e-08 (***)
Primary school
Medium school
High school
High school diploma
1st year of college
2nd year of college (bachelor)
3rd year of college (licence)
4th year of college
Graduate (master)
Graduate (PhD)
Reported education level

Word: dualité ("duality"; Bachelor) -- n = 135

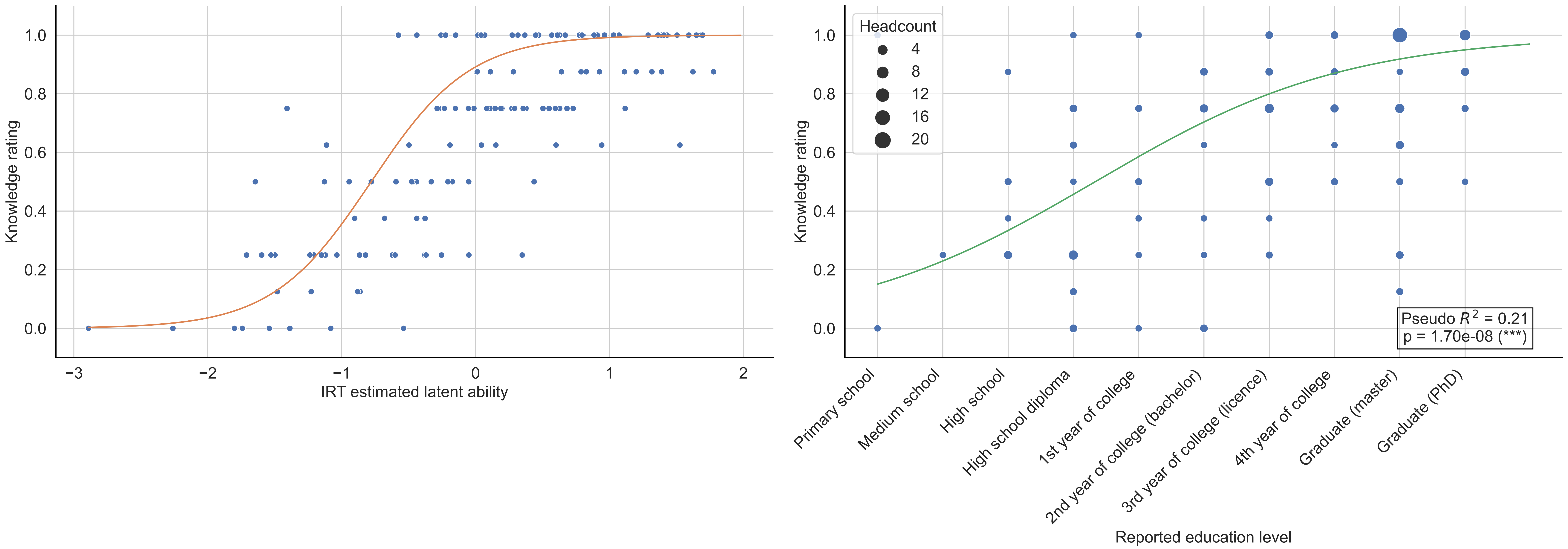

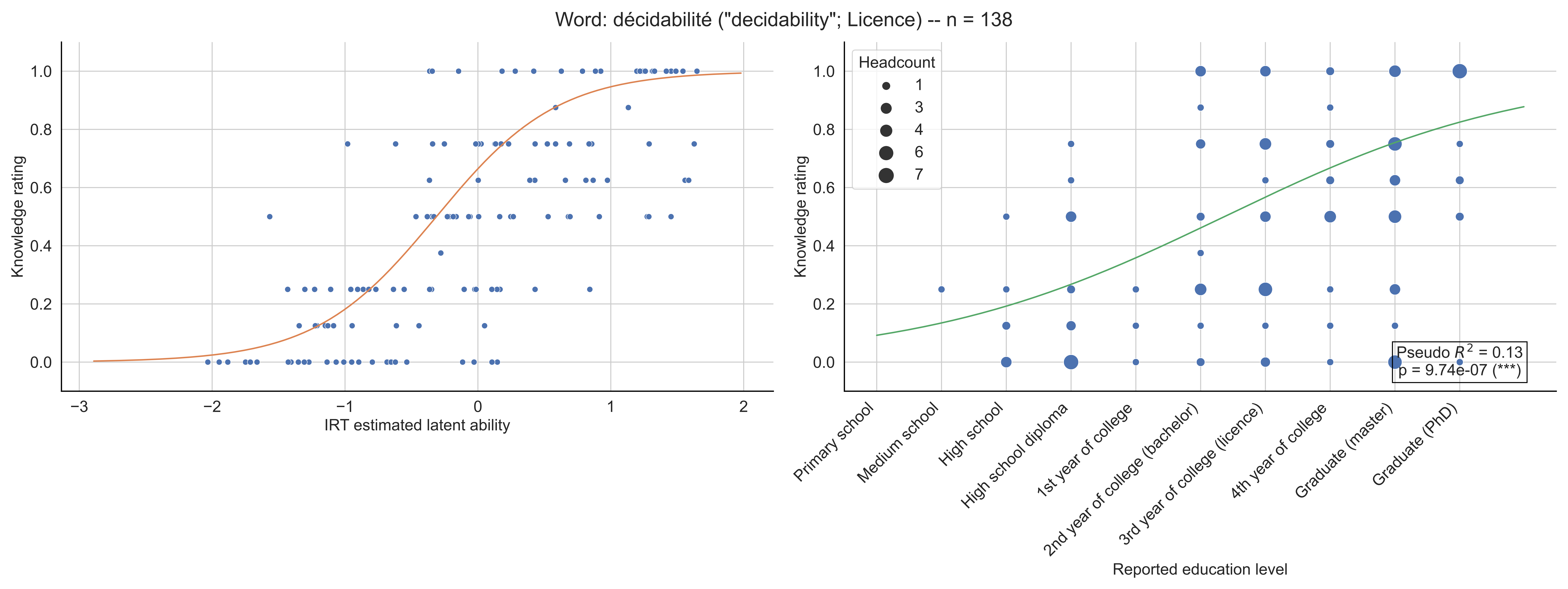
Word: décidabilité ("decidability"; Licence) -- n = 138
Knowledge rating
IRT estimated latent ability
Headcount
1
3
4
6
7
Pseudo R^2 = 0.13
p = 9.74e-07 (***)
Primary school
Medium school
High school
High school diploma
1st year of college
2nd year of college (bachelor)
3rd year of college (licence)
4th year of college
Graduate (master)
Graduate (PhD)
Reported education level

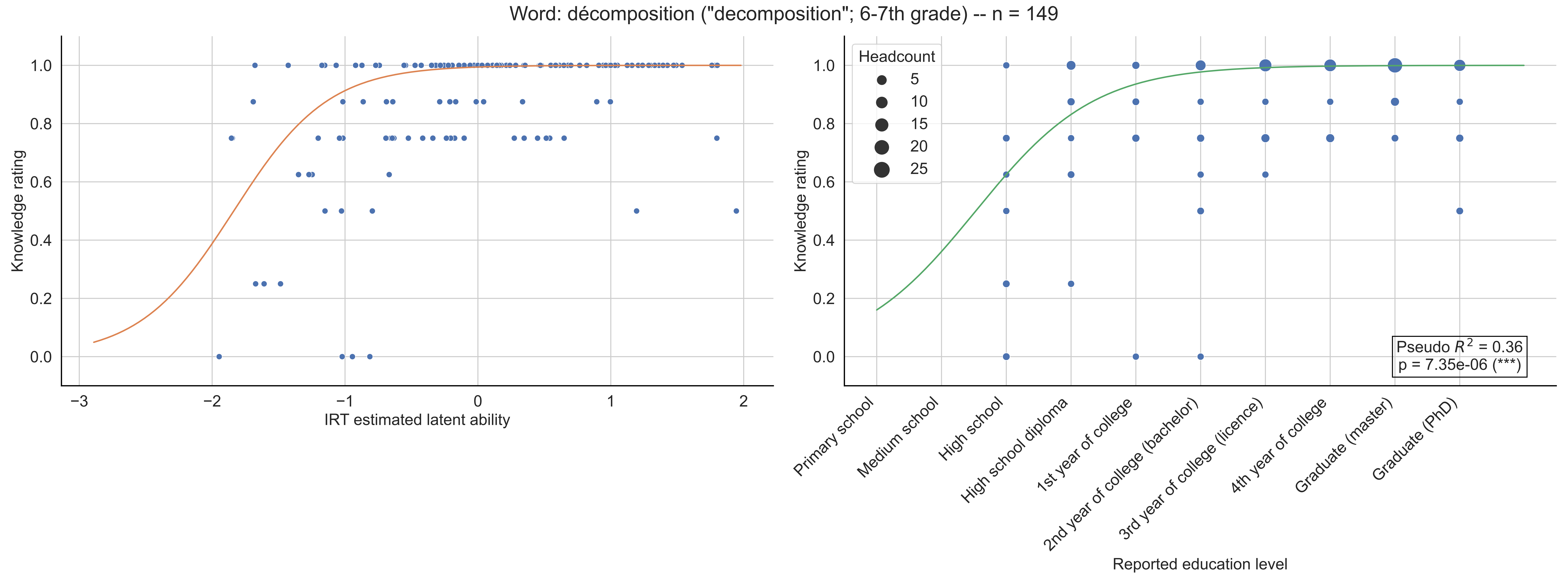

Word: décomposition ("decomposition"; 6-7th grade) -- n = 149
Knowledge rating
IRT estimated latent ability
−3
−2
−1
0
1
2
0.0
0.2
0.4
0.6
0.8
1.0
Headcount
5
10
15
20
25
Pseudo R^2 = 0.36
p = 7.35e-06 (***)
Primary school
Medium school
High school
High school diploma
1st year of college
2nd year of college (bachelor)
3rd year of college (licence)
4th year of college
Graduate (master)
Graduate (PhD)
Reported education level

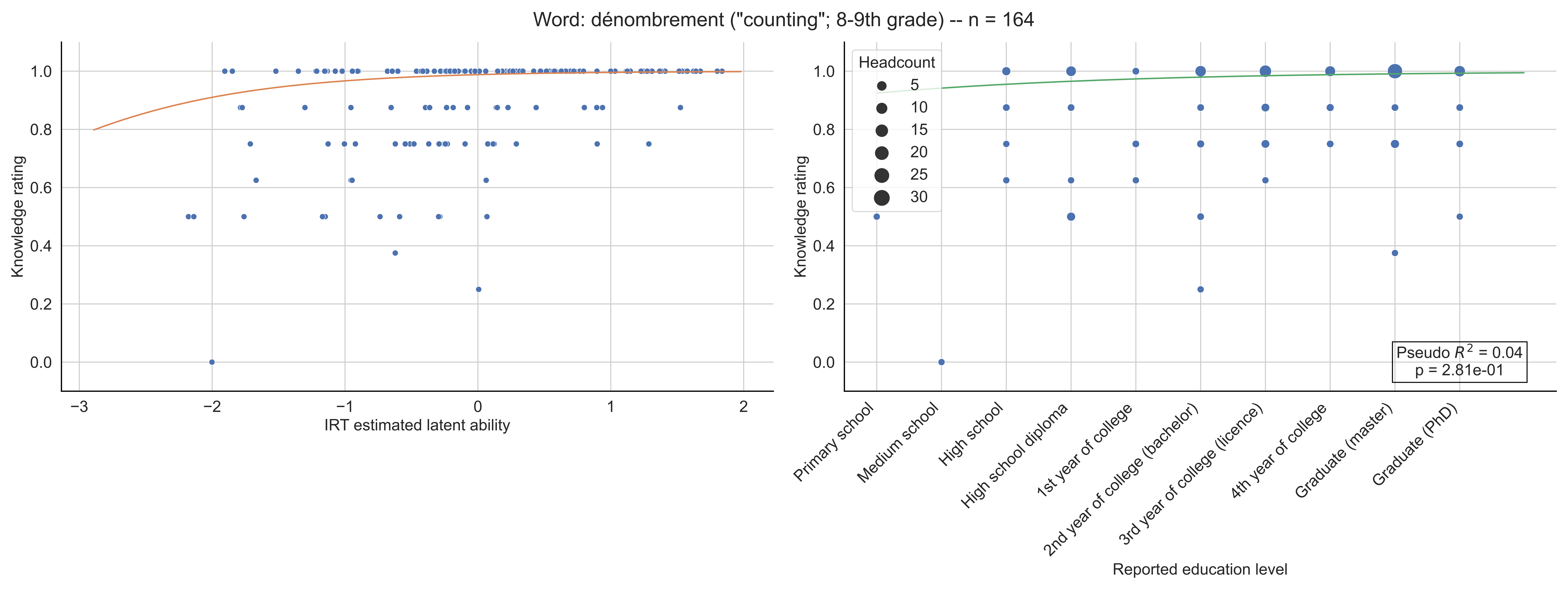

Word: dénombrement ("counting"; 8-9th grade) -- n = 164
Knowledge rating
IRT estimated latent ability
Headcount
5
10
15
20
25
30
Pseudo R^2 = 0.04
p = 2.81e-01
Primary school
Medium school
High school
High school diploma
1st year of college
2nd year of college (bachelor)
3rd year of college (licence)
4th year of college
Graduate (master)
Graduate (PhD)
Reported education level

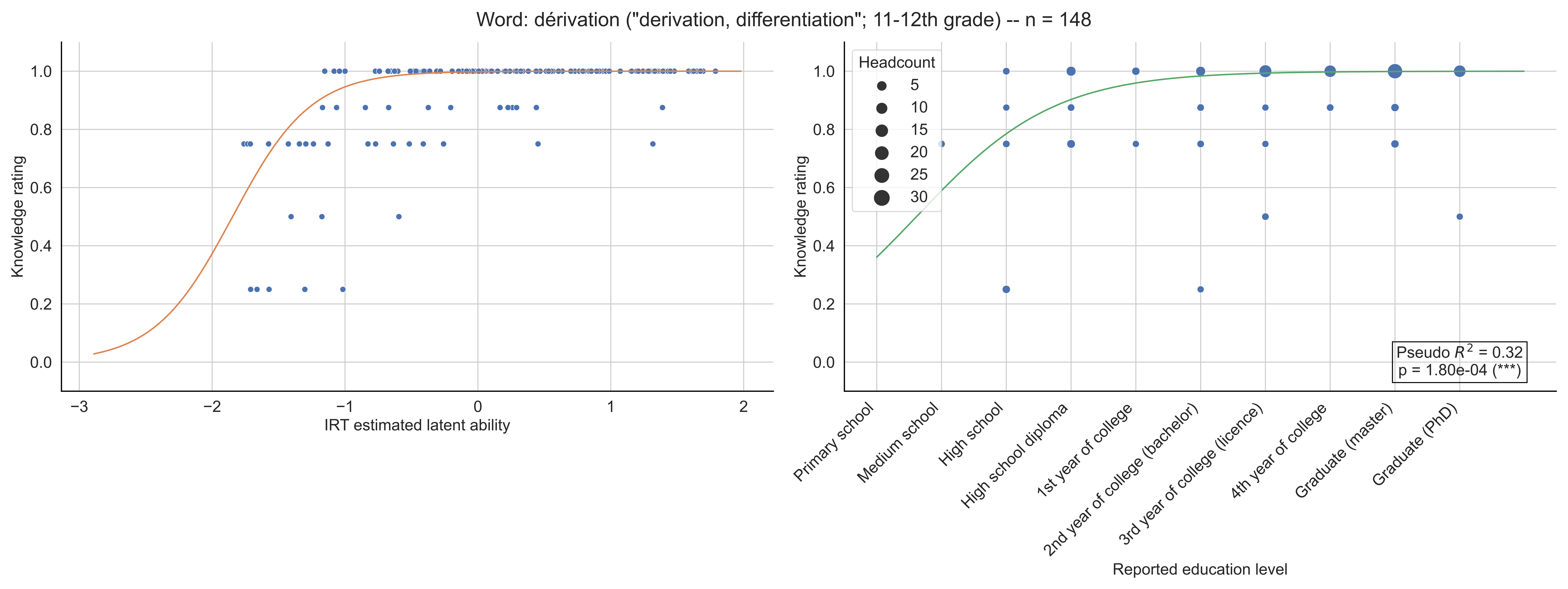

Word: dérivation ("derivation, differentiation"; 11-12th grade) -- n = 148
Knowledge rating
IRT estimated latent ability
Headcount
5
10
15
20
25
30
Pseudo R^2 = 0.32
p = 1.80e-04 (***)
Primary school
Medium school
High school
High school diploma
1st year of college
2nd year of college (bachelor)
3rd year of college (licence)
4th year of college
Graduate (master)
Graduate (PhD)
Reported education level

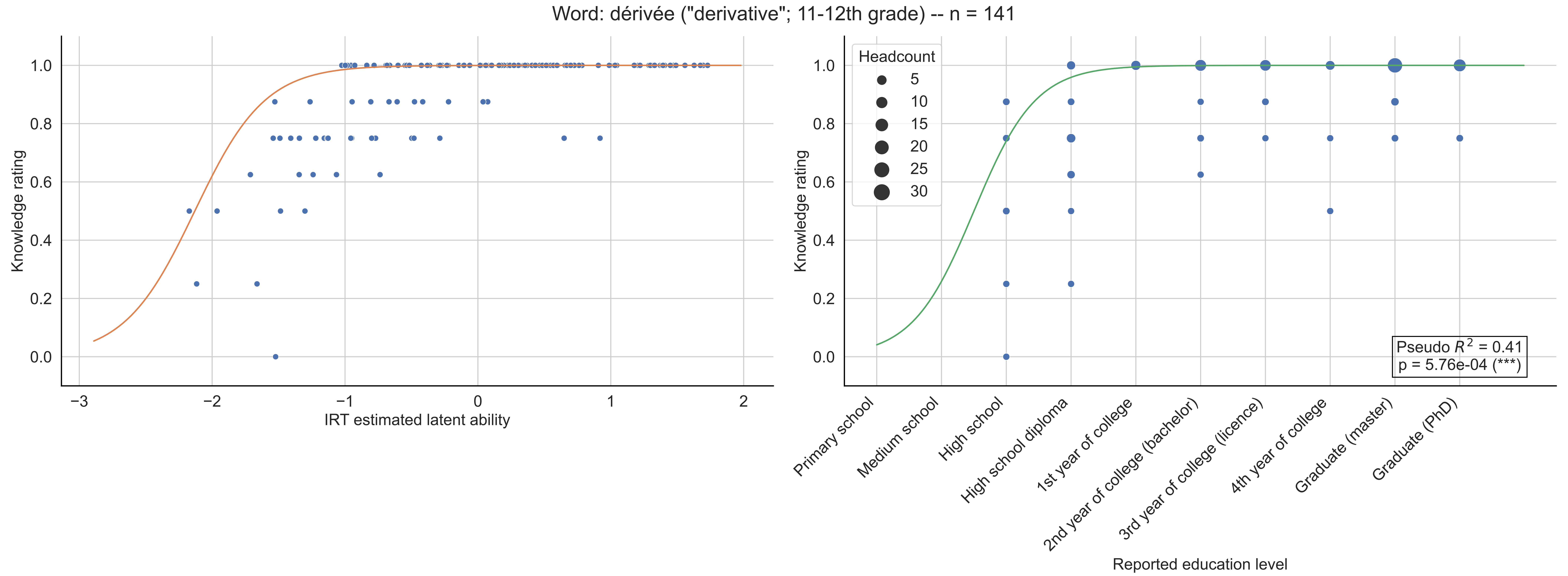

Word: dérivée ("derivative"; 11-12th grade) -- n = 141
Knowledge rating
IRT estimated latent ability
Headcount
5
10
15
20
25
30
Primary school
Medium school
High school
High school diploma
1st year of college
2nd year of college (bachelor)
3rd year of college (licence)
4th year of college
Graduate (master)
Graduate (PhD)
Reported education level
Pseudo R^2 = 0.41
p = 5.76e-04 (***)

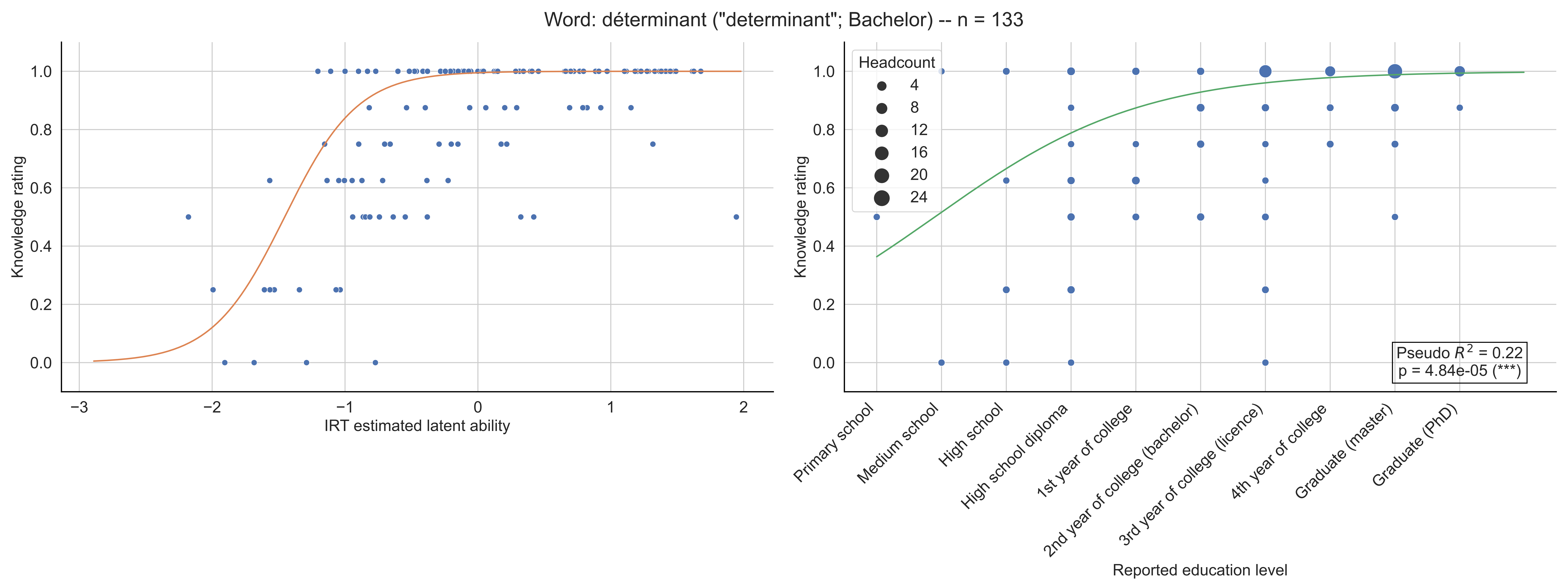

Word: déterminant ("determinant"; Bachelor) -- n = 133
Knowledge rating
IRT estimated latent ability
Headcount
4
8
12
16
20
24
Pseudo R^2 = 0.22
p = 4.84e-05 (***)
Primary school
Medium school
High school
High school diploma
1st year of college
2nd year of college (bachelor)
3rd year of college (licence)
4th year of college
Graduate (master)
Graduate (PhD)
Reported education level

Word: ellipsoïde ("ellipsoid"; Bachelor) -- n = 146

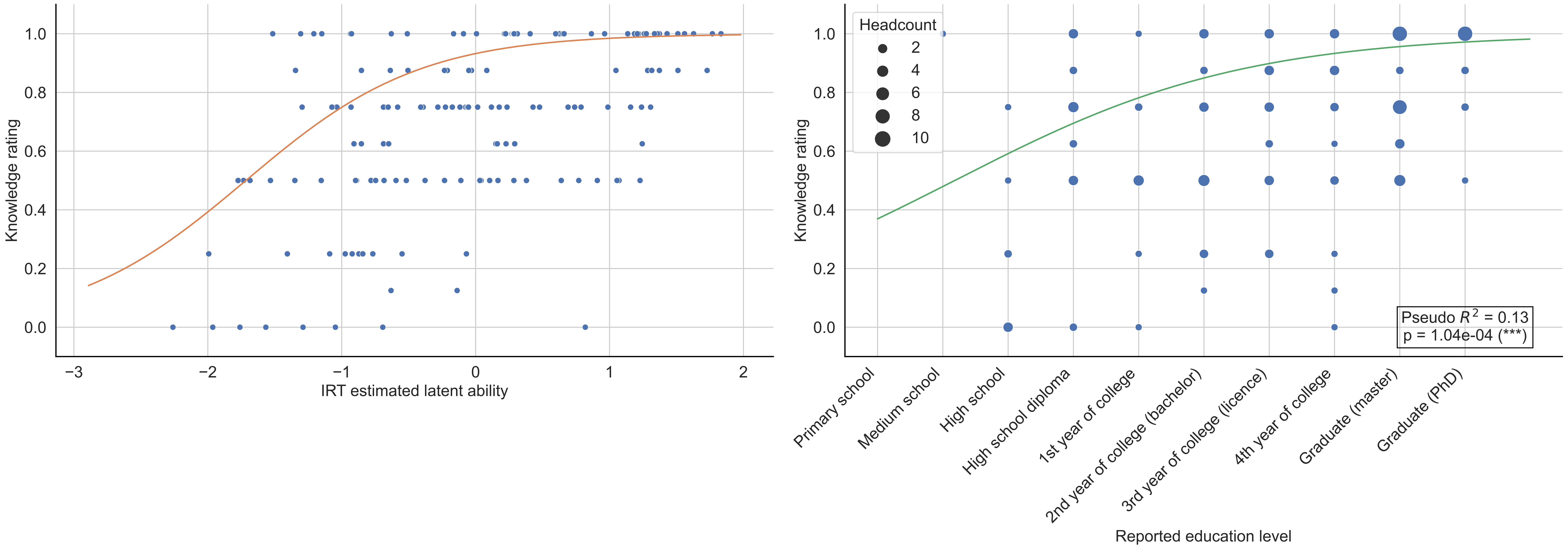

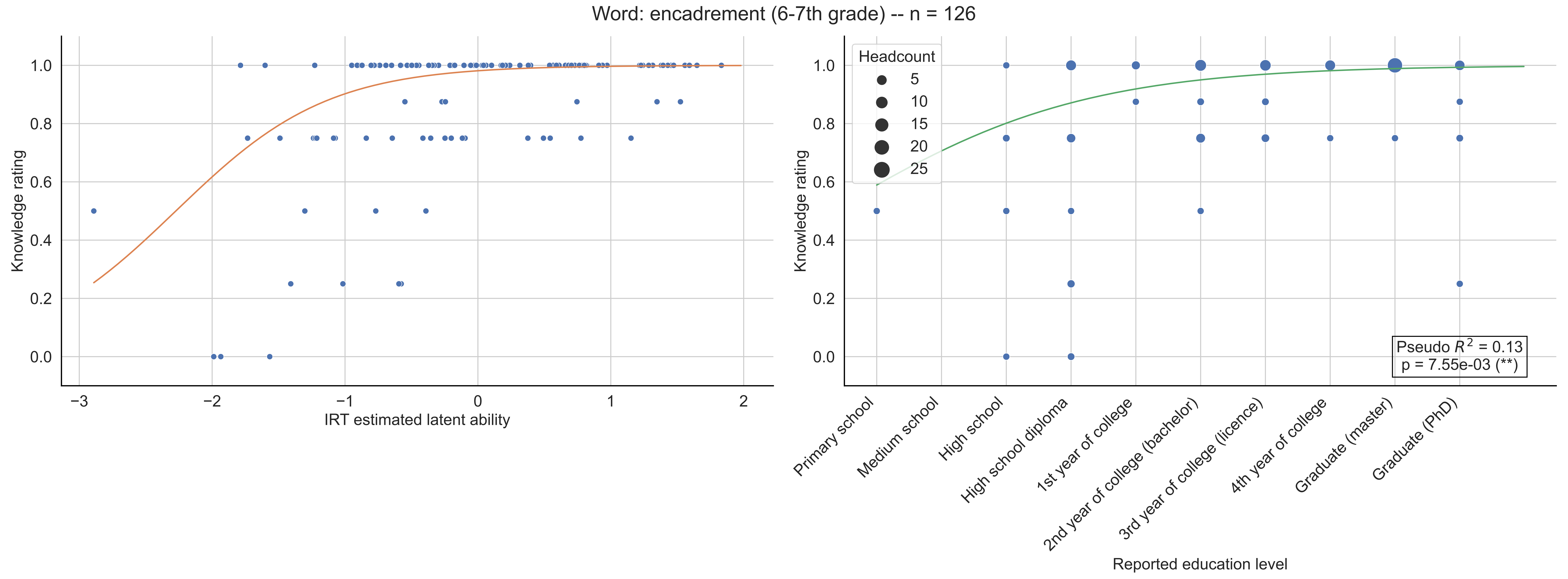

Word: encadrement (6-7th grade) -- n = 126
Knowledge rating
IRT estimated latent ability
Headcount
5
10
15
20
25
Pseudo R^2 = 0.13
p = 7.55e-03 (**)
Primary school
Medium school
High school
High school diploma
1st year of college
2nd year of college (bachelor)
3rd year of college (licence)
4th year of college
Graduate (master)
Graduate (PhD)
Reported education level

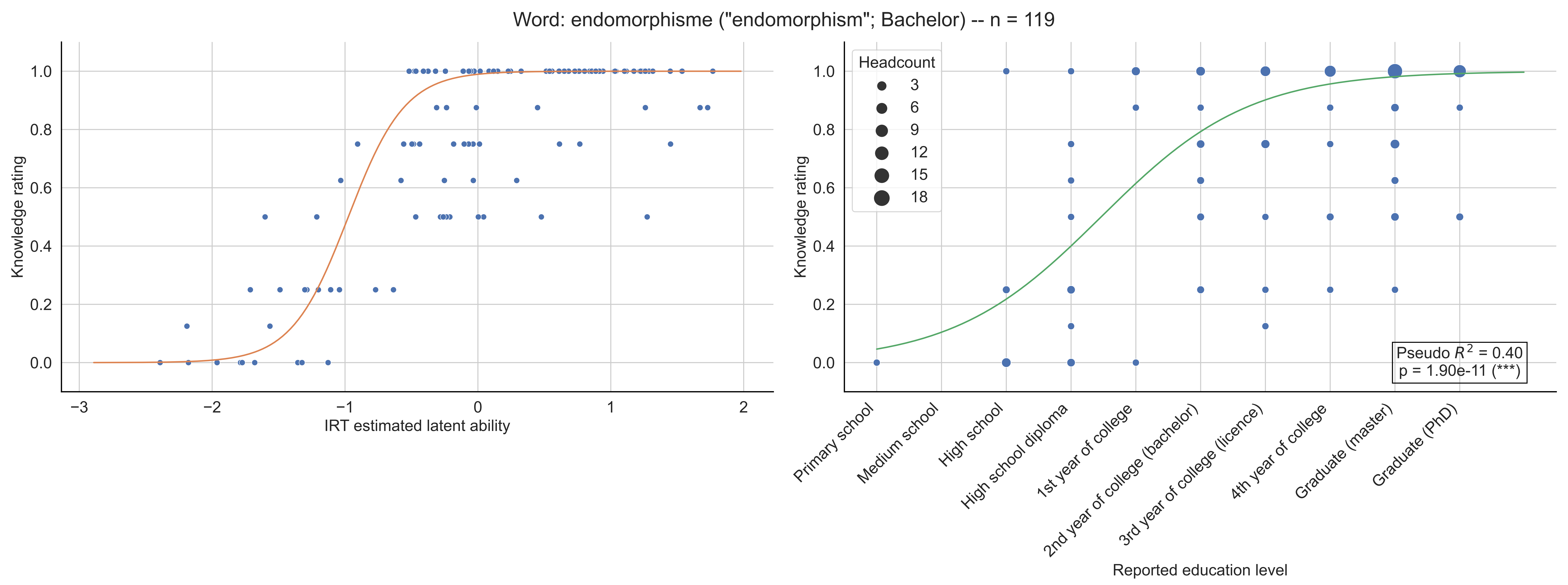
Word: endomorphisme ("endomorphism"; Bachelor) -- n = 119
Knowledge rating
IRT estimated latent ability
Headcount
3
6
9
12
15
18
Pseudo R^2 = 0.40
p = 1.90e-11 (***)
Primary school
Medium school
High school
High school diploma
1st year of college
2nd year of college (bachelor)
3rd year of college (licence)
4th year of college
Graduate (master)
Graduate (PhD)
Reported education level

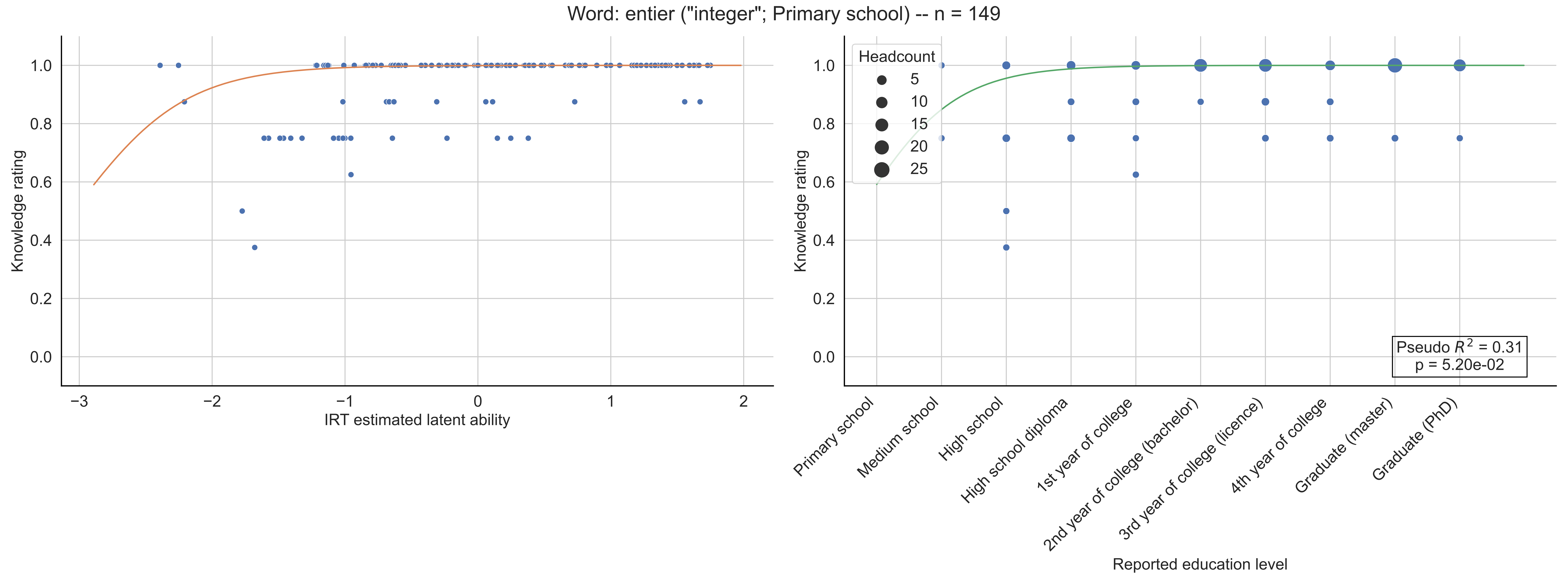

Word: entier ("integer"; Primary school) -- n = 149
Knowledge rating
IRT estimated latent ability
Headcount
5
10
15
20
25
Pseudo R^2 = 0.31
p = 5.20e-02
Primary school
Medium school
High school
High school diploma
1st year of college
2nd year of college (bachelor)
3rd year of college (licence)
4th year of college
Graduate (master)
Graduate (PhD)
Reported education level

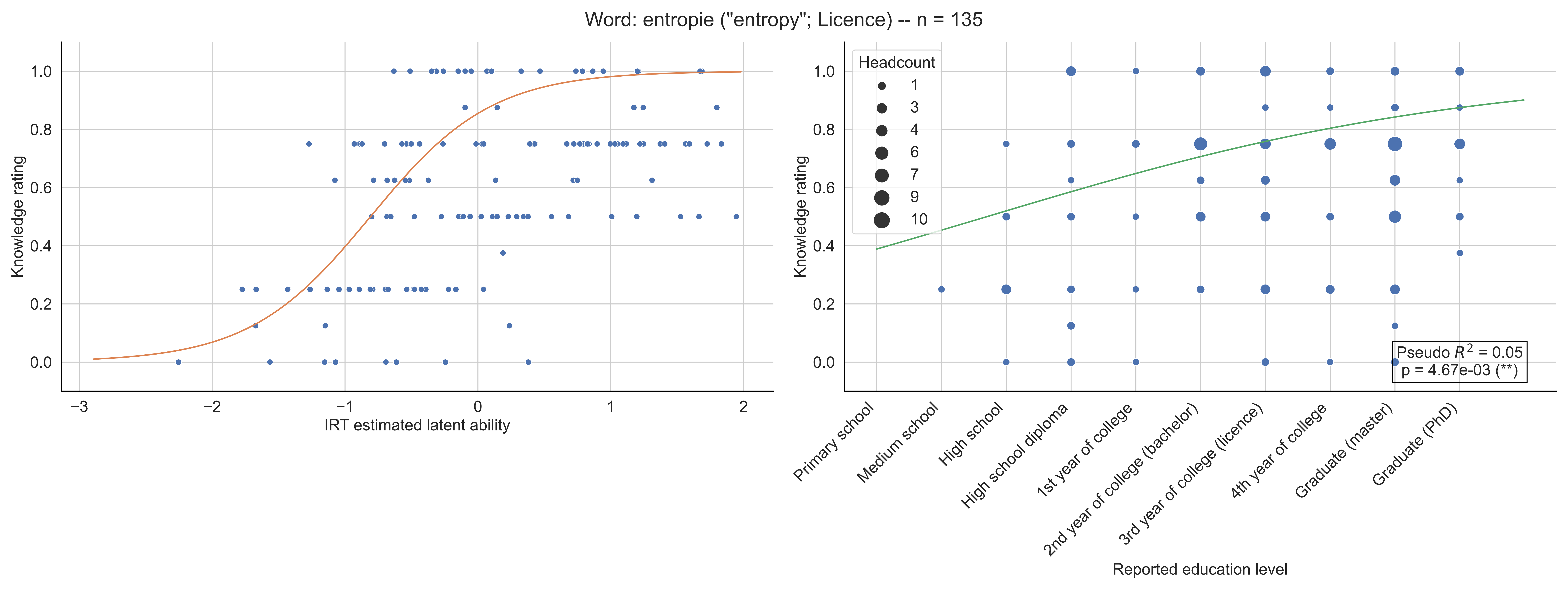
Word: entropie ("entropy"; Licence) -- n = 135
Knowledge rating
IRT estimated latent ability
Headcount
1
3
4
6
7
9
10
Pseudo R^2 = 0.05
p = 4.67e-03 (**)
Primary school
Medium school
High school
High school diploma
1st year of college
2nd year of college (bachelor)
3rd year of college (licence)
4th year of college
Graduate (master)
Graduate (PhD)
Reported education level

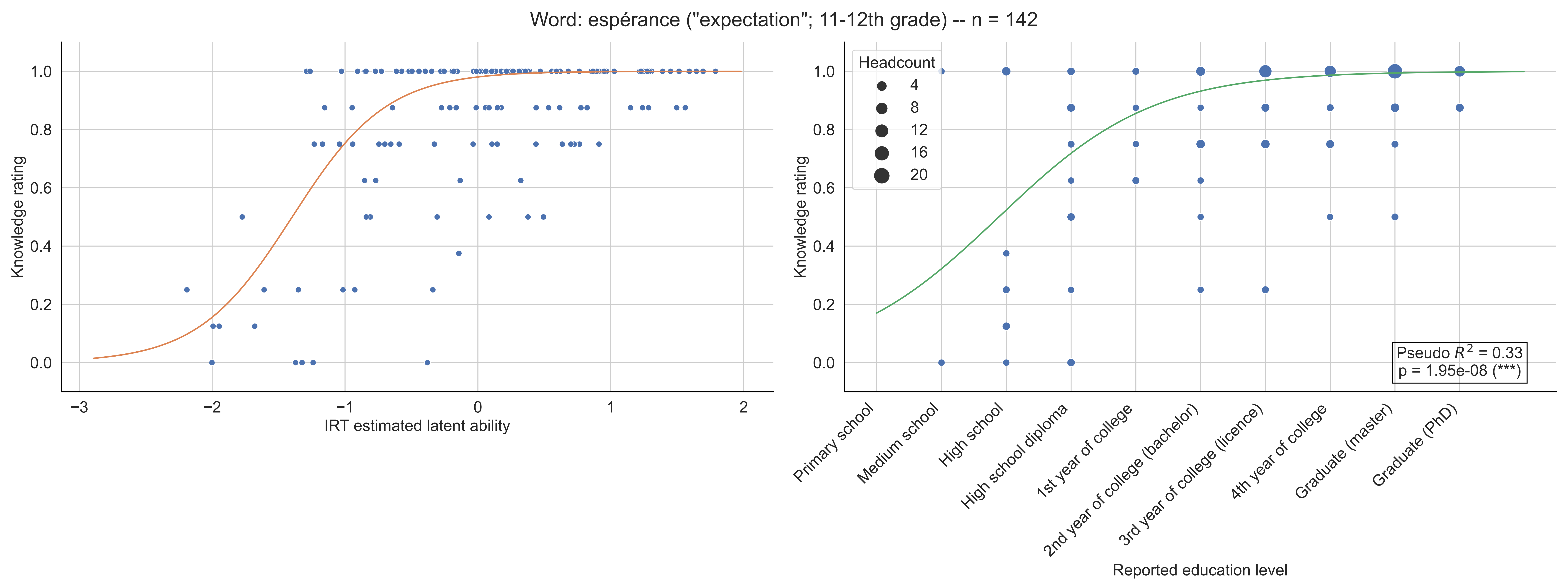

Word: espérance ("expectation"; 11-12th grade) -- n = 142
Knowledge rating
IRT estimated latent ability
Headcount
4
8
12
16
20
Pseudo R^2 = 0.33
p = 1.95e-08 (***)
Primary school
Medium school
High school
High school diploma
1st year of college
2nd year of college (bachelor)
3rd year of college (licence)
4th year of college
Graduate (master)
Graduate (PhD)
Reported education level

Word: estimateur ("estimator"; Licence) -- n = 157

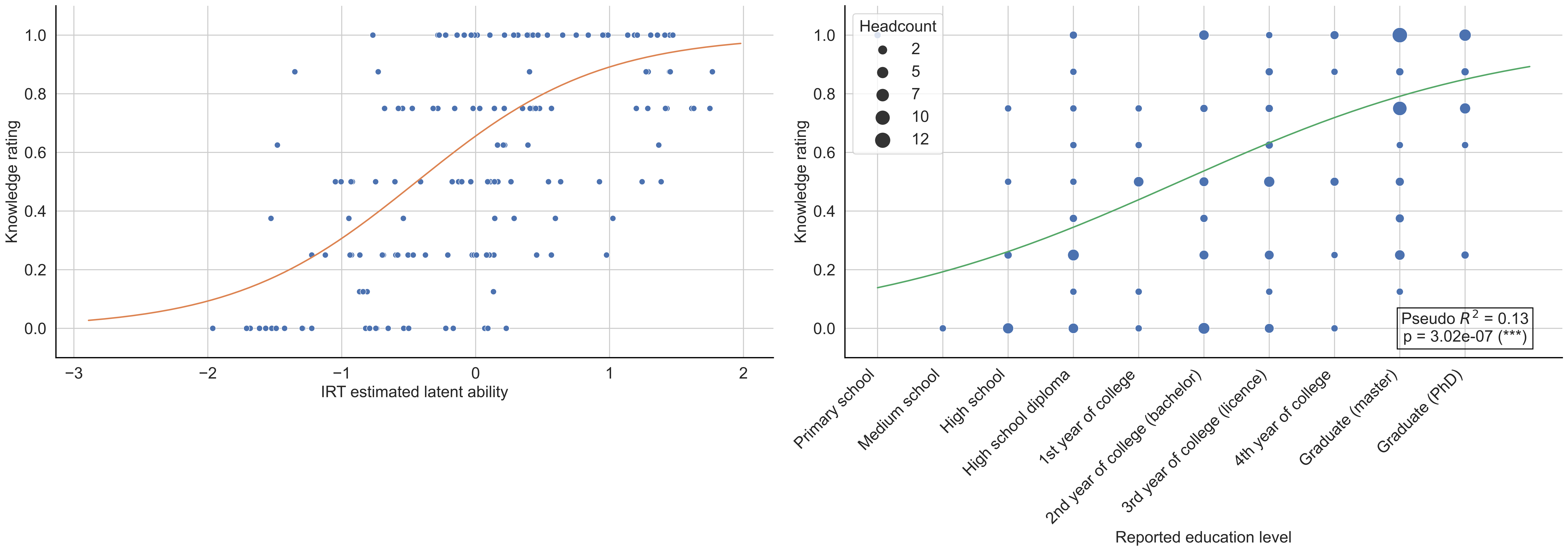

Word: excentricité ("excentricity"; Licence) -- n = 165

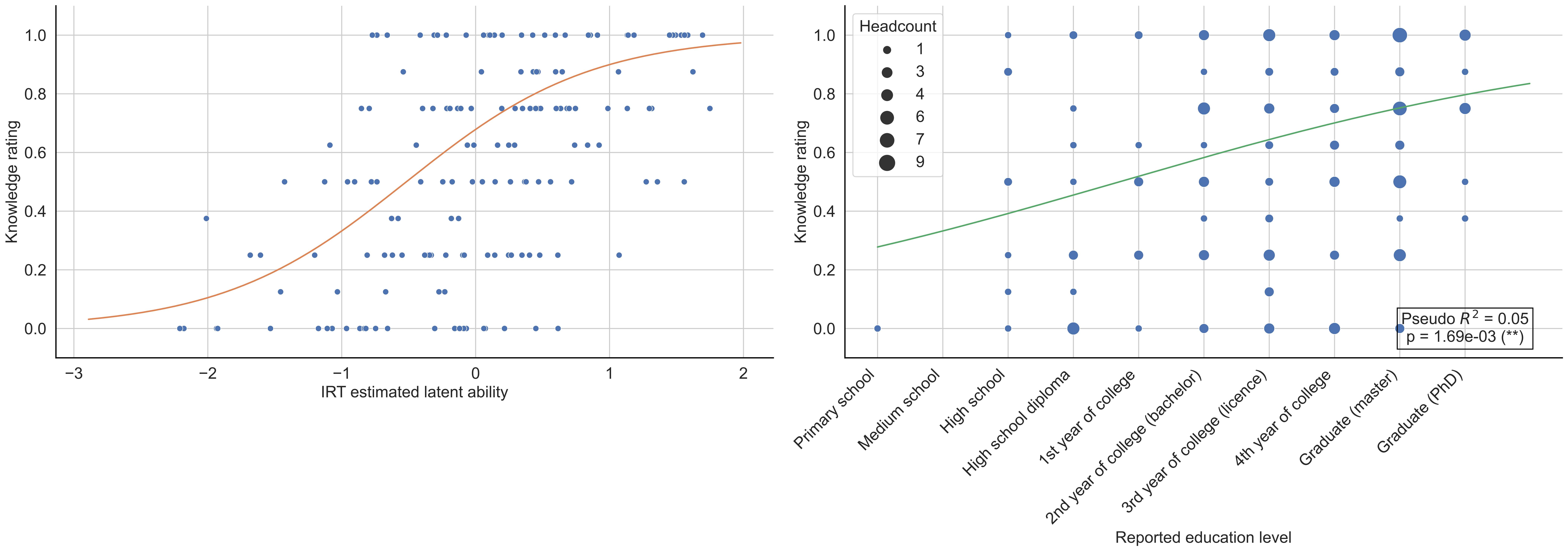

Word: exponentiation ("exponentiation"; 11-12th grade) -- n = 133

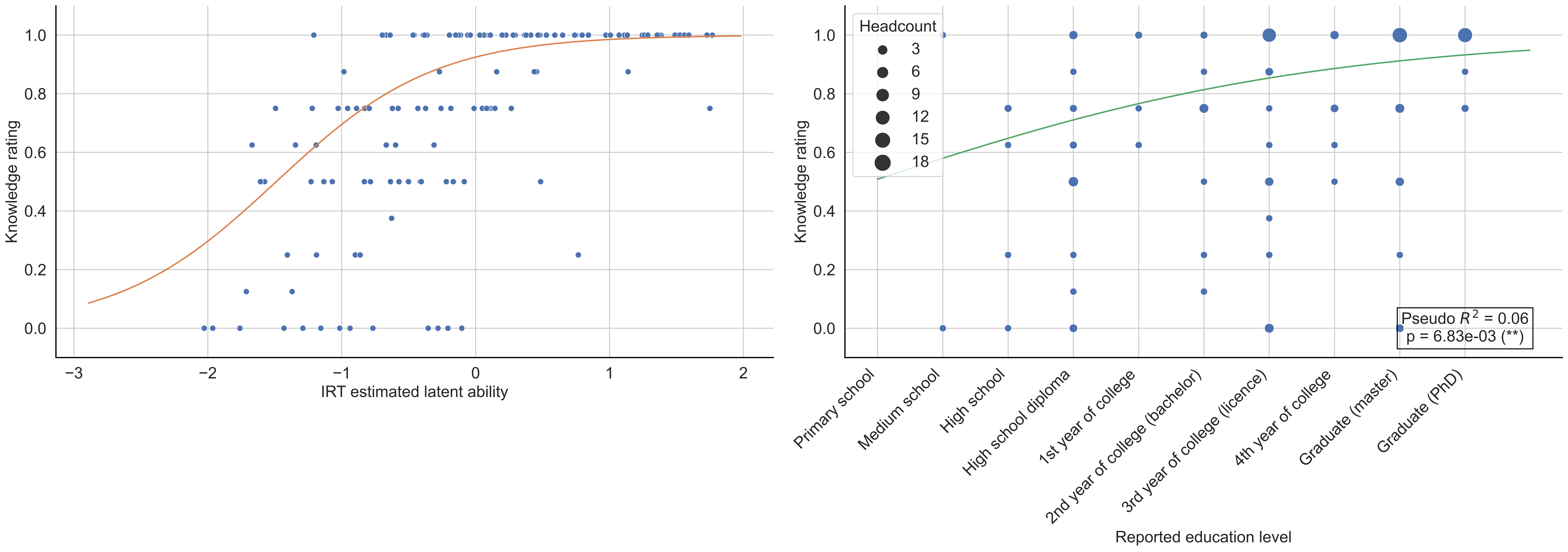

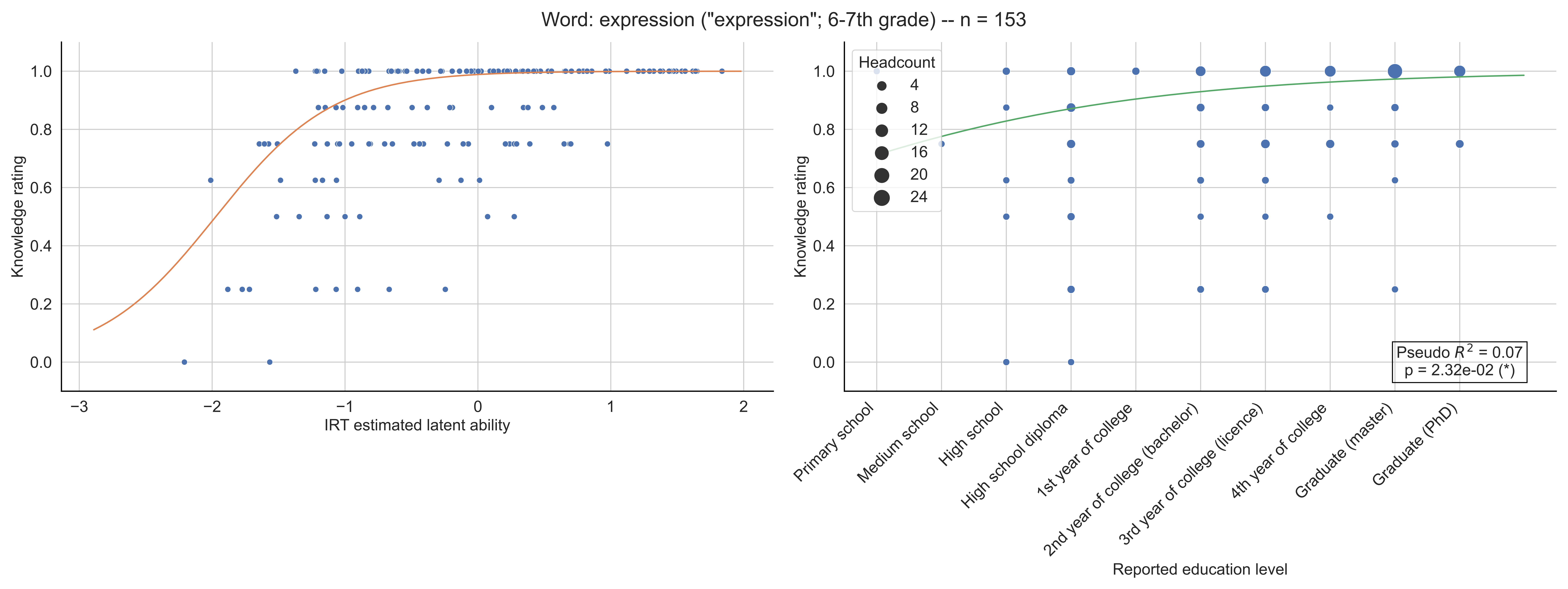

Word: expression ("expression"; 6-7th grade) -- n = 153
Knowledge rating
IRT estimated latent ability
Headcount
4
8
12
16
20
24
Pseudo R^2 = 0.07
p = 2.32e-02 (*)
Primary school
Medium school
High school
High school diploma
1st year of college
2nd year of college (bachelor)
3rd year of college (licence)
4th year of college
Graduate (master)
Graduate (PhD)
Reported education level

Word: extrapolation ("extrapolation"; Bachelor) -- n = 144

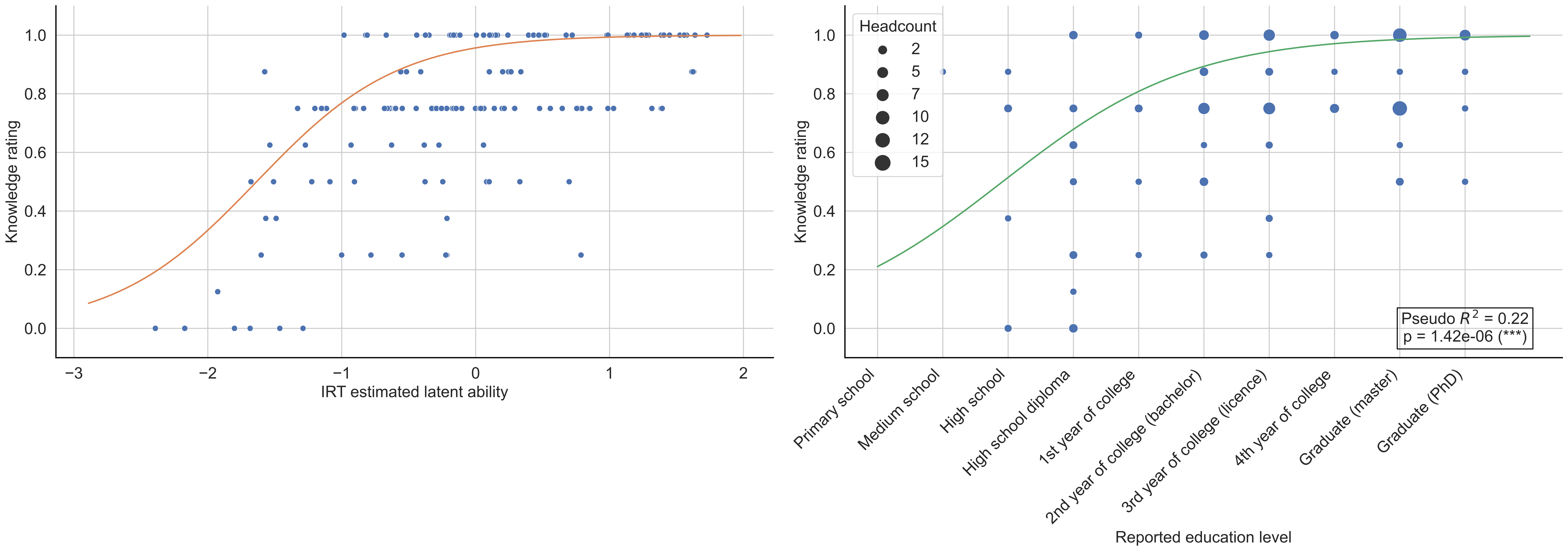

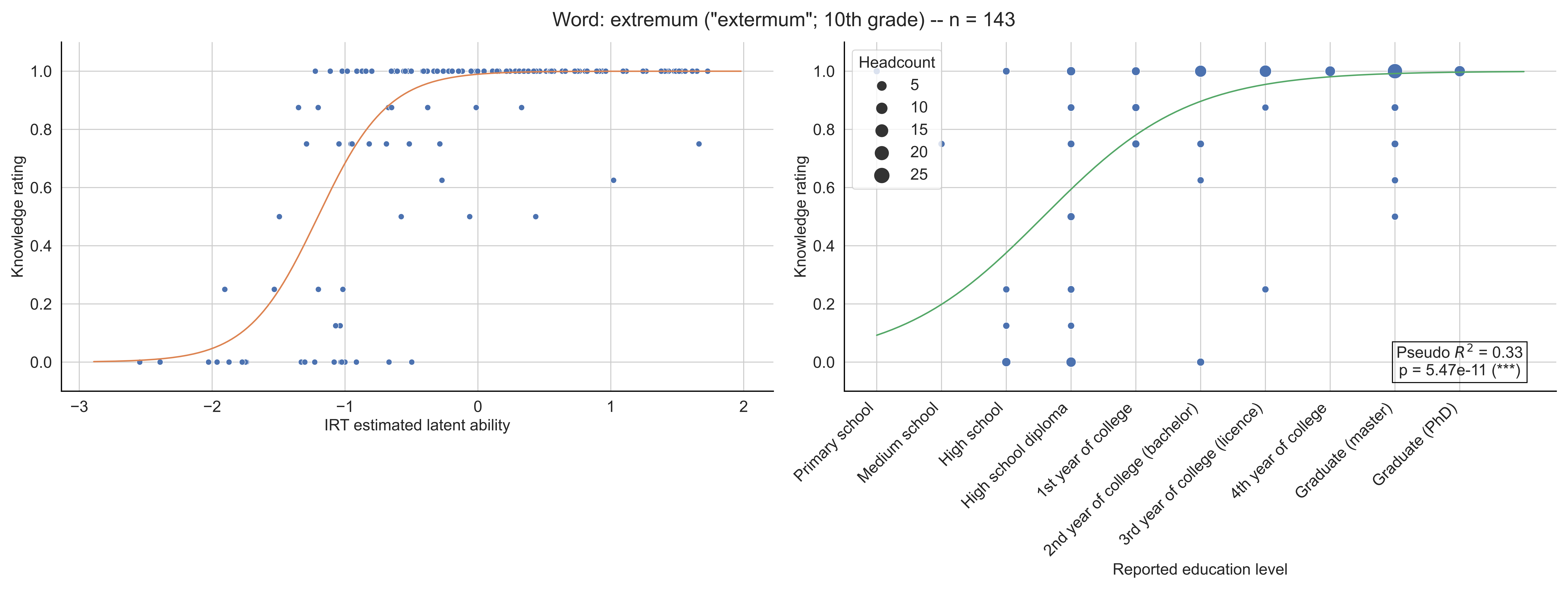

Word: extremum ("extermum"; 10th grade) -- n = 143
Knowledge rating
IRT estimated latent ability
Headcount
5
10
15
20
25
Pseudo R^2 = 0.33
p = 5.47e-11 (***)
Primary school
Medium school
High school
High school diploma
1st year of college
2nd year of college (bachelor)
3rd year of college (licence)
4th year of college
Graduate (master)
Graduate (PhD)
Reported education level

Word: extérieur ("exterior"; Primary school) -- n = 123

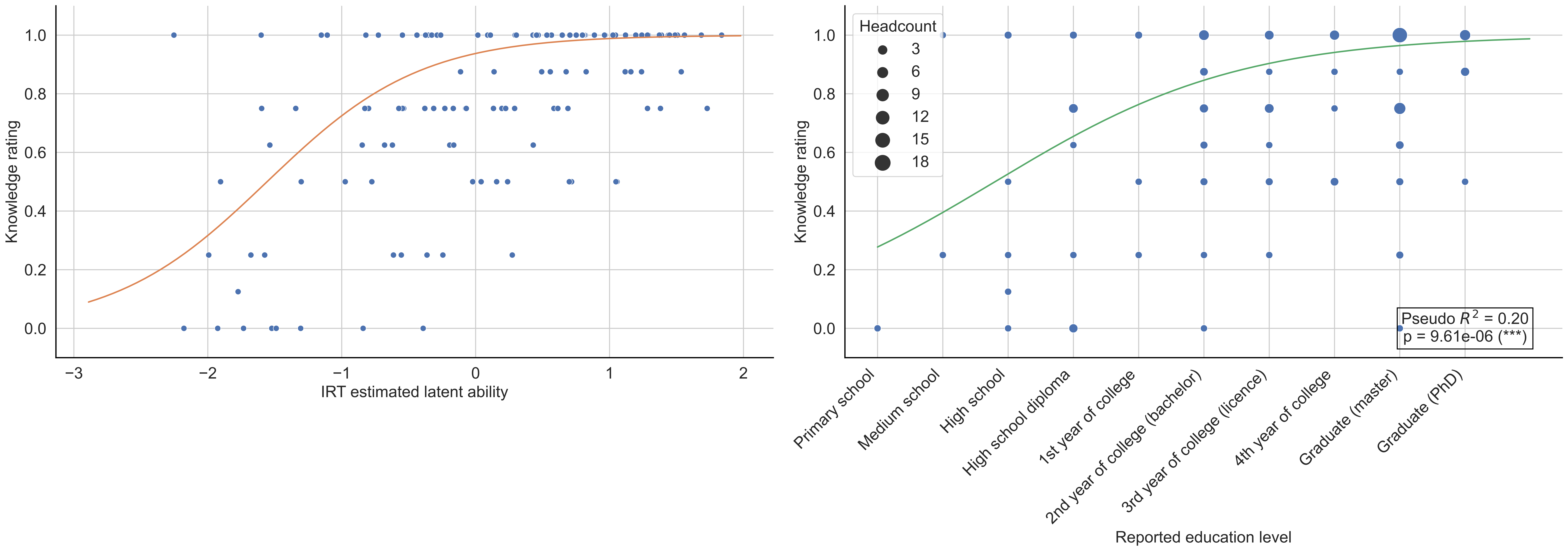

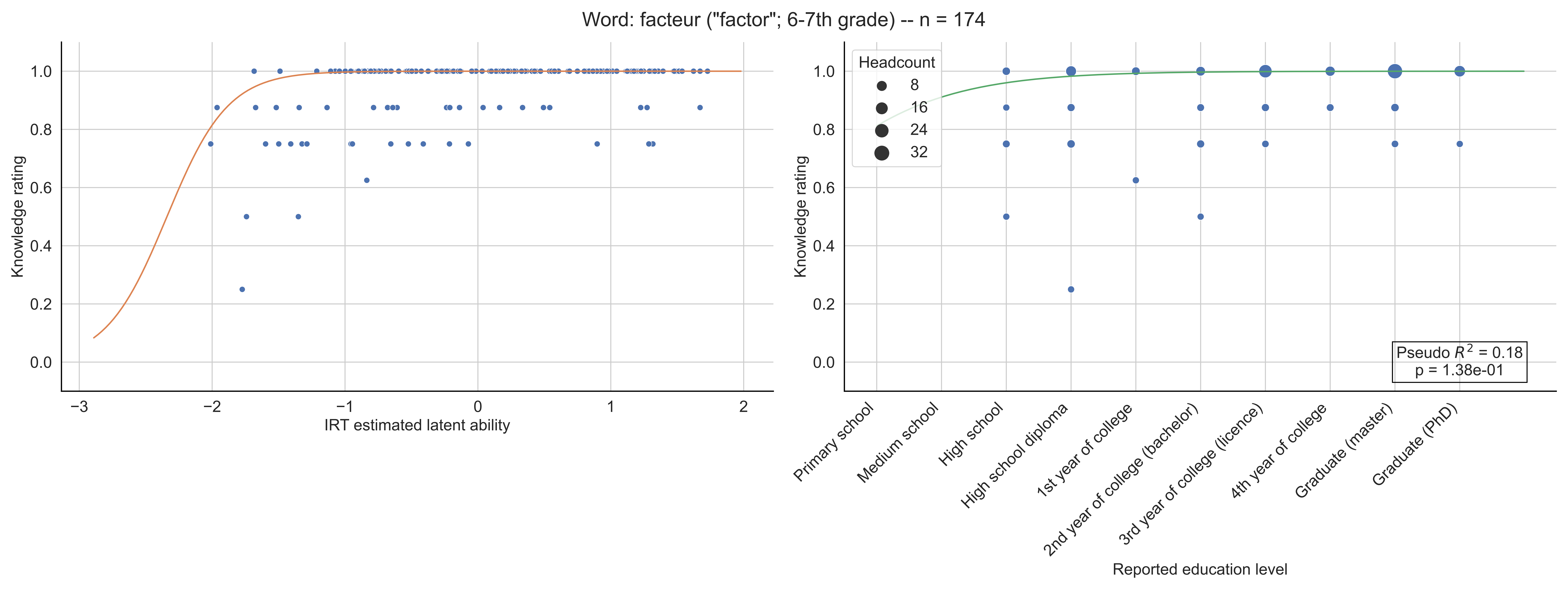
Word: facteur ("factor"; 6-7th grade) -- n = 174
Knowledge rating
IRT estimated latent ability
Headcount
8
16
24
32
Pseudo R^2 = 0.18
p = 1.38e-01
Primary school
Medium school
High school
High school diploma
1st year of college
2nd year of college (bachelor)
3rd year of college (licence)
4th year of college
Graduate (master)
Graduate (PhD)
Reported education level

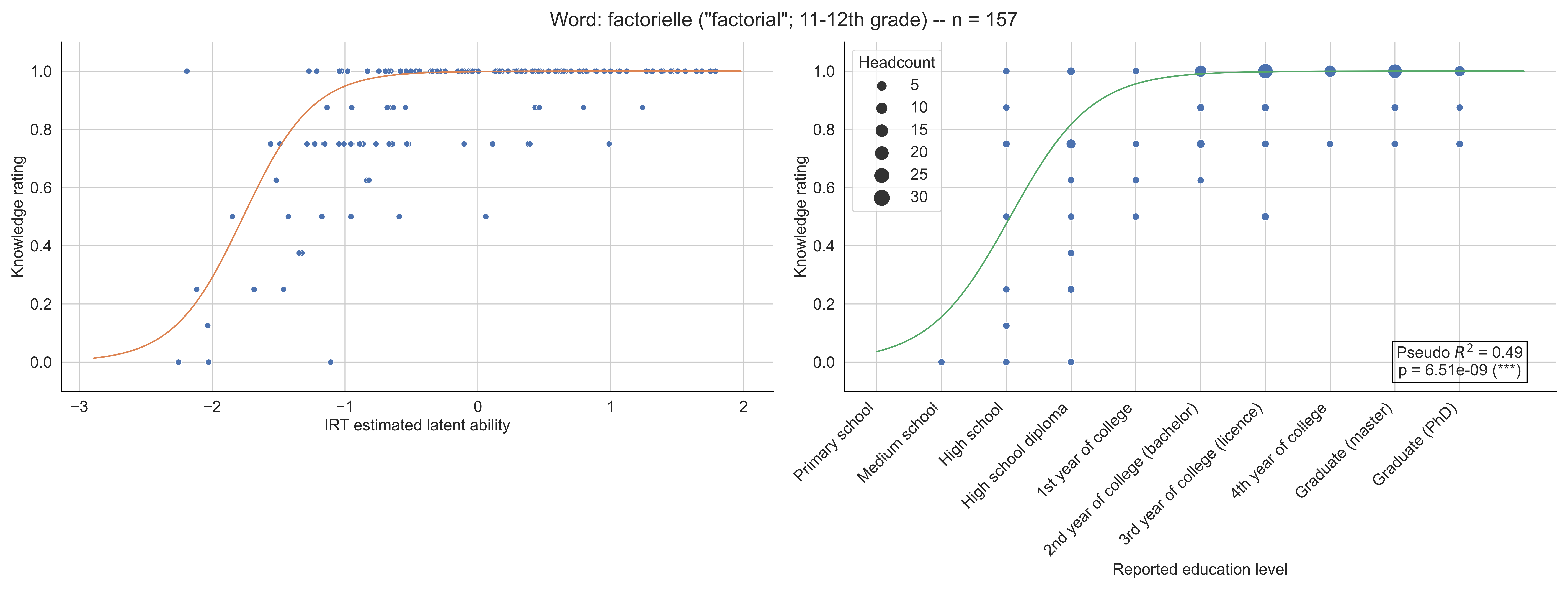
Word: factorielle ("factorial"; 11-12th grade) -- n = 157
Knowledge rating
IRT estimated latent ability
Headcount
5
10
15
20
25
30
Pseudo R^2 = 0.49
p = 6.51e-09 (***)
Primary school
Medium school
High school
High school diploma
1st year of college
2nd year of college (bachelor)
3rd year of college (licence)
4th year of college
Graduate (master)
Graduate (PhD)
Reported education level

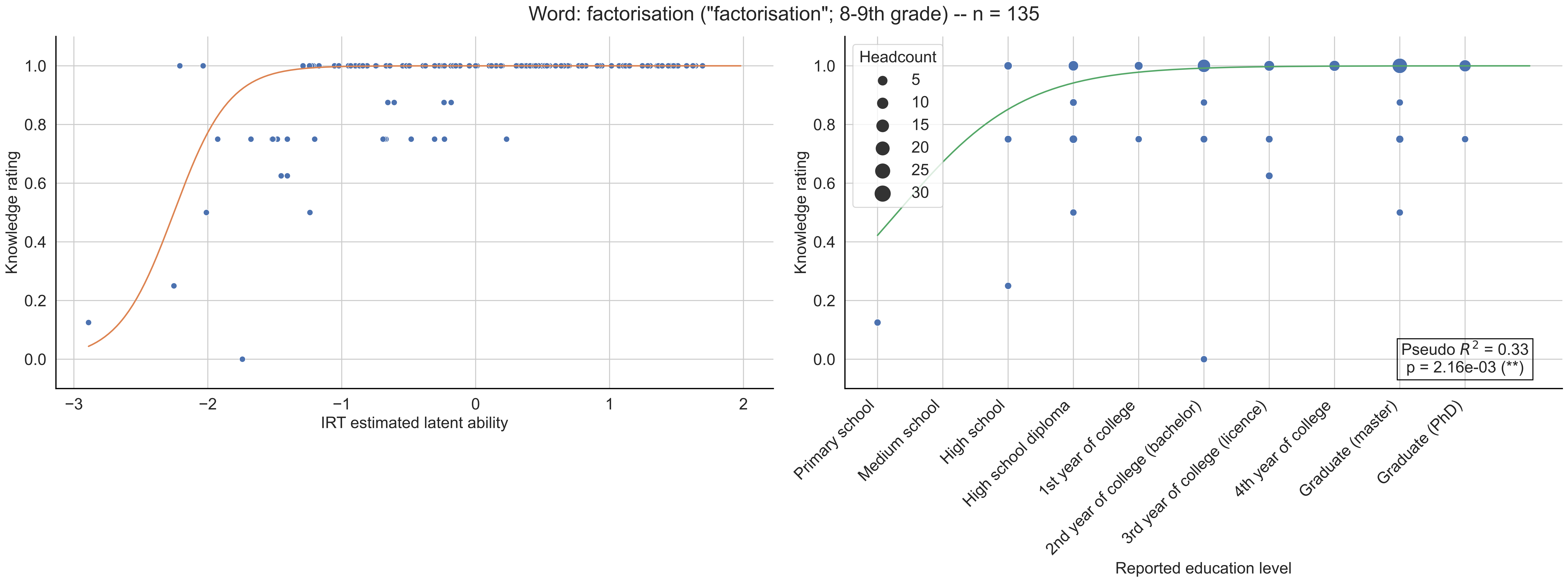

Word: factorisation ("factorisation"; 8-9th grade) -- n = 135
Knowledge rating
IRT estimated latent ability
−3
−2
−1
0
1
2
0.0
0.2
0.4
0.6
0.8
1.0
Headcount
5
10
15
20
25
30
Pseudo R^2 = 0.33
p = 2.16e-03 (**)
Primary school
Medium school
High school
High school diploma
1st year of college
2nd year of college (bachelor)
3rd year of college (licence)
4th year of college
Graduate (master)
Graduate (PhD)
Reported education level

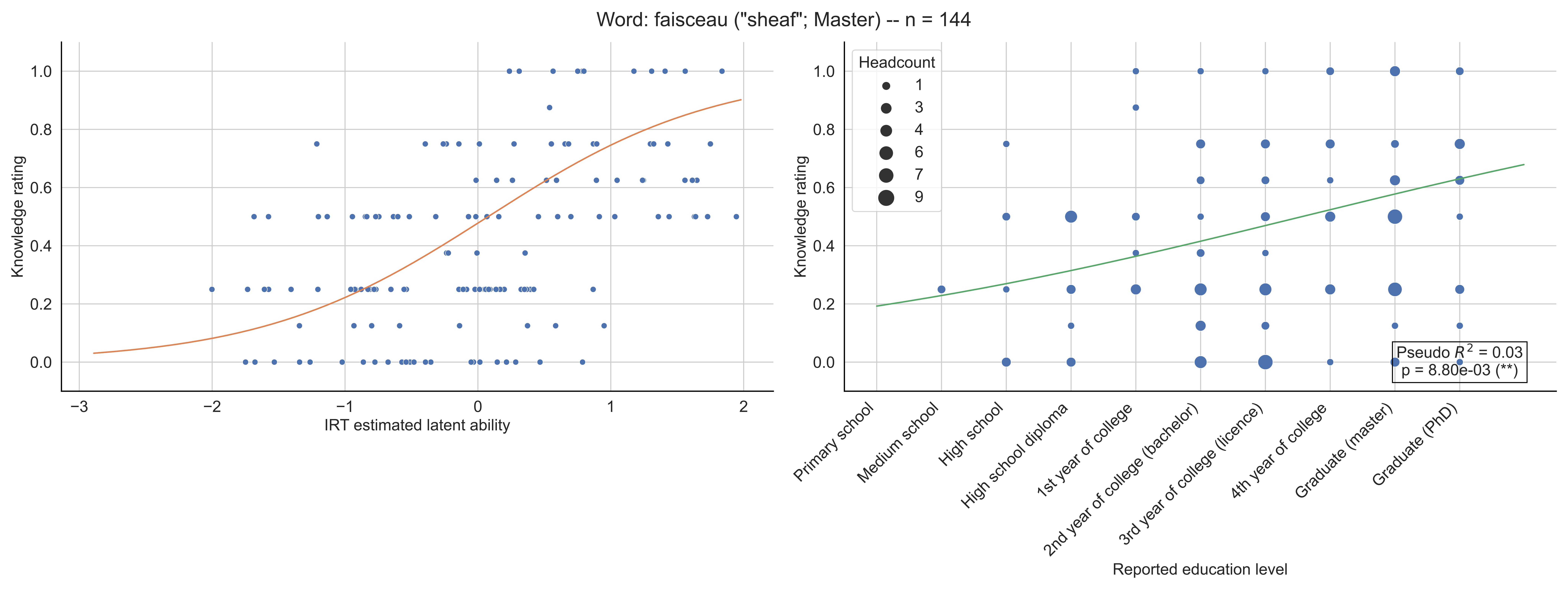

Word: faisceau ("sheaf"; Master) -- n = 144
Knowledge rating
IRT estimated latent ability
Headcount
1
3
4
6
7
9
Pseudo R^2 = 0.03
p = 8.80e-03 (**)
Primary school
Medium school
High school
High school diploma
1st year of college
2nd year of college (bachelor)
3rd year of college (licence)
4th year of college
Graduate (master)
Graduate (PhD)
Reported education level

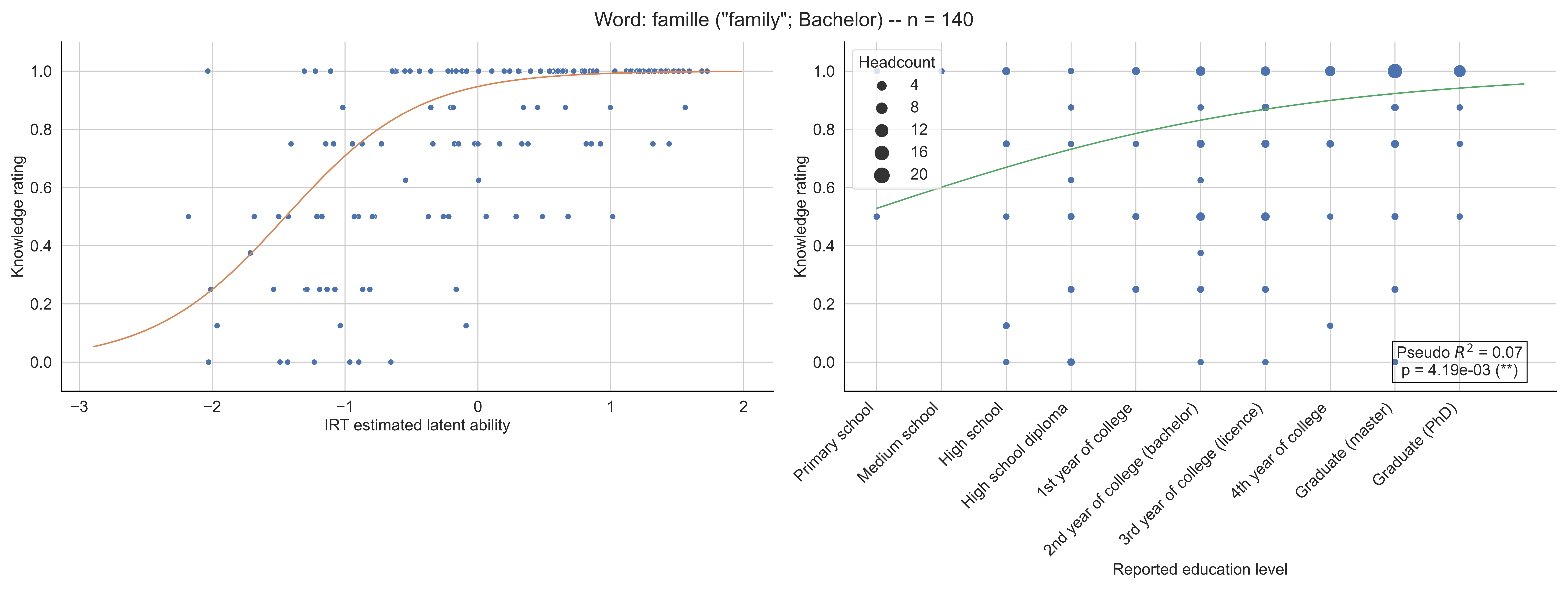
Word: famille ("family"; Bachelor) -- n = 140
Knowledge rating
IRT estimated latent ability
Headcount
4
8
12
16
20
Pseudo R^2 = 0.07
p = 4.19e-03 (**)
Primary school
Medium school
High school
High school diploma
1st year of college
2nd year of college (bachelor)
3rd year of college (licence)
4th year of college
Graduate (master)
Graduate (PhD)
Reported education level

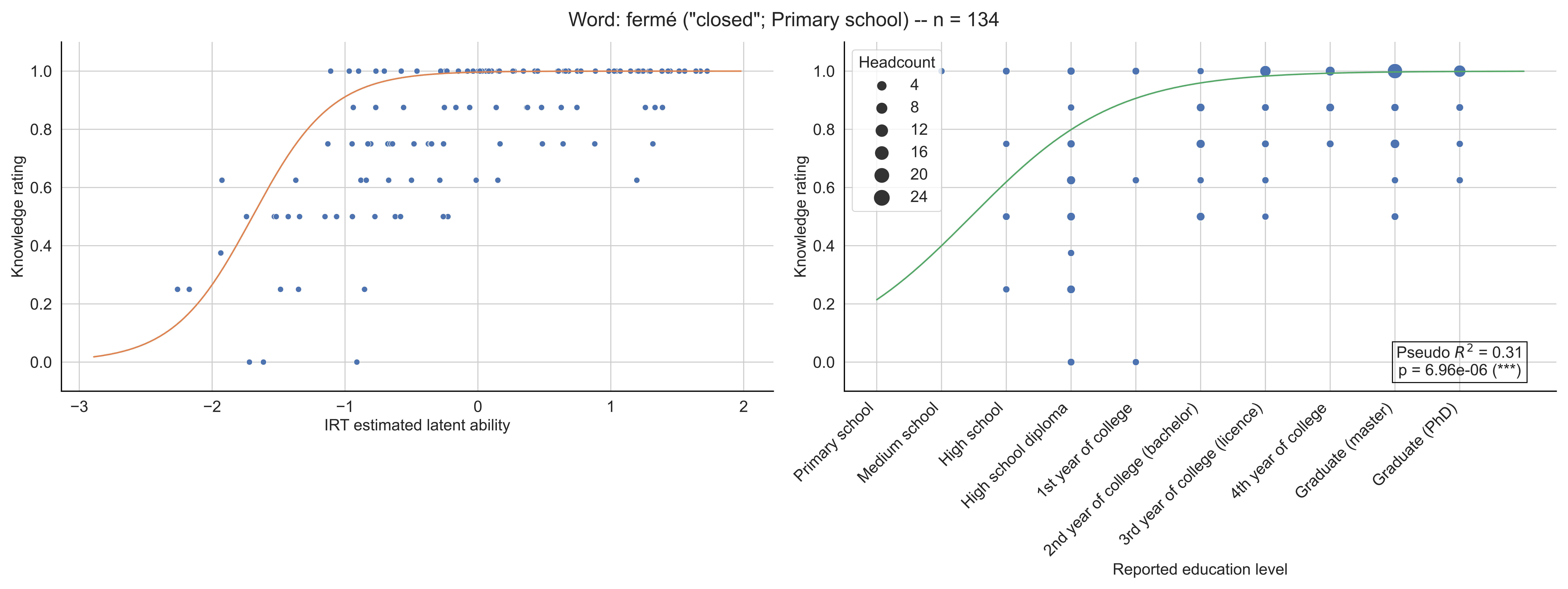

Word: fermé ("closed"; Primary school) -- n = 134
Knowledge rating
IRT estimated latent ability
Headcount
4
8
12
16
20
24
Pseudo R^2 = 0.31
p = 6.96e-06 (***)
Primary school
Medium school
High school
High school diploma
1st year of college
2nd year of college (bachelor)
3rd year of college (licence)
4th year of college
Graduate (master)
Graduate (PhD)
Reported education level

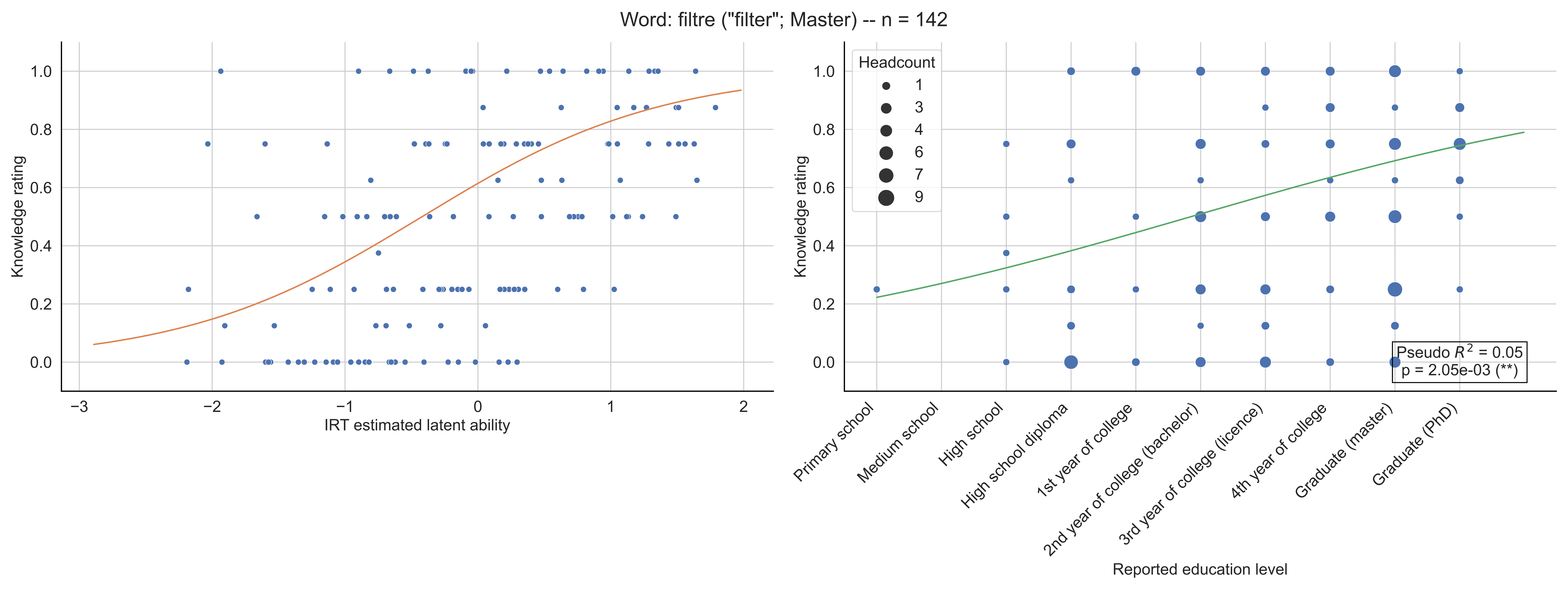

Word: filtre ("filter"; Master) -- n = 142
Knowledge rating
IRT estimated latent ability
Headcount
1
3
4
6
7
9
Pseudo R^2 = 0.05
p = 2.05e-03 (**)
Primary school
Medium school
High school
High school diploma
1st year of college
2nd year of college (bachelor)
3rd year of college (licence)
4th year of college
Graduate (master)
Graduate (PhD)
Reported education level

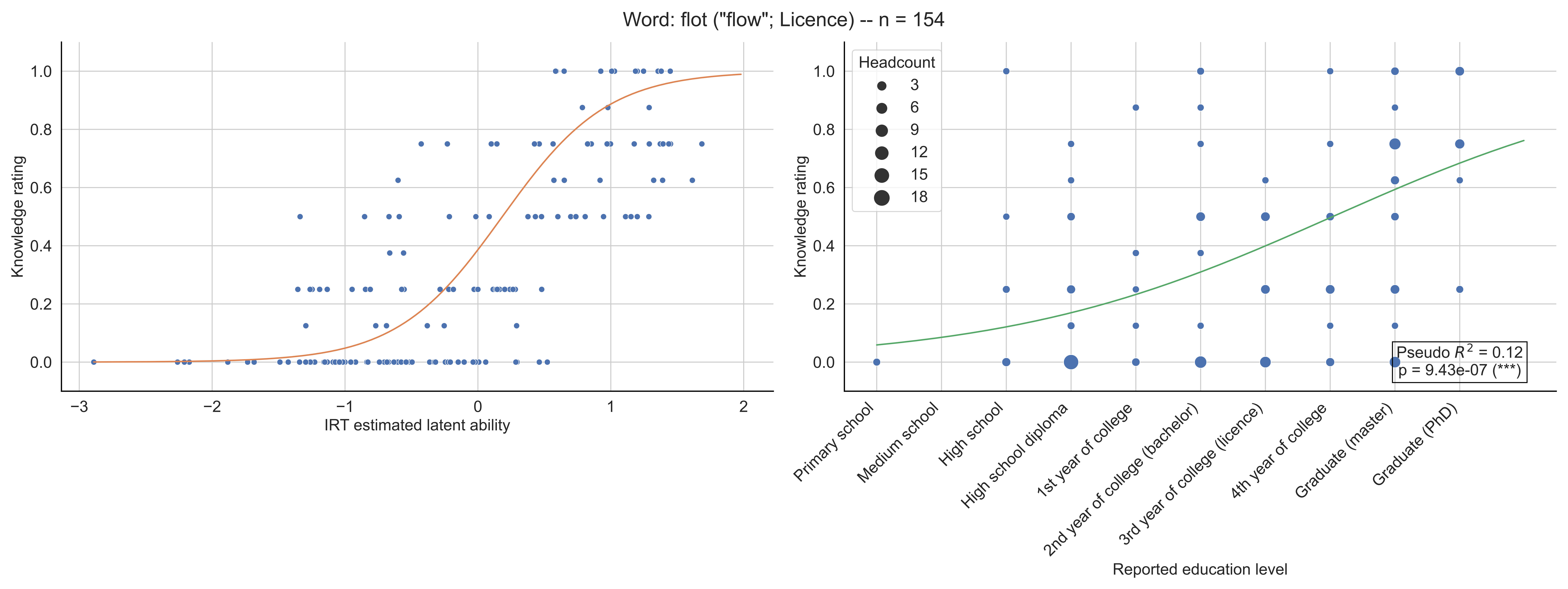

Word: flot ("flow"; Licence) -- n = 154
Knowledge rating
IRT estimated latent ability
Headcount
3
6
9
12
15
18
Pseudo R^2 = 0.12
p = 9.43e-07 (***)
Primary school
Medium school
High school
High school diploma
1st year of college
2nd year of college (bachelor)
3rd year of college (licence)
4th year of college
Graduate (master)
Graduate (PhD)
Reported education level

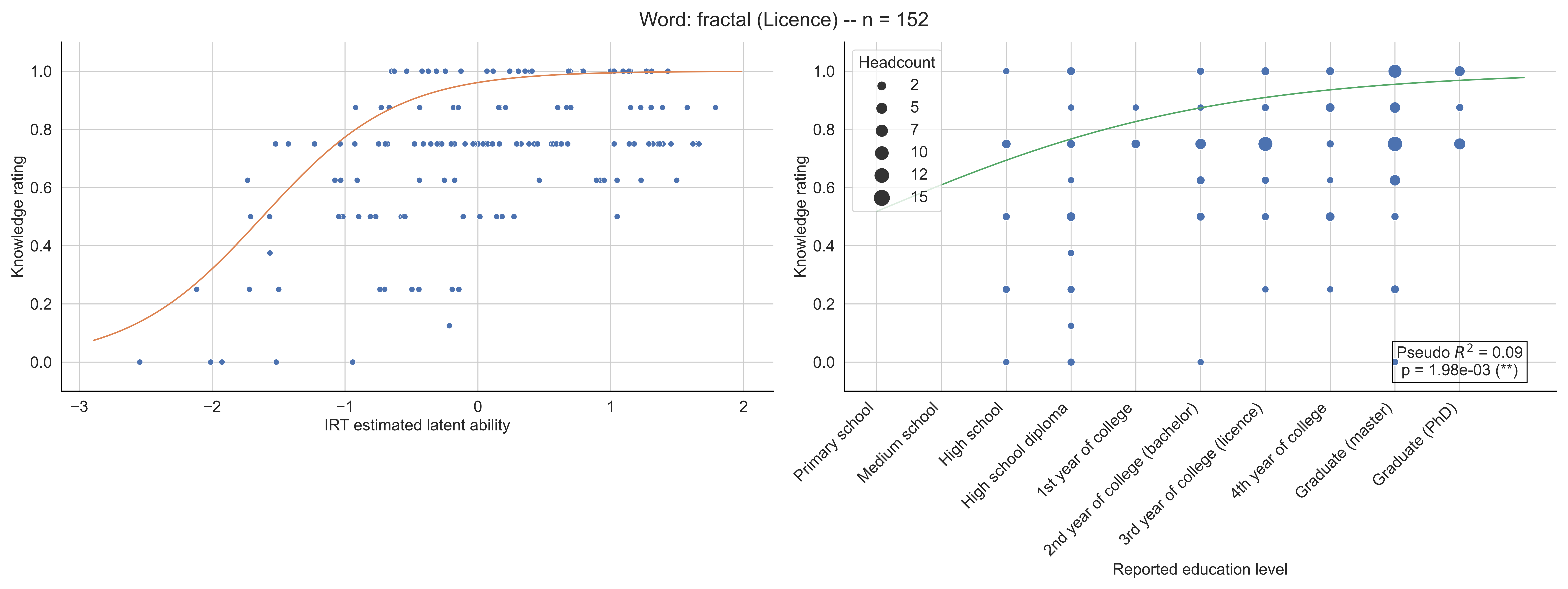

Word: fractal (Licence) -- n = 152
Knowledge rating
IRT estimated latent ability
Headcount
2
5
7
10
12
15
Pseudo R^2 = 0.09
p = 1.98e-03 (**)
Primary school
Medium school
High school
High school diploma
1st year of college
2nd year of college (bachelor)
3rd year of college (licence)
4th year of college
Graduate (master)
Graduate (PhD)
Reported education level

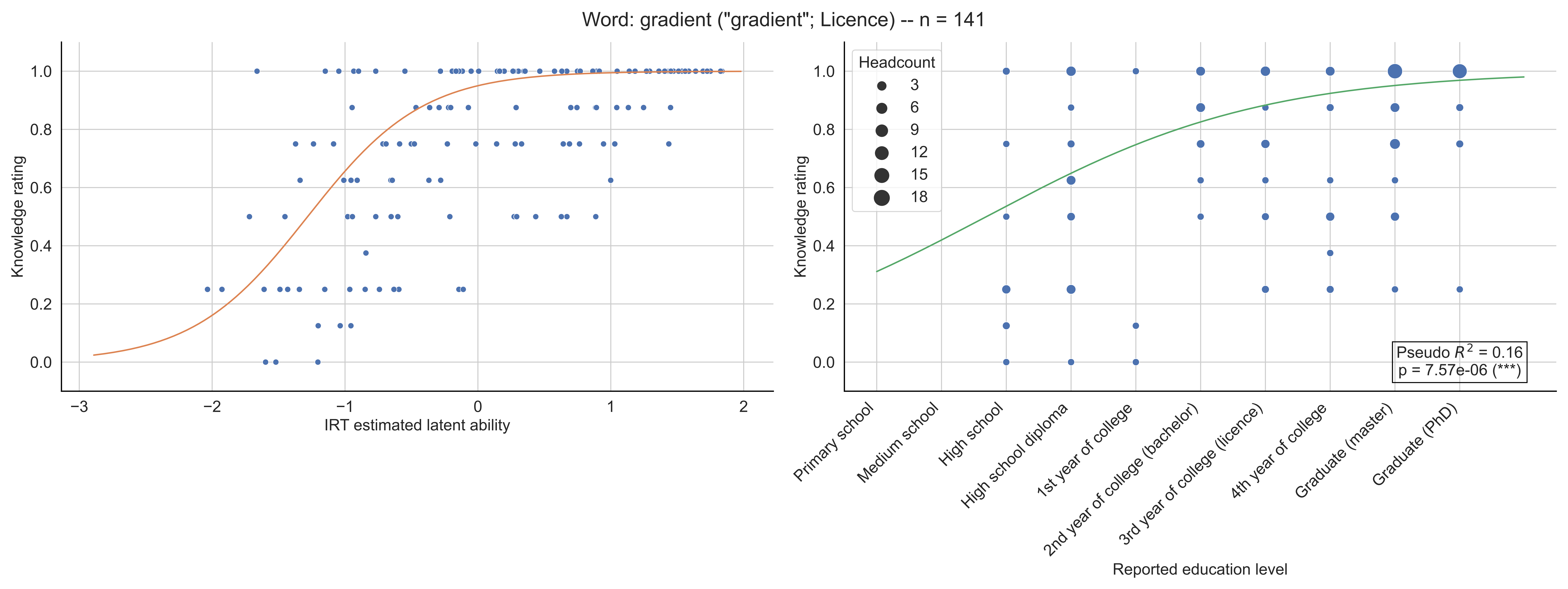

Word: gradient ("gradient"; Licence) -- n = 141
Knowledge rating
IRT estimated latent ability
Headcount
3
6
9
12
15
18
Pseudo R^2 = 0.16
p = 7.57e-06 (***)
Primary school
Medium school
High school
High school diploma
1st year of college
2nd year of college (bachelor)
3rd year of college (licence)
4th year of college
Graduate (master)
Graduate (PhD)
Reported education level

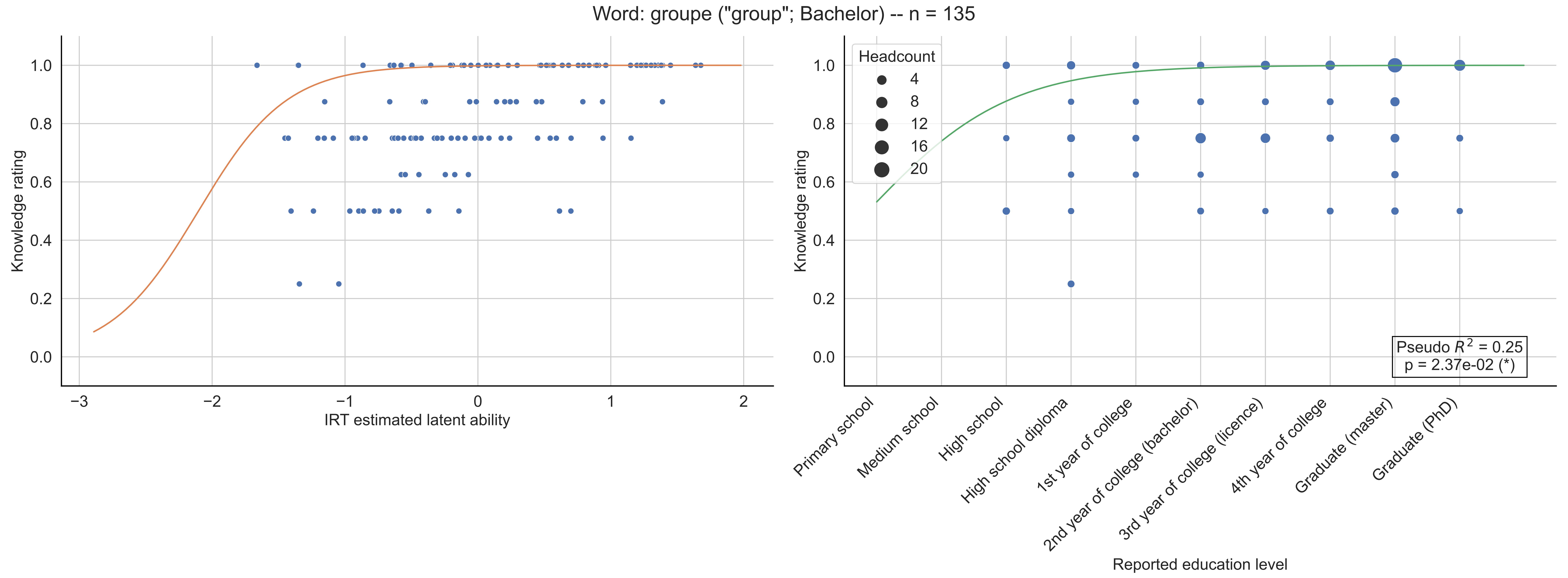

Word: groupe ("group"; Bachelor) -- n = 135
Knowledge rating
1.0
0.8
0.6
0.4
0.2
0.0
−3
−2
−1
0
1
2
IRT estimated latent ability
Headcount
4
8
12
16
20
Pseudo R^2 = 0.25
p = 2.37e-02 (*)
Primary school
Medium school
High school
High school diploma
1st year of college
2nd year of college (bachelor)
3rd year of college (licence)
4th year of college
Graduate (master)
Graduate (PhD)
Reported education level

Word: génératrice ("generating"; Licence) -- n = 147

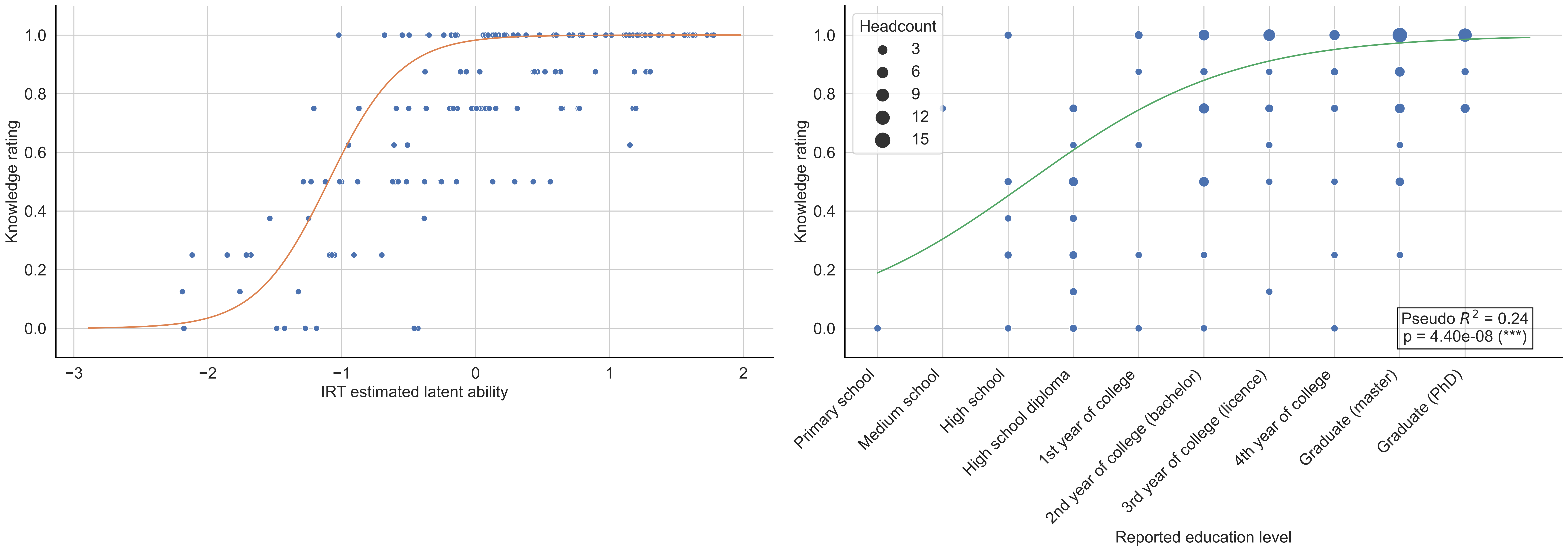

Word: hamiltonien ("hamiltonian"; Licence) -- n = 151

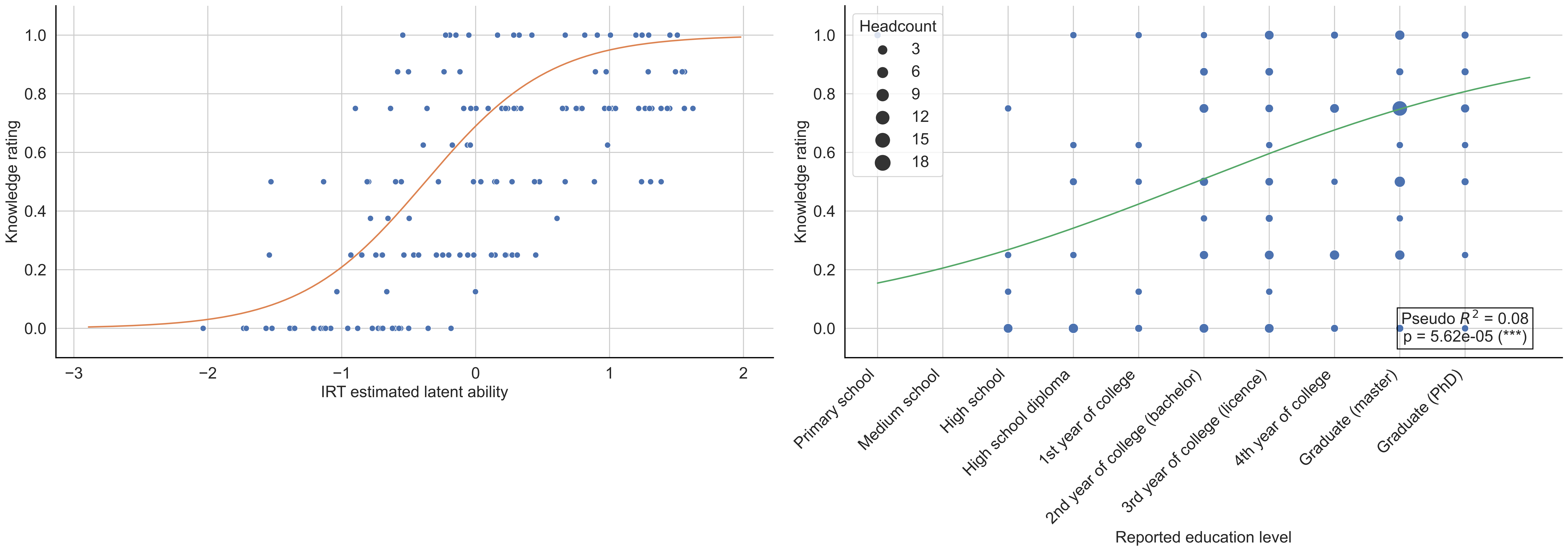

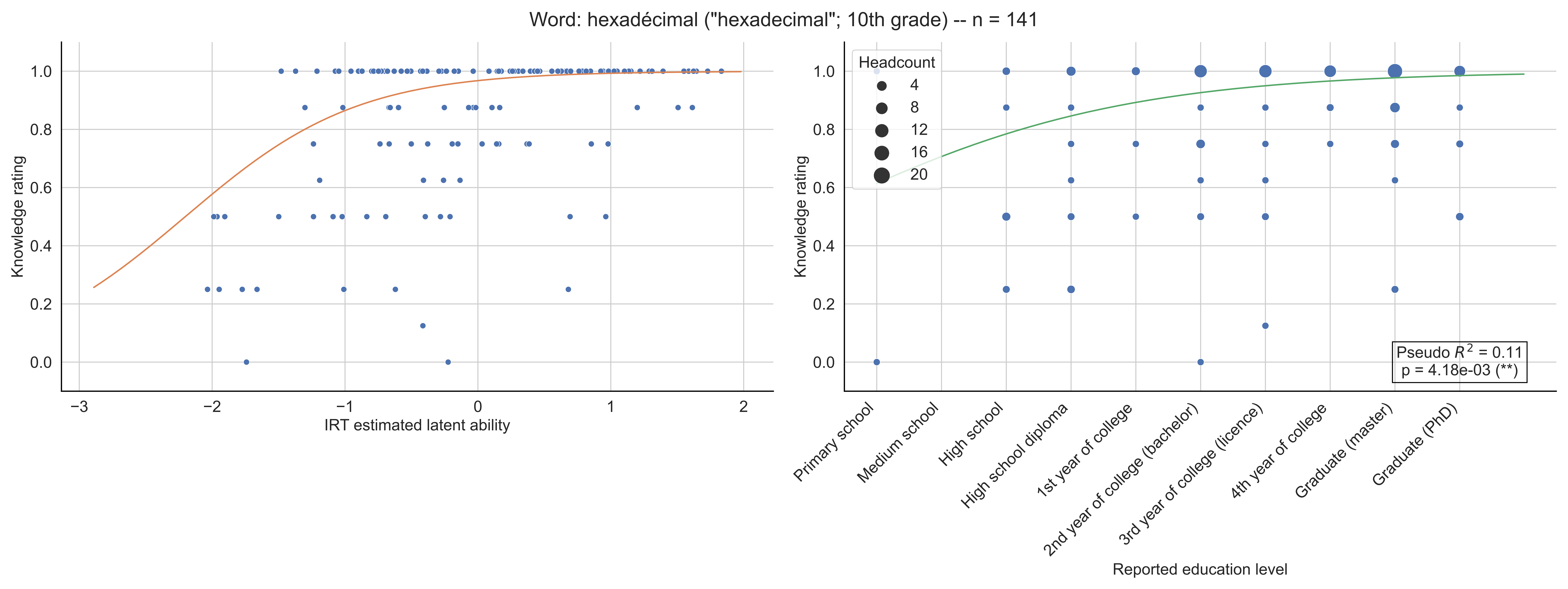

Word: hexadécimal ("hexadecimal"; 10th grade) -- n = 141
Knowledge rating
IRT estimated latent ability
Headcount
4
8
12
16
20
Pseudo R^2 = 0.11
p = 4.18e-03 (**)
Primary school
Medium school
High school
High school diploma
1st year of college
2nd year of college (bachelor)
3rd year of college (licence)
4th year of college
Graduate (master)
Graduate (PhD)
Reported education level

Word: histogramme ("histogram"; 8-9th grade) -- n = 140

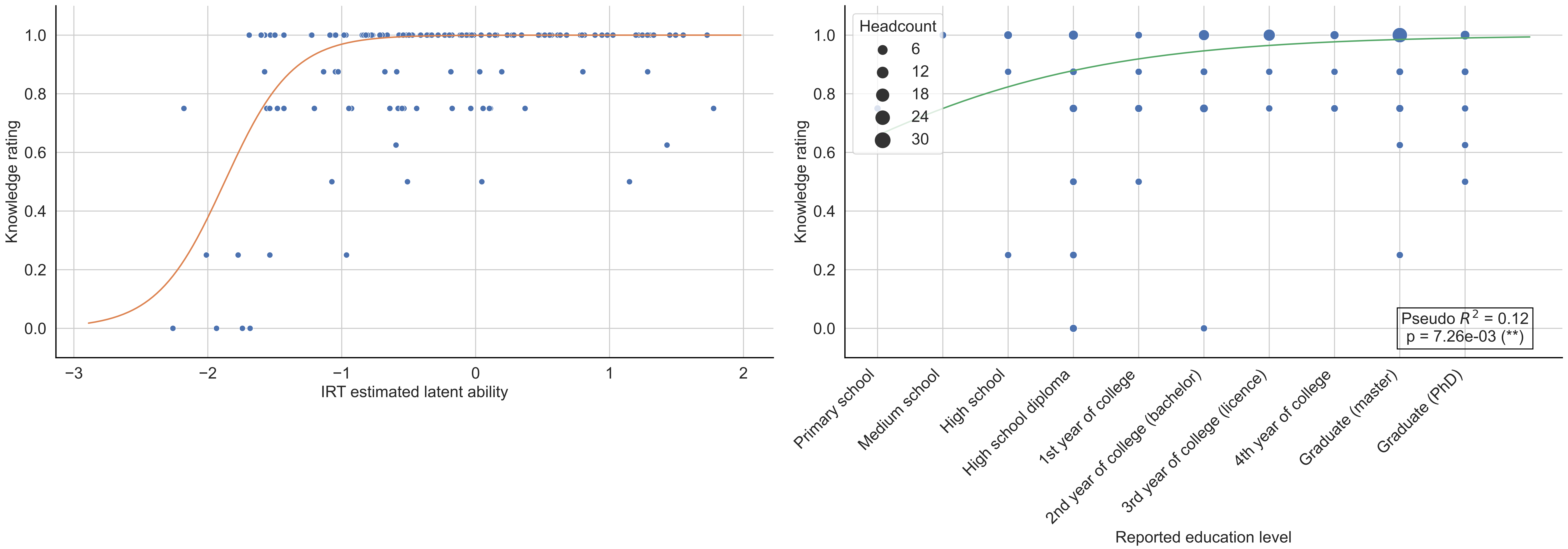

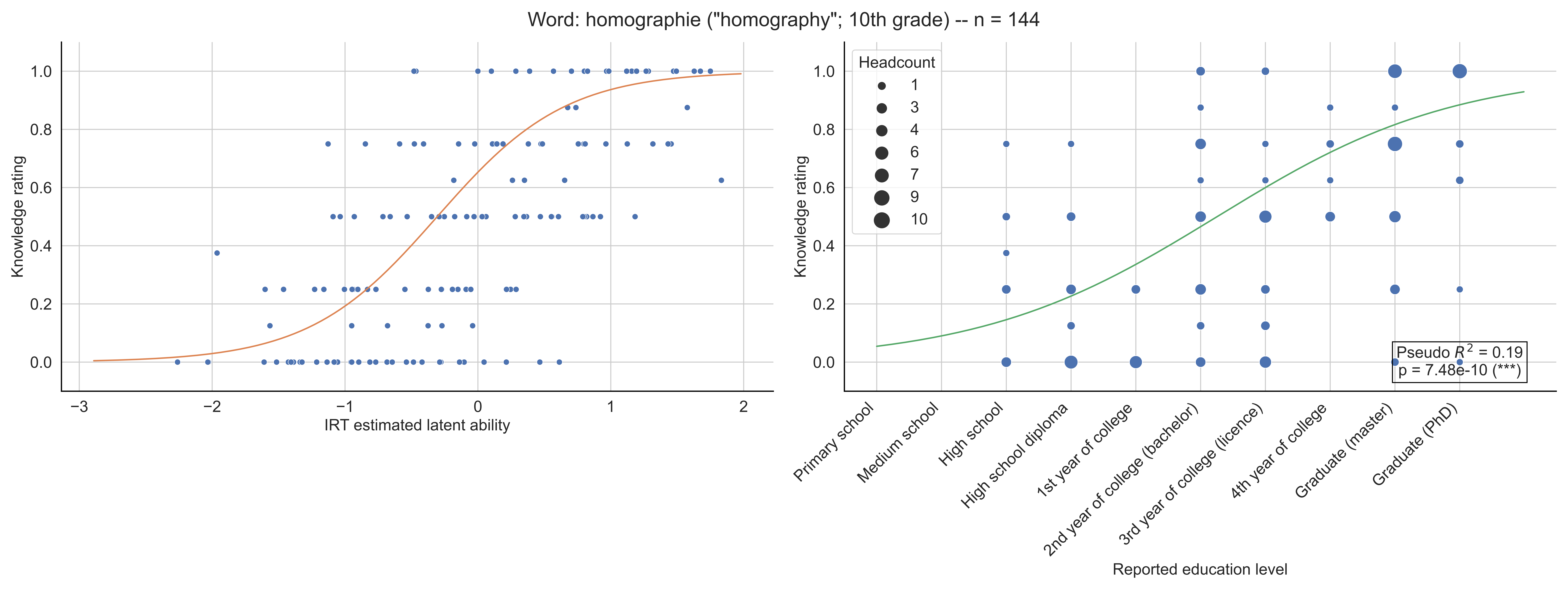

Word: homographie ("homography"; 10th grade) -- n = 144
Knowledge rating
IRT estimated latent ability
Headcount
1
3
4
6
7
9
10
Pseudo R^2 = 0.19
p = 7.48e-10 (***)
Primary school
Medium school
High school
High school diploma
1st year of college
2nd year of college (bachelor)
3rd year of college (licence)
4th year of college
Graduate (master)
Graduate (PhD)
Reported education level

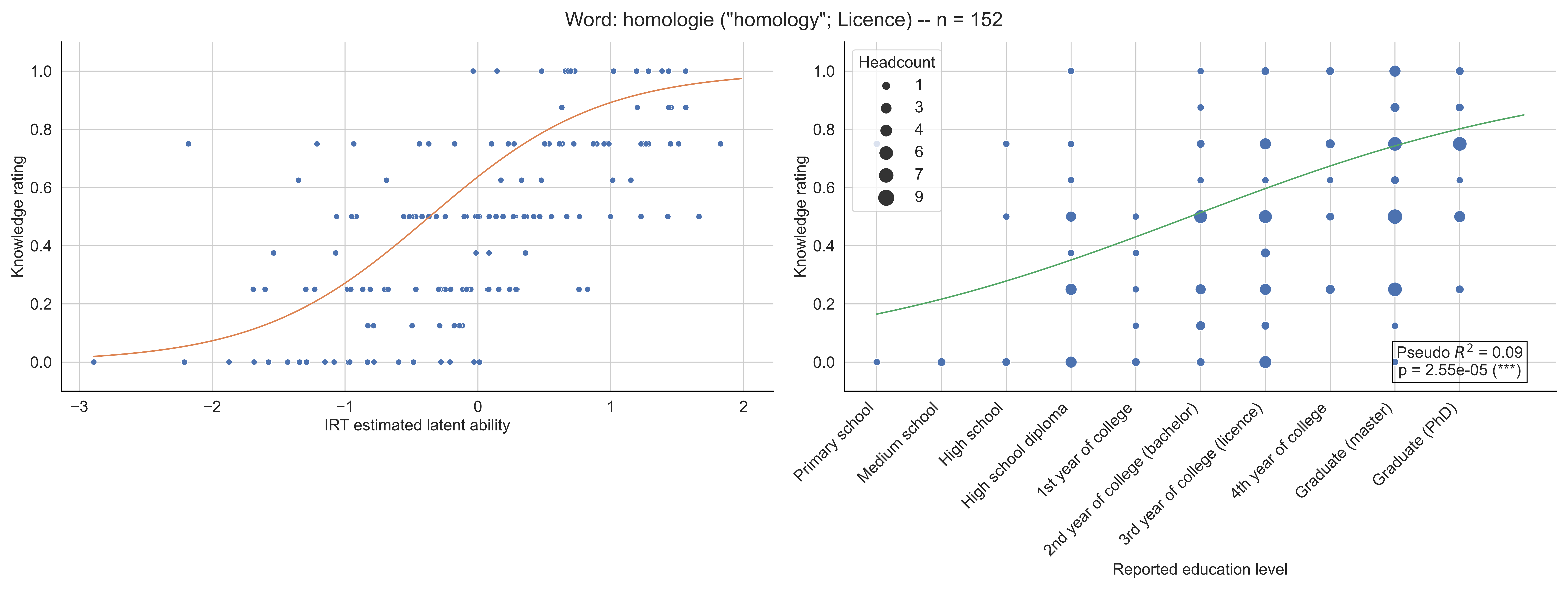
Word: homologie ("homology"; Licence) -- n = 152
Knowledge rating
IRT estimated latent ability
Headcount
1
3
4
6
7
9
Pseudo R^2 = 0.09
p = 2.55e-05 (***)
Primary school
Medium school
High school
High school diploma
1st year of college
2nd year of college (bachelor)
3rd year of college (licence)
4th year of college
Graduate (master)
Graduate (PhD)
Reported education level

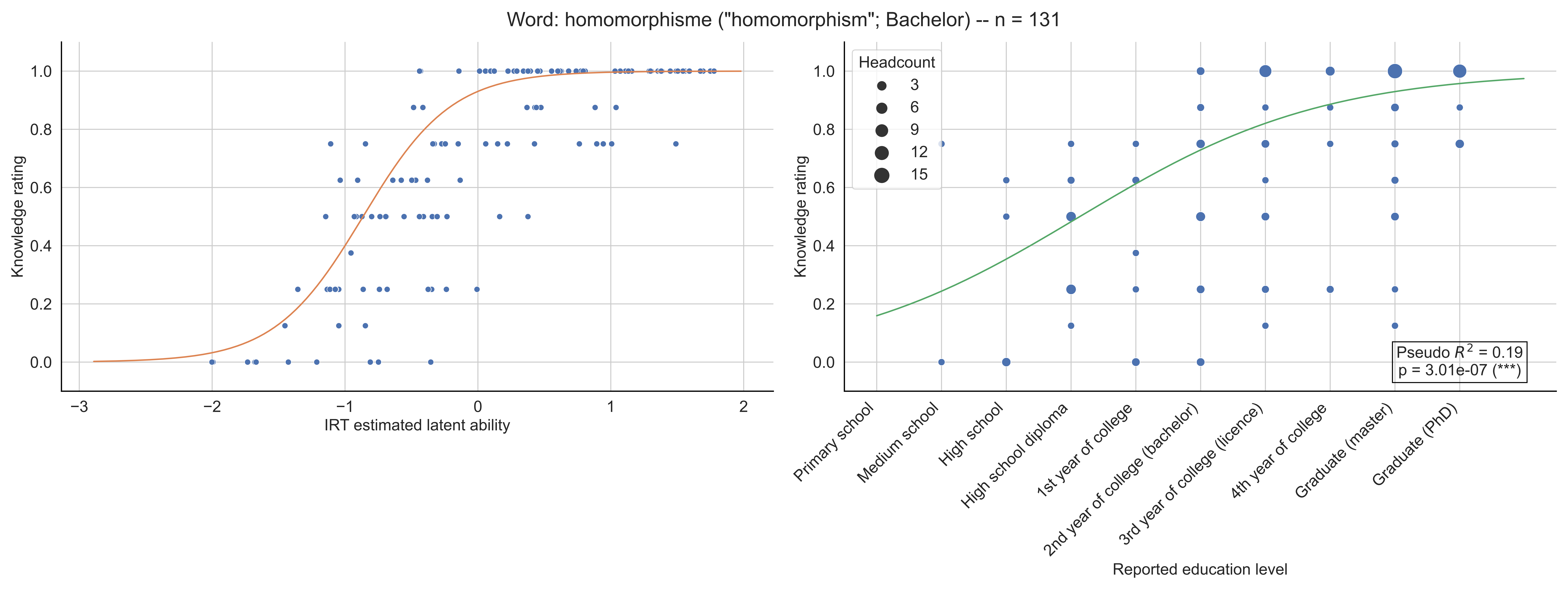
Word: homomorphisme ("homomorphism"; Bachelor) -- n = 131
Knowledge rating
IRT estimated latent ability
Headcount
3
6
9
12
15
Pseudo R^2 = 0.19
p = 3.01e-07 (***)
Primary school
Medium school
High school
High school diploma
1st year of college
2nd year of college (bachelor)
3rd year of college (licence)
4th year of college
Graduate (master)
Graduate (PhD)
Reported education level

Word: homothétie ("homothety"; 8-9th grade) -- n = 138

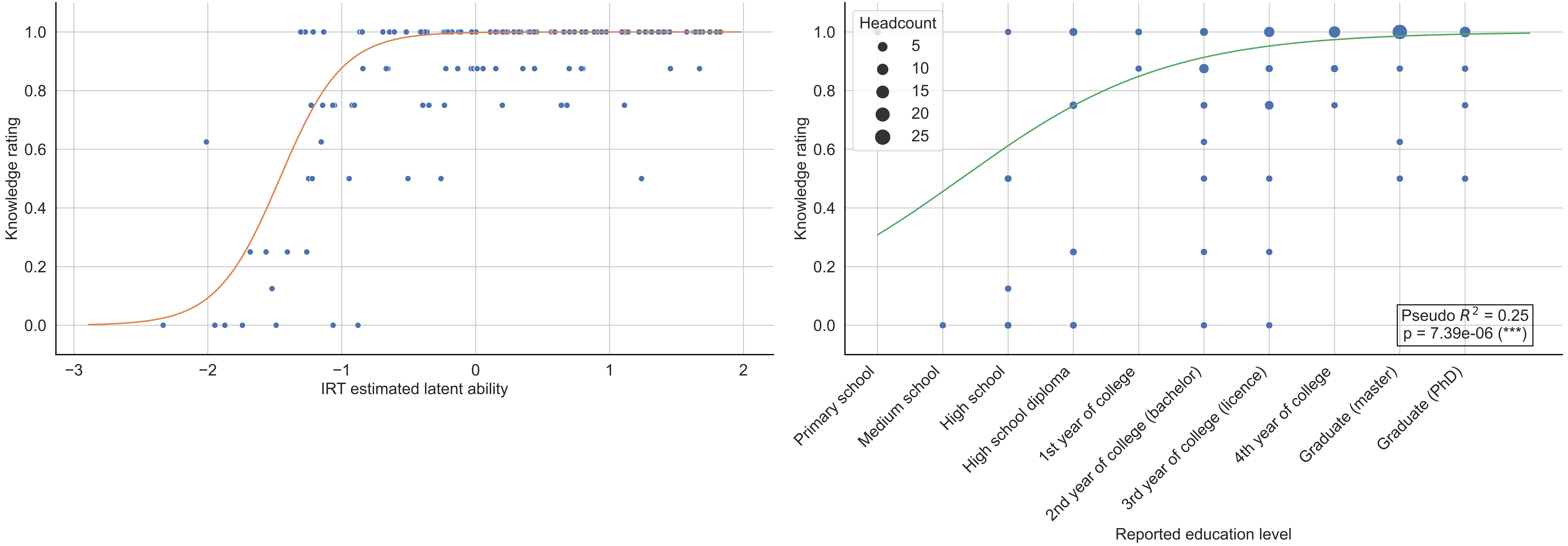

Word: homotopie ("homotopy"; Master) -- n = 131

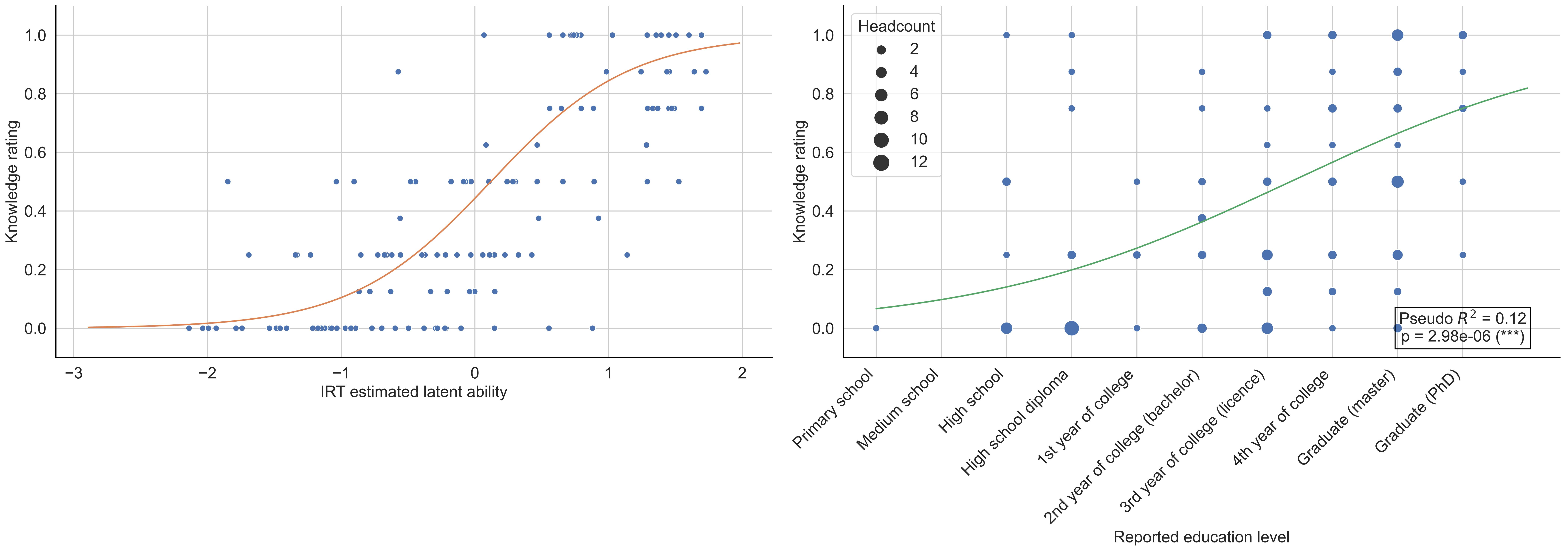

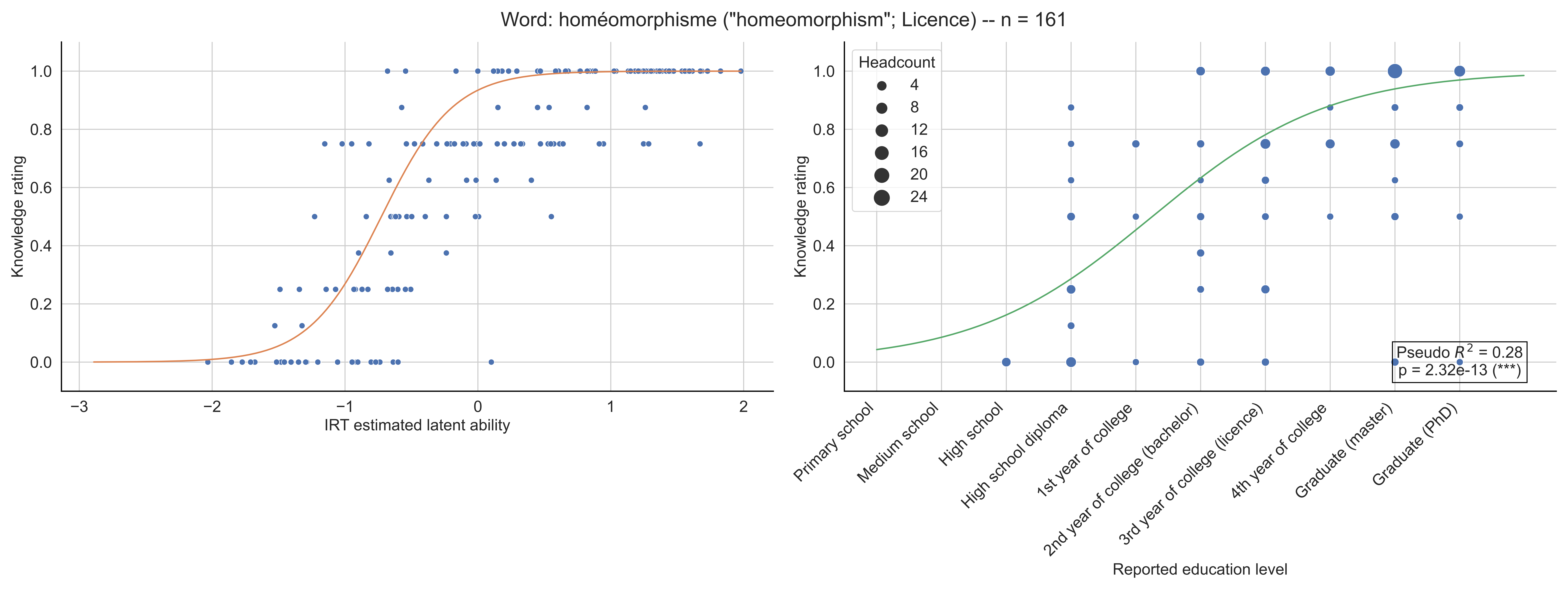

Word: homéomorphisme ("homeomorphism"; Licence) -- n = 161
Knowledge rating
IRT estimated latent ability
Headcount
4
8
12
16
20
24
Pseudo R^2 = 0.28
p = 2.32e-13 (***)
Primary school
Medium school
High school
High school diploma
1st year of college
2nd year of college (bachelor)
3rd year of college (licence)
4th year of college
Graduate (master)
Graduate (PhD)
Reported education level

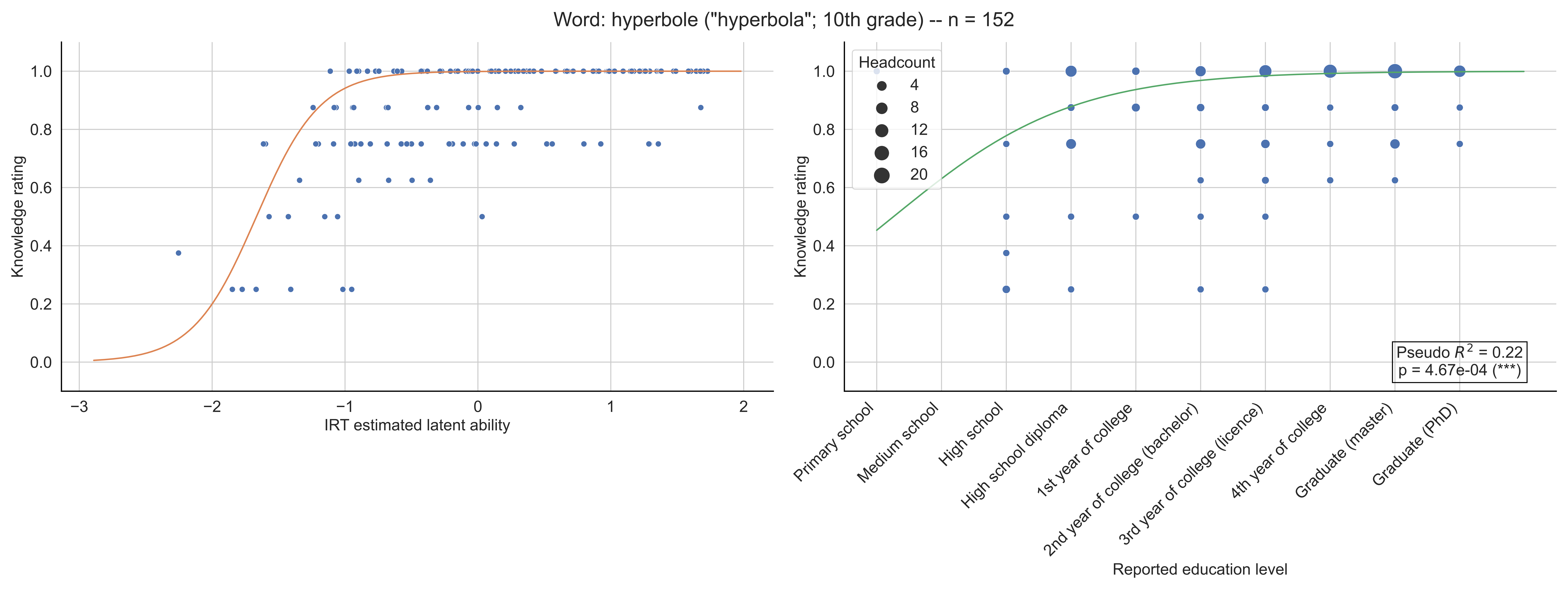

Word: hyperbole ("hyperbola"; 10th grade) -- n = 152
Knowledge rating
IRT estimated latent ability
Headcount
4
8
12
16
20
Pseudo R^2 = 0.22
p = 4.67e-04 (***)
Primary school
Medium school
High school
High school diploma
1st year of college
2nd year of college (bachelor)
3rd year of college (licence)
4th year of college
Graduate (master)
Graduate (PhD)
Reported education level

Word: hypercube ("hypercube"; Bachelor) -- n = 147

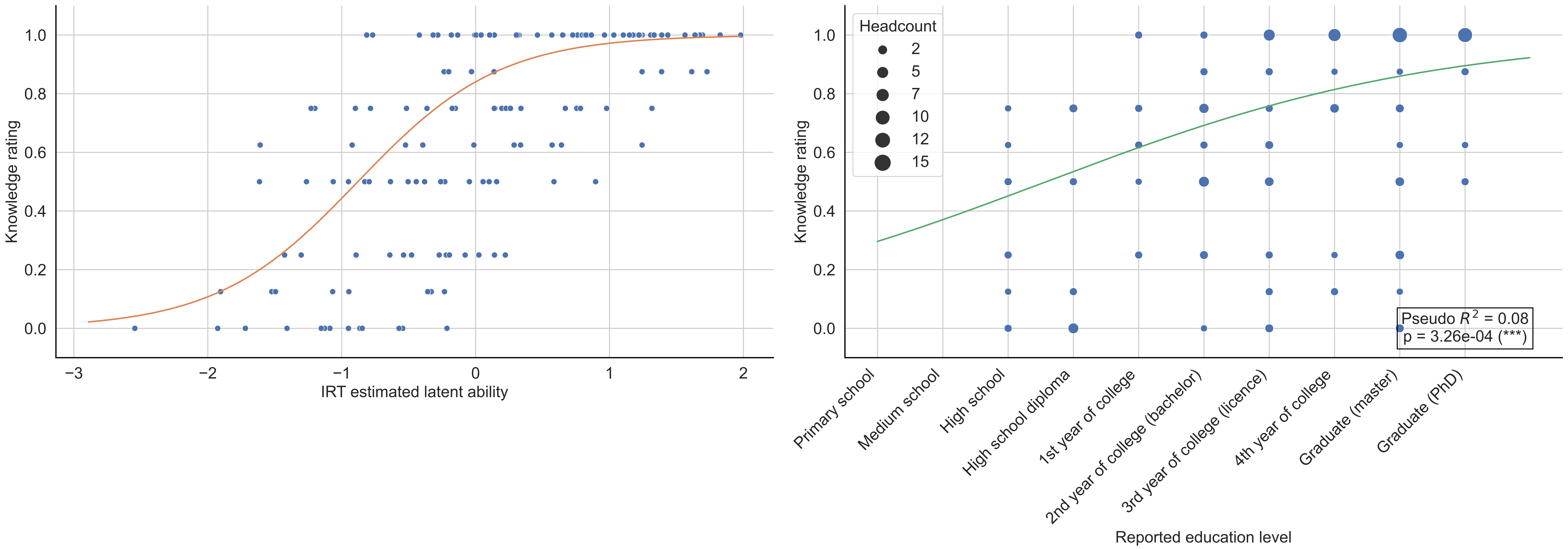

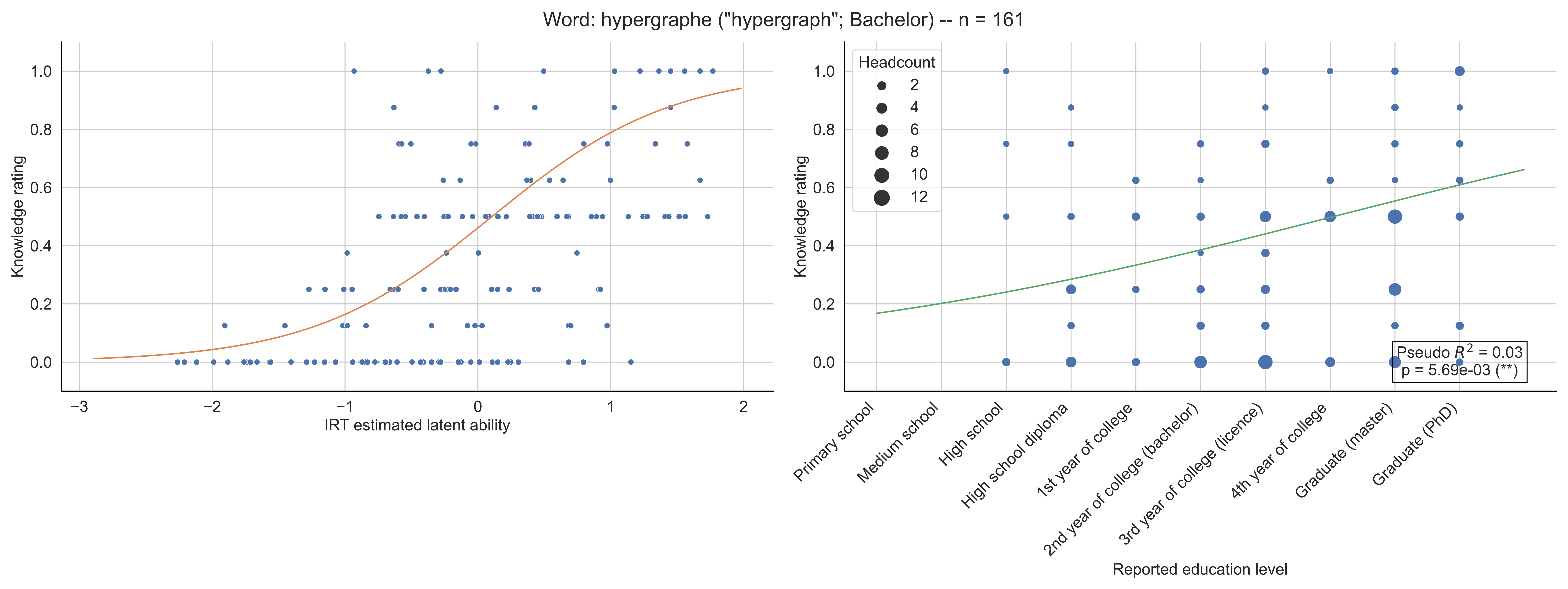

Word: hypergraphe ("hypergraph"; Bachelor) -- n = 161
Knowledge rating
IRT estimated latent ability
Headcount
2
4
6
8
10
12
Pseudo R^2 = 0.03
p = 5.69e-03 (**)
Primary school
Medium school
High school
High school diploma
1st year of college
2nd year of college (bachelor)
3rd year of college (licence)
4th year of college
Graduate (master)
Graduate (PhD)
Reported education level

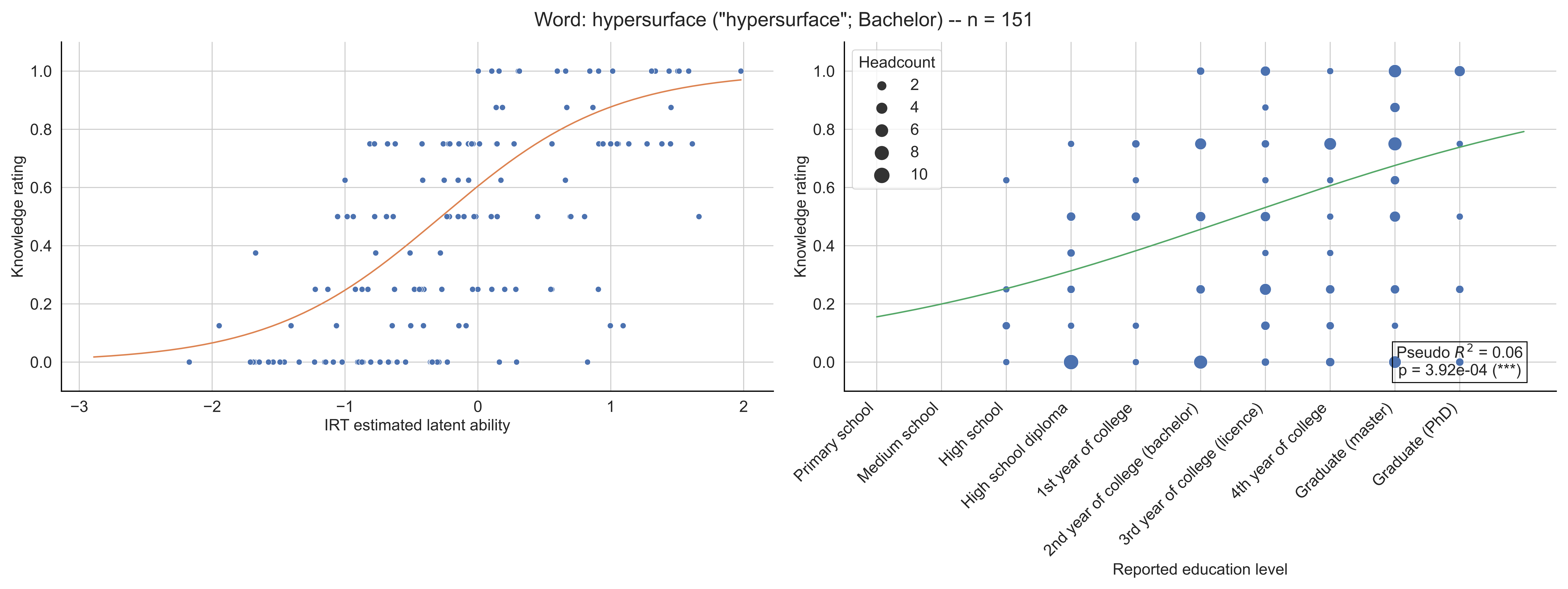

Word: hypersurface ("hypersurface"; Bachelor) -- n = 151
Knowledge rating
IRT estimated latent ability
Headcount
2
4
6
8
10
Pseudo R^2 = 0.06
p = 3.92e-04 (***)
Primary school
Medium school
High school
High school diploma
1st year of college
2nd year of college (bachelor)
3rd year of college (licence)
4th year of college
Graduate (master)
Graduate (PhD)
Reported education level

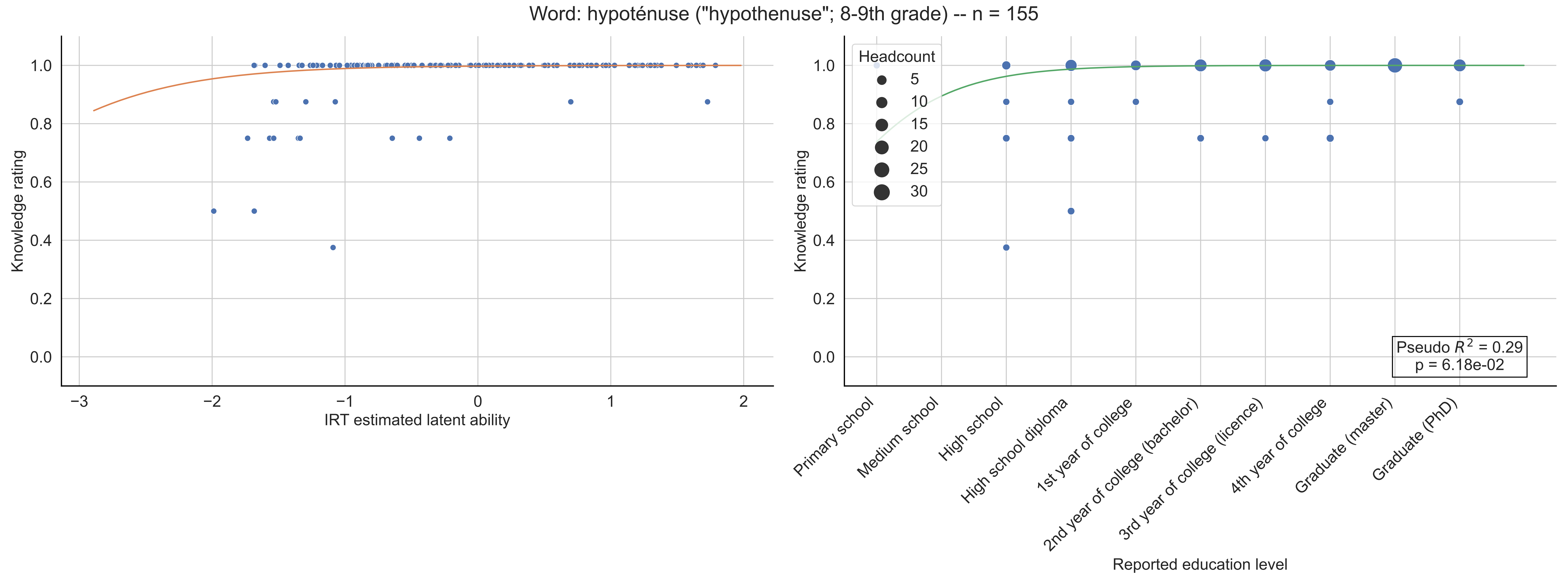

Word: hypoténuse ("hypothenuse"; 8-9th grade) -- n = 155
Knowledge rating
IRT estimated latent ability
Headcount
5
10
15
20
25
30
Primary school
Medium school
High school
High school diploma
1st year of college
2nd year of college (bachelor)
3rd year of college (licence)
4th year of college
Graduate (master)
Graduate (PhD)
Reported education level
Pseudo R^2 = 0.29
p = 6.18e-02

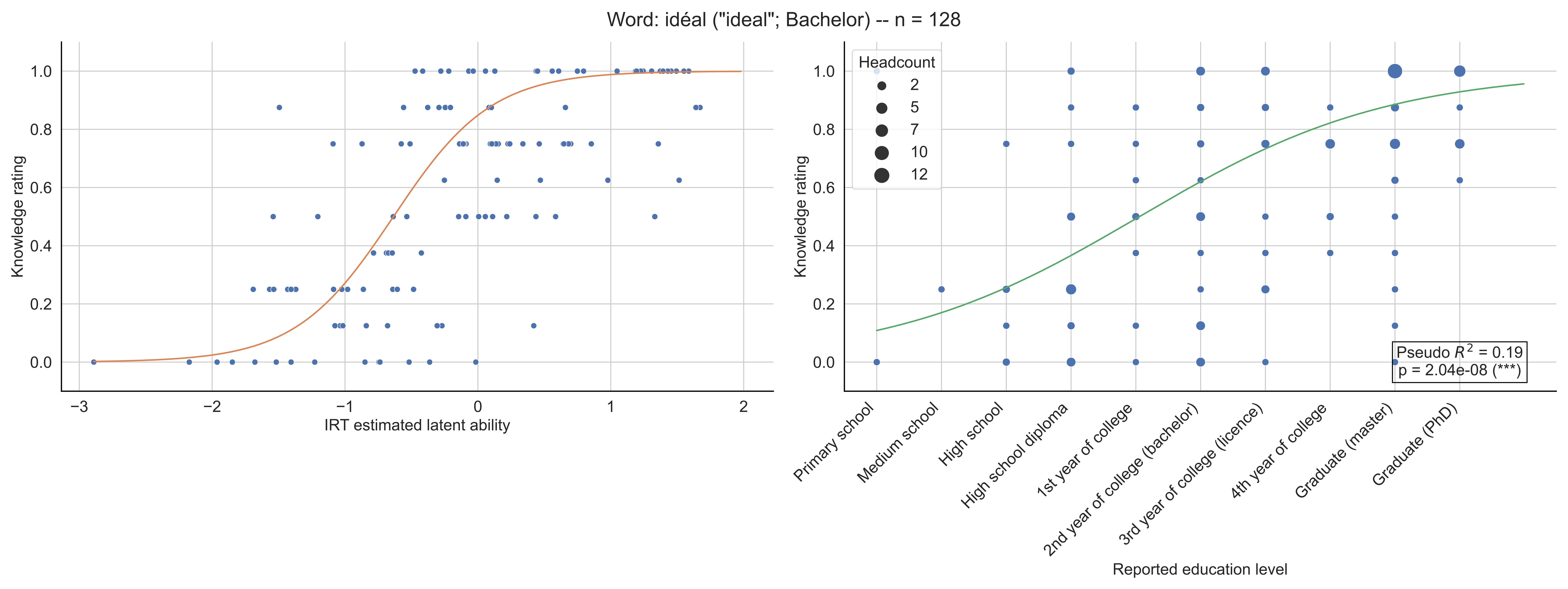
Word: idéal ("ideal"; Bachelor) -- n = 128
Knowledge rating
IRT estimated latent ability
Headcount
2
5
7
10
12
Pseudo R^2 = 0.19
p = 2.04e-08 (***)
Primary school
Medium school
High school
High school diploma
1st year of college
2nd year of college (bachelor)
3rd year of college (licence)
4th year of college
Graduate (master)
Graduate (PhD)
Reported education level

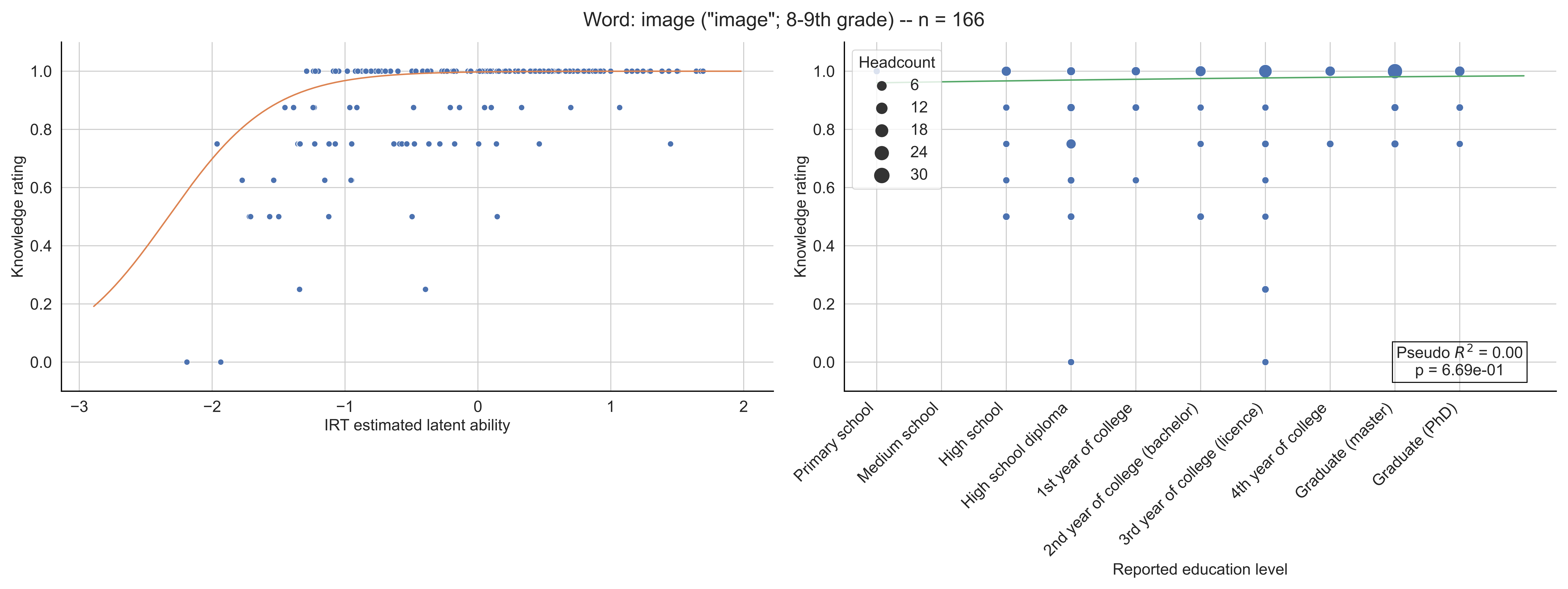
Word: image ("image"; 8-9th grade) -- n = 166
Knowledge rating
IRT estimated latent ability
−3
−2
−1
0
1
2
0.0
0.2
0.4
0.6
0.8
1.0
Headcount
6
12
18
24
30
Pseudo R^2 = 0.00
p = 6.69e-01
Primary school
Medium school
High school
High school diploma
1st year of college
2nd year of college (bachelor)
3rd year of college (licence)
4th year of college
Graduate (master)
Graduate (PhD)
Reported education level

Word: incomplétude ("incompleteness"; Licence) -- n = 131

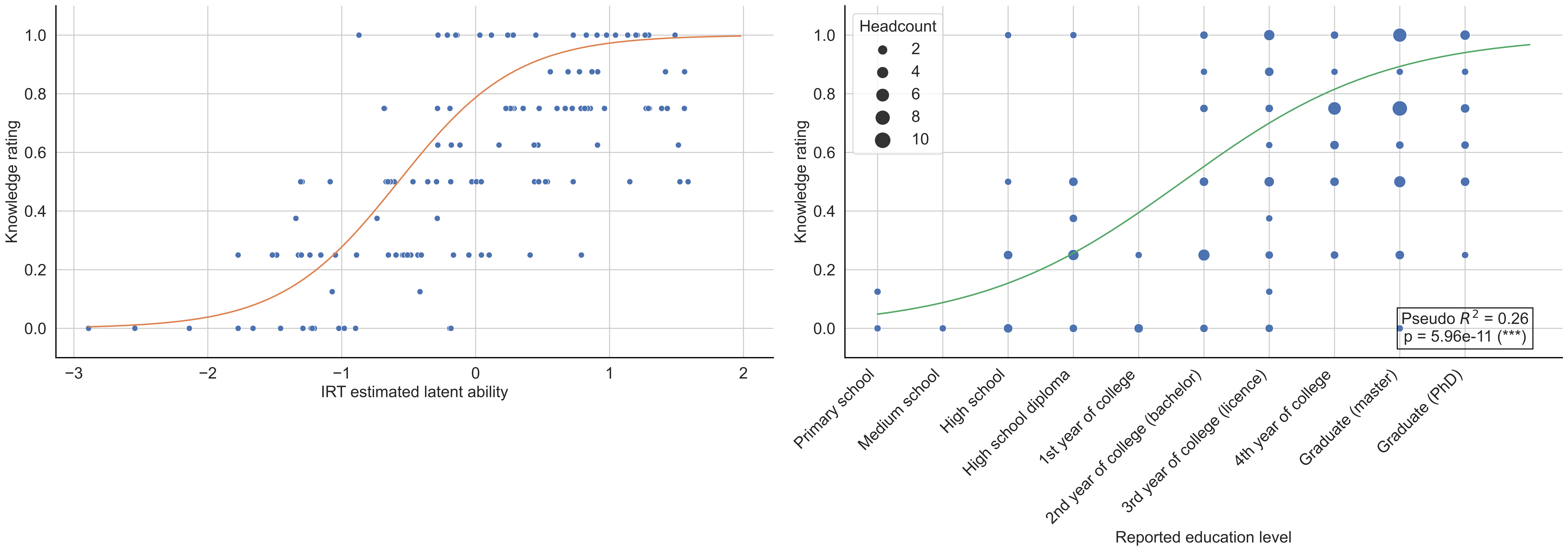

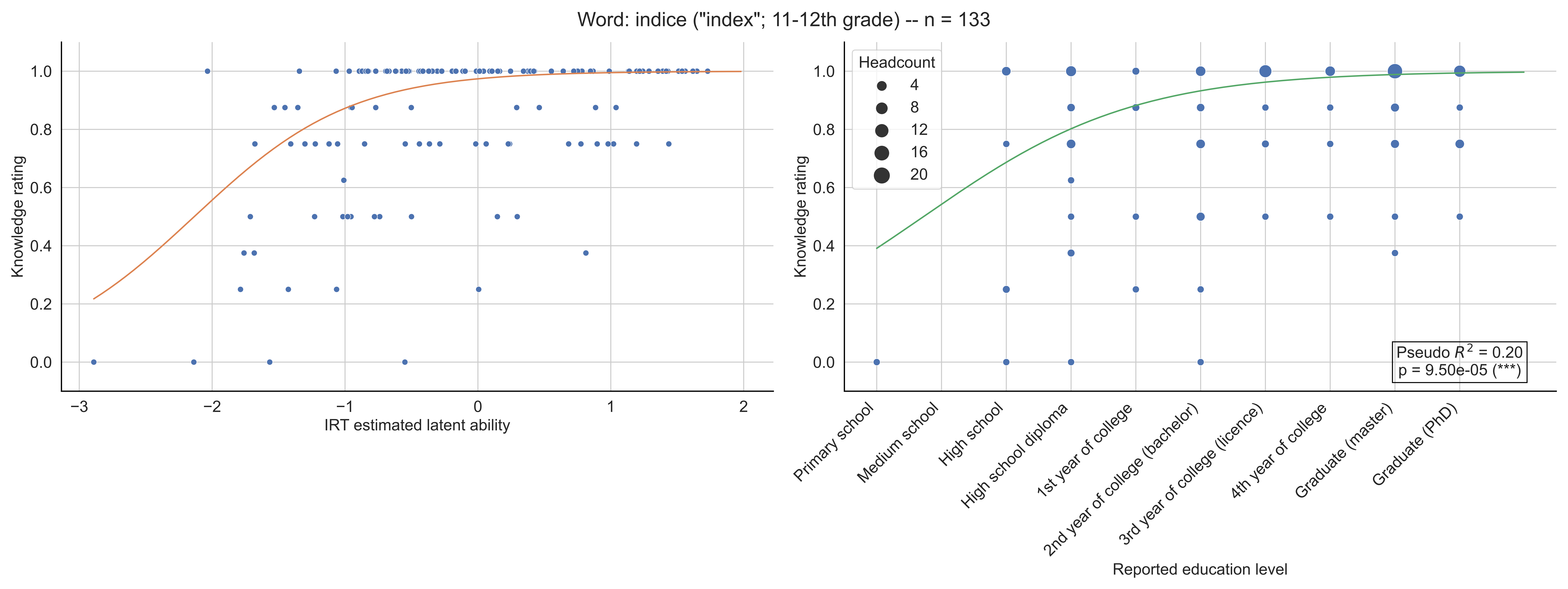

Word: indice ("index"; 11-12th grade) -- n = 133
Knowledge rating
IRT estimated latent ability
Headcount
4
8
12
16
20
Pseudo R^2 = 0.20
p = 9.50e-05 (***)
Primary school
Medium school
High school
High school diploma
1st year of college
2nd year of college (bachelor)
3rd year of college (licence)
4th year of college
Graduate (master)
Graduate (PhD)
Reported education level

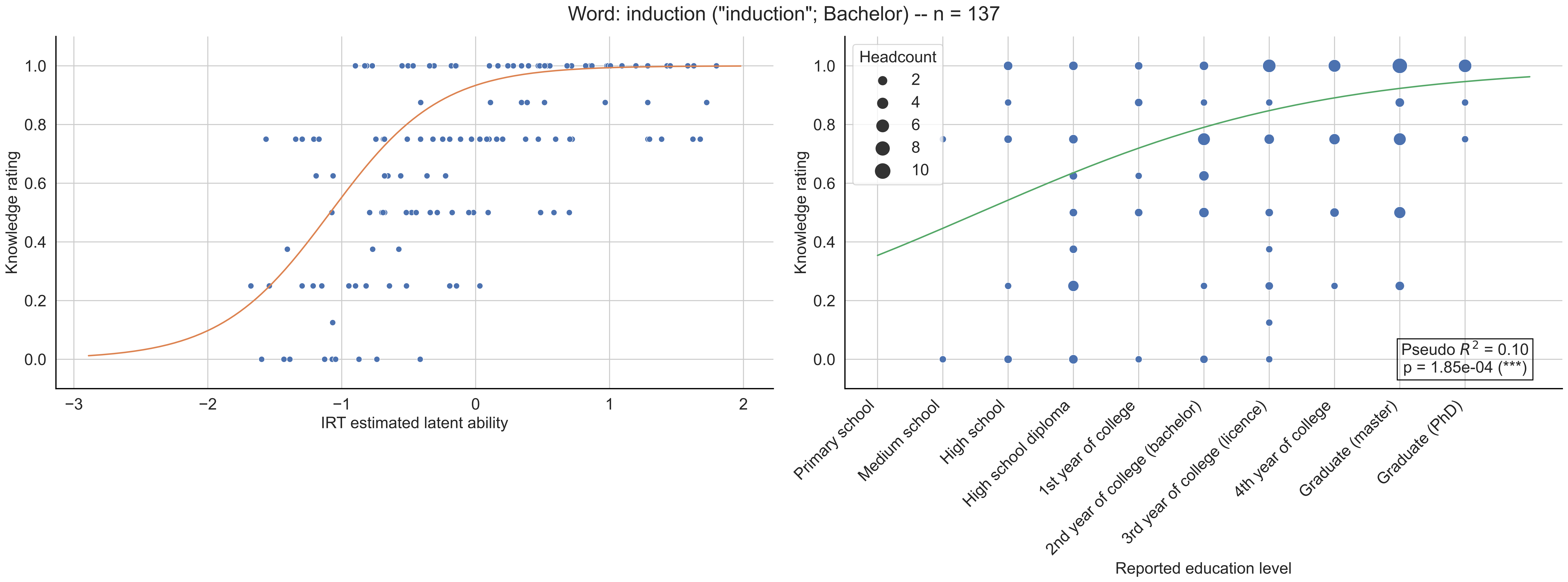

Word: induction ("induction"; Bachelor) -- n = 137
Knowledge rating
IRT estimated latent ability
Headcount
2
4
6
8
10
Primary school
Medium school
High school
High school diploma
1st year of college
2nd year of college (bachelor)
3rd year of college (licence)
4th year of college
Graduate (master)
Graduate (PhD)
Reported education level
Pseudo R^2 = 0.10
p = 1.85e-04 (***)

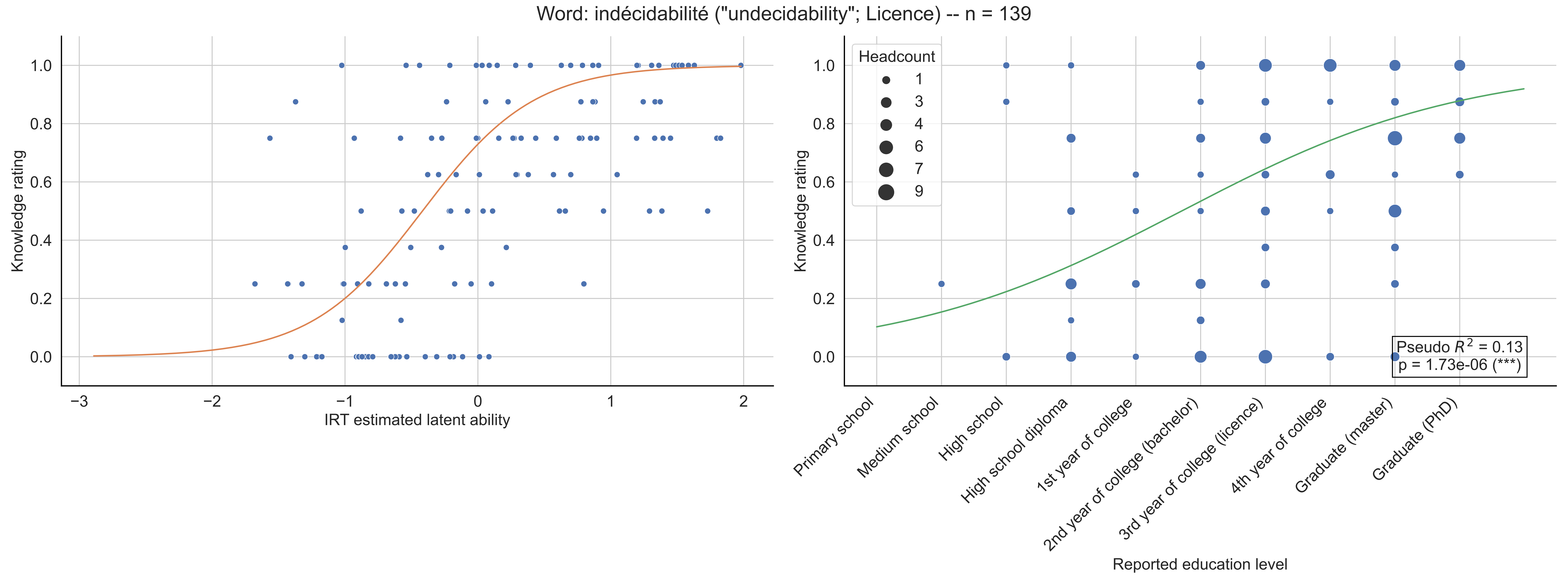
Word: indécidabilité ("undecidability"; Licence) -- n = 139
Knowledge rating
IRT estimated latent ability
Headcount
1
3
4
6
7
9
Pseudo R^2 = 0.13
p = 1.73e-06 (***)
Primary school
Medium school
High school
High school diploma
1st year of college
2nd year of college (bachelor)
3rd year of college (licence)
4th year of college
Graduate (master)
Graduate (PhD)
Reported education level

Word: indépendance ("independence"; 11-12th grade) -- n = 147

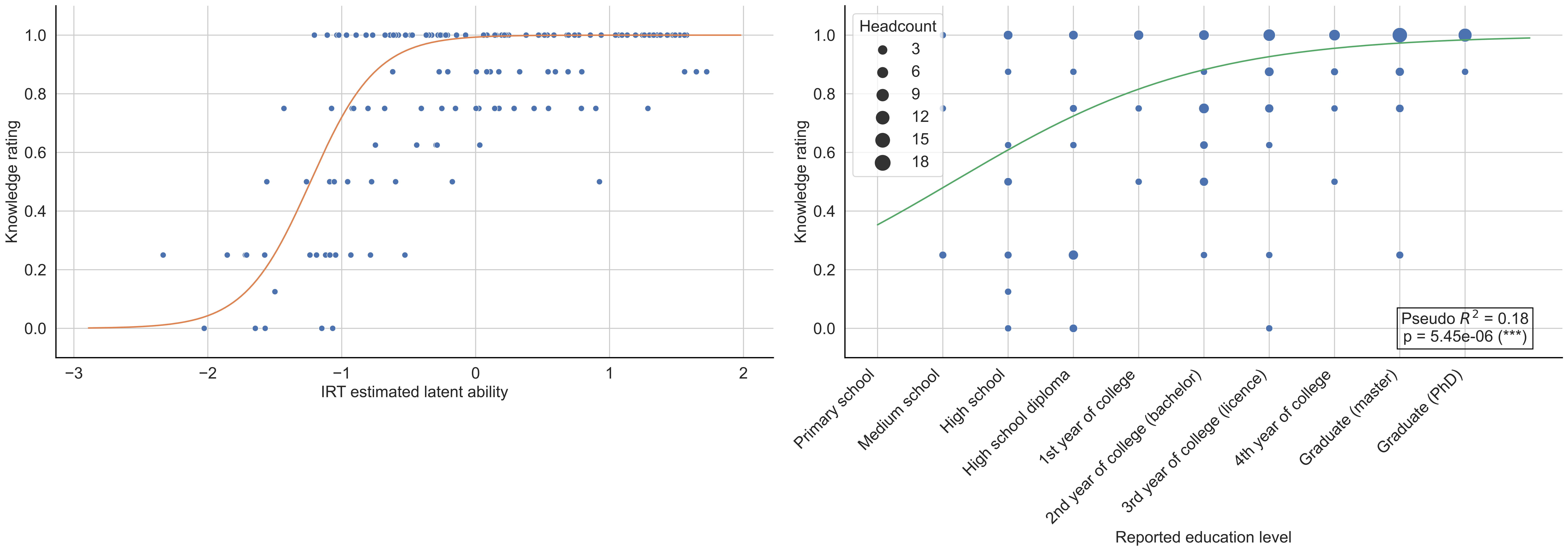

Word: infini ("inifinity, infinite"; 10th grade) -- n = 177

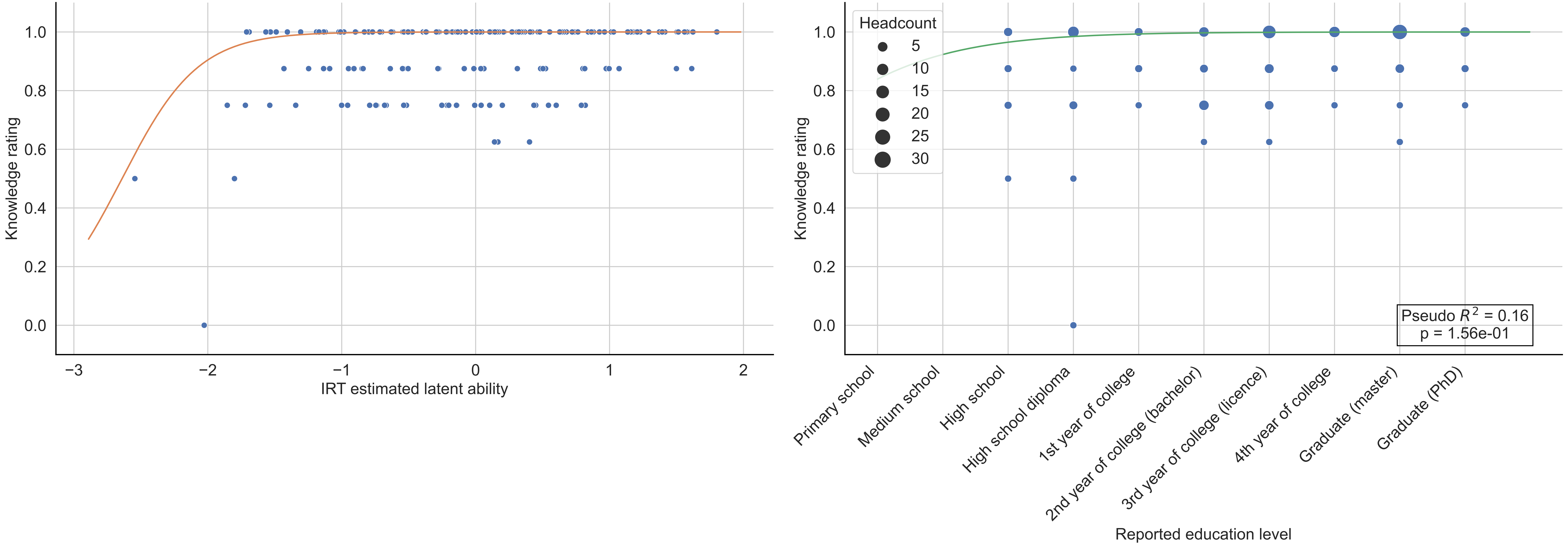

Word: informatique ("computer science"; Primary school) -- n = 131

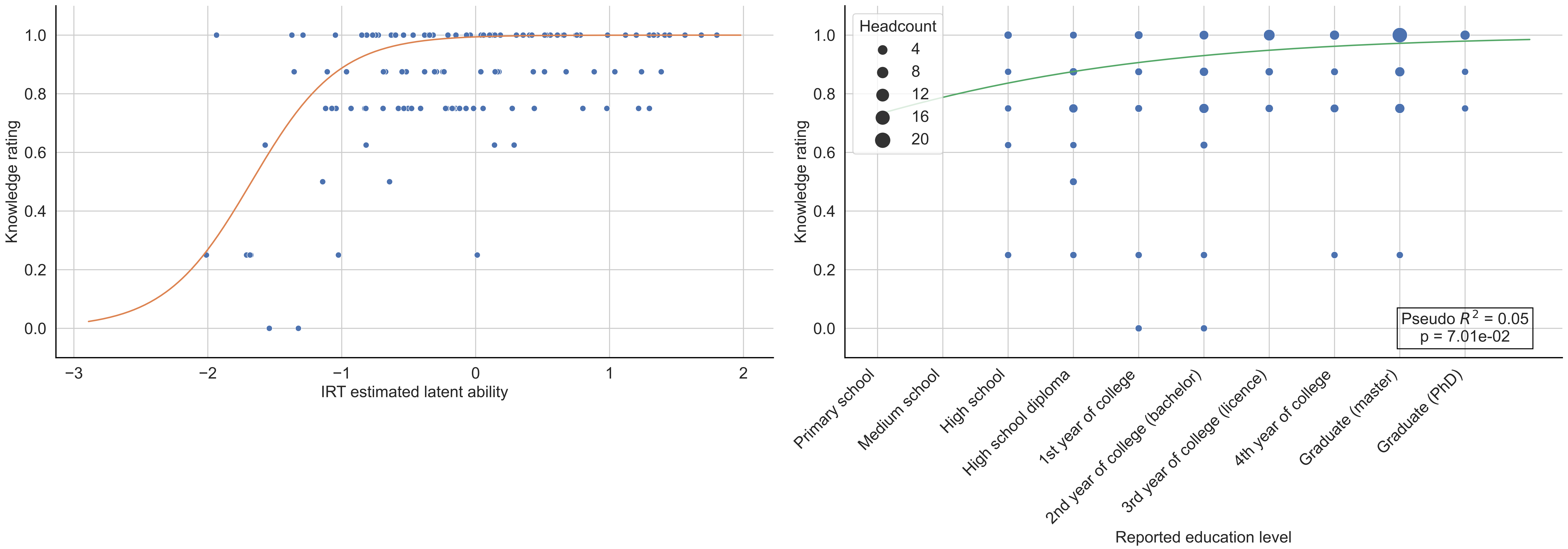

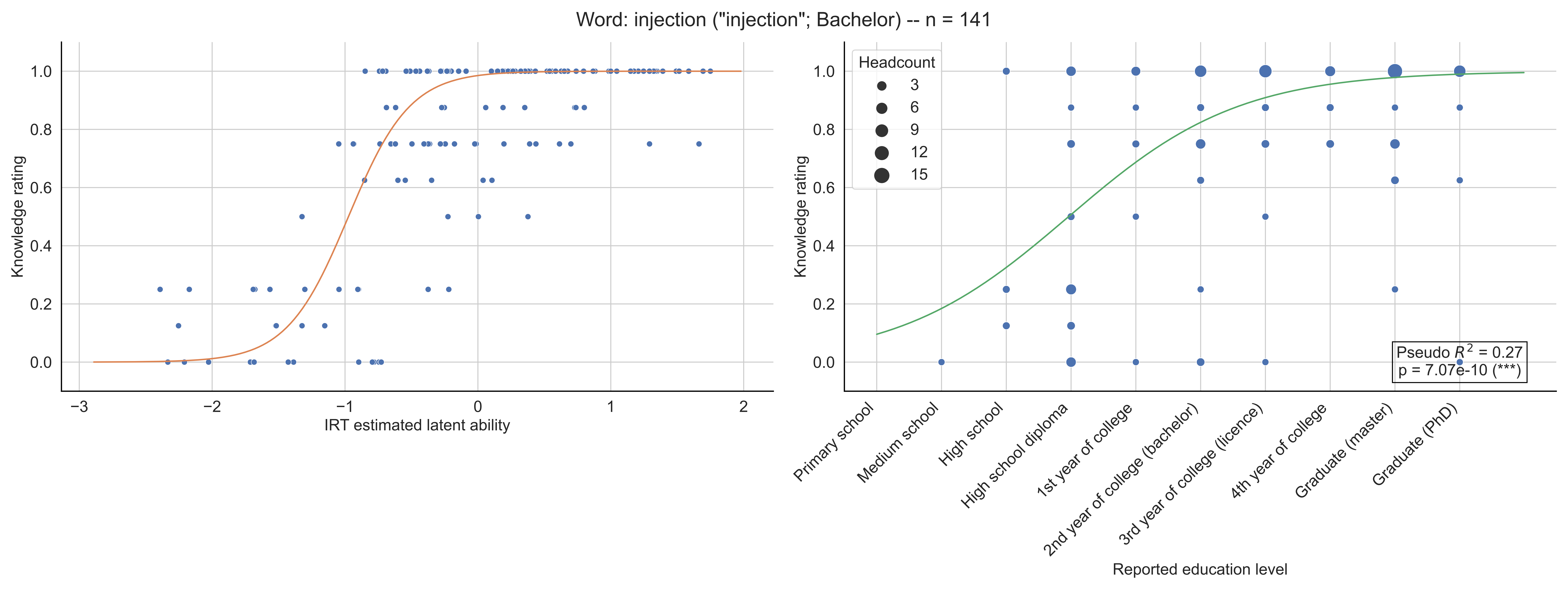

Word: injection ("injection"; Bachelor) -- n = 141
Knowledge rating
IRT estimated latent ability
Headcount
3
6
9
12
15
Pseudo R^2 = 0.27
p = 7.07e-10 (***)
Primary school
Medium school
High school
High school diploma
1st year of college
2nd year of college (bachelor)
3rd year of college (licence)
4th year of college
Graduate (master)
Graduate (PhD)
Reported education level

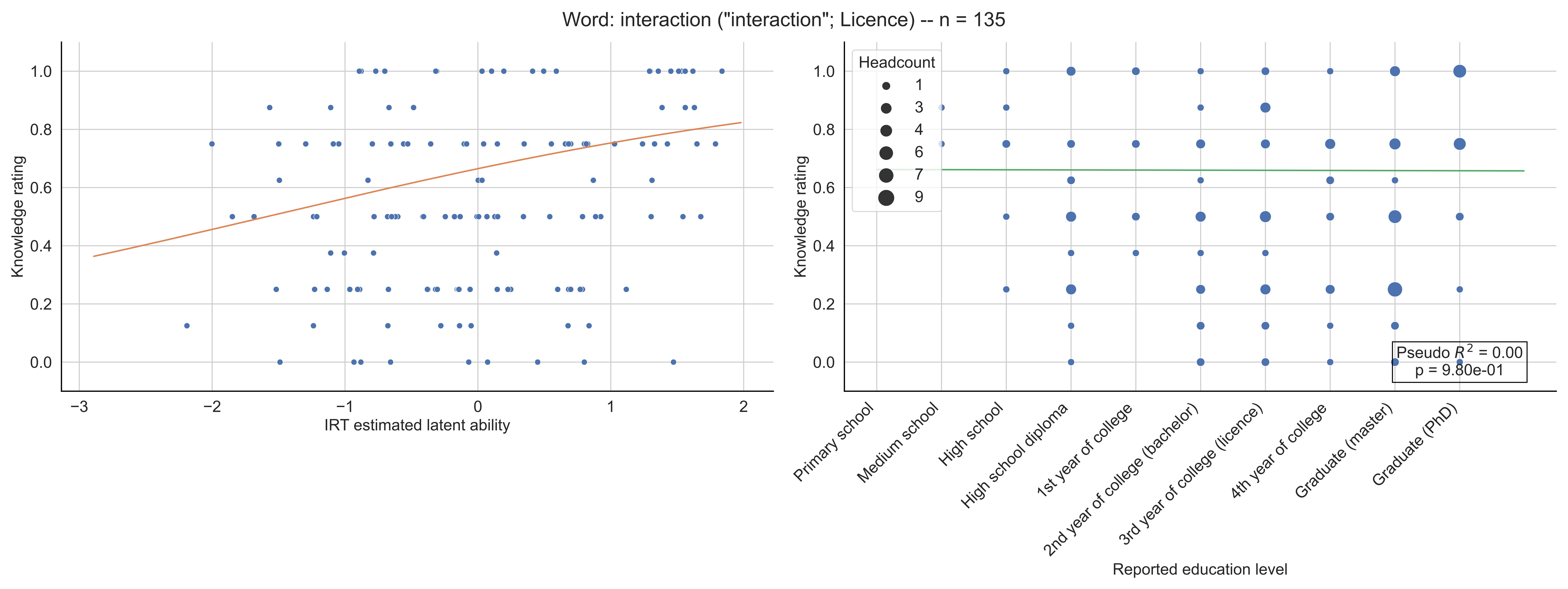

Word: interaction ("interaction"; Licence) -- n = 135
Knowledge rating
IRT estimated latent ability
Headcount
1
3
4
6
7
9
Pseudo R^2 = 0.00
p = 9.80e-01
Primary school
Medium school
High school
High school diploma
1st year of college
2nd year of college (bachelor)
3rd year of college (licence)
4th year of college
Graduate (master)
Graduate (PhD)
Reported education level

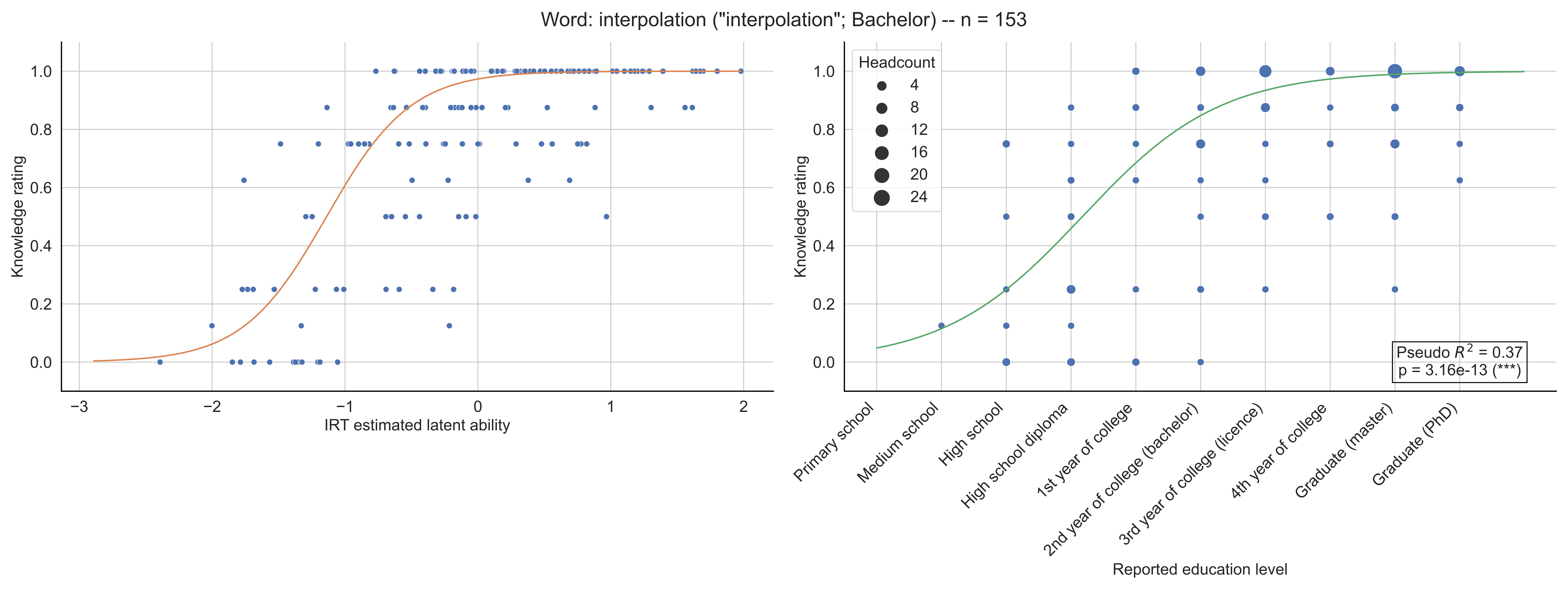
Word: interpolation ("interpolation"; Bachelor) -- n = 153
Knowledge rating
IRT estimated latent ability
Headcount
4
8
12
16
20
24
Pseudo R^2 = 0.37
p = 3.16e-13 (***)
Primary school
Medium school
High school
High school diploma
1st year of college
2nd year of college (bachelor)
3rd year of college (licence)
4th year of college
Graduate (master)
Graduate (PhD)
Reported education level

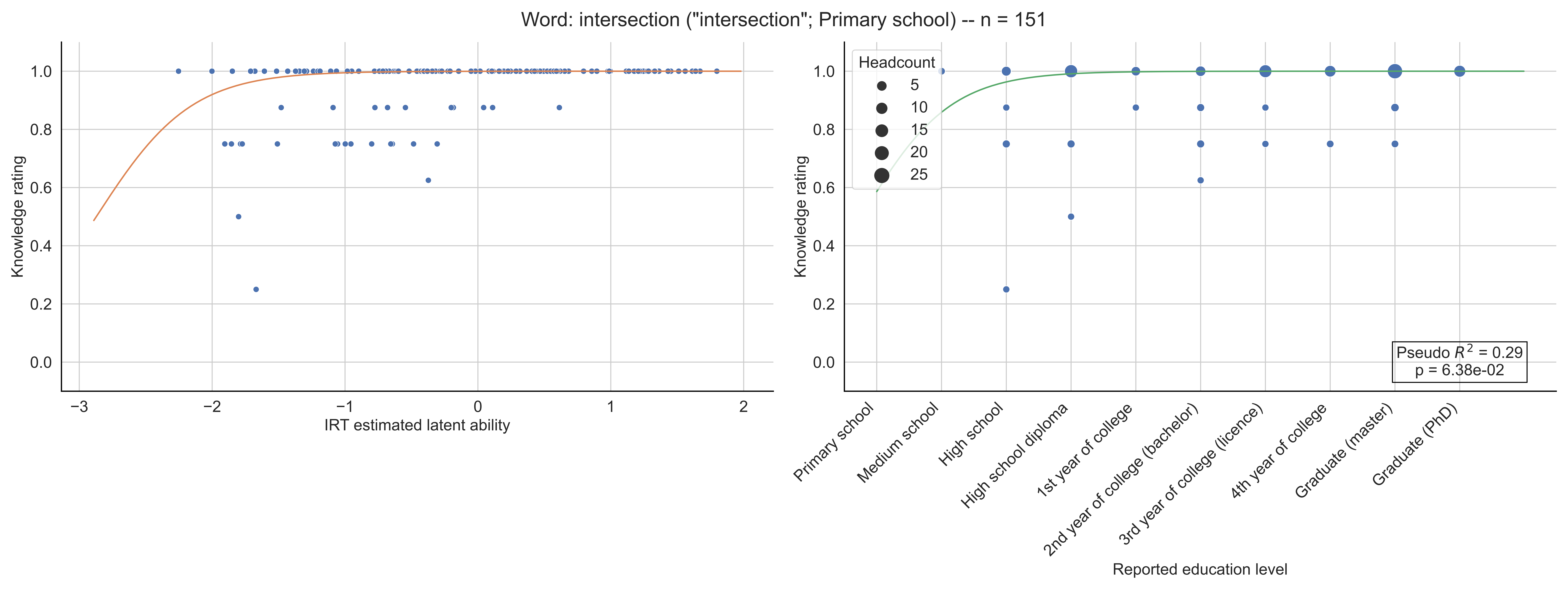

Word: intersection ("intersection"; Primary school) -- n = 151
Knowledge rating
IRT estimated latent ability
Headcount
5
10
15
20
25
Pseudo R^2 = 0.29
p = 6.38e-02
Primary school
Medium school
High school
High school diploma
1st year of college
2nd year of college (bachelor)
3rd year of college (licence)
4th year of college
Graduate (master)
Graduate (PhD)
Reported education level

Word: intégrale ("integral"; 11-12th grade) -- n = 152

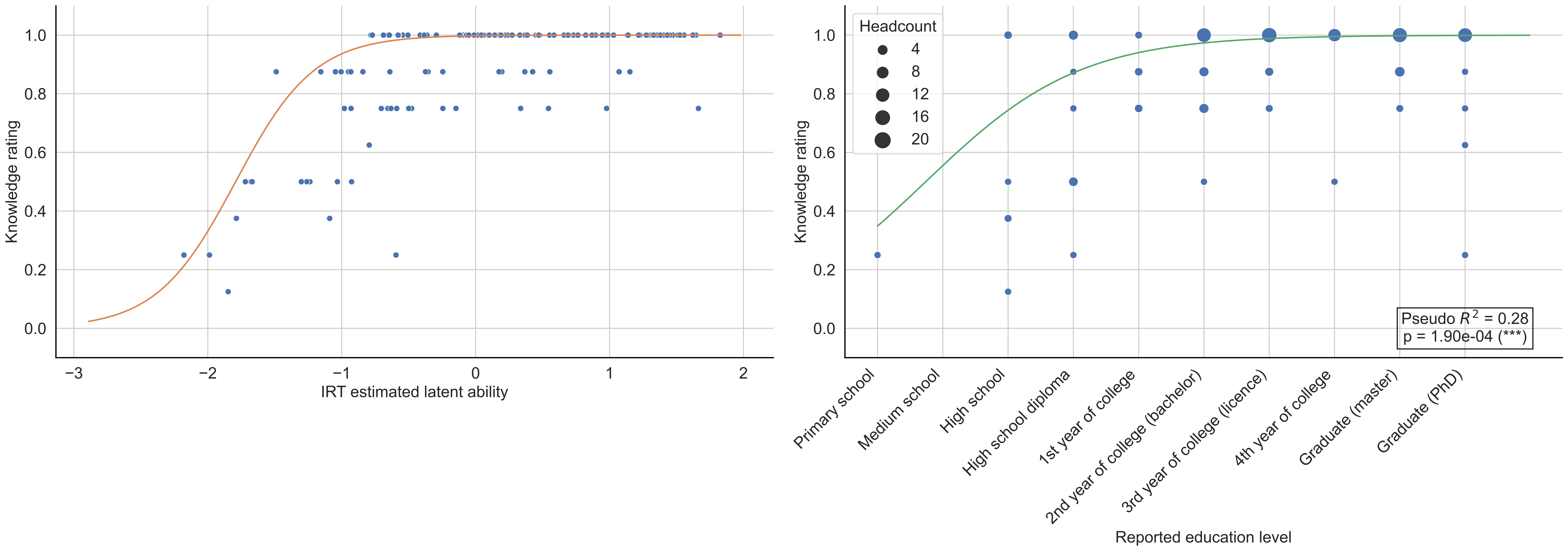

Word: intérieur ("interior"; Primary school) -- n = 159

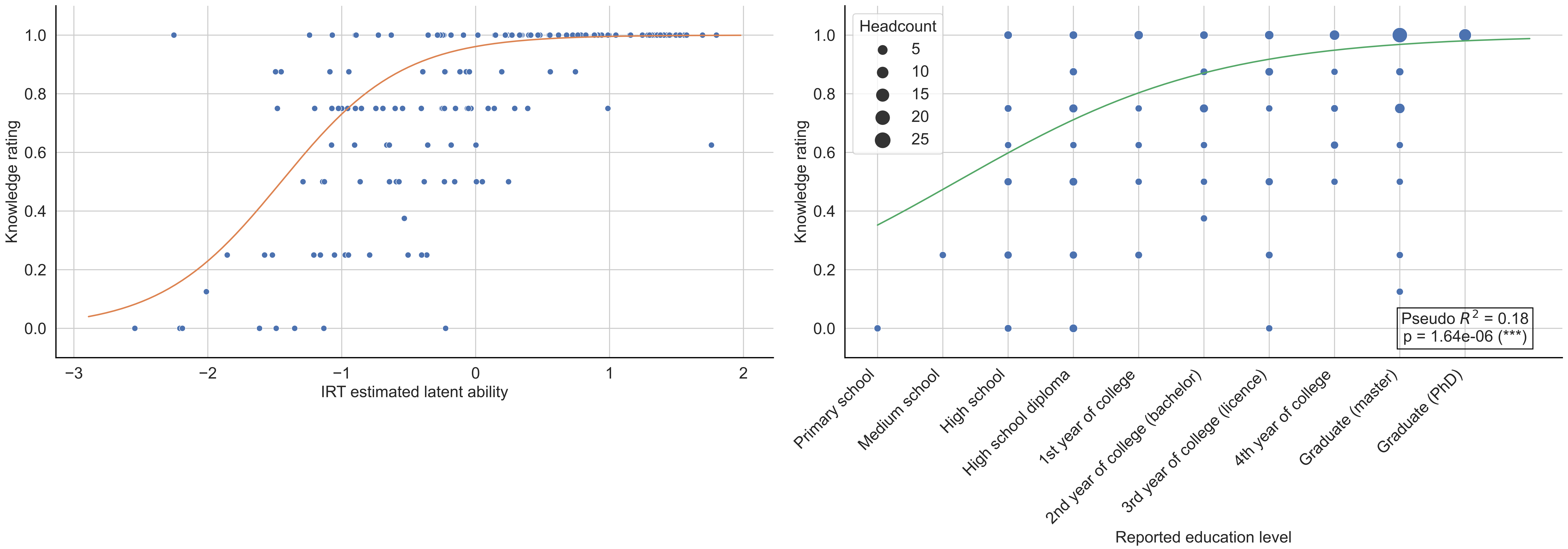

Word: invariant ("invariant"; 11-12th grade) -- n = 133

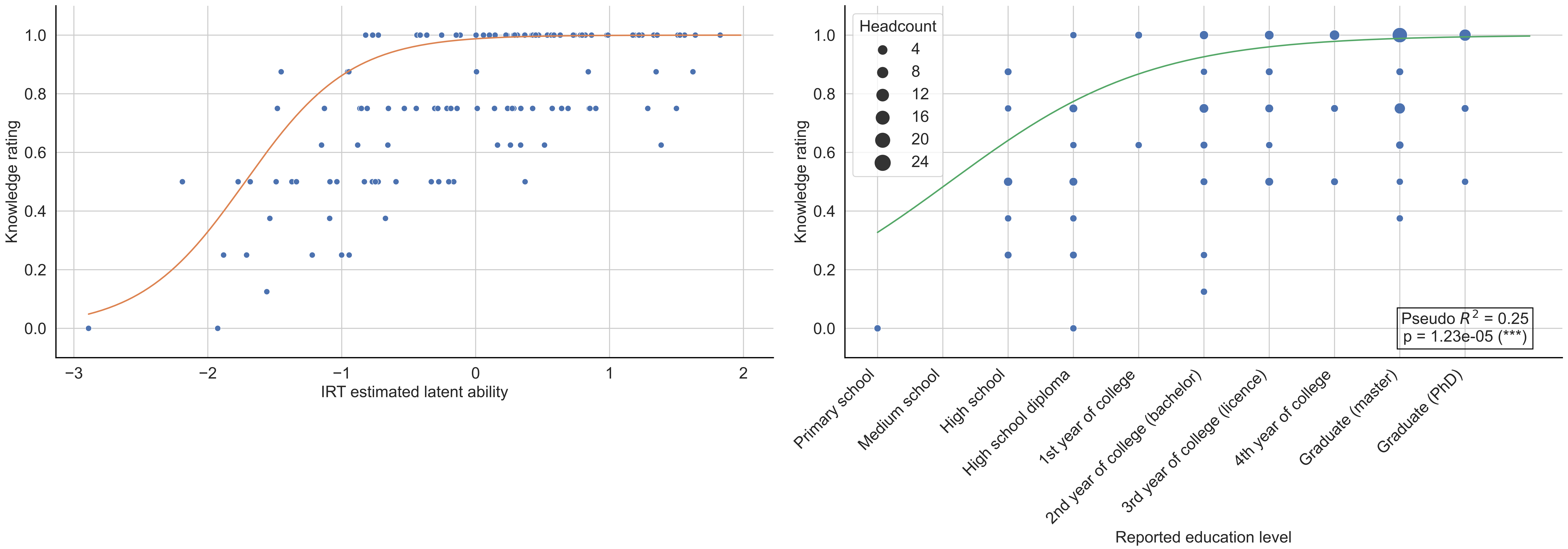

Word: inversible ("invertible"; Bachelor) -- n = 139

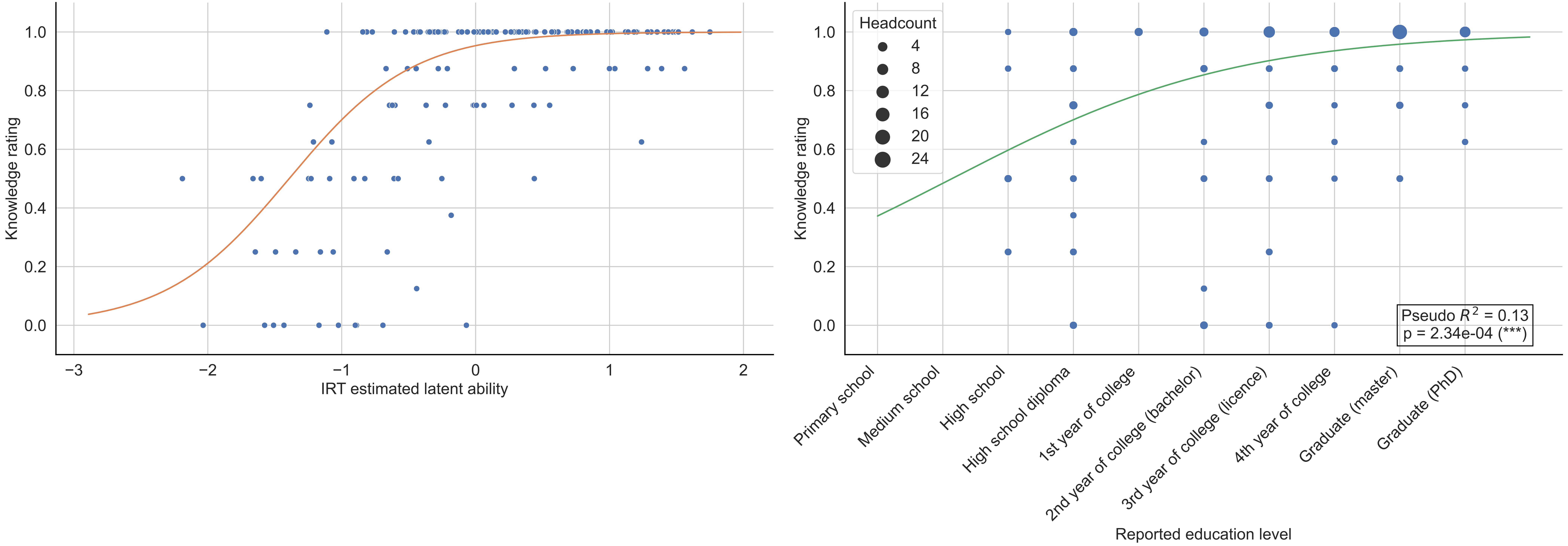

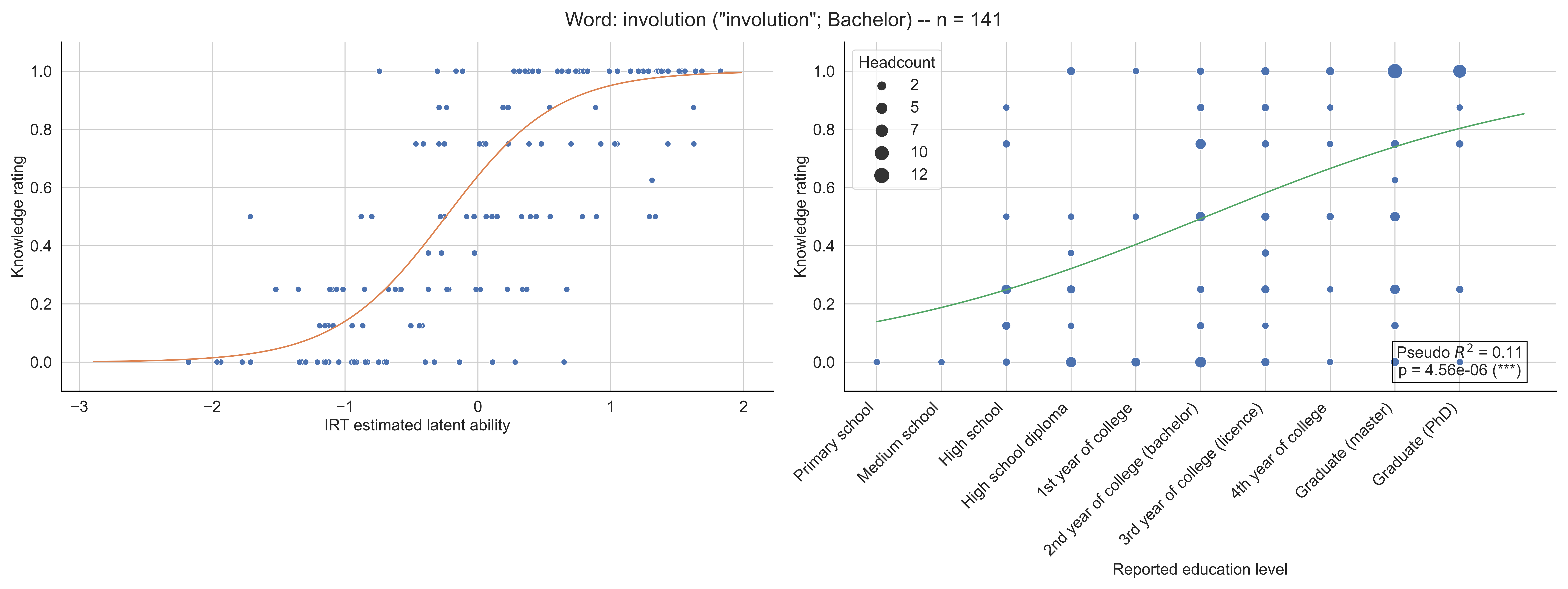

Word: involution ("involution"; Bachelor) -- n = 141
Knowledge rating
IRT estimated latent ability
Headcount
2
5
7
10
12
Pseudo R^2 = 0.11
p = 4.56e-06 (***)
Primary school
Medium school
High school
High school diploma
1st year of college
2nd year of college (bachelor)
3rd year of college (licence)
4th year of college
Graduate (master)
Graduate (PhD)
Reported education level

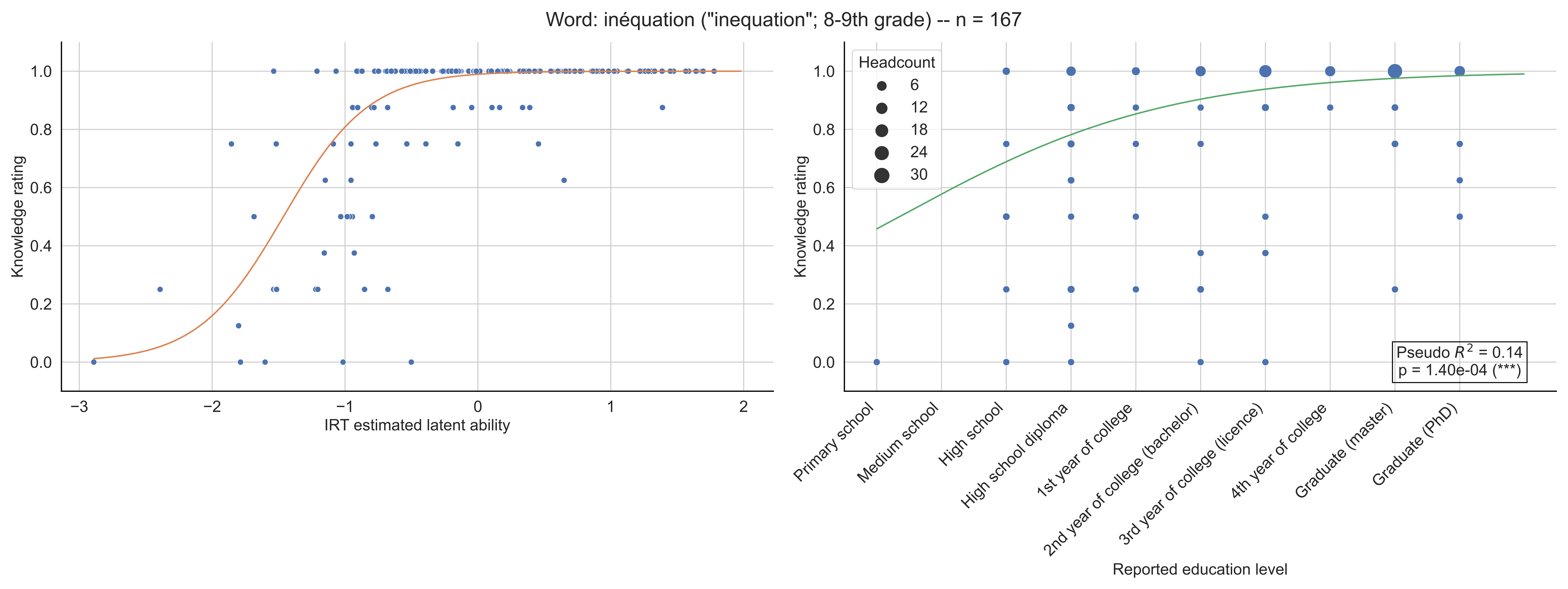
Word: inéquation ("inequation"; 8-9th grade) -- n = 167
Knowledge rating
IRT estimated latent ability
Headcount
6
12
18
24
30
Pseudo R^2 = 0.14
p = 1.40e-04 (***)
Primary school
Medium school
High school
High school diploma
1st year of college
2nd year of college (bachelor)
3rd year of college (licence)
4th year of college
Graduate (master)
Graduate (PhD)
Reported education level

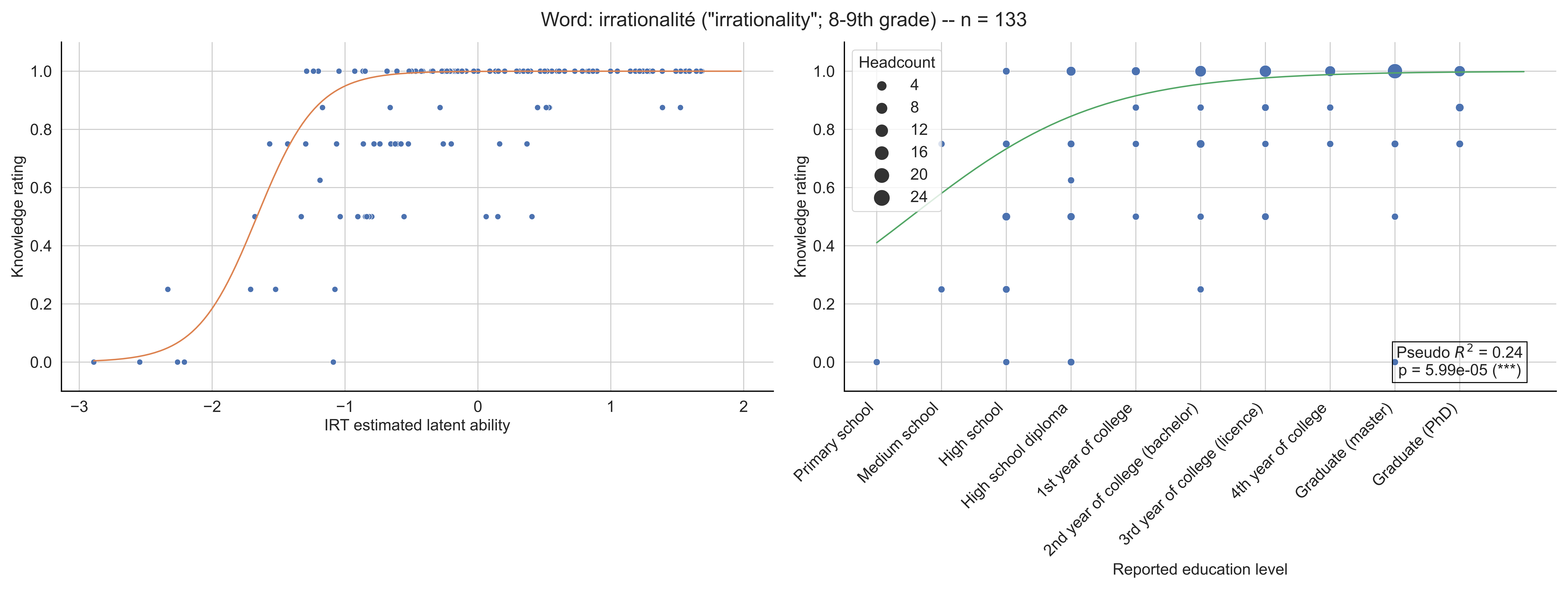

Word: irrationalité ("irrationality"; 8-9th grade) -- n = 133
Knowledge rating
IRT estimated latent ability
Headcount
4
8
12
16
20
24
Pseudo R^2 = 0.24
p = 5.99e-05 (***)
Primary school
Medium school
High school
High school diploma
1st year of college
2nd year of college (bachelor)
3rd year of college (licence)
4th year of college
Graduate (master)
Graduate (PhD)
Reported education level

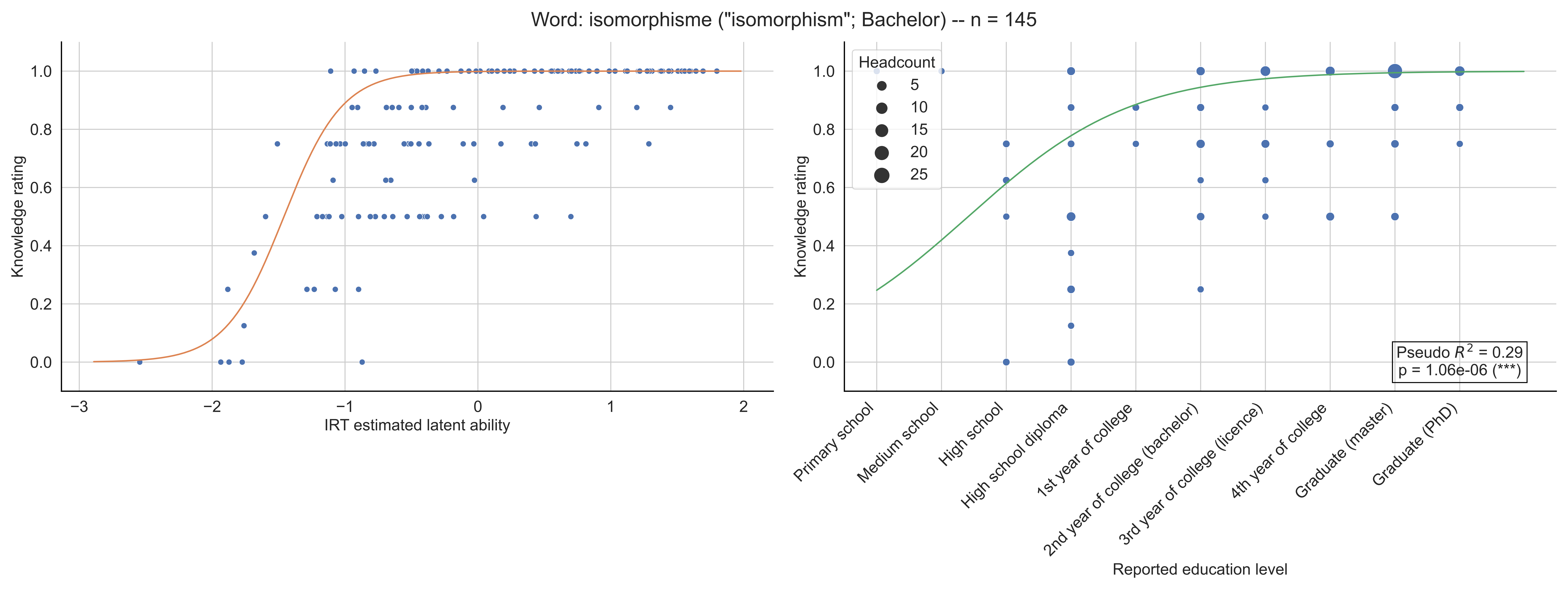

Word: isomorphisme ("isomorphism"; Bachelor) -- n = 145
Knowledge rating
IRT estimated latent ability
Headcount
5
10
15
20
25
Knowledge rating
Primary school
Medium school
High school
High school diploma
1st year of college
2nd year of college (bachelor)
3rd year of college (licence)
4th year of college
Graduate (master)
Graduate (PhD)
Reported education level
Pseudo R^2 = 0.29
p = 1.06e-06 (***)

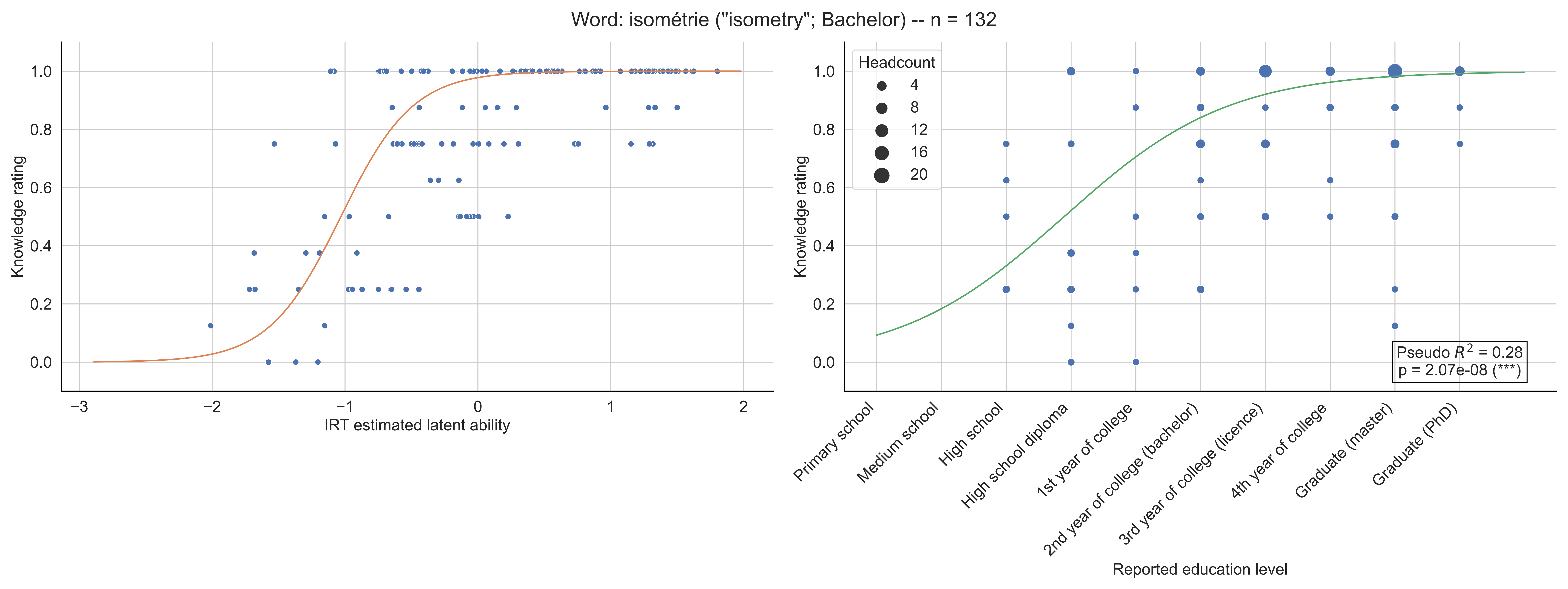

Word: isométrie ("isometry"; Bachelor) -- n = 132
Knowledge rating
IRT estimated latent ability
Headcount
4
8
12
16
20
Pseudo R^2 = 0.28
p = 2.07e-08 (***)
Primary school
Medium school
High school
High school diploma
1st year of college
2nd year of college (bachelor)
3rd year of college (licence)
4th year of college
Graduate (master)
Graduate (PhD)
Reported education level

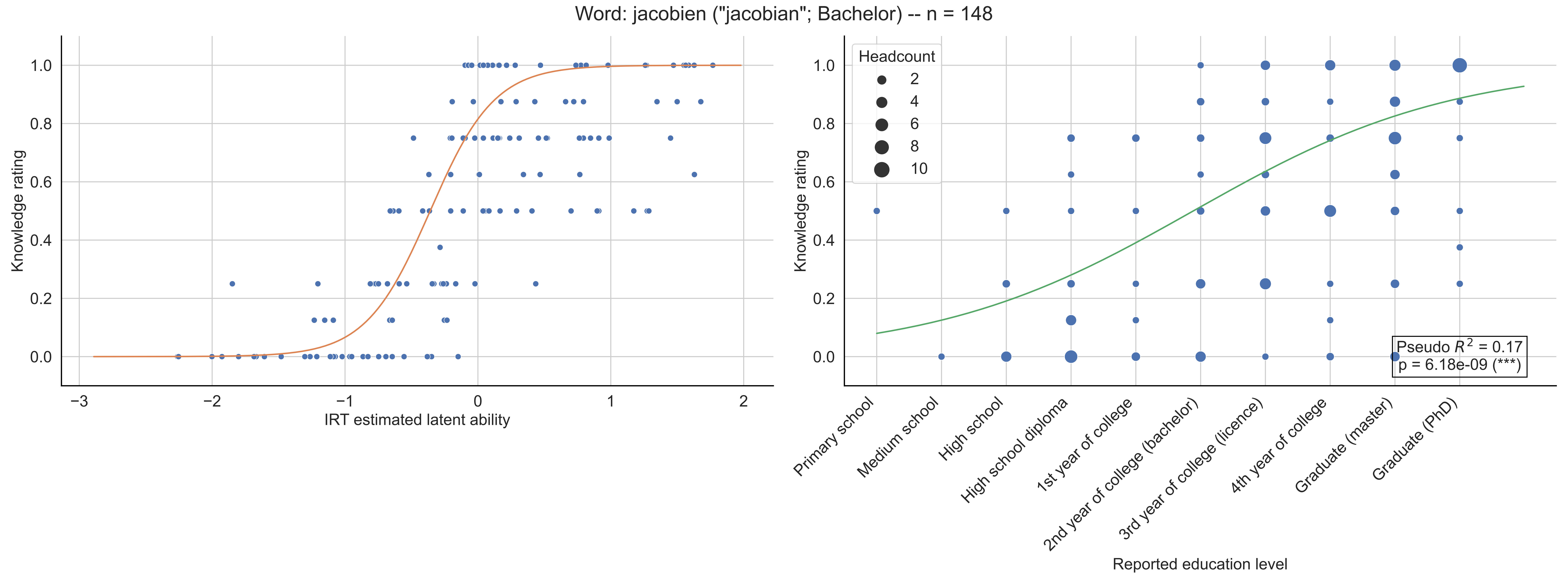

Word: jacobien ("jacobian"; Bachelor) -- n = 148
Knowledge rating
IRT estimated latent ability
Headcount
2
4
6
8
10
Pseudo R^2 = 0.17
p = 6.18e-09 (***)
Primary school
Medium school
High school
High school diploma
1st year of college
2nd year of college (bachelor)
3rd year of college (licence)
4th year of college
Graduate (master)
Graduate (PhD)
Reported education level

Word: lacet ("yaw, lace"; Master) -- n = 142

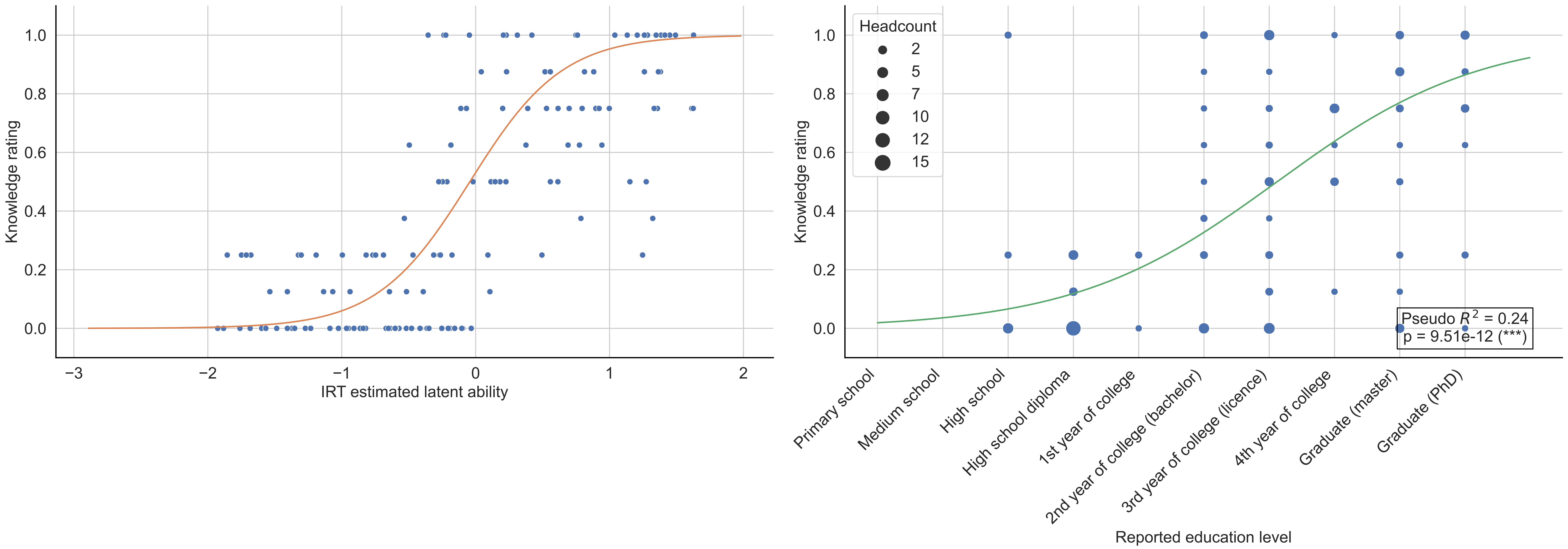

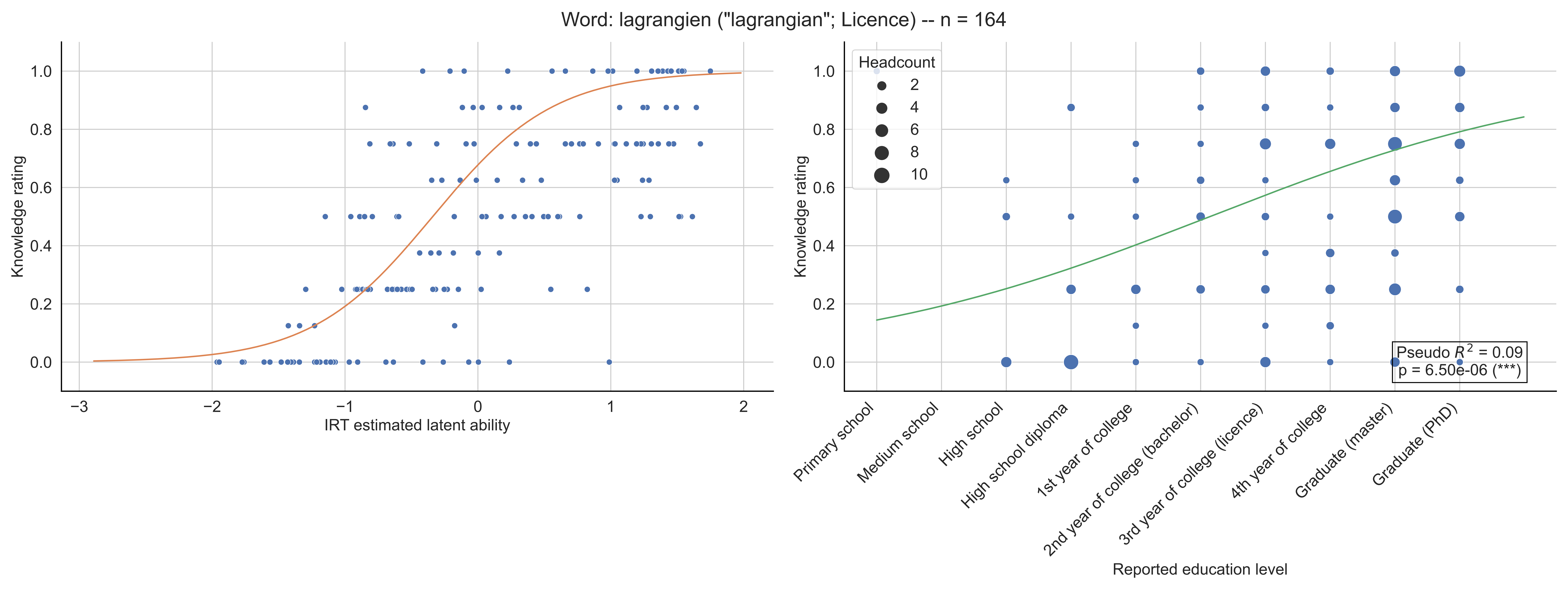

Word: lagrangien ("lagrangian"; Licence) -- n = 164
Knowledge rating
IRT estimated latent ability
Headcount
2
4
6
8
10
Pseudo R^2 = 0.09
p = 6.50e-06 (***)
Primary school
Medium school
High school
High school diploma
1st year of college
2nd year of college (bachelor)
3rd year of college (licence)
4th year of college
Graduate (master)
Graduate (PhD)
Reported education level

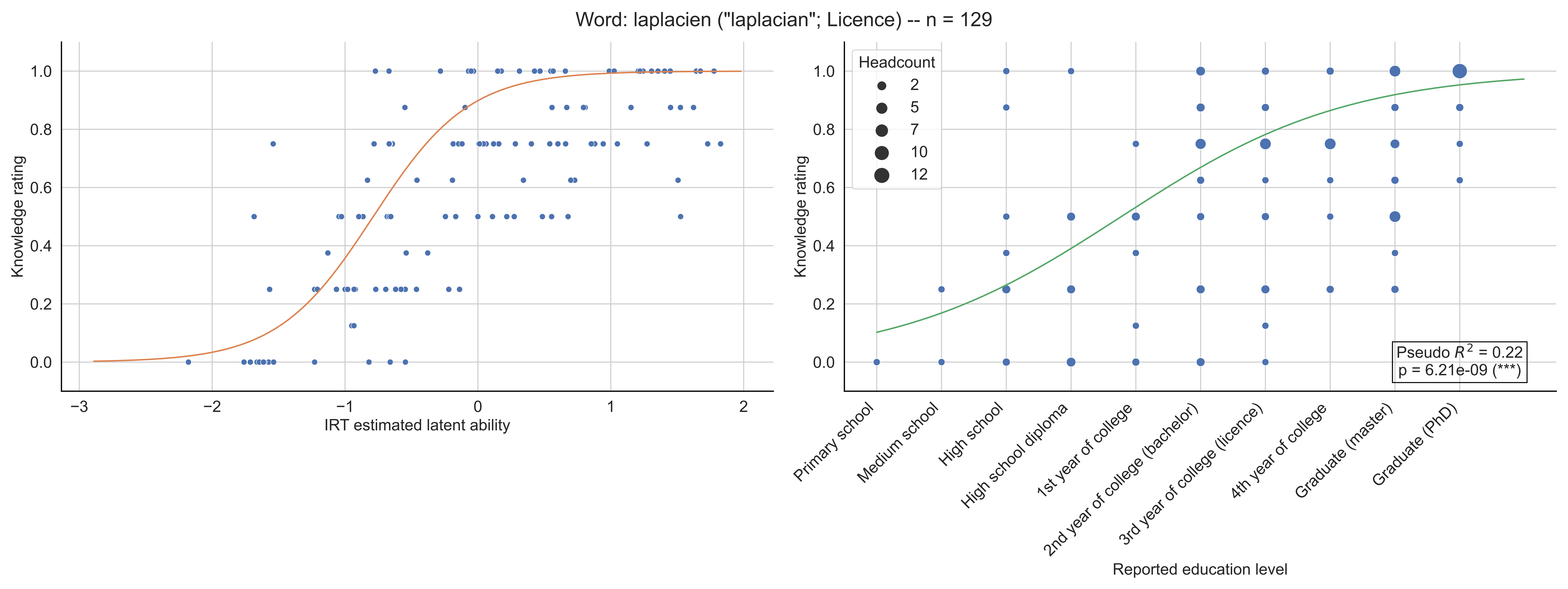

Word: laplacien ("laplacian"; Licence) -- n = 129
Knowledge rating
IRT estimated latent ability
Headcount
2
5
7
10
12
Pseudo R^2 = 0.22
p = 6.21e-09 (***)
Primary school
Medium school
High school
High school diploma
1st year of college
2nd year of college (bachelor)
3rd year of college (licence)
4th year of college
Graduate (master)
Graduate (PhD)
Reported education level

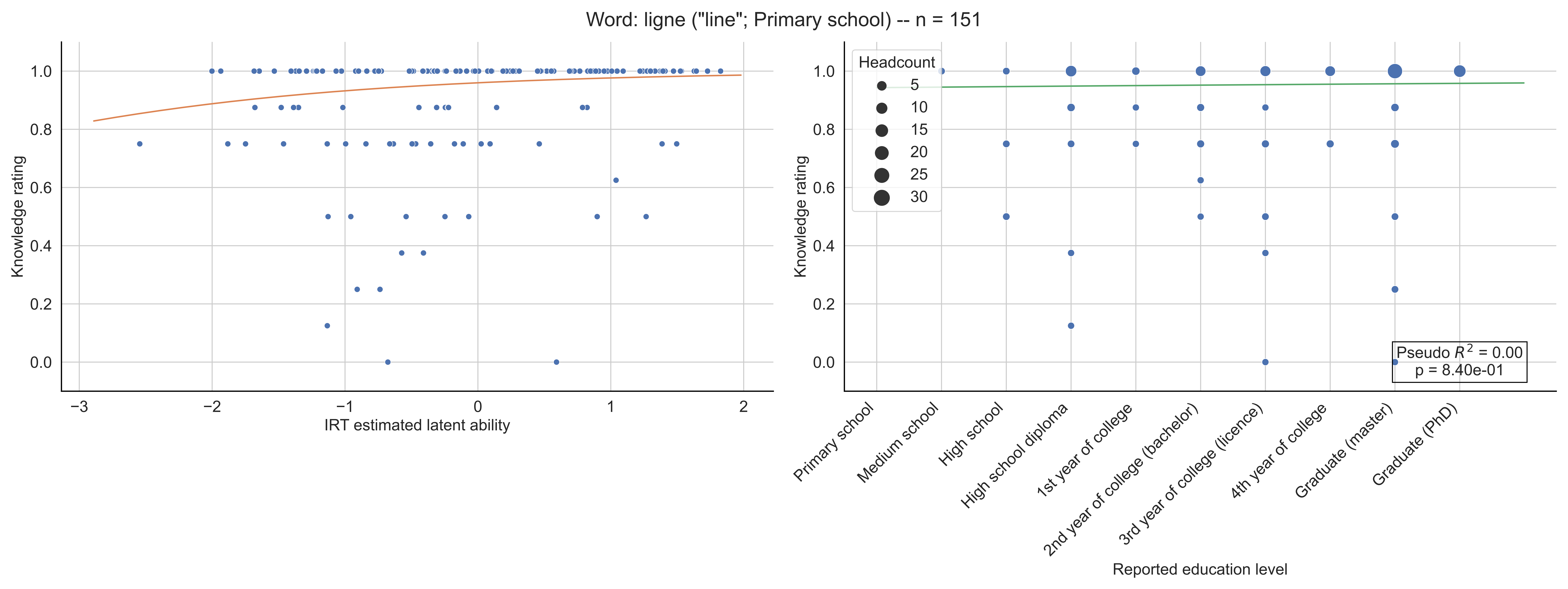
Word: ligne ("line"; Primary school) -- n = 151
Knowledge rating
IRT estimated latent ability
Headcount
5
10
15
20
25
30
Pseudo R^2 = 0.00
p = 8.40e-01
Primary school
Medium school
High school
High school diploma
1st year of college
2nd year of college (bachelor)
3rd year of college (licence)
4th year of college
Graduate (master)
Graduate (PhD)
Reported education level

Word: linéarité ("linearity"; 6-7th grade) -- n = 168

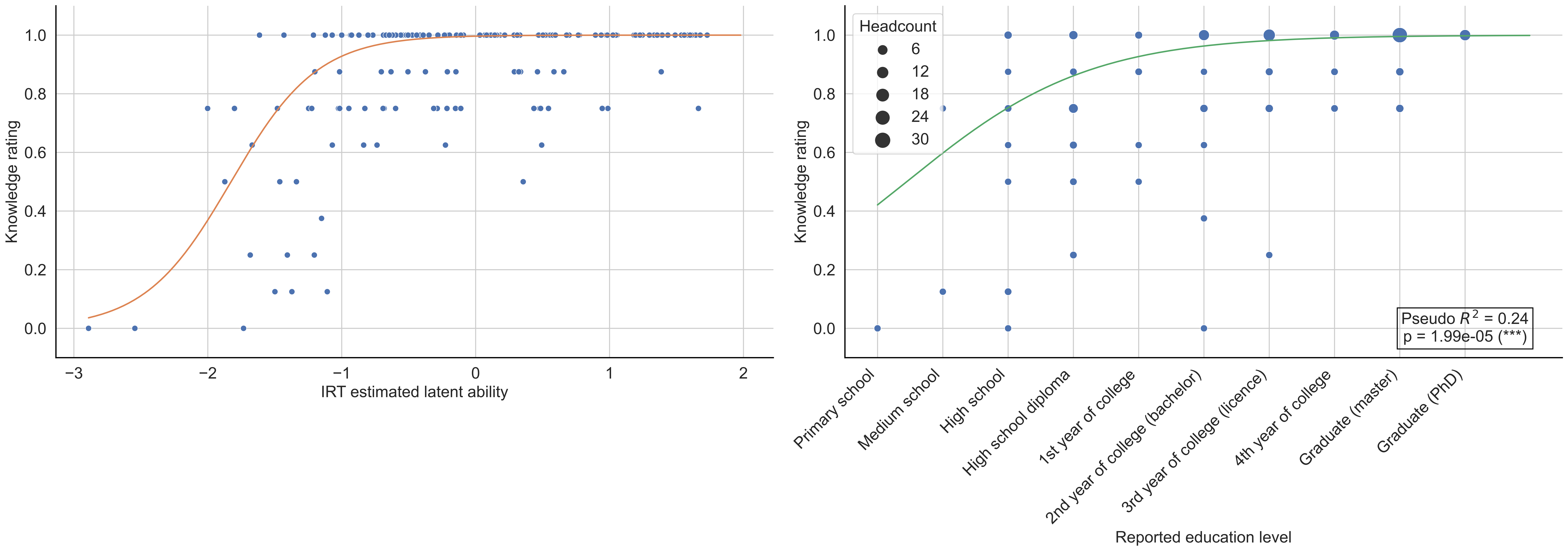

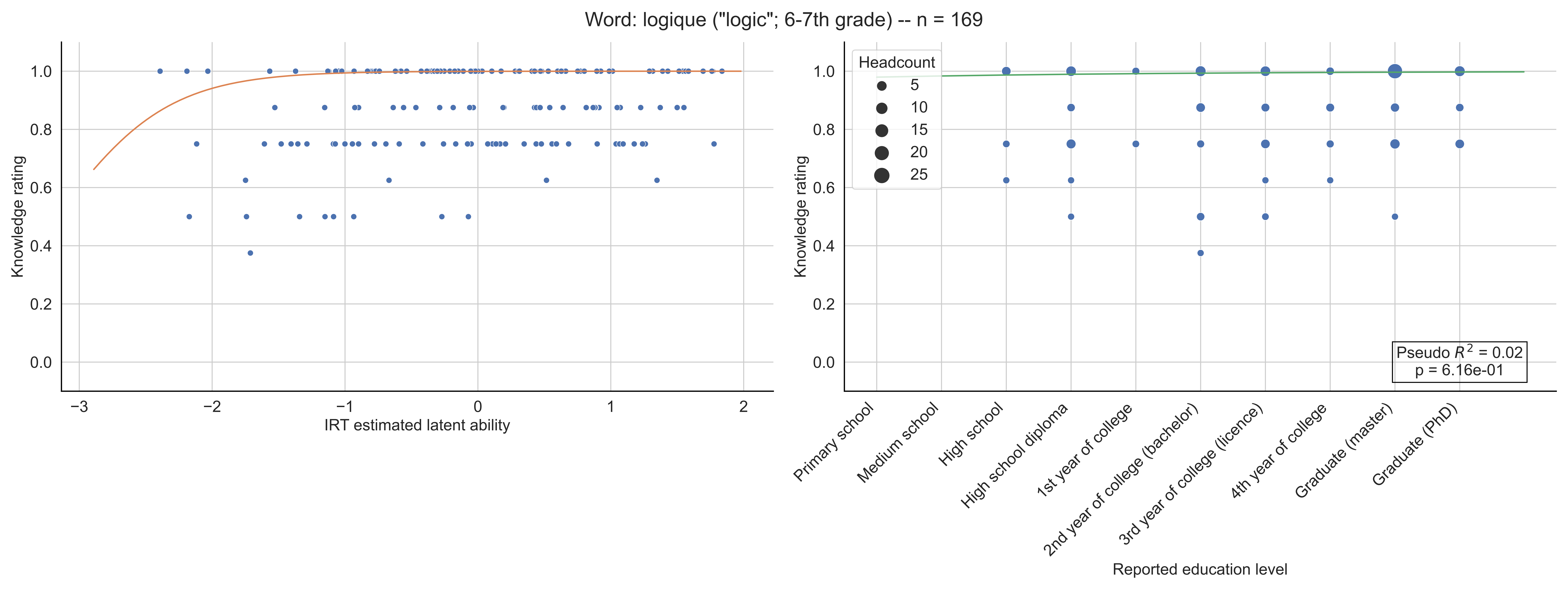
Word: logique ("logic"; 6-7th grade) -- n = 169
Knowledge rating
IRT estimated latent ability
Headcount
5
10
15
20
25
Pseudo R^2 = 0.02
p = 6.16e-01
Primary school
Medium school
High school
High school diploma
1st year of college
2nd year of college (bachelor)
3rd year of college (licence)
4th year of college
Graduate (master)
Graduate (PhD)
Reported education level

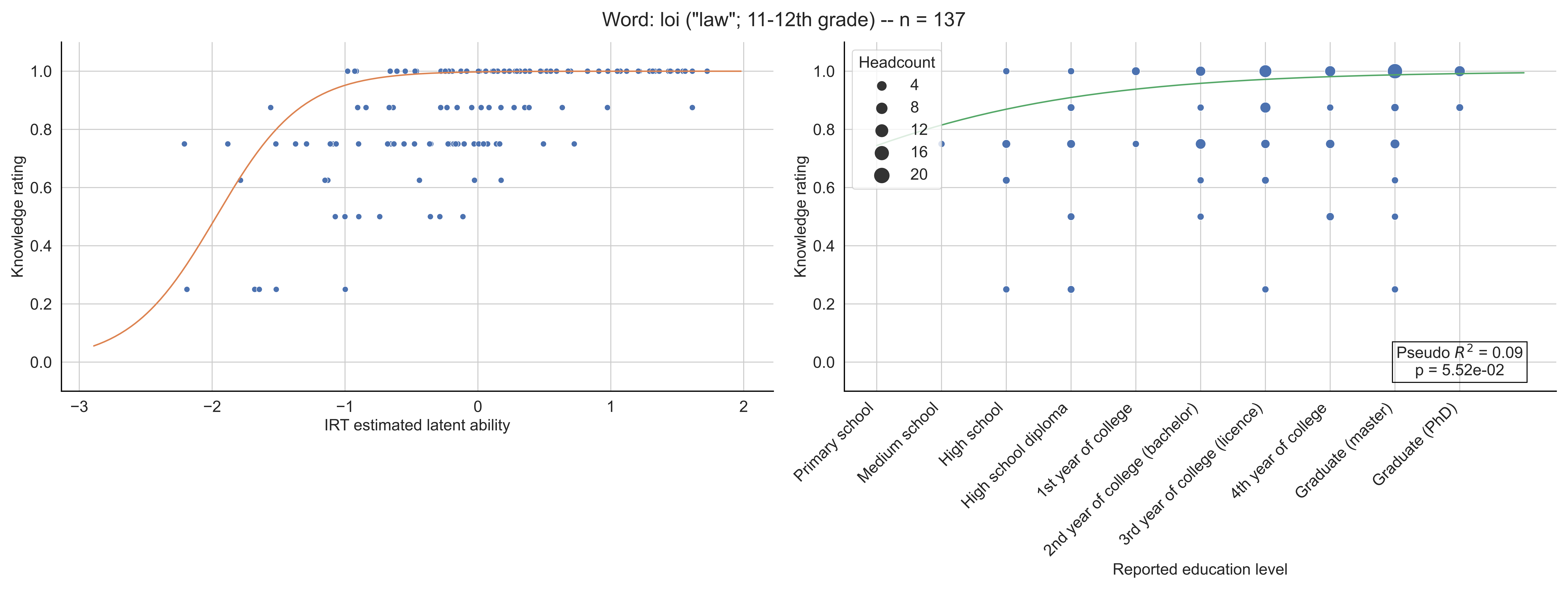

Word: loi ("law"; 11-12th grade) -- n = 137
Knowledge rating
IRT estimated latent ability
Headcount
4
8
12
16
20
Pseudo R^2 = 0.09
p = 5.52e-02
Primary school
Medium school
High school
High school diploma
1st year of college
2nd year of college (bachelor)
3rd year of college (licence)
4th year of college
Graduate (master)
Graduate (PhD)
Reported education level

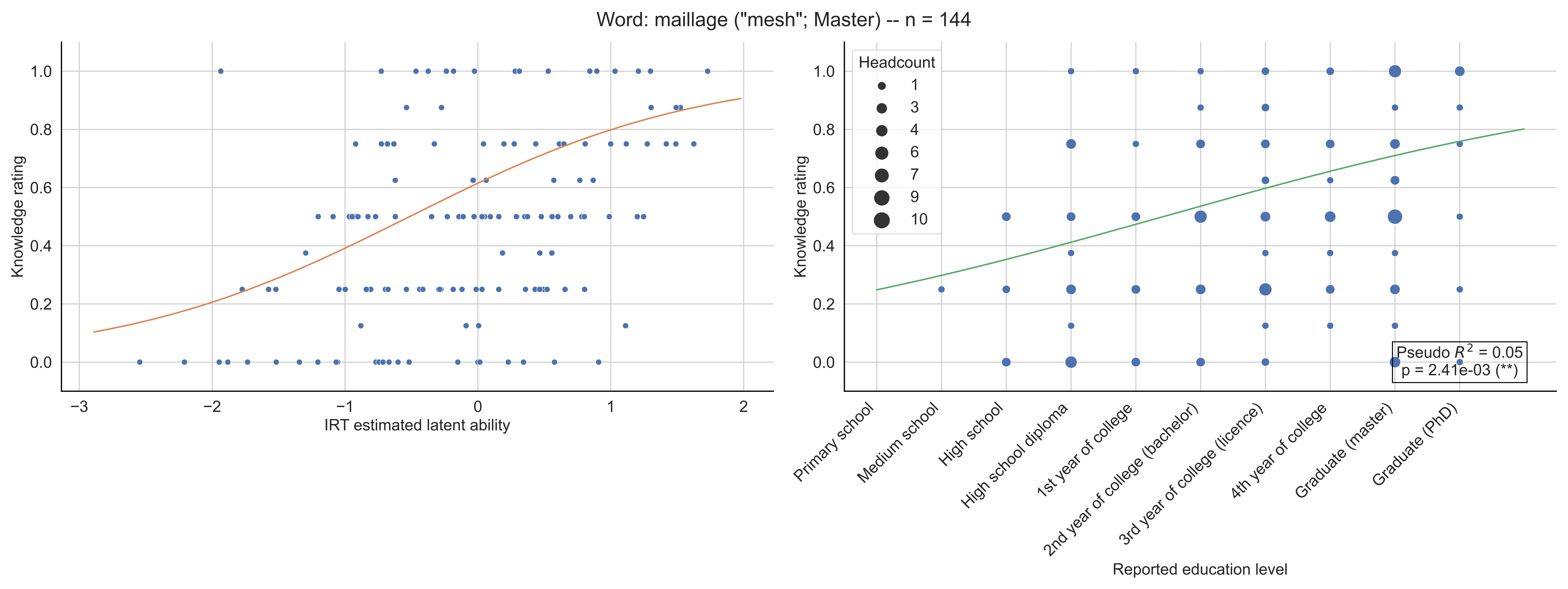
Word: maillage ("mesh"; Master) -- n = 144
Knowledge rating
IRT estimated latent ability
Headcount
1
3
4
6
7
9
10
Pseudo R^2 = 0.05
p = 2.41e-03 (**)
Primary school
Medium school
High school
High school diploma
1st year of college
2nd year of college (bachelor)
3rd year of college (licence)
4th year of college
Graduate (master)
Graduate (PhD)
Reported education level

Word: majoration (11-12th grade) -- n = 139

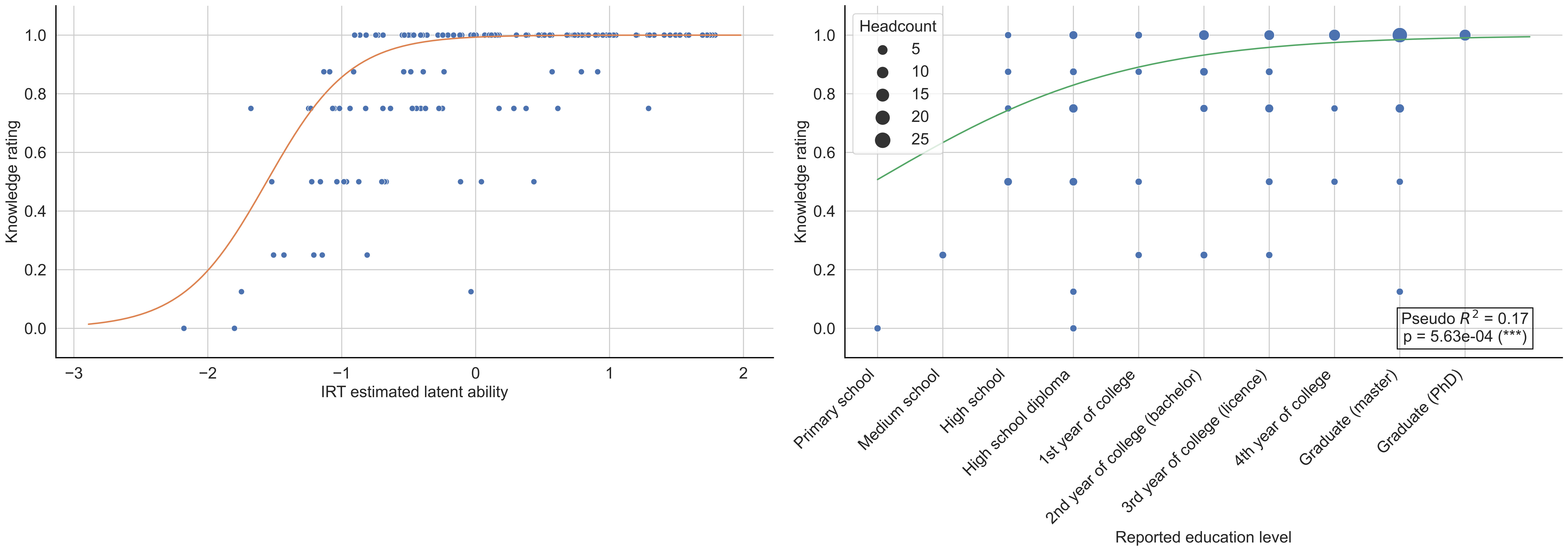

Word: martingale ("martingale"; Licence) -- n = 136

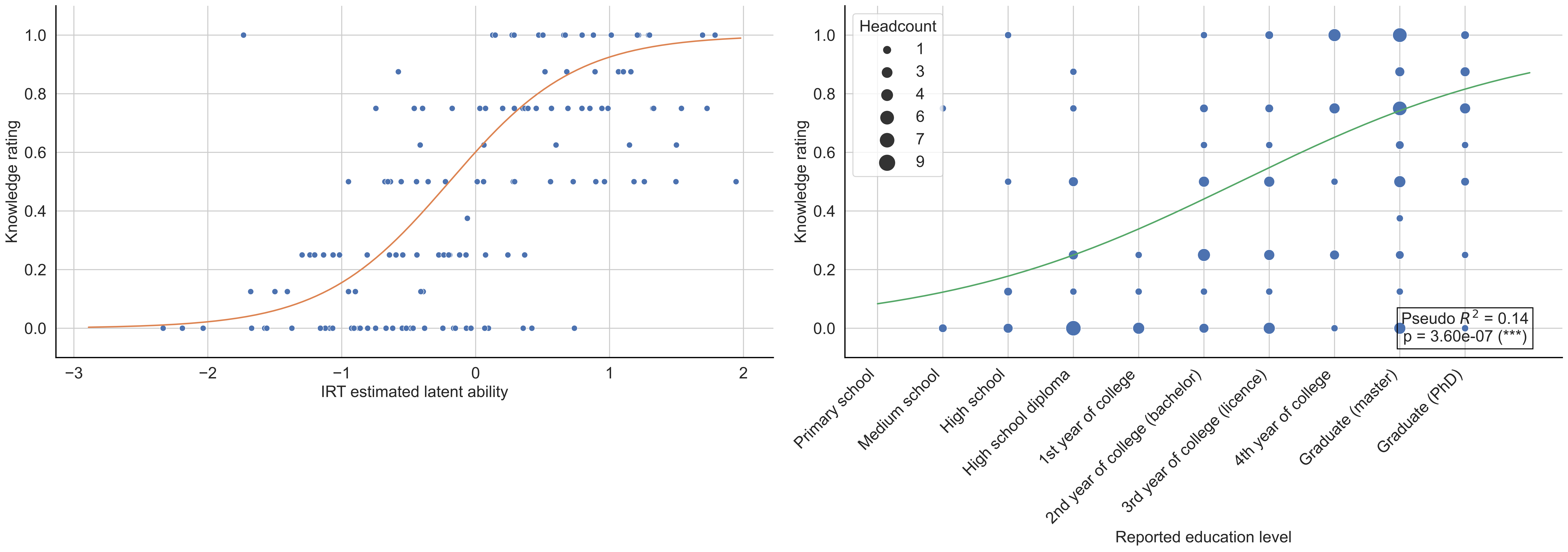

Word: matrice ("matrix"; 11-12th grade) -- n = 148

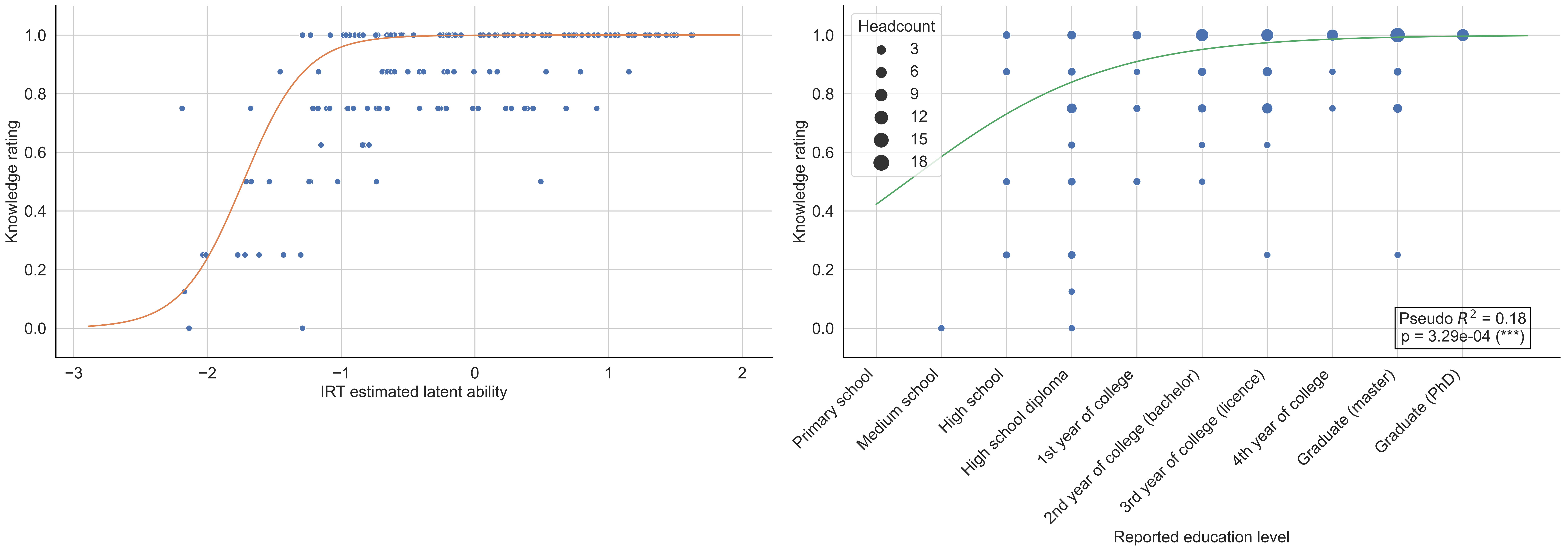

Word: mesure ("measure"; Primary school) -- n = 148

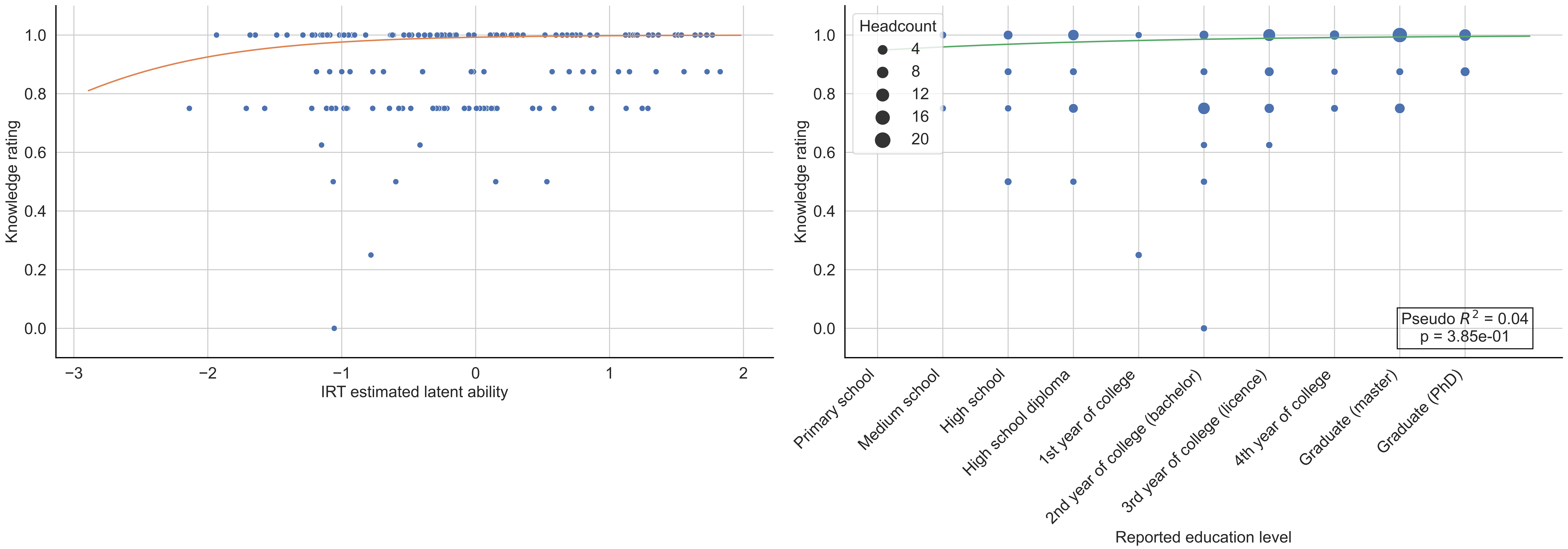

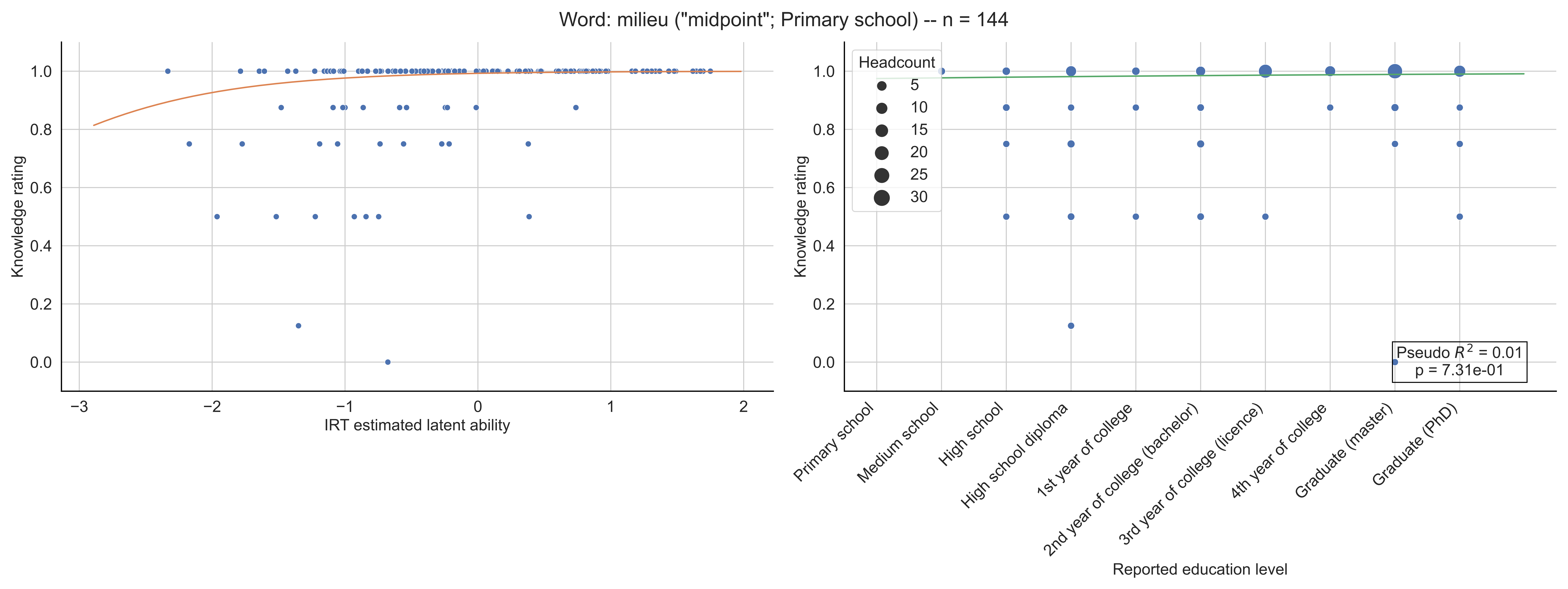
Word: milieu ("midpoint"; Primary school) -- n = 144
Knowledge rating
1.0
0.8
0.6
0.4
0.2
0.0
−3
−2
−1
0
1
2
IRT estimated latent ability
Headcount
5
10
15
20
25
30
Pseudo R^2 = 0.01
p = 7.31e-01
Primary school
Medium school
High school
High school diploma
1st year of college
2nd year of college (bachelor)
3rd year of college (licence)
4th year of college
Graduate (master)
Graduate (PhD)
Reported education level

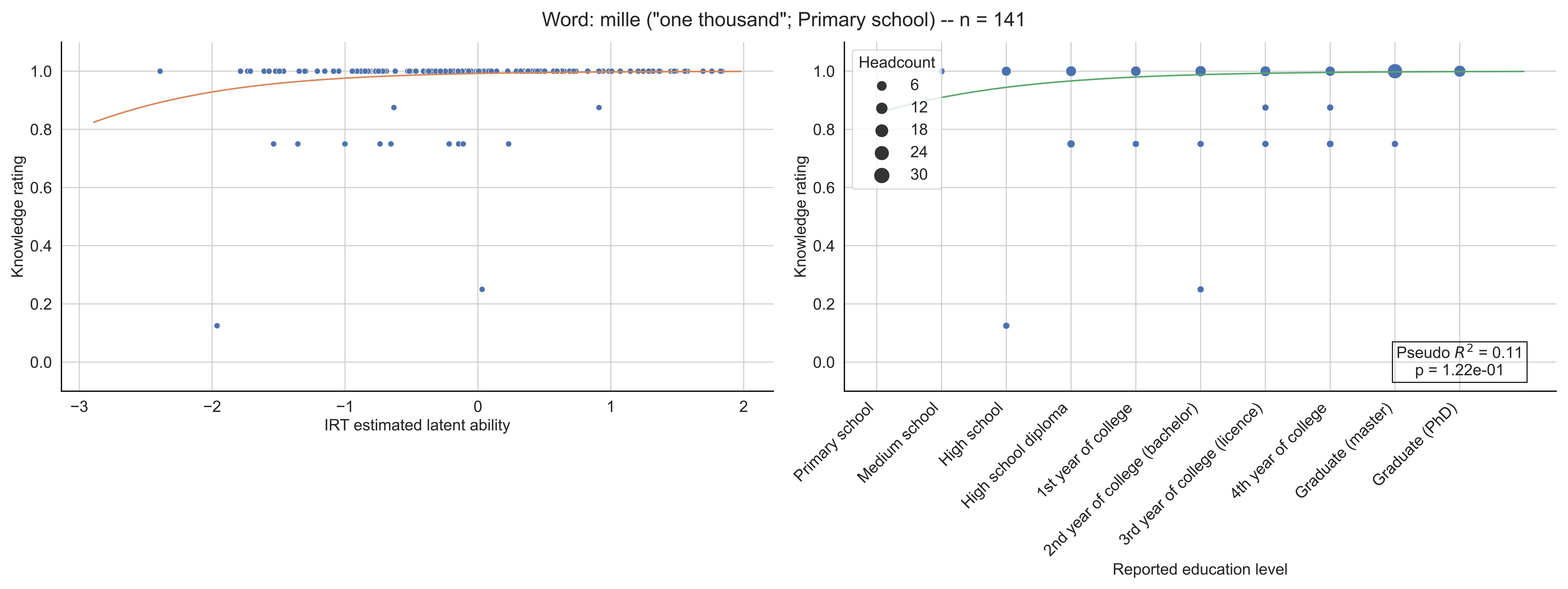

Word: mille ("one thousand"; Primary school) -- n = 141
Knowledge rating
IRT estimated latent ability
Headcount
6
12
18
24
30
Pseudo R^2 = 0.11
p = 1.22e-01
Primary school
Medium school
High school
High school diploma
1st year of college
2nd year of college (bachelor)
3rd year of college (licence)
4th year of college
Graduate (master)
Graduate (PhD)
Reported education level

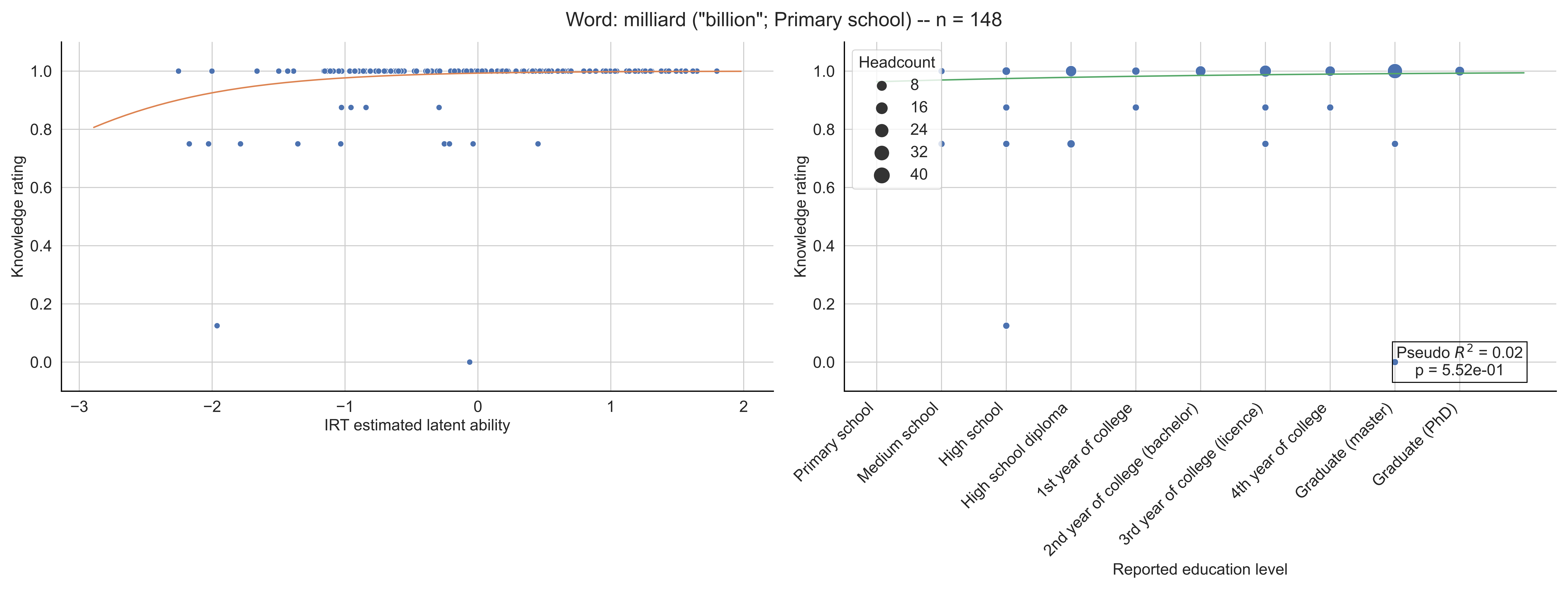
Word: milliard ("billion"; Primary school) -- n = 148
Knowledge rating
IRT estimated latent ability
Headcount
8
16
24
32
40
Primary school
Medium school
High school
High school diploma
1st year of college
2nd year of college (bachelor)
3rd year of college (licence)
4th year of college
Graduate (master)
Graduate (PhD)
Reported education level
Pseudo R^2 = 0.02
p = 5.52e-01

Word: minimum ("minimum"; Primary school) -- n = 139

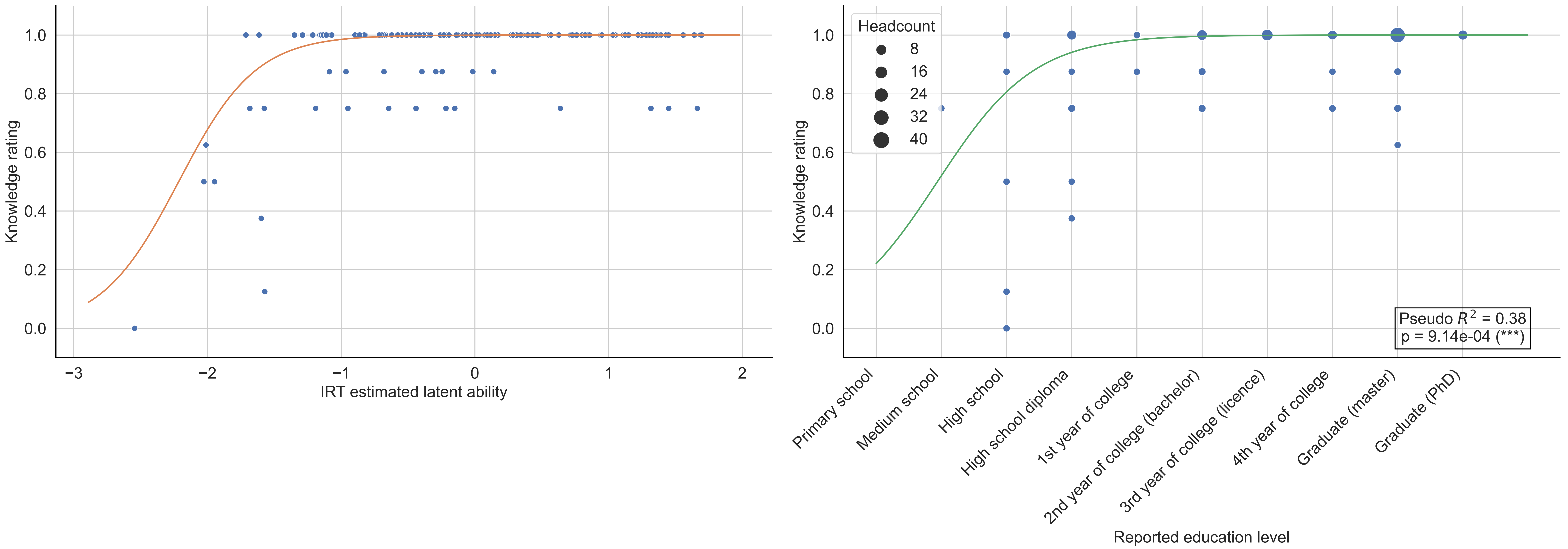

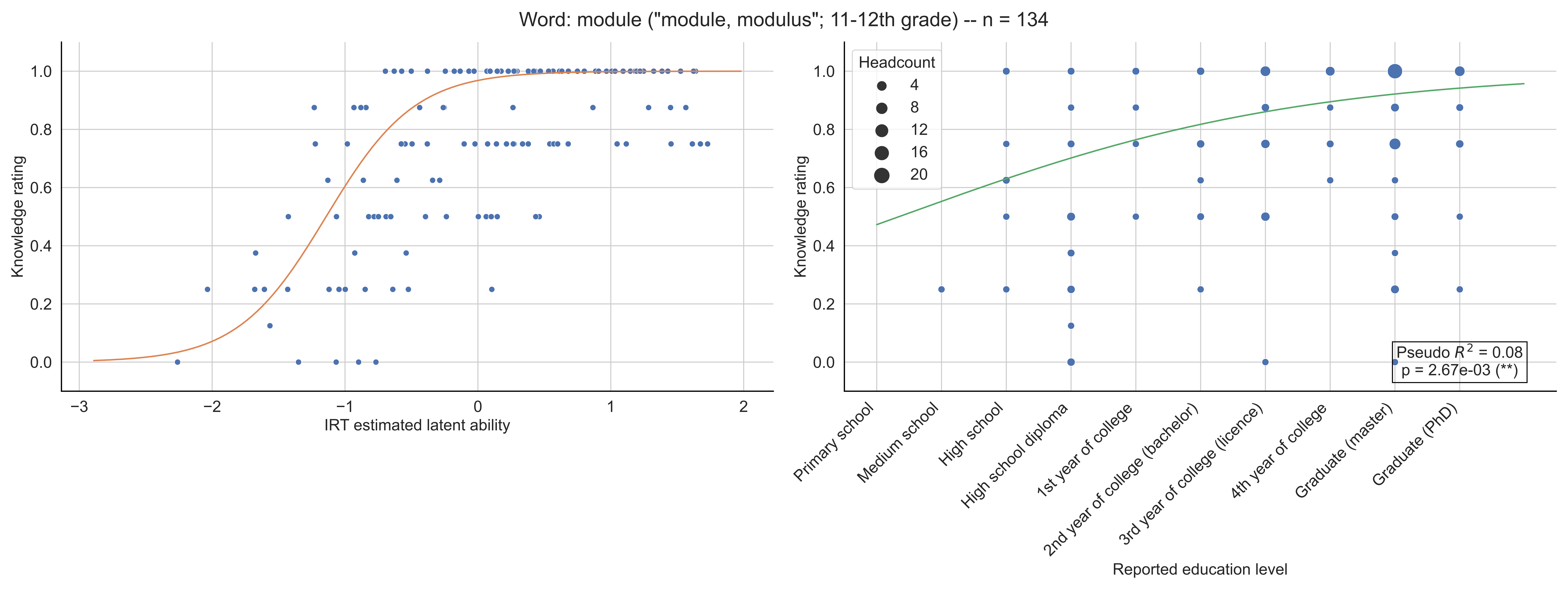
Word: module ("module, modulus"; 11-12th grade) -- n = 134
Knowledge rating
IRT estimated latent ability
Headcount
4
8
12
16
20
Pseudo R^2 = 0.08
p = 2.67e-03 (**)
Primary school
Medium school
High school
High school diploma
1st year of college
2nd year of college (bachelor)
3rd year of college (licence)
4th year of college
Graduate (master)
Graduate (PhD)
Reported education level

Word: modulo ("modulo"; 11-12th grade) -- n = 141

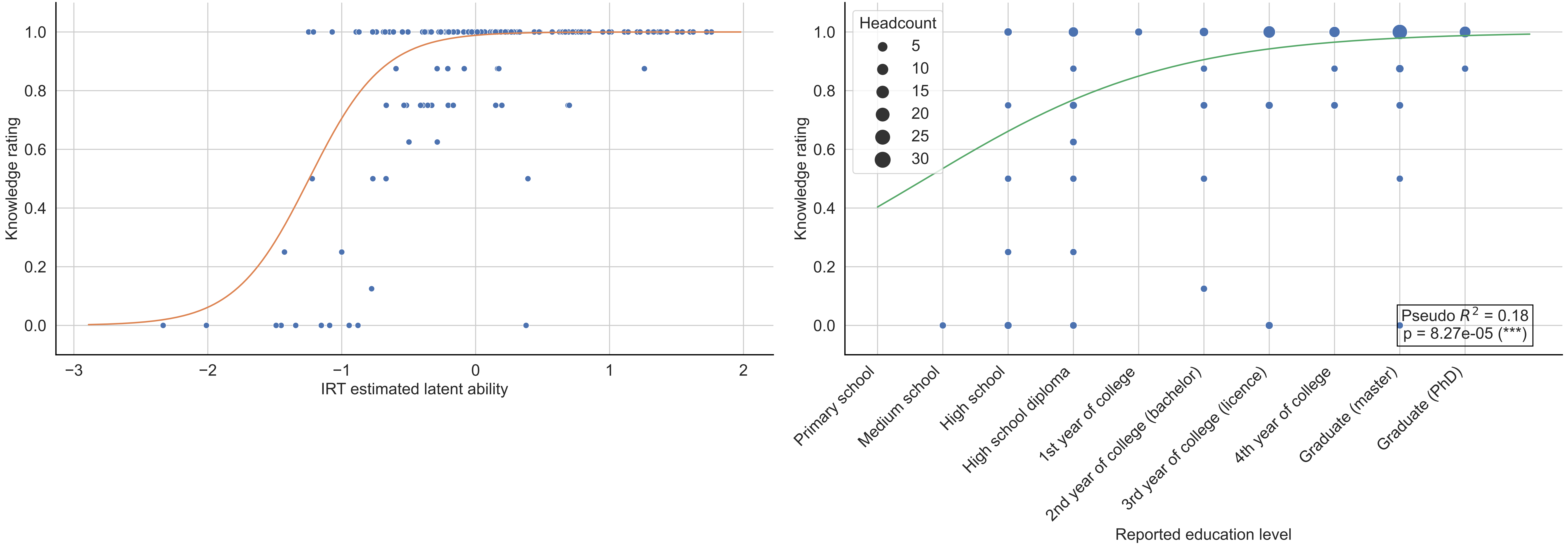

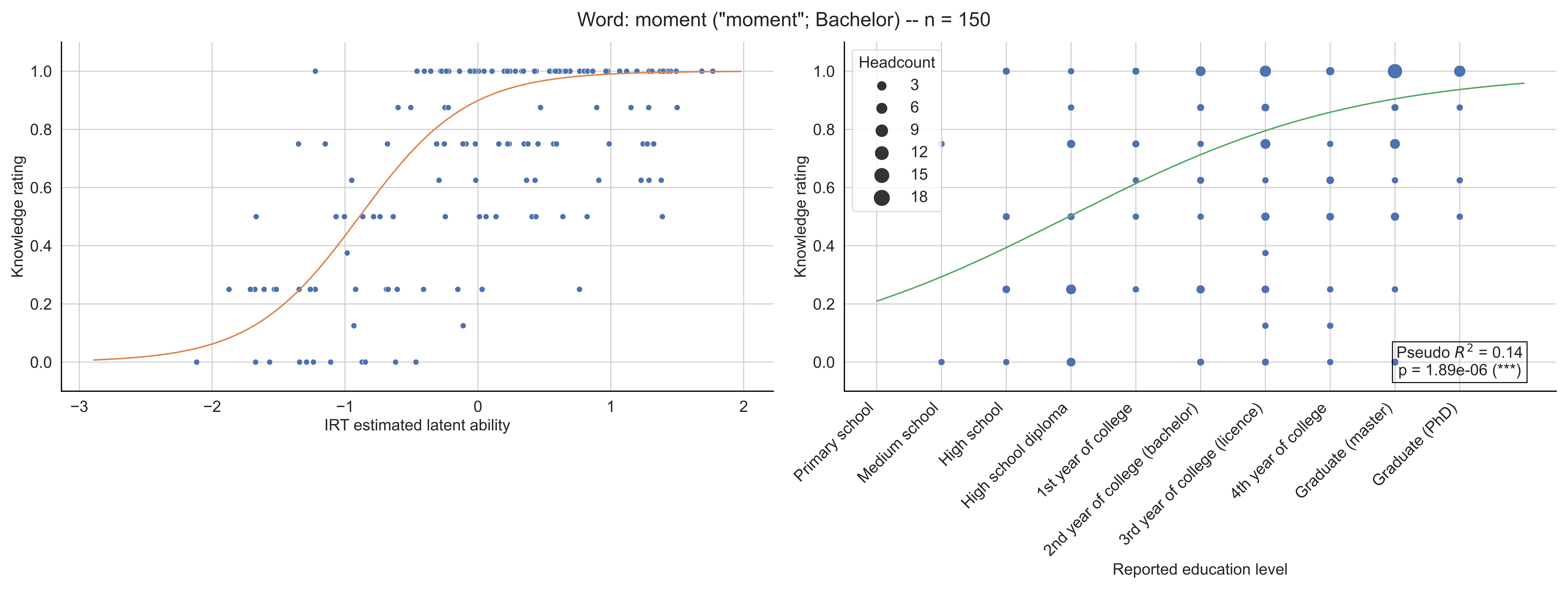

Word: moment ("moment"; Bachelor) -- n = 150
Knowledge rating
IRT estimated latent ability
Headcount
3
6
9
12
15
18
Pseudo R^2 = 0.14
p = 1.89e-06 (***)
Primary school
Medium school
High school
High school diploma
1st year of college
2nd year of college (bachelor)
3rd year of college (licence)
4th year of college
Graduate (master)
Graduate (PhD)
Reported education level

Word: monotonie ("monotonicity"; 10th grade) -- n = 141

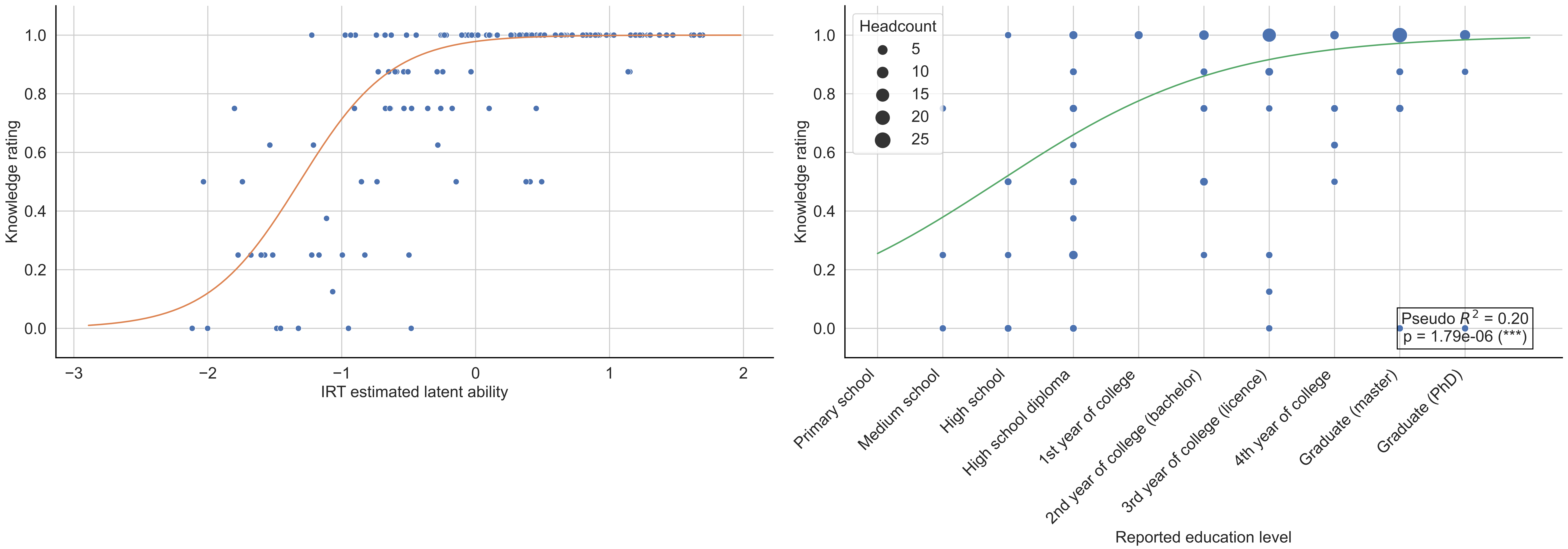

Word: monoïde ("monoid"; Bachelor) -- n = 151

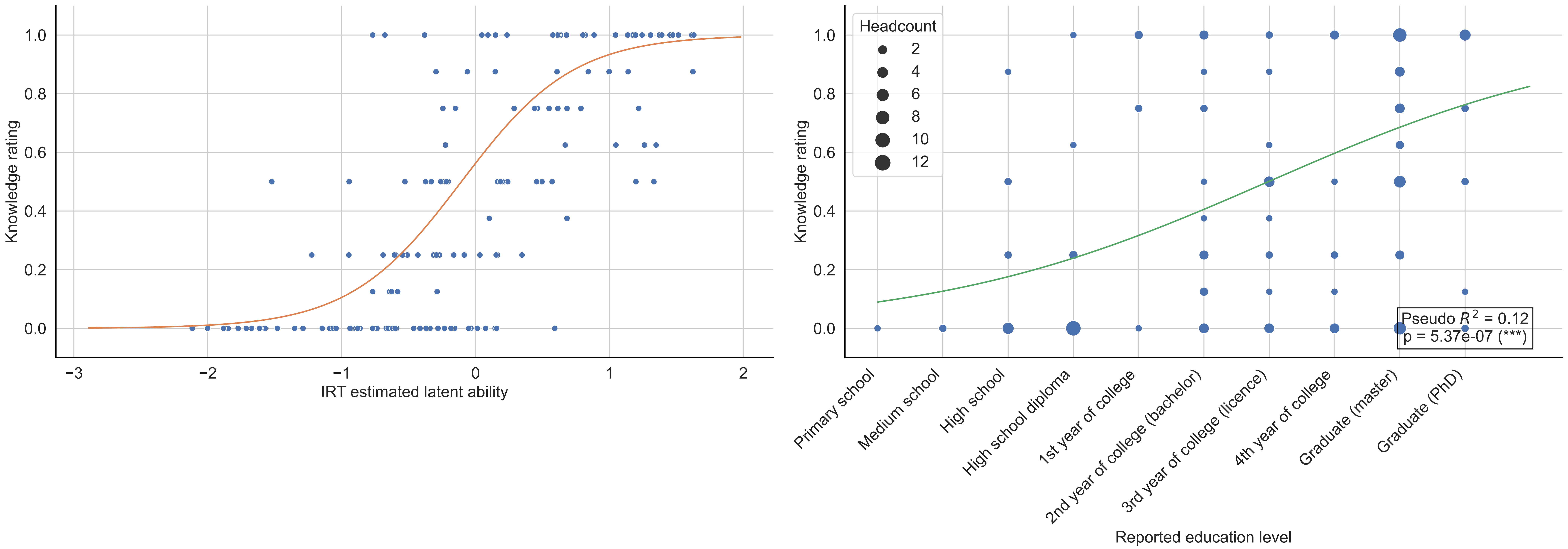

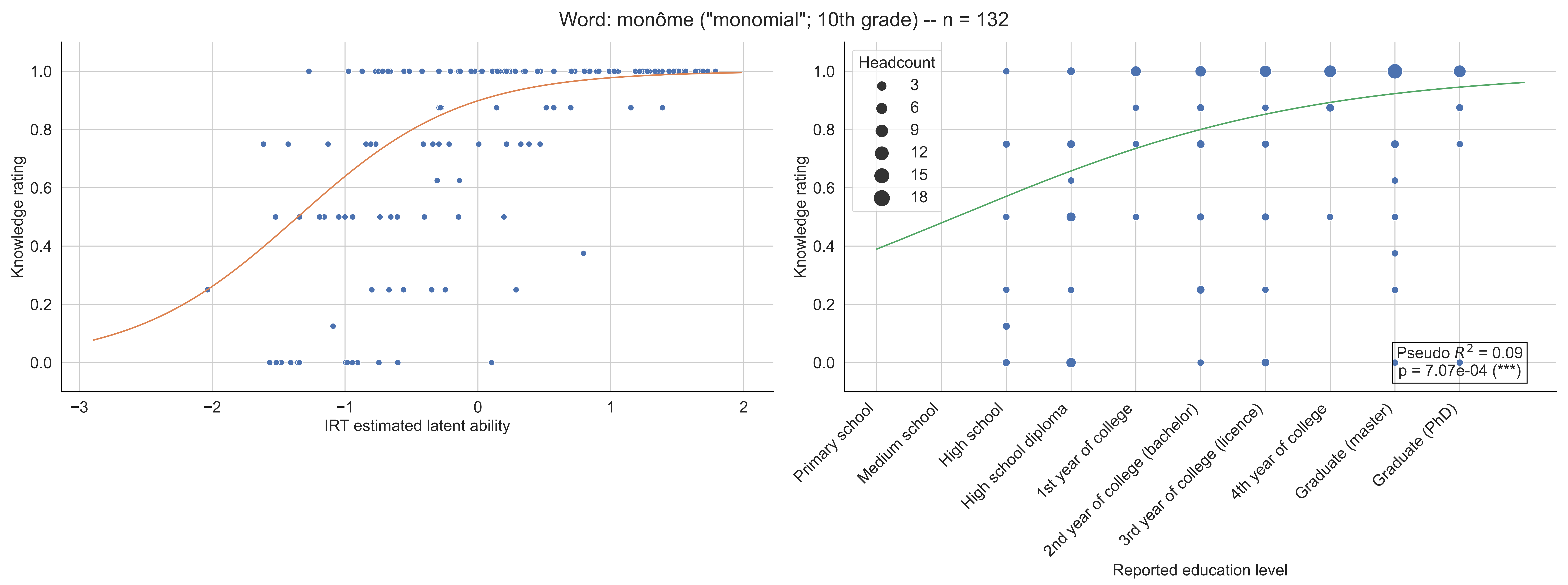

Word: monôme ("monomial"; 10th grade) -- n = 132
Knowledge rating
IRT estimated latent ability
Headcount
3
6
9
12
15
18
Pseudo R^2 = 0.09
p = 7.07e-04 (***)
Primary school
Medium school
High school
High school diploma
1st year of college
2nd year of college (bachelor)
3rd year of college (licence)
4th year of college
Graduate (master)
Graduate (PhD)
Reported education level

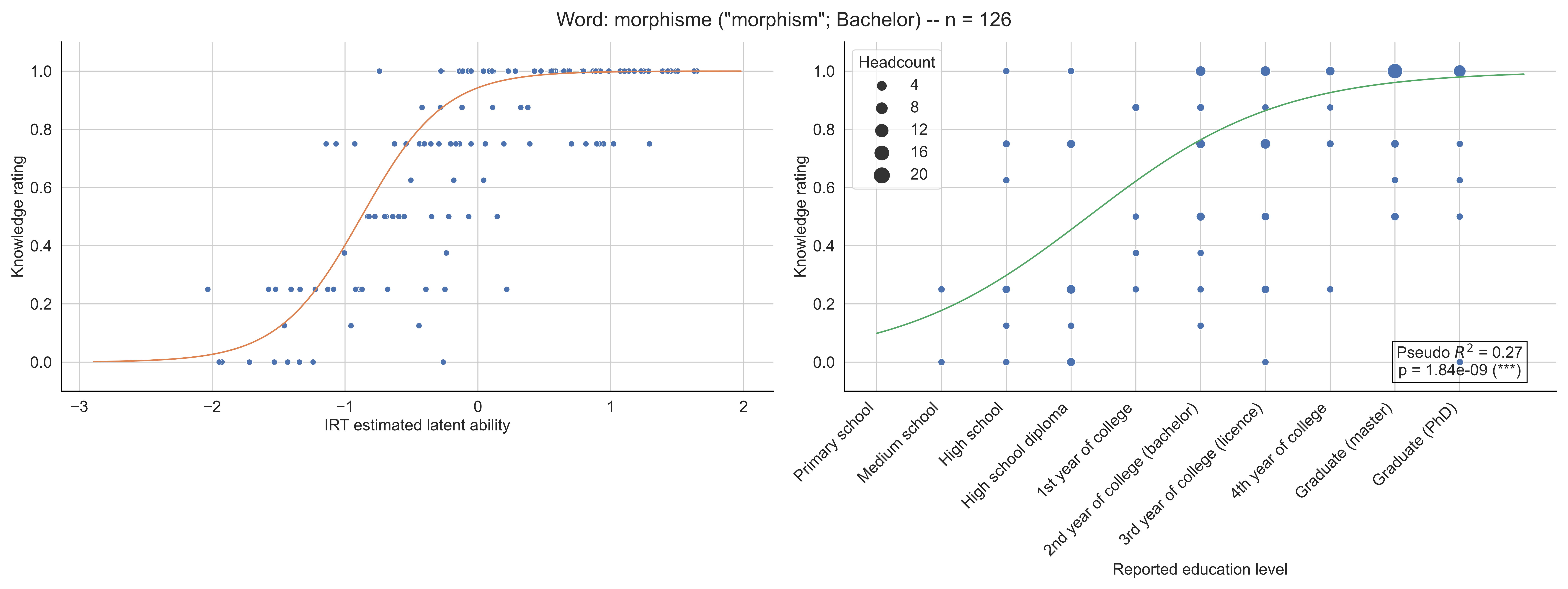

Word: morphisme ("morphism"; Bachelor) -- n = 126
Knowledge rating
IRT estimated latent ability
−3
−2
−1
0
1
2
0.0
0.2
0.4
0.6
0.8
1.0
Headcount
4
8
12
16
20
Pseudo R^2 = 0.27
p = 1.84e-09 (***)
Primary school
Medium school
High school
High school diploma
1st year of college
2nd year of college (bachelor)
3rd year of college (licence)
4th year of college
Graduate (master)
Graduate (PhD)
Reported education level

Word: médiatrice ("perpendicular bisector"; Primary school) -- n = 134

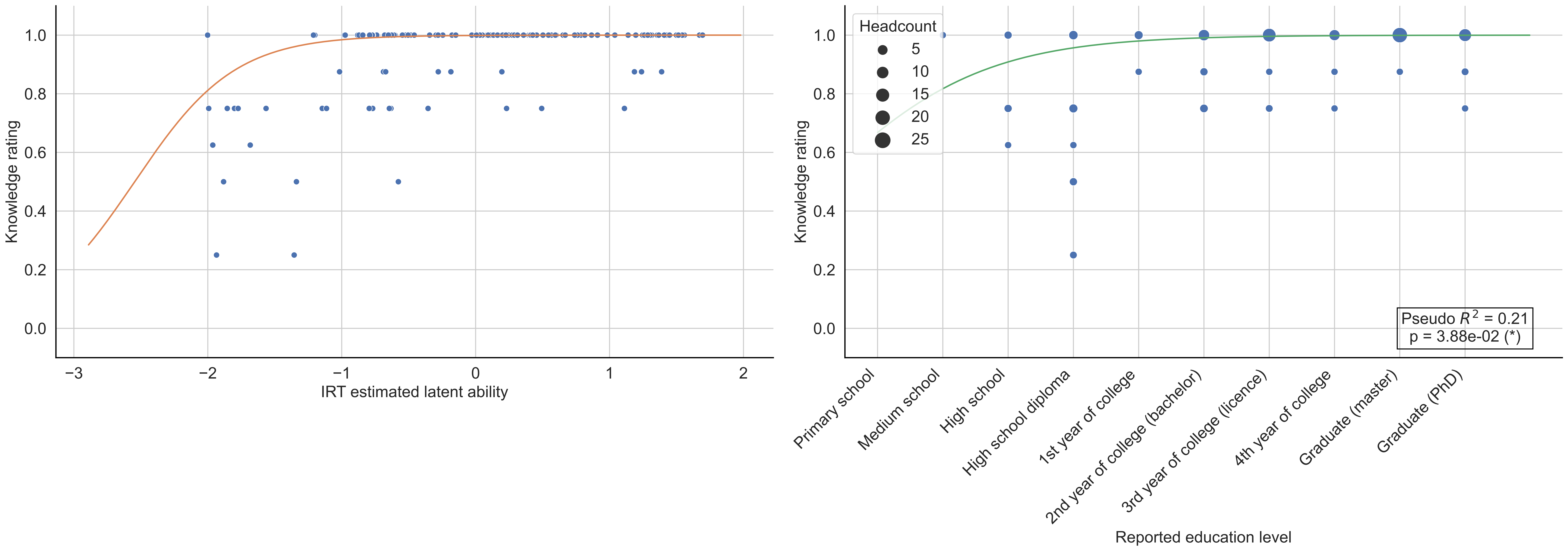

Word: nappe ("nappe"; Licence) -- n = 148

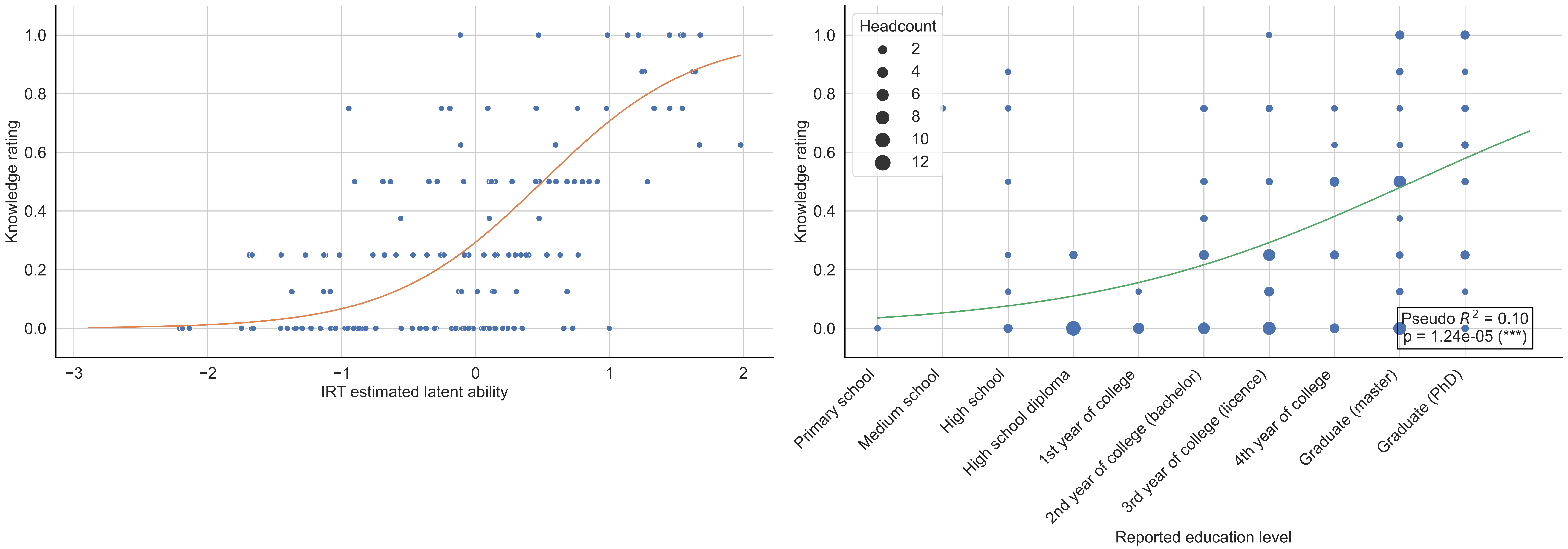

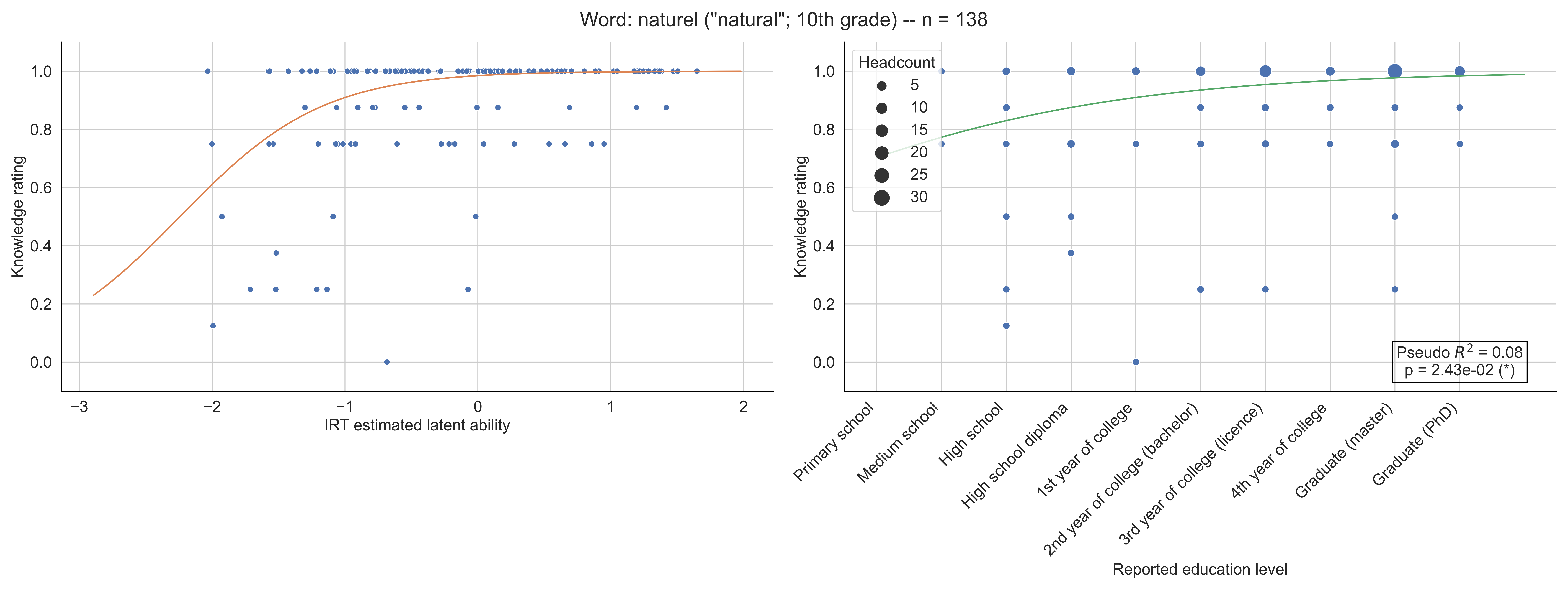

Word: naturel ("natural"; 10th grade) -- n = 138
Knowledge rating
IRT estimated latent ability
Headcount
5
10
15
20
25
30
Pseudo R^2 = 0.08
p = 2.43e-02 (*)
Primary school
Medium school
High school
High school diploma
1st year of college
2nd year of college (bachelor)
3rd year of college (licence)
4th year of college
Graduate (master)
Graduate (PhD)
Reported education level

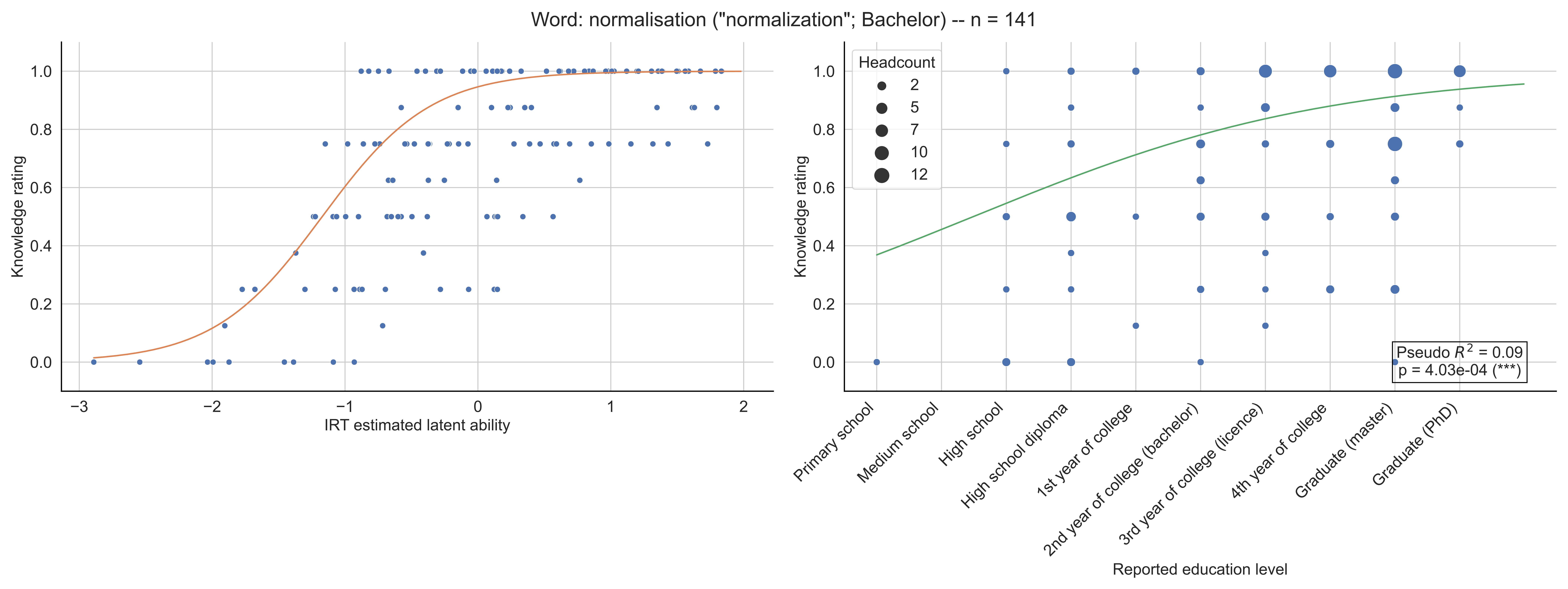

Word: normalisation ("normalization"; Bachelor) -- n = 141
Knowledge rating
IRT estimated latent ability
Headcount
2
5
7
10
12
Pseudo R^2 = 0.09
p = 4.03e-04 (***)
Primary school
Medium school
High school
High school diploma
1st year of college
2nd year of college (bachelor)
3rd year of college (licence)
4th year of college
Graduate (master)
Graduate (PhD)
Reported education level

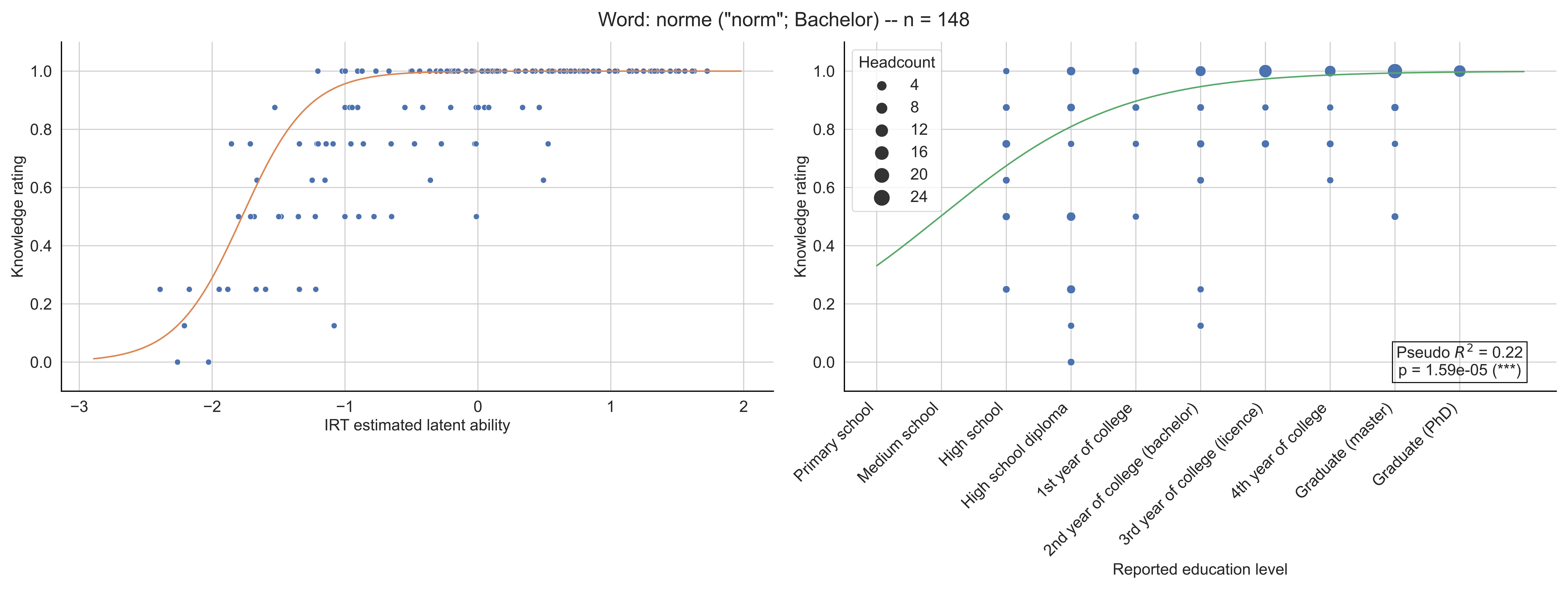
Word: norme ("norm"; Bachelor) -- n = 148
Knowledge rating
IRT estimated latent ability
−3
−2
−1
0
1
2
0.0
0.2
0.4
0.6
0.8
1.0
Headcount
4
8
12
16
20
24
Primary school
Medium school
High school
High school diploma
1st year of college
2nd year of college (bachelor)
3rd year of college (licence)
4th year of college
Graduate (master)
Graduate (PhD)
Reported education level
Pseudo R^2 = 0.22
p = 1.59e-05 (***)

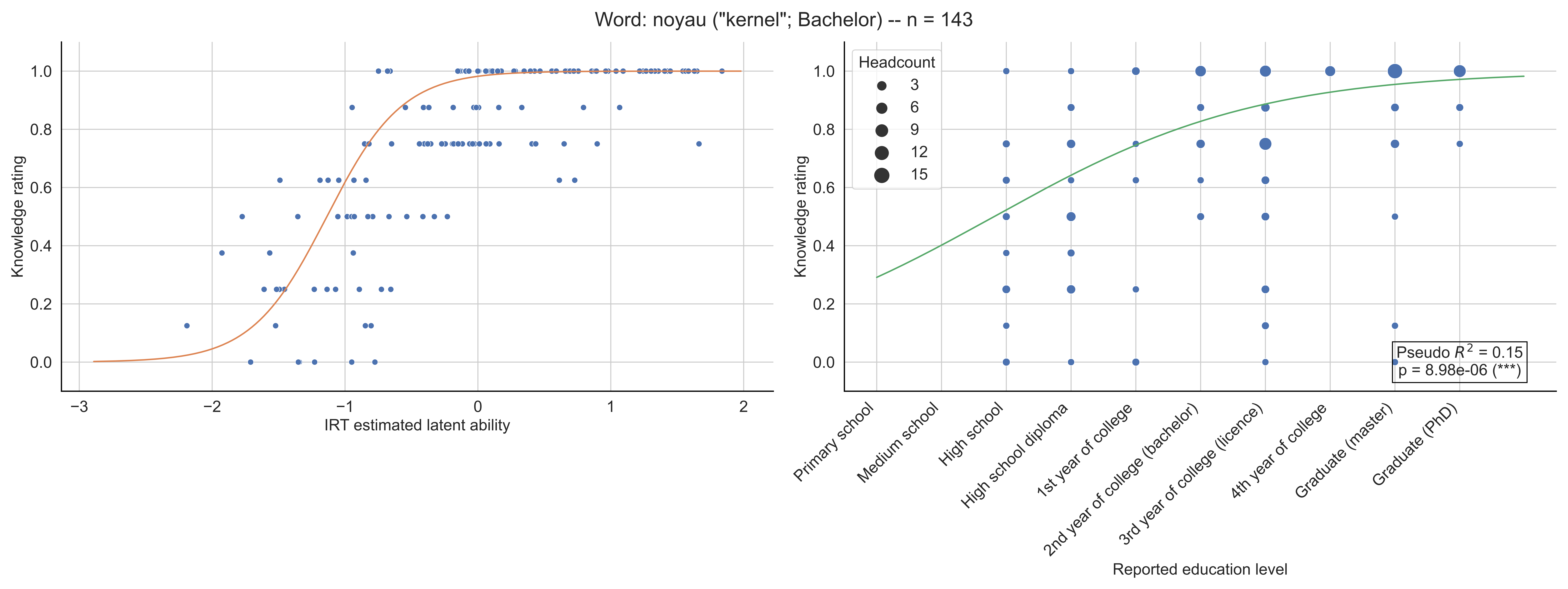

Word: noyau ("kernel"; Bachelor) -- n = 143
Knowledge rating
IRT estimated latent ability
Headcount
3
6
9
12
15
Pseudo R^2 = 0.15
p = 8.98e-06 (***)
Primary school
Medium school
High school
High school diploma
1st year of college
2nd year of college (bachelor)
3rd year of college (licence)
4th year of college
Graduate (master)
Graduate (PhD)
Reported education level

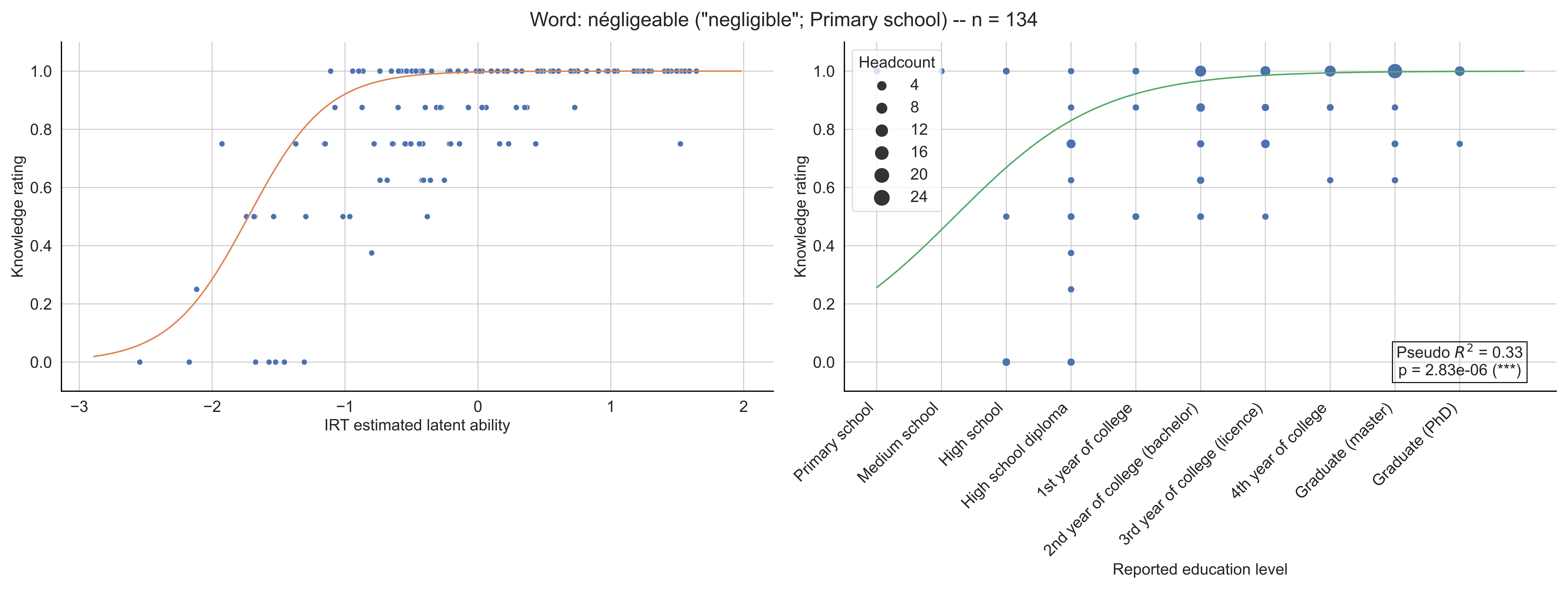

Word: négligeable ("negligible"; Primary school) -- n = 134
Knowledge rating
IRT estimated latent ability
Headcount
4
8
12
16
20
24
Pseudo R^2 = 0.33
p = 2.83e-06 (***)
Primary school
Medium school
High school
High school diploma
1st year of college
2nd year of college (bachelor)
3rd year of college (licence)
4th year of college
Graduate (master)
Graduate (PhD)
Reported education level

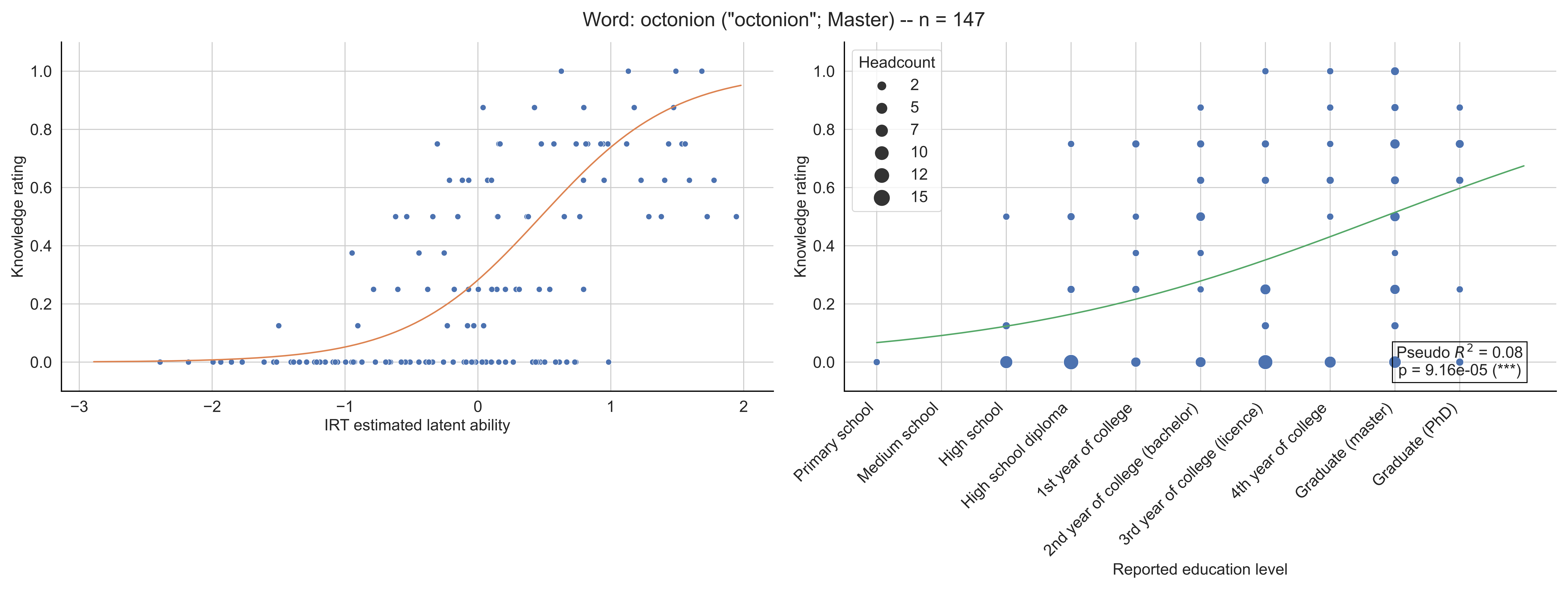

Word: octonion ("octonion"; Master) -- n = 147
Knowledge rating
IRT estimated latent ability
Headcount
2
5
7
10
12
15
Pseudo R^2 = 0.08
p = 9.16e-05 (***)
Primary school
Medium school
High school
High school diploma
1st year of college
2nd year of college (bachelor)
3rd year of college (licence)
4th year of college
Graduate (master)
Graduate (PhD)
Reported education level

Word: opposé ("opposite??"; 6-7th grade) -- n = 143

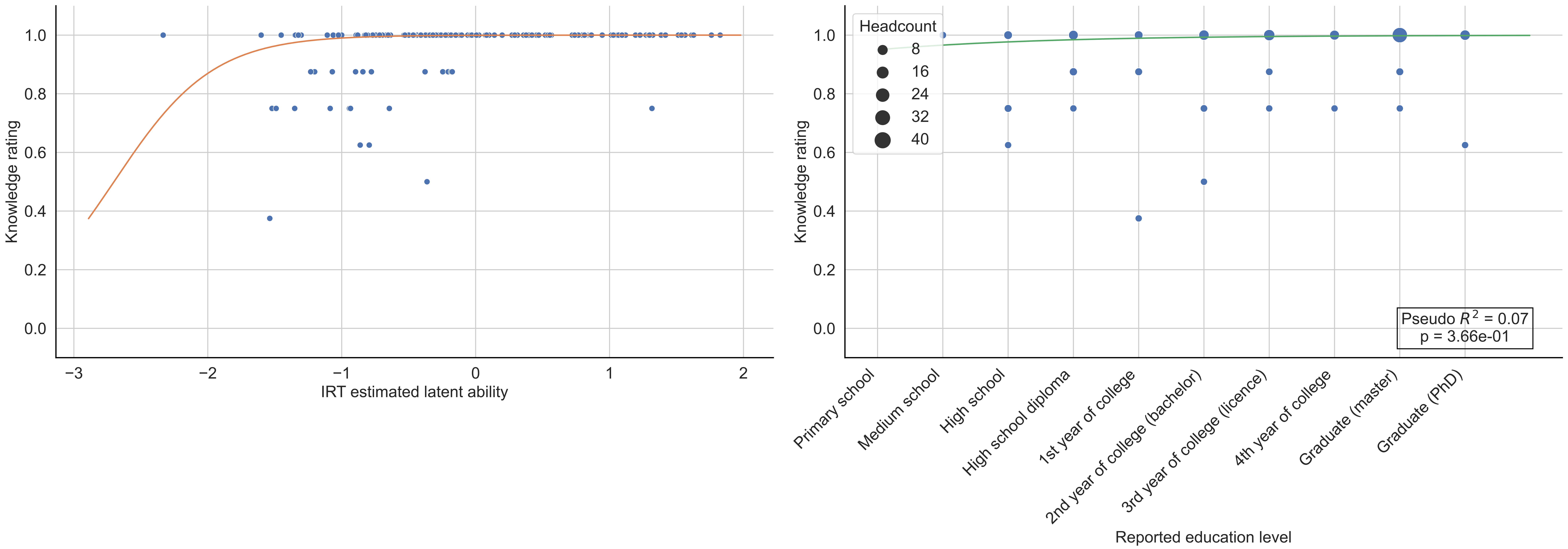

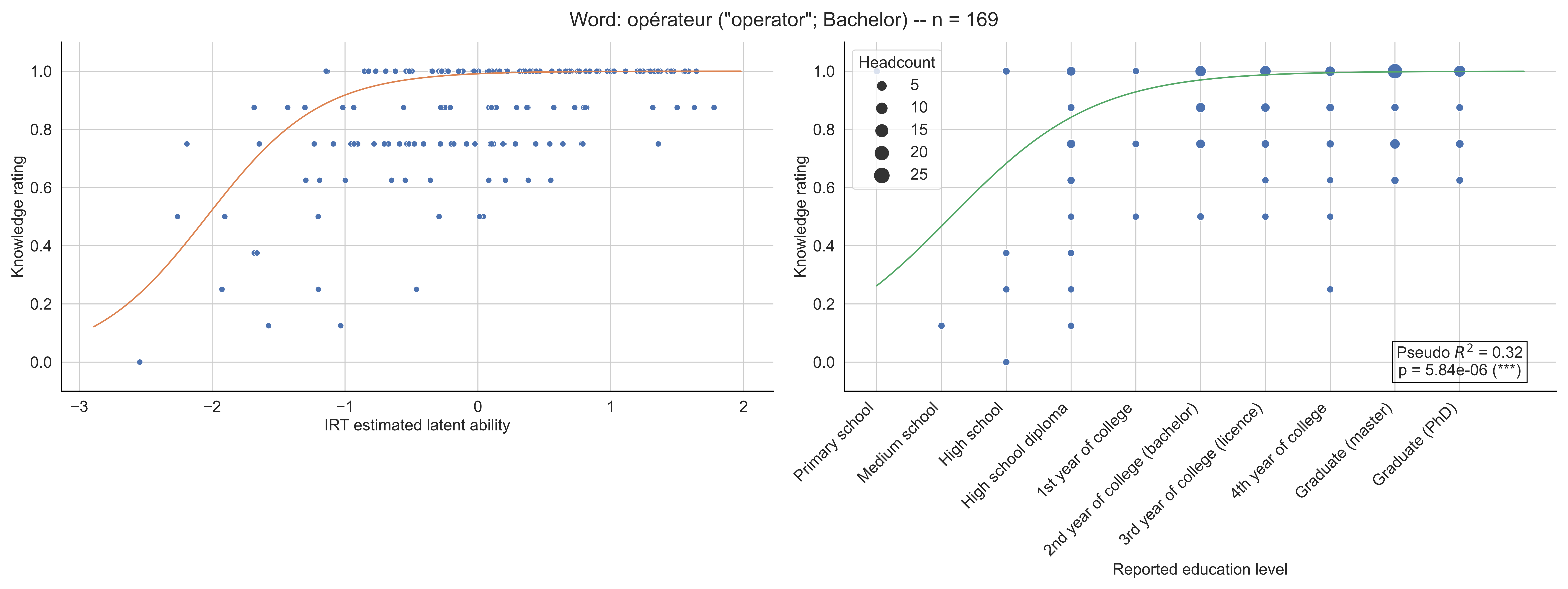

Word: opérateur ("operator"; Bachelor) -- n = 169
Knowledge rating
IRT estimated latent ability
Headcount
5
10
15
20
25
Pseudo R^2 = 0.32
p = 5.84e-06 (***)
Primary school
Medium school
High school
High school diploma
1st year of college
2nd year of college (bachelor)
3rd year of college (licence)
4th year of college
Graduate (master)
Graduate (PhD)
Reported education level

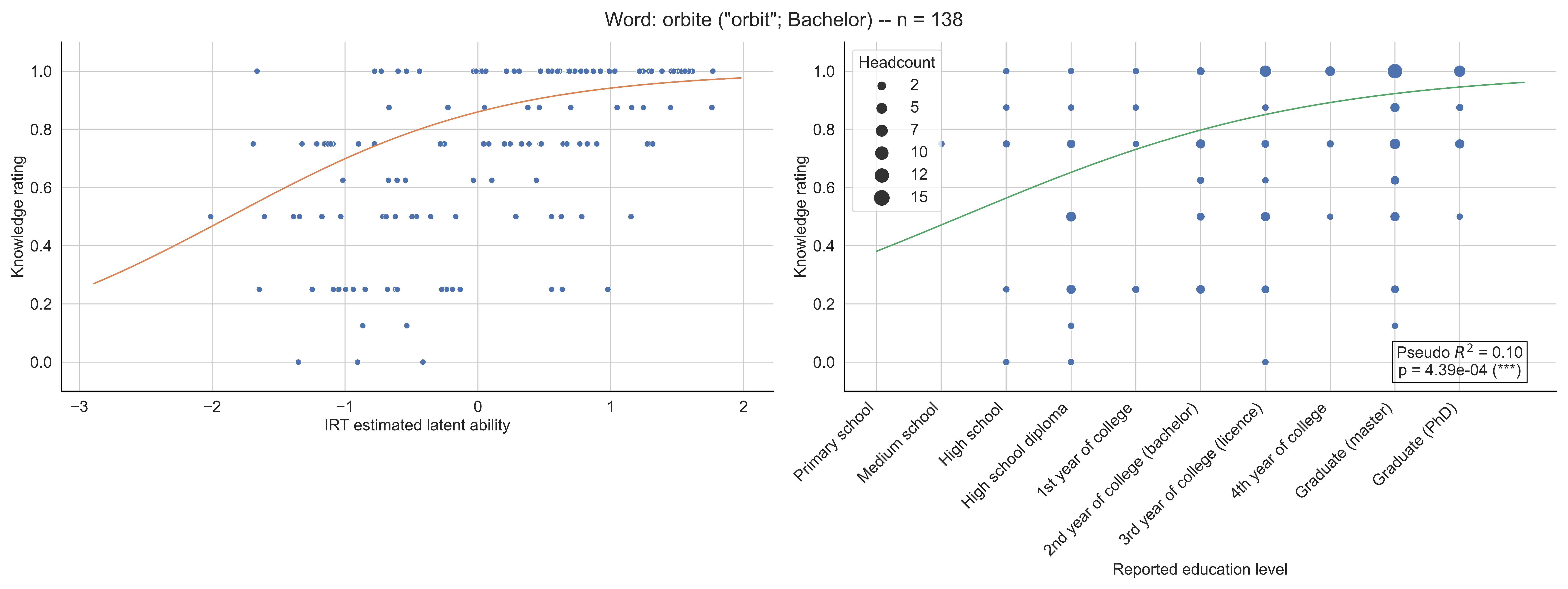

Word: orbite ("orbit"; Bachelor) -- n = 138
Knowledge rating
IRT estimated latent ability
Headcount
2
5
7
10
12
15
Pseudo R^2 = 0.10
p = 4.39e-04 (***)
Primary school
Medium school
High school
High school diploma
1st year of college
2nd year of college (bachelor)
3rd year of college (licence)
4th year of college
Graduate (master)
Graduate (PhD)
Reported education level

Word: ordre ("order"; Primary school) -- n = 151

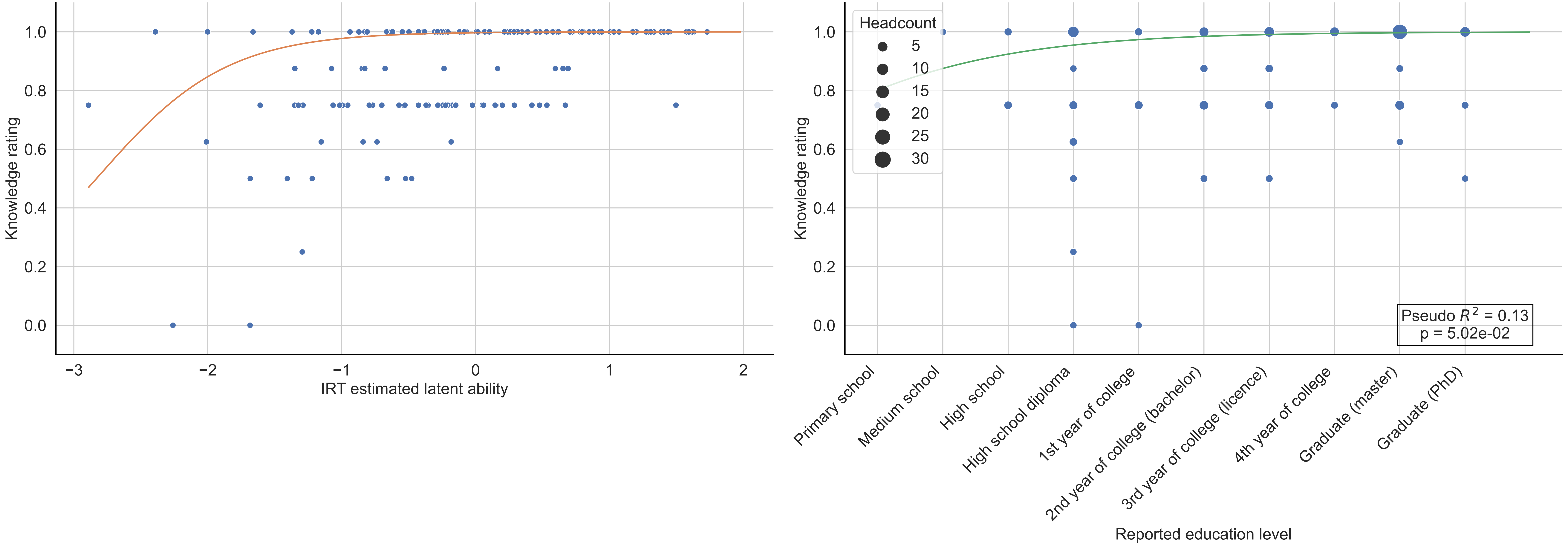

Word: origine ("origin"; 6-7th grade) -- n = 122

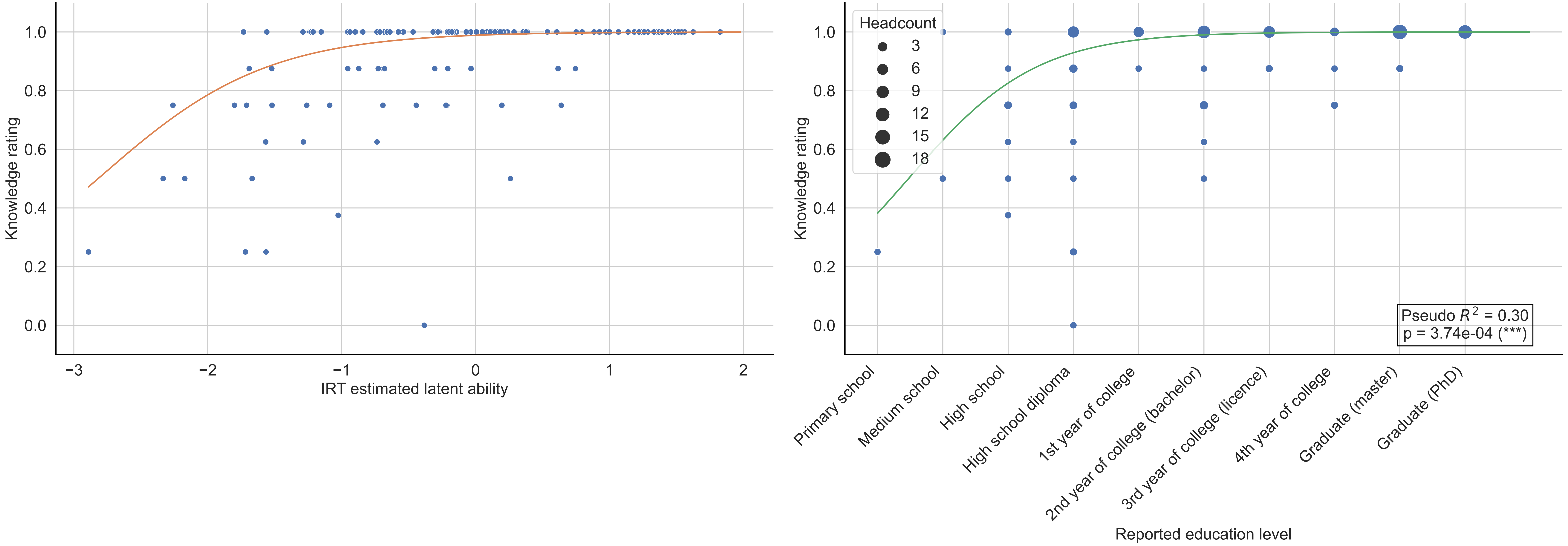

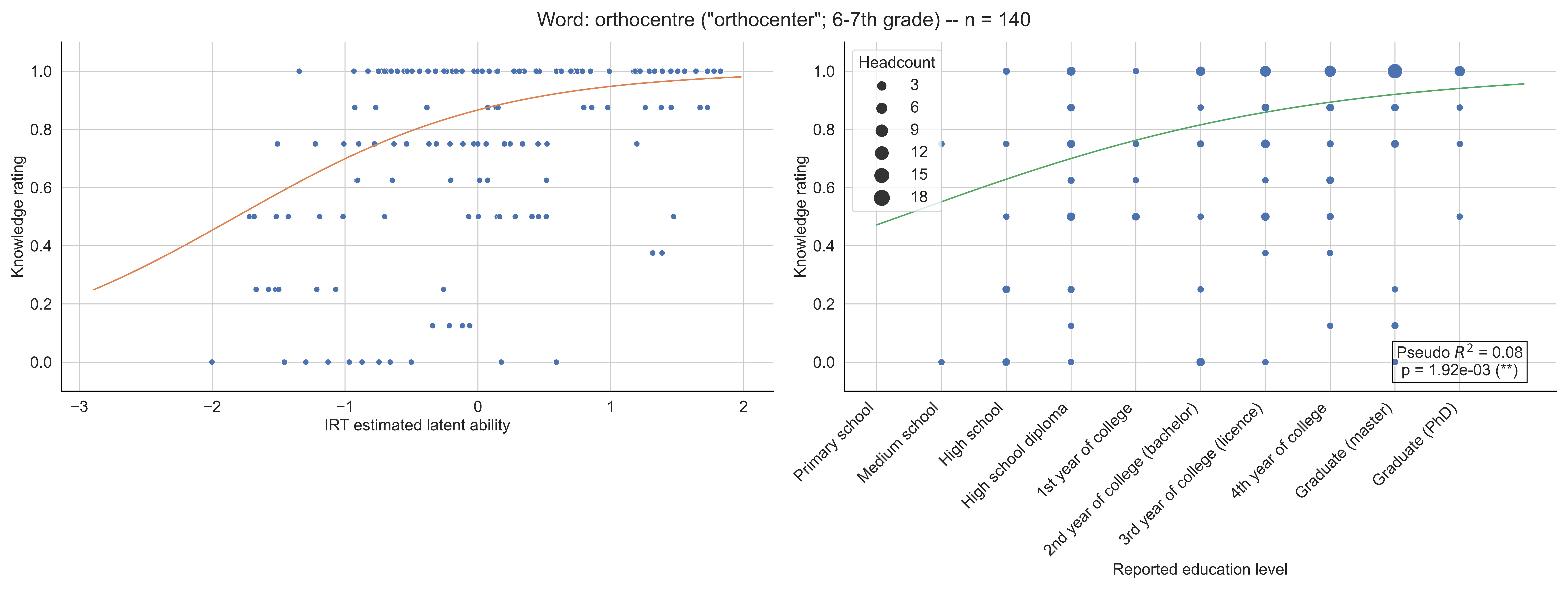

Word: orthocentre ("orthocenter"; 6-7th grade) -- n = 140
Knowledge rating
IRT estimated latent ability
Headcount
3
6
9
12
15
18
Primary school
Medium school
High school
High school diploma
1st year of college
2nd year of college (bachelor)
3rd year of college (licence)
4th year of college
Graduate (master)
Graduate (PhD)
Reported education level
Pseudo R^2 = 0.08
p = 1.92e-03 (**)

Word: orthogonalité ("orthogonality"; 11-12th grade) -- n = 122

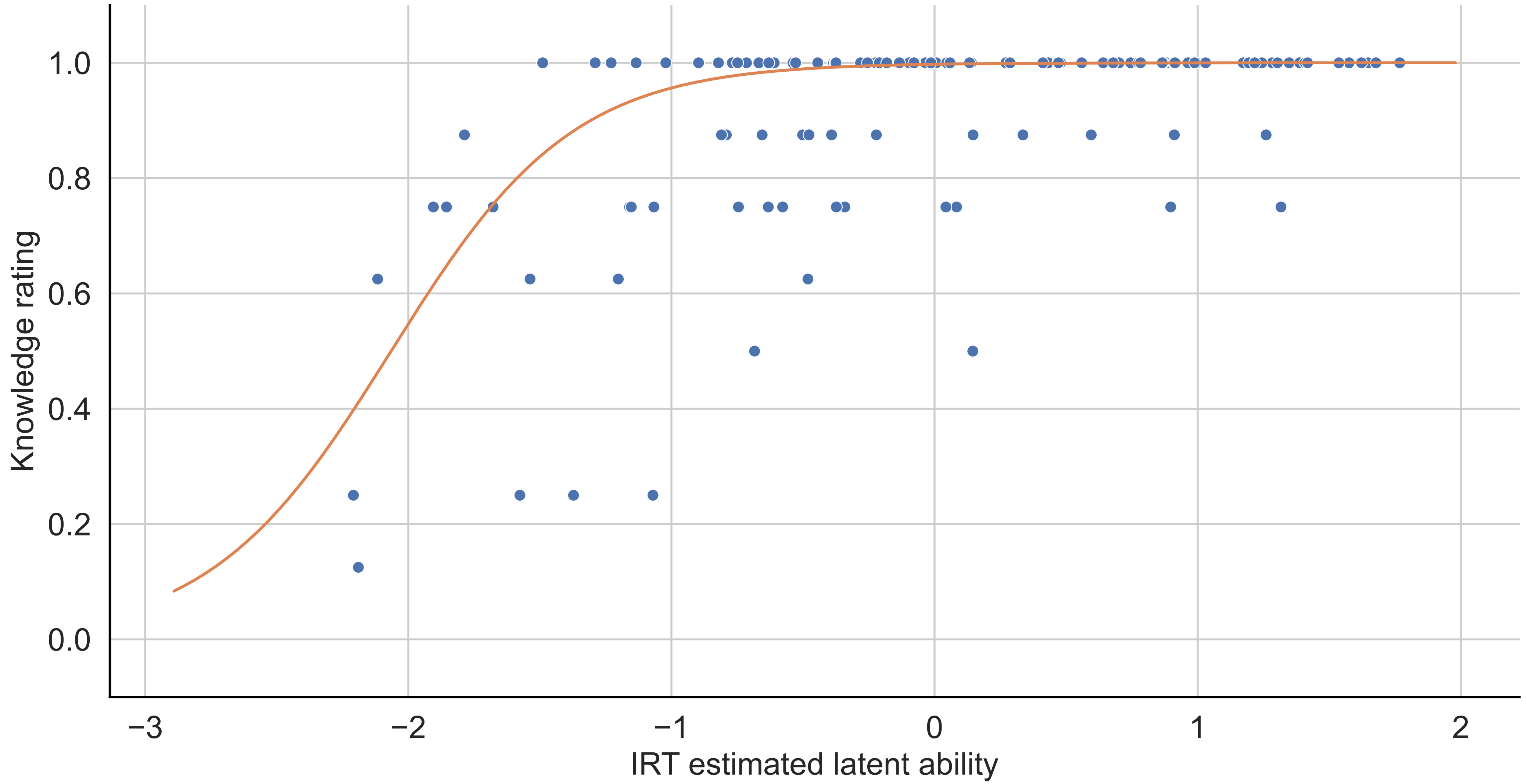

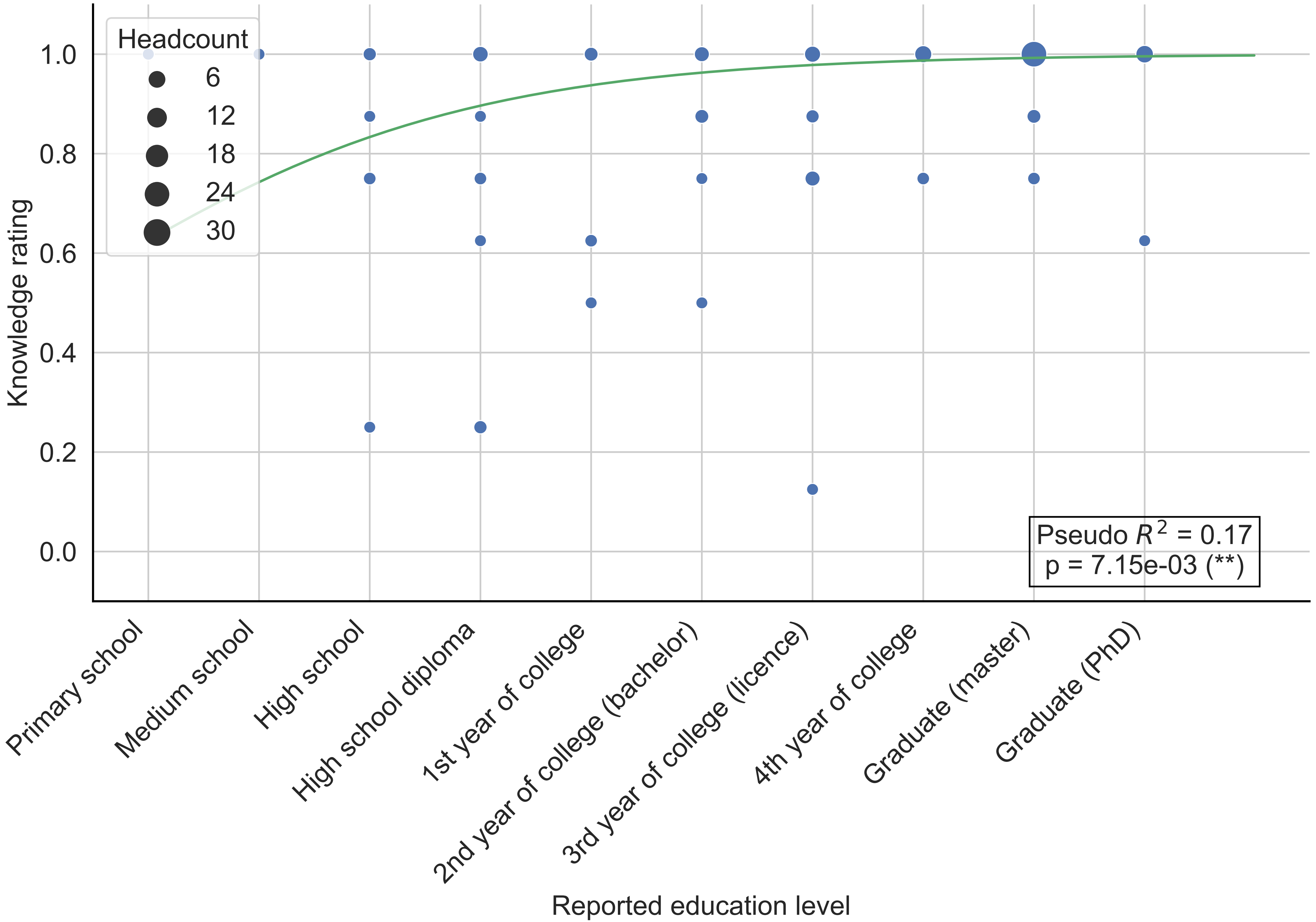

Word: ouvert ("open"; Bachelor) -- n = 155

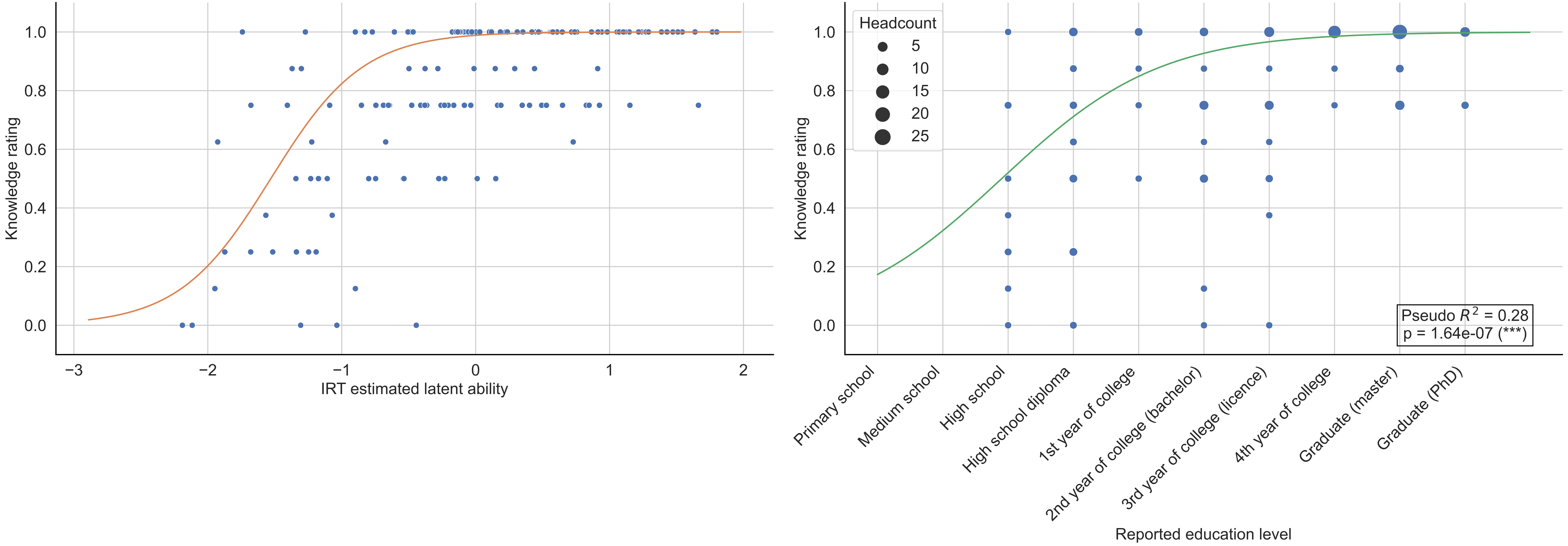

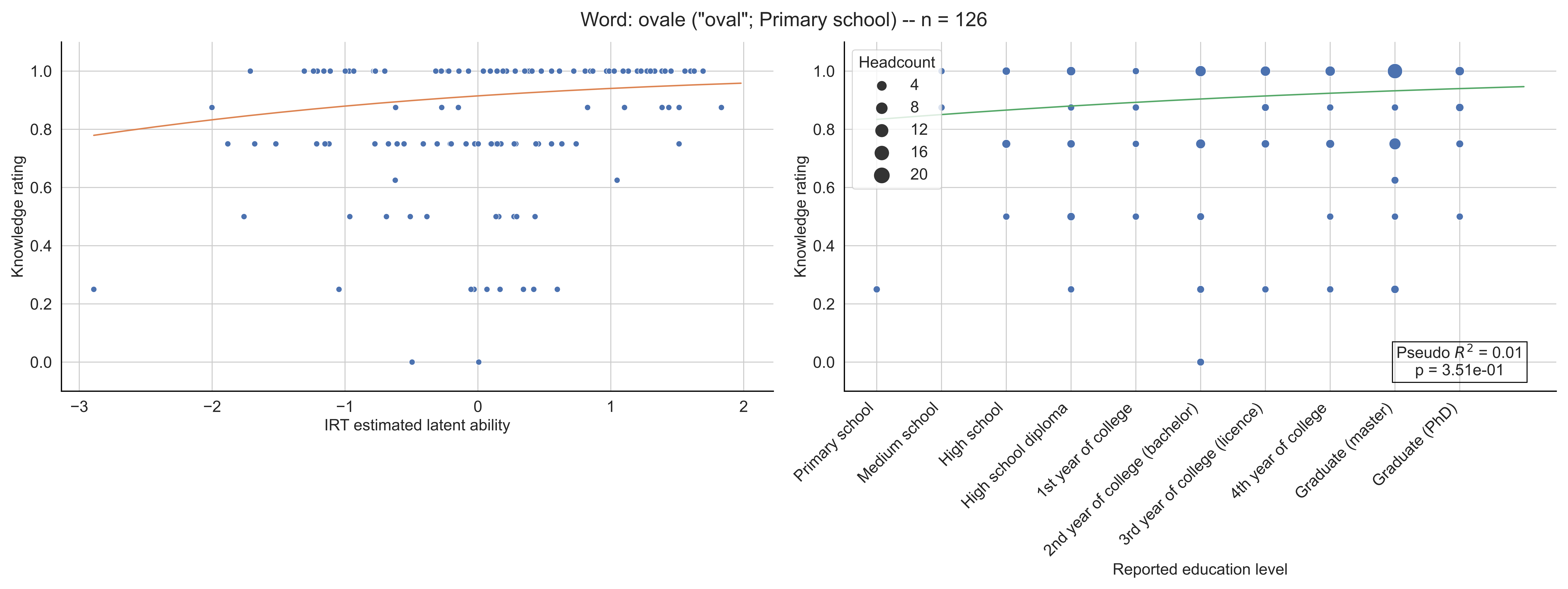

Word: ovale ("oval"; Primary school) -- n = 126
Knowledge rating
IRT estimated latent ability
Headcount
4
8
12
16
20
Pseudo R^2 = 0.01
p = 3.51e-01
Primary school
Medium school
High school
High school diploma
1st year of college
2nd year of college (bachelor)
3rd year of college (licence)
4th year of college
Graduate (master)
Graduate (PhD)
Reported education level

Word: parallélogramme ("parallelogram"; Primary school) -- n = 143

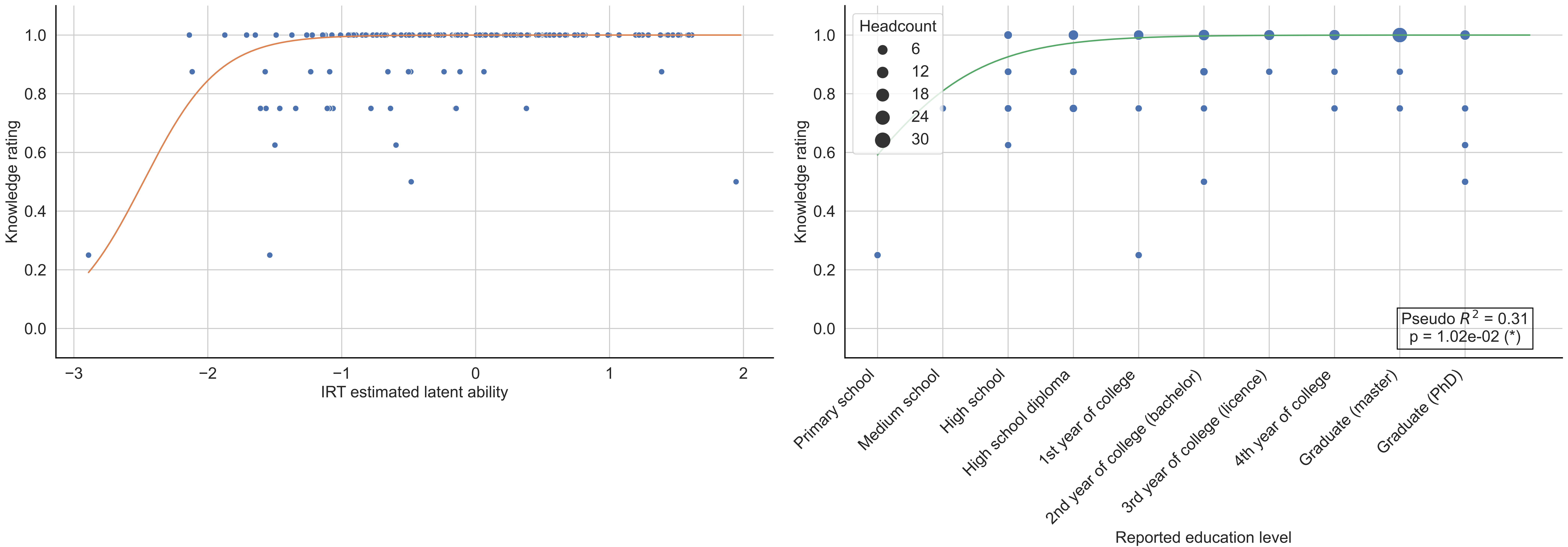

Word: paramétrisation ("parametrization"; Bachelor) -- n = 158

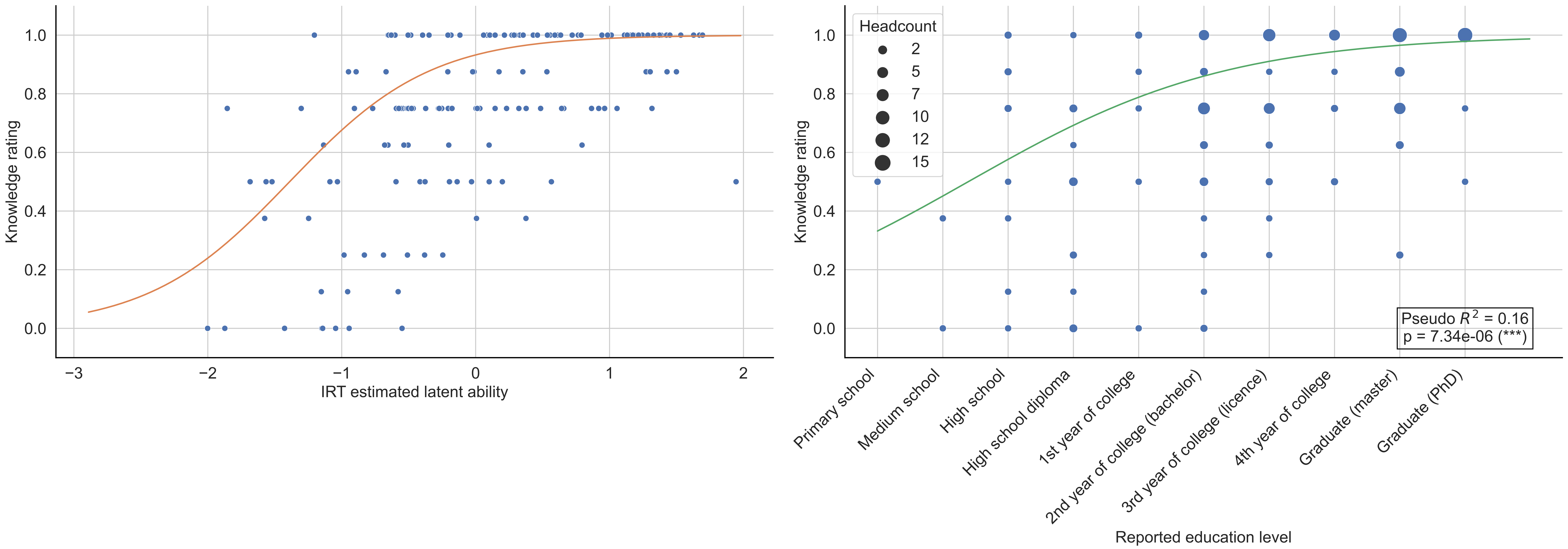

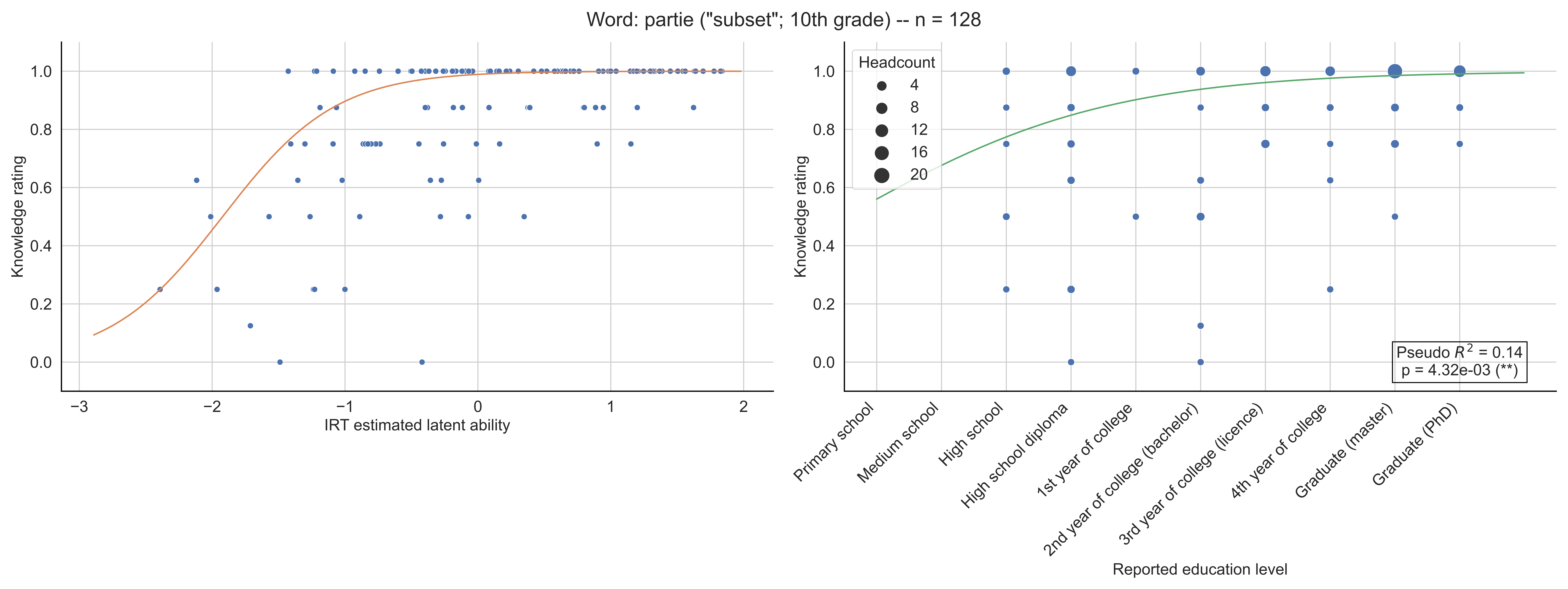

Word: partie ("subset"; 10th grade) -- n = 128
Knowledge rating
IRT estimated latent ability
Headcount
4
8
12
16
20
Pseudo R^2 = 0.14
p = 4.32e-03 (**)
Primary school
Medium school
High school
High school diploma
1st year of college
2nd year of college (bachelor)
3rd year of college (licence)
4th year of college
Graduate (master)
Graduate (PhD)
Reported education level

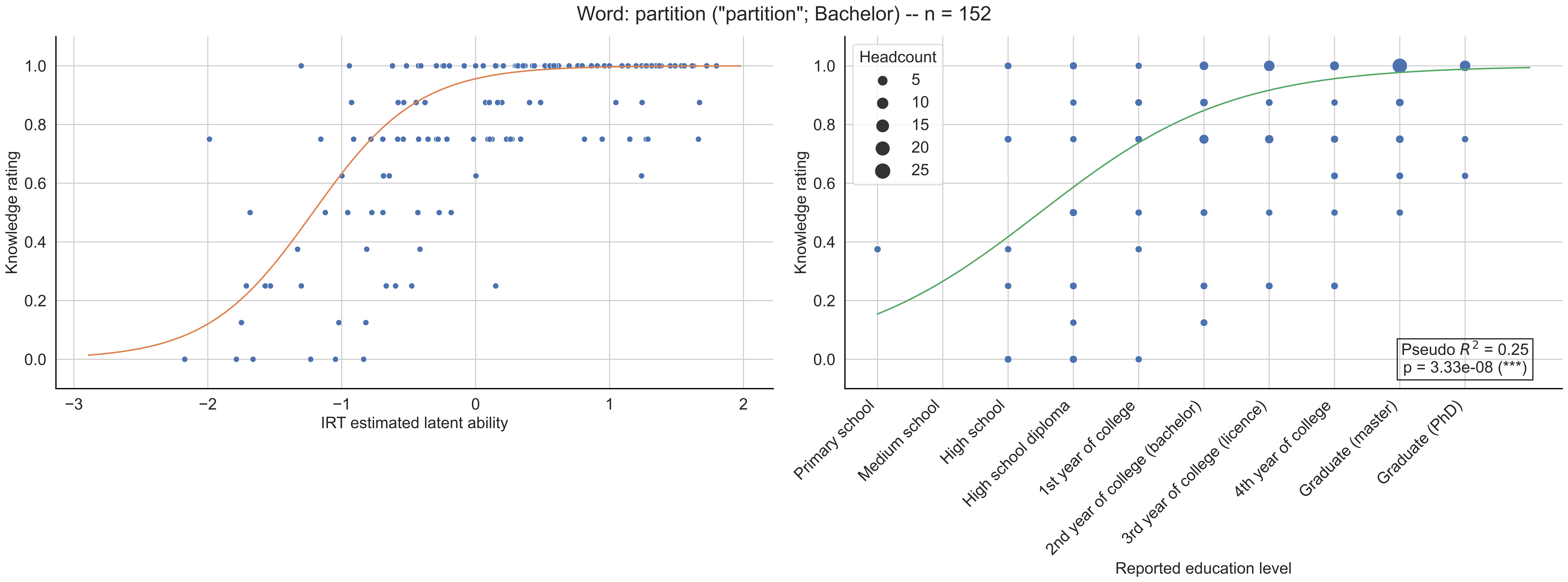

Word: partition ("partition"; Bachelor) -- n = 152
Knowledge rating
IRT estimated latent ability
Headcount
5
10
15
20
25
Pseudo R^2 = 0.25
p = 3.33e-08 (***)
Primary school
Medium school
High school
High school diploma
1st year of college
2nd year of college (bachelor)
3rd year of college (licence)
4th year of college
Graduate (master)
Graduate (PhD)
Reported education level

Word: pavage ("tiling"; Licence) -- n = 136

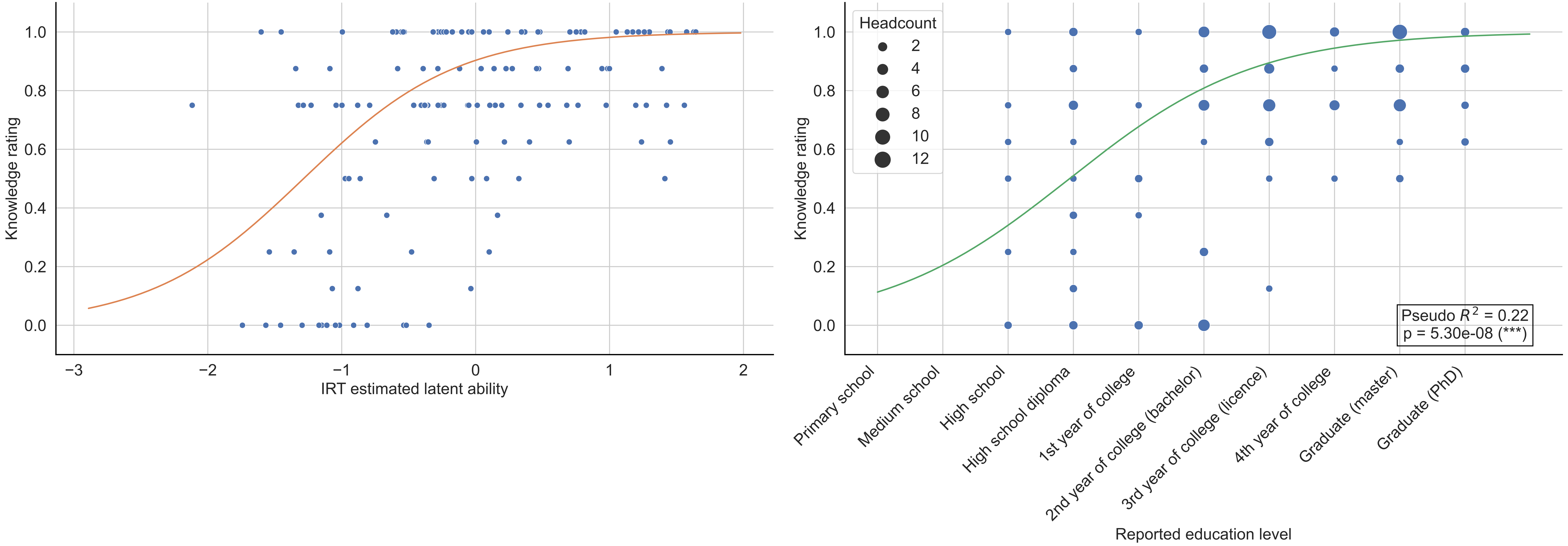

Word: permutation ("permutation"; Bachelor) -- n = 139

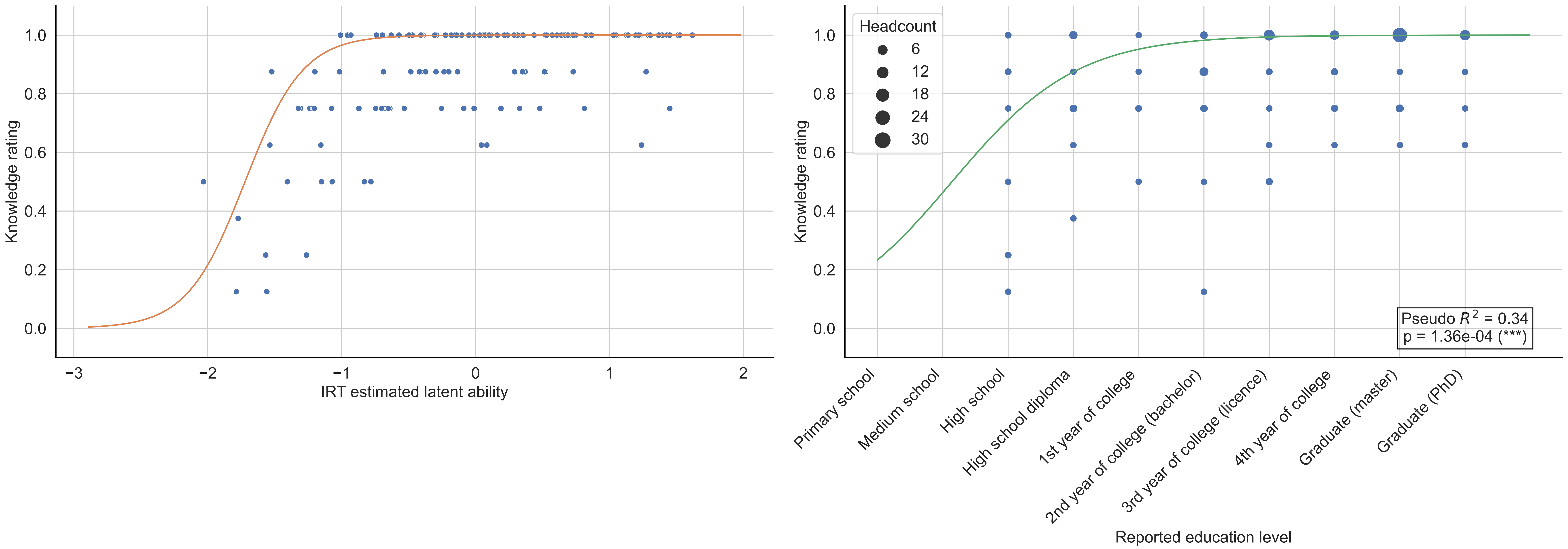

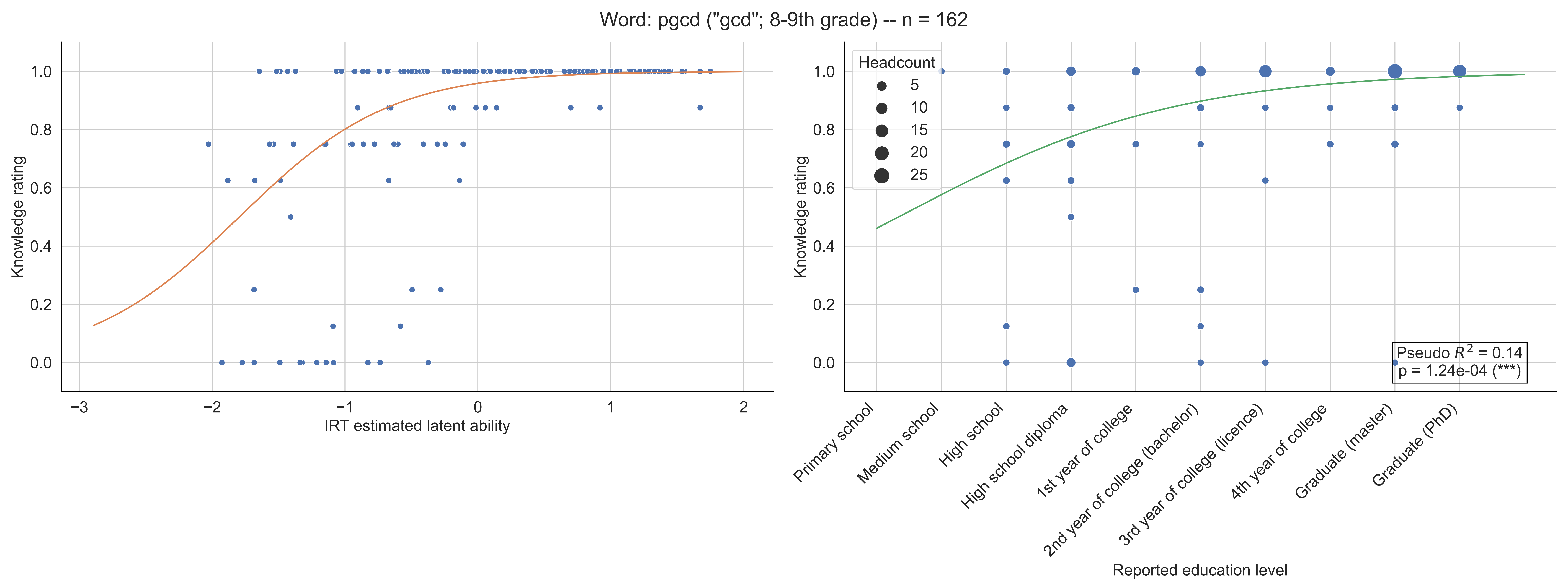

Word: pgcd ("gcd"; 8-9th grade) -- n = 162
Knowledge rating
IRT estimated latent ability
−3
−2
−1
0
1
2
0.0
0.2
0.4
0.6
0.8
1.0
Headcount
5
10
15
20
25
Pseudo R^2 = 0.14
p = 1.24e-04 (***)
Primary school
Medium school
High school
High school diploma
1st year of college
2nd year of college (bachelor)
3rd year of college (licence)
4th year of college
Graduate (master)
Graduate (PhD)
Reported education level

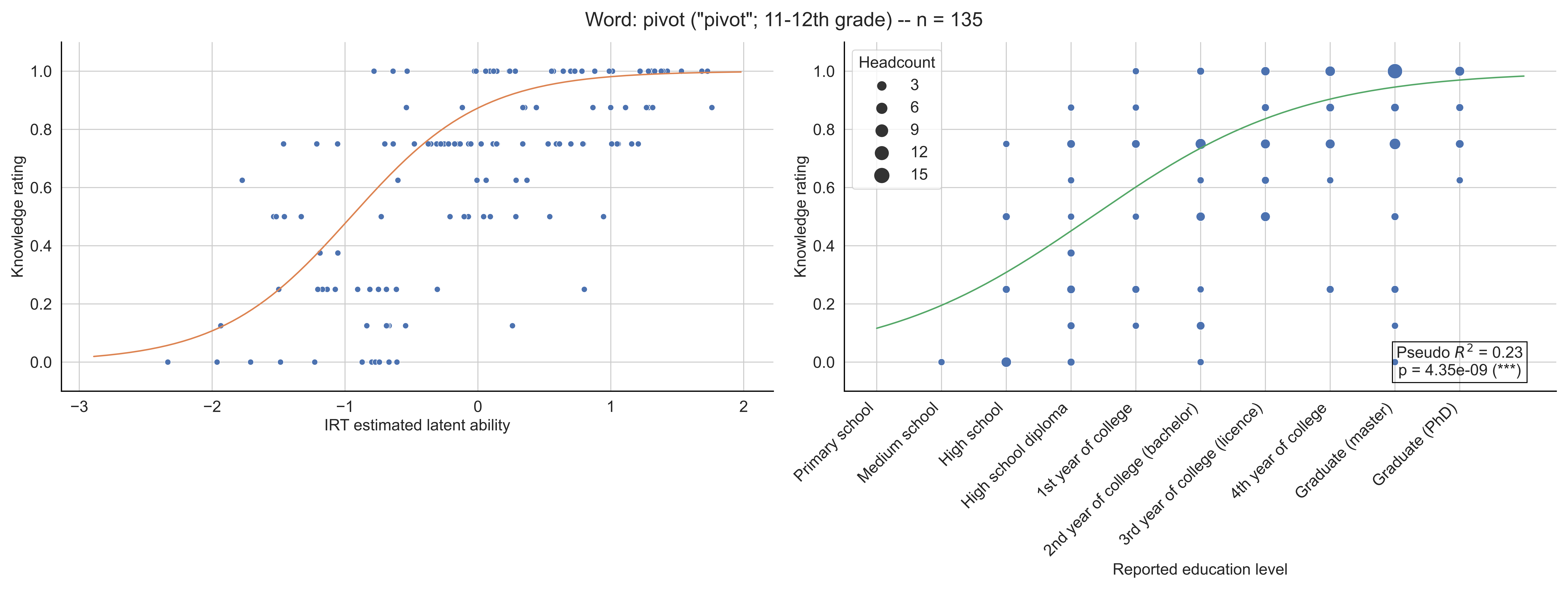

Word: pivot ("pivot"; 11-12th grade) -- n = 135
Knowledge rating
IRT estimated latent ability
Headcount
3
6
9
12
15
Pseudo R^2 = 0.23
p = 4.35e-09 (***)
Primary school
Medium school
High school
High school diploma
1st year of college
2nd year of college (bachelor)
3rd year of college (licence)
4th year of college
Graduate (master)
Graduate (PhD)
Reported education level

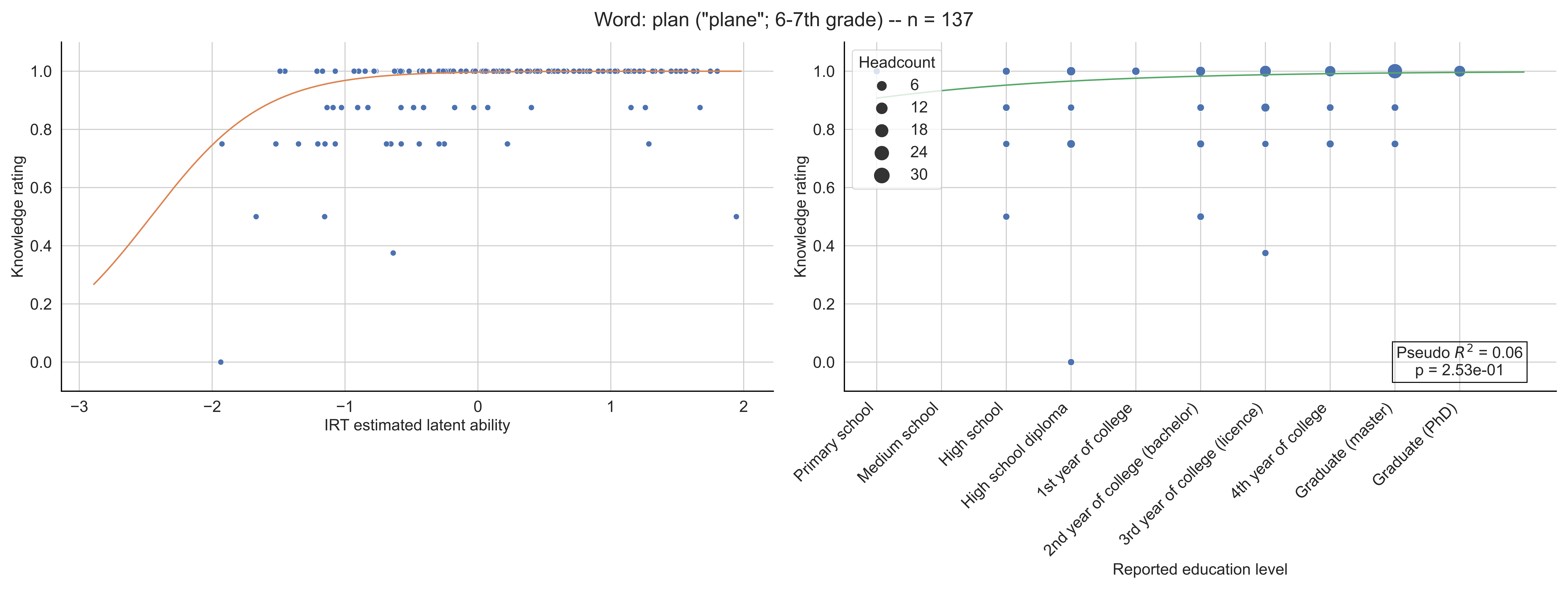
Word: plan ("plane"; 6-7th grade) -- n = 137
Knowledge rating
IRT estimated latent ability
Headcount
6
12
18
24
30
Pseudo R^2 = 0.06
p = 2.53e-01
Primary school
Medium school
High school
High school diploma
1st year of college
2nd year of college (bachelor)
3rd year of college (licence)
4th year of college
Graduate (master)
Graduate (PhD)
Reported education level

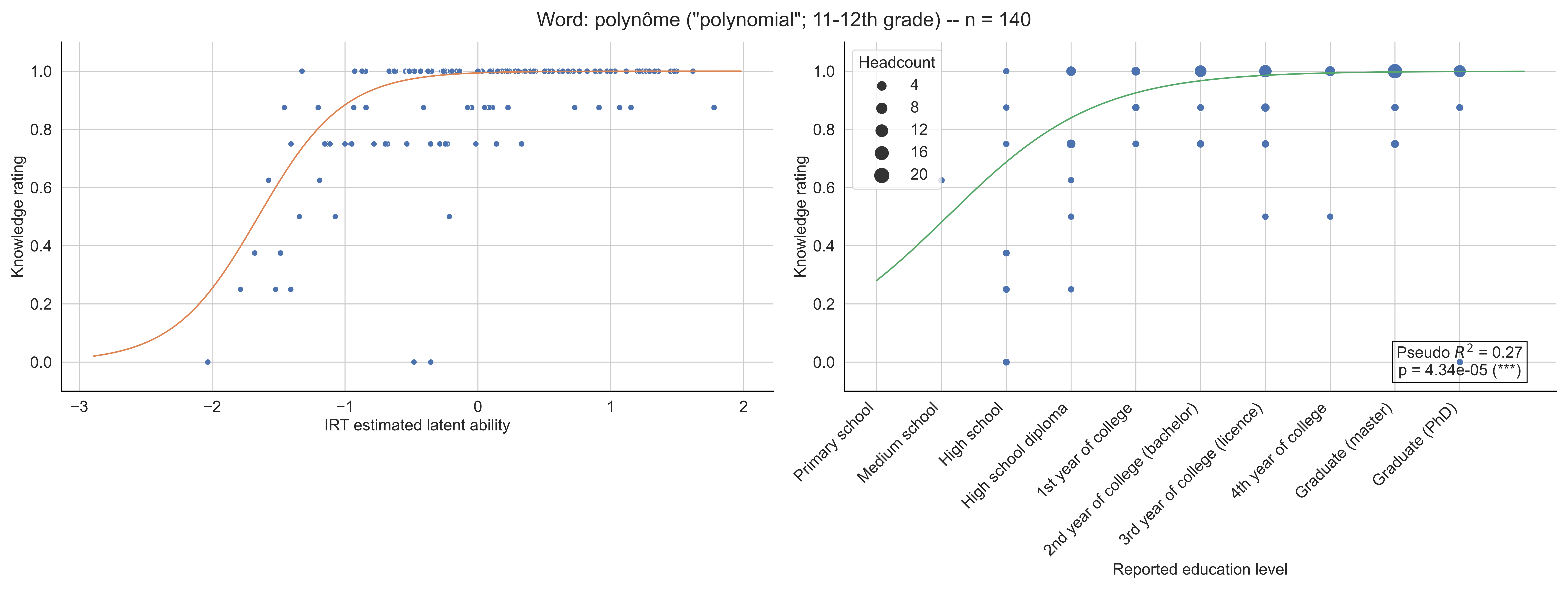

Word: polynôme ("polynomial"; 11-12th grade) -- n = 140
Knowledge rating
IRT estimated latent ability
−3
−2
−1
0
1
2
0.0
0.2
0.4
0.6
0.8
1.0
Headcount
4
8
12
16
20
Pseudo R^2 = 0.27
p = 4.34e-05 (***)
Primary school
Medium school
High school
High school diploma
1st year of college
2nd year of college (bachelor)
3rd year of college (licence)
4th year of college
Graduate (master)
Graduate (PhD)
Reported education level

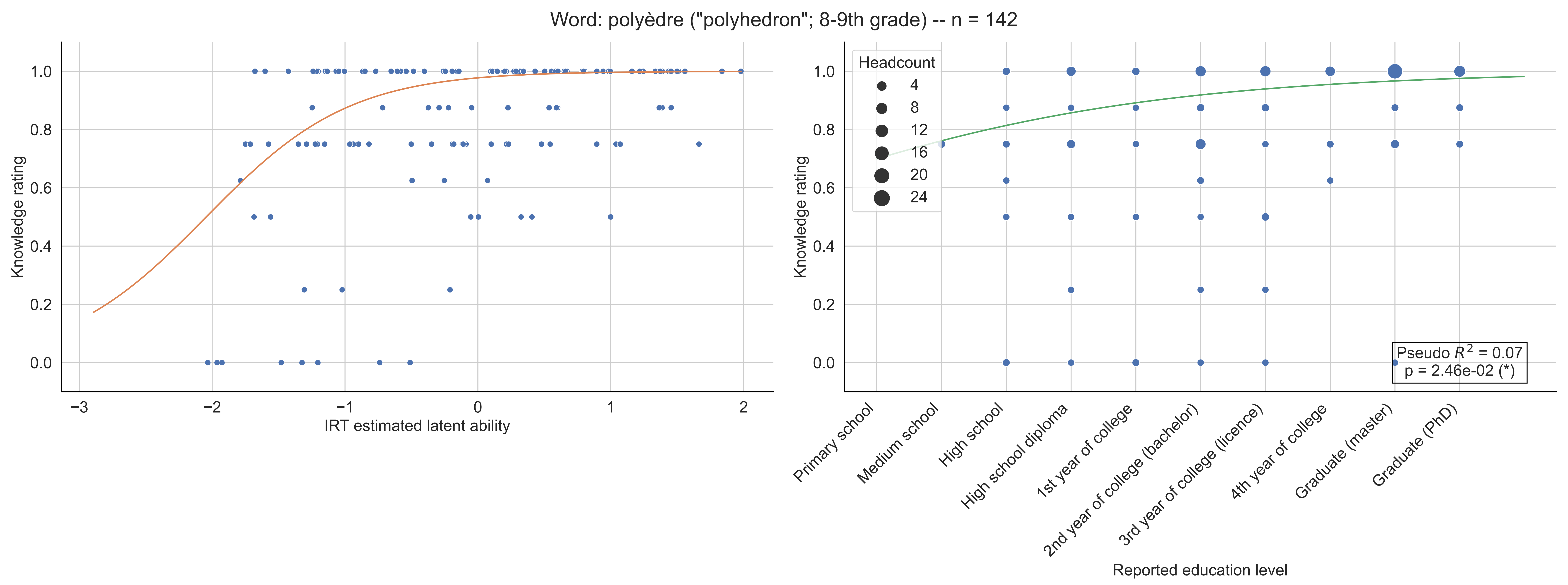
Word: polyèdre ("polyhedron"; 8-9th grade) -- n = 142
Knowledge rating
IRT estimated latent ability
Headcount
4
8
12
16
20
24
Pseudo R^2 = 0.07
p = 2.46e-02 (*)
Primary school
Medium school
High school
High school diploma
1st year of college
2nd year of college (bachelor)
3rd year of college (licence)
4th year of college
Graduate (master)
Graduate (PhD)
Reported education level

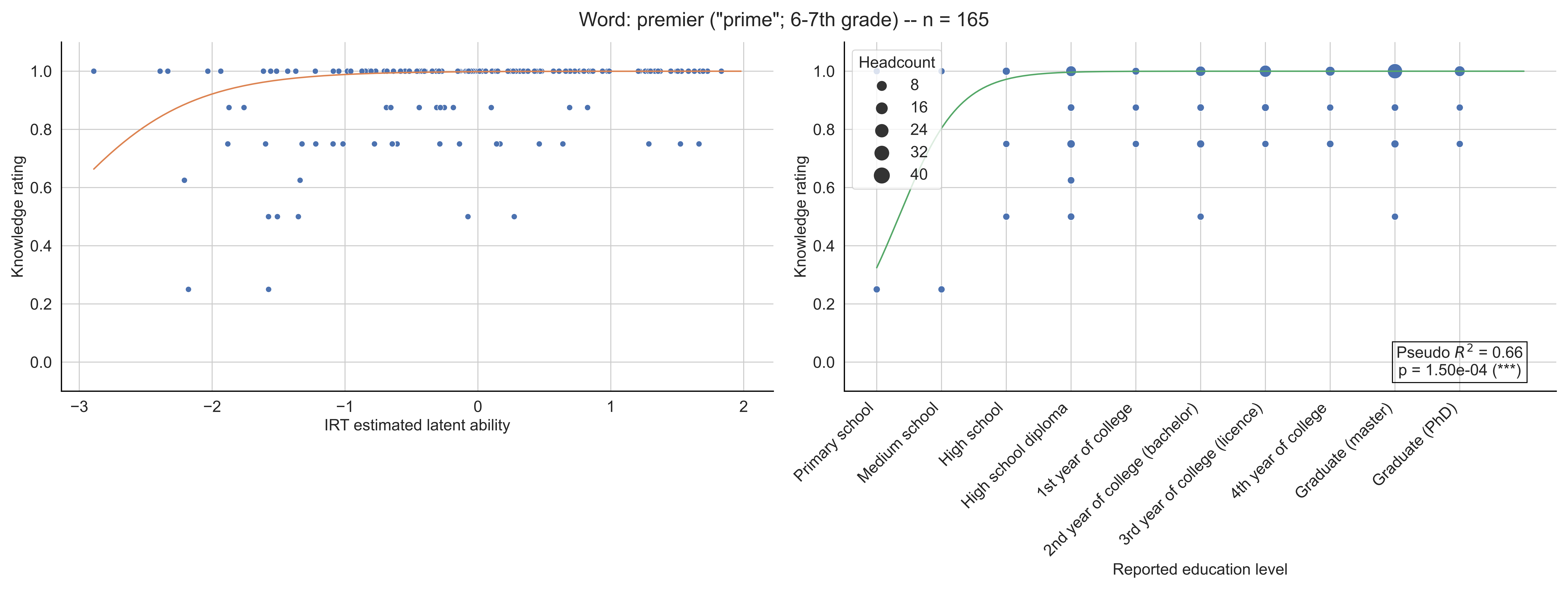

Word: premier ("prime"; 6-7th grade) -- n = 165
Knowledge rating
IRT estimated latent ability
−3
−2
−1
0
1
2
0.0
0.2
0.4
0.6
0.8
1.0
Headcount
8
16
24
32
40
Pseudo R^2 = 0.66
p = 1.50e-04 (***)
Primary school
Medium school
High school
High school diploma
1st year of college
2nd year of college (bachelor)
3rd year of college (licence)
4th year of college
Graduate (master)
Graduate (PhD)
Reported education level

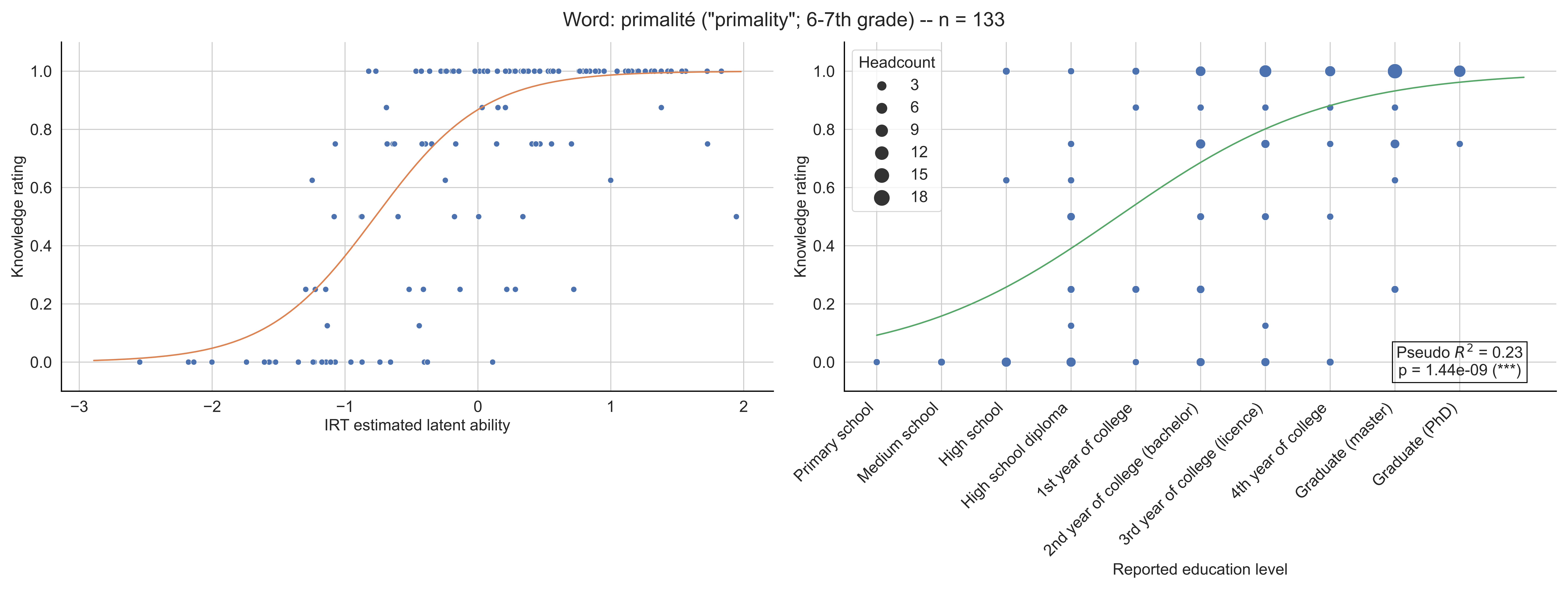
Word: primalité ("primality"; 6-7th grade) -- n = 133
Knowledge rating
IRT estimated latent ability
Headcount
3
6
9
12
15
18
Pseudo R^2 = 0.23
p = 1.44e-09 (***)
Primary school
Medium school
High school
High school diploma
1st year of college
2nd year of college (bachelor)
3rd year of college (licence)
4th year of college
Graduate (master)
Graduate (PhD)
Reported education level

Word: prisme ("prism"; 6-7th grade) -- n = 139

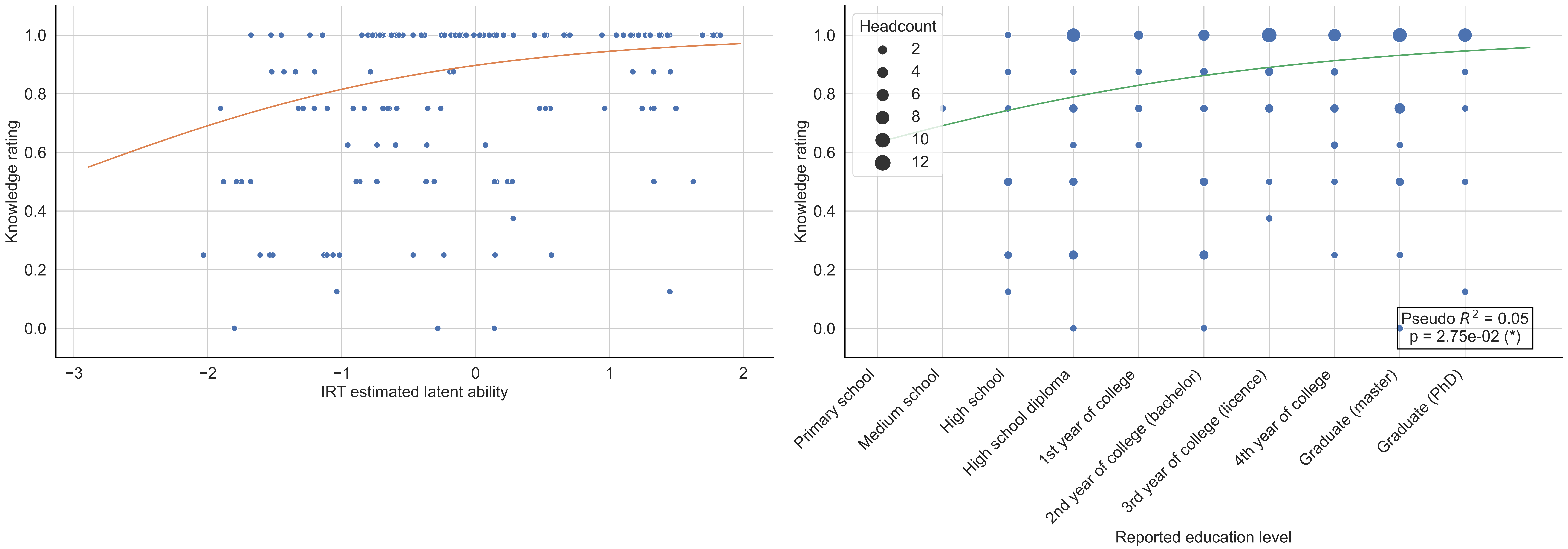

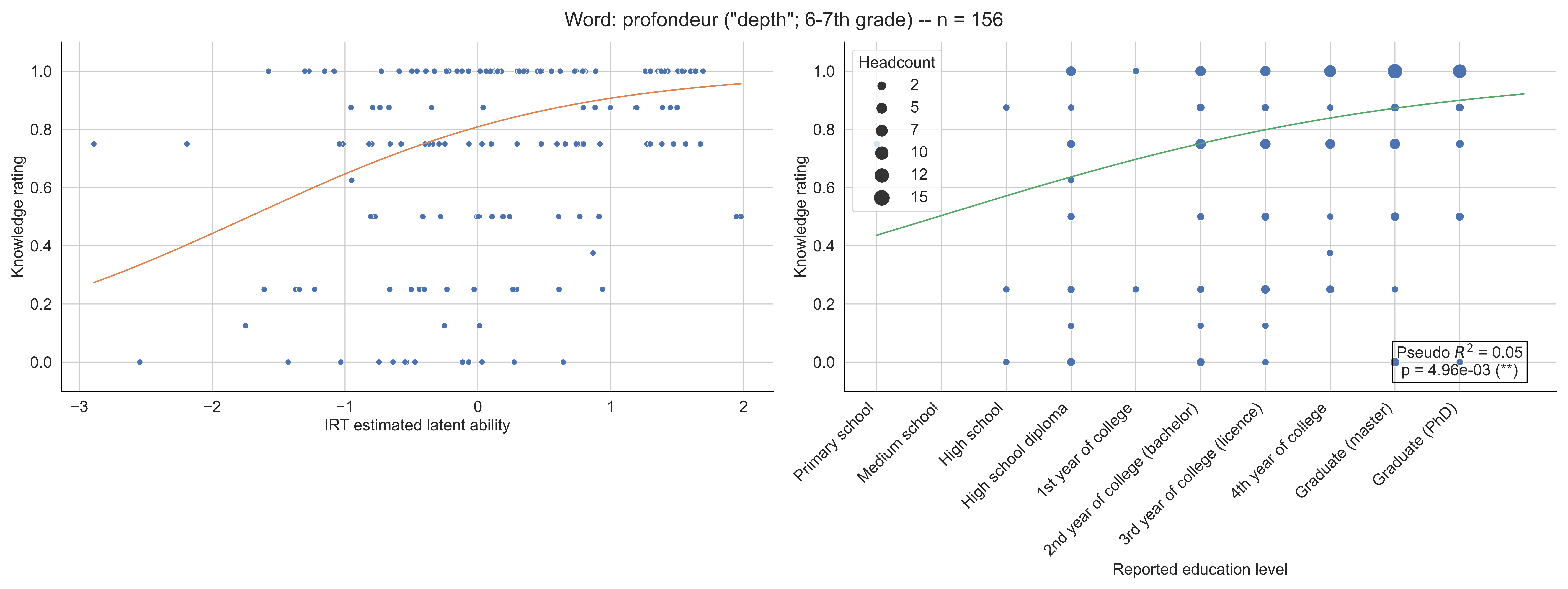
Word: profondeur ("depth"; 6-7th grade) -- n = 156
Knowledge rating
IRT estimated latent ability
Headcount
2
5
7
10
12
15
Pseudo R^2 = 0.05
p = 4.96e-03 (**)
Primary school
Medium school
High school
High school diploma
1st year of college
2nd year of college (bachelor)
3rd year of college (licence)
4th year of college
Graduate (master)
Graduate (PhD)
Reported education level

Word: prédicat ("predicate"; Licence) -- n = 125

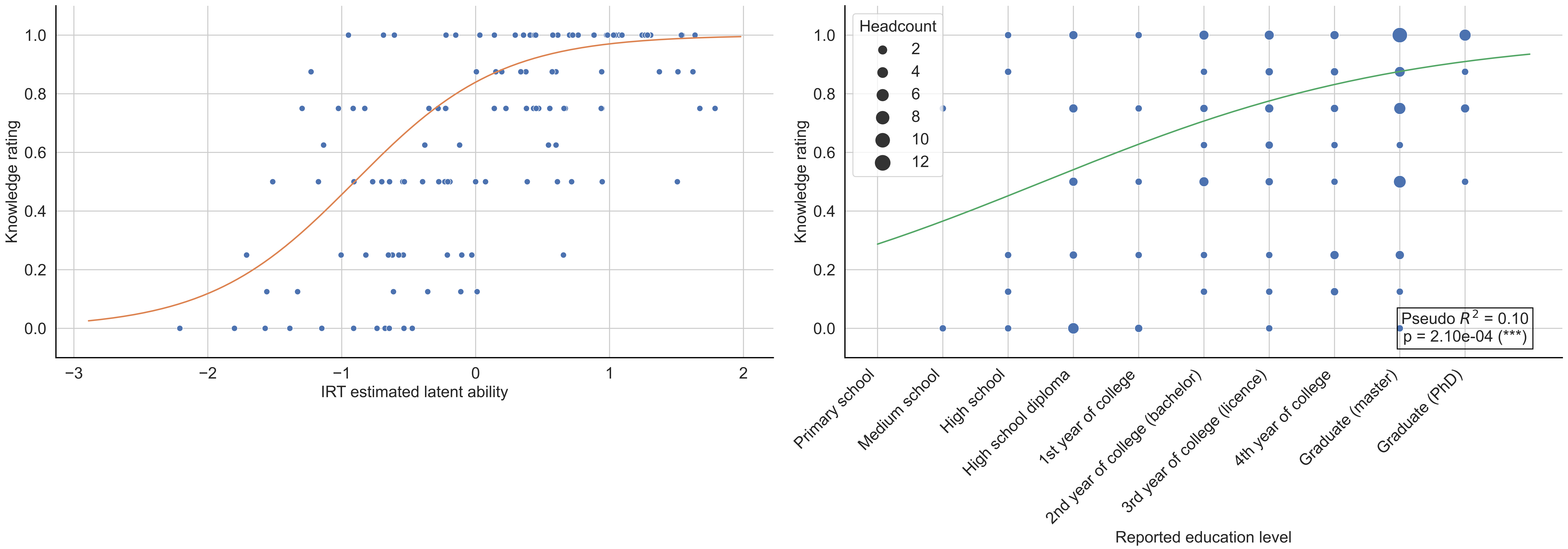

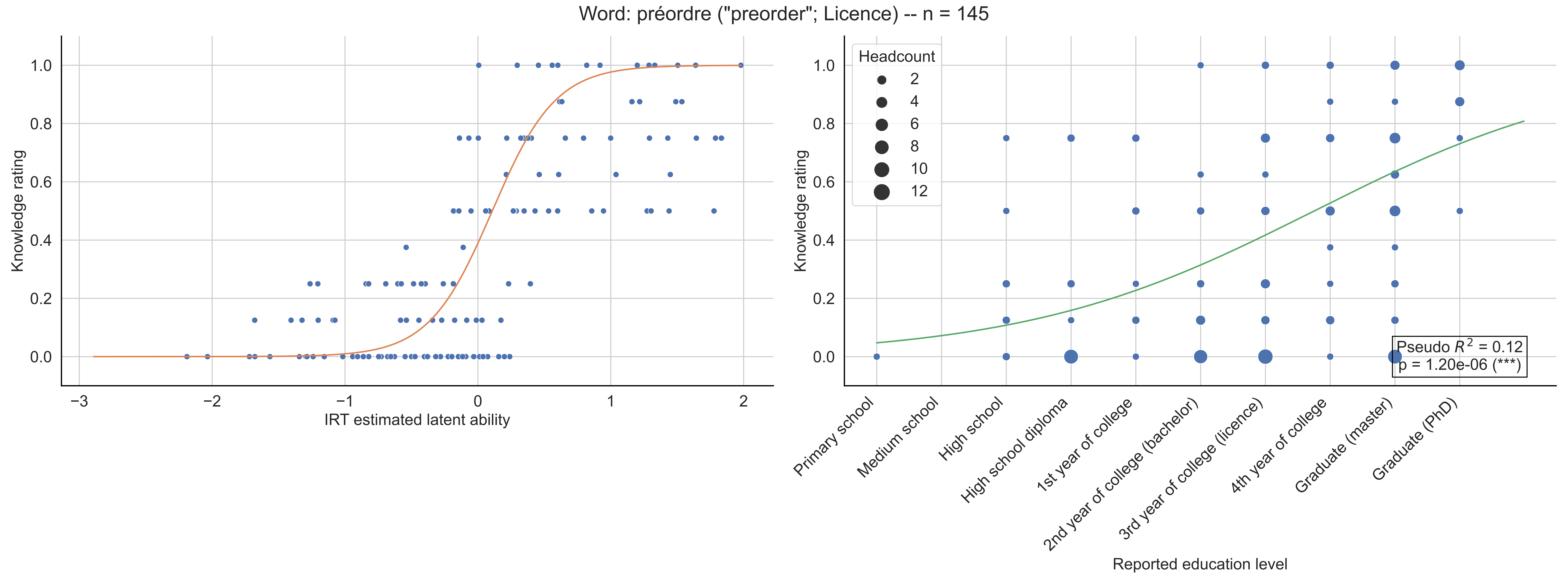

Word: préordre ("preorder"; Licence) -- n = 145
Knowledge rating
IRT estimated latent ability
Headcount
2
4
6
8
10
12
Pseudo R^2 = 0.12
p = 1.20e-06 (***)
Primary school
Medium school
High school
High school diploma
1st year of college
2nd year of college (bachelor)
3rd year of college (licence)
4th year of college
Graduate (master)
Graduate (PhD)
Reported education level

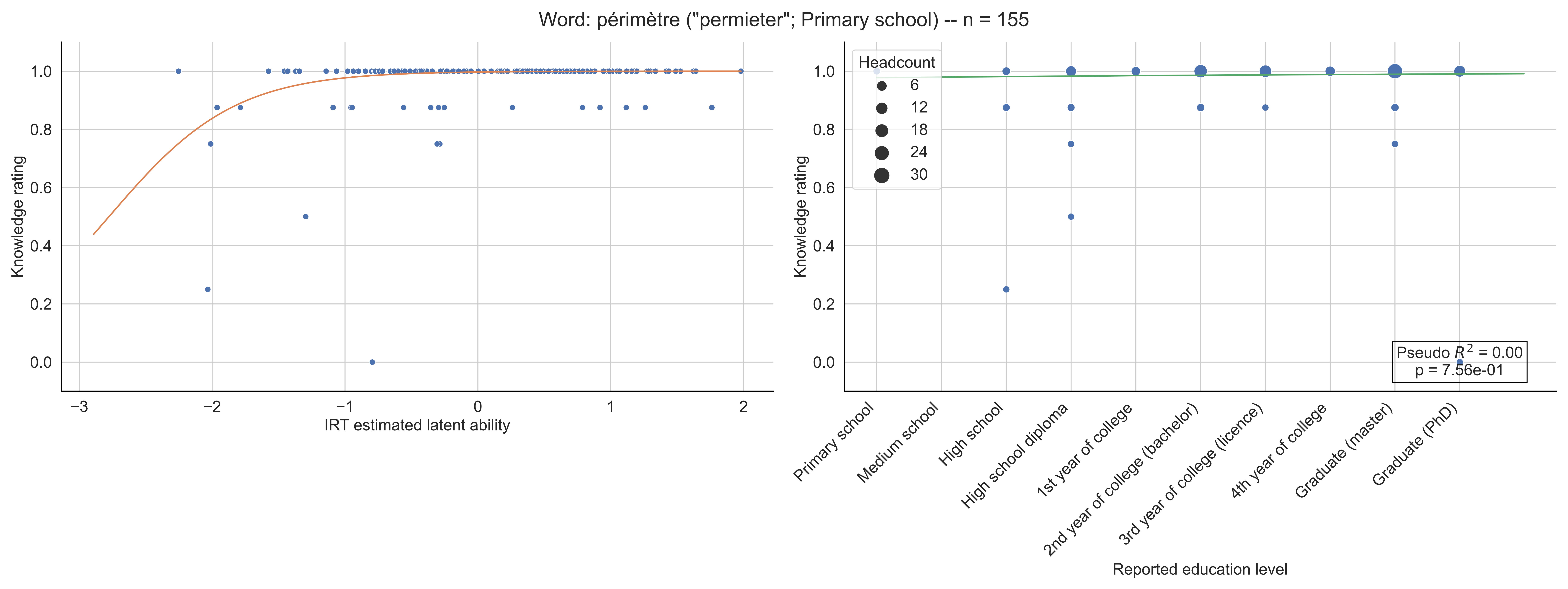

Word: périmètre ("permieter"; Primary school) -- n = 155
Knowledge rating
IRT estimated latent ability
Headcount
6
12
18
24
30
Pseudo R^2 = 0.00
p = 7.56e-01
Primary school
Medium school
High school
High school diploma
1st year of college
2nd year of college (bachelor)
3rd year of college (licence)
4th year of college
Graduate (master)
Graduate (PhD)
Reported education level

Word: périodicité ("periodicity"; 11-12th grade) -- n = 145

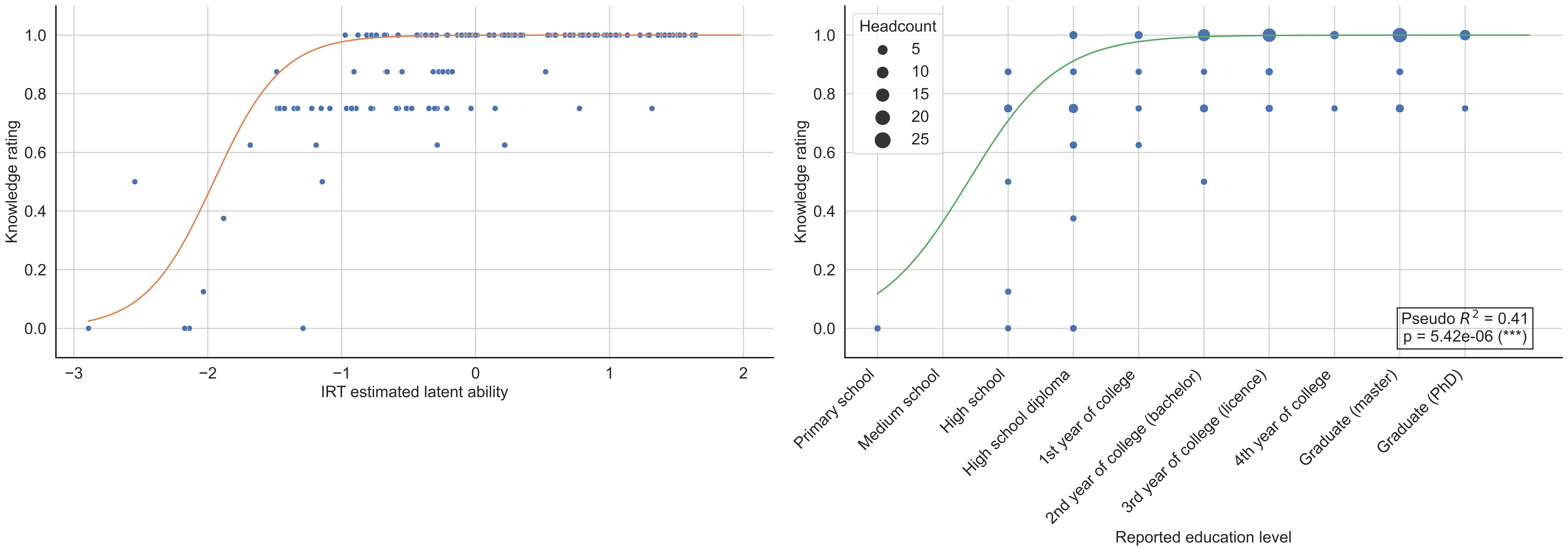

Word: pôle ("pole"; Licence) -- n = 120

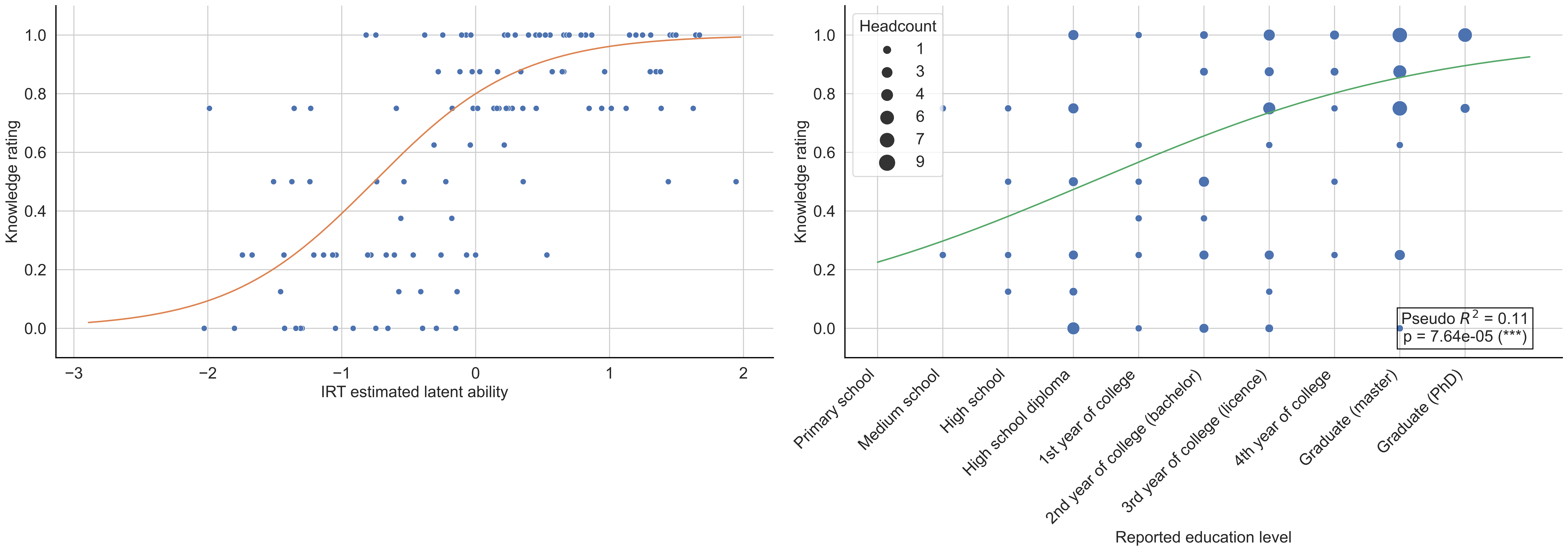

Word: quadrature ("quadrature"; Master) -- n = 166

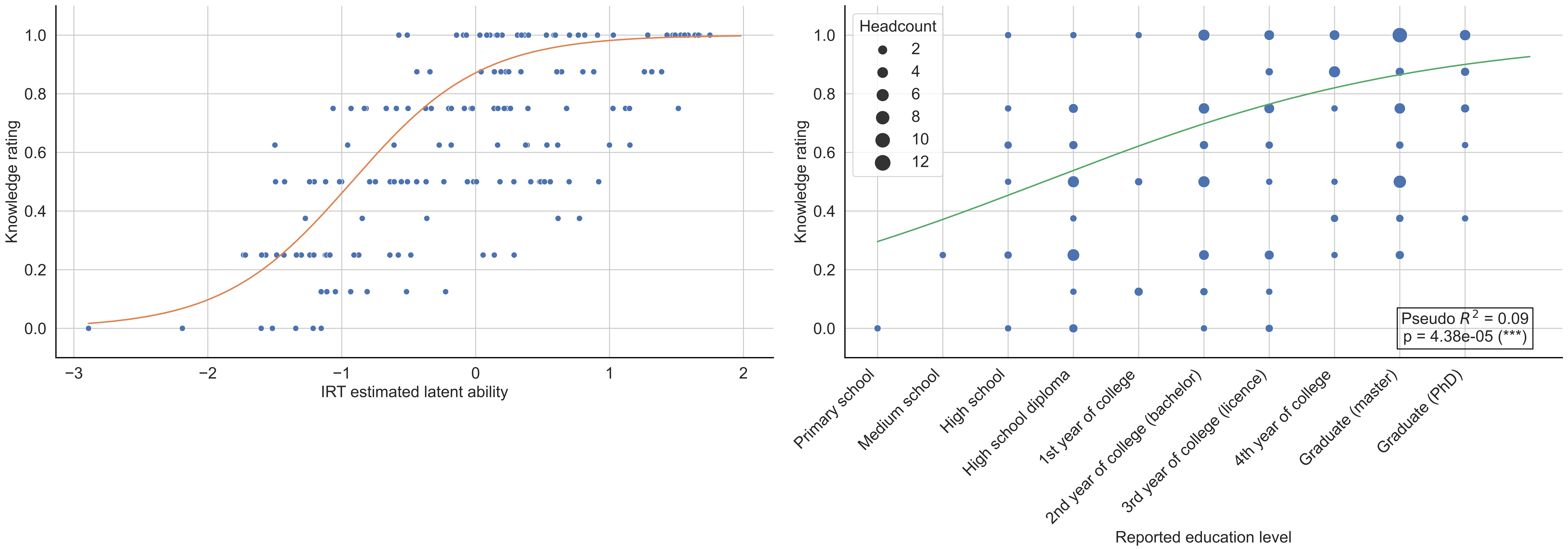

Word: quadrilatère ("quadrilateral"; Primary school) -- n = 140

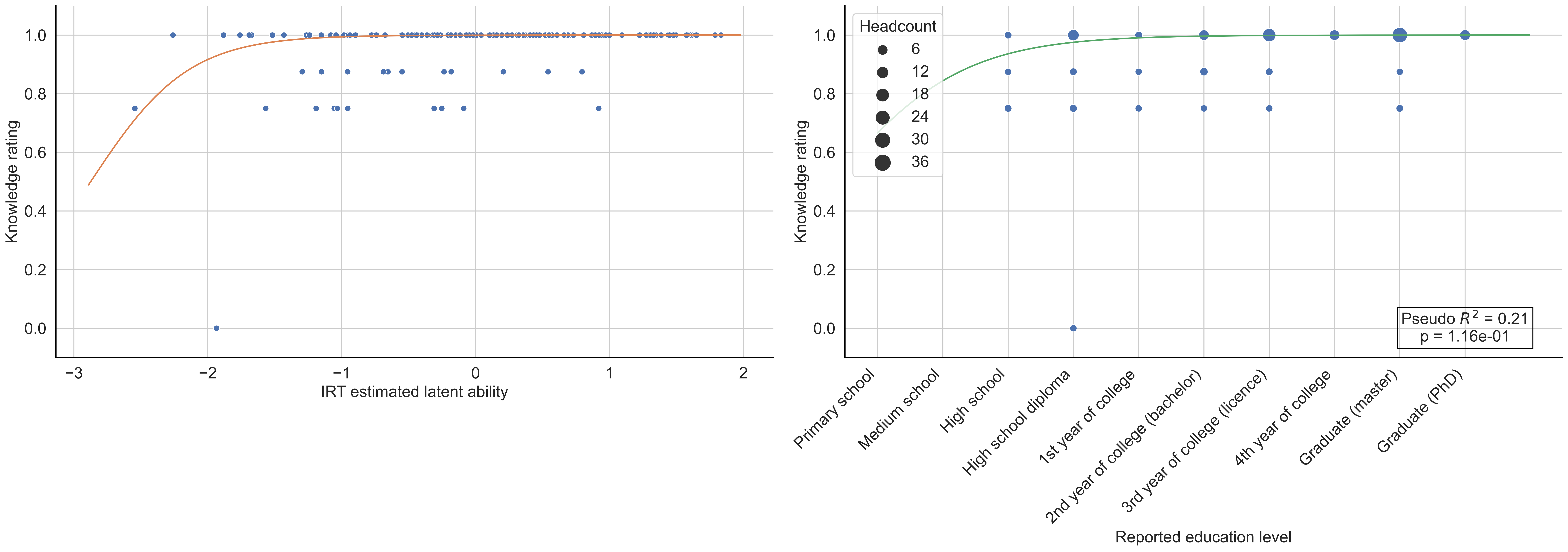

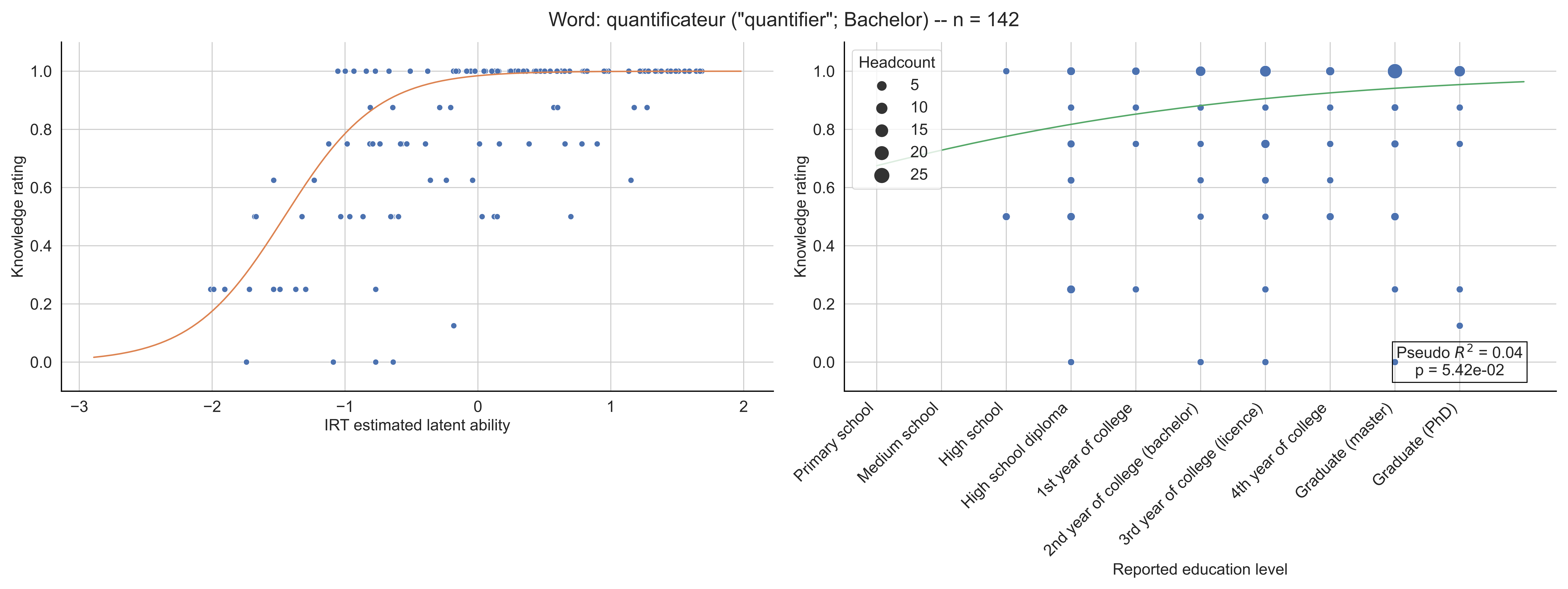

Word: quantificateur ("quantifier"; Bachelor) -- n = 142
Knowledge rating
IRT estimated latent ability
Headcount
5
10
15
20
25
Pseudo R^2 = 0.04
p = 5.42e-02
Primary school
Medium school
High school
High school diploma
1st year of college
2nd year of college (bachelor)
3rd year of college (licence)
4th year of college
Graduate (master)
Graduate (PhD)
Reported education level

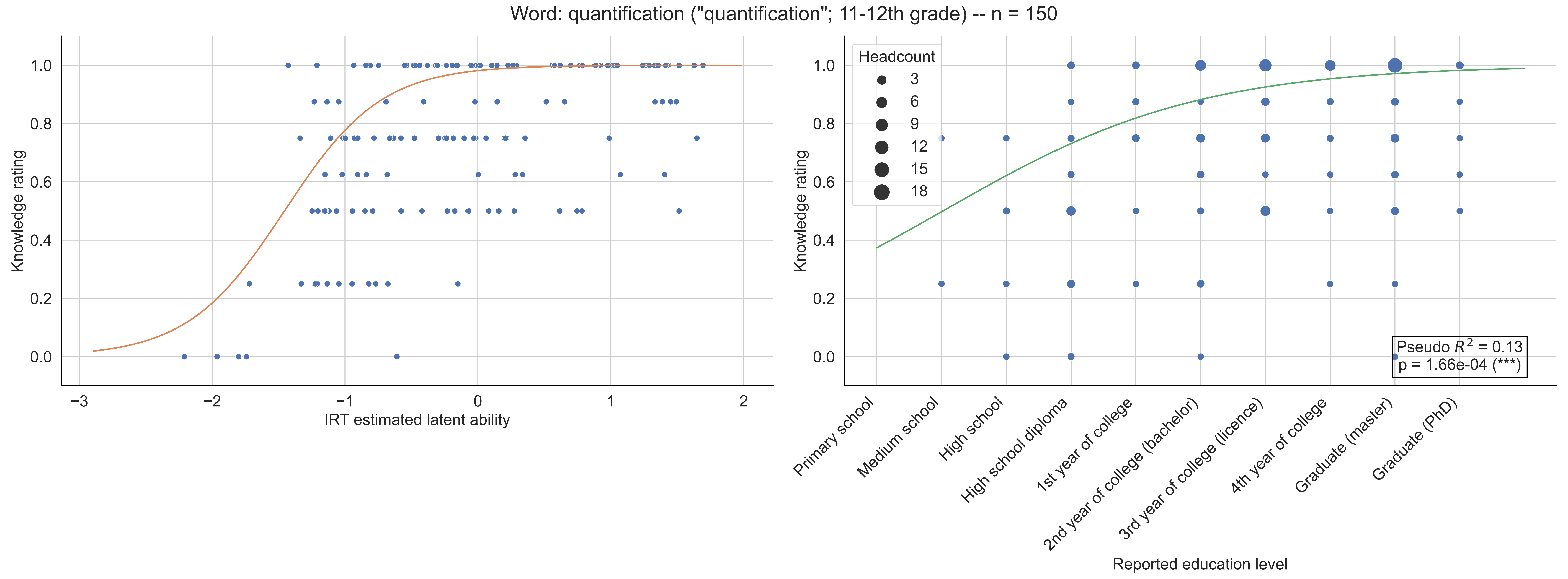

Word: quantification ("quantification"; 11-12th grade) -- n = 150
Knowledge rating
IRT estimated latent ability
Headcount
3
6
9
12
15
18
Pseudo R^2 = 0.13
p = 1.66e-04 (***)
Primary school
Medium school
High school
High school diploma
1st year of college
2nd year of college (bachelor)
3rd year of college (licence)
4th year of college
Graduate (master)
Graduate (PhD)
Reported education level

Word: quartile ("quartile"; 10th grade) -- n = 138

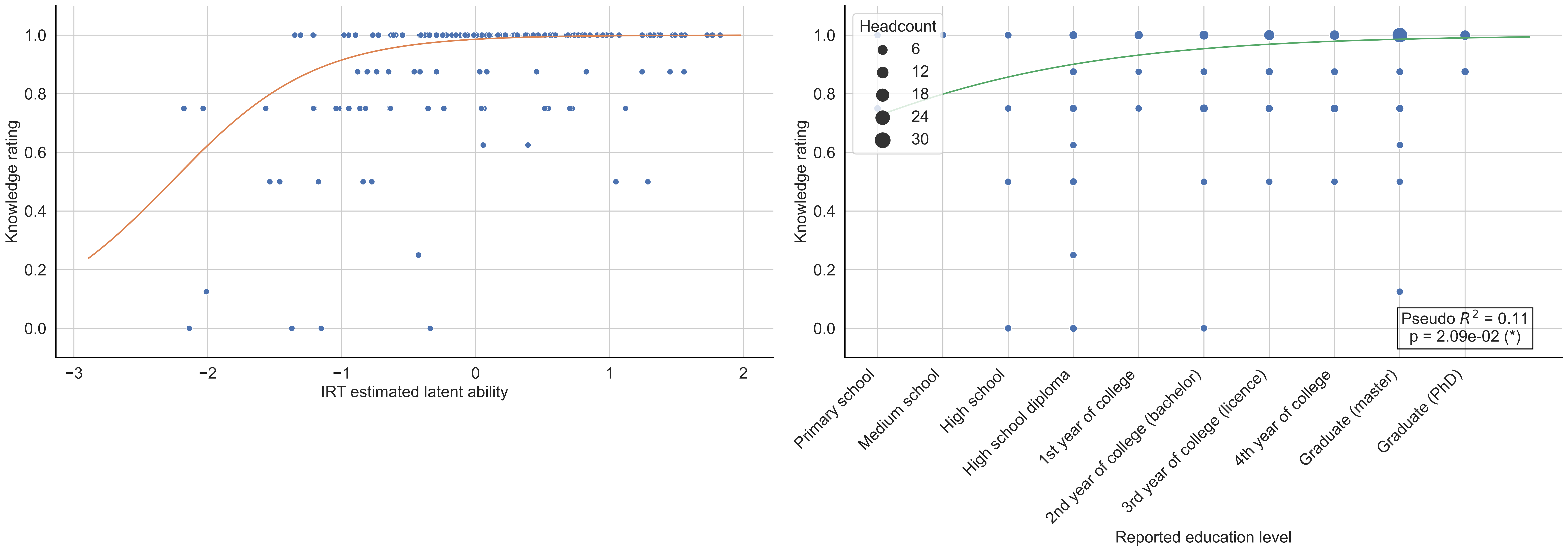

Word: quaternion ("quaternion"; Bachelor) -- n = 139

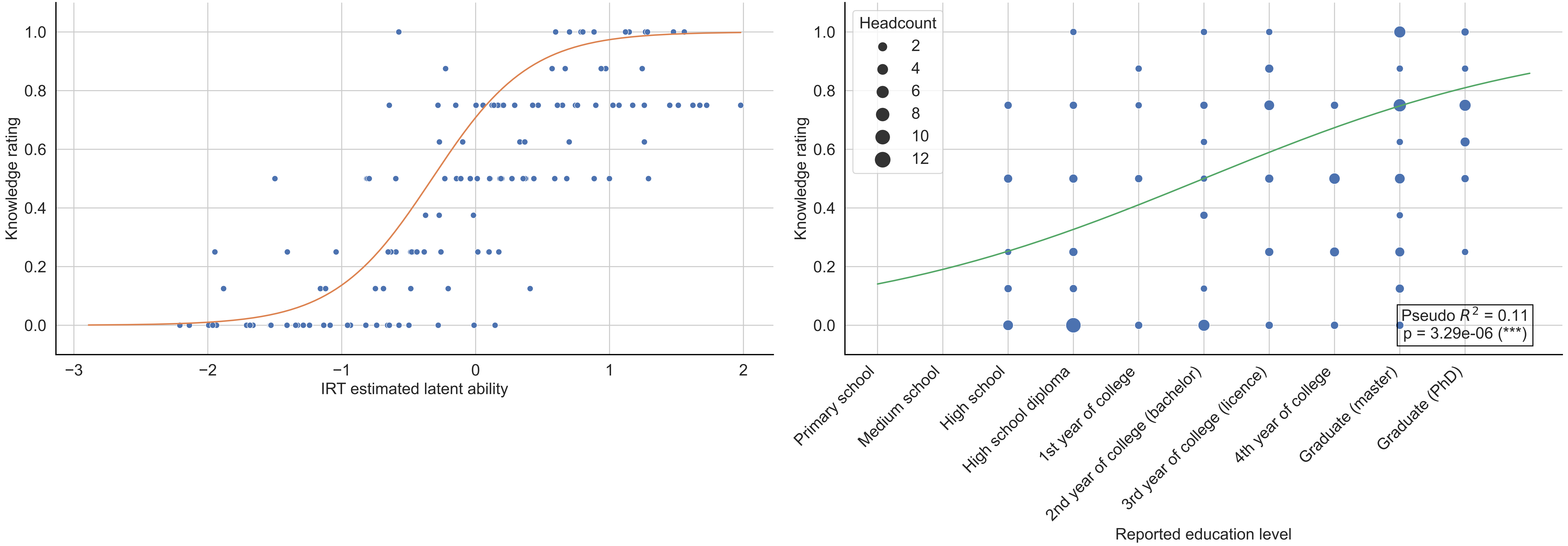

Word: quatorze ("fourteen"; Primary school) -- n = 153

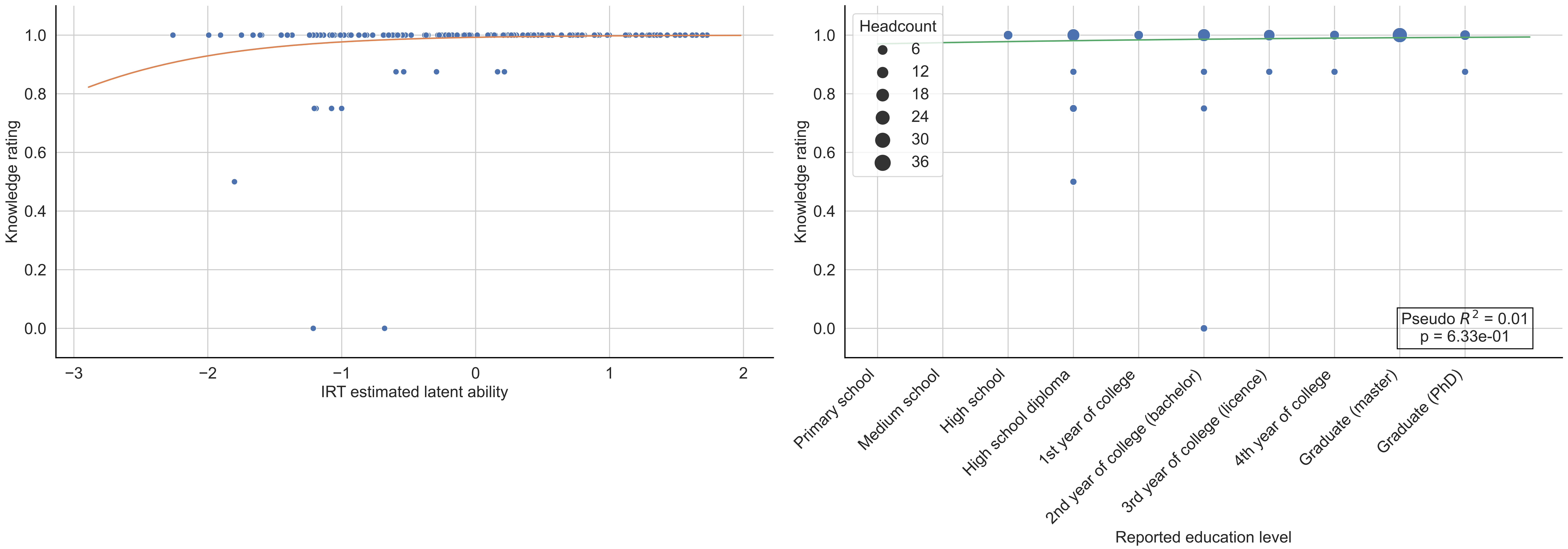

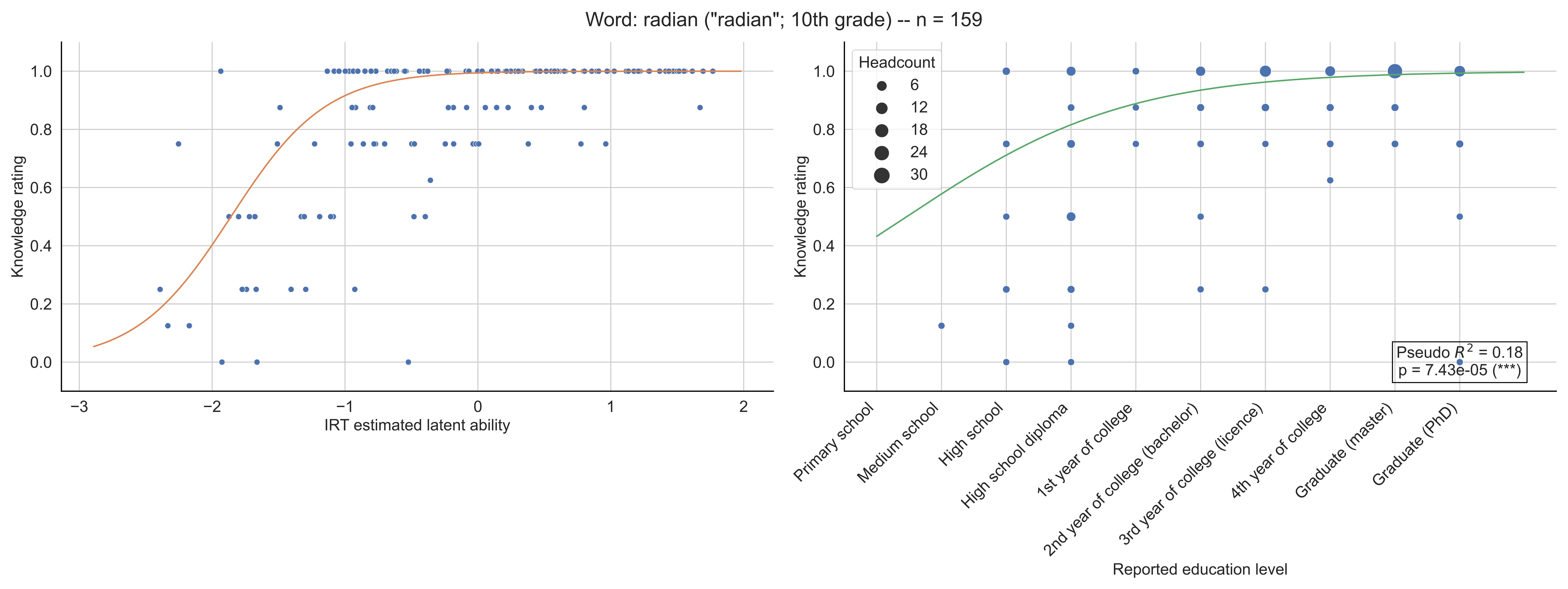

Word: radian ("radian"; 10th grade) -- n = 159
Knowledge rating
IRT estimated latent ability
Headcount
6
12
18
24
30
Pseudo R^2 = 0.18
p = 7.43e-05 (***)
Primary school
Medium school
High school
High school diploma
1st year of college
2nd year of college (bachelor)
3rd year of college (licence)
4th year of college
Graduate (master)
Graduate (PhD)
Reported education level

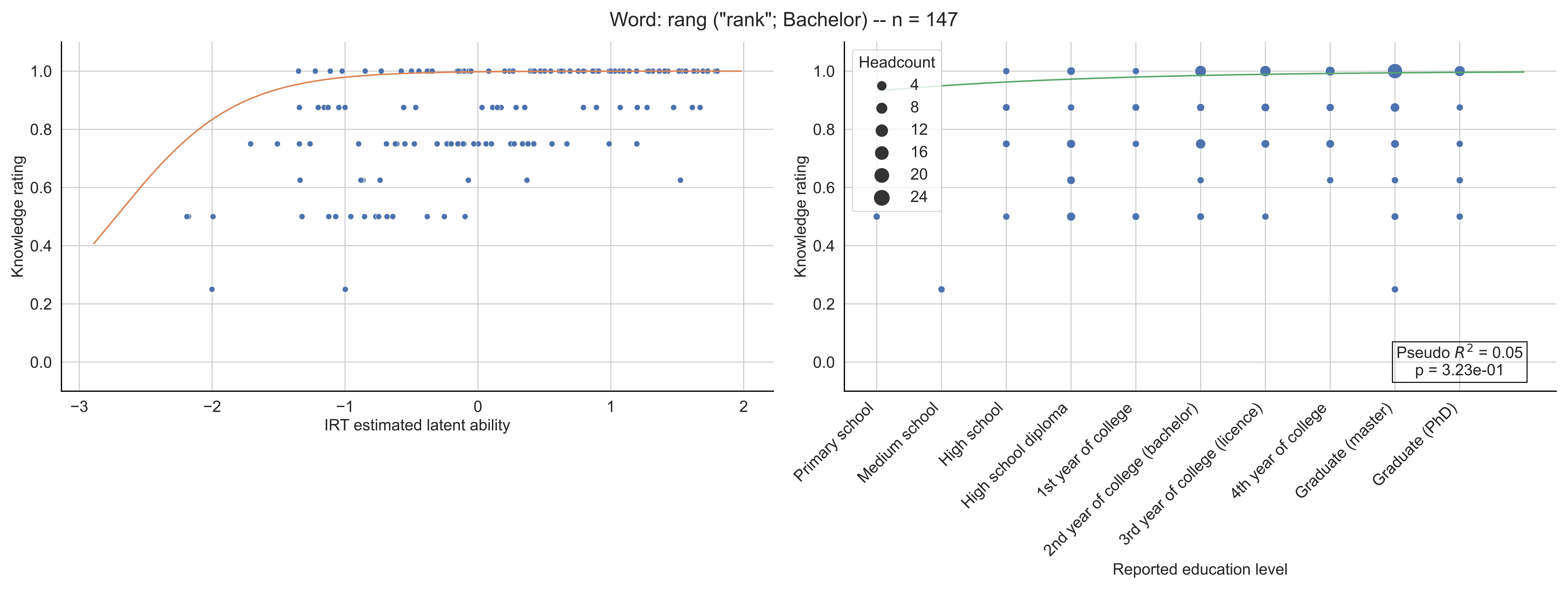

Word: rang ("rank"; Bachelor) -- n = 147
Knowledge rating
1.0
0.8
0.6
0.4
0.2
0.0
−3
−2
−1
0
1
2
IRT estimated latent ability
Headcount
4
8
12
16
20
24
Pseudo R^2 = 0.05
p = 3.23e-01
Primary school
Medium school
High school
High school diploma
1st year of college
2nd year of college (bachelor)
3rd year of college (licence)
4th year of college
Graduate (master)
Graduate (PhD)
Reported education level

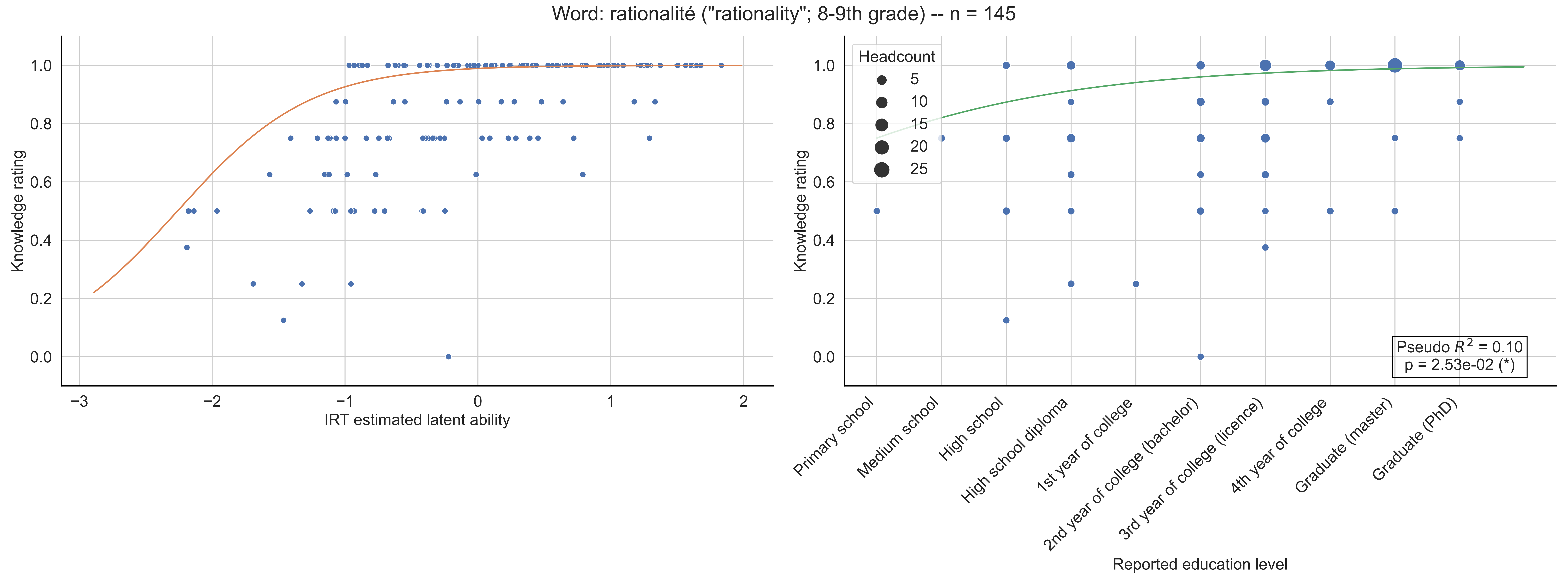

Word: rationalité ("rationality"; 8-9th grade) -- n = 145
Knowledge rating
IRT estimated latent ability
Headcount
5
10
15
20
25
Primary school
Medium school
High school
High school diploma
1st year of college
2nd year of college (bachelor)
3rd year of college (licence)
4th year of college
Graduate (master)
Graduate (PhD)
Reported education level
Pseudo R^2 = 0.10
p = 2.53e-02 (*)

Word: rationnel ("rationnal"; 10th grade) -- n = 153

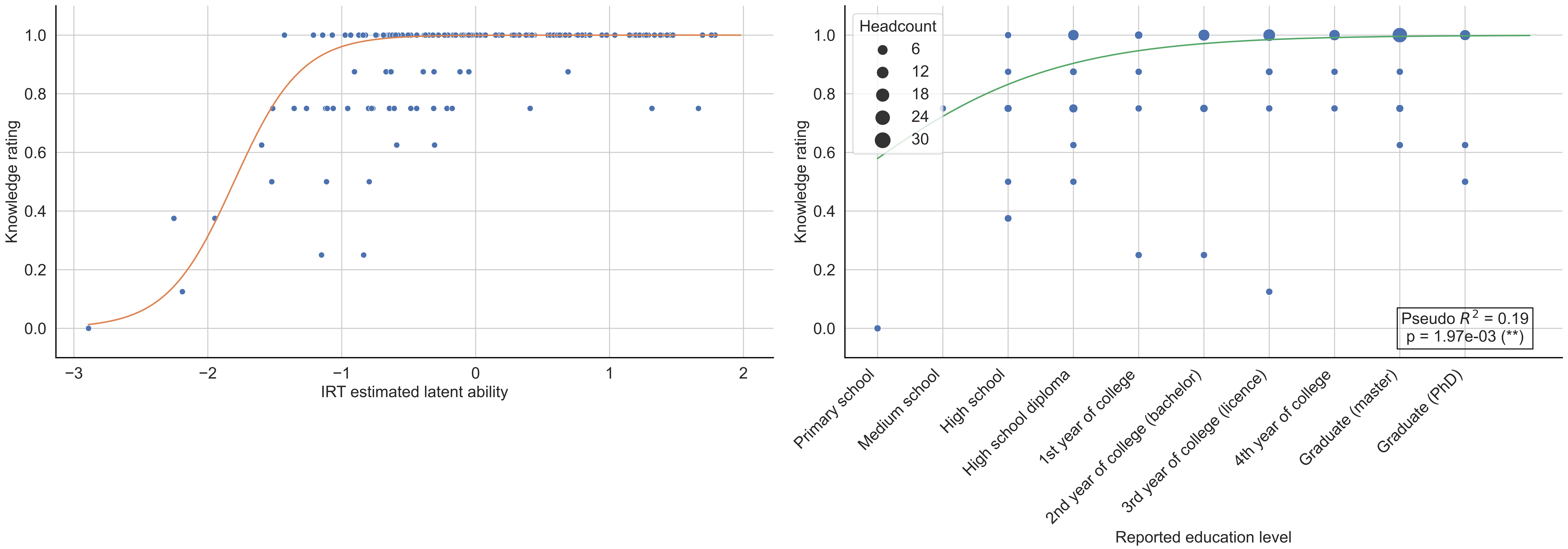

Word: relatif (6-7th grade) -- n = 150

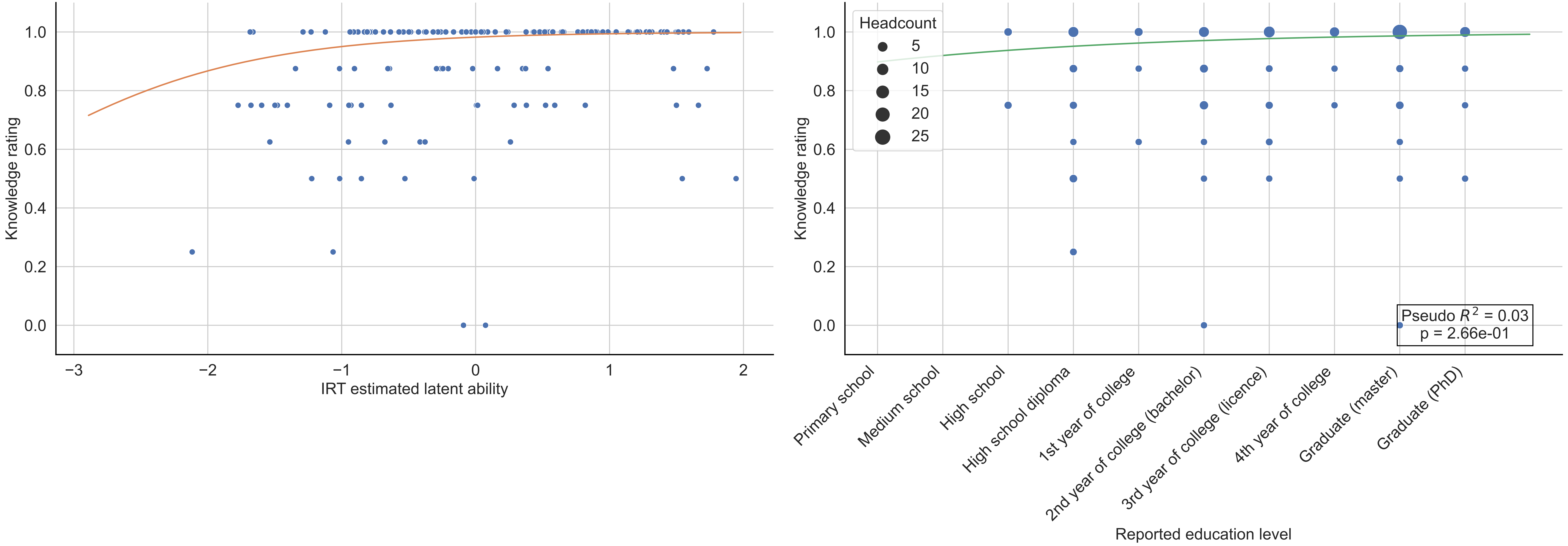

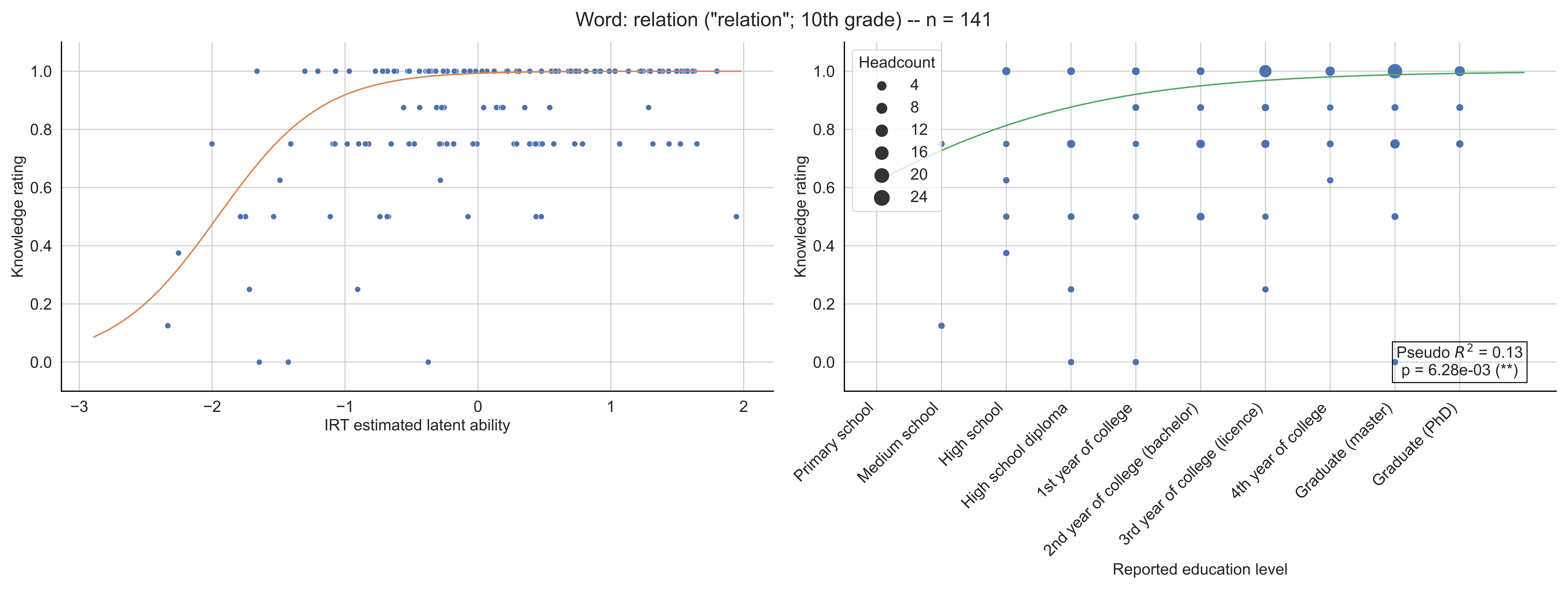

Word: relation ("relation"; 10th grade) -- n = 141
Knowledge rating
IRT estimated latent ability
Headcount
4
8
12
16
20
24
Primary school
Medium school
High school
High school diploma
1st year of college
2nd year of college (bachelor)
3rd year of college (licence)
4th year of college
Graduate (master)
Graduate (PhD)
Reported education level
Pseudo R^2 = 0.13
p = 6.28e-03 (**)

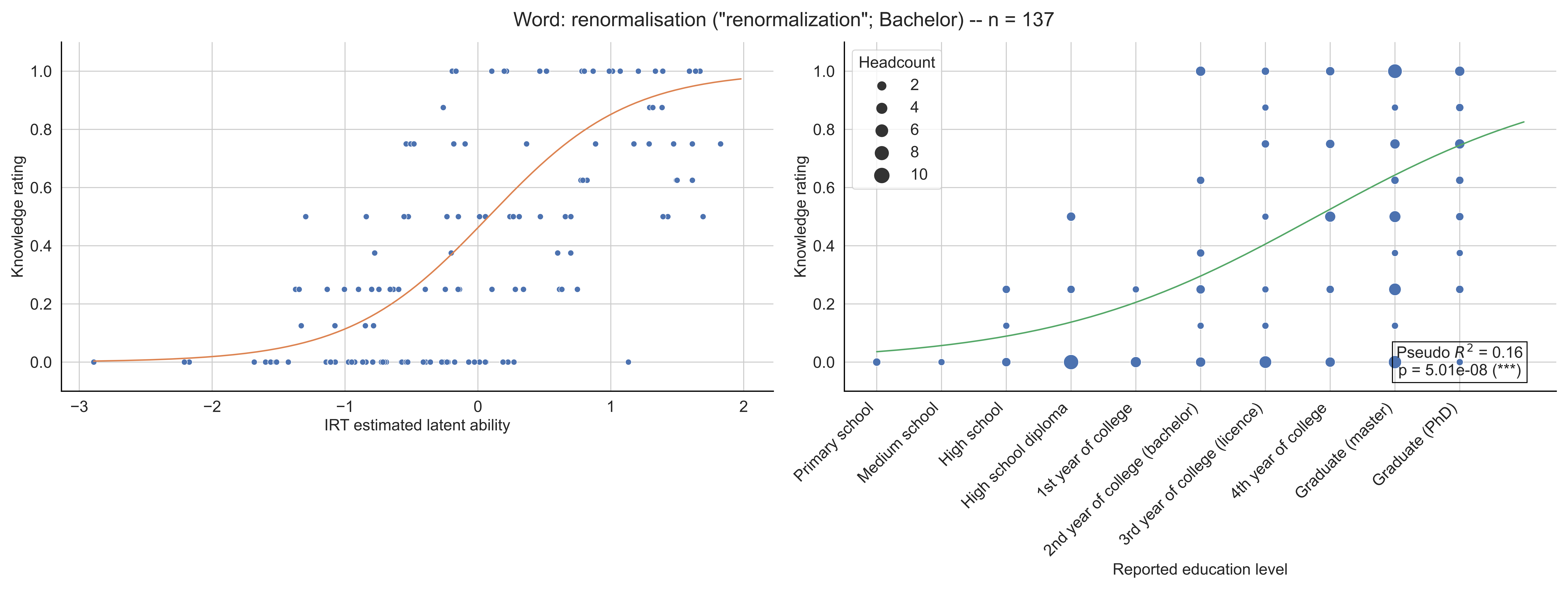
Word: renormalisation ("renormalization"; Bachelor) -- n = 137
Knowledge rating
IRT estimated latent ability
Headcount
2
4
6
8
10
Pseudo R^2 = 0.16
p = 5.01e-08 (***)
Primary school
Medium school
High school
High school diploma
1st year of college
2nd year of college (bachelor)
3rd year of college (licence)
4th year of college
Graduate (master)
Graduate (PhD)
Reported education level

Word: représentation ("representation"; 8-9th grade) -- n = 148

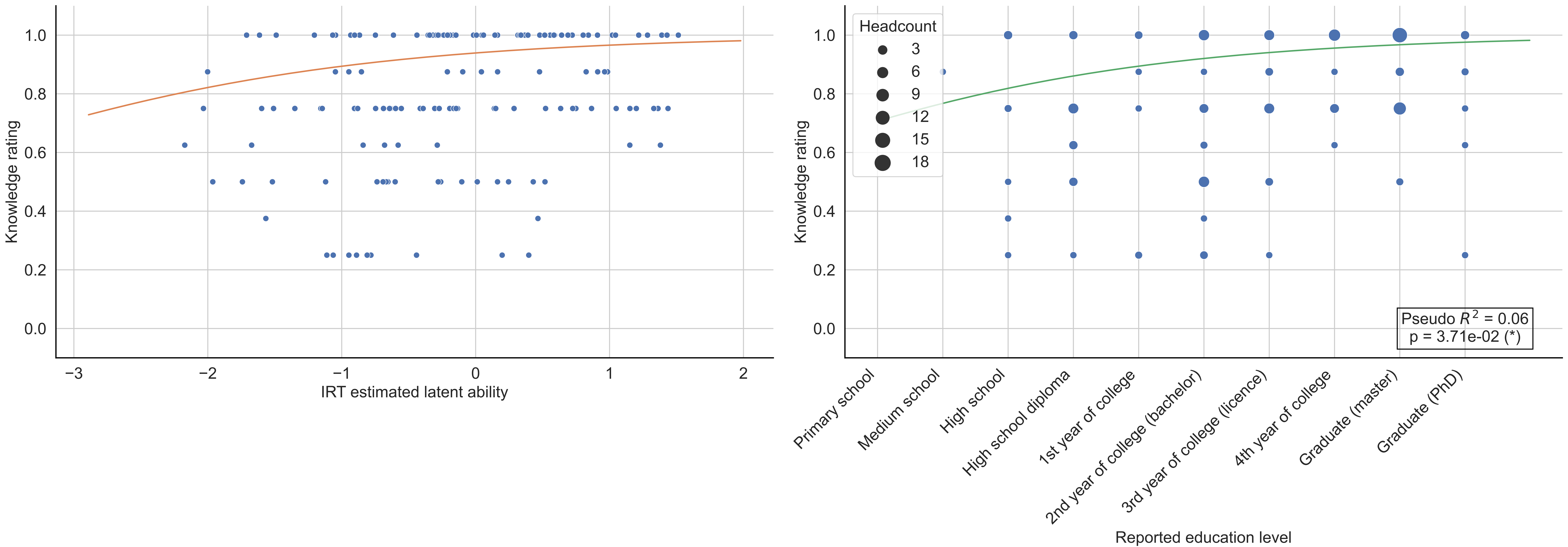

Word: reste ("remainder"; Primary school) -- n = 141

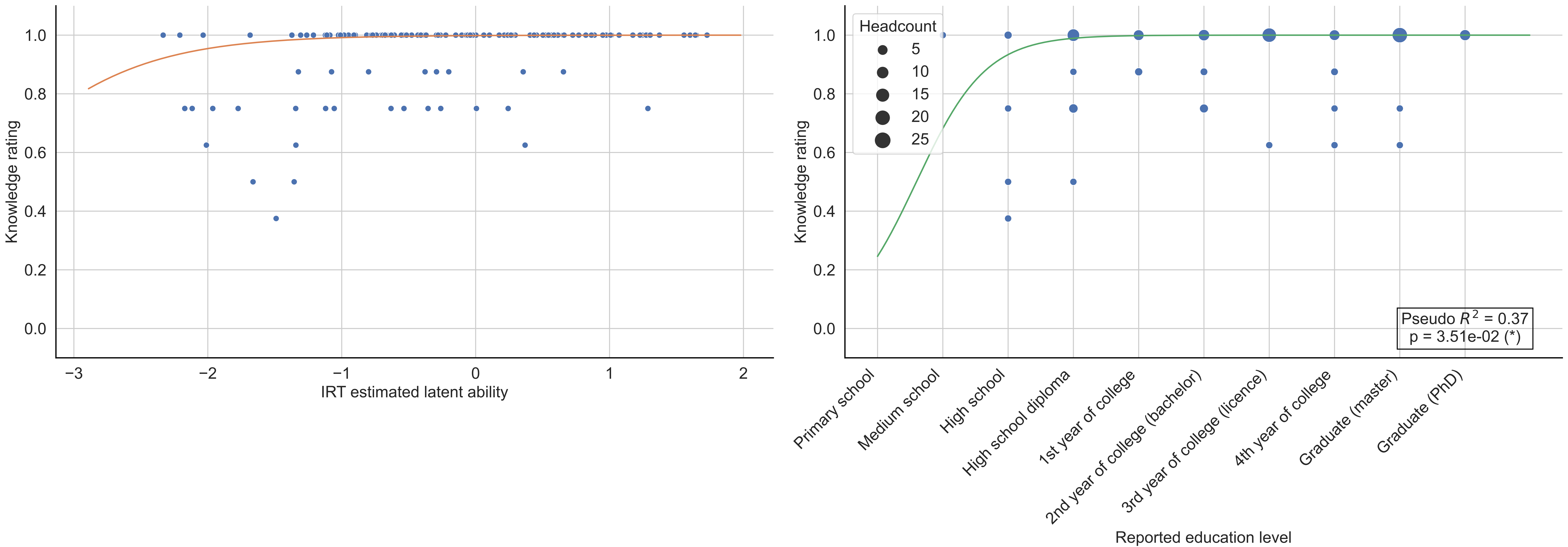

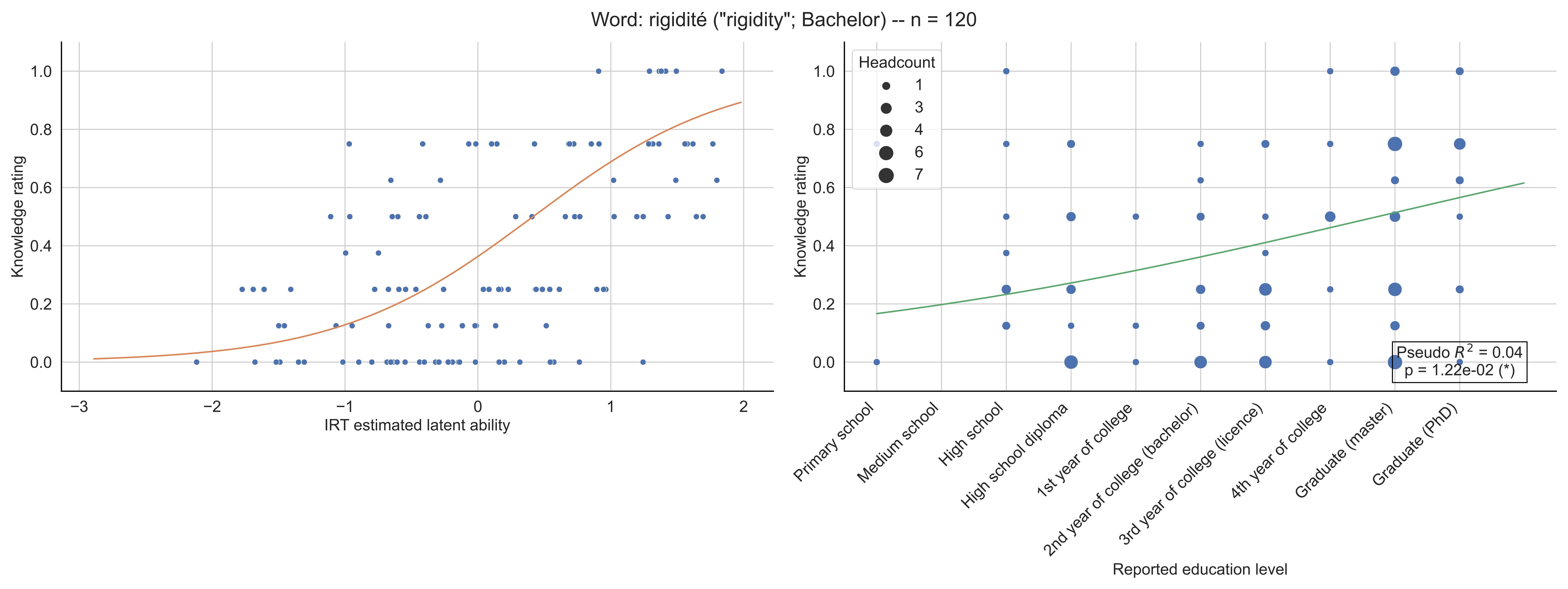

Word: rigidité ("rigidity"; Bachelor) -- n = 120
Knowledge rating
IRT estimated latent ability
Headcount
1
3
4
6
7
Pseudo R^2 = 0.04
p = 1.22e-02 (*)
Primary school
Medium school
High school
High school diploma
1st year of college
2nd year of college (bachelor)
3rd year of college (licence)
4th year of college
Graduate (master)
Graduate (PhD)
Reported education level

Word: rotation ("rotation"; 6-7th grade) -- n = 146

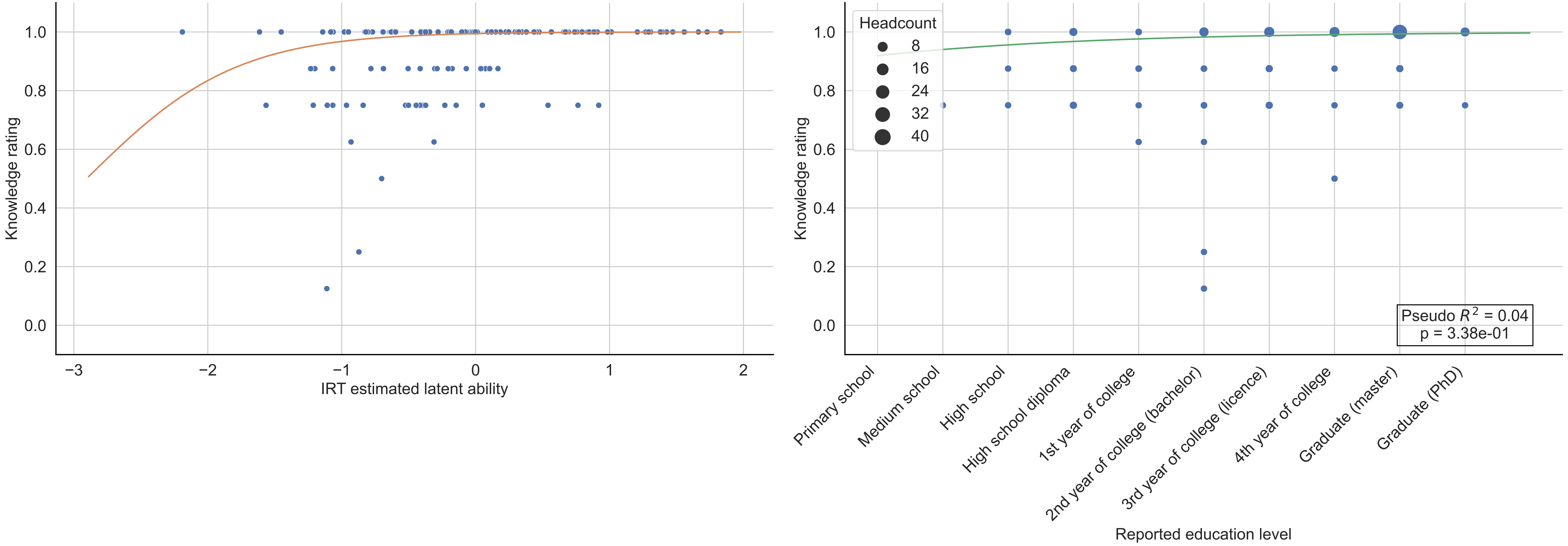

Word: réciprocité ("reciprocity"; Licence) -- n = 162

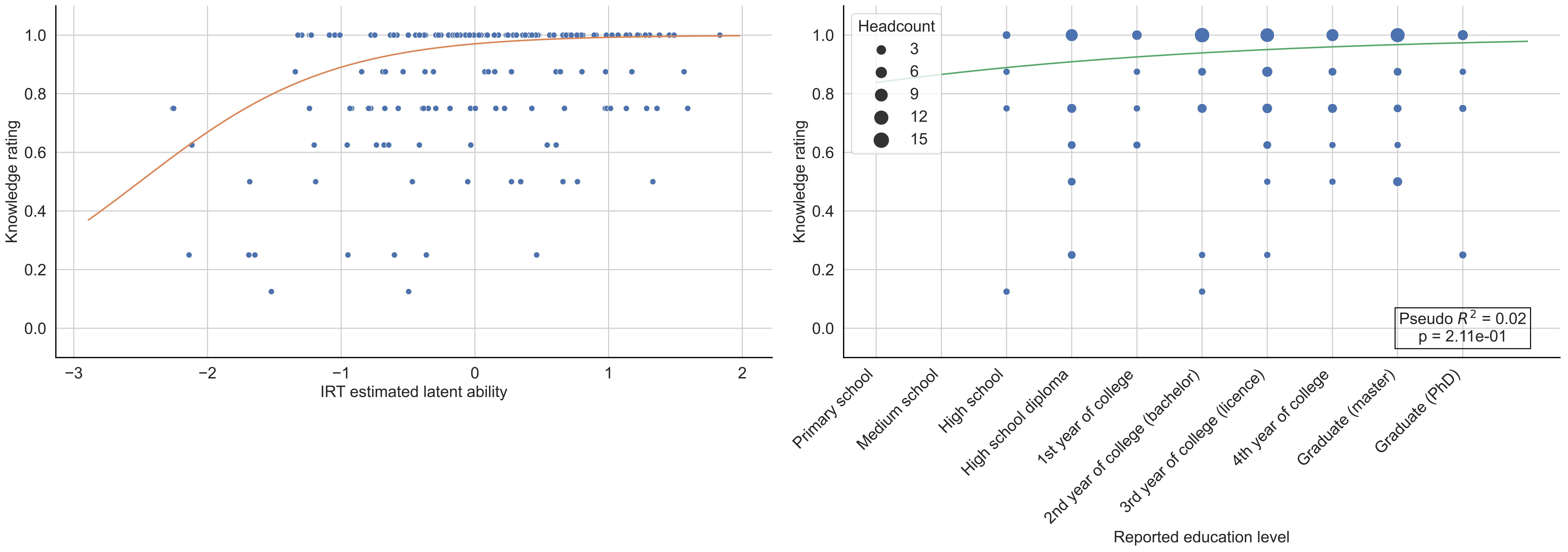

Word: réciproque ("reciprocal"; 8-9th grade) -- n = 150

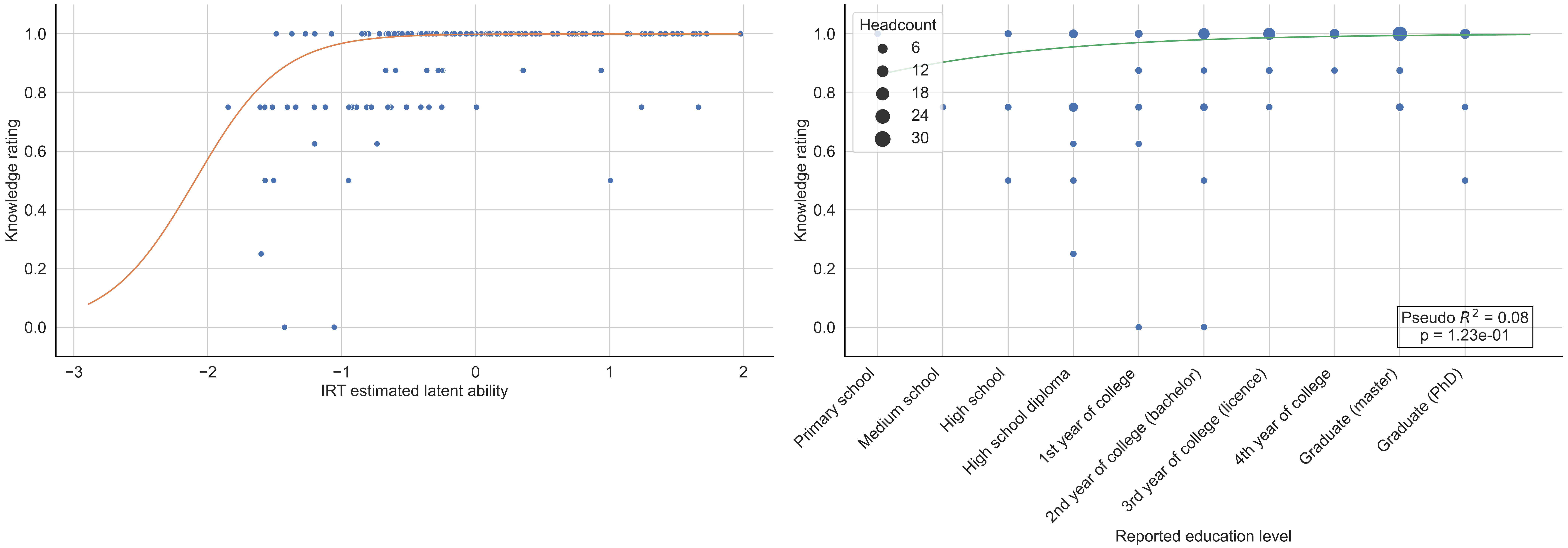

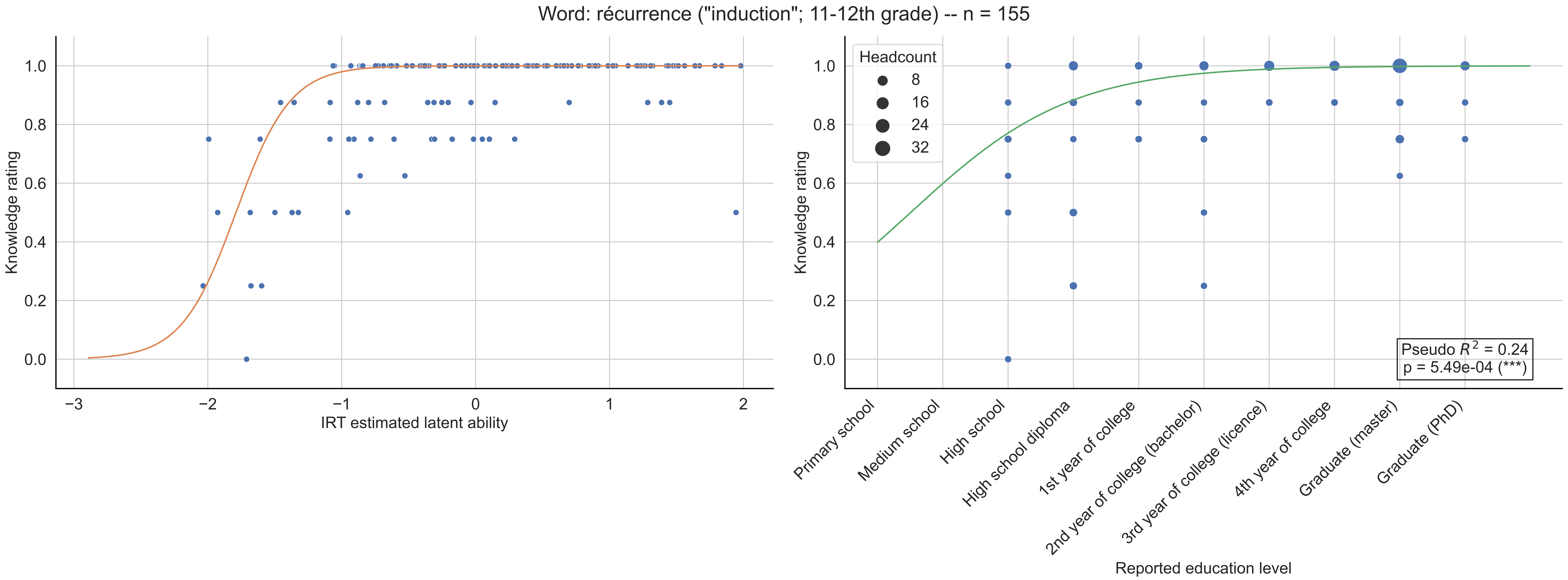

Word: récurrence ("induction"; 11-12th grade) -- n = 155
Knowledge rating
IRT estimated latent ability
−3
−2
−1
0
1
2
0.0
0.2
0.4
0.6
0.8
1.0
Headcount
8
16
24
32
Pseudo R^2 = 0.24
p = 5.49e-04 (***)
Primary school
Medium school
High school
High school diploma
1st year of college
2nd year of college (bachelor)
3rd year of college (licence)
4th year of college
Graduate (master)
Graduate (PhD)
Reported education level

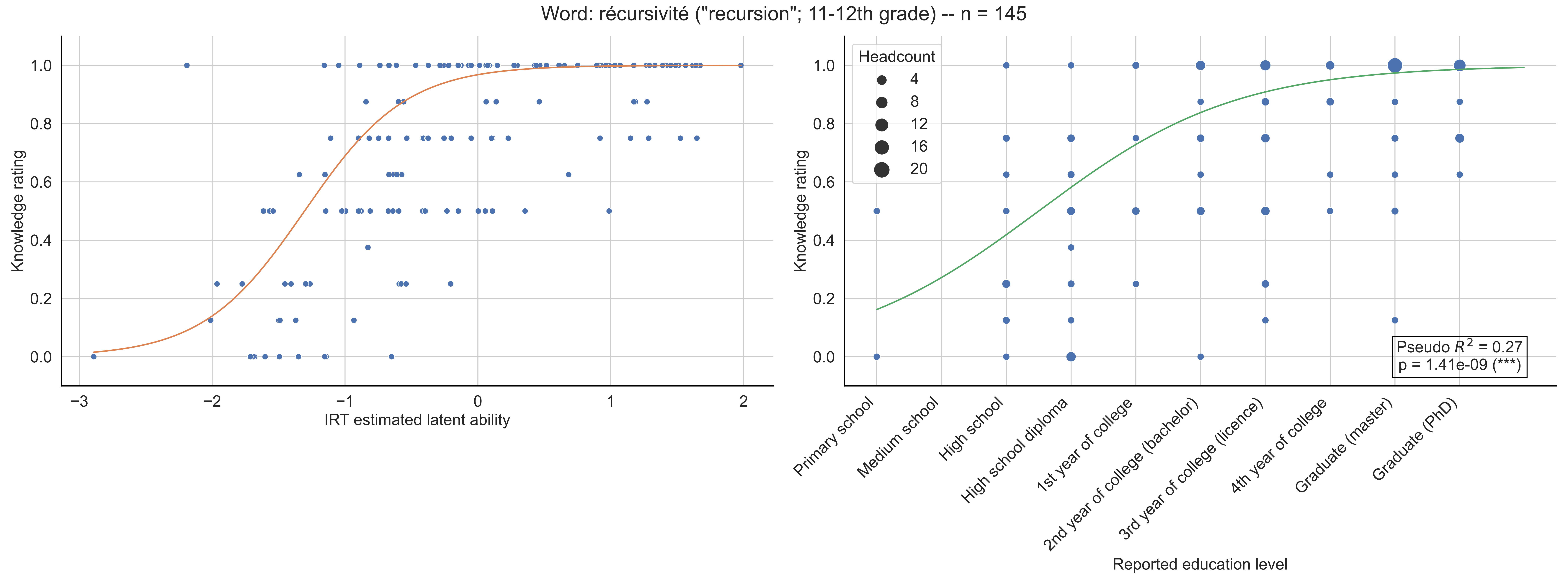

Word: récursivité ("recursion"; 11-12th grade) -- n = 145
Knowledge rating
IRT estimated latent ability
Headcount
4
8
12
16
20
Pseudo R^2 = 0.27
p = 1.41e-09 (***)
Primary school
Medium school
High school
High school diploma
1st year of college
2nd year of college (bachelor)
3rd year of college (licence)
4th year of college
Graduate (master)
Graduate (PhD)
Reported education level

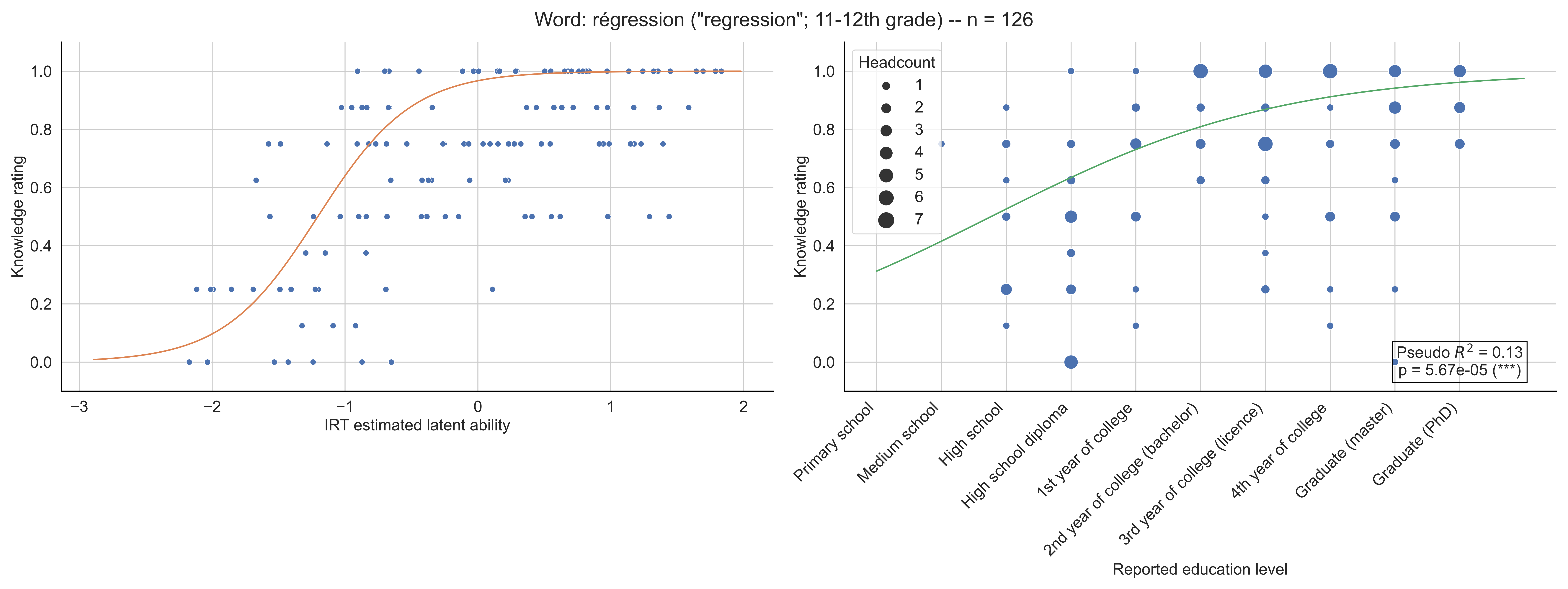

Word: régression ("regression"; 11-12th grade) -- n = 126
Knowledge rating
IRT estimated latent ability
Headcount
1
2
3
4
5
6
7
Pseudo R^2 = 0.13
p = 5.67e-05 (***)
Primary school
Medium school
High school
High school diploma
1st year of college
2nd year of college (bachelor)
3rd year of college (licence)
4th year of college
Graduate (master)
Graduate (PhD)
Reported education level

Word: résidu ("residue"; Licence) -- n = 140

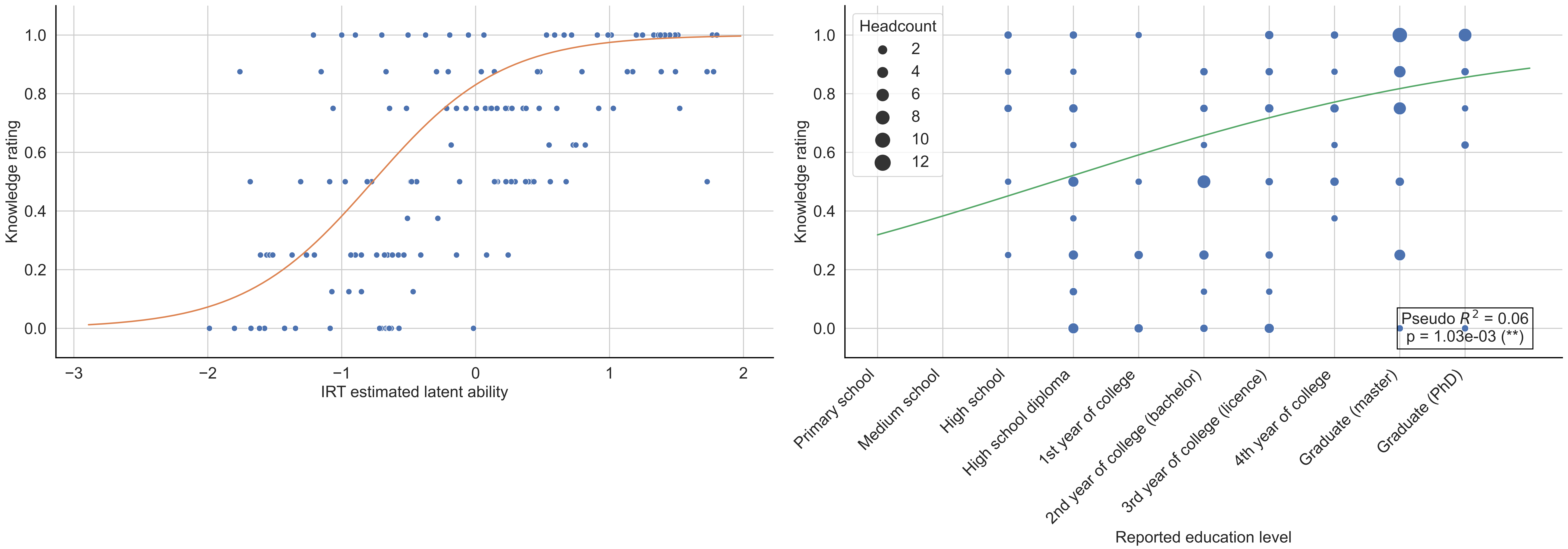

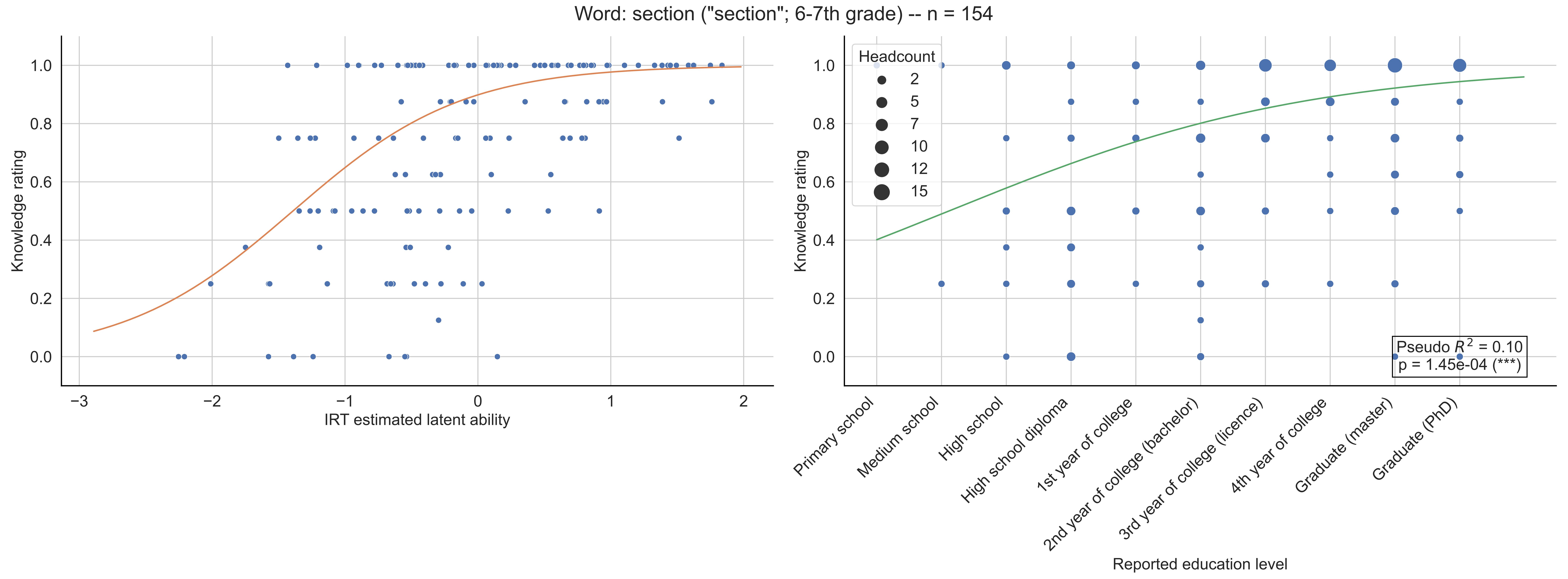

Word: section ("section"; 6-7th grade) -- n = 154
Knowledge rating
IRT estimated latent ability
Headcount
2
5
7
10
12
15
Pseudo R^2 = 0.10
p = 1.45e-04 (***)
Primary school
Medium school
High school
High school diploma
1st year of college
2nd year of college (bachelor)
3rd year of college (licence)
4th year of college
Graduate (master)
Graduate (PhD)
Reported education level

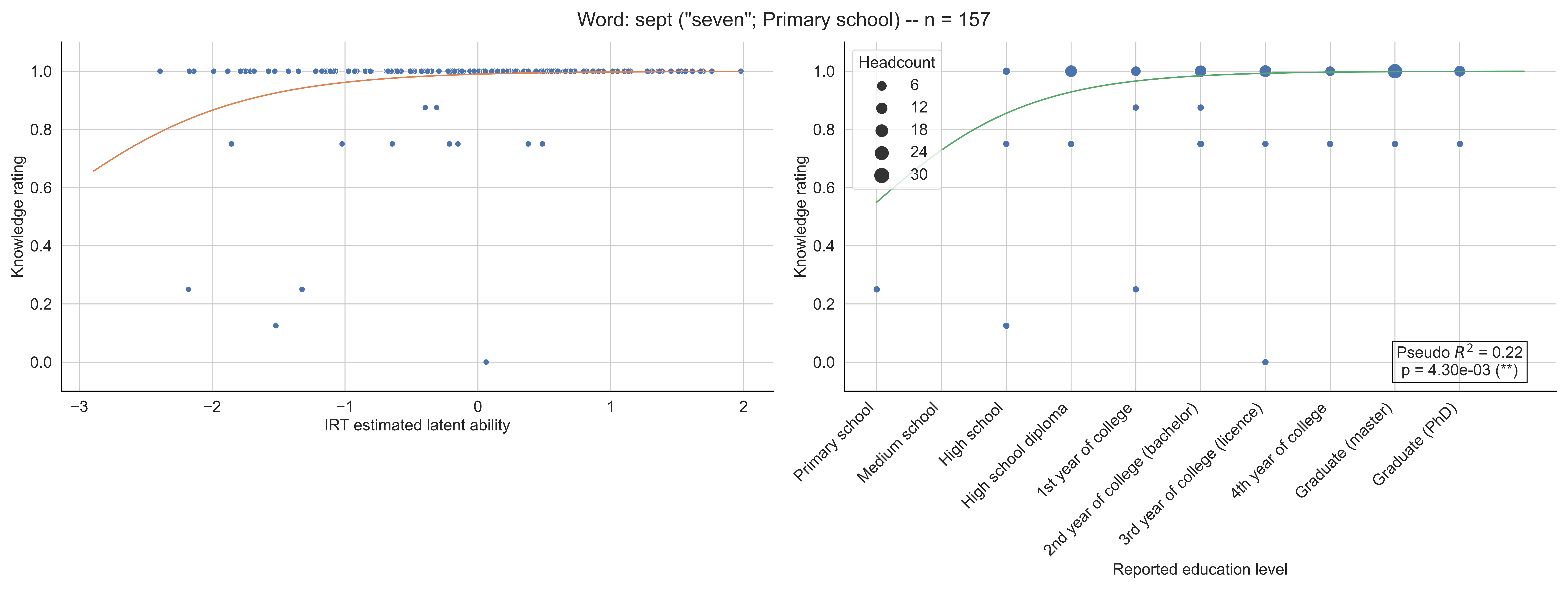

Word: sept ("seven"; Primary school) -- n = 157
Knowledge rating
IRT estimated latent ability
Headcount
6
12
18
24
30
Pseudo R^2 = 0.22
p = 4.30e-03 (**)
Primary school
Medium school
High school
High school diploma
1st year of college
2nd year of college (bachelor)
3rd year of college (licence)
4th year of college
Graduate (master)
Graduate (PhD)
Reported education level

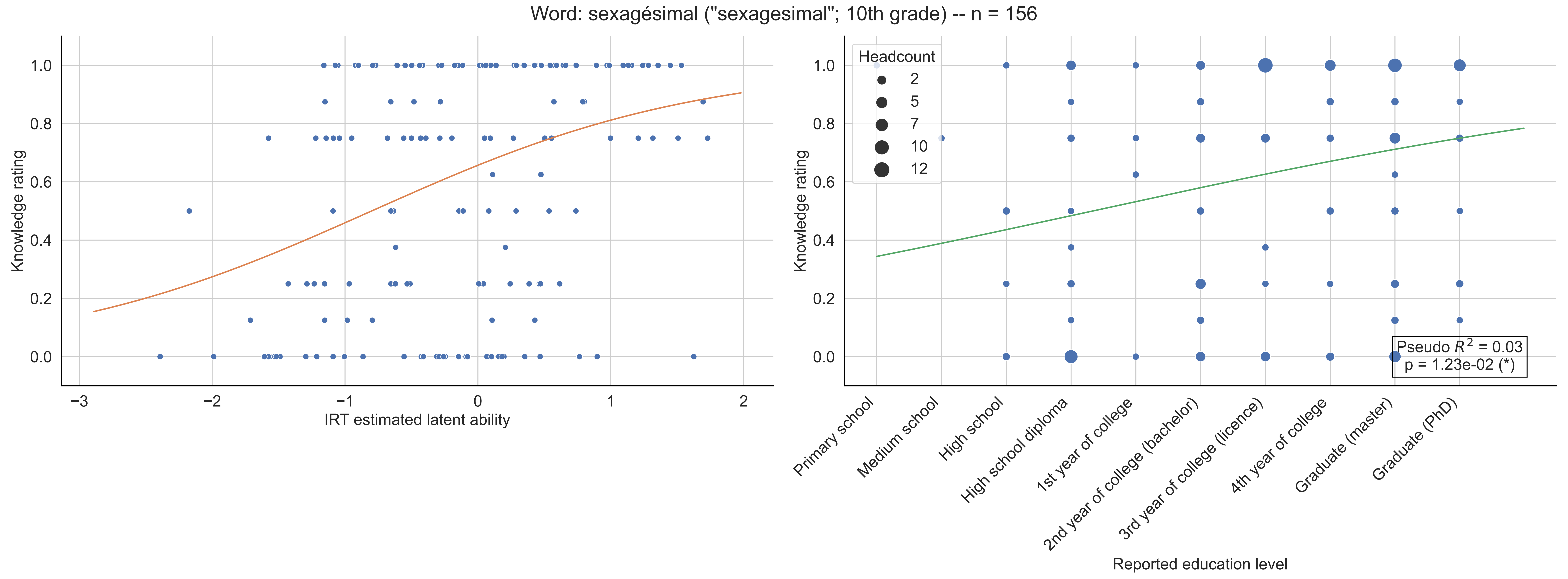

Word: sexagésimal ("sexagesimal"; 10th grade) -- n = 156
Knowledge rating
1.0
0.8
0.6
0.4
0.2
0.0
−3
−2
−1
0
1
2
IRT estimated latent ability
Headcount
2
5
7
10
12
Pseudo R^2 = 0.03
p = 1.23e-02 (*)
Primary school
Medium school
High school
High school diploma
1st year of college
2nd year of college (bachelor)
3rd year of college (licence)
4th year of college
Graduate (master)
Graduate (PhD)
Reported education level

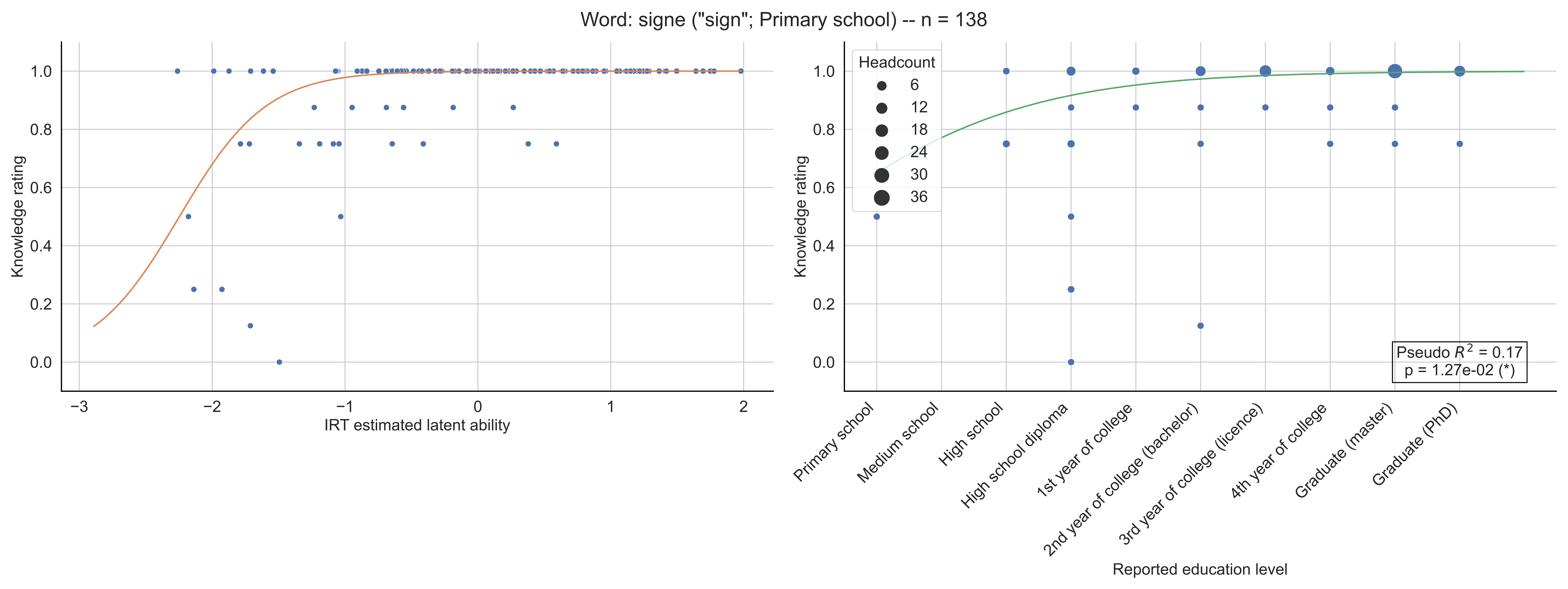

Word: signe ("sign"; Primary school) -- n = 138
Knowledge rating
IRT estimated latent ability
Headcount
6
12
18
24
30
36
Pseudo R^2 = 0.17
p = 1.27e-02 (*)
Primary school
Medium school
High school
High school diploma
1st year of college
2nd year of college (bachelor)
3rd year of college (licence)
4th year of college
Graduate (master)
Graduate (PhD)
Reported education level

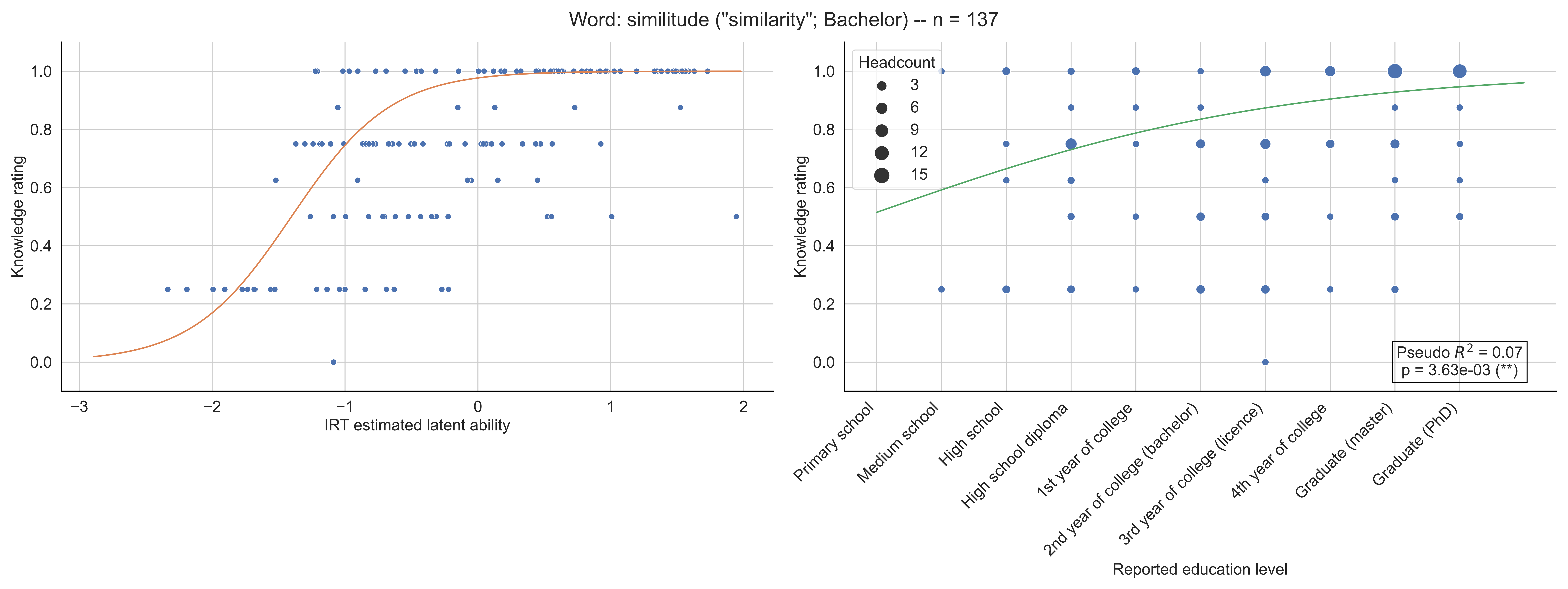
Word: similitude ("similarity"; Bachelor) -- n = 137
Knowledge rating
IRT estimated latent ability
Headcount
3
6
9
12
15
Pseudo R^2 = 0.07
p = 3.63e-03 (**)
Primary school
Medium school
High school
High school diploma
1st year of college
2nd year of college (bachelor)
3rd year of college (licence)
4th year of college
Graduate (master)
Graduate (PhD)
Reported education level

Word: simplexe ("simplex"; Licence) -- n = 155

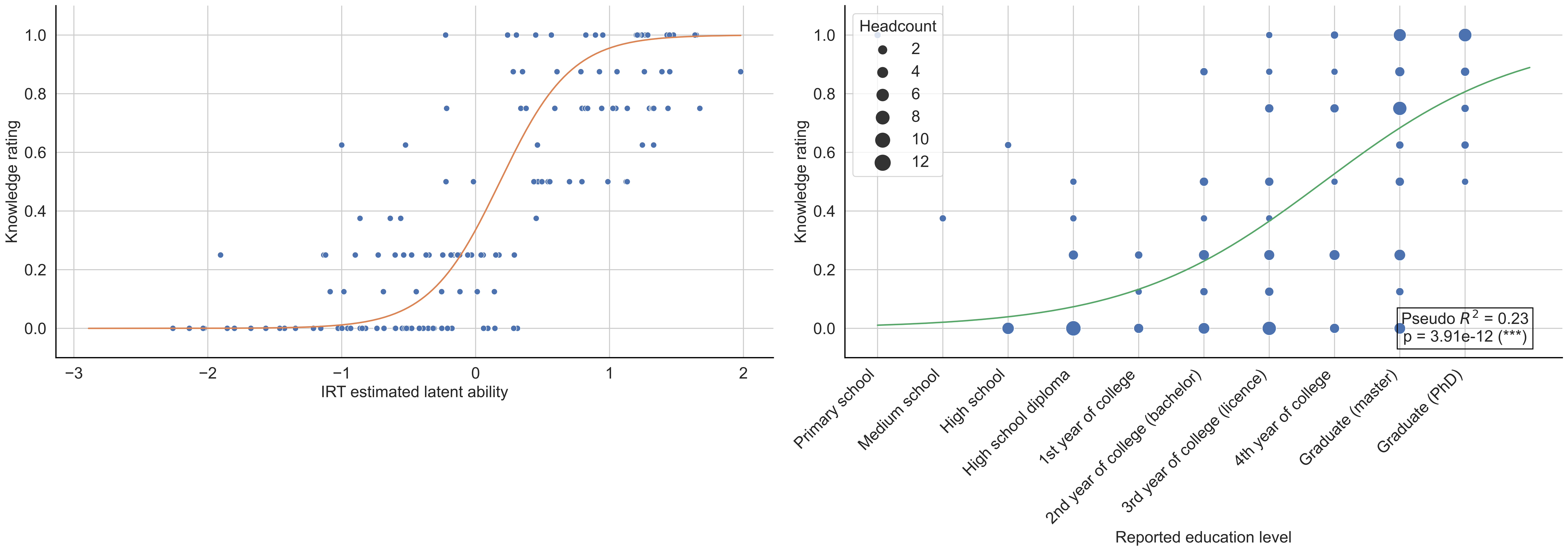

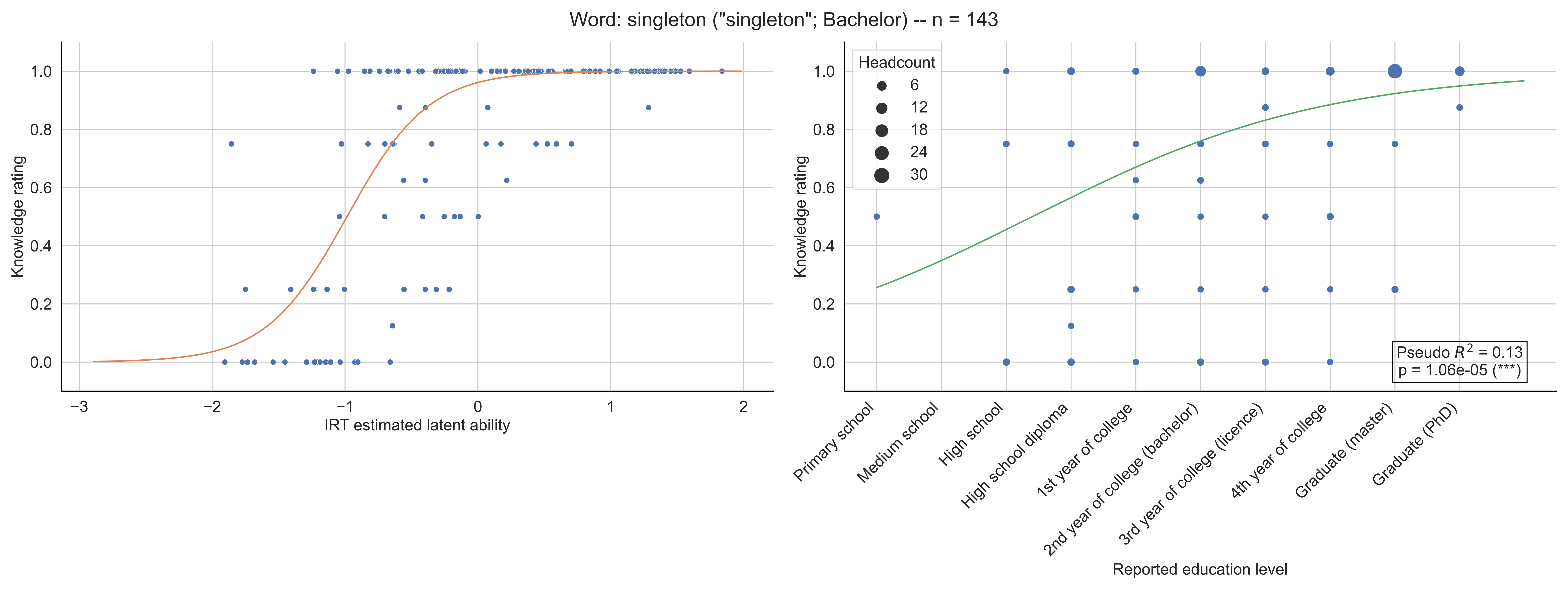

Word: singleton ("singleton"; Bachelor) -- n = 143
Knowledge rating
IRT estimated latent ability
Headcount
6
12
18
24
30
Pseudo R^2 = 0.13
p = 1.06e-05 (***)
Primary school
Medium school
High school
High school diploma
1st year of college
2nd year of college (bachelor)
3rd year of college (licence)
4th year of college
Graduate (master)
Graduate (PhD)
Reported education level

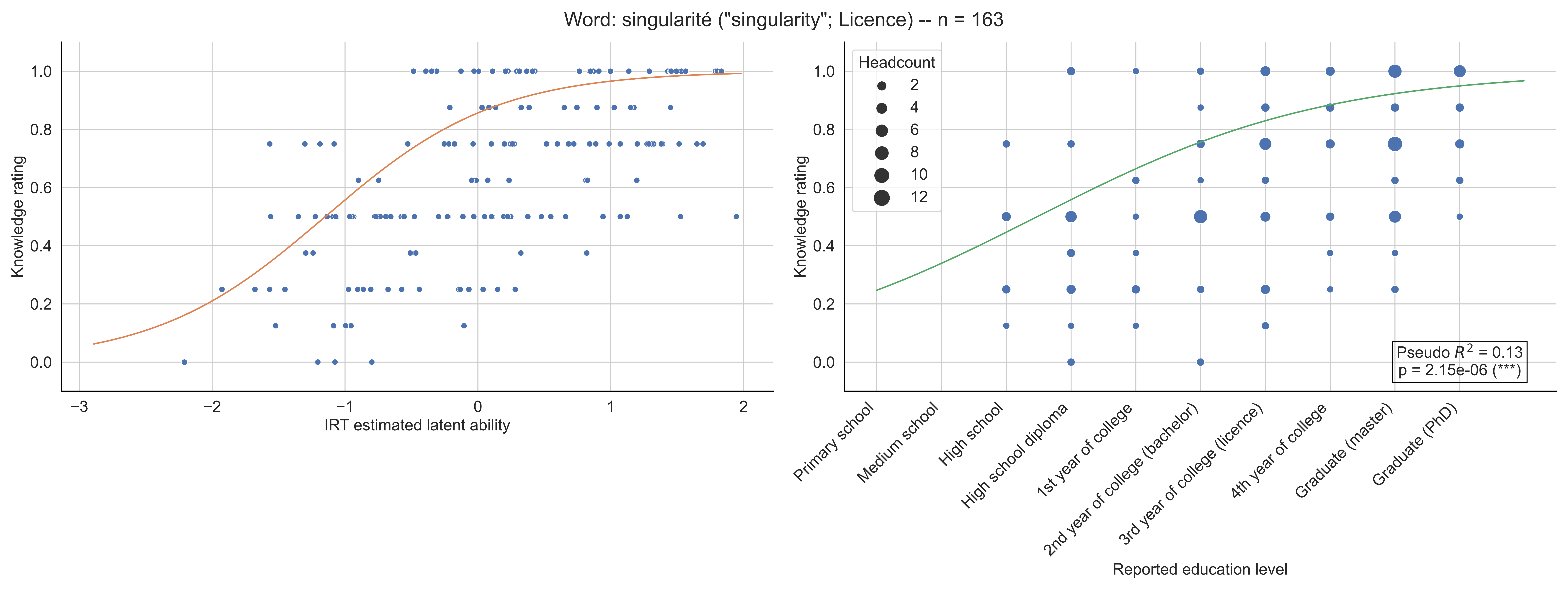

Word: singularité ("singularity"; Licence) -- n = 163
Knowledge rating
IRT estimated latent ability
Headcount
2
4
6
8
10
12
Pseudo R^2 = 0.13
p = 2.15e-06 (***)
Primary school
Medium school
High school
High school diploma
1st year of college
2nd year of college (bachelor)
3rd year of college (licence)
4th year of college
Graduate (master)
Graduate (PhD)
Reported education level

Word: sommation ("summation"; Bachelor) -- n = 135

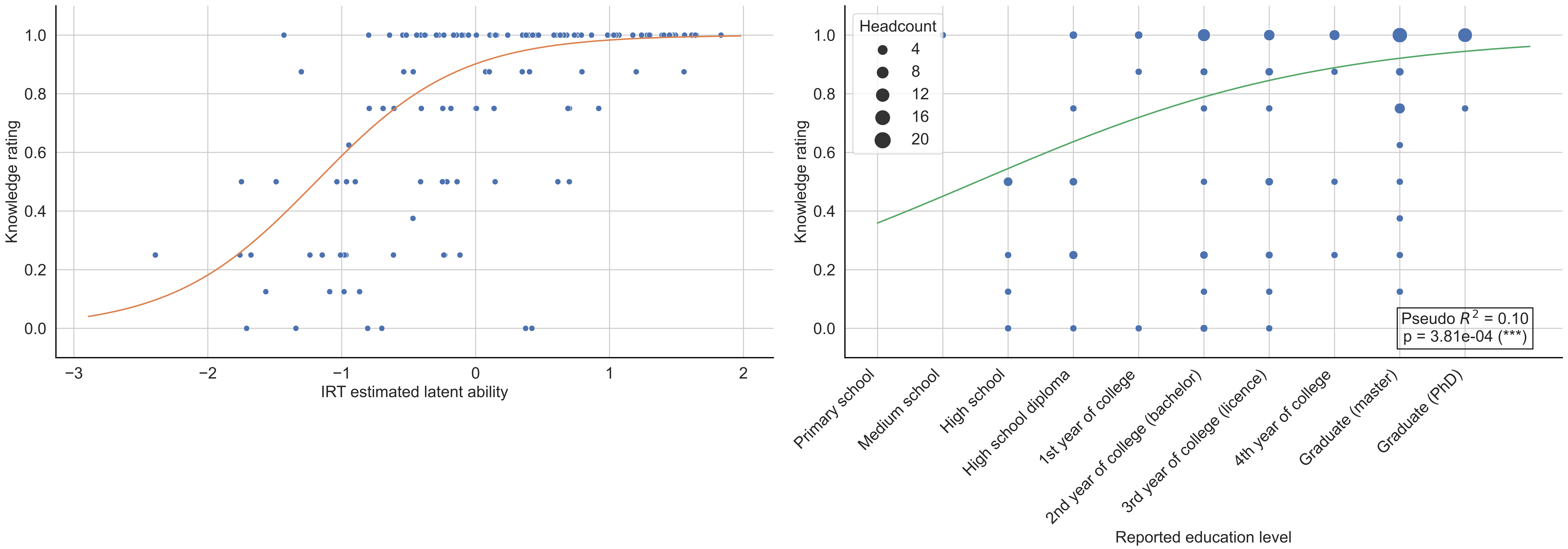

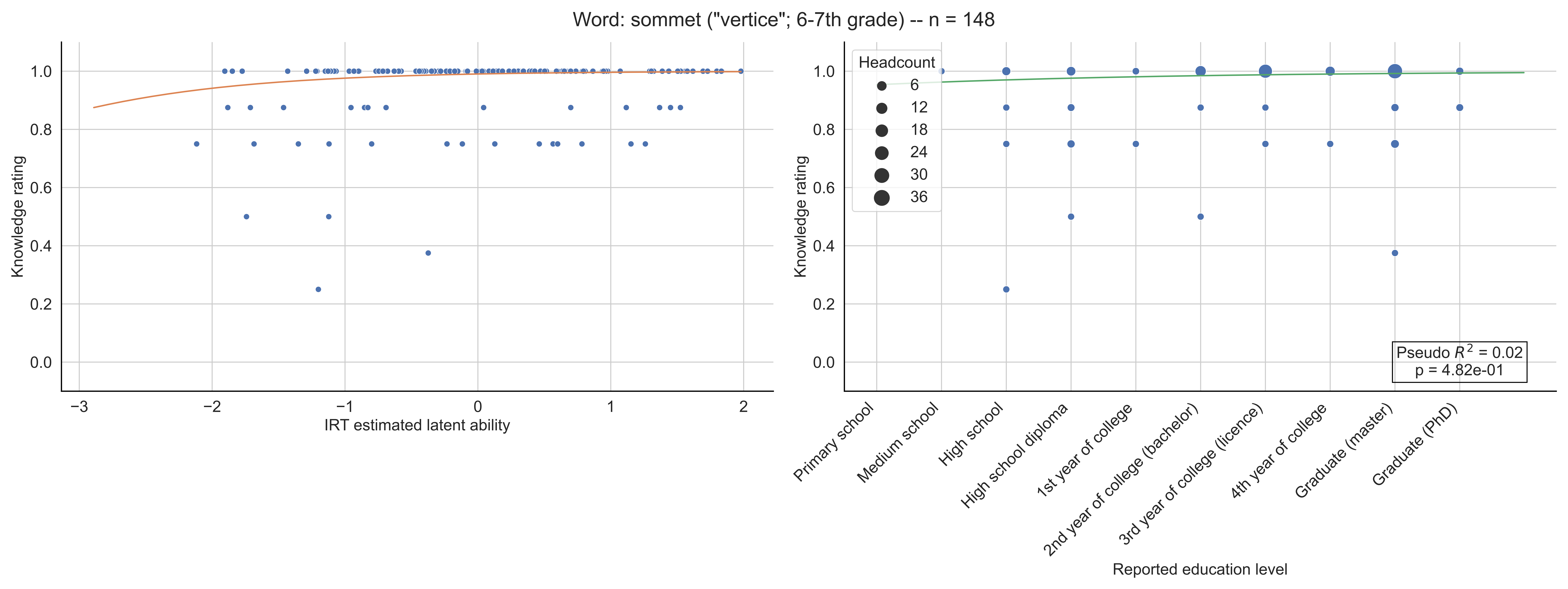

Word: sommet ("vertice"; 6-7th grade) -- n = 148
Knowledge rating
IRT estimated latent ability
Headcount
6
12
18
24
30
36
Pseudo R^2 = 0.02
p = 4.82e-01
Primary school
Medium school
High school
High school diploma
1st year of college
2nd year of college (bachelor)
3rd year of college (licence)
4th year of college
Graduate (master)
Graduate (PhD)
Reported education level

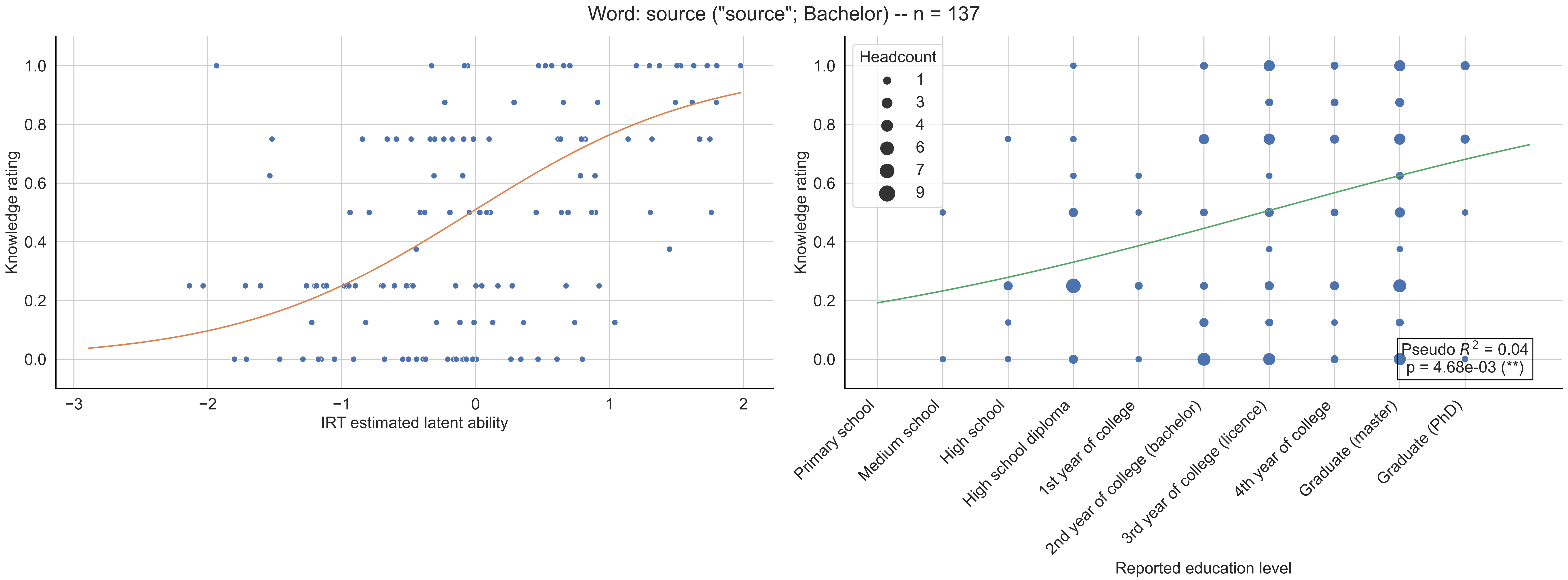
Word: source ("source"; Bachelor) -- n = 137
Knowledge rating
IRT estimated latent ability
Headcount
1
3
4
6
7
9
Pseudo R^2 = 0.04
p = 4.68e-03 (**)
Primary school
Medium school
High school
High school diploma
1st year of college
2nd year of college (bachelor)
3rd year of college (licence)
4th year of college
Graduate (master)
Graduate (PhD)
Reported education level

Word: spectre ("spectrum"; Bachelor) -- n = 149

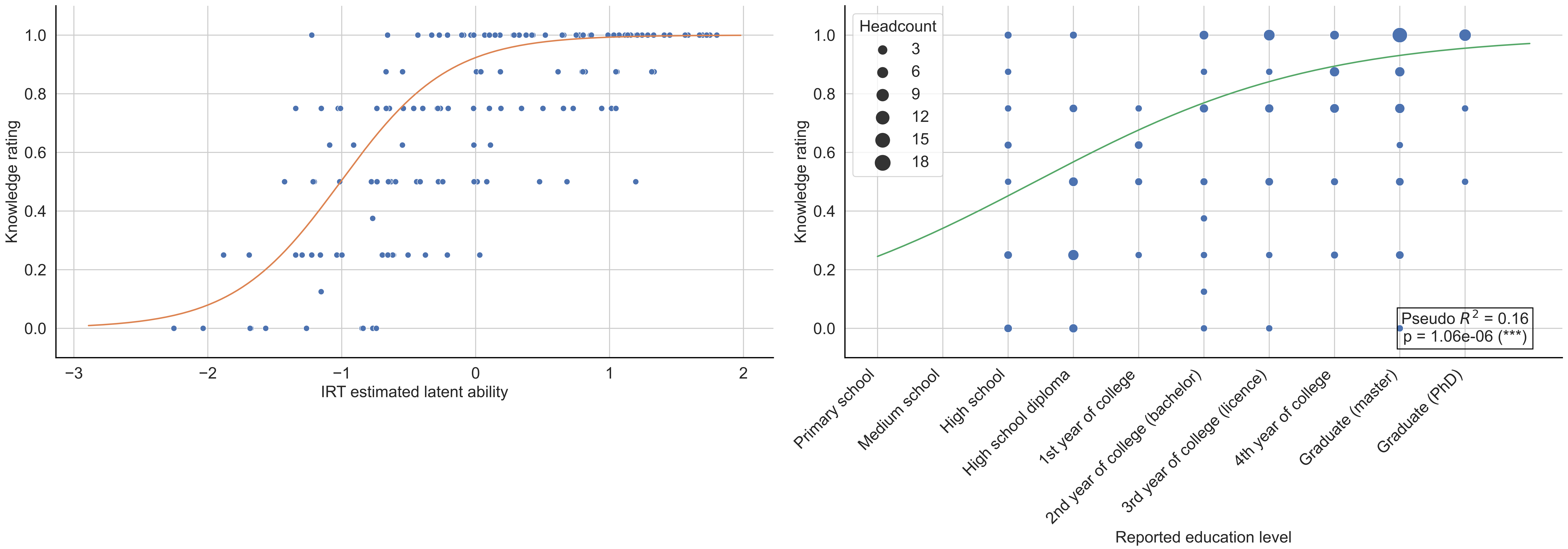

Word: structure ("structure"; Bachelor) -- n = 154

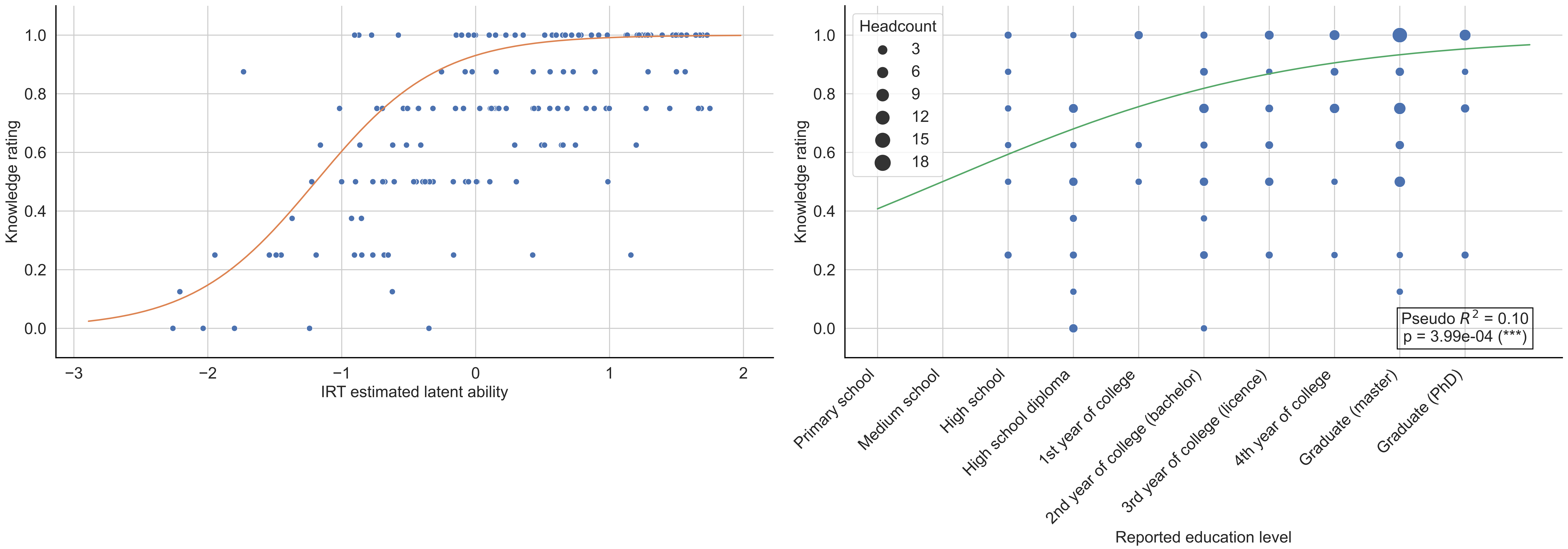

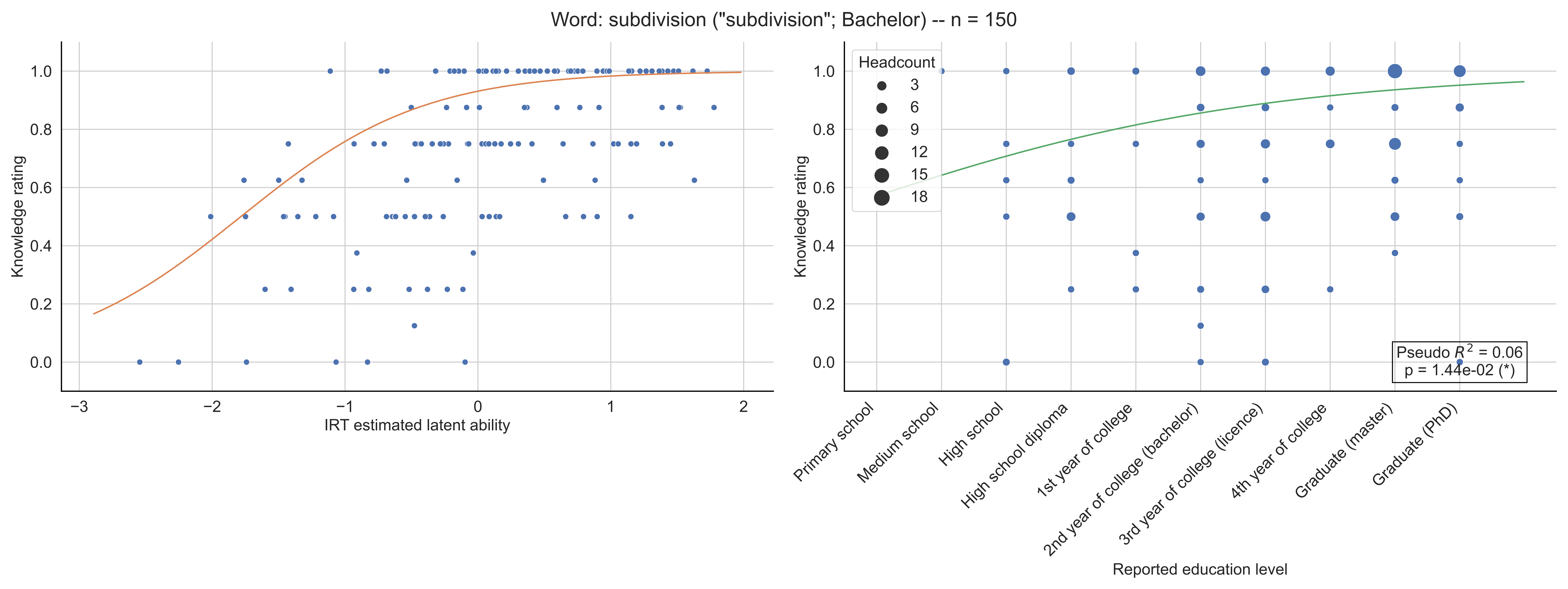
Word: subdivision ("subdivision"; Bachelor) -- n = 150
Knowledge rating
IRT estimated latent ability
Headcount
3
6
9
12
15
18
Pseudo R^2 = 0.06
p = 1.44e-02 (*)
Primary school
Medium school
High school
High school diploma
1st year of college
2nd year of college (bachelor)
3rd year of college (licence)
4th year of college
Graduate (master)
Graduate (PhD)
Reported education level

Word: supplémentaire ("complementary"; Bachelor) -- n = 122

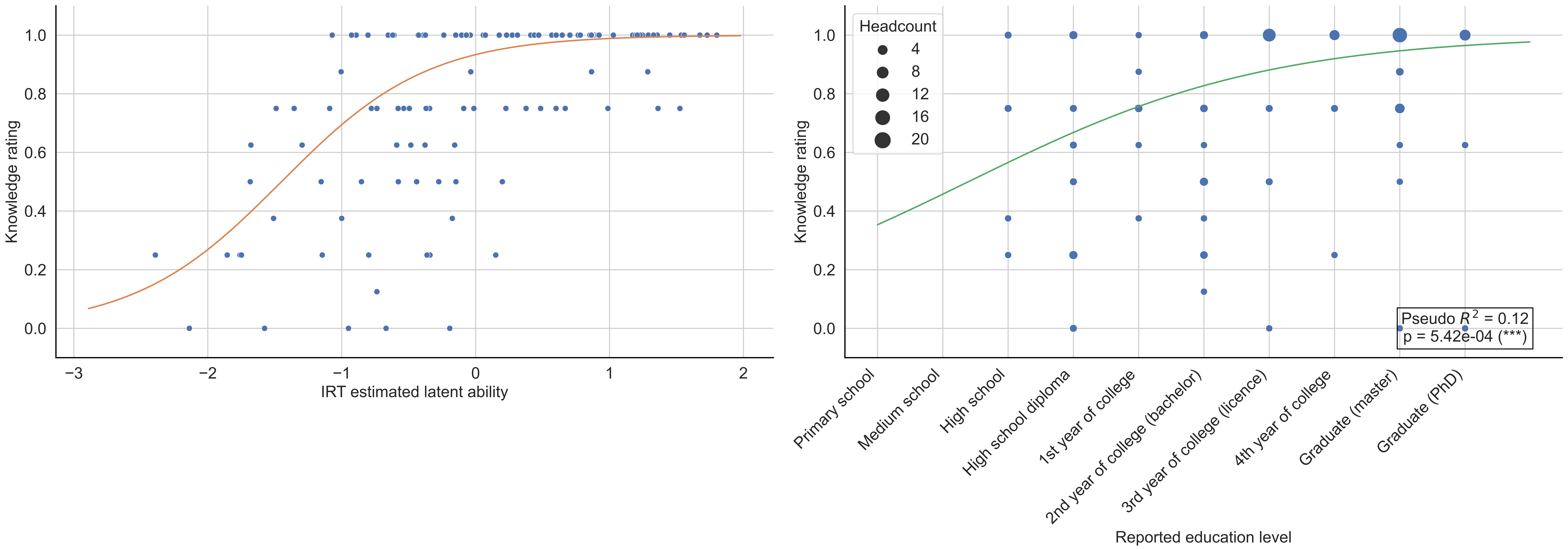

Word: support ("support"; Bachelor) -- n = 142

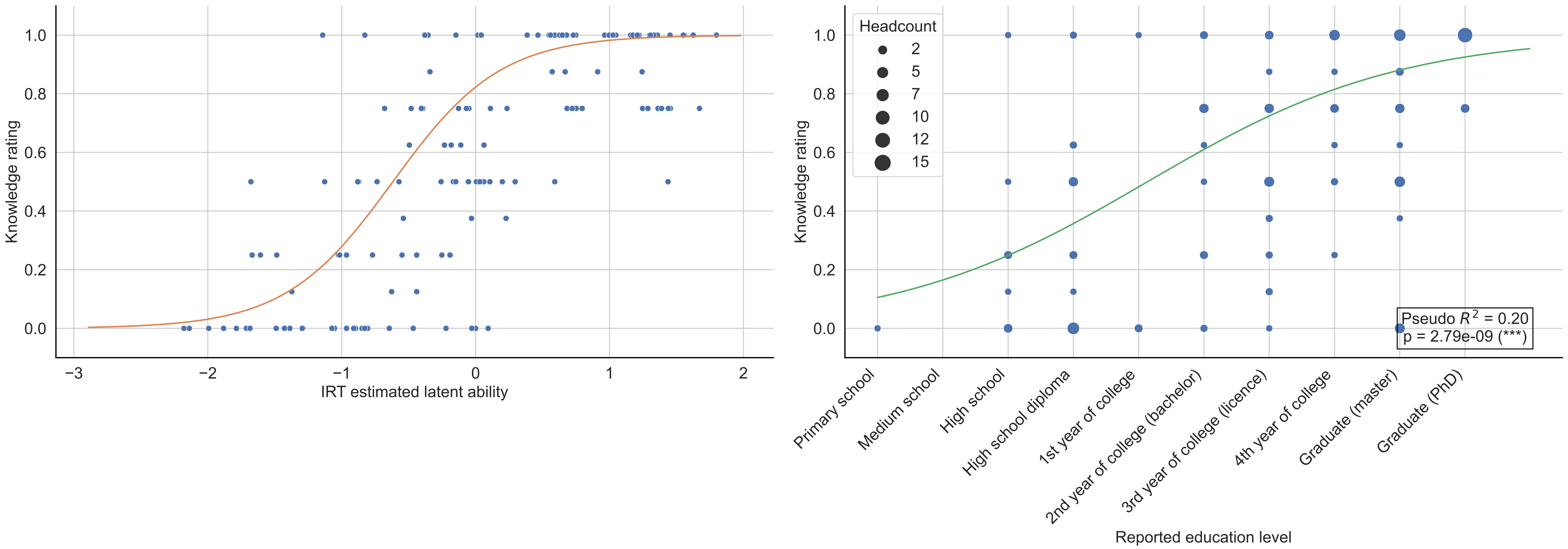

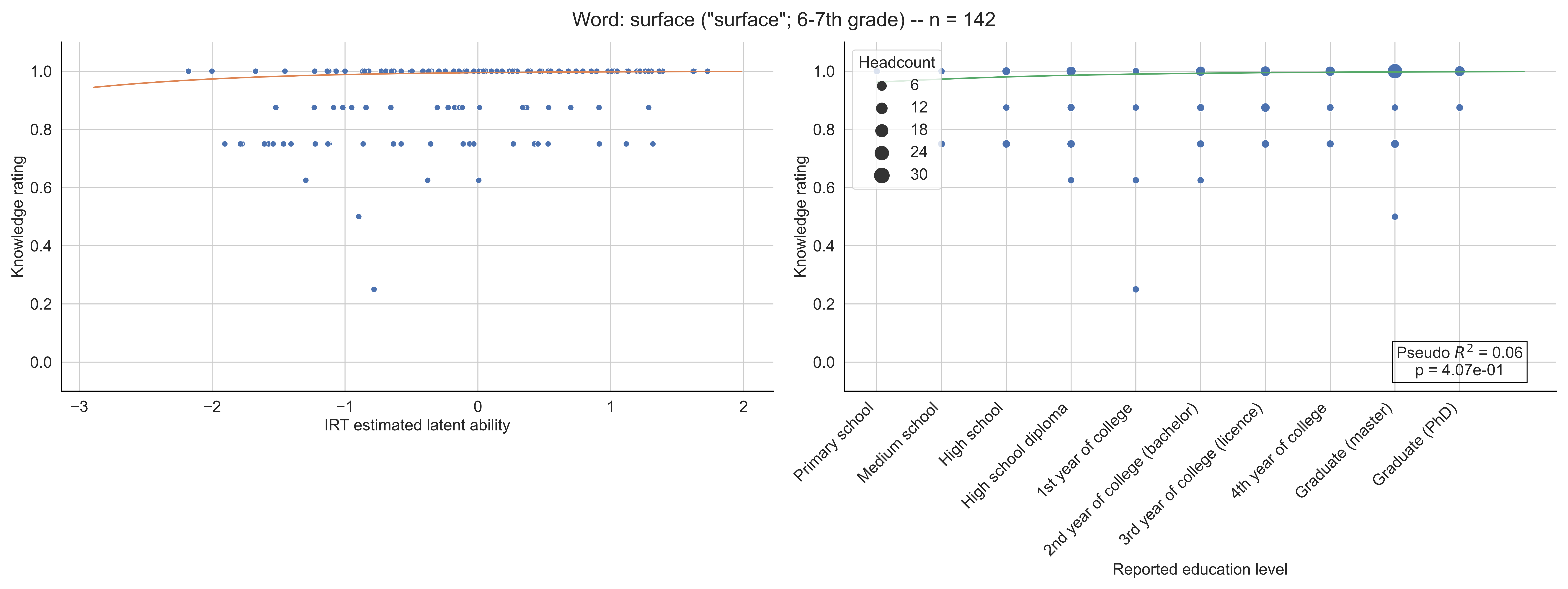

Word: surface ("surface"; 6-7th grade) -- n = 142
Knowledge rating
IRT estimated latent ability
Headcount
6
12
18
24
30
Pseudo R^2 = 0.06
p = 4.07e-01
Primary school
Medium school
High school
High school diploma
1st year of college
2nd year of college (bachelor)
3rd year of college (licence)
4th year of college
Graduate (master)
Graduate (PhD)
Reported education level

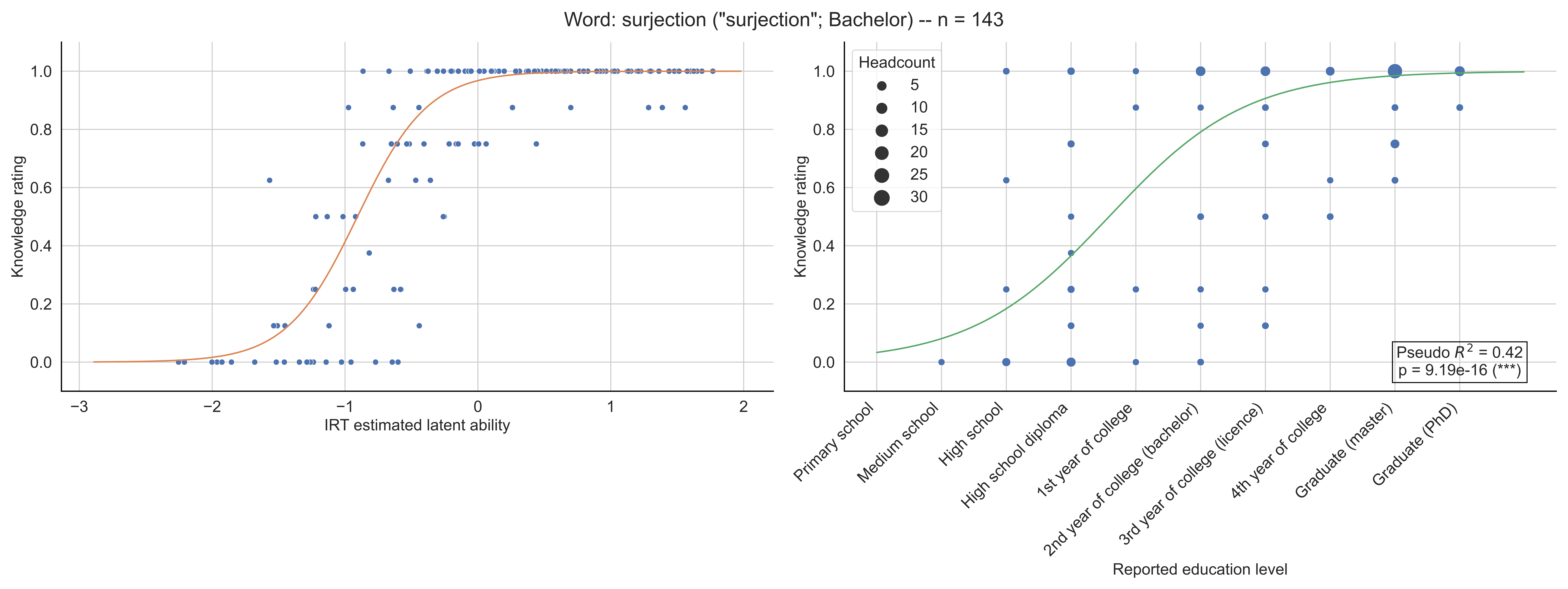
Word: surjection ("surjection"; Bachelor) -- n = 143
Knowledge rating
IRT estimated latent ability
Headcount
5
10
15
20
25
30
Pseudo R^2 = 0.42
p = 9.19e-16 (***)
Primary school
Medium school
High school
High school diploma
1st year of college
2nd year of college (bachelor)
3rd year of college (licence)
4th year of college
Graduate (master)
Graduate (PhD)
Reported education level

Word: sécante ("secant"; Bachelor) -- n = 117

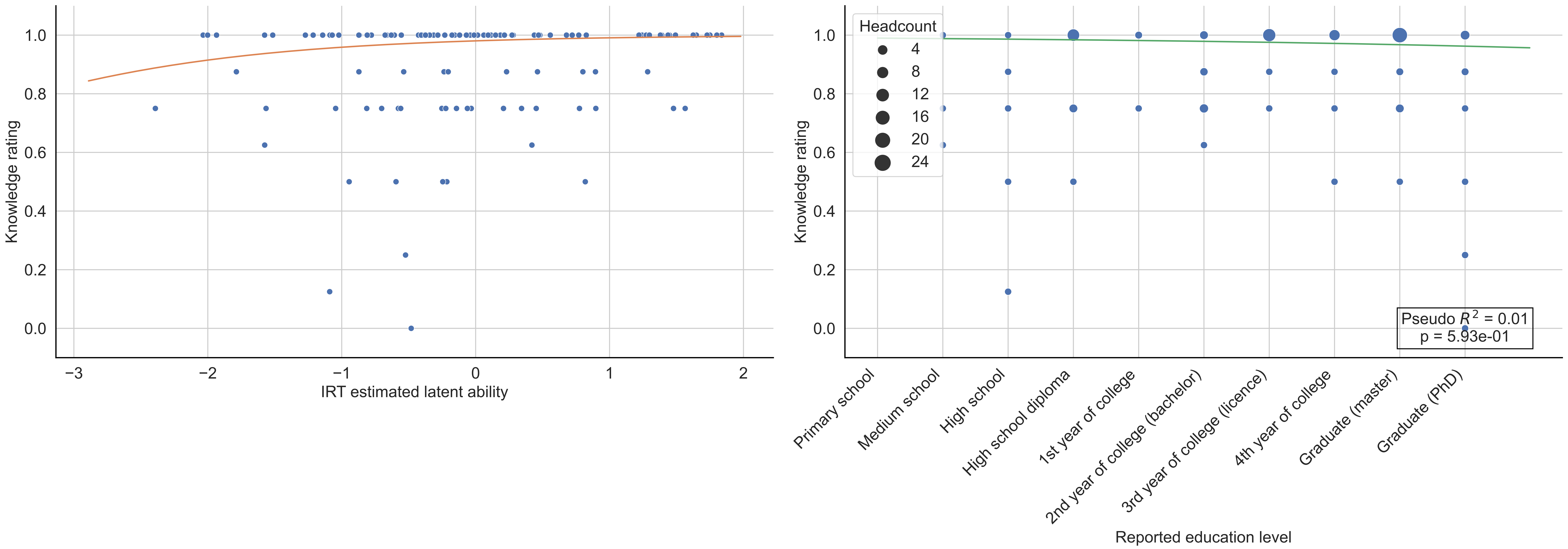

Word: séparation ("separation"; Licence) -- n = 144

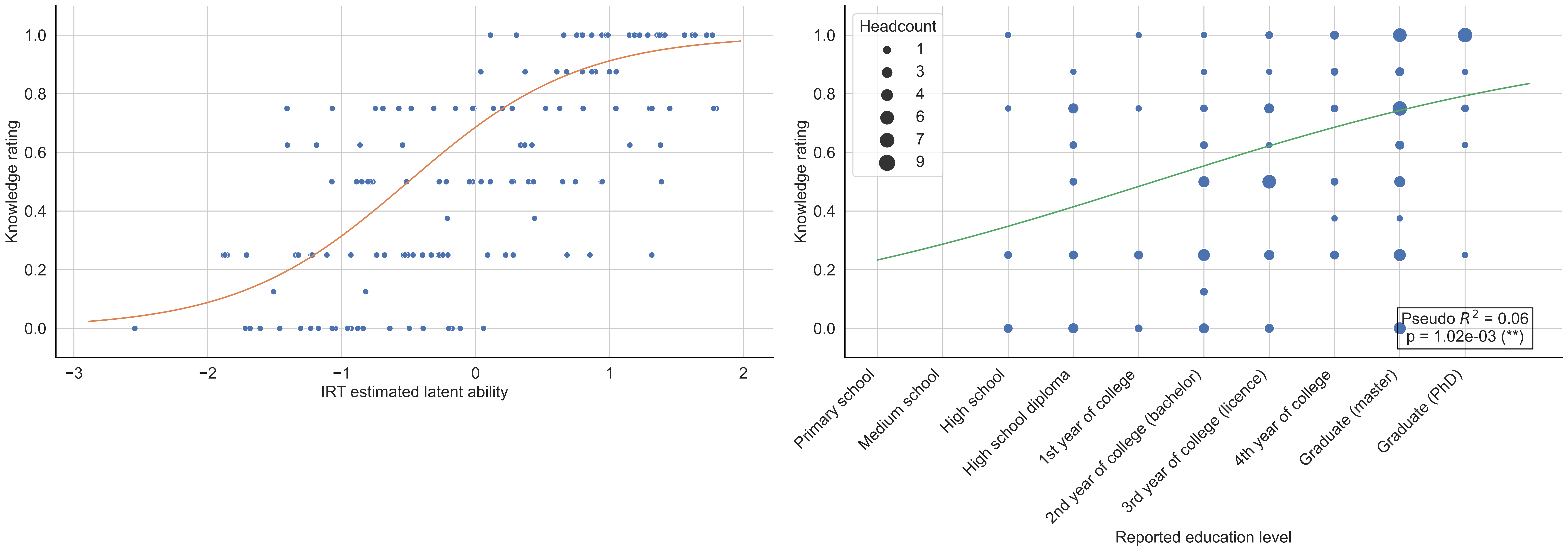

Word: série ("series"; Bachelor) -- n = 122

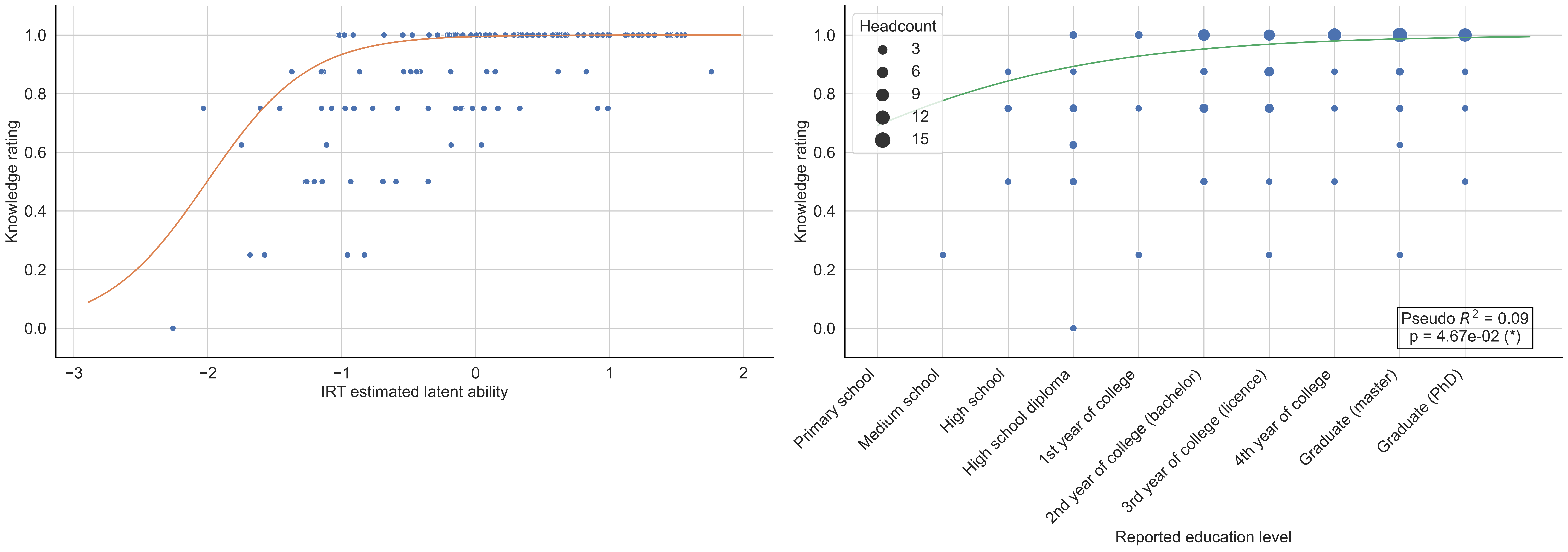

Word: tangence ("tangency??"; Bachelor) -- n = 164

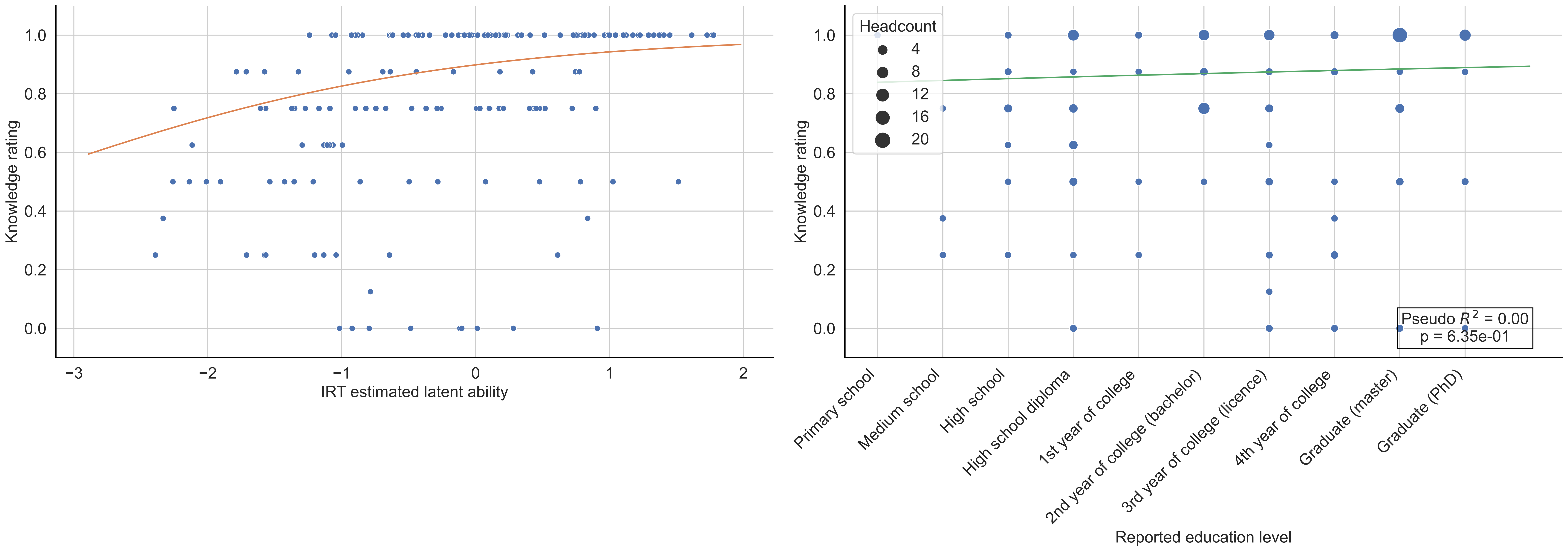

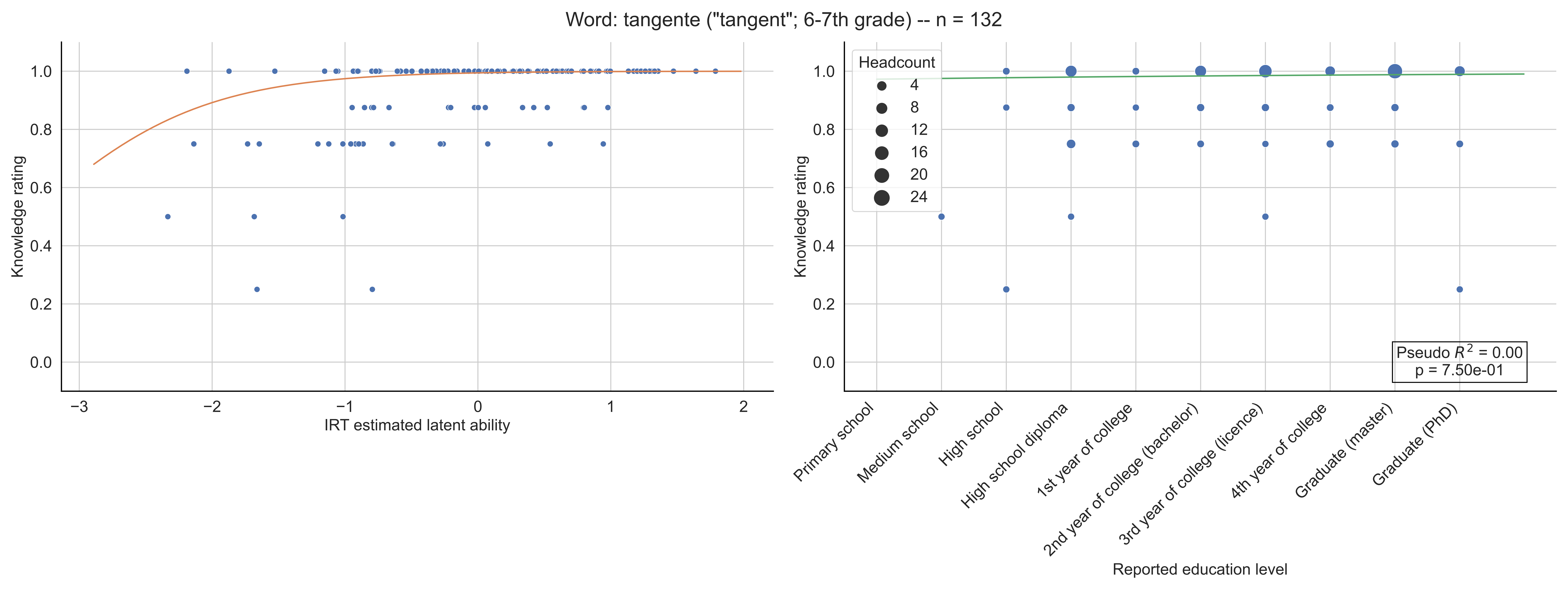
Word: tangente ("tangent"; 6-7th grade) -- n = 132
Knowledge rating
IRT estimated latent ability
Headcount
4
8
12
16
20
24
Primary school
Medium school
High school
High school diploma
1st year of college
2nd year of college (bachelor)
3rd year of college (licence)
4th year of college
Graduate (master)
Graduate (PhD)
Reported education level
Pseudo R^2 = 0.00
p = 7.50e-01

Word: tenseur ("tensor"; Licence) -- n = 134

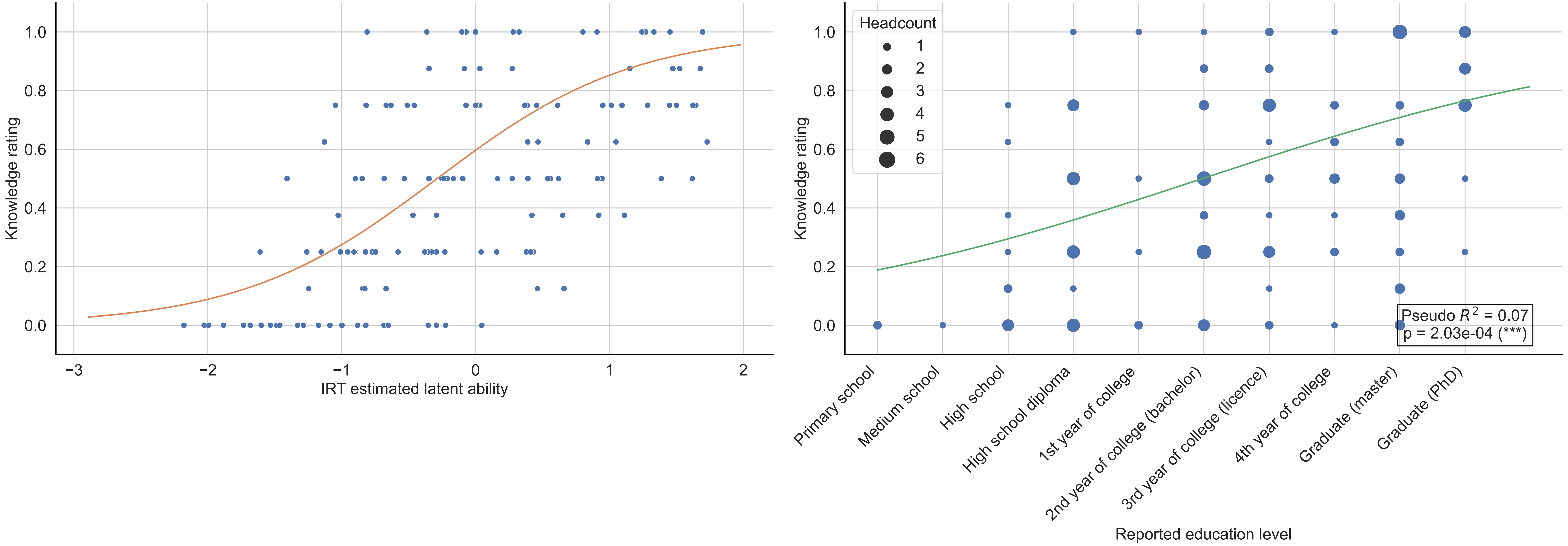

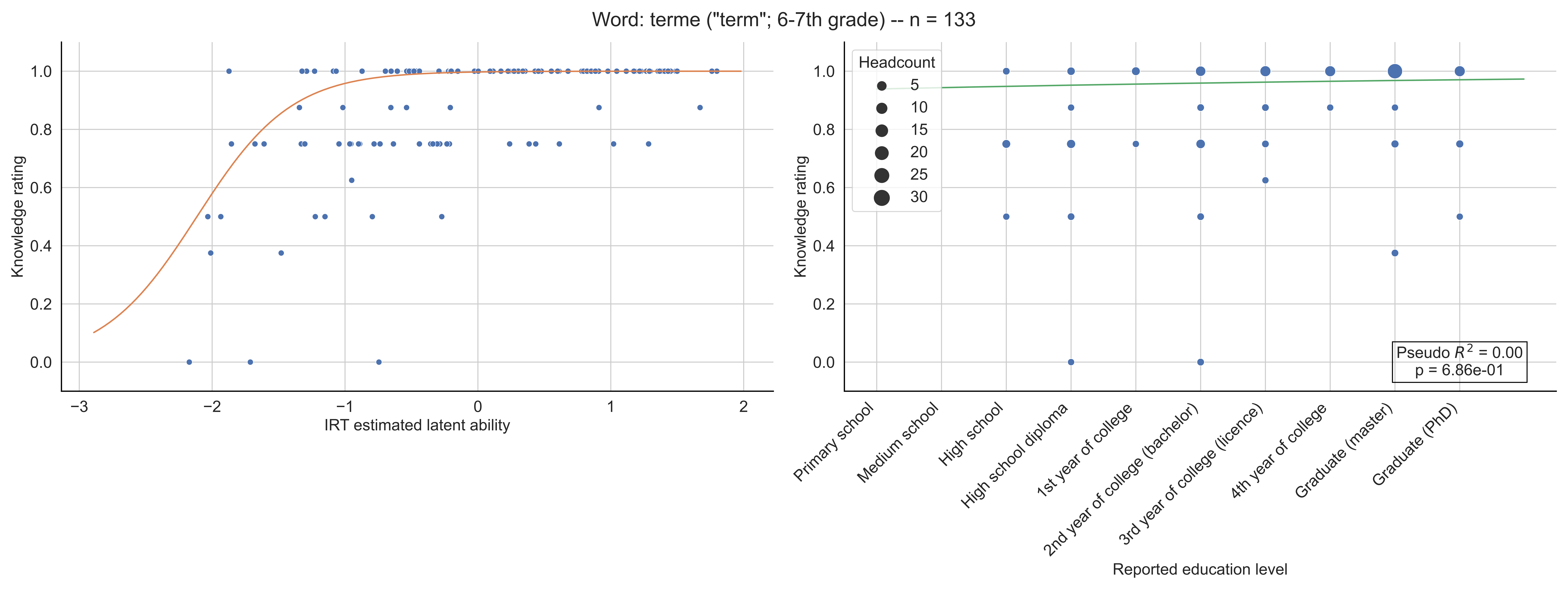

Word: terme ("term"; 6-7th grade) -- n = 133
Knowledge rating
IRT estimated latent ability
Headcount
5
10
15
20
25
30
Pseudo R^2 = 0.00
p = 6.86e-01
Primary school
Medium school
High school
High school diploma
1st year of college
2nd year of college (bachelor)
3rd year of college (licence)
4th year of college
Graduate (master)
Graduate (PhD)
Reported education level

Word: ternaire ("ternary"; 10th grade) -- n = 146

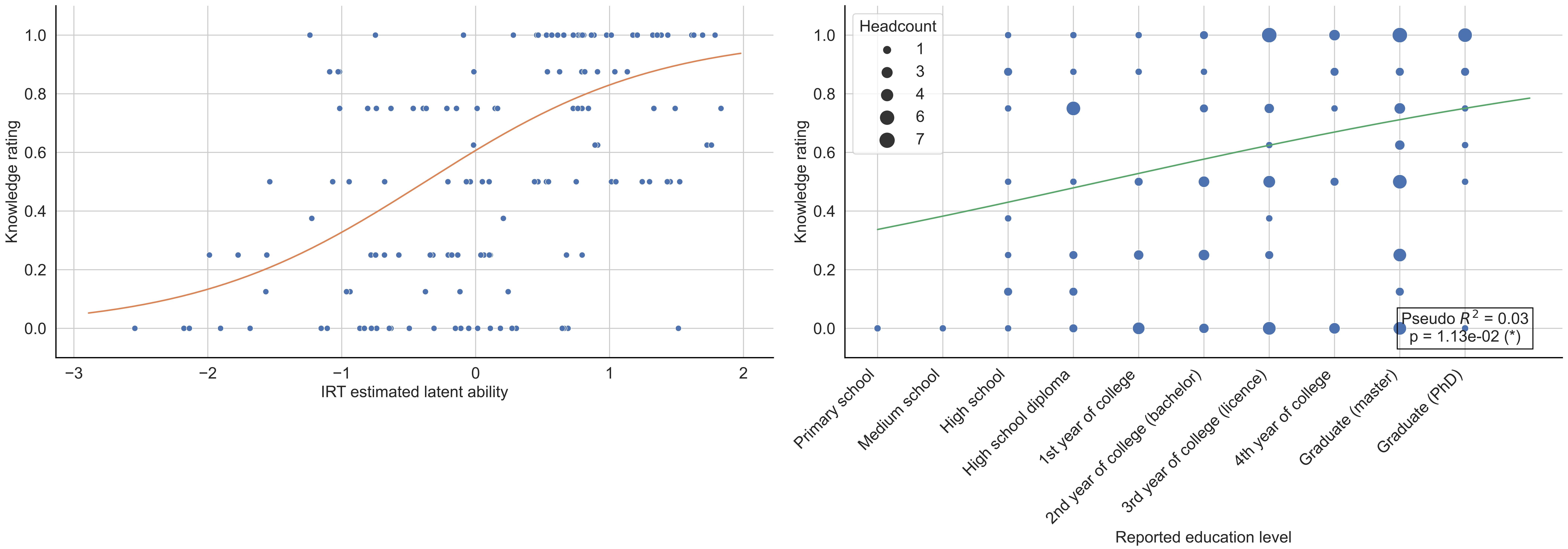

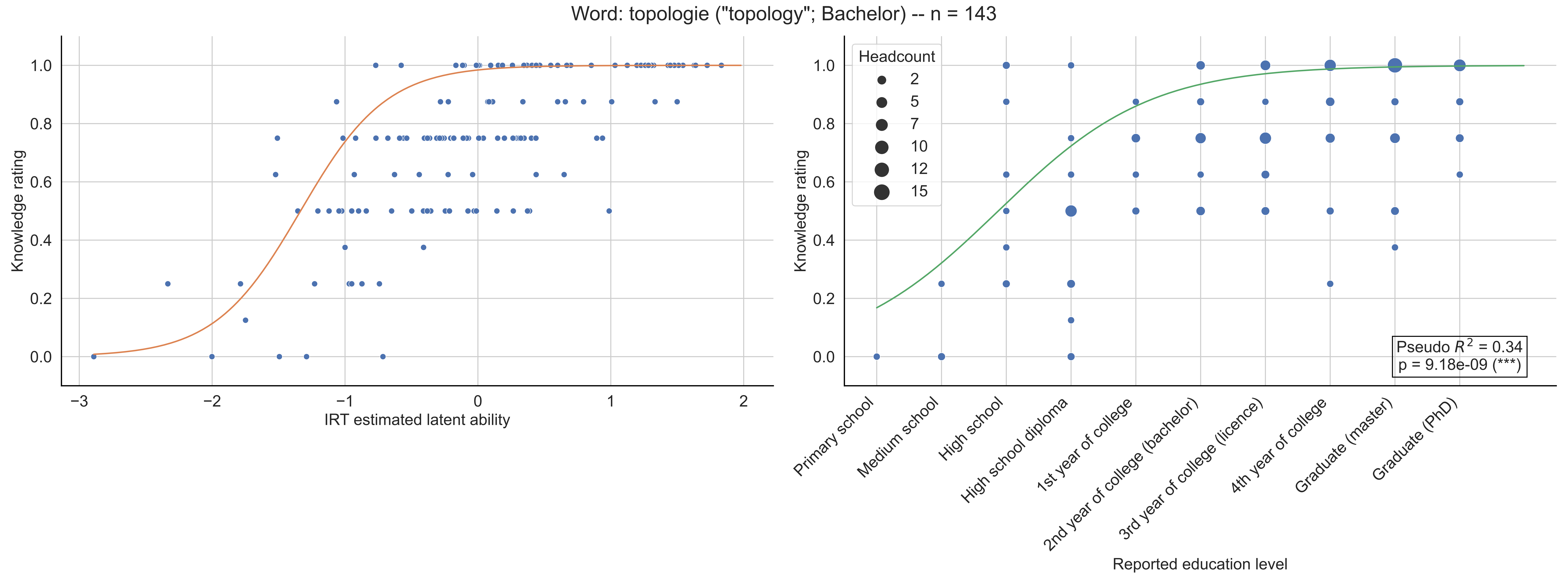

Word: topologie ("topology"; Bachelor) -- n = 143
Knowledge rating
IRT estimated latent ability
−3
−2
−1
0
1
2
0.0
0.2
0.4
0.6
0.8
1.0
Headcount
2
5
7
10
12
15
Pseudo R^2 = 0.34
p = 9.18e-09 (***)
Primary school
Medium school
High school
High school diploma
1st year of college
2nd year of college (bachelor)
3rd year of college (licence)
4th year of college
Graduate (master)
Graduate (PhD)
Reported education level

Word: tore ("torus"; Licence) -- n = 134

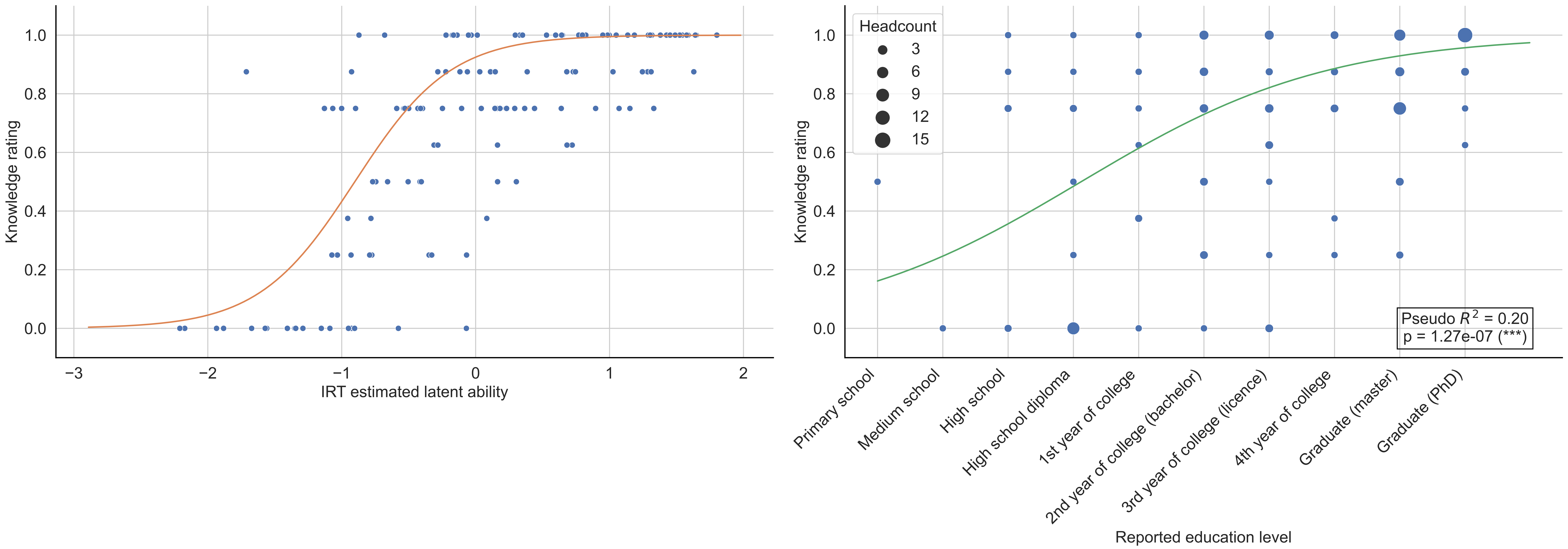

Word: torseur ("torsor"; Licence) -- n = 151

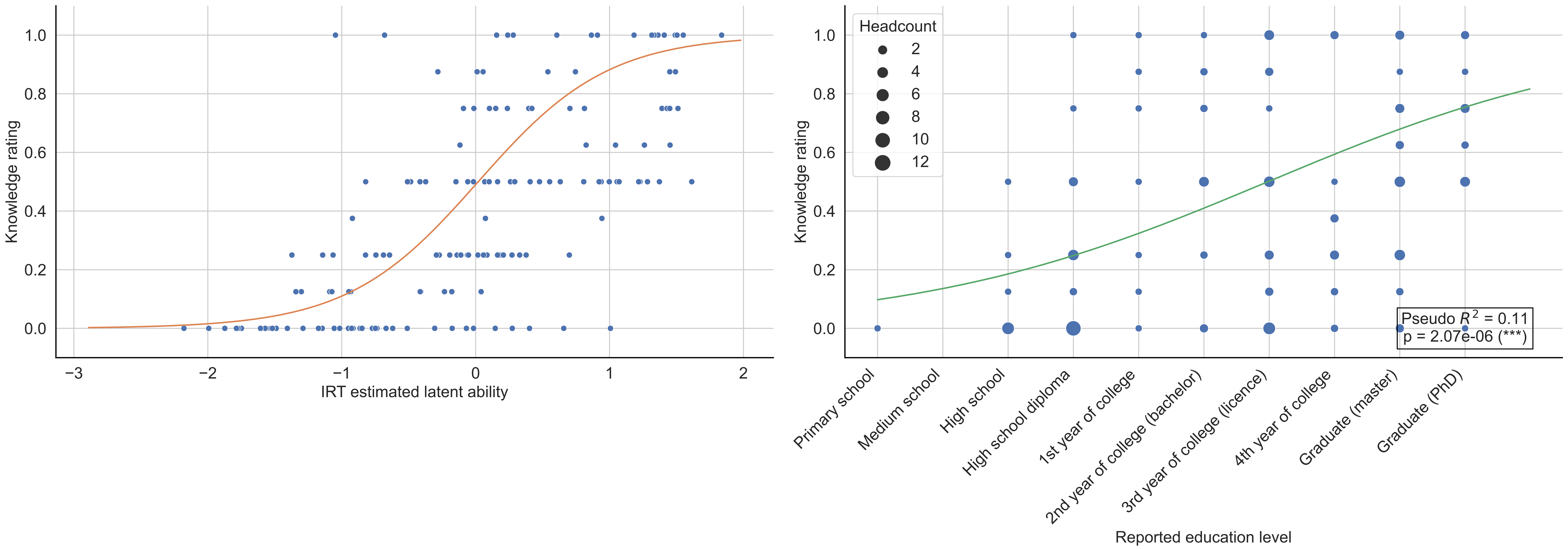

Word: torsion ("torsion"; Licence) -- n = 148

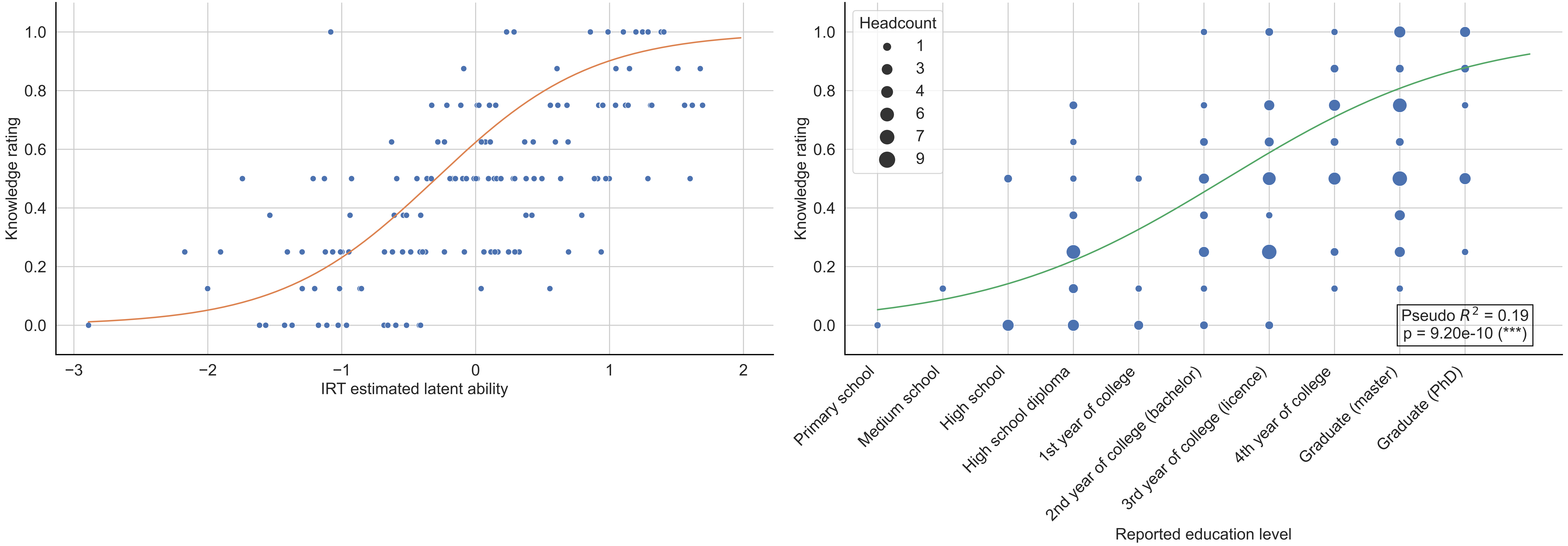

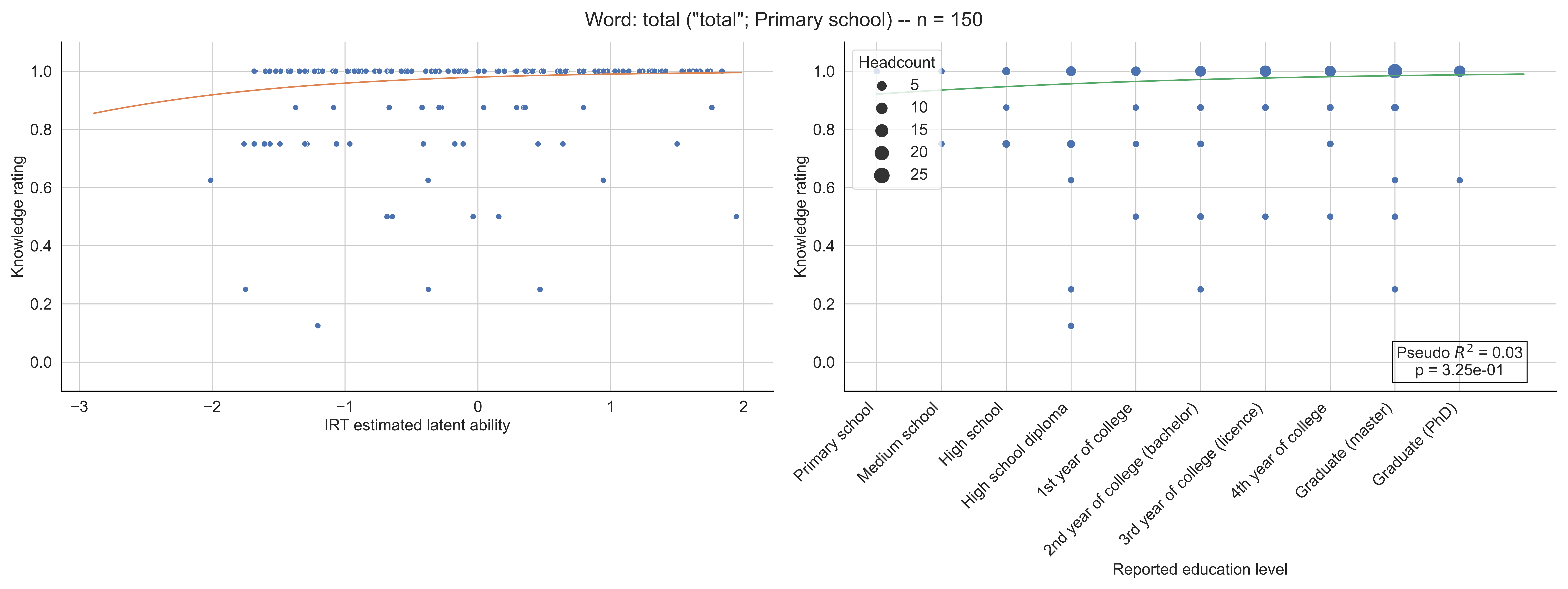

Word: total ("total"; Primary school) -- n = 150
Knowledge rating
IRT estimated latent ability
Headcount
5
10
15
20
25
Pseudo R^2 = 0.03
p = 3.25e-01
Primary school
Medium school
High school
High school diploma
1st year of college
2nd year of college (bachelor)
3rd year of college (licence)
4th year of college
Graduate (master)
Graduate (PhD)
Reported education level

Word: trace ("trace"; Bachelor) -- n = 142

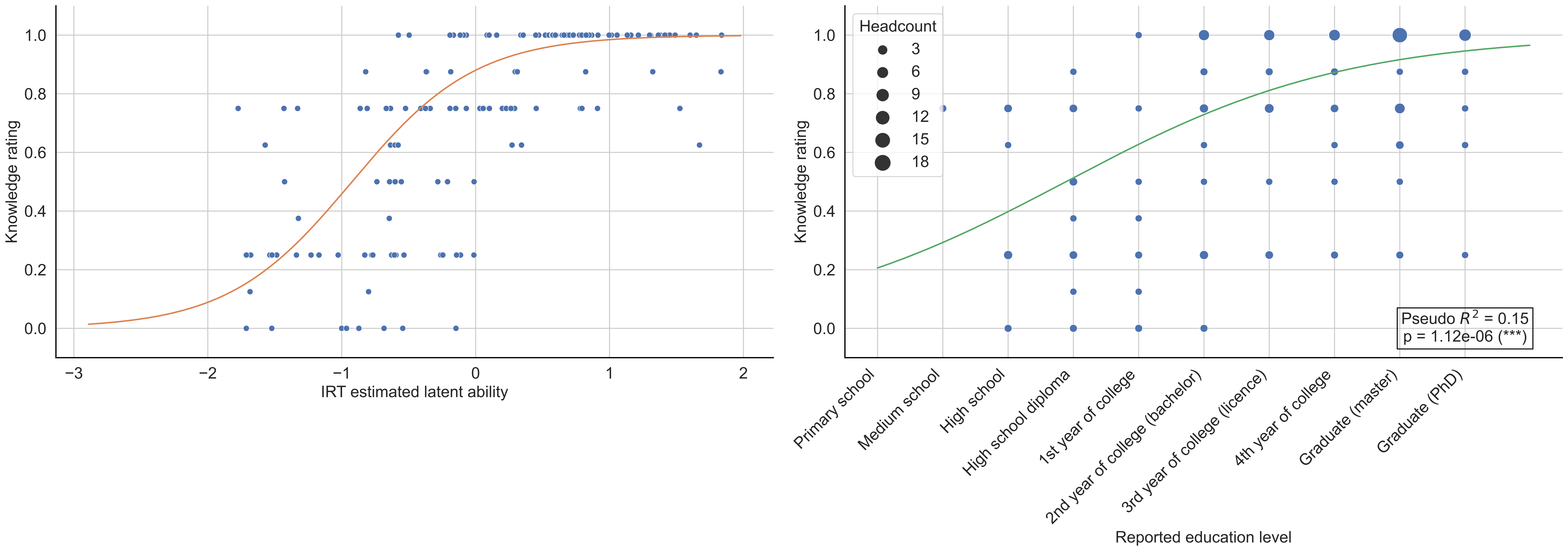

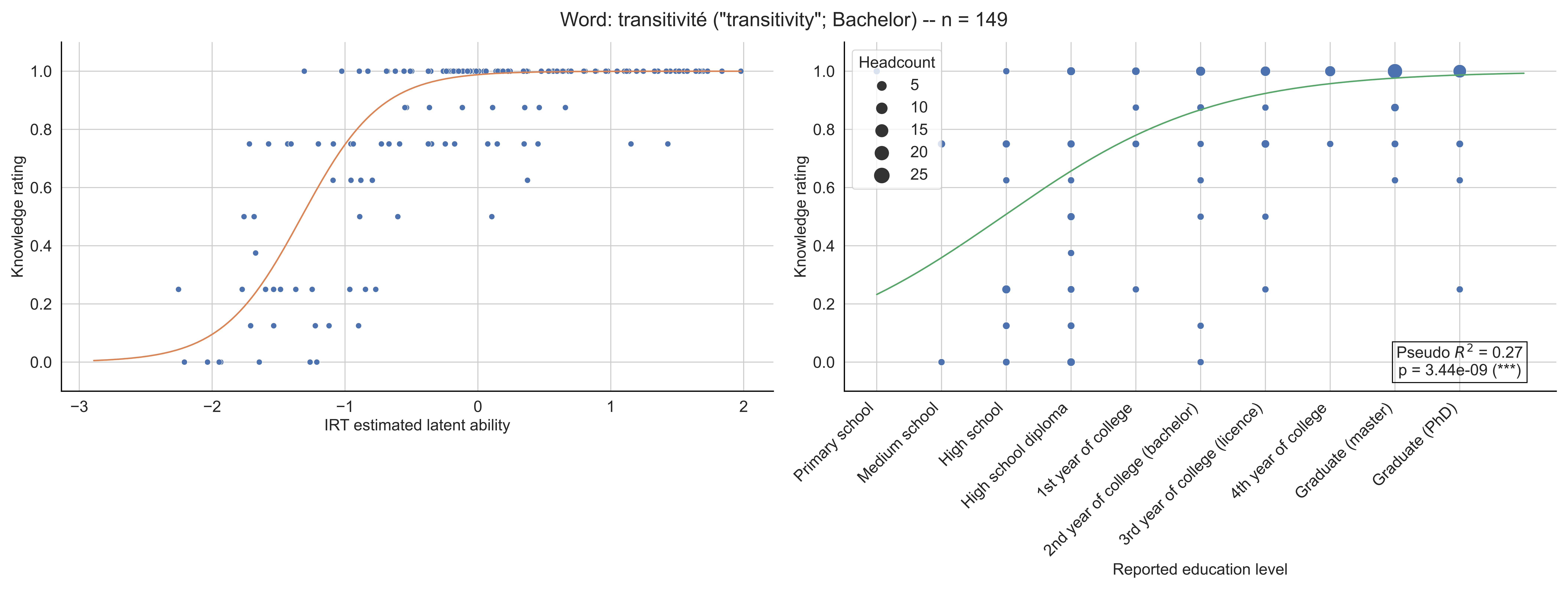

Word: transitivité ("transitivity"; Bachelor) -- n = 149
Knowledge rating
IRT estimated latent ability
Headcount
5
10
15
20
25
Pseudo R^2 = 0.27
p = 3.44e-09 (***)
Primary school
Medium school
High school
High school diploma
1st year of college
2nd year of college (bachelor)
3rd year of college (licence)
4th year of college
Graduate (master)
Graduate (PhD)
Reported education level

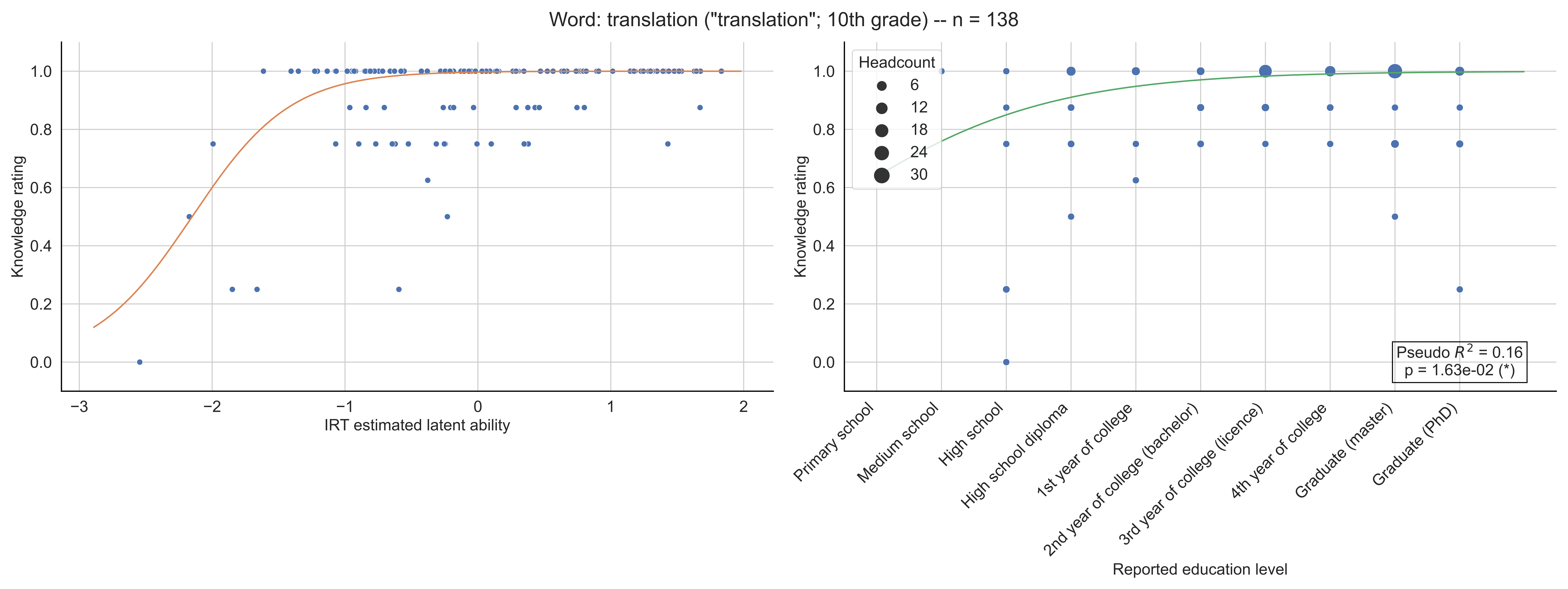
Word: translation ("translation"; 10th grade) -- n = 138
Knowledge rating
IRT estimated latent ability
Headcount
6
12
18
24
30
Pseudo R^2 = 0.16
p = 1.63e-02 (*)
Primary school
Medium school
High school
High school diploma
1st year of college
2nd year of college (bachelor)
3rd year of college (licence)
4th year of college
Graduate (master)
Graduate (PhD)
Reported education level

Word: transposition ("transposition"; Bachelor) -- n = 154

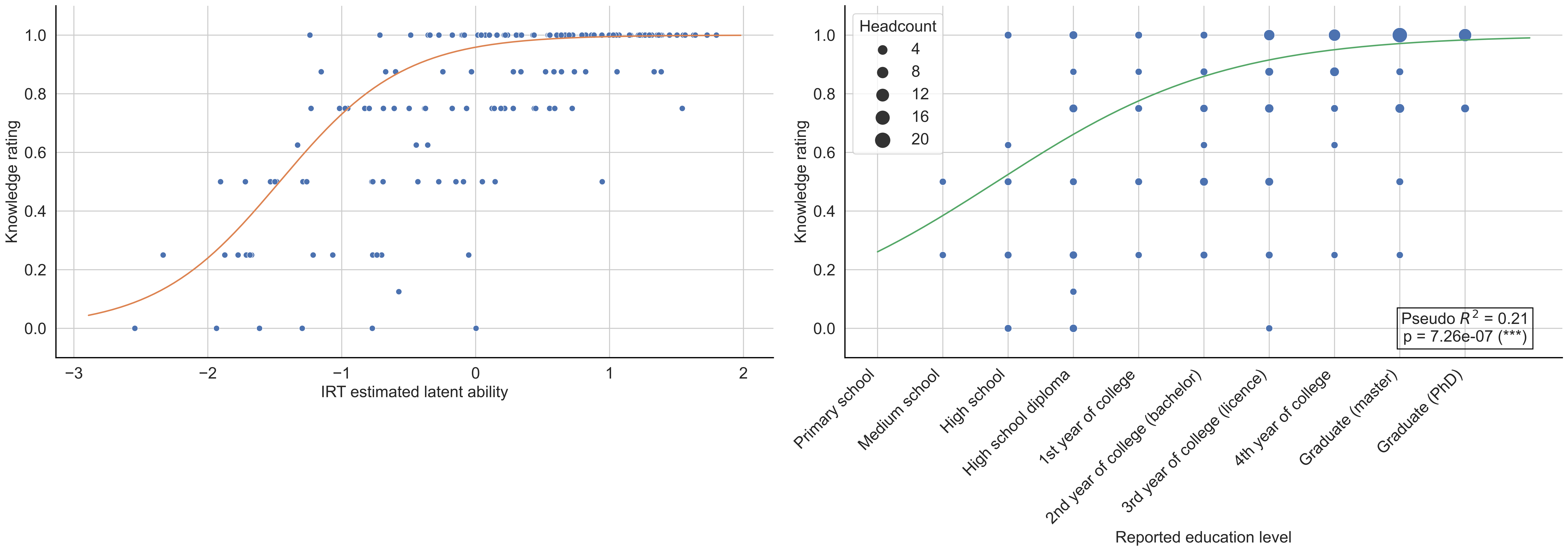

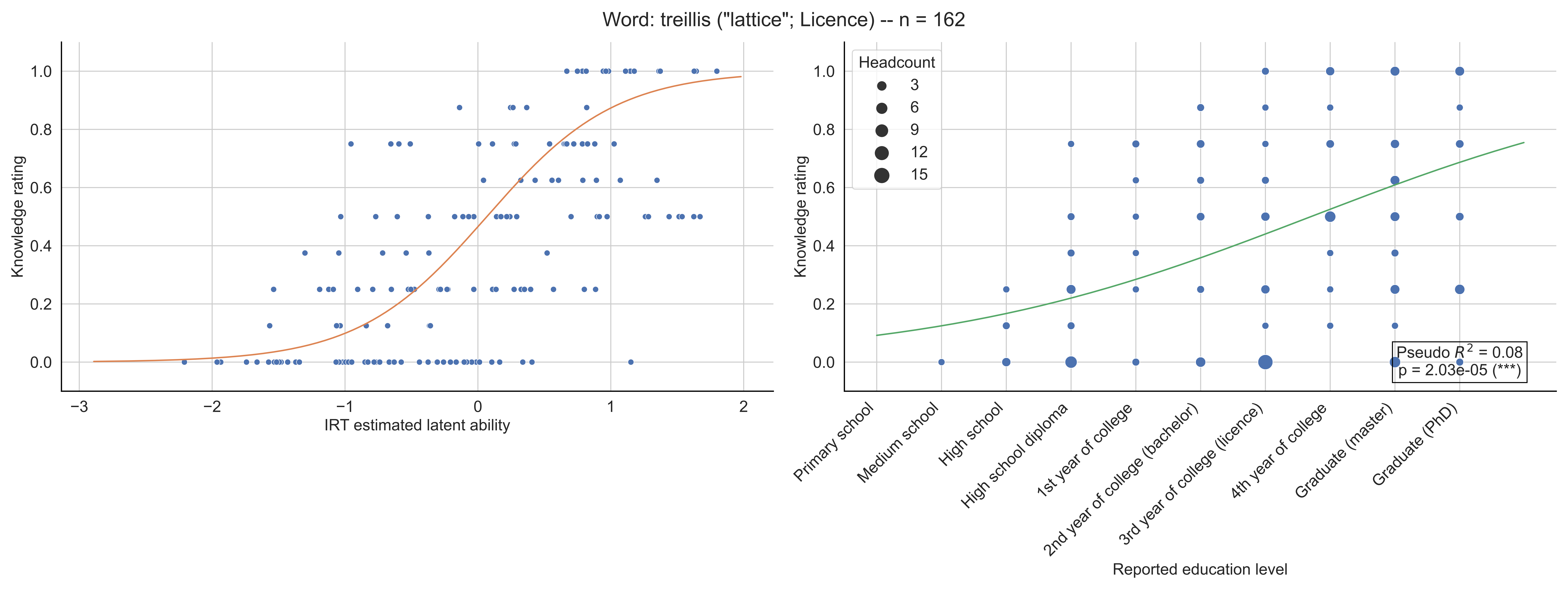

Word: treillis ("lattice"; Licence) -- n = 162
Knowledge rating
IRT estimated latent ability
Headcount
3
6
9
12
15
Pseudo R^2 = 0.08
p = 2.03e-05 (***)
Primary school
Medium school
High school
High school diploma
1st year of college
2nd year of college (bachelor)
3rd year of college (licence)
4th year of college
Graduate (master)
Graduate (PhD)
Reported education level

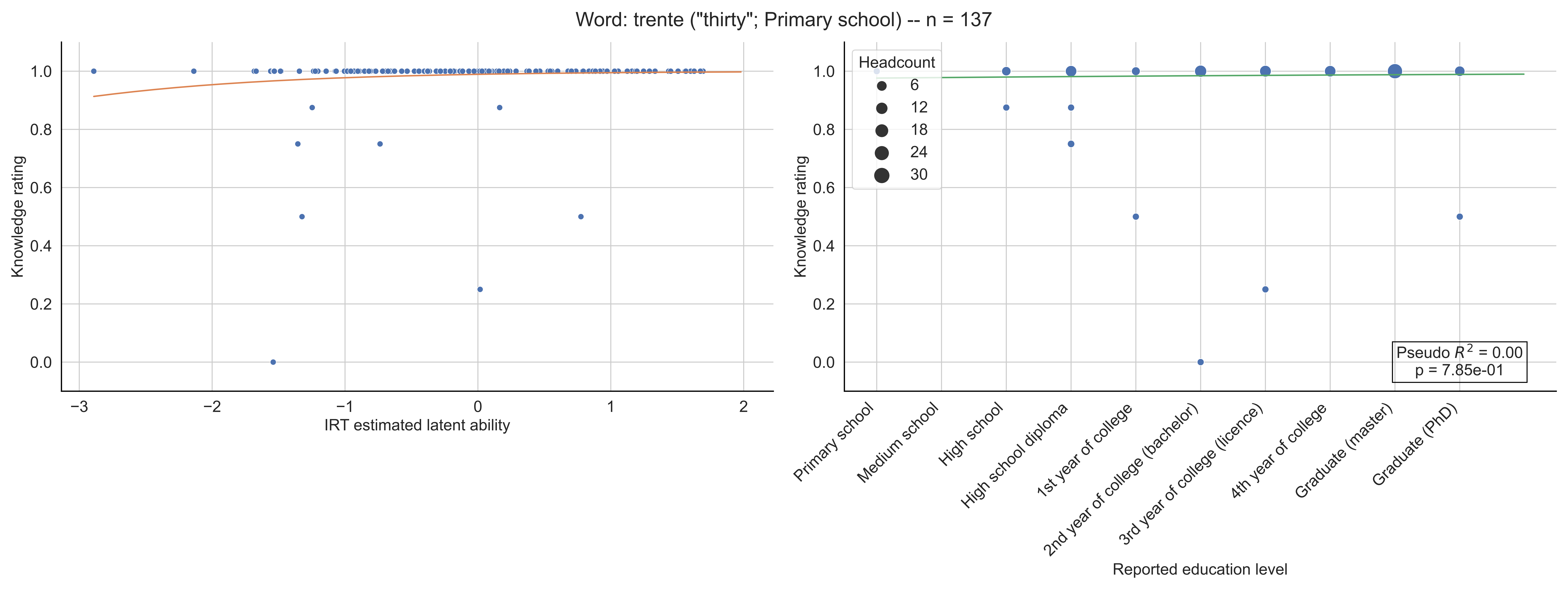
Word: trente ("thirty"; Primary school) -- n = 137
Knowledge rating
IRT estimated latent ability
Headcount
6
12
18
24
30
Pseudo R^2 = 0.00
p = 7.85e-01
Primary school
Medium school
High school
High school diploma
1st year of college
2nd year of college (bachelor)
3rd year of college (licence)
4th year of college
Graduate (master)
Graduate (PhD)
Reported education level

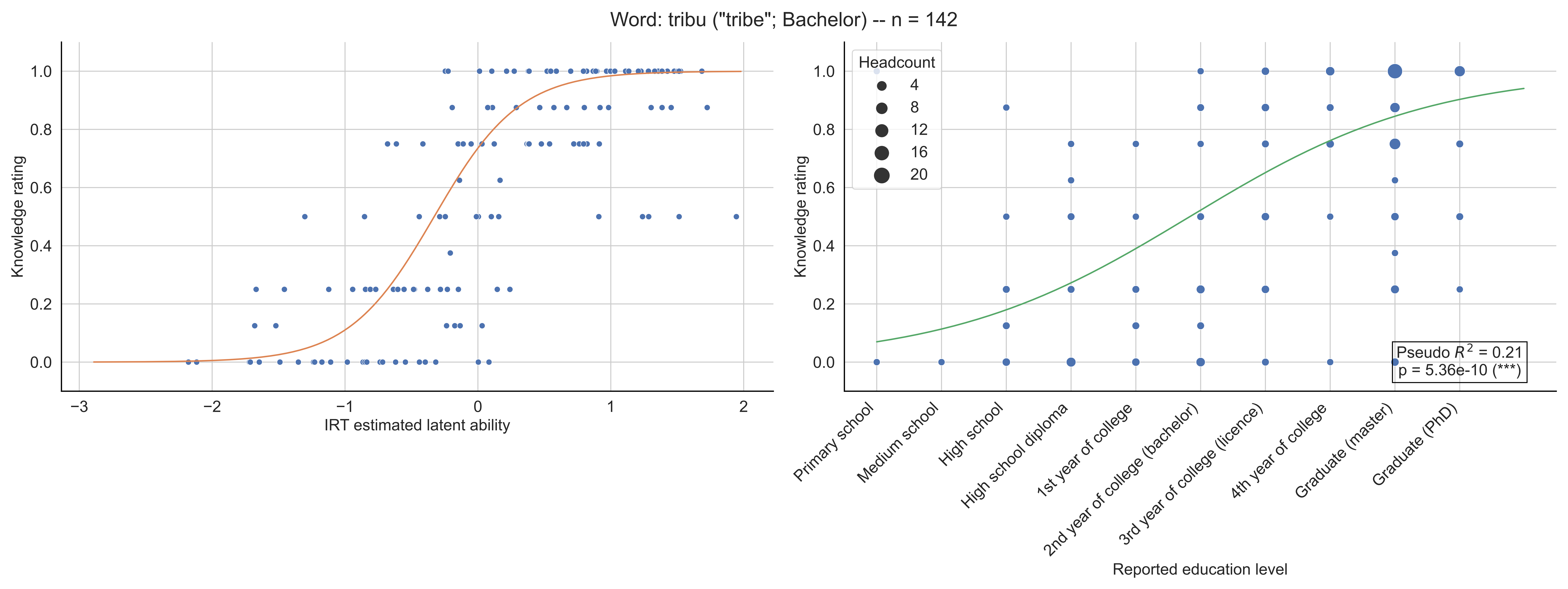
Word: tribu ("tribe"; Bachelor) -- n = 142
Knowledge rating
IRT estimated latent ability
Headcount
4
8
12
16
20
Pseudo R^2 = 0.21
p = 5.36e-10 (***)
Primary school
Medium school
High school
High school diploma
1st year of college
2nd year of college (bachelor)
3rd year of college (licence)
4th year of college
Graduate (master)
Graduate (PhD)
Reported education level

Word: trigonométrie ("trigonometry"; 8-9th grade) -- n = 135

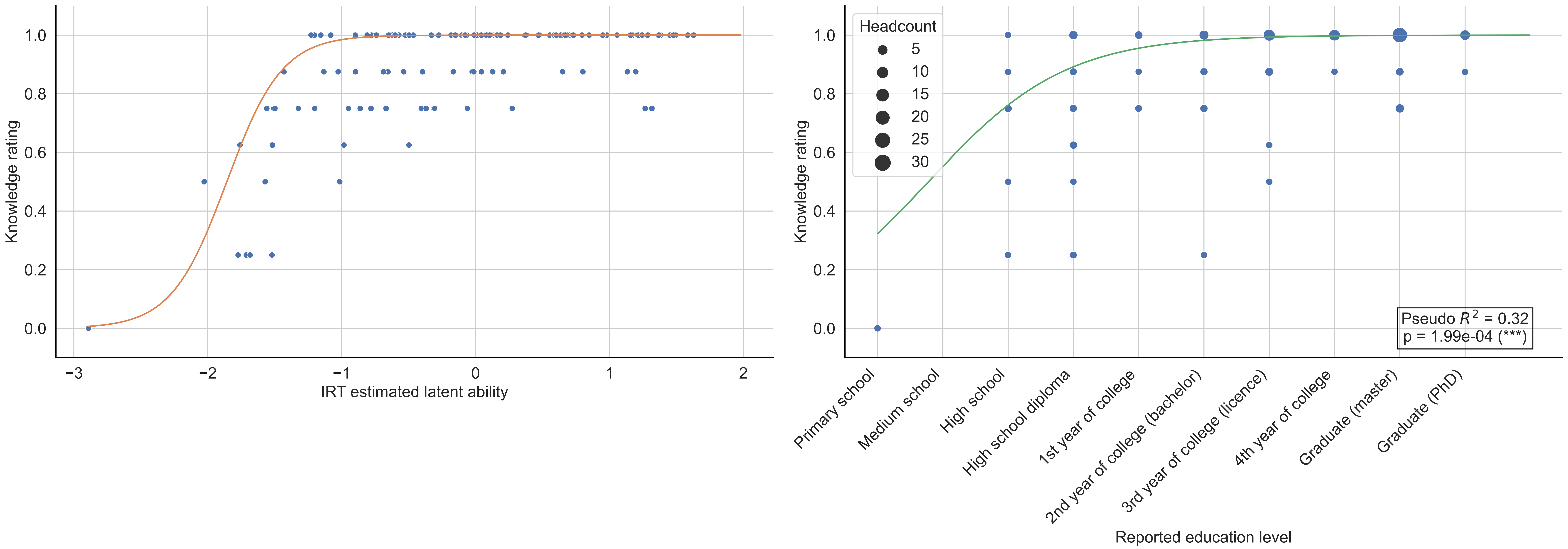

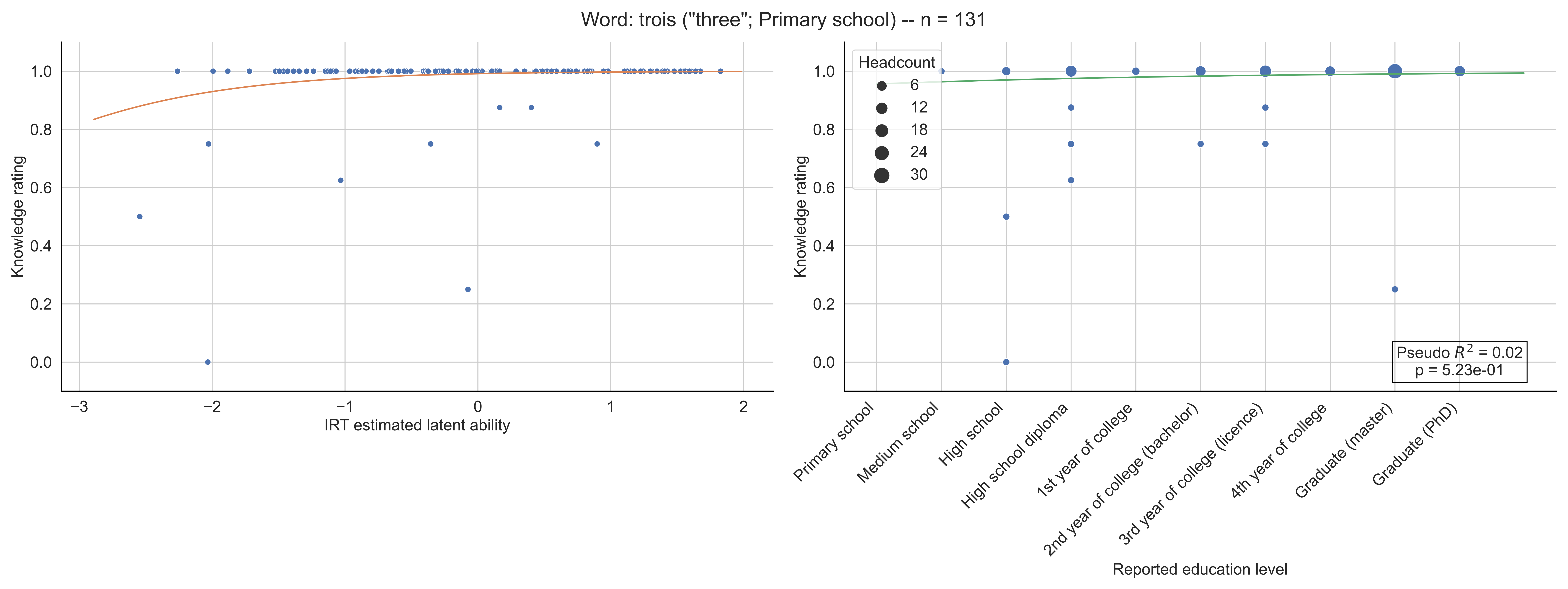

Word: trois ("three"; Primary school) -- n = 131
Knowledge rating
1.0
0.8
0.6
0.4
0.2
0.0
−3
−2
−1
0
1
2
IRT estimated latent ability
Headcount
6
12
18
24
30
Pseudo R^2 = 0.02
p = 5.23e-01
Primary school
Medium school
High school
High school diploma
1st year of college
2nd year of college (bachelor)
3rd year of college (licence)
4th year of college
Graduate (master)
Graduate (PhD)
Reported education level

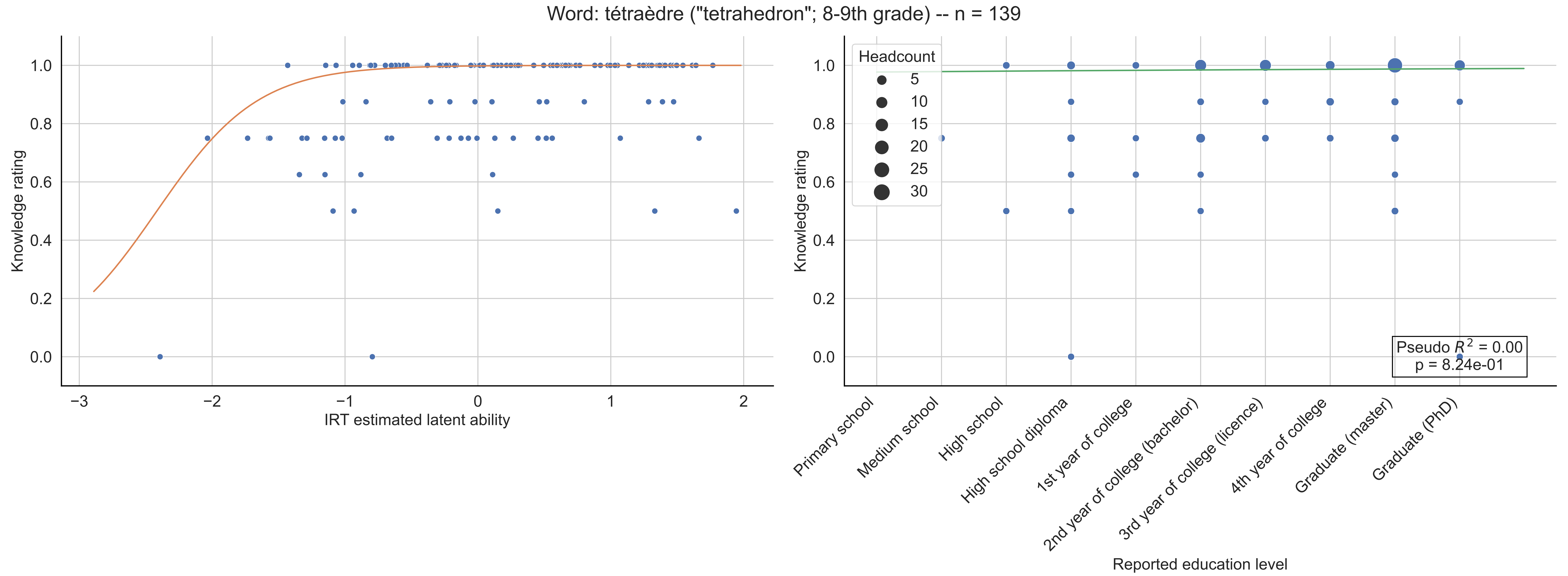
Word: tétraèdre ("tetrahedron"; 8-9th grade) -- n = 139
Knowledge rating
IRT estimated latent ability
Headcount
5
10
15
20
25
30
Pseudo R^2 = 0.00
p = 8.24e-01
Primary school
Medium school
High school
High school diploma
1st year of college
2nd year of college (bachelor)
3rd year of college (licence)
4th year of college
Graduate (master)
Graduate (PhD)
Reported education level

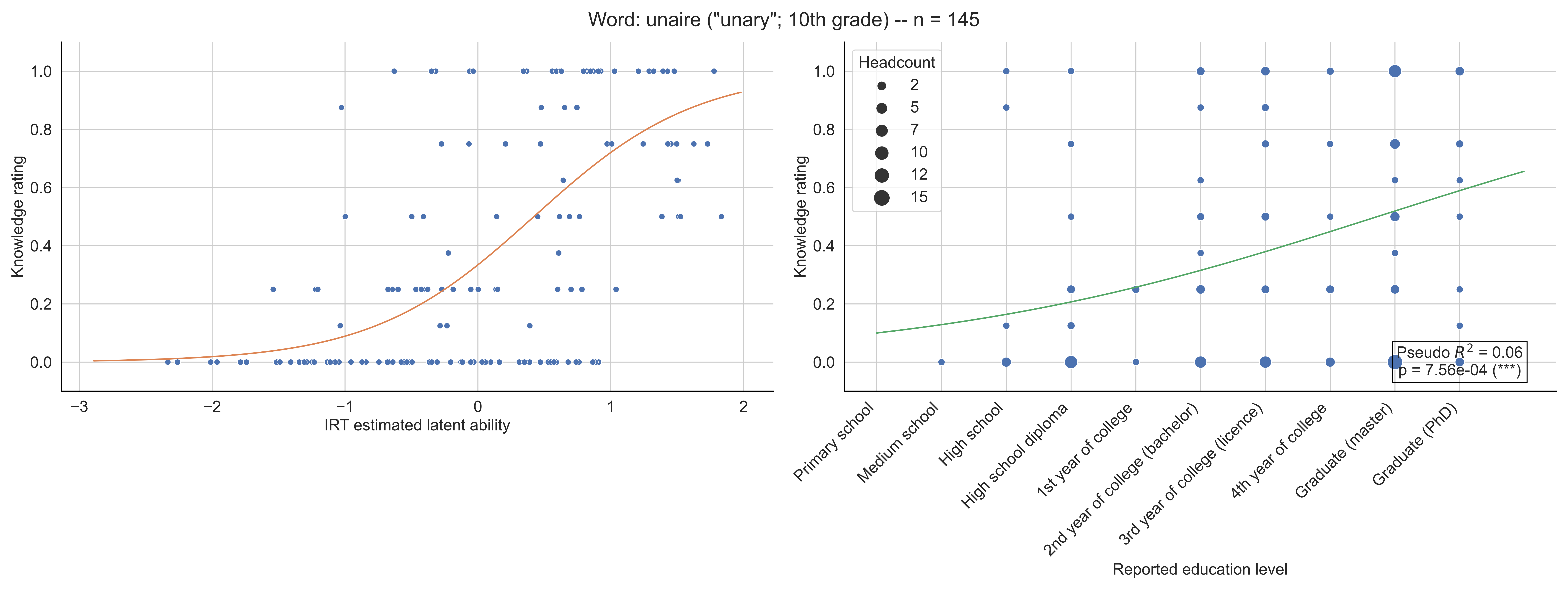

Word: unaire ("unary"; 10th grade) -- n = 145
Knowledge rating
IRT estimated latent ability
Headcount
2
5
7
10
12
15
Pseudo R^2 = 0.06
p = 7.56e-04 (***)
Primary school
Medium school
High school
High school diploma
1st year of college
2nd year of college (bachelor)
3rd year of college (licence)
4th year of college
Graduate (master)
Graduate (PhD)
Reported education level

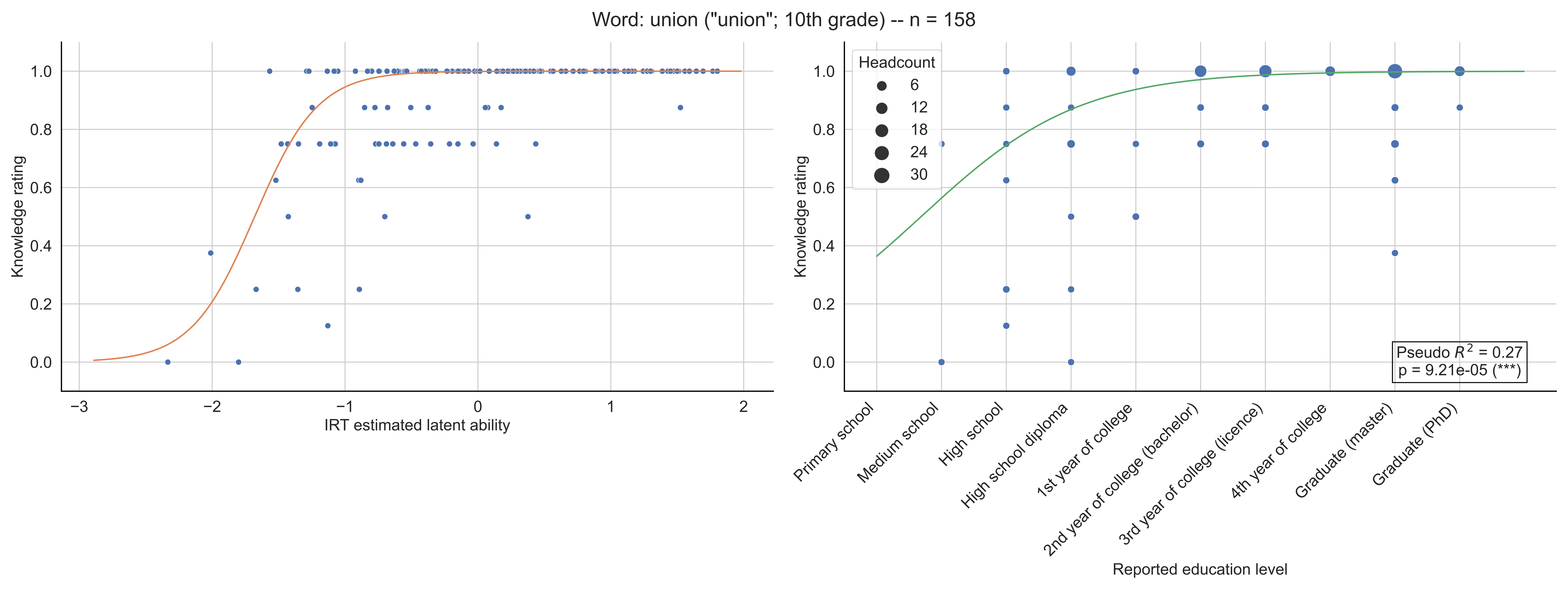

Word: union ("union"; 10th grade) -- n = 158
Knowledge rating
IRT estimated latent ability
Headcount
6
12
18
24
30
Pseudo R^2 = 0.27
p = 9.21e-05 (***)
Primary school
Medium school
High school
High school diploma
1st year of college
2nd year of college (bachelor)
3rd year of college (licence)
4th year of college
Graduate (master)
Graduate (PhD)
Reported education level

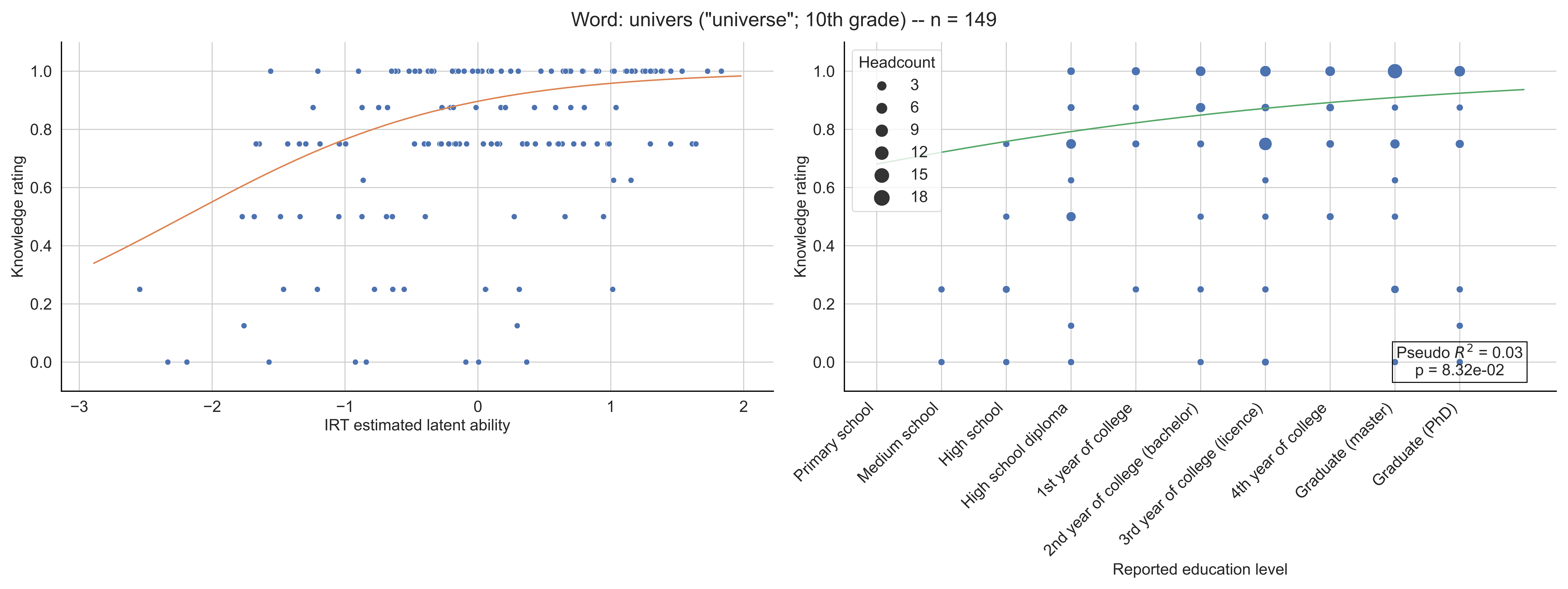

Word: univers ("universe"; 10th grade) -- n = 149
Knowledge rating
IRT estimated latent ability
Headcount
3
6
9
12
15
18
Pseudo R^2 = 0.03
p = 8.32e-02
Primary school
Medium school
High school
High school diploma
1st year of college
2nd year of college (bachelor)
3rd year of college (licence)
4th year of college
Graduate (master)
Graduate (PhD)
Reported education level

Word: valuation ("valuation"; Bachelor) -- n = 131

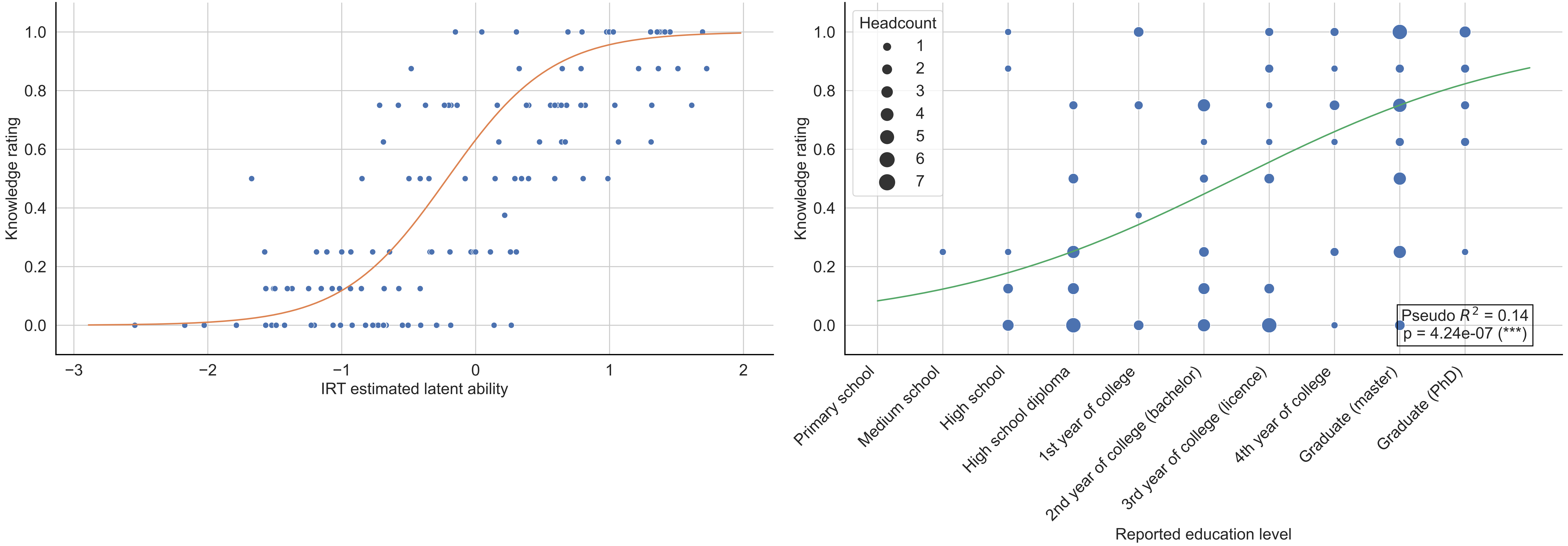

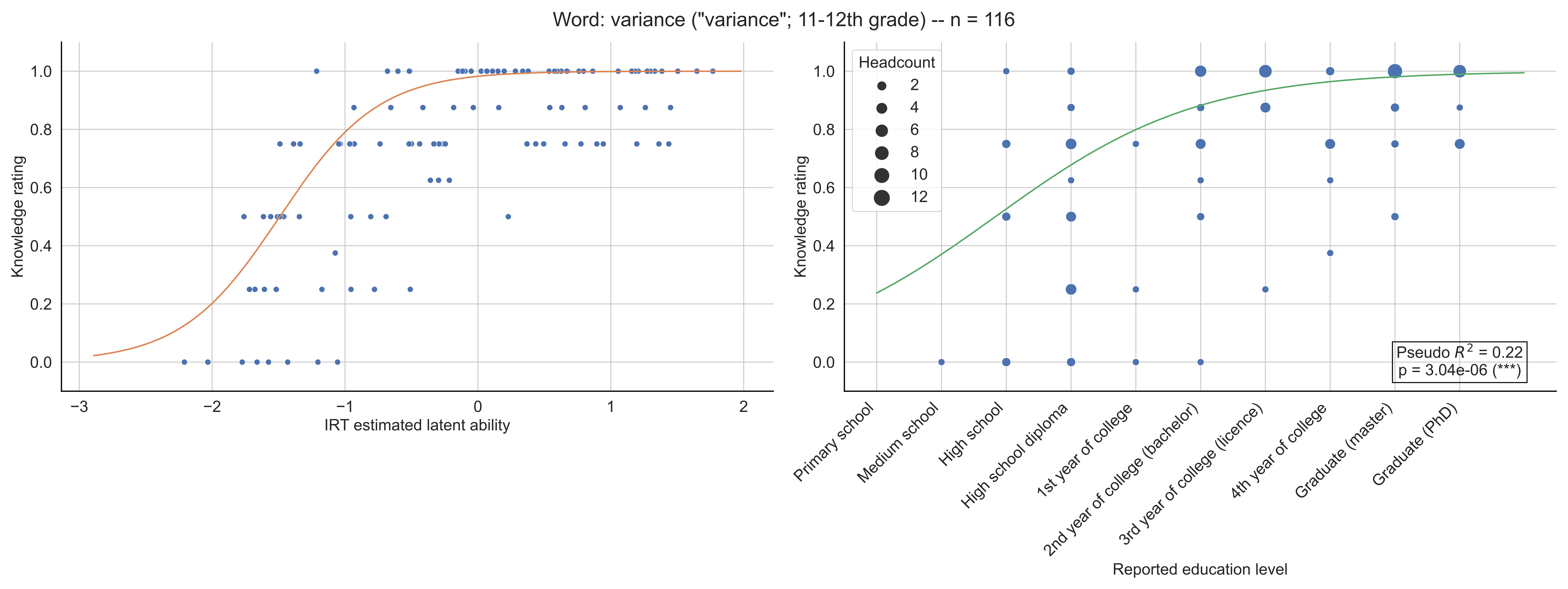

Word: variance ("variance"; 11-12th grade) -- n = 116
Knowledge rating
IRT estimated latent ability
Headcount
2
4
6
8
10
12
Primary school
Medium school
High school
High school diploma
1st year of college
2nd year of college (bachelor)
3rd year of college (licence)
4th year of college
Graduate (master)
Graduate (PhD)
Reported education level
Pseudo R^2 = 0.22
p = 3.04e-06 (***)

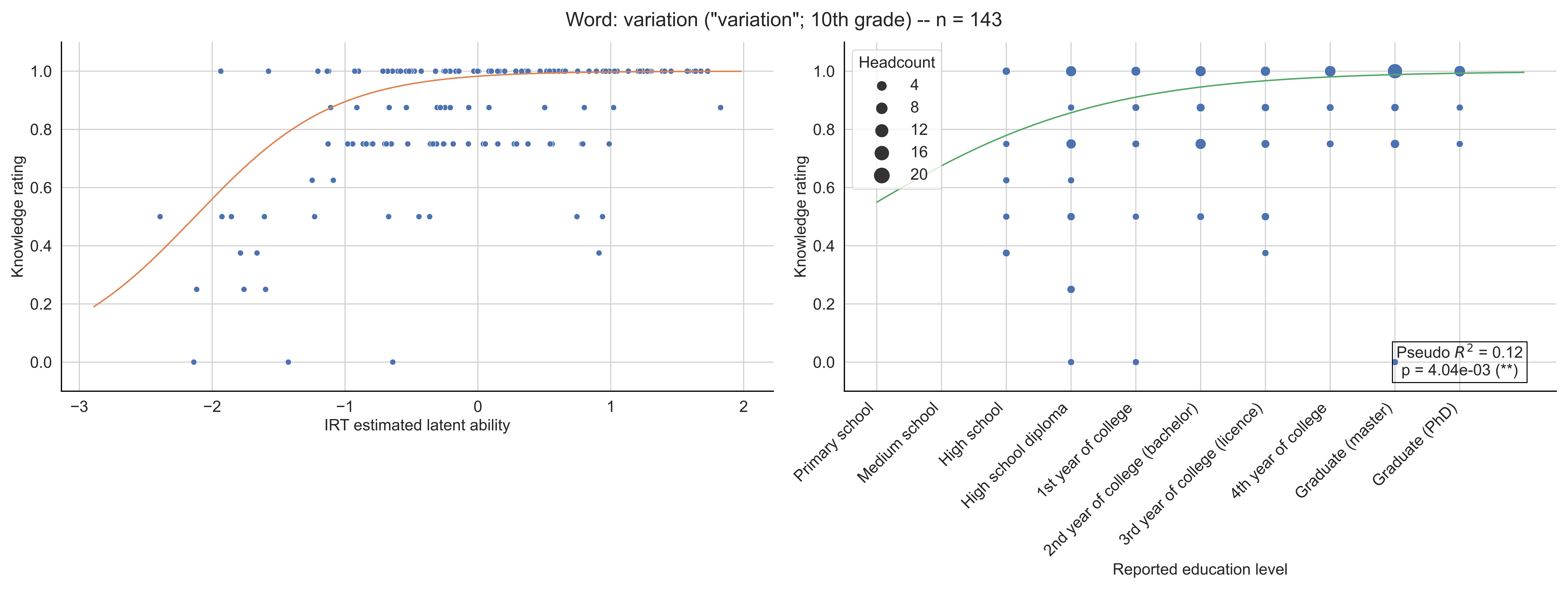

Word: variation ("variation"; 10th grade) -- n = 143
Knowledge rating
IRT estimated latent ability
Headcount
4
8
12
16
20
Pseudo R^2 = 0.12
p = 4.04e-03 (**)
Primary school
Medium school
High school
High school diploma
1st year of college
2nd year of college (bachelor)
3rd year of college (licence)
4th year of college
Graduate (master)
Graduate (PhD)
Reported education level

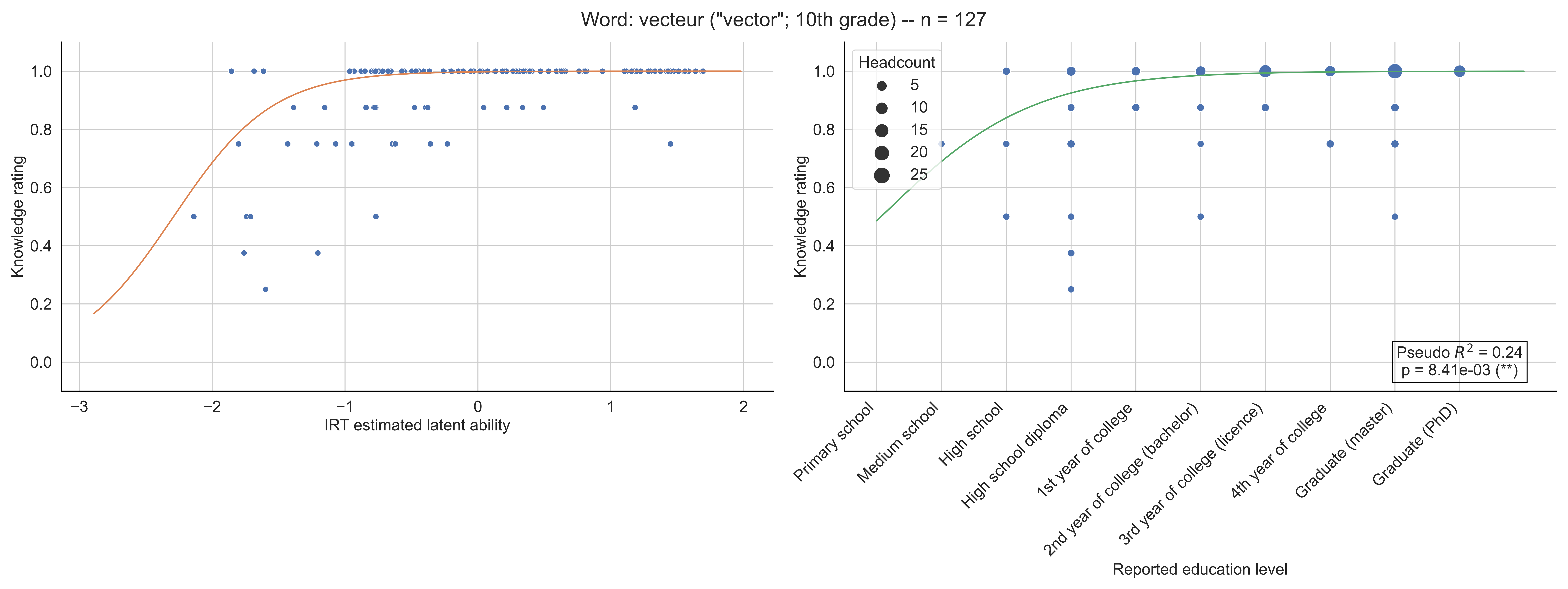

Word: vecteur ("vector"; 10th grade) -- n = 127
Knowledge rating
IRT estimated latent ability
Headcount
5
10
15
20
25
Pseudo R^2 = 0.24
p = 8.41e-03 (**)
Primary school
Medium school
High school
High school diploma
1st year of college
2nd year of college (bachelor)
3rd year of college (licence)
4th year of college
Graduate (master)
Graduate (PhD)
Reported education level

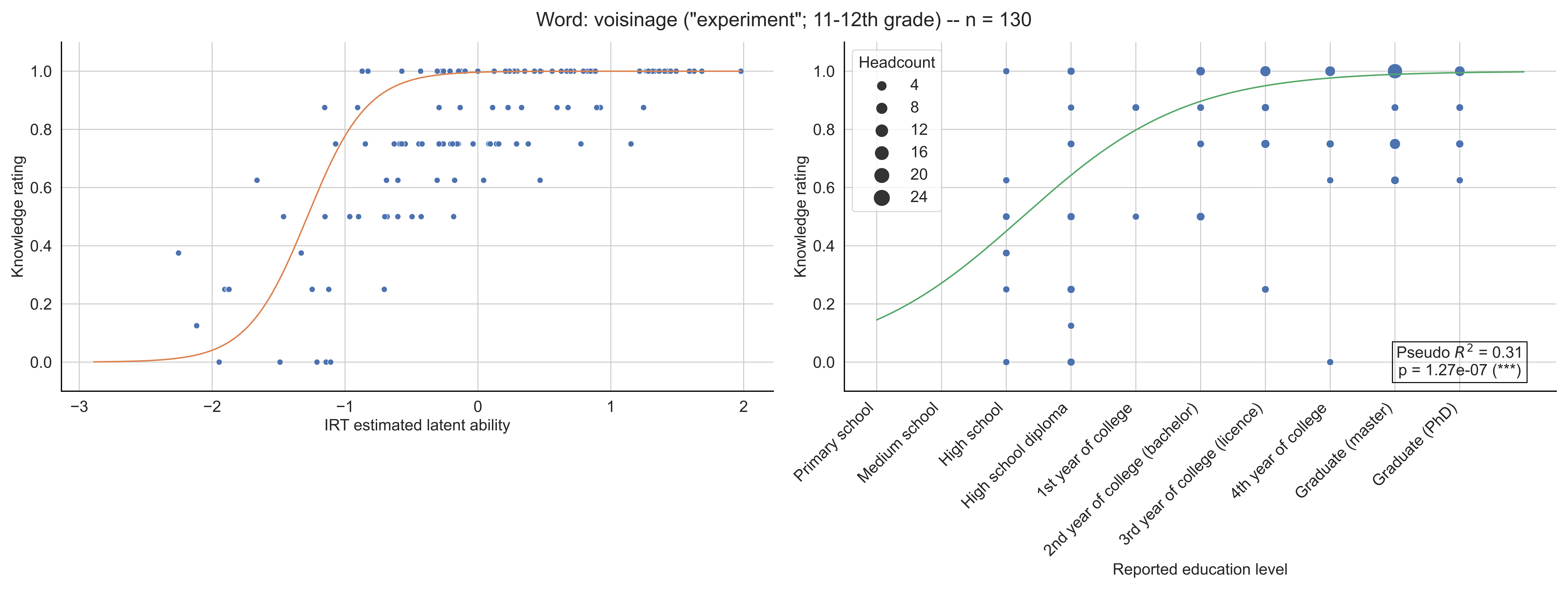

Word: voisinage ("experiment"; 11-12th grade) -- n = 130
Knowledge rating
IRT estimated latent ability
Headcount
4
8
12
16
20
24
Pseudo R^2 = 0.31
p = 1.27e-07 (***)
Primary school
Medium school
High school
High school diploma
1st year of college
2nd year of college (bachelor)
3rd year of college (licence)
4th year of college
Graduate (master)
Graduate (PhD)
Reported education level

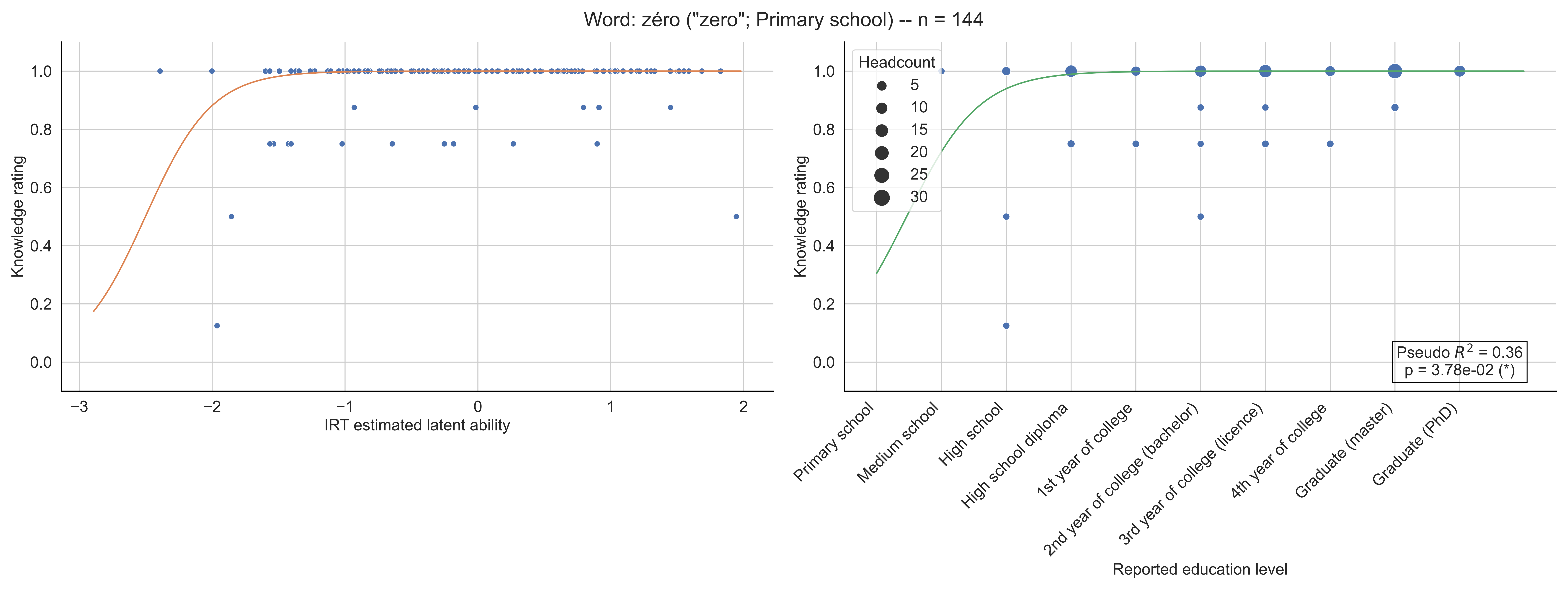

Word: zéro ("zero"; Primary school) -- n = 144
Knowledge rating
IRT estimated latent ability
Headcount
5
10
15
20
25
30
Pseudo R^2 = 0.36
p = 3.78e-02 (*)
Primary school
Medium school
High school
High school diploma
1st year of college
2nd year of college (bachelor)
3rd year of college (licence)
4th year of college
Graduate (master)
Graduate (PhD)
Reported education level

Word: élément ("element"; 10th grade) -- n = 132

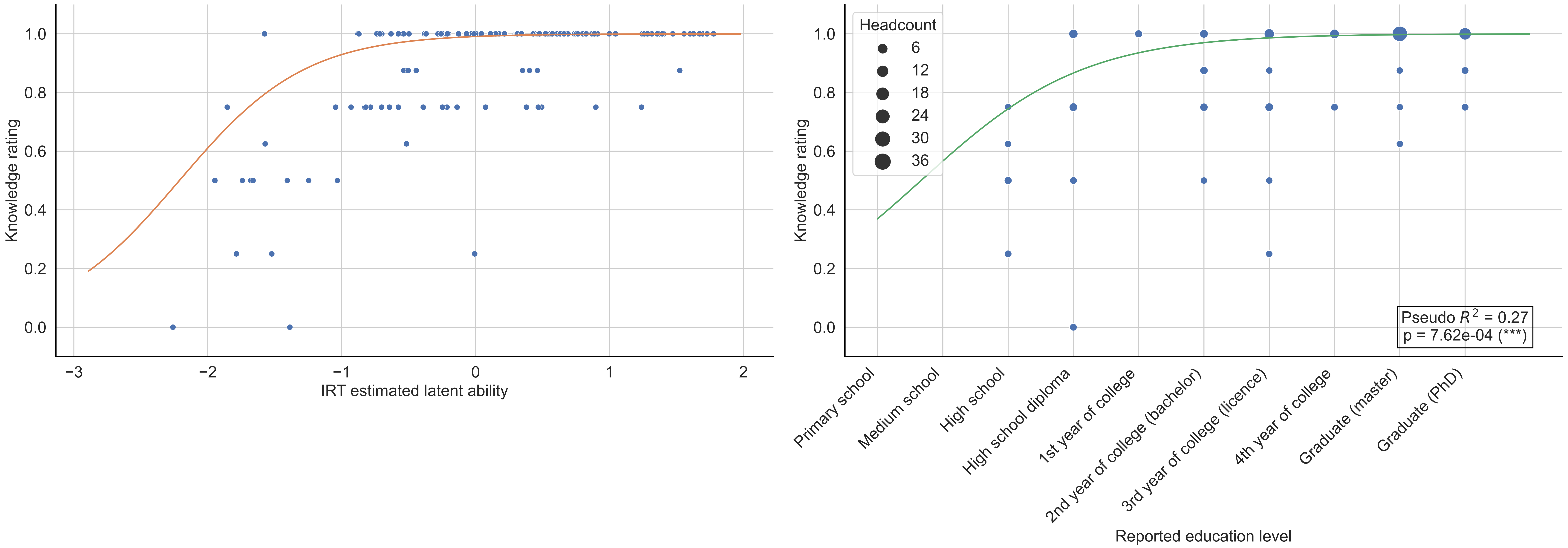

Word: étoile ("star"; Primary school) -- n = 140

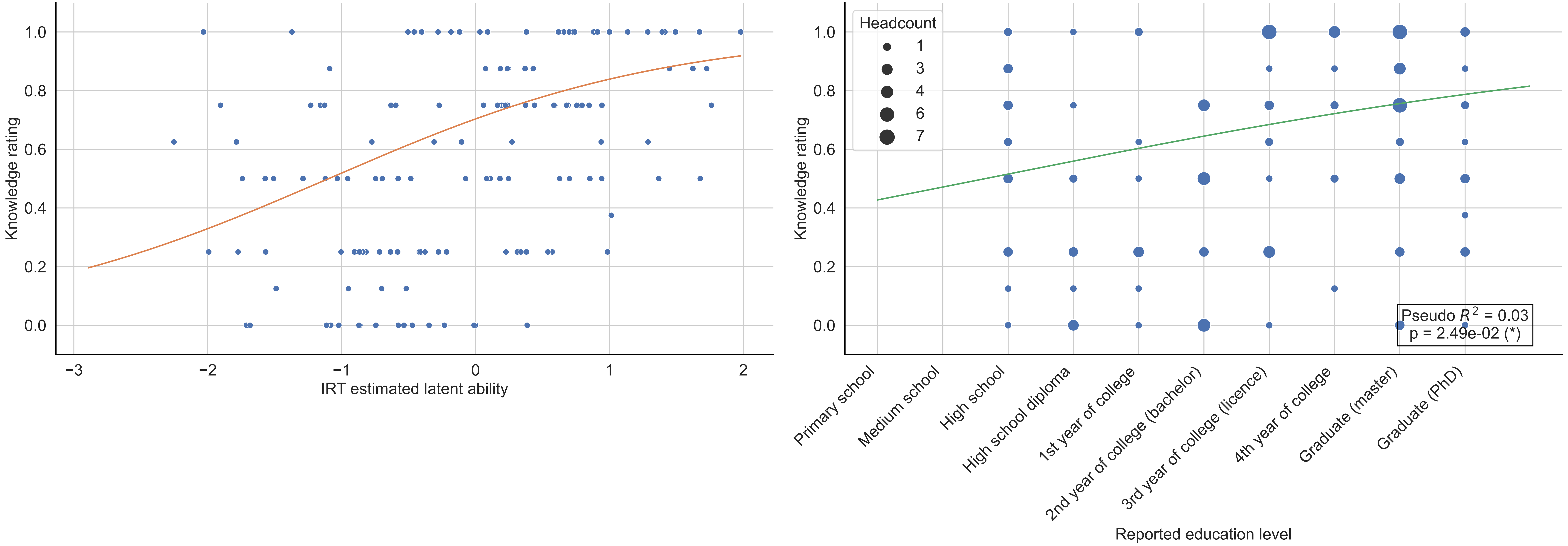

Word: évaluation ("evaluation"; Bachelor) -- n = 159

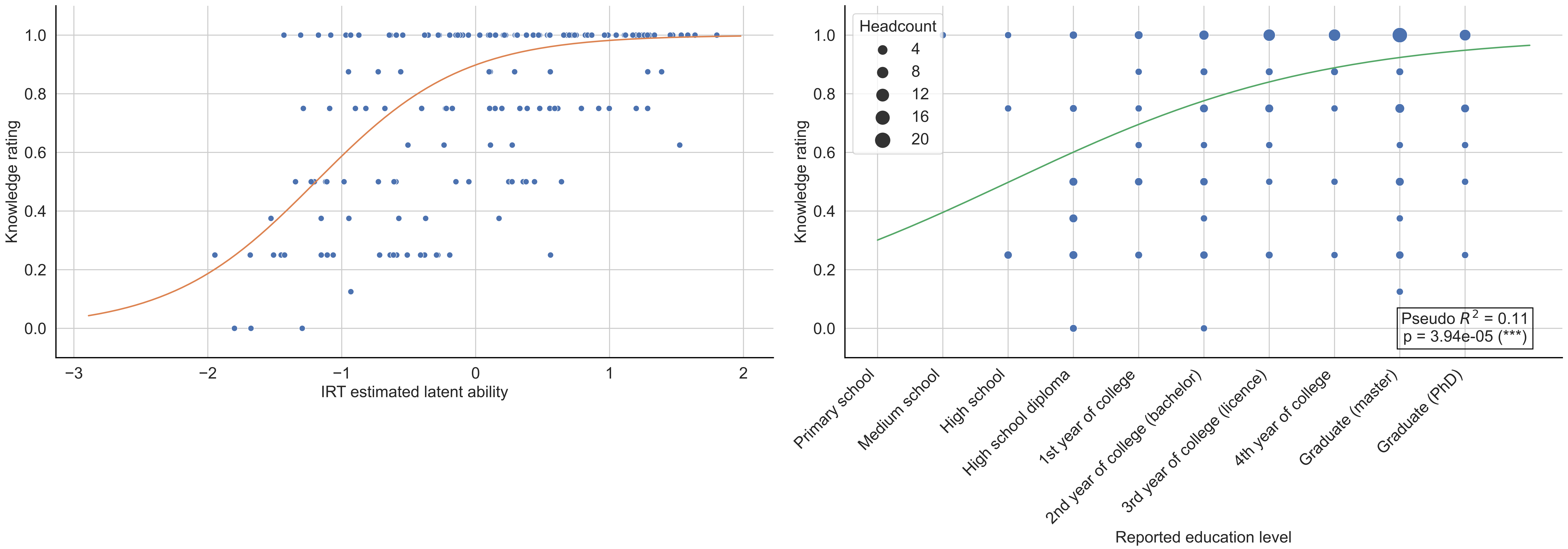

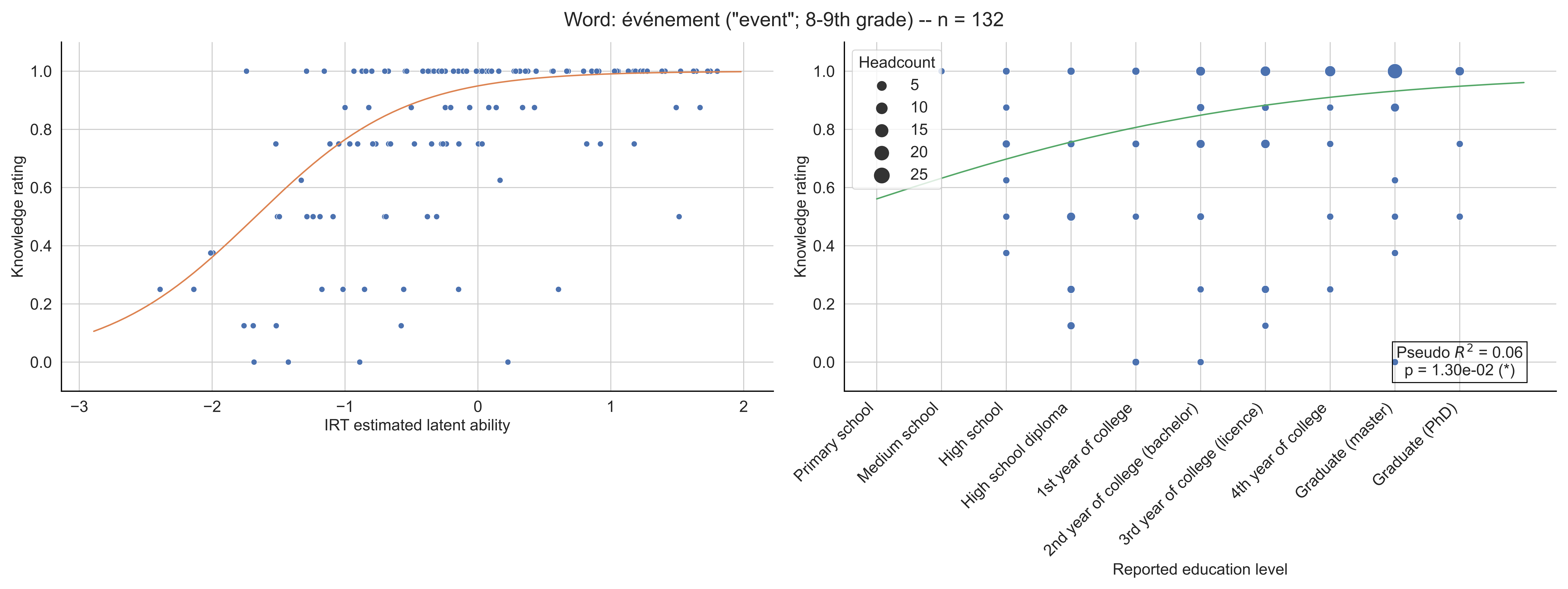
Word: événement ("event"; 8-9th grade) -- n = 132
Knowledge rating
IRT estimated latent ability
Headcount
5
10
15
20
25
Pseudo R^2 = 0.06
p = 1.30e-02 (*)
Primary school
Medium school
High school
High school diploma
1st year of college
2nd year of college (bachelor)
3rd year of college (licence)
4th year of college
Graduate (master)
Graduate (PhD)
Reported education level
