# Supplementary material for: Mapping and modeling the semantic space of math concepts
Source: Cognition. Author manuscript; Available in PMC 2025 Nov 26. (PMC7618406; doi:10.1016/j.cognition.2024.105971)
Supplement: Supplementary Material [file EMS210436-supplement-Supplementary_Material.zip › 1-s2.0-S0010027724002579-mmc2.docx]

## S2 Table. Number of pairs selected for the similarity rating task for each word grade.

| **Word grade** | **Number of pairs selected for the online experiment** |
| --- | --- |
| Primary school | 1,021 |
| 6-7th grade | 472 |
| 8-9th grade | 202 |
| 10th grade | 334 |
| 11-12th grade | 433 |
| bachelor | 789 |
| *licence* | 370 |
| master | 135 |
